# Supplementary material for: Chemoenzymatic Synthesis of Asymmetric Bisecting Bi‑, Tri‑, and Tetra-Antennary N‑Glycans
Source: J Am Chem Soc. 2025 Oct 1;147(41):37357–71. doi: 10.1021/jacs.5c11009 (PMC12532208; doi:10.1021/jacs.5c11009)
Supplement: Supplementary file 1 [file ja5c11009_si_001.pdf]

Supporting Information

Chemoenzymatic Synthesis of Asymmetric Bisecting Bi-, Tri-, and Tetra-Antennary *N*-Glycans

Balasaheb K. Ghotekar, Seema K. Bhagwat, Pradeep Chopra, Thomas Buckley, and Geert-Jan Boons*

Table of Contents

	page
1. Materials and Methods	S3
2. General Procedure for SGP Extraction and Trimming	S5
2a. Extraction of Sialyl Glycopeptide (SGP)	S5
2b. Enzymatic Modification of SGP to Prepare Glycosyl Asparagine-2-naphthyl methylcarbamate (**1**)	S6
3. General Protocols for Enzymatic Modification of Glycosyl Asparagine	S9
4. General Protocol for Purification	S13
5. Enzymatic Synthesis	S14
**Scheme S1**. Synthesis of bi-antennary *N*-glycans **2** and **3**	S14
**Scheme S2**. Synthesis of bi-antennary azido derivatives **17** and **18**	S14
**Scheme S3**. Attempt towards the synthesis of **S5-S8** using GnT-IVB and GnT-V	S14
**Scheme S4**. Synthesis of **S11** and **S12** from **3**	S15
**Scheme S5**. Synthesis of **S15** and **S16** from **2**	S15
**Scheme S6**. Synthesis of **S19**, **S20**, and **S21**	S16
6. Condition for Liquid Chromatography (LC) Trace Analysis	S17
7. NMR Nomenclature	S18
8. Synthesis and Characterization of *N*-Glycans	S19
9. Kinetic Parameters of GlcNAc Transfer GnT-III, GnT-IV and GnT-V for Multi-antennary Glycans (including **Figure S63**)	S117
10. Glycan Microarray Development	S119
11. References	S122
12. NMR Spectra	S123

1. Materials and Methods

All Chemical reagents were purchased from Sigma-Aldrich (unless noted) and used without further purification. Egg yolk powder (catalogue# 40504) was purchased from Natural Foods Inc, *Clostridium perfringens* Neuraminidase (catalogue# P0720) was obtained from New England Biolabs and Pronase from *Streptomyces griseus* (catalogue# P5147-1G) was purchased from Sigma-Aldrich. Reverse phase column chromatography was performed on Bondapak C-18 (Acetonitile:10mM ammonium bicarbonate or Water as eluent). Size exclusion chromatography was carried out on a Bio-Gel P-2 or G-50 gel column using 100 mM ammonium bicarbonate as eluant. HILIC-HPLC purification of compounds was performed on a Shimadzu 20AD UFLC LCMS-IT-TOF with a Waters XBridge BEH, Amide column, 5 µm, 10 x 250 mm. HPLC grade acetonitrile and water were purchased from Fischer. ^{1}H and ^{13}C NMR spectra were recorded on Bruker 600 or 900 MHz spectrometer. Signals are reported in terms of chemical shift [δ in parts per million (ppm)] relative to tetramethylsilane (TMS) as the internal standard. NMR data is presented as follows: Chemical shift, multiplicity (s = singlet, br. s = broad singlet, d = doublet, t = triplet, dd = doublet of doublet, m = multiplet and/or multiple resonances), coupling constant in Hertz (Hz), integration. All NMR signals were assigned on the basis of 1D: ^{1}H NMR, ^{13}C NMR and 2D: COSY, HSQC, TOCSY, HMBC, NOESY experiments. Mass spectras were recorded on Shimadzu LC/MS-IT-TOF mass spectrometer. [‡]Carbon assignments were extracted from HSQC experiment.

All enzymatic reactions were performed in aqueous buffers at the appropriate pH for each enzyme. Recombinant human glycosyl transferases including α-1,3-mannosyl-glycoprotein 2-β-*N*-acetylglucosaminyltransferase (GnT-I), α-1,6-mannosyl-glycoprotein 2-β-*N*-acetylglucosaminyltransferase (GnT-II), alpha-1,3-mannosyl-glycoprotein 4-beta-*N*-acetylglucosaminyltransferase B (GnT-IVB), alpha-1,6-mannosylglycoprotein 6-beta-*N*-acetylglucosaminyltransferase (GnT-V), beta-1,4-mannosyl-glycoprotein 4-beta-*N*-acetylglucosaminyltransferase (GnT-III), β-1,4-galactosyltransferase 1 (B4GalT1), β-galactoside-α-2,6-sialyltransferase 1 (ST6Gal1), and β-galactoside-α-2, 3-sialyltransferase 4 (ST3Gal4) Fucosyltransferase 8 (FUT8), Fucosyltransferase 6 (FUT6) were provided by Dr. K. W. Moremen (Complex Carbohydrate Research Center, Athens, GA, USA) which were expressed according to published protocols.[1-4] β-*N*-acetylglucosaminidase S was purchased from New England Biolab

(Catalog # P0744S). Alkaline phosphatase from calf intestine (CIAP) and bovine serum albumin (BSA) were purchased from Sigma-Aldrich. Uridine 5′-diphospho-*N*-acetylglucosamine (UDP-GlcNAc) was purchased from Sigma-Aldrich. Uridine 5′-diphospho-galactose diphosphate galactose (UDP-Gal) and cytidine 5′monophospho-N-acetylneuraminc acid (CMP-Neu5Ac) were both purchased from Roche. Guanosine. 5'-diphospho-β-L-fucose (GDP-Fuc) was purchased from Carbosynth.Uridine 5′-diphospho-*N*-trifluoroacetylglucosamine (UDP-GlcNTFA) was synthesized utilizing a one-pot three-enzyme combination as previously reported.[4]

2. General Procedure for SGP Extraction and Trimming

2a. Extraction of Sialyl Glycopeptide (SGP)

SGP was obtained from commercially available egg yolk powder following and modifying our previously reported procedure.[5] Briefly, an acetone suspension (2.0 L) of egg yolk powder (1.0 kg) was stirred at room temperature to remove lipids and other organic soluble components. After 2 h, the filtrate was discarded, and the insoluble powder was re-suspended in acetone and the same process was repeated again. Next, solid residue obtained was re-suspended with aq. acetone (70% v/v, 2.0 L), stirred for 2 h, filtered again, filtrate was discarded. Next, solid residue obtained was re-suspended with aq. acetone (40% v/v, 2.0 L), stirred for 2 h, and filtered. The insoluble material was discarded, and the filtrate was concentrated under reduced pressure at 40 °C. The collected liquid was concentrated in vacuo, transferred to centrifugal tubes, and kept at 0 °C. After 12 h, tubes were centrifuged (10 min, at 3500 rpm) to remove solid insoluble material. The clear liquid was transferred to fresh centrifugal tubes and diluted with dichloromethane (20 mL, DCM) and centrifuged again (10 min, at 3500 rpm). The aqueous layer was collected, concentrated under vacuum to minimum volume which was loaded on to a Bio-Gel G50 column SEC column. For elution 0.1 M ammonium bicarbonat e was employed and the desired fractions were pooled and concentrated in vacuo to yield pure SGP as a fluffy, white powder (0.9 g SGP/kg of egg yolk powder). The purity of SGP was determined by liquid chromatography-mass spectrometry (LC-MS) analysis on a Shimadzu LC/MS-IT-TOF mass spectrometer using a Xbridge amide analytical column (3.5 µm, 2.1 x 150 mm) with linear gradient of 80-50% solvent B (100% acetonitrile) in A (100% water) over a 60 min period at a flow rate of 0.25 mL/min.

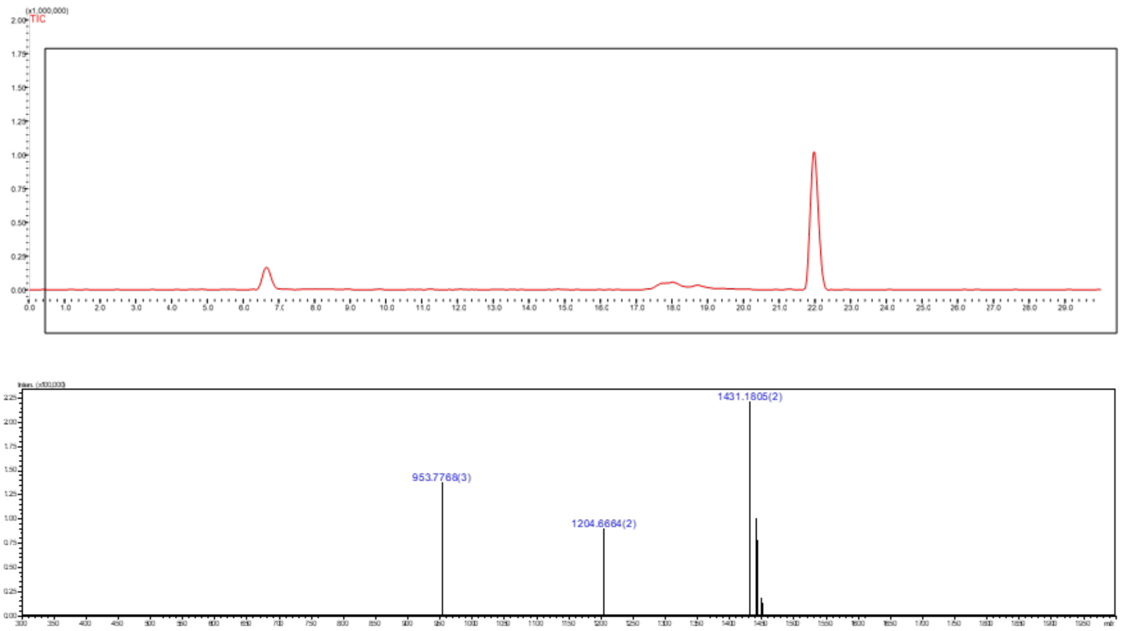

No.	Structure	Observed mass
1.	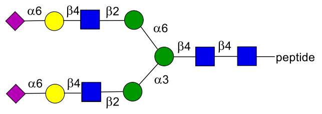	1431.1805(2)<br>953.7768(3)
2.	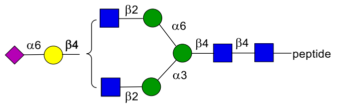	1204.6664(2)<br>Secondary ion fragmentation

Figure S1. Liquid chromatography (LC) trace and mass (ESI-MS) spectrum (peak at retention time = 22.0 min) of sialylglycopeptide (SGP).

2b. Enzymatic Modification of SGP to Prepare Glycosyl Asparagine-2-naphthyl Methylcarbamate (1)

To a solution of a sialoglycopeptide (SGP, 10 mM, 1.1 g) in sodium acetate buffer (50 mM, pH 5.5) containing $CaCl_2$ (5 mM) was added neuraminidase from *Clostridium perfringens* ((New England Biolabs # P0720L, 160 µL, 2000 units), and the mixture was incubated at 37 °C for 18 h. once ESI-MS shows completion of reaction, the pH of the reaction mixture was adjusted to 5 with acetic acid after which, β-galactosidase (800 µL, 800 units) from *Aspergillus niger* (Megazyme # E-BGLAN) were added. The reaction was incubated at 37 °C with shaking overnight, after which

another 200 μL of *β*-galactosidase were added. There are no any substrates in reaction solution and full galactose removal was monitored by ESI-MS, after which an equal volume of cooled alcohol was added and kept at 0 °C to precipitate protein residuals. The supernatant liquid was concentrated in vacuum and purified by size-exclusion chromatography using G-50 BioGel eluting with a 100 mM ammonium bicarbonate solution. Product **S1** containing fractions were lyophilized to obtain the desired product in the form of a white, amorphous solid.

Peptide attached *N*-glycan **S1** (500 mg) was dissolved in 2.5 mL of Tris buffer (100 mM, pH 8.0) containing 5 mM $CaCl_2$ with final reaction concentration of 10 mM. Pronase from *Streptomyces griseus* (SigmaAldrich # P5147-1G, 200 mg) was added, and the reaction was incubated for 3 days at 50 °C. Following an 3 days incubation period at 50 °C, portion of 120 mg Pronase was added. The reaction was monitored by ESI-MS, until there is no starting material remains. Equal amount of Ethanol was added into the reaction mixture, shake, and kept at 0 °C to precipitate protein residuals. Thereafter, supernatant liquid concentrated under reduced pressure at 40 °C and purified by size-exclusion chromatography using P2 BioGel eluting with a 100 mM ammonium bicarbonate solution. Product **S2** containing fractions were combined and lyophilized to afford the desired product **S2** as a white amorphous solid. Next, compound **S2** (0.2 g, 0.139 mmol) was dissolved in 1,4-Dioxane: H_2O (1:1, 14 mL) and 2-naphthylmethyl (NAP) chlorocarbonate (92 mg, 0.419 mmol), Sodium bicarbonate (0.12 g, 1.39 mmol) were added at room temparature for 16 h. After complete consumption of starting material (Monitored by TLC and LC-MS), concentrated and purified by size-exclusion chromatography using P2 BioGel eluting with a 100 mM ammonium bicarbonate solution to afford **S3** as white amorphous solid. The fractions containing the trimmed Compound **S3** were pooled, lyophilized, and dissolved in 10 mL of Tris buffer (100 mM, pH 7.5). To this mixture BSA (1 mg), calf intestine alkaline phosphatase (CIAP, 100 μL, 2kU/mL), $MgCl_2$ (10mM), GDP-Fucose (0.2 g), and FUT8 (200 μL, 1 mg/mL) were added and the reaction was incubated overnight at 37 °C with shaking. The reaction was lyophilized and purified by size-exclusion chromatography using P-2 BioGel eluting with a 0.1 M ammonium bicarbonate solution. The fractions containing **1** were pooled, lyophilized, and subjected to C18 followed by P-4 (extrafine, <45 μm) Biogel size-exclusion column chromatography purification according to the general protocol (0.125 g, 52% over 2 steps).

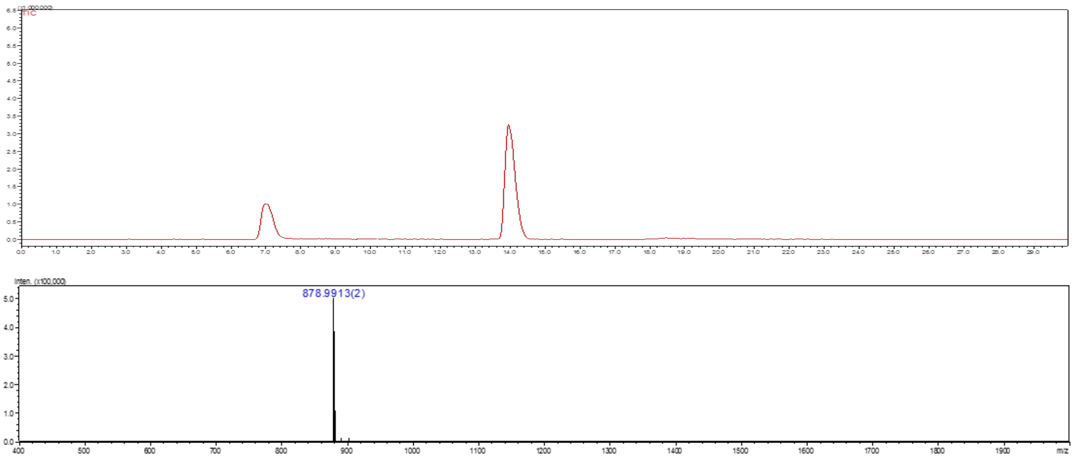

Figure S2. Liquid chromatography (LC) trace and mass (ESI-MS) spectrum (peak at retention time = 13.5 min) of **1**.

3. General Protocols for Enzymatic Modification of Glycosyl Asparagine

3a. General Procedure for the Installation of β1,2-GlcNAc or β1,2-GlcNTFA using GnT-I

Asparagine acceptor (1.0 equiv) and UDP-GlcNAc or UDP-GlcNTFA (1.5 equiv) were dissolved in a MES buffer (100 mM, pH 6.5) containing $MnCl_2$ (10 mM) keeping a final acceptor concentration of 10 mM. Recombinant GnT-I (50 µg/µmol acceptor) were successively added, and the reaction mixture was incubated overnight at 37 °C with shaking. Reaction progress was monitored by LC-ESI-MS and another portion of GnT-I was added if starting material remained after 18 h. The product was purified by C18 followed by P-4 (extrafine, <45 µm) Biogel size-exclusion column chromatography according to the general protocol.

3b. General Procedure for the Installation of β1,2-GlcNAc or β1,2-GlcNTFA using GnT-II

Acceptor (1.0 equiv) and UDP-GlcNAc or UDP-GlcNTFA (1.5 equiv) were dissolved in a MES buffer (100 mM, pH 6.5) containing $MnCl_2$ (10 mM) to provide a final acceptor concentration of 10 mM. GnT-II (50 µg/µmol acceptor) were successively added, and the mixture solution was incubated overnight at 37 °C with shaking. Reaction progress was monitored by ESI-MS and if starting material remained after 18 h another portion of GnT-II was added until no starting material could be detected. The product was purified by C18 followed by P-4 (extrafine, <45 µm) Biogel size-exclusion column chromatography according to the general protocol.

3c. General Procedure for the Installation of β1,4-GlcNAc using GnT-III

Asparagine acceptor (1.0 equiv) and UDP-GlcNAc (1.5 equiv) were dissolved in a MES buffer (100 mM, pH 6.5) containing $MnCl_2$ (2 mM), and BSA (1% total volume, stock solution = 10 mg/mL) to provide a final acceptor concentration of 10 mM. GnT-III (50 µg/µmol acceptor) were successively added, and the mixture solution was incubated overnight at 37 °C with shaking. Reaction progress was monitored by ESI-MS and if starting material remained after 18 h another portion of GnT-III was added until no starting material could be detected. The product was purified by C18 followed by P-4 (extrafine, <45 µm) Biogel size-exclusion column chromatography according to the general protocol.

3d. General Procedure for the Installation of β1,4-GlcNAc using GnT-IVB

Acceptor (1.0 equiv) and UDP-GlcNAc (1.5 equiv) were dissolved in a Tris buffer (100 mM, pH 7.5) containing $MnCl_2$ (5 mM), and BSA (1% total volume, stock solution = 10 mg/mL) to provide a final acceptor concentration of 10 mM. CIAP (1% total volume, stock solution = 1 kU/mL) and GnT-IVB (50 μg/μmol acceptor) were successively added, and the mixture solution was incubated overnight at 37 °C with shaking. Reaction progress was monitored by ESI-MS and if starting material remained after 18 h another portion of GnT-IVB was added until no starting material could be detected. The product was purified by C18 followed by P-4 (extrafine, <45 μm) Biogel size-exclusion column chromatography according to the general protocol.

3e. General Procedure for the Installation of β1,6-GlcNAc or β1,6-GlcNTFA using GnT-V

Asparagine acceptor (1.0 equiv) and UDP-GlcNAc or UDP-GlcNTFA (1.5 equiv) were dissolved in a sodium cacodylate buffer (100 mM, pH 6.5) containing MnCl2 (2 mM), and BSA (1% total volume, stock solution = 10 mg/mL) to provide a final acceptor concentration of 10 mM. CIAP (1% total volume, stock solution = 1 kU/mL) and GnT-V (50 μg/μmol acceptor) were successively added, and the mixture solution was incubated overnight at 37 °C with shaking. Reaction progress was monitored by ESI-MS and if starting material remained after 18 h another portion of GnT-V was added until no starting material could be detected. The product was purified by C18 followed by P-4 (extrafine, <45 μm) Biogel size-exclusion column chromatography according to the general protocol.

3f. General Procedure for the Installation of β1,4 Gal using B4GalT1

Glycosyl asparagine acceptor (1 equiv) and UDP-Gal (1.5 equiv per Gal to be added) were dissolved to prepare an reaction concentration of 5 mM in a Tris buffer (100 mM, pH 7.5) containing $MnCl_2$ (10 mM) and BSA (1% total volume, stock solution = 10 mg/mL). CIAP (1% total volume, stock solution = 1 kU/mL) and B4GalT1 (0.5% wt/wt relative to acceptor substrate) were added, and the reaction mixture was incubated overnight at 37 °C with gentle shaking. Reaction progress was monitored by LC-ESI-MS, and if starting material remained after 18 h another portion of B4GalT1 was added until no starting material could be detected. The product was purified by C18 followed by P-4 (extrafine, <45 μm) Biogel size-exclusion column chromatography according to the general protocol.

3g. General Procedure for the Installation of α2,3 Neu5Ac using ST3Gal4 or ST6Gal1

Asparagine acceptor (1 equiv) and CMP-Neu5Ac (1.5 equiv per Neu5Ac to be added) were dissolved at a final acceptor concentration of 5 mM in a sodium cacodylate buffer (50 mM, pH 7.2) containing BSA (1% total volume, stock solution = 10 mg/mL). CIAP (1% total volume, stock solution = 1 kU/mL) and ST3Gal4 or ST6Gal1 (1% wt/wt relative to acceptor substrate) were added, and the reaction mixture was incubated overnight at 37 °C with gentle shaking. Reaction progress was monitored by ESI-TOF MS, and if starting material remained after 18 h another portion of ST3Gal4 or ST6Gal1 was added until no starting material could be detected. The product was purified by C18 followed by P-4 (extrafine, <45 μm) Biogel size-exclusion column chromatography according to the general protocol.

3h. General Procedure for the Installation of α1,3 Fuc using FUT6

Acceptor (1 equiv) and GDP-Fuc (1.5 equiv per Fuc to be added) were dissolved at a final acceptor concentration of 5 mM in a Tris buffer (50 mM, pH 7.3) containing $MnCl_2$ (10 mM). CIAP (1% total volume, stock solution = 1 kU/mL) and FUT6 (1% wt/wt) were added, and the reaction mixture was incubated overnight at 37 °C with gentle shaking. Reaction progress was monitored by ESI-TOF MS, and if starting material remained after 18 h another portion of FUT6 was added until no starting material could be detected. The product was purified by C18 followed by P-4 (extrafine, <45 μm) Biogel size-exclusion column chromatography according to the general protocol.

3i. General Procedure for Removal of TFA Group of *N*-Glycans

N-Glycan was dissolved in deionized water with the final reaction concentration of 5 mM. The pH of the reaction solution was adjusted to 10 using NaOH (1 M), and the mixture solution was incubated for 3-5 h at 37 °C with shaking. When complete, the reaction was neutralized by aqueous acetic acid (1 M). The product was first purified by C18 biogel [Eluent – 10mM ammonium bicarbonate and water:acetonitrile (8:2)] followed by P-4 (extrafine, <45 μm) Biogel size-exclusion column chromatography.

3j. General Procedure for Amine Acetylation of *N*-Glycans

The amine containing *N*-glycans were dissolved (1 equiv) in 50 mM of water. Solids of $NaHCO_3$ (10 equiv, pH 8.0) and Ac_2O (1.2 equiv) were added to convert the NH_2 moiety into NHAc moiety and the reaction mixture was gently mixed until all solids were dissolved and incubated for 1–2 h at 37 °C. The reaction progress was monitored by LC-ESI-MS and additional $NaHCO_3$ (depending upon pH) and Ac_2O was added if the starting material was remaining. The product was first purified by C18 biogel [Eluent - water and 10mM ammonium bicarbonate:acetonitrile (8:2)] followed by P-4 (extrafine, <45 μm) Biogel size-exclusion column chromatography.

3k. General Procedure for Removal of 2-Naphthyl methylcarbamate Group

N-Glycans were dissolved in a 50% tert-butanol/water solution (1 mg/mL substrate concentration). To this solution Pd/C (1 mg/2 mg of starting material) was added. The reaction was stirred vigorously under an atmosphere of hydrogen (1 atm) and monitored by LC-ESI-MS. Once the reaction had gone to completion, it was filtered through a Whatman syringe filter (0.2 μm) to remove the catalyst, and the filtrate was lyophilized to provide the final product.

3l. General Procedure for the Conversion of $GlcNH_2$ into $GlcN_3$

Intermediate $GlcNH_2$ glycans (5 μmol, 1 equiv) was dissolved in water (2 mL) and to this solution was added imidazole-1-sulfonyl azide hydrogen sulfate (50 μmol), K_2CO_3 (50 μmol) and catalytic $CuSO_4.5H_2O$. The reaction mixture was incubated overnight at 37 °C with gentle shaking. Reaction progress was monitored by MALDI-TOF MS. The reaction mixture was lyophilized and the salts were removed by C18 biogel [Eluent - water and water:acetonitrile (8:2)] followed by P-4 (extrafine, <45 μm) Biogel size-exclusion column chromatography.

4. General Protocol for Purification

The reaction mixture was centrifuged over a Nanosep® Omega ultrafiltration device (10 kDa MWCO) to remove proteins. The decanted solution was first loaded on C18 Biogel and washed with 8-10 mL H_2O to remove excess sugar nucleotides and further product was eluted using 10 mM ammonium bicarbonate (or H_2O for base sensitive TFA containing compound) : acetonitrile (8:2). Fractions containing product were collected and lyophilized. The lyophilized product was dissolved and loaded on P-4 (extrafine, <45 μm) Biogel size-exclusion column chromatography using 100 mM ammonium bicarbonate as eluent.

5. Enzymatic Synthesis

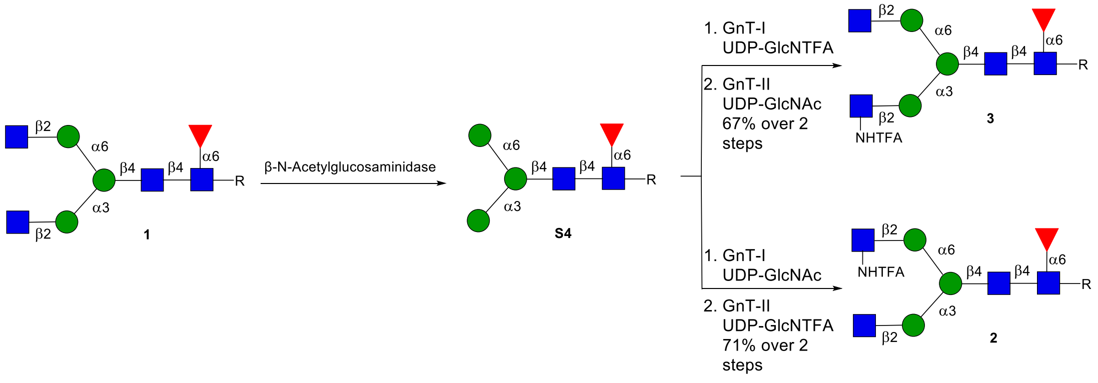

Scheme S1. Synthesis of bi-antennary *N*-glycans **2** and **3**.

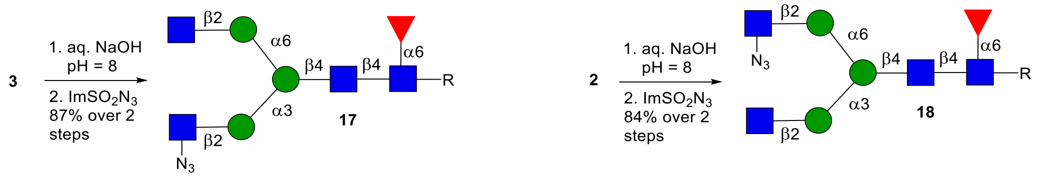

Scheme S2. Synthesis of bi-antennary azido derivatives **17** and **18**.

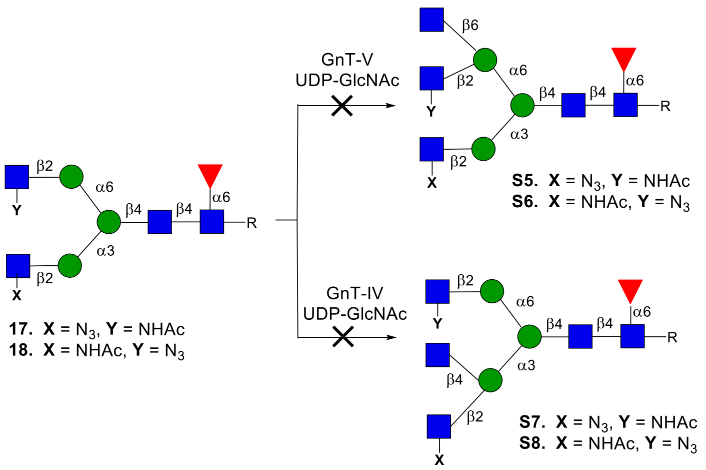

Scheme S3. Attempt towards the synthesis of **S5-S8** using GnT-IVB and GnT-V.

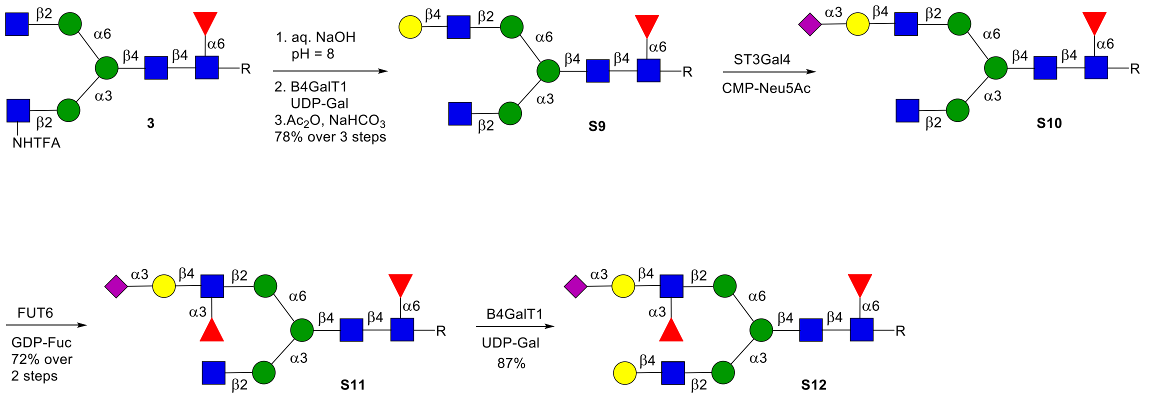

Scheme S4. Synthesis of asymmetric bi-antennary glycosyl asparagine **S11** and **S12** from **3**.

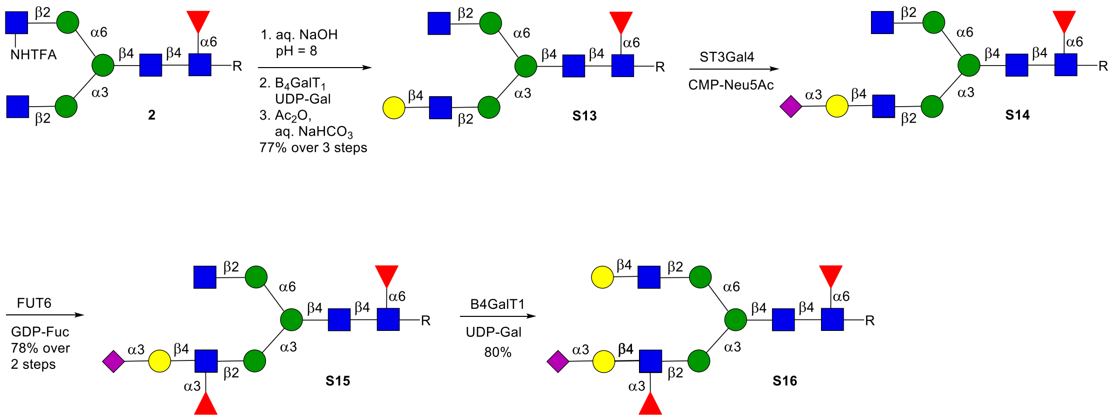

Scheme S5. Synthesis of asymmetric bi-antennary glycosyl asparagine **S15** and **S16** from **2**.

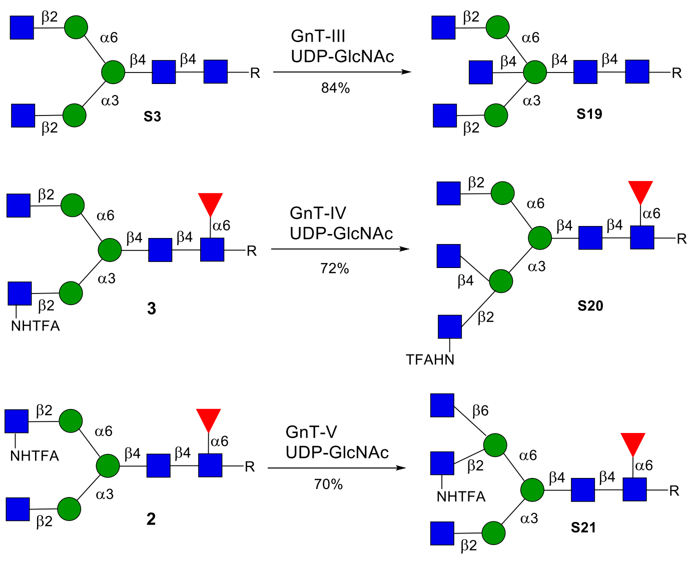

Scheme S6. Synthesis of glycosyl asparagine **S19**, **S20**, and **S21**.

6. Condition for Liquid Chromatography (LC) Trace Analysis

Liquid Chromatography (LC) was performed on a Shimadzu LC-ESI-IT-TOF with a Waters XBridge BEH, Amide column, 3.5 µm, 2.1 x 150 mm (Semi-preparative HILIC) at a flow rate of 0.16 mL/min, injection volume of 100-500 µL, with 1% of the flow is diverted to the ESI-MS detector using a splitter. Mobile phase A was 10 mM ammonium formate in water, adjusted to pH 3.5 with formic acid; mobile phase B was acetonitrile. The general condition using a linear gradient is as follows:

**Time (Min)**	**B %**	**A%**
0.01	80	20
18	50	50
20	25	75
24	25	75
26	80	20
30	80	20

The common peak in LC trace at retention time = 6 to 7 min is Salt peak from sodium and ammonium formate.

7. NMR Nomenclature

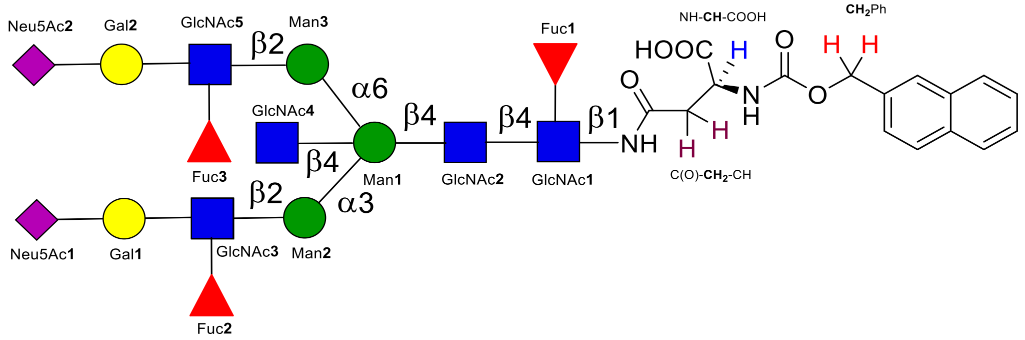

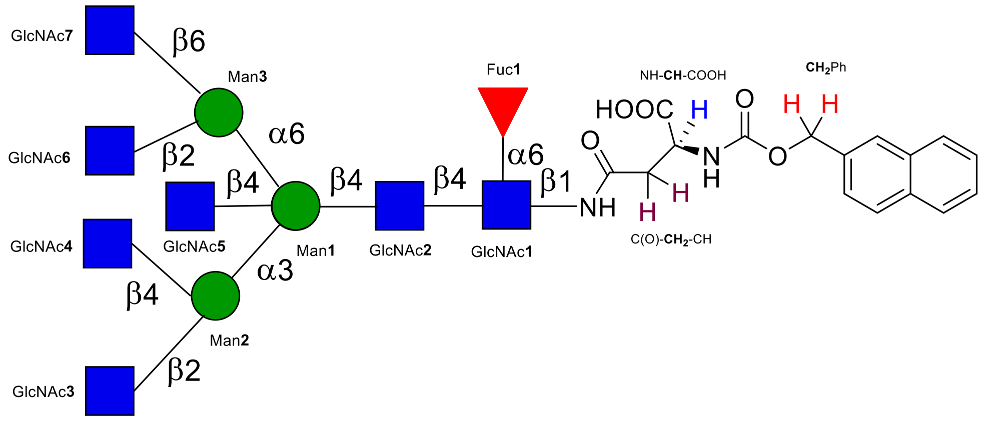

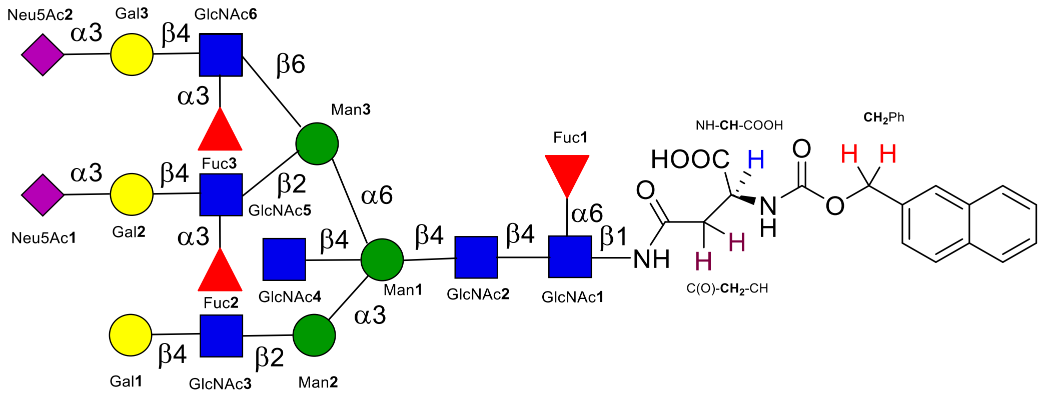

8. Synthesis and Characterization of *N*-Glycans

Compound 1

1 was synthesized from SGP (1.1 g, 401.2 μmol) following the general protocol **2b**.

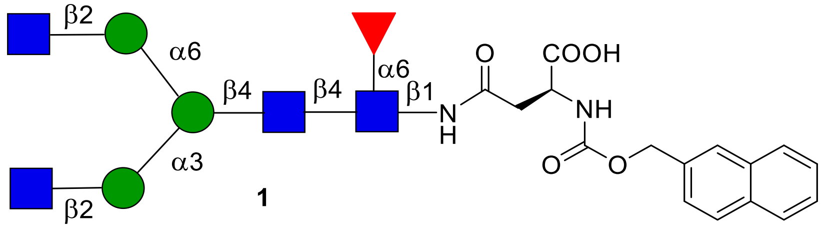

1

^{1}H NMR (600 MHz, D_2O): δ (ppm)

	H1	H2	H3	H4	H5	H6	NHAc
GlcNAc**1**	4.85	3.69	3.44	3.38	N/R[a]	N/R	2.02-1.79 (12H)
GlcNAc**2**	4.55	3.70	3.50	3.65	N/R	N/R	2.02-1.79 (12H)
GlcNAc**3**	4.47	3.63	3.47	3.38	N/R	N/R	2.02-1.79 (12H)
GlcNAc**4**	4.47	3.63	3.47	3.36	N/R	N/R	2.02-1.79 (12H)
Man**1**	4.70	4.17	3.69	3.54	N/R	N/R	-
Man**2**	5.03	4.10	3.82	3.42	3.54	N/R	-
Man**3**	4.84	4.03	3.81	3.42	3.54	N/R	-
Fuc**1**	4.72	3.65	3.72	3.60	3.94	1.05	-

Signal	Proton	Carbon
**Aromatic**	7.92-7.84 (m, 4H)<br>7.53-7.47 (m, 3H)	128.2-125.8
**CH_2**Ph	5.32 (d, J = 12.7 Hz)<br>5.14 (d, J = 12.6 Hz)	66.7
NH-**CH**-COOH	4.29 (dd, J = 9.5, 3.6 Hz)	53.0
C(O)-**CH_2**-CH	2.75 (dd, J = 14.8, 2.9 Hz)<br>2.51 (dd, J = 15.0, 9.9 Hz)	38.6

^{13}C NMR (150 MHz, D_2O): δ (ppm)

	**C1**
GlcNAc**1**	78.2
GlcNAc**2**	100.8
GlcNAc**3**	99.6
GlcNAc**4**	99.6
Man**1**	100.5
Man**2**	99.5
Man**3**	97.0
Fuc**1**	99.2

[a] Not reported

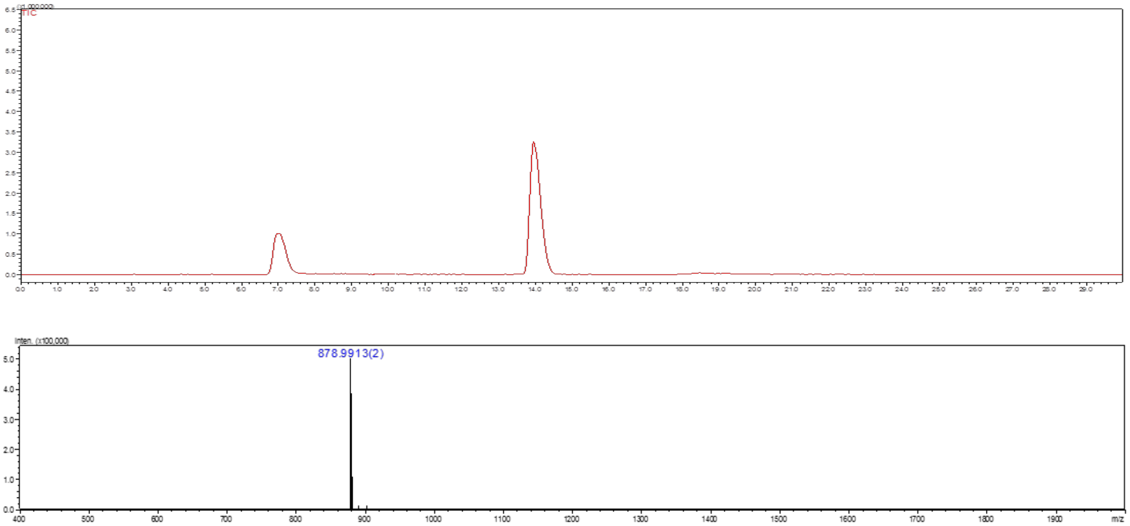

Figure S3. Liquid chromatography (LC) trace and mass (ESI-MS) spectrum (peak at retention time = 14.0 min) of **1**.

Compound 10

10 was synthesized from **1** (2 mg, 1.13 μmol) following the general protocol **3d** for the installation of GlcNAc moiety using UDP-GlcNAc. The product **10** was obtained as a white fluffy solid (1.8 mg, 81%).

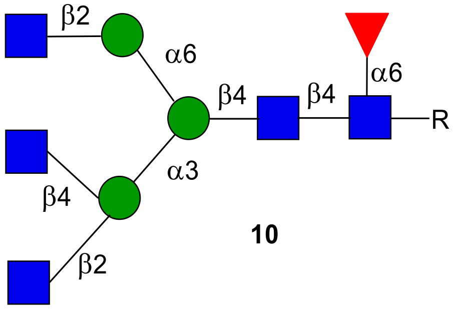

10

^{1}H NMR (600 MHz, D_2O): δ (ppm)

	**H1**	**H2**	**H3**	**H4**	**H5**	**H6**	**NHAc**
GlcNAc**1**	4.86	3.70	3.44	3.39	N/R[a]	N/R	2.03-1.80 (15H)
GlcNAc**2**	4.55	3.71	3.50	3.65	N/R	N/R	2.03-1.80 (15H)
GlcNAc**3**	4.45	3.68	3.44	3.41	N/R	N/R	2.03-1.80 (15H)
GlcNAc**4**	4.48	3.63	3.47	3.37	N/R	N/R	2.03-1.80 (15H)
GlcNAc**5**	4.48	3.63	3.47	3.37	N/R	N/R	2.03-1.80 (15H)
Man**1**	4.68	4.14	3.69	3.55	N/R	N/R	-
Man**2**	5.04	4.14	3.97	3.55	3.52	N/R	-
Man**3**	4.84	4.04	3.83	3.54	3.43	N/R	-
Fuc**1**	4.72	3.67	3.73	3.61	3.95	1.08	-

**Signal**	**Proton**	**Carbon**
**Aromatic**	7.93-7.85 (m, 4H)<br>7.54-7.48 (m, 3H)	128.4-125.7
**CH_2**Ph	5.34 (d, J = 12.8 Hz)<br>5.14 (d, J = 12.7 Hz)	66.9
NH-**CH**-COOH	4.30 (dd, J = 9.5, 3.6 Hz)	53.1
C(O)-**CH_2**-CH	2.75 (dd, J = 14.8, 2.9 Hz)<br>2.51 (dd, J = 15.0, 9.9 Hz)	38.6

^{13}C NMR (150 MHz, D_2O): δ (ppm)

	**C1**
GlcNAc**1**	78.2
GlcNAc**2**	100.8
GlcNAc**3**	99.6
GlcNAc**4**	101.7
GlcNAc**5**	99.6
Man**1**	100.3
Man**2**	99.0
Man**3**	97.0
Fuc**1**	99.2

[a] Not reported

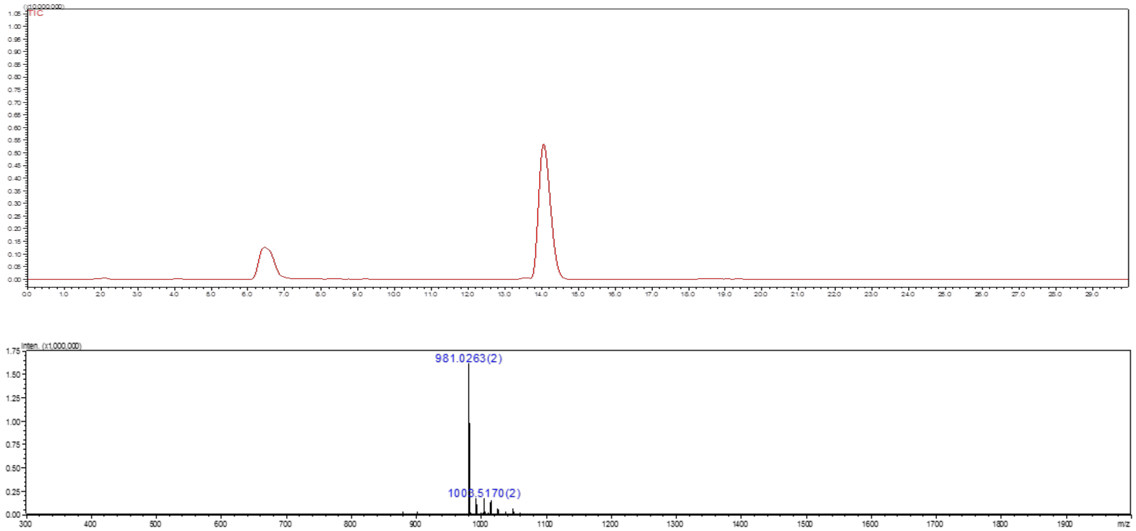

Figure S4. Liquid chromatography (LC) trace and mass (ESI-MS) spectrum (peak at retention time = 14.2 min) of **10**.

Compound 11

11 was synthesized from **1** (10 mg, 5.67 μmol) following the general protocol **3e** for the installation of GlcNAc moiety using UDP-GlcNAc. The product **11** was obtained as a white fluffy solid (10.1 mg, 91%).

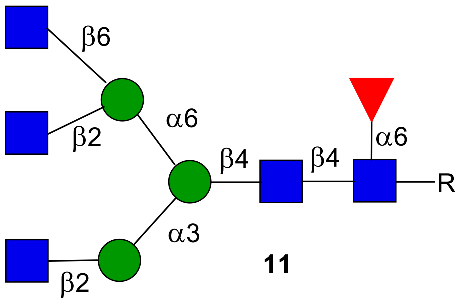

11

^{1}**H NMR (900 MHz, D_2O): δ (ppm)**

	H1	H2	H3	H4	H5	H6	NHAc
GlcNAc**1**	4.86	3.70	3.45	3.38	N/R[a]	N/R	2.02-1.79 (15H)
GlcNAc**2**	4.54	3.69	3.50	3.65	N/R	N/R	2.02-1.79 (15H)
GlcNAc**3**	4.48	3.59/3.64[b]	3.45/3.47[b]	3.36/3.38[b]	N/R	N/R	2.02-1.79 (15H)
GlcNAc**4**	4.48	3.59/3.64[b]	3.45/3.47[b]	3.36/3.38[b]	N/R	N/R	2.02-1.79 (15H)
GlcNAc**5**	4.46	3.64	3.49	3.39	N/R	N/R	2.02-1.79 (15H)
Man**1**	4.69	4.17	3.69	3.54	N/R	N/R	-
Man**2**	5.05	4.12	3.83	3.67	N/R	N/R	-
Man**3**	4.79	4.02	3.80	3.66	3.34	N/R	-
Fuc**1**	4.73	3.66	3.71	3.61	3.93	1.06	-

Signal	Proton	Carbon
**Aromatic**	7.91-7.85 (m, 4H)<br>7.53-7.47 (m, 3H)	128.3-125.7
**CH_2**Ph	5.33 (d, *J* = 12.8 Hz)<br>5.14 (d, *J* = 12.7 Hz)	66.8
NH-**CH**-COOH	4.29 (dd, *J* = 9.5, 3.6 Hz)	53.1
C(O)-**CH_2**-CH	2.75 (dd, *J* = 14.8, 2.9 Hz)<br>2.51 (dd, *J* = 15.0, 9.9 Hz)	38.6

^{13}C NMR (225 MHz, D_2O): δ (ppm)

	**C1**
GlcNAc**1**	78.2
GlcNAc**2**	100.8
GlcNAc**3**	99.7
GlcNAc**4**	99.6
GlcNAc**5**	101.6
Man**1**	100.4
Man**2**	99.5
Man**3**	97.1
Fuc**1**	99.2

[a] Not reported
[b] Could not be determined

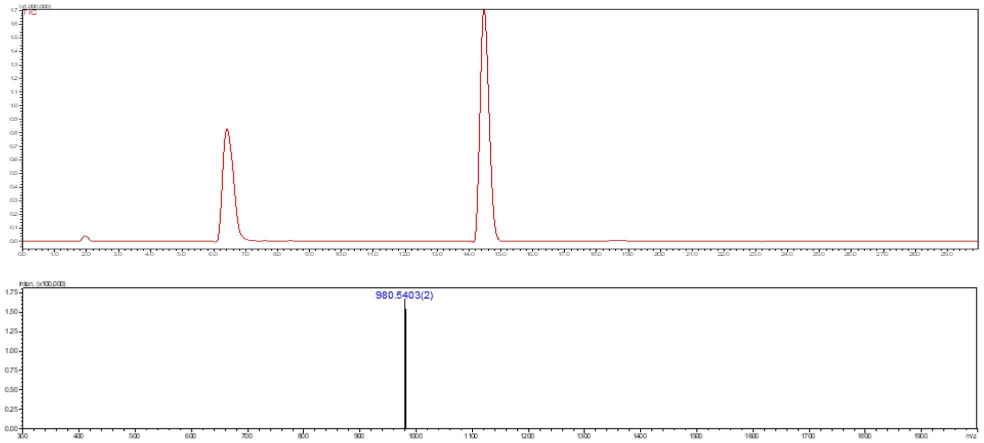

Figure S5. Liquid chromatography (LC) trace and mass (ESI-MS) spectrum (peak at retention time = 14.5 min) of **11**.

Compound 12

12 was synthesized from **11** (7 mg, 3.56 µmol) following the general protocol **3d** for the installation of GlcNAc moiety using UDP-GlcNAc. The product **12** was obtained as a white fluffy solid (7.7 mg, 93%).

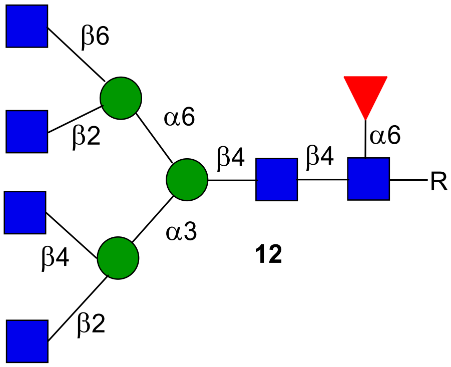

12

^{1}H NMR (600 MHz, D_2O): δ (ppm)

	H1	H2	H3	H4	H5	H6	NHAc
GlcNAc**1**	4.84	3.69	3.50	3.36	N/R[a]	N/R	2.01-1.78 (18H)
GlcNAc**2**	4.53	3.69	3.49	3.66	N/R	N/R	2.01-1.78 (18H)
GlcNAc**3**	4.48/4.47[b]	3.59/3.62[b]	3.48/3.49[b]	3.36/3.38[b]	N/R	N/R	2.01-1.78 (18H)
GlcNAc**4**	4.46	3.66/3.64[b]	3.50/3.49[b]	3.42	N/R	N/R	2.01-1.78 (18H)
GlcNAc**5**	4.48/4.47[b]	3.59/3.62[b]	3.48/3.49[b]	3.36/3.38[b]	N/R	N/R	2.01-1.78 (18H)
GlcNAc**6**	4.44	3.66/3.64[b]	3.50/3.49[b]	3.38	N/R	N/R	2.01-1.78 (18H)
Man**1**	4.69	4.15	3.70	3.41	N/R	N/R	-
Man**2**	5.02	4.13	3.96	3.53	3.72	3.50	-
Man**3**	4.77	4.00	3.78	3.66	N/R	N/R	-
Fuc**1**	4.71	4.65	3.70	3.60	3.94	1.05	-

Signal	Proton	Carbon
**Aromatic**	7.90-7.83 (m, 4H)<br>7.52-7.46 (m, 3H)	128.4-125.7
**CH_2**Ph	5.31 (d, J = 12.8 Hz)<br>5.12 (d, J = 12.7 Hz)	66.8

NH-**CH**-COOH	4.28 (dd, *J* = 9.5, 3.6 Hz)	53.1
C(O)-**CH_2**-CH	2.74 (dd, *J* = 14.8, 2.9 Hz)<br>2.50 (dd, *J* = 15.0, 9.9 Hz)	38.7

^{13}C NMR (150 MHz, D_2O): δ (ppm)

	**C1**
GlcNAc**1**	78.2
GlcNAc**2**	100.9
GlcNAc**3**	99.7
GlcNAc**4**	101.6
GlcNAc**5**	99.7
GlcNAc**6**	101.6
Man**1**	100.3
Man**2**	99.1
Man**3**	97.2
Fuc**1**	99.2

[a] Not reported
[b] Could not be determined

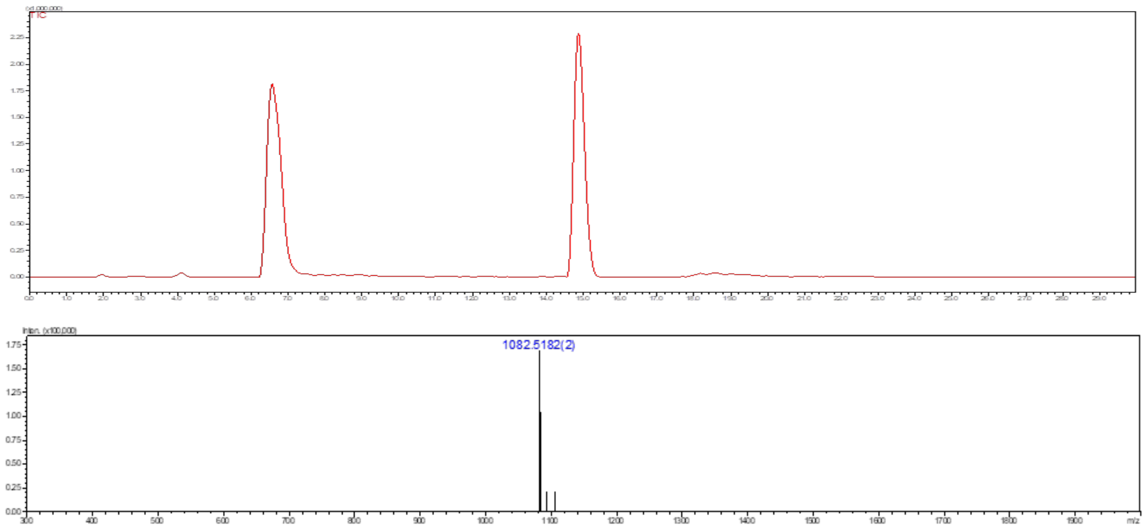

Figure S6. Liquid chromatography (LC) trace and mass (ESI-MS) spectrum (peak at retention time = 14.9 min) of **12**.

Compound 13

13 was synthesized from **10** (3 mg, 1.52 μmol) following the general protocol **3c** for the installation of GlcNAc moiety using UDP-GlcNAc. The product **13** was obtained as a white fluffy solid (3.1 mg, 93%).

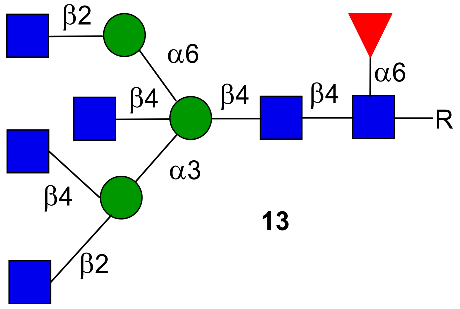

13

^{1}H NMR (900 MHz, D_2O): δ (ppm)

	**H1**	**H2**	**H3**	**H4**	**H5**	**H6**	**NHAc**
GlcNAc**1**	4.83	3.68	3.43	3.36	N/R[a]	N/R	2.01-1.78 (18H)
GlcNAc**2**	4.52	3.70	3.46	3.62	N/R	N/R	2.01-1.78 (18H)
GlcNAc**3**	4.45	3.62	3.49	3.38	N/R	N/R	2.01-1.78 (18H)
GlcNAc**4**	4.43	3.67	3.46	3.39	N/R	N/R	2.01-1.78 (18H)
GlcNAc**5**	4.37	3.60	3.31	3.18	3.48	N/R	2.01-1.78 (18H)
GlcNAc**6**	4.45	3.62	3.49	3.38	N/R	N/R	2.01-1.78 (18H)
Man**1**	4.59	4.06	3.76	N/R	N/R	N/R	-
Man**2**	4.96	4.19	3.96	3.49	N/R	N/R	-
Man**3**	4.90	4.06	3.75	3.46	N/R	N/R	-
Fuc**1**	4.71	3.65	3.70	3.59	3.92	1.04	-

**Signal**	**Proton**	**Carbon**
**Aromatic**	7.90-7.84 (m, 4H)<br>7.51-7.46 (m, 3H)	128.4-125.7
**CH_2**Ph	5.31 (d, J = 12.8 Hz)<br>5.12 (d, J = 12.7 Hz)	66.8
NH-**CH**-COOH	4.28 (dd, J = 9.5, 3.6 Hz)	53.1

C(O)-$\mathbf{CH_2}$-CH	2.74 (dd, J = 14.8, 2.9 Hz)<br>2.50 (dd, J = 15.0, 9.9 Hz)	38.6

^{13}C NMR (225 MHz, D_2O): δ (ppm)

	**C1**
GlcNAc**1**	78.2
GlcNAc**2**	100.9
GlcNAc**3**	99.8
GlcNAc**4**	101.6
GlcNAc**5**	100.5
GlcNAc**6**	99.8
Man**1**	100.1
Man**2**	99.4
Man**3**	97.6
Fuc**1**	99.1

[a] Not reported

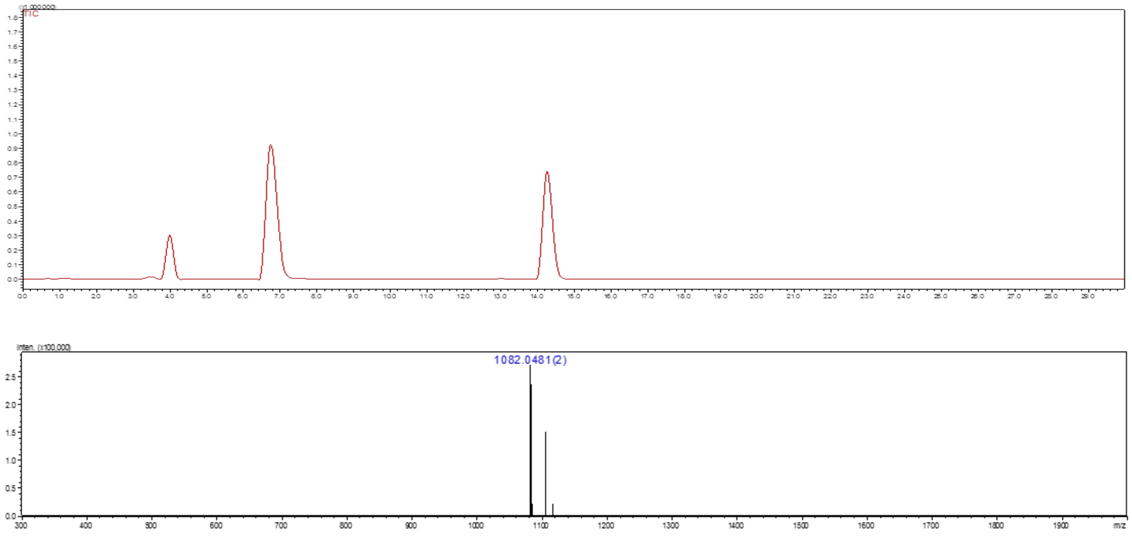

Figure S7. Liquid chromatography (LC) trace and mass (ESI-MS) spectrum (peak at retention time = 14.3 min) of **13**.

Compound 5

14 was synthesized from **11** (3 mg, 1.52 µmol) following the general protocol **3c** for the installation of GlcNAc moiety using UDP-GlcNAc. The product **14** was obtained as a white fluffy solid (2.7 mg, 82%).

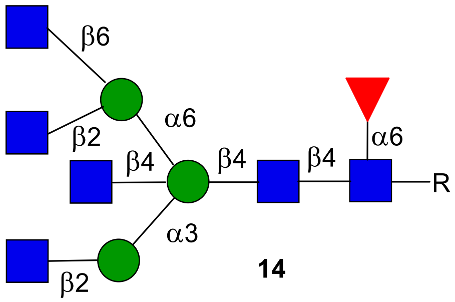

14

^{1}H NMR (900 MHz, D_2O): δ (ppm)

	**H1**	**H2**	**H3**	**H4**	**H5**	**H6**	**NHAc**
GlcNAc**1**	4.91	3.75	3.50	3.44	N/R[a]	N/R	2.08-1.85 (18H)
GlcNAc**2**	4.59	3.76	3.54	3.71	N/R	N/R	2.08-1.85 (18H)
GlcNAc**3**	4.56	3.71	3.48	N/R	N/R	N/R	2.08-1.85 (18H)
GlcNAc**4**	4.47	3.69	3.37	3.26	3.59	N/R	2.08-1.85 (18H)
GlcNAc**5**	4.54	3.65	3.44	3.36	N/R	N/R	2.08-1.85 (18H)
GlcNAc**6**	4.50	3.72	3.56	3.44	N/R	N/R	2.08-1.85 (18H)
Man**1**	4.68	4.15	3.84	3.51	N/R	N/R	-
Man**2**	5.04	4.22	3.88	3.46	3.57	N/R	-
Man**3**	4.89	4.11	3.79	3.41	3.53	N/R	-
Fuc**1**	4.79	3.71	3.76	3.66	4.00	1.10	-

**Signal**	**Proton**	**Carbon**
**Aromatic**	7.91-7.85 (m, 4H)<br>7.53-7.47 (m, 3H)	128.2-125.5
**CH_2**Ph	5.33 (d, J = 12.8 Hz)<br>5.14 (d, J = 12.7 Hz)	66.9
NH-**CH**-COOH	4.29 (dd, J = 9.5, 3.6 Hz)	53.1

C(O)-**CH$_2$**-CH	2.75 (dd, J = 14.8, 2.9 Hz)<br>2.51 (dd, J = 15.0, 9.9 Hz)	38.9

^{13}C NMR (225 MHz, D_2O): δ (ppm)

	**C1**
GlcNAc**1**	78.2
GlcNAc**2**	100.9
GlcNAc**3**	99.8
GlcNAc**4**	99.9
GlcNAc**5**	99.8
GlcNAc**6**	101.7
Man**1**	99.9
Man**2**	99.8
Man**3**	97.9
Fuc**1**	99.2

[a] Not reported

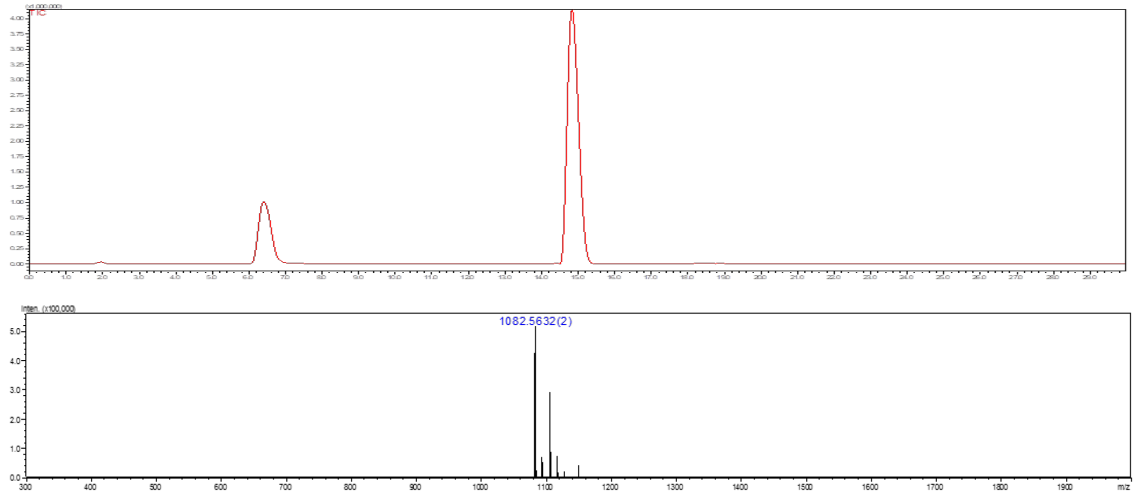

Figure S8. Liquid chromatography (LC) trace and mass (ESI-MS) spectrum (peak at retention time = 14.9 min) of **14**.

Compound 15

15 was synthesized from **12** (4 mg, 1.84 µmol) following the general protocol **3c** for the installation of GlcNAc moiety using UDP-GlcNAc. The product **15** was obtained as a white fluffy solid (3.9 mg, 91%).

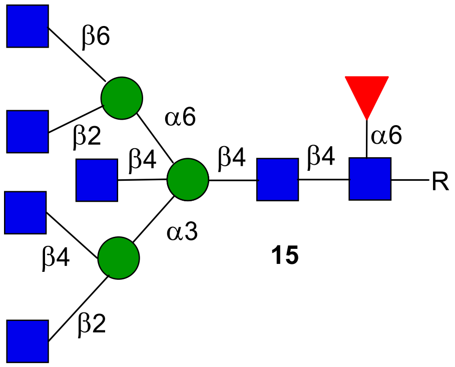

15

^{1}H NMR (600 MHz, D_2O): δ (ppm)

	**H1**	**H2**	**H3**	**H4**	**H5**	**H6**	**NHAc**
GlcNAc**1**	4.68	3.70	3.50	3.38	N/R[a]	N/R	2.02-1.79 (21H)
GlcNAc**2**	3.53	3.72	3.48	3.65	N/R	N/R	2.02-1.79 (21H)
GlcNAc**3**	4.50	3.64	3.52	3.41	N/R	N/R	2.02-1.79 (21H)
GlcNAc**4**	4.44	3.66	3.50	3.42	N/R	N/R	2.02-1.79 (21H)
GlcNAc**5**	4.41	3.61	3.31	3.21	3.53	N/R	2.02-1.79 (21H)
GlcNAc**6**	4.48	3.59	3.38	3.31	N/R	N/R	2.02-1.79 (21H)
GlcNAc**7**	4.43	3.68	3.51	3.39	N/R	N/R	2.02-1.79 (21H)
Man**1**	4.61	4.06	3.79	4.08	N/R	N/R	-
Man**2**	4.98	4.20	3.97	3.51	N/R	N/R	-
Man**3**	4.82	4.04	3.73	3.48	N/R	N/R	-
Fuc**1**	4.73	3.67	3.72	3.62	3.93	1.06	-

**Signal**	**Proton**	**Carbon**
**Aromatic**	7.91-7.85 (m, 4H)<br>7.53-7.47 (m, 3H)	128.4-125.7
**CH$_2$**Ph	5.32 (d, J = 12.8 Hz)	66.8

	5.14 (d, J = 12.7 Hz)	
NH-**CH**-COOH	4.29 (dd, J = 9.5, 3.6 Hz)	53.1
C(O)-**CH₂**-CH	2.75 (dd, J = 14.8, 2.9 Hz)<br>2.51 (dd, J = 15.0, 9.9 Hz)	38.6

^{13}C NMR (150 MHz, D_2O): δ (ppm)

	**C1**
GlcNAc**1**	78.2
GlcNAc**2**	100.9
GlcNAc**3**	99.9
GlcNAc**4**	101.7
GlcNAc**5**	99.8
GlcNAc**6**	99.9
GlcNAc**7**	101.7
Man**1**	99.8
Man**2**	99.5
Man**3**	98.0
Fuc**1**	99.2

[a] Not reported

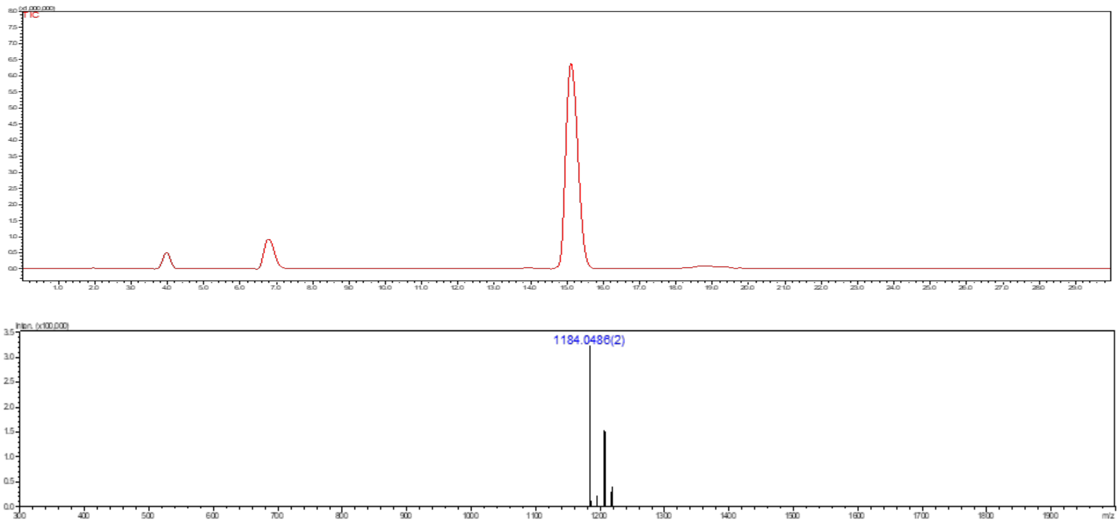

Figure S9. Liquid chromatography (LC) trace and mass (ESI-MS) spectrum (peak at retention time = 15.2 min) of **15**.

Compound 16

Compound **16** was synthesized from **1** (6 mg, 3.40 µmol) following the general protocol **3c** for the installation of GlcNAc moiety using UDP-GlcNAc. The product **16** was obtained as a white fluffy solid (5.6 mg, 88%).

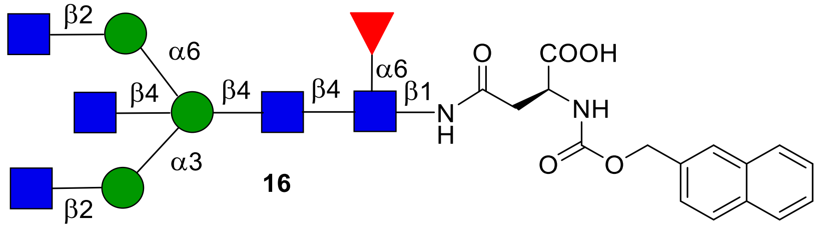

^{1}H NMR (600 MHz, D_2O): δ (ppm)

	H1	H2	H3	H4	H5	H6	NHAc
GlcNAc**1**	4.85	3.69	3.44	3.38	N/R[a]	N/R	2.02-1.79 (12H)
GlcNAc**2**	4.54	3.72	3.48	3.66	N/R	N/R	2.02-1.79 (12H)
GlcNAc**3**	4.48	3.65	3.50	3.41	N/R	N/R	2.02-1.79 (12H)
GlcNAc**4**	4.39	3.62	3.33	3.19	3.49	3.62	2.02-1.79 (12H)
GlcNAc**5**	4.47	3.63	3.50	3.41	N/R	N/R	2.02-1.79 (12H)
Man**1**	4.61	4.10	3.79	4.00	3.46	N/R	-
Man**2**	4.98	4.17	3.82	3.40	3.53	3.61	-
Man**3**	4.92	4.07	3.75	3.41	3.55	3.56	-
Fuc**1**	4.72	3.65	3.71	3.61	3.93	1.05	-

Signal	Proton	Carbon
**Aromatic**	7.95-7.82 (m, 4H)<br>7.54-7.47 (m, 3H)	128.4-125.7
**CH_2**Ph	5.33 (d, *J* = 12.7 Hz)<br>5.14 (d, *J* = 12.6 Hz)	66.9
NH-**CH**-COOH	4.29 (dd, *J* = 9.5, 3.6 Hz)	53.1
C(O)-**CH_2**-CH	2.73 (dd, *J* = 14.8, 2.9 Hz)<br>2.51 (dd, *J* = 15.0, 9.9 Hz)	38.7

^{13}C NMR (150 MHz, D_2O): δ (ppm)

	C1
GlcNAc**1**	78.2
GlcNAc**2**	100.8
GlcNAc**3**	99.9
GlcNAc**4**	100.6
GlcNAc**5**	99.5
Man**1**	100.1
Man**2**	99.9
Man**3**	97.7
Fuc**1**	99.2

[a] Not reported

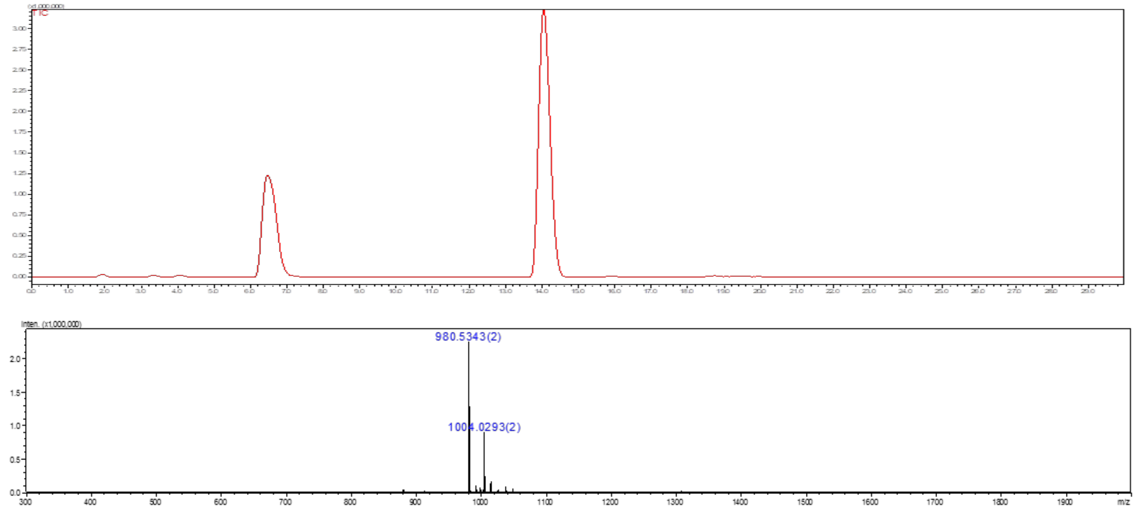

Figure S10. Liquid chromatography (LC) trace and mass (ESI-MS) spectrum (peak at retention time = 14.1 min) of **16**.

Compound R

The amine of *N*-glycan compound **16** was subjected for the hydrogenation using a general protocol **3k** to obtain **R** as a white solid.

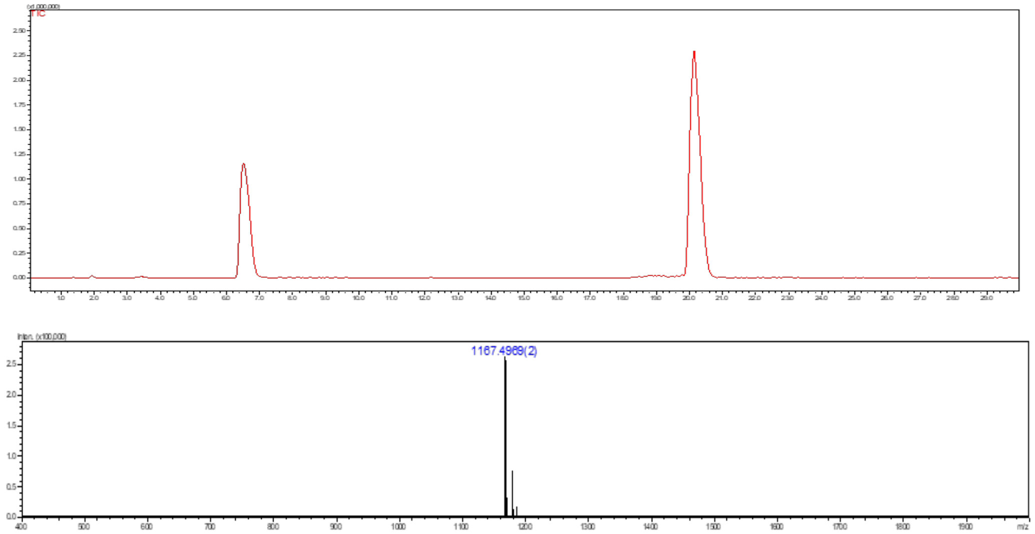

Figure S11. Liquid chromatography (LC) trace and mass (ESI-MS) spectrum (peak at retention time = 20.2 min) of **R**.

Compound 6

Compound **6** was synthesized from **3** (18 mg, 1.65 µmol) following the general protocol **3c** for the installation of GlcNAc moiety using UDP-GlcNAc. The product **6** was obtained as a white fluffy solid (19.9 mg, 79%).

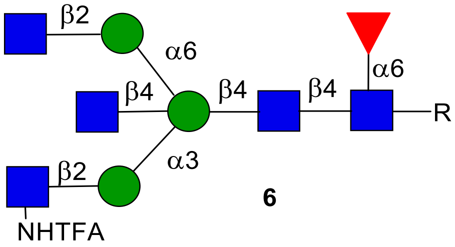

6

^{1}H NMR (600 MHz, D_2O): δ (ppm)

	H1	H2	H3	H4	H5	H6	NHAc
GlcNAc**1**	4.85	3.70	3.44	3.39	3.61	N/R[a]	2.02-1.79 (12H)

GlcNAc**2**	4.54	3.71	3.49	3.64	N/R	N/R	2.02-1.79 (12H)
GlcNTFA	4.46	3.63	3.43	N/R	N/R	N/R	-
GlcNAc**3**	4.39	3.62	3.34	3.19	3.49	N/R	2.02-1.79 (12H)
GlcNAc**4**	4.61	3.74	3.41	3.32	N/R	N/R	2.02-1.79 (12H)
Man**1**	4.61	4.09	3.76	3.98	3.47	3.82	-
Man**2**	4.93	1.19	3.83	3.36	3.47	N/R	-
Man**3**	4.92	4.07	3.76	3.42	3.55	N/R	-
Fuc**1**	4.73	3.66	3.72	3.62	3.95	1.07	-

**Signal**	**Proton**	**Carbon**
**Aromatic**	7.91-7.84 (m, 4H)<br>7.53-7.48 (m, 3H)	128.3-125.7
**CH_2**Ph	5.33 (d, J = 12.8 Hz)<br>5.14 (d, J = 12.7 Hz)	66.9
NH-**CH**-COOH	4.29 (dd, J = 9.5, 3.6 Hz)	53.1
C(O)-**CH_2**-CH	2.75 (dd, J = 14.8, 2.9 Hz)<br>2.51 (dd, J = 15.0, 9.9 Hz)	38.6

^{13}C NMR (150 MHz, D_2O): δ (ppm)

	**C1**
GlcNAc**1**	78.2
GlcNAc**2**	100.8
GlcNTFA	98.9
GlcNAc**3**	100.5
GlcNAc**4**	99.4
Man**1**	100.1
Man**2**	99.8
Man**3**	97.6
Fuc**1**	99.1

[a] Not reported

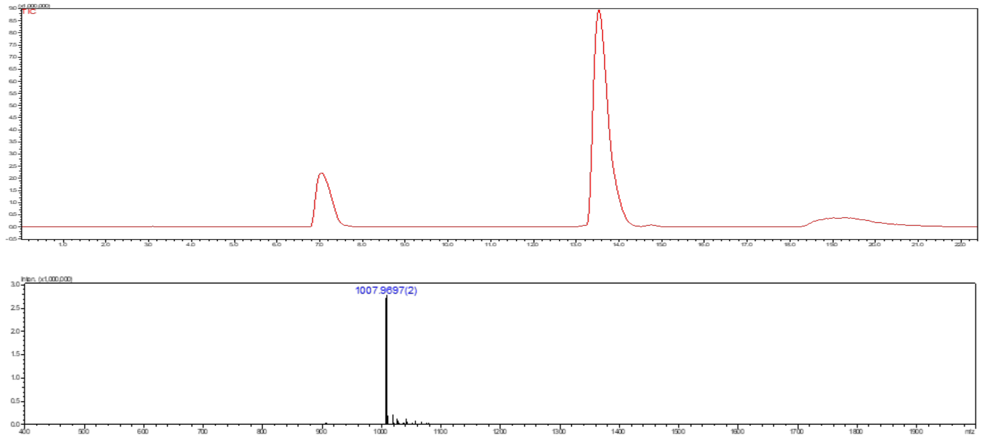

Figure S12. Liquid chromatography (LC) trace and mass (ESI-MS) spectrum (peak at retention time = 13.7 min) of **6**.

Compound 4

4 was synthesized from **2** (3 mg, 1.65 µmol) following the general protocol **3c** for the installation of GlcNAc moiety using UDP-GlcNAc. The product **4** was obtained as a white fluffy solid (2.3 mg, 76%).

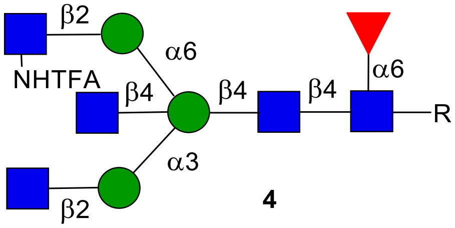

4

^{1}H NMR (600 MHz, D_2O): δ (ppm)

	H1	H2	H3	H4	H5	H6	NHAc
GlcNAc**1**	4.85	3.69	3.44	3.39	3.61	N/R[a]	2.01-1.79 (12H)
GlcNAc**2**	4.54	3.71	3.49	3.66	N/R	N/R	2.01-1.79 (12H)
GlcNAc**3**	4.47	3.63	3.52	3.38	N/R	N/R	2.01-1.79 (12H)
GlcNAc**4**	4.37	3.62	3.35	3.18	3.49	N/R	2.01-1.79 (12H)
GlcNTFA	4.60	3.73	3.54	3.44	3.38	N/R	-

Man**1**	4.60	4.09	3.78	3.97	3.46	3.79	-
Man**2**	4.98	4.17	3.82	3.41	3.62	N/R	-
Man**3**	4.89	4.11	3.77	3.35	3.53	N/R	-
Fuc**1**	4.72	3.66	3.72	3.60	3.94	1.05	-

**Signal**	**Proton**	**Carbon**
**Aromatic**	7.91-7.84 (m, 4H)<br>7.53-7.47 (m, 3H)	128.4-125.7
**CH_2**Ph	5.33 (d, J = 12.8 Hz)<br>5.14 (d, J = 12.7 Hz)	66.9
NH-**CH**-COOH	4.29 (dd, J = 9.5, 3.6 Hz)	53.1
C(O)-**CH_2**-CH	2.75 (dd, J = 14.8, 2.9 Hz)<br>2.51 (dd, J = 15.0, 9.9 Hz)	38.6

^{13}C NMR (150 MHz, D_2O): δ (ppm)

	**C1**
GlcNAc**1**	78.2
GlcNAc**2**	100.9
GlcNAc**3**	99.9
GlcNAc**4**	100.6
GlcNTFA	98.7
Man**1**	100.2
Man**2**	99.9
Man**3**	97.6
Fuc**1**	99.2

[a] Not reported

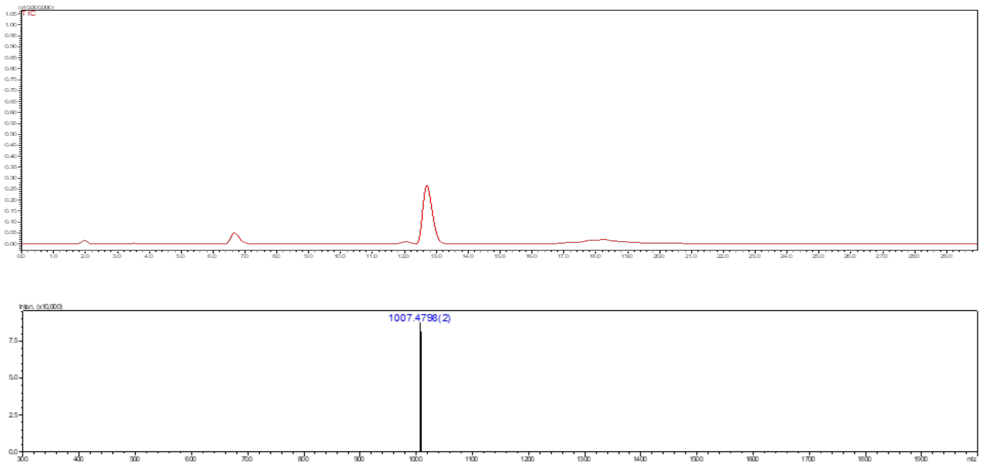

Figure S13. Liquid chromatography (LC) trace and mass (ESI-MS) spectrum (peak at retention time = 12.8 min) of **4**.

Compound 17

17 was prepared starting from **3** (2.9 mg, 1.65 μmol) by first converting GlcNHTFA to $GlcNH_2$ following general procedure **3i** to get amine intermediate. To the resulting intermediate was subjected for the conversion of $GlcNH_2$ to $GlcN_3$ using the general procedure **3l** to obtain **17** as a white solid (2.4 mg, 87% over 2 steps).

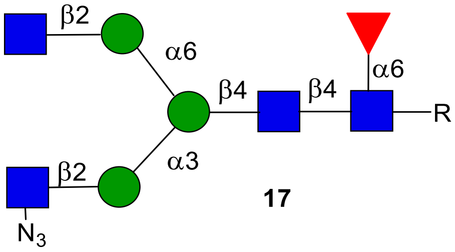

17

^{1}H NMR (600 MHz, D_2O): δ (ppm)

	H1	H2	H3	H4	H5	H6	NHAc
GlcNAc**1**	4.86	3.70	3.44	3.38	3.61	N/R[a]	2.02-1.79 (9H)
GlcNAc**2**	4.55	3.71	3.51	N/R	N/R	N/R	2.02-1.79 (9H)
$GlcN_3$	4.55	3.31	3.35	N/R	N/R	N/R	-
GlcNAc**3**	4.46	3.63	3.48	3.36	N/R	N/R	2.02-1.79 (9H)

Man**1**	4.71	4.17	3.70	3.57	N/R	N/R	-
Man**2**	5.05	4.11	3.82	3.36	3.54	N/R	-
Man**3**	5.04	4.14	3.89	3.57	N/R	N/R	-
Fuc**1**	4.72	3.66	3.71	3.60	3.93	1.04	-

**Signal**	**Proton**	**Carbon**
**Aromatic**	7.91-7.84 (m, 4H)<br>7.53-7.48 (m, 3H)	128.3-125.7
$\mathbf{CH_2}$Ph	5.33 (d, J = 12.8 Hz)<br>5.14 (d, J = 12.7 Hz)	66.8
NH-**CH**-COOH	4.29 (dd, J = 9.5, 3.6 Hz)	53.1
C(O)-$\mathbf{CH_2}$-CH	2.75 (dd, J = 14.8, 2.9 Hz)<br>2.51 (dd, J = 15.0, 9.9 Hz)	38.6

^{13}C NMR (150 MHz, D_2O): δ (ppm)

	**C1**
GlcNAc**1**	78.0
GlcNAc**2**	100.8
$GlcN_3$	99.6
GlcNAc**3**	99.7
Man**1**	100.5
Man**2**	99.2
Man**3**	96.6
Fuc**1**	99.1

[a] Not reported

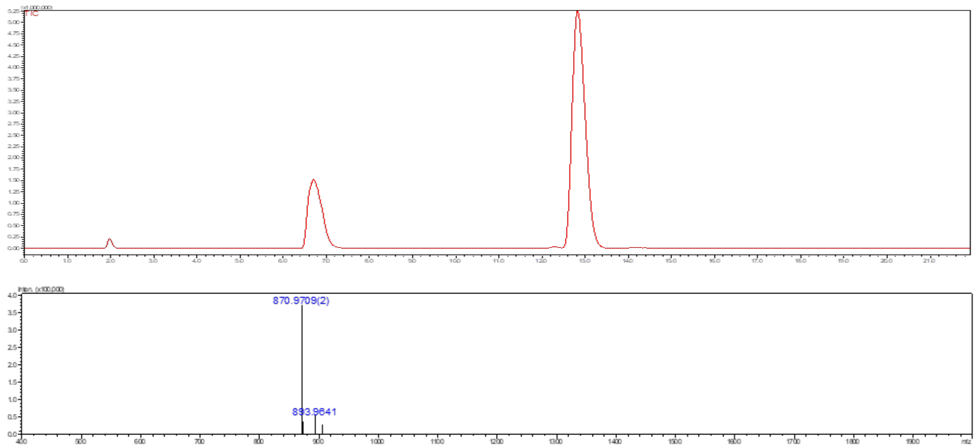

Figure S14. Liquid chromatography (LC) trace and mass (ESI-MS) spectrum (peak at retention time = 12.9 min) of **17**.

Compound 18

18 was prepared starting from **2** (2.6 mg, 1.49 μmol) by first converting GlcNHTFA to $GlcNH_2$ following general procedure **3i** to get amine intermediate. To the resulting intermediate was subjected for the conversion of $GlcNH_2$ to $GlcN_3$ using the general procedure **3l** to obtain **18** as a white solid (2.1 mg, 84% over 2 steps).

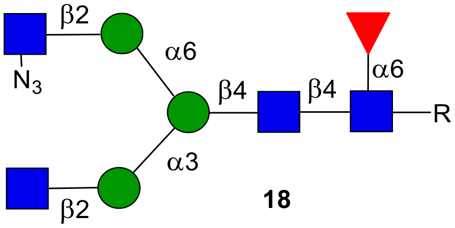

18

^{1}H NMR (600 MHz, D_2O): δ (ppm)

	H1	H2	H3	H4	H5	H6	NHAc
GlcNAc**1**	4.87	3.75	3.45	3.38	3.61	N/R[a]	2.01-1.79 (9H)
GlcNAc**2**	4.56	3.71	3.50	N/R	N/R	N/R	2.01-1.79 (9H)
GlcNAc**3**	4.48	3.63	3.48	3.36	N/R	N/R	2.01-1.79 (9H)
$GlcN_3$	4.56	3.31	3.33	N/R	N/R	N/R	-

Man**1**	4.71	4.20	3.72	3.56	N/R	N/R	-
Man**2**	5.22	4.23	3.89	3.57	N/R	N/R	-
Man**3**	4.84	4.04	3.82	3.44	3.54	N/R	-
Fuc**1**	4.73	3.67	3.71	3.60	3.94	1.05	-

**Signal**	**Proton**	**Carbon**
**Aromatic**	7.91-7.84 (m, 4H)<br>7.53-7.47 (m, 3H)	128.4-125.7
**CH_2**Ph	5.33 (d, *J* = 12.8 Hz)<br>5.14 (d, *J* = 12.7 Hz)	66.9
NH-**CH**-COOH	4.29 (dd, *J* = 9.5, 3.6 Hz)	53.1
C(O)-**CH_2**-CH	2.75 (dd, *J* = 14.8, 2.9 Hz)<br>2.51 (dd, *J* = 15.0, 9.9 Hz)	38.6

^{13}C NMR (150 MHz, D_2O): δ (ppm)

	**C1**
GlcNAc**1**	78.2
GlcNAc**2**	100.9
GlcNAc**3**	100.1
$GlcN_3$	99.6
Man**1**	100.4
Man**2**	99.6
Man**3**	97.1
Fuc**1**	99.2

[a] Not reported

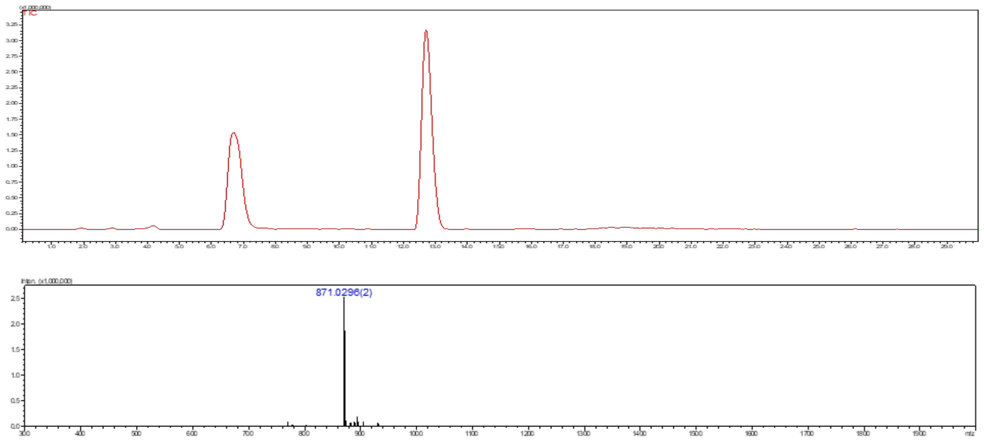

Figure S15. Liquid chromatography (LC) trace and mass (ESI-MS) spectrum (peak at retention time = 12.8 min) of **18**.

Compound 21

21 was prepared starting from **2** (3 mg, 1.48 µmol) by first installing a β1,4 GlcNAc using the general procedure **3d** for GnT-IVB. The reaction was monitored by ESI-MS and after complete conversion to MGAT4 arm installed intermediate [confirmed by LC-ESI-MS, peak at retention time = 12.5 min, m/z = 1007.5259(1)]. To the resulting compound was subjected for the installation of a β1,4 GlcNAc using the general procedure **3c** to obtain **21** as a white solid (2.2 mg, 66% over 2 steps).

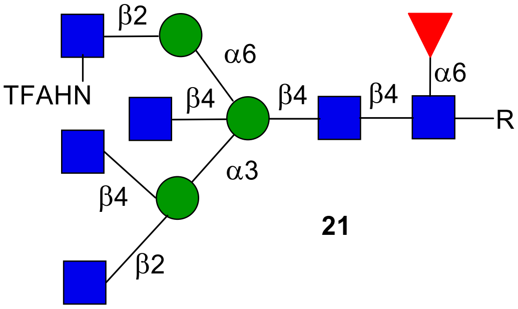

21

^{1}H NMR (600 MHz, D_2O): δ (ppm)

	H1	H2	H3	H4	H5	H6	NHAc
GlcNAc**1**	4.86	3.69	3.44	3.39	N/R[a]	N/R	2.01-1.79 (15H)
GlcNAc**2**	4.54	3.71	3.48	3.63	N/R	N/R	2.01-1.79 (15H)
GlcNAc**3**	4.46	3.65	3.51	3.38	N/R	N/R	2.01-1.79 (15H)

GlcNAc**4**	4.43	3.68	3.49	3.43	N/R	N/R	2.01-1.79 (15H)
GlcNAc**5**	4.37	3.62	3.33	3.18	3.49	N/R	2.01-1.79 (15H)
GlcNTFA	4.60	3.74	3.54	3.44	N/R	N/R	-
Man**1**	4.59	4.07	3.77	3.97	3.52	N/R	-
Man**2**	4.97	4.21	3.97	3.52	3.65	3.46	-
Man**3**	4.89	4.10	3.77	3.35	3.53	N/R	-
Fuc**1**	4.73	3.66	3.73	3.61	3.94	1.06	-

**Signal**	**Proton**	**Carbon**
**Aromatic**	7.91-7.85 (m, 4H)<br>7.53-7.48 (m, 3H)	128.4-125.9
**CH$_2$**Ph	5.33 (d, *J* = 12.8 Hz)<br>5.14 (d, *J* = 12.7 Hz)	66.8
NH-**CH**-COOH	4.29 (dd, *J* = 9.5, 3.6 Hz)	53.2
C(O)-**CH$_2$**-CH	2.75 (dd, *J* = 14.8, 2.9 Hz)<br>2.51 (dd, *J* = 15.0, 9.9 Hz)	38.9

^{13}C NMR (150 MHz, D_2O): δ (ppm)

	**C1**
GlcNAc**1**	78.2
GlcNAc**2**	100.9
GlcNAc**3**	99.9
GlcNAc**4**	101.8
GlcNAc**4**	100.6
GlcNTFA	98.8
Man**1**	100.1
Man**2**	99.6
Man**3**	97.5
Fuc**1**	99.2

[a] Not reported

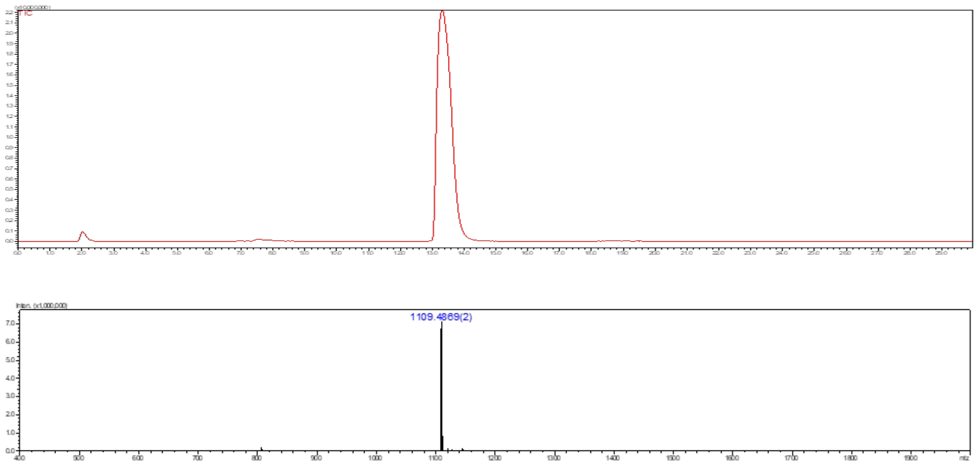

Figure S16. Liquid chromatography (LC) trace and mass (ESI-MS) spectrum (peak at retention time = 13.5 min) of **21**.

Compound 22

22 was prepared starting from **3** (14.2 mg, 7.82 μmol) by first installing a β1,4 GlcNAc using the general procedure **3e** for GnT-V. The reaction was monitored by ESI-MS and after complete conversion to MGAT5 installed intermediate [confirmed by LC-ESI-MS, peak at retention time = 12.8 min, m/z = 1007.5259(1)], then fraction containing compound was lyophilized. To the resulting compound was subjected for the installation of a β1,4 GlcNAc using the general procedure **3c** to obtain **22** as a white solid (10.2 mg, 59% over 2 steps).

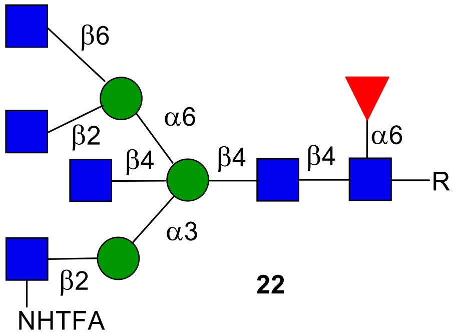

22

^{1}H NMR (600 MHz, D_2O): δ (ppm)

	H1	H2	H3	H4	H5	H6	NHAc
GlcNAc**1**	4.86	3.69	3.45	3.39	N/R[a]	N/R	2.02-1.79 (15H)
GlcNAc**2**	4.53	3.73	3.48	3.65	N/R	N/R	2.02-1.79 (15H)

GlcNTFA	4.66	3.75	3.47	N/R	N/R	N/R	-
GlcNAc**3**	4.42	3.63	3.32	3.21	3.54	N/R	2.02-1.79 (15H)
GlcNAc**4**	4.49	3.60	3.39	3.32	N/R	N/R	2.02-1.79 (15H)
GlcNAc**5**	4.45	3.67	3.51	3.41	N/R	N/R	2.02-1.79 (15H)
Man**1**	4.62	4.10	3.77	4.05	3.45	N/R	-
Man**2**	4.93	4.18	3.84	3.35	N/R	N/R	-
Man**3**	4.83	4.05	3.73	3.35	N/R	N/R	-
Fuc**1**	4.73	3.67	3.72	3.61	3.94	1.06	-

Signal	Proton	Carbon
**Aromatic**	7.91-7.84 (m, 4H)<br>7.53-7.47 (m, 3H)	128.4-125.7
**CH_2**Ph	5.33 (d, *J* = 12.8 Hz)<br>5.14 (d, *J* = 12.7 Hz)	66.8
NH-**CH**-COOH	4.29 (dd, *J* = 9.5, 3.6 Hz)	53.0
C(O)-**CH_2**-CH	2.75 (dd, *J* = 14.8, 2.9 Hz)<br>2.51 (dd, *J* = 15.0, 9.9 Hz)	38.6

^{13}C NMR (150 MHz, D_2O): δ (ppm)

	**C1**
GlcNAc**1**	78.2
GlcNAc**2**	101.0
GlcNTFA	98.9
GlcNAc**3**	99.7
GlcNAc**4**	99.7
GlcNAc**5**	101.7
Man**1**	99.9
Man**2**	99.9
Man**3**	98.0
Fuc**1**	99.2

[a] Not reported

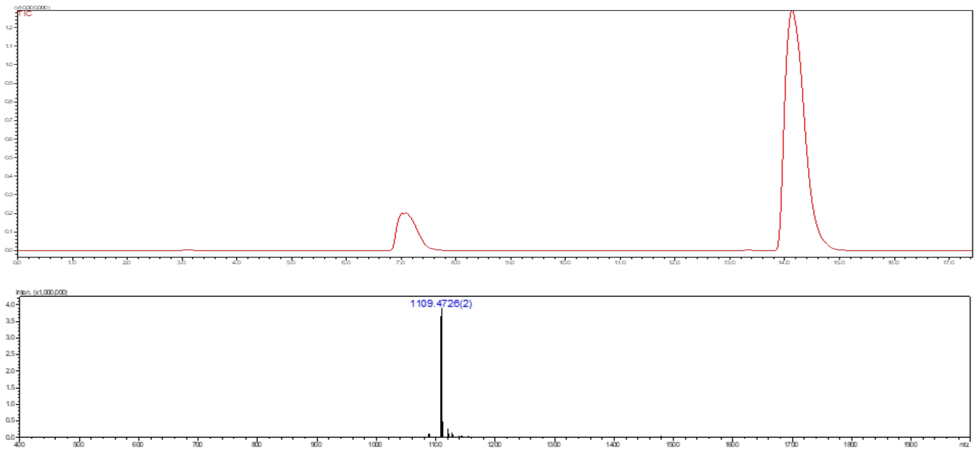

Figure S17. Liquid chromatography (LC) trace and mass (ESI-MS) spectrum (peak at retention time = 14.3 min) of **22**.

Compound 25

N-Glycan **6** (10.1 mg, 5.10 µmol) was dissolved in deionized water with the final reaction concentration of 5 mM. The pH of the reaction solution was adjusted to 10 using NaOH (1 M), and the mixture solution was incubated for 3-5 h at 37 °C with shaking. After complete conversion of starting material to amine intermediate **23** [confirmed by LC-ESI-MS, peak at retention time = 15.8 min, m/z = 959.5598(1)]. Then, reaction was neutralized by aqueous acetic acid (1 M). The product was purified by C18 biogel [Eluent - water and water:acetonitrile (8:2)]. Next, compound **23** was subjected for the installation of a β1,4 Gal using the general procedure **3f** to obtained **24** as a white solid. The amine of *N*-glycan compound **24** was subjected for the acetylation using a general protocol **3j** to obtain **25** as a white solid (5.4 mg, 51% over 3 steps).

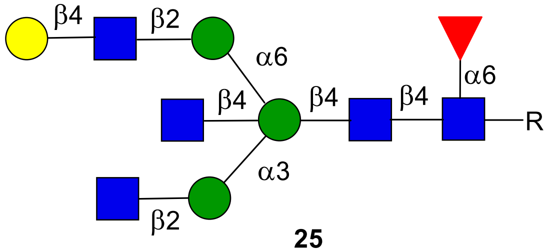

25

^{1}H NMR (900 MHz, D_2O): δ (ppm)

	**H1**	**H2**	**H3**	**H4**	**H5**	**H6**	**NHAc**
GlcNAc**1**	4.85	3.69	3.60	3.51	N/R[a]	N/R	2.02-1.79 (15H)
GlcNAc**2**	4.53	3.72	3.64	3.67	3.49	N/R	2.02-1.79 (15H)
GlcNAc**3**	4.50	3.67	3.56	N/R	N/R	N/R	2.02-1.79 (15H)
GlcNAc**4**	4.39	3.61	3.33	3.19	3.49	N/R	2.02-1.79 (15H)
GlcNAc**5**	4.48	3.64	3.53	3.41	N/R	N/R	2.02-1.79 (15H)
Man**1**	4.60	4.09	3.78	4.00	3.46	N/R	-
Man**2**	4.98	4.17	3.83	3.63	3.85	3.49	-
Man**3**	4.93	4.06	3.75	3.55	3.40	N/R	-
Fuc**1**	4.73	3.66	3.72	3.62	3.94	1.05	-
Gal**1**	4.40	3.47	3.66	3.85	N/R	N/R	-

**Signal**	**Proton**	**Carbon**
**Aromatic**	7.92-7.85 (m, 4H)<br>7.53-7.48 (m, 3H)	128.3-125.7
**CH_2**Ph	5.32 (d, *J* = 12.8 Hz)<br>5.14 (d, *J* = 12.7 Hz)	66.9
NH-**CH**-COOH	4.29 (dd, *J* = 9.5, 3.6 Hz)	53.1
C(O)-**CH_2**-CH	2.75 (dd, *J* = 14.8, 2.9 Hz)<br>2.51 (dd, *J* = 15.0, 9.9 Hz)	38.7

^{13}C NMR (225 MHz, D_2O): δ (ppm)

	**C1**
GlcNAc**1**	78.1
GlcNAc**2**	100.9
GlcNAc**3**	99.4
GlcNAc**4**	100.6
GlcNAc**5**	99.8

Man**1**	100.0
Man**2**	99.9
Man**3**	97.7
Fuc**1**	99.2
Gal**1**	102.9

[a] Not reported

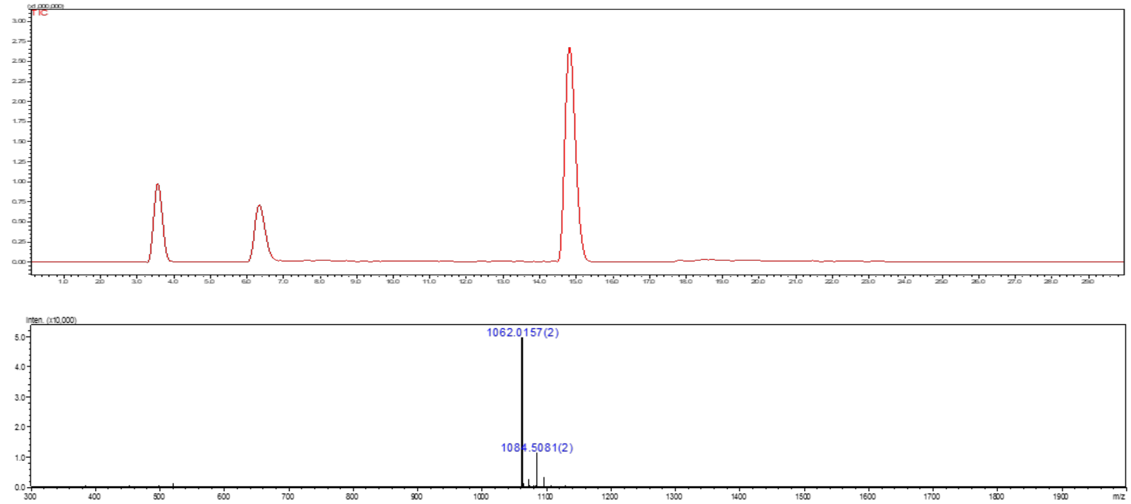

Figure S18. Liquid chromatography (LC) trace and mass (ESI-MS) spectrum (peak at retention time = 14.9 min) of **25**.

Compound A

The amine of *N*-glycan compound **25** was subjected for the hydrogenation using a general protocol **3k** to obtain **A** as a white solid.

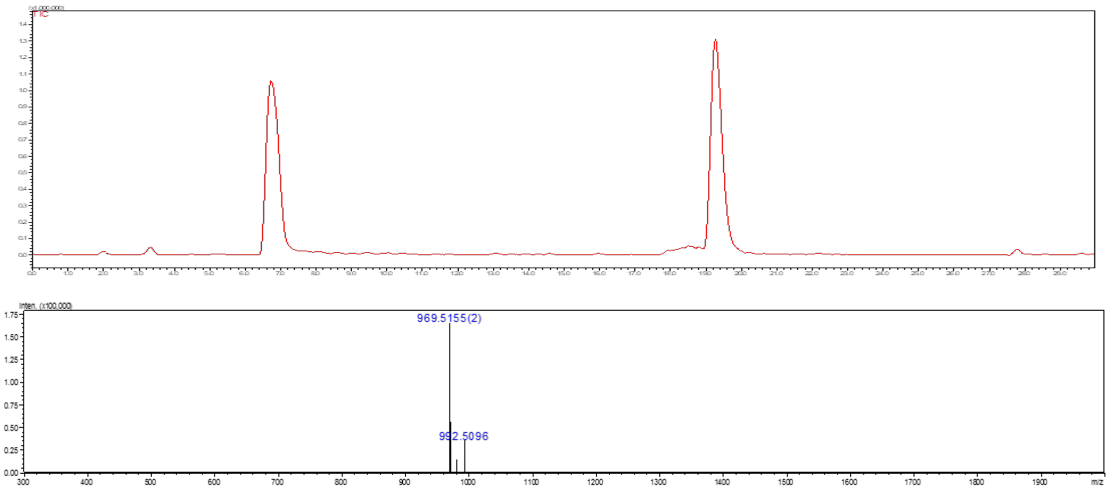

Figure S19. Liquid chromatography (LC) trace and mass (ESI-MS) spectrum (peak at retention time = 19.4 min) of **A**.

Compound 26

26 was synthesized from **25** (12 mg, 5.64 μmol) following the general protocol **3g** for the installation of Neu5Ac moiety using ST3Gal4. The product **26** was obtained as a white fluffy solid (10.2 mg, 75%).

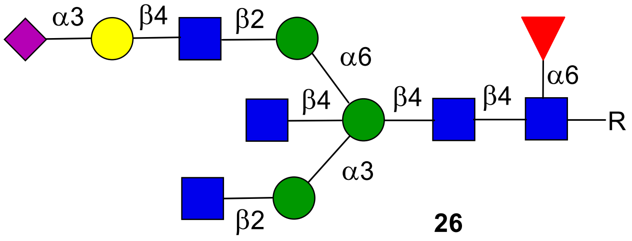

26

^{1}H NMR (600 MHz, D_2O): δ (ppm)

	**H1**	**H2**	**H3**	**H4**	**H5**	**H6**	**NHAc**
GlcNAc**1**	4.85	3.68	3.59	3.45	N/R[a]	N/R	2.02-1.79 (15H)
GlcNAc**2**	4.53	3.71	3.63	3.66	3.48	N/R	2.02-1.79 (15H)
GlcNAc**3**	4.49	3.67	3.56	N/R	N/R	N/R	2.02-1.79 (15H)
GlcNAc**4**	4.38	3.62	3.32	3.20	3.49	N/R	2.02-1.79 (15H)
GlcNAc**5**	4.47	3.65	3.51	3.40	N/R	N/R	2.02-1.79 (15H)
Man**1**	4.60	4.09	3.80	4.00	3.45	N/R	**-**
Man**2**	4.98	4.16	3.83	3.62	3.84	N/R	**-**
Man**3**	4.93	4.07	3.76	3.54	3.40	N/R	**-**
Fuc**1**	4.73	3.67	3.71	3.61	3.94	1.07	**-**
Gal**1**	4.47	3.51	4.04	3.88	N/R	N/R	**-**

	**H1**	**H2**	**H3**	**H4**	**H5**	**H6**	**H7**	**H8**	**H9**
Neu5Ac9	-	-	2.68<br>1.71	3.61	3.77	N/R	N/R	N/R	N/R

**Signal**	**Proton**	**Carbon**
**Aromatic**	7.91-7.85 (m, 4H)<br>7.53-7.48 (m, 3H)	128.3-125.7
**CH_2**Ph	5.33 (d, *J* = 12.8 Hz)<br>5.13 (d, *J* = 12.7 Hz)	66.9
NH-**CH**-COOH	4.29 (dd, *J* = 9.5, 3.6 Hz)	53.0
C(O)-**CH_2**-CH	2.75 (dd, *J* = 14.8, 2.9 Hz)<br>2.51 (dd, *J* = 15.0, 9.9 Hz)	38.7

^{13}C NMR (150 MHz, D_2O): δ (ppm)

	**C1**
GlcNAc**1**	78.2

GlcNAc**2**	100.9
GlcNAc**3**	99.4
GlcNAc**4**	100.6
GlcNAc**5**	99.8
Man**1**	100.0
Man**2**	99.9
Man**3**	97.8
Fuc**1**	99.1
Gal**1**	102.7

[a] Not reported

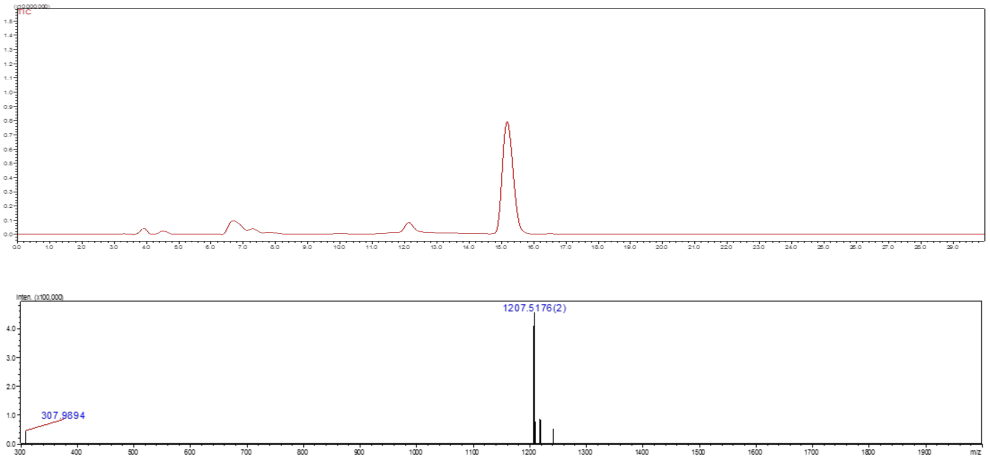

Figure S20. Liquid chromatography (LC) trace and mass (ESI-MS) spectrum (peak at retention time = 15.2 min) of **26**.

Compound B

The amine of *N*-glycan compound **26** was subjected for the hydrogenation using a general protocol **3k** to obtain **B** as a white solid.

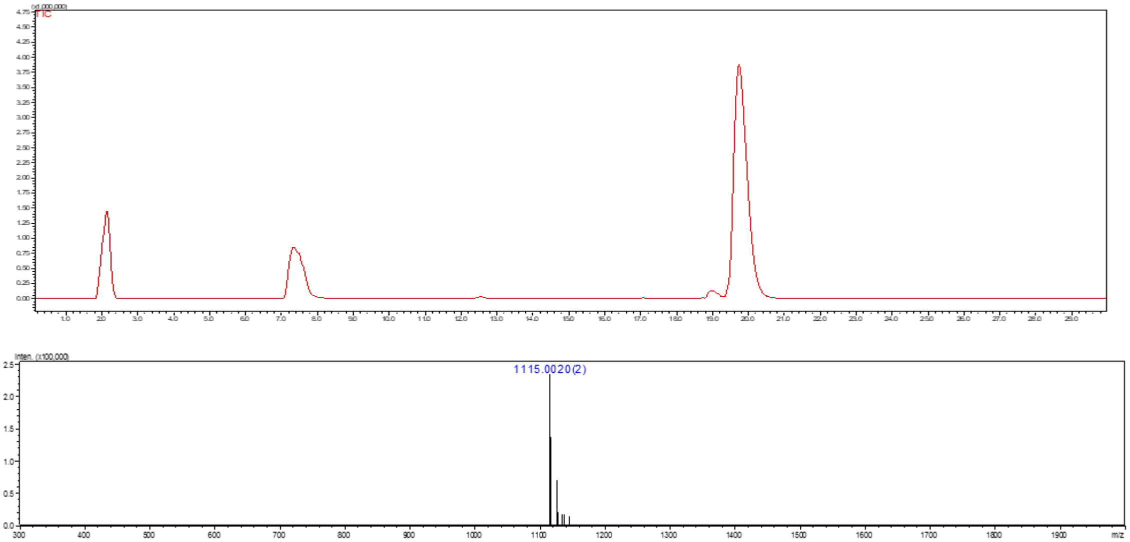

Figure S21. Liquid chromatography (LC) trace and mass (ESI-MS) spectrum (peak at retention time = 19.2 min) of **B**.

Compound 27

27 was synthesized from **25** (3 mg, 1.40 µmol) following the general protocol **3g** for the installation of Neu5Ac moiety using ST6Gal1. The product **27** was obtained as a white fluffy solid (2.9 mg, 86%).

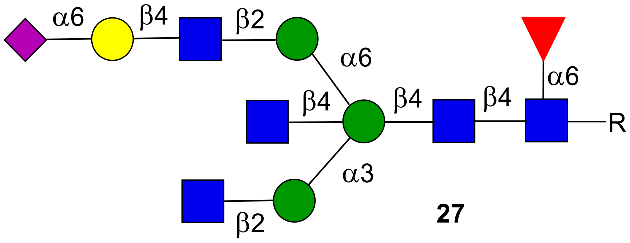

27

^{1}H NMR (900 MHz, D_2O): δ (ppm)

	**H1**	**H2**	**H3**	**H4**	**H5**	**H6**	**NHAc**
GlcNAc**1**	4.87	3.70	3.59	3.44	3.39	N/R[a]	2.02-1.79 (15H)
GlcNAc**2**	4.54	3.70	3.64	3.65	3.49	N/R	2.02-1.79 (15H)
GlcNAc**3**	4.53	3.67	3.60	N/R	N/R	N/R	2.02-1.79 (15H)
GlcNAc**4**	4.40	3.63	3.35	3.19	3.50	N/R	2.02-1.79 (15H)
GlcNAc**5**	4.49	3.65	3.53	3.40	N/R	N/R	2.02-1.79 (15H)
Man**1**	4.61	4.11	3.81	4.09	3.49	N/R	-
Man**2**	4.99	4.18	3.83	3.63	3.41	N/R	-
Man**3**	4.94	4.08	3.78	3.54	3.43	N/R	-
Fuc**1**	4.75	3.67	3.71	3.61	3.95	1.07	-
Gal**1**	4.38	3.47	3.59	3.87	N/R	N/R	-

	**H1**	**H2**	**H3**	**H4**	**H5**	**H6**	**H7**	**H8**	**H9**
Neu5Ac9	-	-	2.60<br>1.65	3.58	3.73	N/R	N/R	N/R	N/R

**Signal**	**Proton**	**Carbon**
**Aromatic**	7.92-7.85 (m, 4H)<br>7.53-7.48 (m, 3H)	128.4-125.7
**CH_2**Ph	5.33 (d, *J* = 12.8 Hz)<br>5.14 (d, *J* = 12.7 Hz)	66.8
NH-**CH**-COOH	4.29 (dd, *J* = 9.5, 3.6 Hz)	53.0
C(O)-**CH_2**-CH	2.75 (dd, *J* = 14.8, 2.9 Hz)<br>2.51 (dd, *J* = 15.0, 9.9 Hz)	38.7

^{13}C NMR (225 MHz, D_2O): δ (ppm)

	**C1**
GlcNAc**1**	78.2
GlcNAc**2**	100.9
GlcNAc**3**	99.2
GlcNAc**4**	100.6
GlcNAc**5**	100.1
Man**1**	100.2
Man**2**	100.0
Man**3**	97.5
Fuc**1**	99.3
Gal**1**	103.6

[a] Not reported

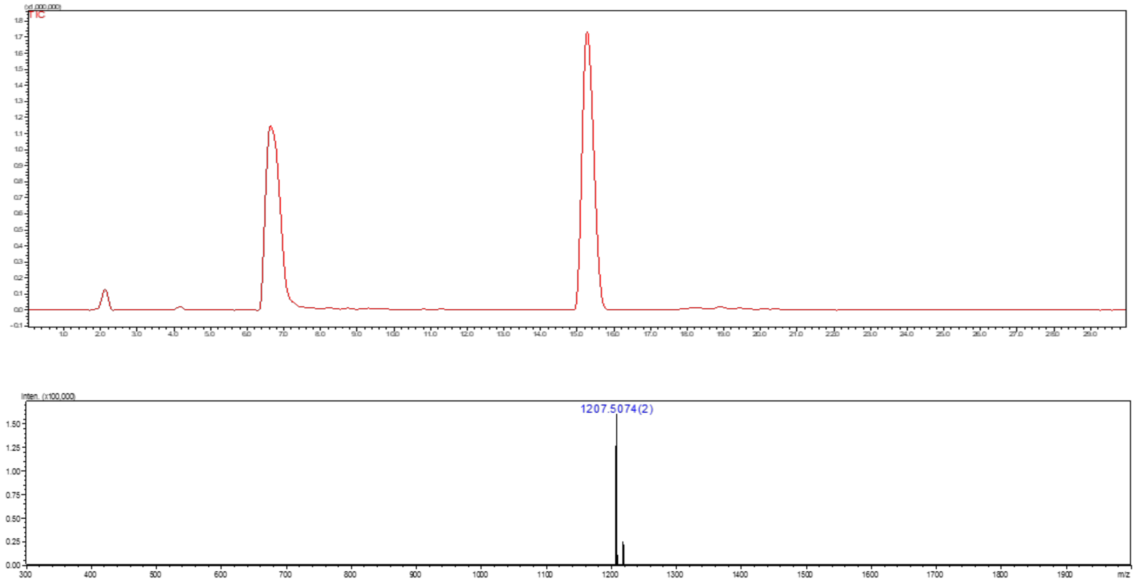

Figure S22. Liquid chromatography (LC) trace and mass (ESI-MS) spectrum (peak at retention time = 15.4 min) of **27**.

Compound C

The amine of *N*-glycan compound **27** was subjected for the hydrogenation using a general protocol **3k** to obtain **C** as a white solid.

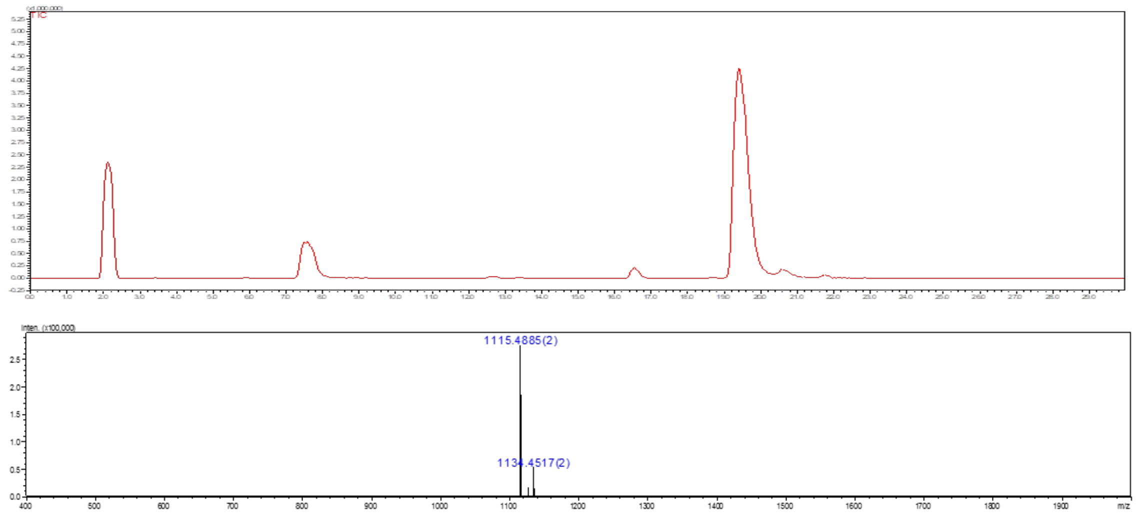

Figure S23. Liquid chromatography (LC) trace and mass (ESI-MS) spectrum (peak at retention time = 19.5 min) of **C**.

Compound 28

28 was synthesized from **26** (1 mg, 0.41 μmol) following the general protocol **3h** for the installation of Fuc moiety using FUT6. The product **28** was obtained as a white fluffy solid (0.85 mg, 80%).

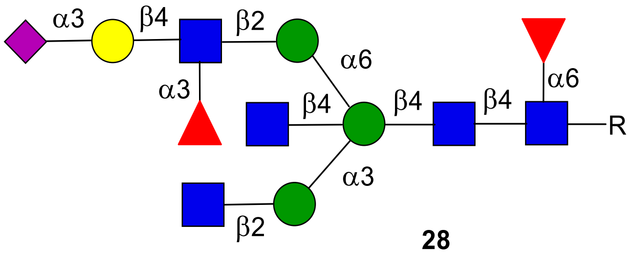

28

^{1}H NMR (600 MHz, D_2O): δ (ppm)

	**H1**	**H2**	**H3**	**H4**	**H5**	**H6**	**NHAc**
GlcNAc**1**	4.88	3.70	3.62	3.44	N/R[a]	N/R	2.02-1.79 (15H)
GlcNAc**2**	4.54	3.73	3.65	3.15	N/R	N/R	2.02-1.79 (15H)
GlcNAc**3**	4.50	3.85	3.50	N/R	N/R	N/R	2.02-1.79 (15H)
GlcNAc**4**	4.39	3.62	3.35	3.19	3.49	N/R	2.02-1.79 (15H)
GlcNAc**5**	4.48	3.65	3.52	3.40	N/R	N/R	2.02-1.79 (15H)
Man**1**	4.62	4.11	3.79	3.98	3.50	N/R	**-**
Man**2**	4.98	4.17	3.83	3.64	3.40	N/R	**-**
Man**3**	4.94	4.06	3.78	3.54	3.40	N/R	**-**
Fuc**1**	4.74	3.65	3.72	3.61	3.95	1.07	**-**
Gal**1**	4.44	3.44	4.00	3.85	N/R	N/R	**-**
Fuc**2**	5.05	3.61	3.84	3.71	4.75	1.10	**-**

	**H1**	**H2**	**H3**	**H4**	**H5**	**H6**	**H7**	**H8**	**H9**
Neu5Ac9	-	-	2.68<br>1.72	3.61	3.76	N/R	N/R	N/R	N/R

**Signal**	**Proton**	**Carbon**
**Aromatic**	7.91-7.85 (m, 4H)<br>7.53-7.47 (m, 3H)	128.4-125.7
**CH₂**Ph	5.32 (d, J = 12.8 Hz)<br>5.16 (d, J = 12.7 Hz)	66.9
NH-**CH**-COOH	4.38 (dd, J = 9.5, 3.6 Hz)	53.1
C(O)-**CH₂**-CH	2.77 (dd, J = 14.8, 2.9 Hz)<br>2.59 (dd, J = 15.0, 9.9 Hz)	38.6

^{13}C NMR (150 MHz, D_2O): δ (ppm)

	**C1**
GlcNAc**1**	78.2
GlcNAc**2**	100.9
GlcNAc**3**	98.9
GlcNAc**4**	100.4
GlcNAc**5**	99.9
Man**1**	100.2
Man**2**	99.9
Man**3**	97.3
Fuc**1**	99.2
Gal**1**	101.6
Fuc**2**	98.4

[a] Not reported

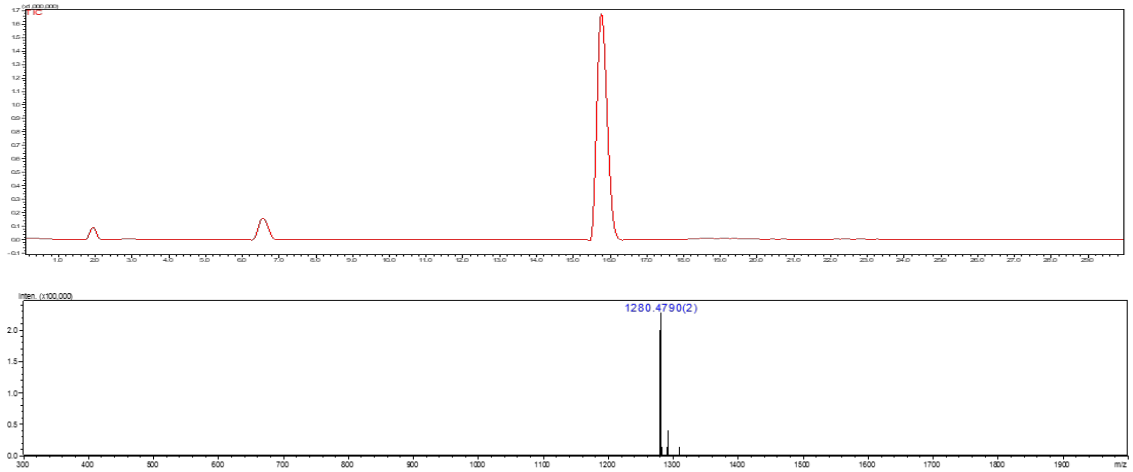

Figure S24. Liquid chromatography (LC) trace and mass (ESI-MS) spectrum (peak at retention time = 15.9 min) of **28**.

Compound E

The amine of *N*-glycan compound **28** was subjected for the hydrogenation using a general protocol **3k** to obtain **E** as a white solid.

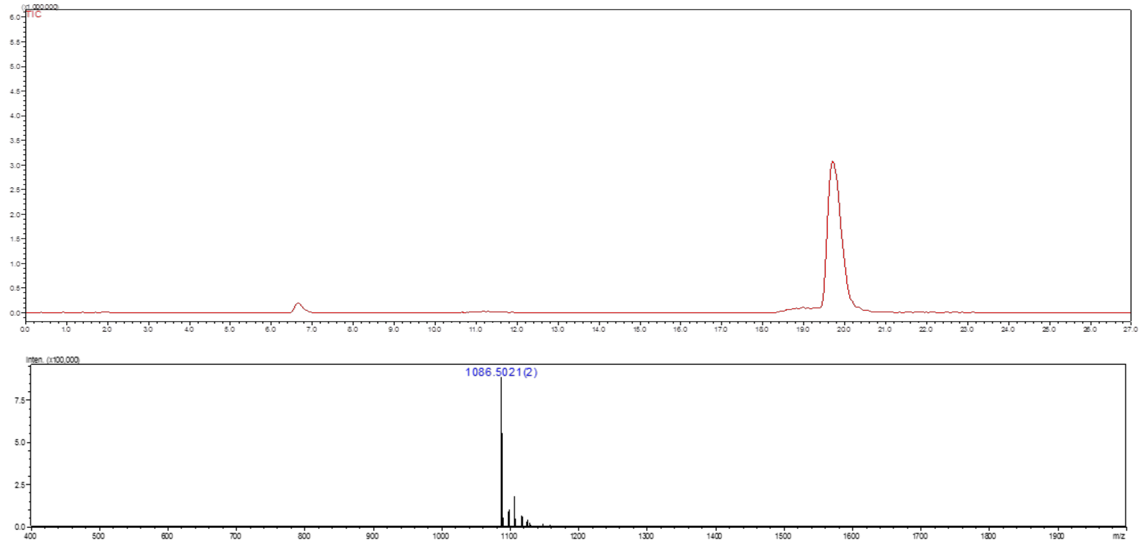

Figure S25. Liquid chromatography (LC) trace and mass (ESI-MS) spectrum (peak at retention time = 19.8 min) of **E**.

Compound 29

29 was synthesized from **26** (2 mg, 0.82 μmol) following the general protocol **3f** for the installation of Gal moiety using B4GalT1. The product **29** was obtained as a white fluffy solid (1.45 mg, 68%).

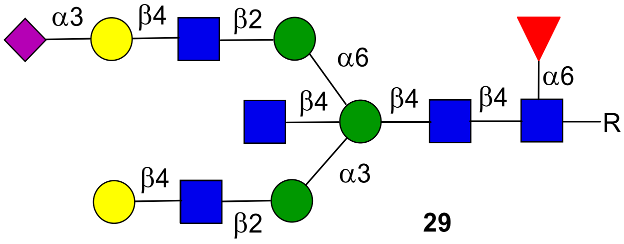

29

^{1}H NMR (600 MHz, D_2O): δ (ppm)

	**H1**	**H2**	**H3**	**H4**	**H5**	**H6**	**NHAc**
GlcNAc**1**	4.85	3.69	3.59	N/R[a]	N/R	N/R	2.03-1.79 (15H)
GlcNAc**2**	4.53	3.73	3.64	3.66	3.48	N/R	2.03-1.79 (15H)
GlcNAc**3**	4.51	3.69	3.55	N/R	N/R	N/R	2.03-1.79 (15H)
GlcNAc**4**	4.38	3.61	3.32	3.19	3.39	N/R	2.03-1.79 (15H)
GlcNAc**5**	4.49	3.67	3.55	3.46	N/R	N/R	2.03-1.79 (15H)
Man**1**	4.60	4.08	3.79	4.00	3.45	N/R	-
Man**2**	4.98	4.20	3.83	3.62	N/R	N/R	-
Man**3**	4.94	4.07	3.74	3.54	3.40	N/R	-
Fuc**1**	4.73	3.66	3.71	3.62	3.93	1.07	-
Gal**1**	4.38	3.47	N/R	3.84	N/R	N/R	-
Gal**2**	4.48	3.51	4.04	3.89	N/R	N/R	-

	**H1**	**H2**	**H3**	**H4**	**H5**	**H6**	**H7**	**H8**	**H9**
Neu5Ac9	-	-	2.68<br>1.73	3.60	3.77	N/R	N/R	N/R	N/R

**Signal**	**Proton**	**Carbon**
**Aromatic**	7.92-7.85 (m, 4H)<br>7.53-7.48 (m, 3H)	128.3-125.7
**CH_2**Ph	5.33 (d, *J* = 12.8 Hz)<br>5.14 (d, *J* = 12.7 Hz)	66.8
NH-**CH**-COOH	4.29 (dd, *J* = 9.5, 3.6 Hz)	53.0
C(O)-**CH_2**-CH	2.75 (dd, *J* = 14.8, 2.9 Hz)<br>2.51 (dd, *J* = 15.0, 9.9 Hz)	38.6

^{13}C NMR (150 MHz, D_2O): δ (ppm)

	**C1**
GlcNAc**1**	78.2
GlcNAc**2**	100.9
GlcNAc**3**	99.7
GlcNAc**4**	100.6
GlcNAc**5**	99.4
Man**1**	100.0
Man**2**	99.9
Man**3**	97.7
Fuc**1**	99.2
Gal**1**	102.9
Gal**2**	102.7

[a] Not reported

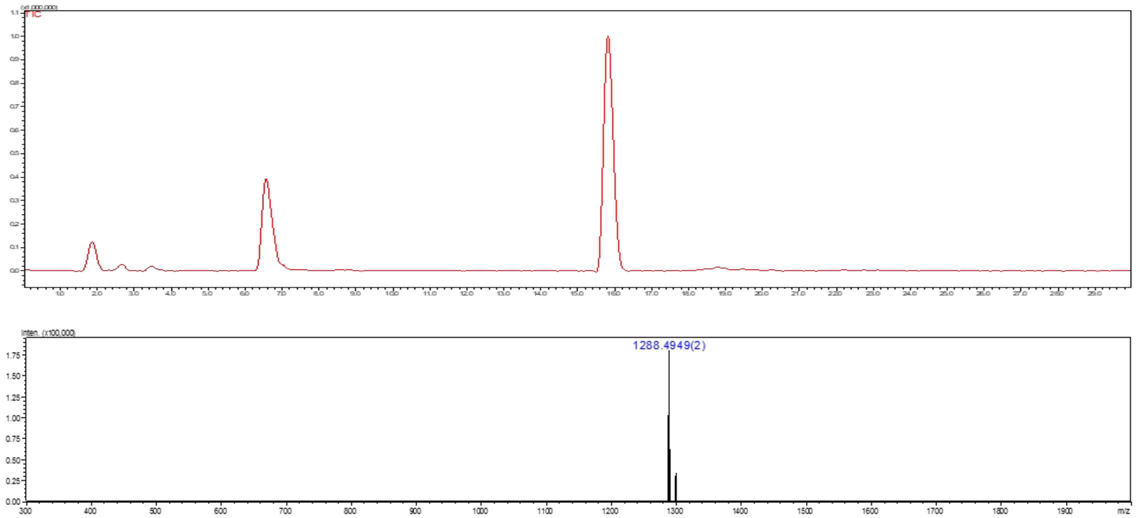

Figure S26. Liquid chromatography (LC) trace and mass (ESI-MS) spectrum (peak at retention time = 15.9 min) of **29**.

Compound D

The amine of *N*-glycan compound **29** was subjected for the hydrogenation using a general protocol **3k** to obtain **D** as a white solid.

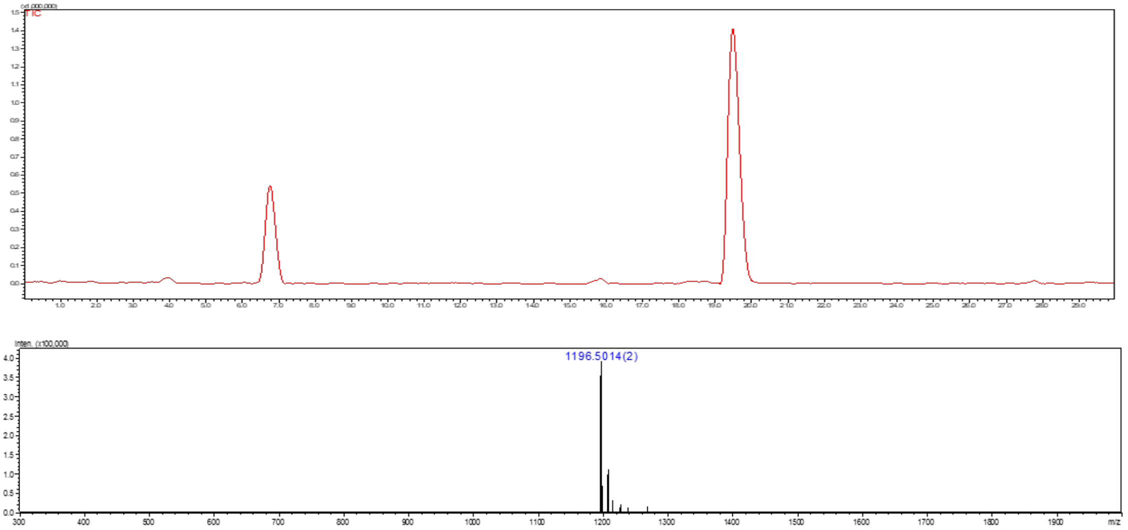

Figure S27. Liquid chromatography (LC) trace and mass (ESI-MS) spectrum (peak at retention time = 19.6 min) of **D**.

Compound 7

7 was synthesized from **28** (1.5 mg, 0.58 μmol) following the general protocol **3f** for the installation of Gal moiety using B4GalT1. The product **7** was obtained as a white fluffy solid (0.99 mg, 63%).

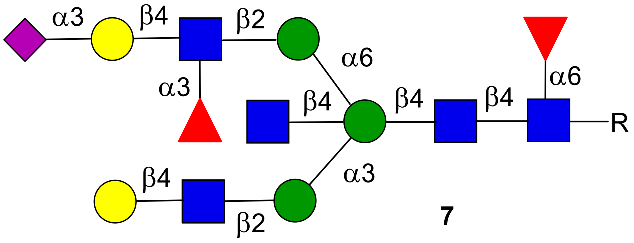

7

^{1}H NMR (900 MHz, D_2O): δ (ppm)

	H1	H2	H3	H4	H5	H6	NHAc
GlcNAc**1**	4.84	3.69	3.59	N/R[a]	N/R	N/R	2.03-1.79 (15H)
GlcNAc**2**	4.52	3.73	3.65	N/R	N/R	N/R	2.03-1.79 (15H)
GlcNAc**3**	4.50	3.69	3.50	N/R	N/R	N/R	2.03-1.79 (15H)
GlcNAc**4**	4.39	3.62	3.33	3.18	3.49	N/R	2.03-1.79 (15H)
GlcNAc**5**	4.49	3.68	N/R	N/R	N/R	N/R	2.03-1.79 (15H)
Man**1**	4.61	4.10	3.78	3.98	3.50	N/R	-
Man**2**	4.97	4.17	3.83	N/R	N/R	N/R	-
Man**3**	4.94	4.05	3.76	3.54	3.40	N/R	-
Fuc**1**	4.72	3.65	3.71	3.62	3.93	1.06	-
Fuc**2**	5.04	3.62	3.84	3.65	3.75	1.10	-
Gal**1**	4.39	3.46	3.94	N/R	N/R	N/R	-
Gal**2**	4.45	3.43	4.00	N/R	N/R	N/R	-

	H1	H2	H3	H4	H5	H6	H7	H8	H9
Neu5Ac9	-	-	2.68<br>171	3.60	3.76	N/R	N/R	N/R	N/R

Signal	Proton	Carbon
**Aromatic**	7.92-7.85 (m, 4H)<br>7.52-7.47 (m, 3H)	128.4-125.7
**CH_2**Ph	5.32 (d, *J* = 12.8 Hz)<br>5.14 (d, *J* = 12.7 Hz)	66.8
NH-**CH**-COOH	4.29 (dd, *J* = 9.5, 3.6 Hz)	53.1
C(O)-**CH_2**-CH	2.75 (dd, *J* = 14.8, 2.9 Hz)<br>2.51 (dd, *J* = 15.0, 9.9 Hz)	38.8

^{13}C NMR (225 MHz, D_2O): δ (ppm)

	**C1**
GlcNAc**1**	78.1
GlcNAc**2**	100.9
GlcNAc**3**	99.7
GlcNAc**4**	100.4
GlcNAc**5**	99.7
Man**1**	100.4
Man**2**	99.9
Man**3**	97.2
Fuc**1**	99.2
Gal**1**	102.8
Gal**2**	101.5
Fuc**2**	98.4

[a] Not reported

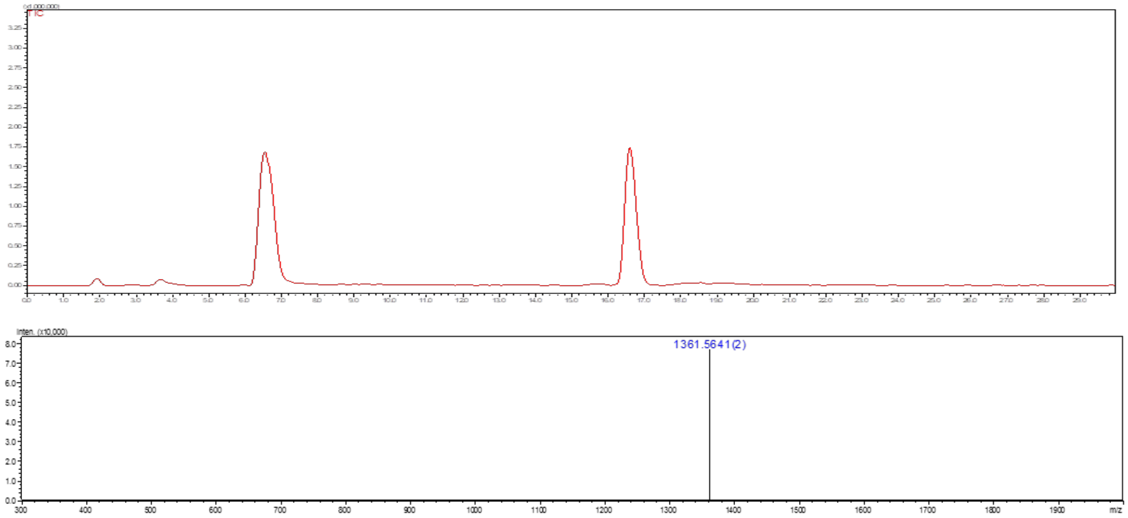

Figure S28. Liquid chromatography (LC) trace and mass (ESI-MS) spectrum (peak at retention time = 16.6 min) of **7**.

Compound F

The amine of *N*-glycan compound **7** was subjected for the hydrogenation using a general protocol **3k** to obtain **F** as a white solid.

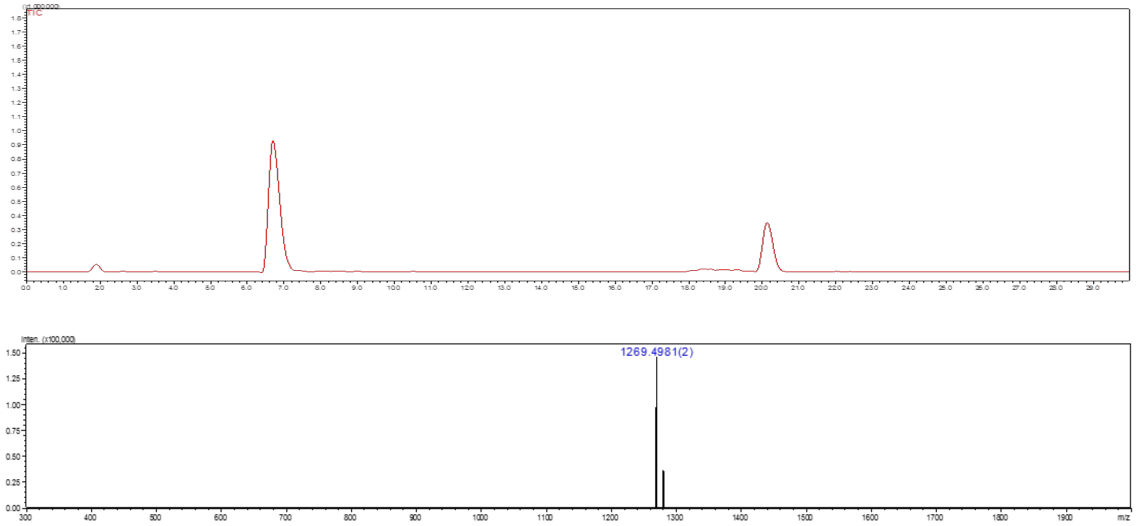

Figure S29. Liquid chromatography (LC) trace and mass (ESI-MS) spectrum (peak at retention time = 20.2 min) of **F**.

Compound 30

N-Glycan **4** (22 mg, 10.90 µmol) was dissolved in deionized water with the final reaction concentration of 5 mM. The pH of the reaction solution was adjusted to 10 using NaOH (1 M), and the mixture solution was incubated for 3-5 h at 37 °C with shaking. After complete conversion of starting material to amine intermediate **30** [confirmed by LC-ESI-MS, peak at retention time = 15.8 min, m/z = 959.5598(1)]. Then, reaction was neutralized by aqueous acetic acid (1 M). The product was purified by C18 biogel [Eluent - water and water:acetonitrile (8:2)]. Next, compound **30** was subjected for the installation of a β1,4 Gal using the general procedure **3f** to obtained **31** as a white solid which was subjected for the acetylation using a general protocol **3j** to obtain **32** as a white solid (11.9 mg, 55% over 3 steps).

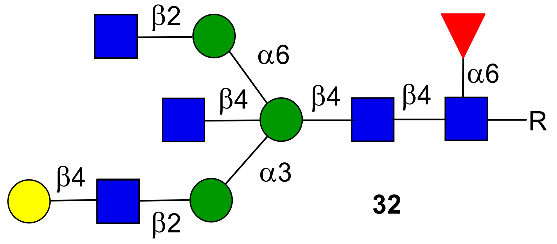

32

^{1}H NMR (600 MHz, D_2O): δ (ppm)

	**H1**	**H2**	**H3**	**H4**	**H5**	**H6**	**NHAc**
GlcNAc**1**	4.86	3.70	3.60	3.44	3.38	N/R[a]	2.02-1.79 (15H)
GlcNAc**2**	4.54	3.73	3.65	3.64	N/R	N/R	2.02-1.79 (15H)
GlcNAc**3**	4.50	3.69	3.54	N/R	N/R	N/R	2.02-1.79 (15H)
GlcNAc**4**	4.39	3.63	3.33	3.19	3.49	N/R	2.02-1.79 (15H)
GlcNAc**5**	4.48	3.64	3.41	N/R	N/R	N/R	2.02-1.79 (15H)
Man**1**	4.61	4.11	3.80	3.66	N/R	N/R	-
Man**2**	4.98	4.18	3.83	3.63	N/R	3.52	-
Man**3**	4.93	4.07	3.76	3.55	3.40	N/R	-
Fuc**1**	4.73	3.67	3.72	3.62	3.94	1.06	-
Gal**1**	4.40	3.48	3.59	3.85	N/R	N/R	-

**Signal**	**Proton**	**Carbon**
**Aromatic**	7.92-7.84 (m, 4H)<br>7.53-7.48 (m, 3H)	128.3-125.7
**CH_2**Ph	5.33 (d, J = 12.8 Hz)<br>5.14 (d, J = 12.7 Hz)	66.8
NH-**CH**-COOH	4.29 (dd, J = 9.5, 3.6 Hz)	53.1
C(O)-**CH_2**-CH	2.75 (dd, J = 14.8, 2.9 Hz)<br>2.51 (dd, J = 15.0, 9.9 Hz)	38.6

^{13}C NMR (150 MHz, D_2O): δ (ppm)

	**C1**
GlcNAc**1**	78.2
GlcNAc**2**	100.9
GlcNAc**3**	99.3
GlcNAc**4**	100.5
GlcNAc**5**	99.9
Man**1**	100.0
Man**2**	99.8
Man**3**	97.7
Fuc**1**	99.2
Gal**1**	103.0

[a] Not reported

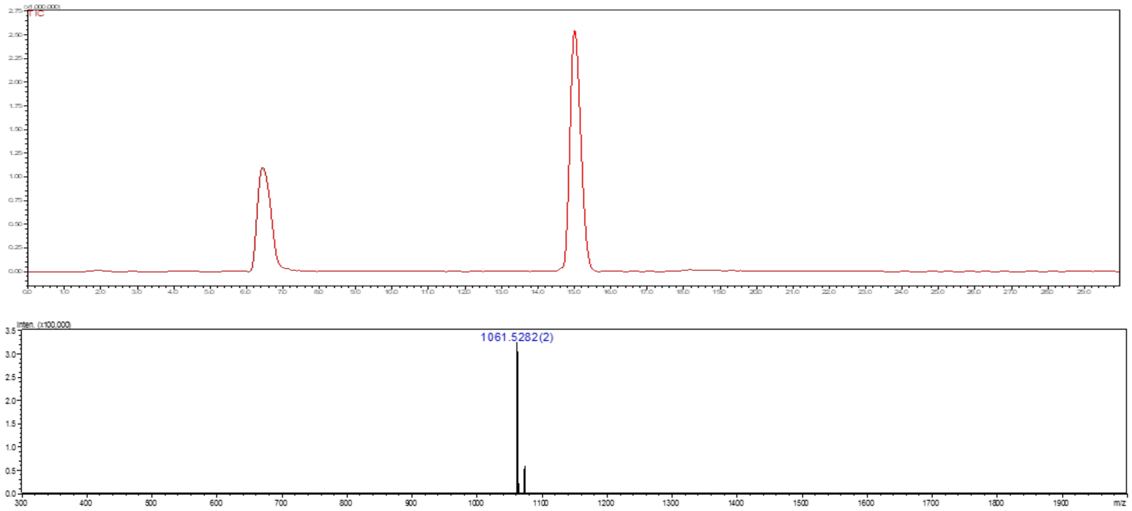

Figure S30. Liquid chromatography (LC) trace and mass (ESI-MS) spectrum (peak at retention time = 15.0 min) of **32**.

Compound G

The amine of *N*-glycan compound **32** was subjected for the hydrogenation using a general protocol **3k** to obtain **G** as a white solid.

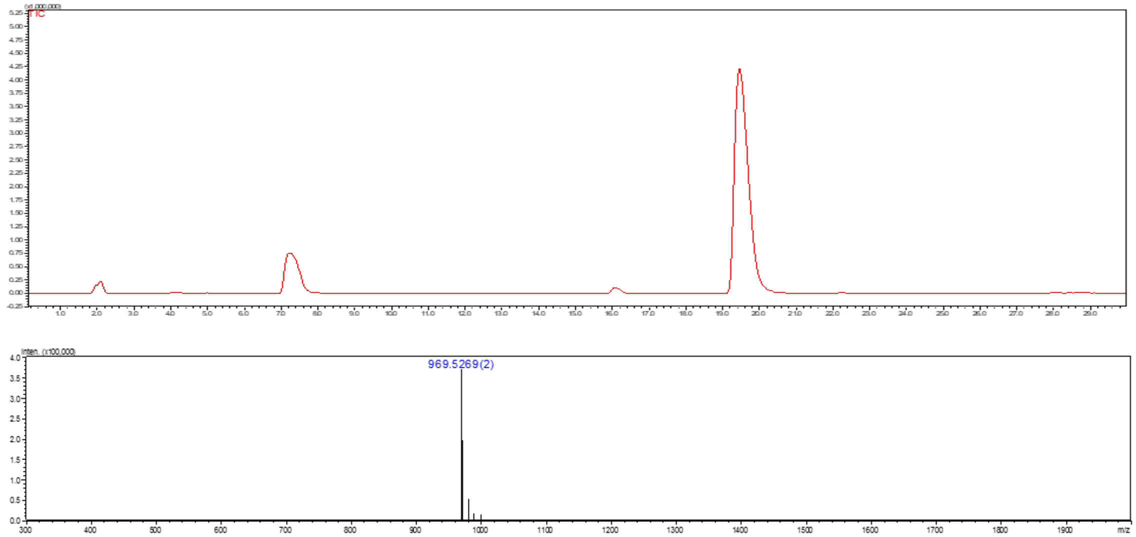

Figure S31. Liquid chromatography (LC) trace and mass (ESI-MS) spectrum (peak at retention time = 19.7 min) of **G**.

Compound 33

33 was synthesized from **32** (10 mg, 4.69 μmol) following the general protocol **3g** for the installation of Neu5Ac moiety using ST3Gal4. The product **33** was obtained as a white fluffy solid (9.0 mg, 79%).

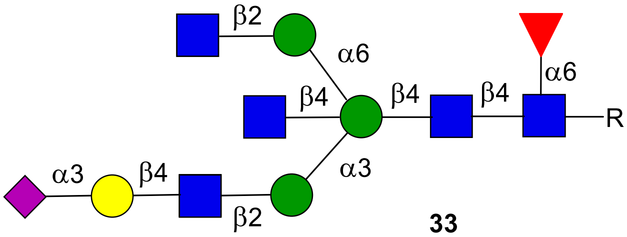

33

^{1}H NMR (600 MHz, D_2O): δ (ppm)

	H1	H2	H3	H4	H5	H6	NHAc
GlcNAc**1**	4.85	3.70	3.60	3.44	N/R[a]	N/R	2.02-1.79 (15H)
GlcNAc**2**	3.54	3.72	3.64	3.64	3.48	N/R	2.02-1.79 (15H)
GlcNAc**3**	4.50	3.68	3.49	N/R	N/R	N/R	2.02-1.79 (15H)
GlcNAc**4**	4.38	3.62	3.34	3.18	3.50	N/R	2.02-1.79 (15H)
GlcNAc**5**	4.47	3.64	3.54	N/R	N/R	N/R	2.02-1.79 (15H)
Man**1**	4.62	4.10	3.80	3.98	3.47	N/R	-
Man**2**	4.98	4.18	3.83	3.62	N/R	N/R	-
Man**3**	4.92	4.08	3.78	3.54	3.41	N/R	-
Fuc**1**	4.73	3.66	3.72	3.61	3.94	1.05	-
Gal**1**	4.47	3.49	4.04	3.89	N/R	N/R	-

	H1	H2	H3	H4	H5	H6	H7	H8	H9
Neu5Ac9	-	-	2.68<br>1.73	3.61	3.77	N/R	N/R	N/R	N/R

Signal	Proton	Carbon
**Aromatic**	7.92-7.85 (m, 4H)<br>7.52-7.48 (m, 3H)	128.4-125.8
**CH**$_2$Ph	5.33 (d, *J* = 12.8 Hz)<br>5.14 (d, *J* = 12.7 Hz)	66.7
NH-**CH**-COOH	4.29 (dd, *J* = 9.5, 3.6 Hz)	53.1
C(O)-**CH**$_2$-CH	2.75 (dd, *J* = 14.8, 2.9 Hz)<br>2.51 (dd, *J* = 15.0, 9.9 Hz)	38.7

^{13}C NMR (150 MHz, D_2O): δ (ppm)

	**C1**
GlcNAc**1**	78.0
GlcNAc**2**	100.8
GlcNAc**3**	99.7
GlcNAc**4**	100.6
GlcNAc**5**	99.5
Man**1**	100.1
Man**2**	99.9
Man**3**	97.6
Fuc**1**	99.2
Gal**1**	102.6

[a] Not reported

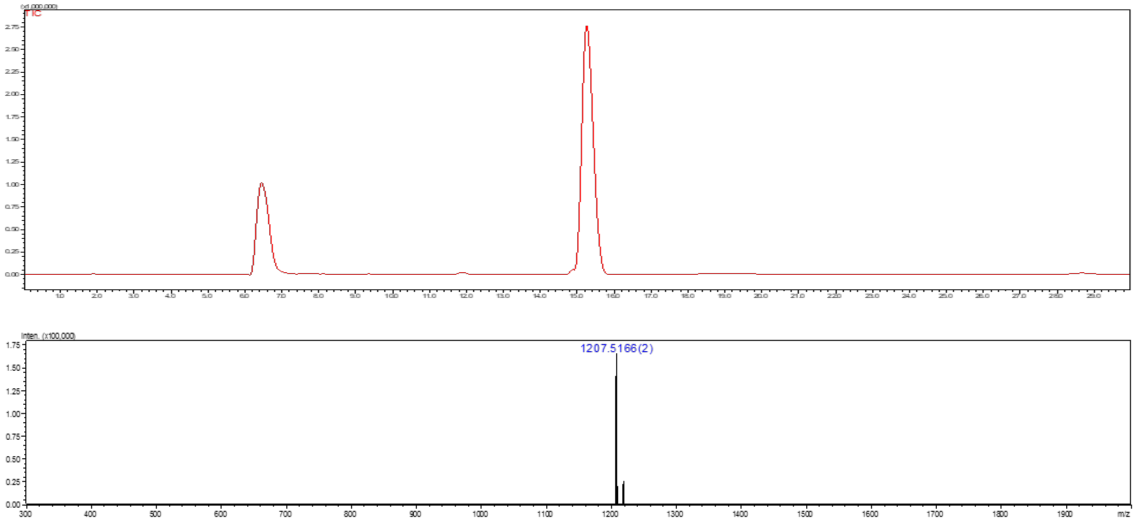

Figure S32. Liquid chromatography (LC) trace and mass (ESI-MS) spectrum (peak at retention time = 15.4 min) of **33**.

Compound H

The amine of *N*-glycan compound **33** was subjected for the hydrogenation using a general protocol **3k** to obtain **H** as a white solid.

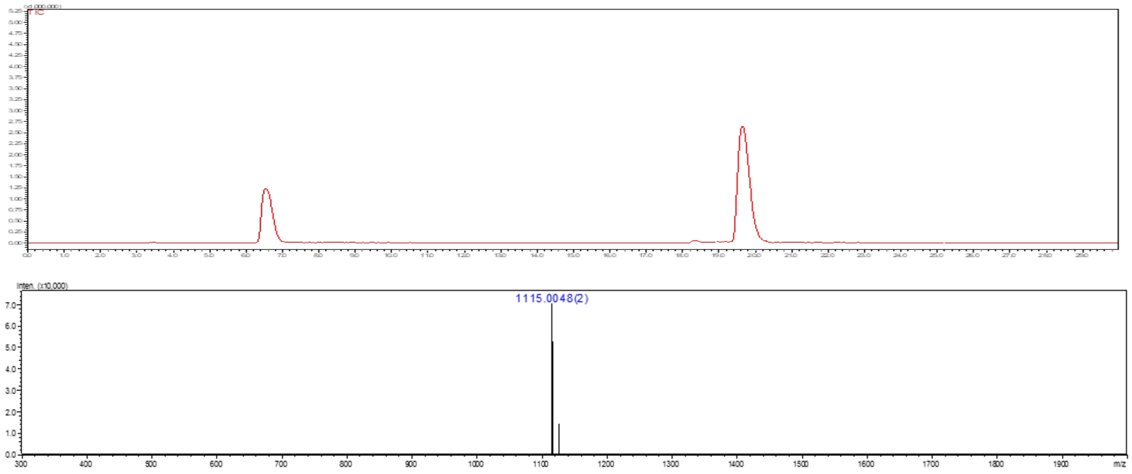

Figure S33. Liquid chromatography (LC) trace and mass (ESI-MS) spectrum (peak at retention time = 19.3 min) of **H**.

Compound 34

34 was synthesized from **32** (3 mg, 1.40 µmol) following the general protocol **3g** for the installation of Neu5Ac moiety using ST6Gal1. The product **34** was obtained as a white fluffy solid (3.0 mg, 88%).

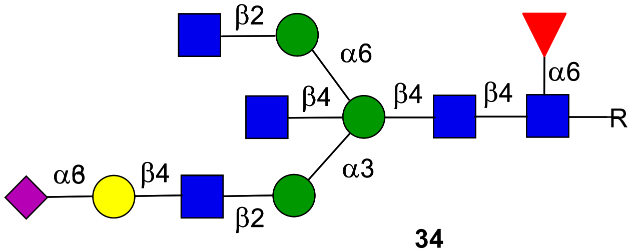

34

^{1}H NMR (600 MHz, D_2O): δ (ppm)

	**H1**	**H2**	**H3**	**H4**	**H5**	**H6**	**NHAc**
GlcNAc**1**	4.85	3.69	3.60	3.43	N/R[a]	N/R	2.02-1.79 (15H)
GlcNAc**2**	4.54	3.73	3.62	N/R	3.48	N/R	2.02-1.79 (15H)
GlcNAc**3**	4.54	3.69	3.49	N/R	N/R	N/R	2.02-1.79 (15H)
GlcNAc**4**	4.39	3.61	3.34	3.19	3.49	N/R	2.02-1.79 (15H)
GlcNAc**5**	4.47	3.63	3.41	3.34	N/R	N/R	2.02-1.79 (15H)
Man**1**	4.62	4.10	3.80	3.99	3.48	N/R	**-**
Man**2**	5.00	4.19	3.83	3.63	N/R	N/R	**-**
Man**3**	4.92	4.08	3.77	3.54	3.40	N/R	**-**
Fuc**1**	4.73	3.66	3.71	3.60	3.95	1.06	**-**
Gal**1**	4.37	3.45	N/R	3.86	N/R	N/R	**-**

	**H1**	**H2**	**H3**	**H4**	**H5**	**H6**	**H7**	**H8**	**H9**
Neu5Ac9	**-**	**-**	2.59<br>1.65	3.58	3.73	N/R	N/R	N/R	N/R

**Signal**	**Proton**	**Carbon**
**Aromatic**	7.92-7.85 (m, 4H)<br>7.52-7.48 (m, 3H)	128.4-125.7
**CH_2**Ph	5.33 (d, J = 12.8 Hz)<br>5.14 (d, J = 12.7 Hz)	66.9
NH-**CH**-COOH	4.29 (dd, J = 9.5, 3.6 Hz)	53.1
C(O)-**CH_2**-CH	2.75 (dd, J = 14.8, 2.9 Hz)<br>2.51 (dd, J = 15.0, 9.9 Hz)	38.6

^{13}C NMR (150 MHz, D_2O): δ (ppm)

	**C1**
GlcNAc**1**	78.2
GlcNAc**2**	100.9
GlcNAc**3**	99.4
GlcNAc**4**	99.4
GlcNAc**5**	100.6
Man**1**	100.1
Man**2**	99.8
Man**3**	97.7
Fuc**1**	99.2
Gal**1**	103.4

[a] Not reported

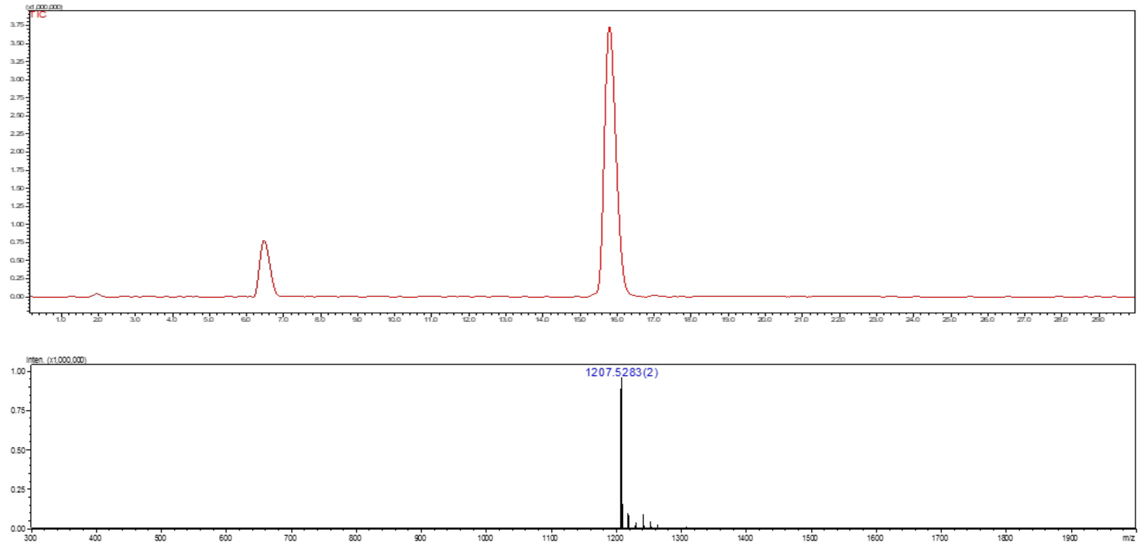

Figure S34. Liquid chromatography (LC) trace and mass (ESI-MS) spectrum (peak at retention time = 15.9 min) of **34**.

Compound I

The amine of *N*-glycan compound **34** was subjected for the hydrogenation using a general protocol **3k** to obtain **I** as a white solid.

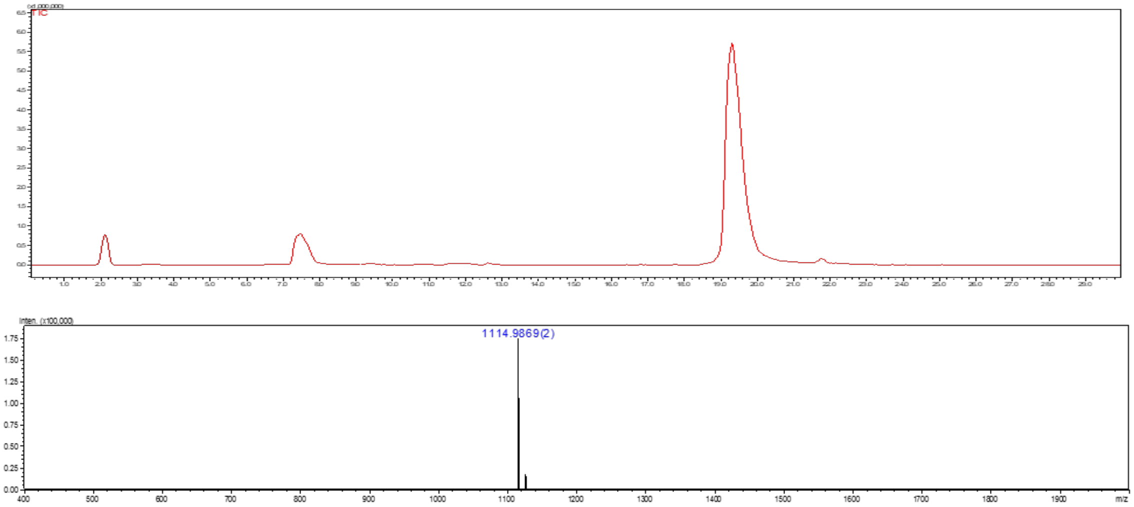

Figure S35. Liquid chromatography (LC) trace and mass (ESI-MS) spectrum (peak at retention time = 19.4 min) of **I**.

Compound 35

35 was synthesized from **33** (4 mg, 1.65 µmol) following the general protocol **3h** for the installation of Fuc moiety using FUT6. The product **35** was obtained as a white fluffy solid (3.5 mg, 84%).

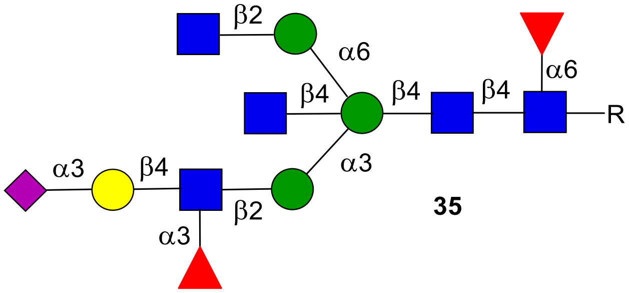

35

^{1}H NMR (600 MHz, D_2O): δ (ppm)

	**H1**	**H2**	**H3**	**H4**	**H5**	**H6**	**NHAc**
GlcNAc**1**	4.86	3.70	3.60	3.45	N/R[a]	N/R	2.02-1.79 (15H)
GlcNAc**2**	4.55	3.72	3.65	3.65	3.49	N/R	2.02-1.79 (15H)
GlcNAc**3**	4.51	3.87	3.52	N/R	N/R	N/R	2.02-1.79 (15H)
GlcNAc**4**	4.38	3.63	3.36	3.18	3.19	N/R	2.02-1.79 (15H)
GlcNAc**5**	4.47	3.64	3.41	3.40	N/R	N/R	2.02-1.79 (15H)
Man**1**	4.61	4.10	3.79	3.97	3.48	N/R	-
Man**2**	4.96	4.17	3.82	3.62	3.38	N/R	-
Man**3**	4.92	4.08	3.78	3.55	3.41	N/R	-
Fuc**1**	4.73	3.66	3.72	3.62	3.94	1.09	-
Gal**1**	4.45	3.46	4.03	3.88	N/R	N/R	-
Fuc**2**	5.06	3.61	3.89	3.69	4.75	1.09	-

	**H1**	**H2**	**H3**	**H4**	**H5**	**H6**	**H7**	**H8**	**H9**
Neu5Ac9	-	-	2.68<br>1.71	3.60	3.77	N/R	N/R	N/R	N/R

**Signal**	**Proton**	**Carbon**
**Aromatic**	7.92-7.85 (m, 4H)<br>7.52-7.47 (m, 3H)	128.4-125.7
**CH_2**Ph	5.32 (d, *J* = 12.8 Hz)<br>5.13 (d, *J* = 12.7 Hz)	66.8
NH-**CH**-COOH	4.29 (dd, *J* = 9.5, 3.6 Hz)	53.0
C(O)-**CH_2**-CH	2.75 (dd, *J* = 14.8, 2.9 Hz)<br>2.51 (dd, *J* = 15.0, 9.9 Hz)	38.7

^{13}C NMR (150 MHz, D_2O): δ (ppm)

	**C1**
GlcNAc**1**	78.2
GlcNAc**2**	100.9
GlcNAc**3**	99.4
GlcNAc**4**	100.7
GlcNAc**5**	99.5
Man**1**	100.2
Man**2**	99.7
Man**3**	97.7
Fuc**1**	99.2
Gal**1**	101.7
Fuc**2**	98.5

[a] Not reported

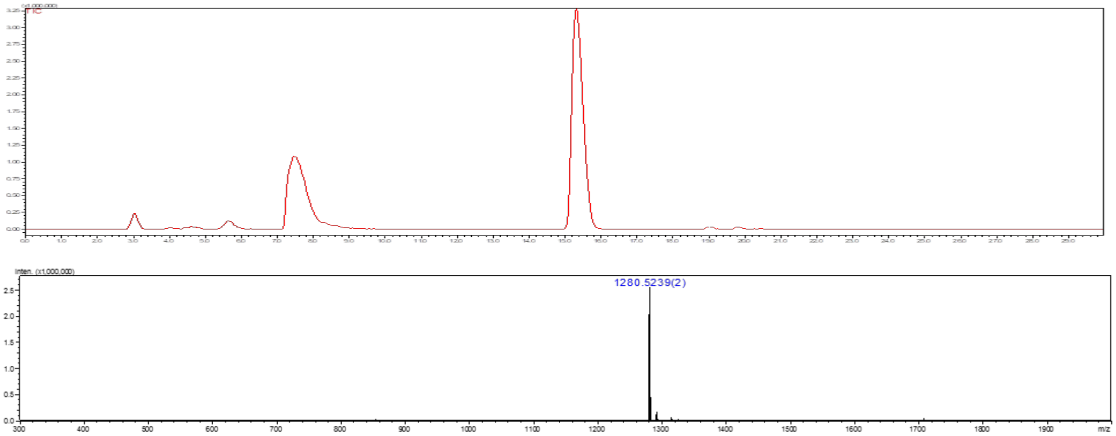

Figure S36. Liquid chromatography (LC) trace and mass (ESI-MS) spectrum (peak at retention time = 15.5 min) of **35**.

Compound K

The amine of *N*-glycan compound **35** was subjected for the hydrogenation using a general protocol **3k** to obtain **K** as a white solid.

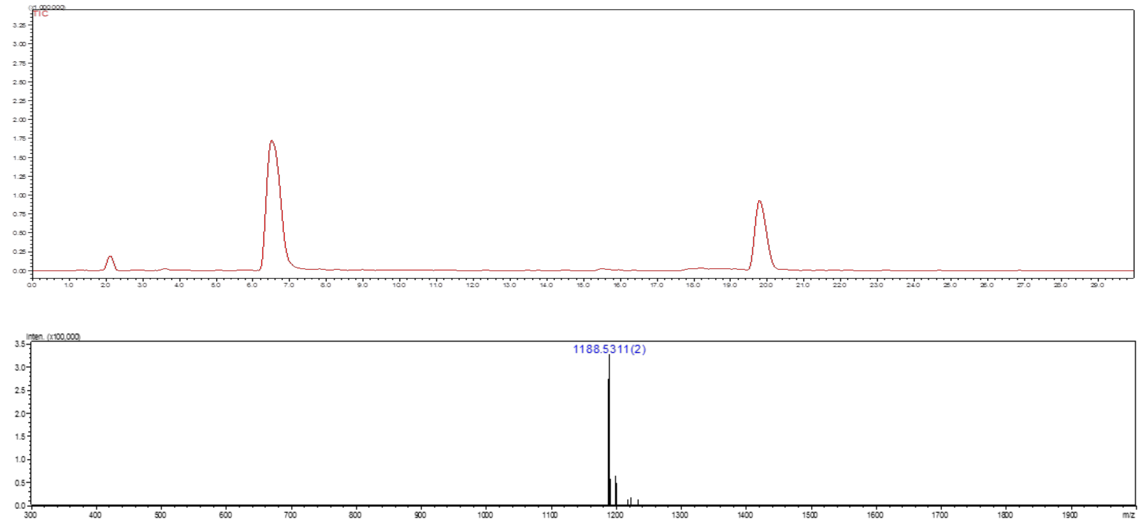

Figure S37. Liquid chromatography (LC) trace and mass (ESI-MS) spectrum (peak at retention time = 19.8 min) of **K**.

Compound 39

36 was synthesized from **33** (3 mg, 1.24 μmol) following the general protocol **3f** for the installation of Gal moiety using B4GalT1. The product **36** was obtained as a white fluffy solid (2.30 mg, 71%).

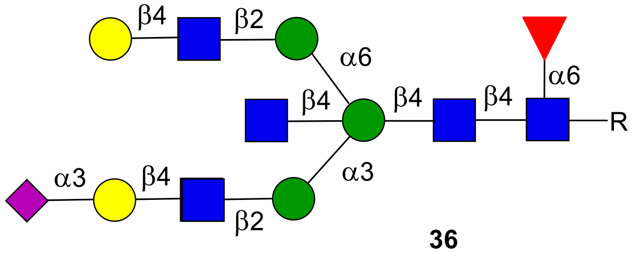

36

^{1}H NMR (900 MHz, D_2O): δ (ppm)

	**H1**	**H2**	**H3**	**H4**	**H5**	**H6**	**NHAc**
GlcNAc**1**	4.87	3.70	3.60	N/R[a]	N/R	N/R	2.02-1.79 (15H)
GlcNAc**2**	4.55	3.73	3.62	3.66	3.48	N/R	2.02-1.79 (15H)
GlcNAc**3**	4.52	3.70	3.53	N/R	N/R	N/R	2.02-1.79 (15H)
GlcNAc**4**	4.38	3.62	3.33	3.19	3.49	N/R	2.02-1.79 (15H)
GlcNAc**5**	4.51	3.68	3.56	3.46	N/R	N/R	2.02-1.79 (15H)
Man**1**	4.61	4.10	3.80	4.01	3.46	N/R	**-**
Man**2**	4.99	4.18	3.83	3.63	N/R	N/R	**-**
Man**3**	4.94	4.07	3.78	3.55	3.40	N/R	**-**
Fuc**1**	4.74	3.67	3.72	3.61	3.95	1.07	**-**
Gal**1**	4.47	3.49	4.03	3.88	N/R	N/R	**-**
Gal**2**	4.39	3.48	N/R	3.87	N/R	N/R	**-**

	**H1**	**H2**	**H3**	**H4**	**H5**	**H6**	**H7**	**H8**	**H9**
Neu5Ac9	**-**	**-**	2.68<br>1.72	3.60	3.77	N/R	N/R	N/R	N/R

**Signal**	**Proton**	**Carbon**
**Aromatic**	7.92-7.85 (m, 4H)<br>7.52-7.48 (m, 3H)	128.4-125.7
**CH_2**Ph	5.32 (d, J = 12.8 Hz)<br>5.14 (d, J = 12.7 Hz)	66.7
NH-**CH**-COOH	4.32 (dd, J = 9.5, 3.6 Hz)	53.0
C(O)-**CH_2**-CH	2.75 (dd, J = 14.8, 2.9 Hz)<br>2.54 (dd, J = 15.0, 9.9 Hz)	38.6

^{13}C NMR (225 MHz, D_2O): δ (ppm)

	**C1**
GlcNAc**1**	78.2
GlcNAc**2**	100.9
GlcNAc**3**	99.7
GlcNAc**4**	100.6
GlcNAc**5**	99.4
Man**1**	100.0
Man**2**	99.9
Man**3**	97.6
Fuc**1**	99.2
Gal**1**	102.9
Gal**2**	102.6

[a] Not reported

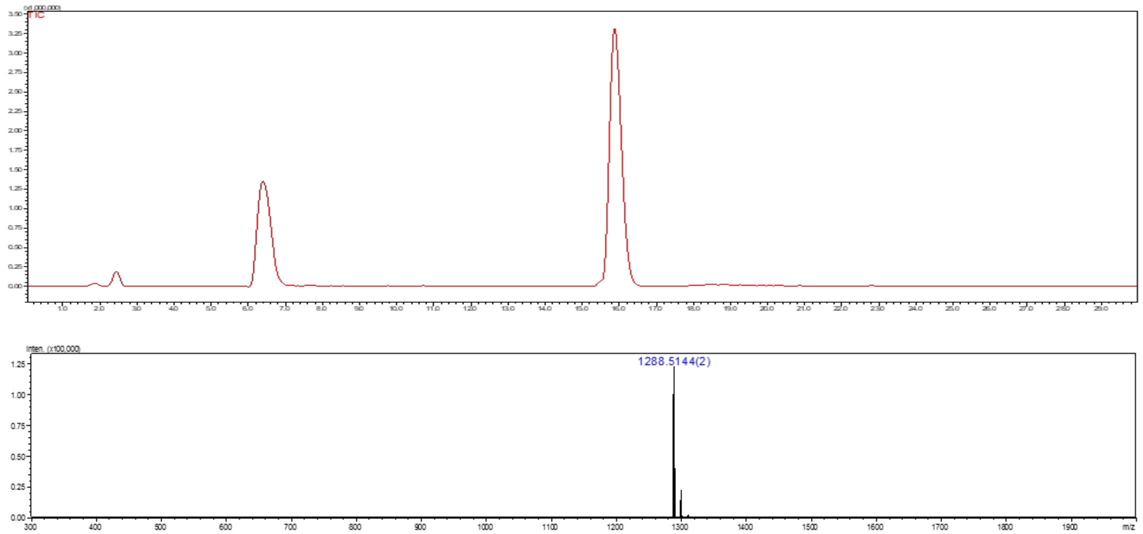

Figure S38. Liquid chromatography (LC) trace and mass (ESI-MS) spectrum (peak at retention time = 15.9 min) of **36**.

Compound J

The amine of *N*-glycan compound **36** was subjected for the hydrogenation using a general protocol **3k** to obtain **J** as a white solid.

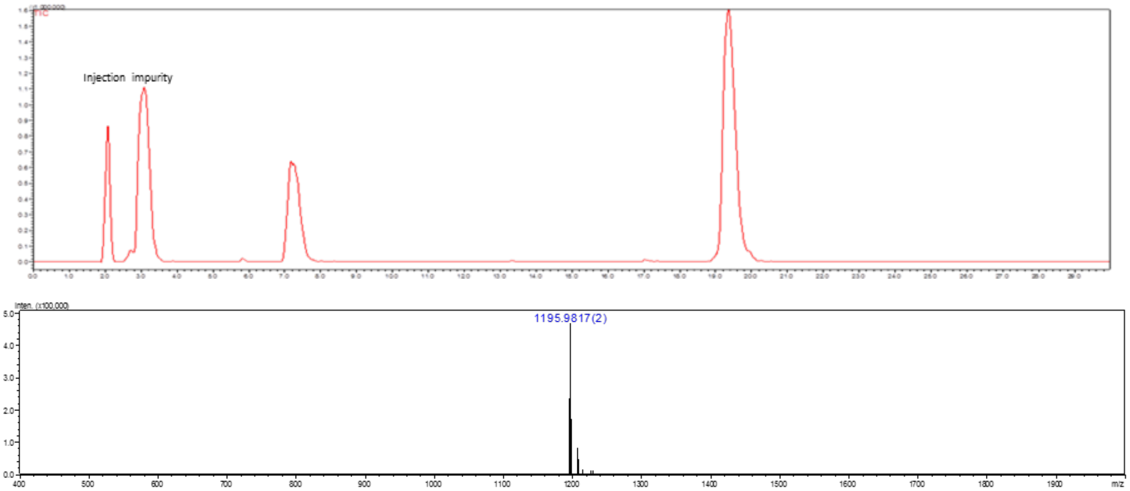

Figure S39. Liquid chromatography (LC) trace and mass (ESI-MS) spectrum (peak at retention time = 19.5 min) of **J**.

Compound 5

5 was synthesized from **35** (3 mg, 1.16 µmol) following the general protocol **3f** for the installation of Gal moiety using B4GalT1. The product **5** was obtained as a white fluffy solid (2.1 mg, 66%).

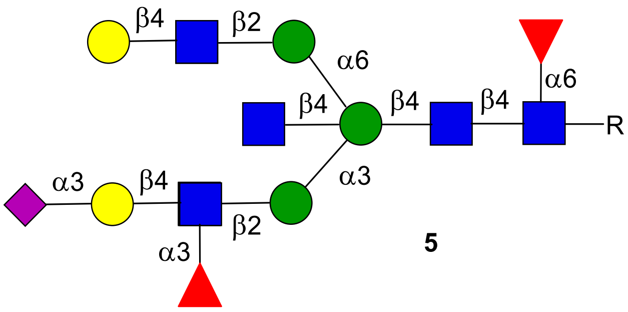

^{1}H NMR (600 MHz, D_2O): δ (ppm)

	H1	H2	H3	H4	H5	H6	NHAc
GlcNAc**1**	4.86	3.70	3.60	N/R[a]	N/R	N/R	2.02-1.80 (15H)
GlcNAc**2**	4.54	3.73	3.62	3.66	3.48	N/R	2.02-1.80 (15H)
GlcNAc**3**	4.51	3.86	3.54	N/R	N/R	N/R	2.02-1.80 (15H)
GlcNAc**4**	4.38	3.62	3.34	3.18	3.50	N/R	2.02-1.80 (15H)
GlcNAc**5**	4.50	3.67	3.57	3.46	N/R	N/R	2.02-1.80 (15H)
Man**1**	4.61	4.10	3.80	3.97	3.47	N/R	**-**
Man**2**	4.96	4.18	3.83	3.63	N/R	N/R	**-**
Man**3**	4.94	4.06	3.77	3.55	3.41	N/R	**-**
Fuc**1**	4.73	3.68	3.72	3.60	3.94	1.06	**-**
Fuc**2**	5.06	3.61	N/R	3.67	4.74	1.09	**-**
Gal**1**	4.39	3.47	N/R	3.85	N/R	N/R	**-**
Gal**2**	4.45	3.46	4.01	3.87	N/R	N/R	**-**

	H1	H2	H3	H4	H5	H6	H7	H8	H9
Neu5Ac9	**-**	**-**	2.67<br>1.71	3.60	3.77	N/R	N/R	N/R	N/R

Signal	Proton	Carbon
**Aromatic**	7.91-7.85 (m, 4H)<br>7.53-7.48 (m, 3H)	128.3-125.6
**CH_2**Ph	5.35 (d, J = 12.8 Hz)<br>5.15 (d, J = 12.7 Hz)	66.8
NH-**CH**-COOH	4.35 (dd, J = 9.5, 3.6 Hz)	52.6
C(O)-**CH_2**-CH	2.76 (dd, J = 14.8, 2.9 Hz)<br>2.55 (dd, J = 15.0, 9.9 Hz)	38.4

^{13}C NMR (150 MHz, D_2O): δ (ppm)

	**C1**
GlcNAc**1**	78.2
GlcNAc**2**	100.8
GlcNAc**3**	99.4
GlcNAc**4**	100.6
GlcNAc**5**	99.4
Man**1**	100.0
Man**2**	99.8
Man**3**	97.6
Fuc**1**	99.1
Gal**1**	102.9
Gal**2**	101.7
Fuc**2**	98.5

[a] Not reported

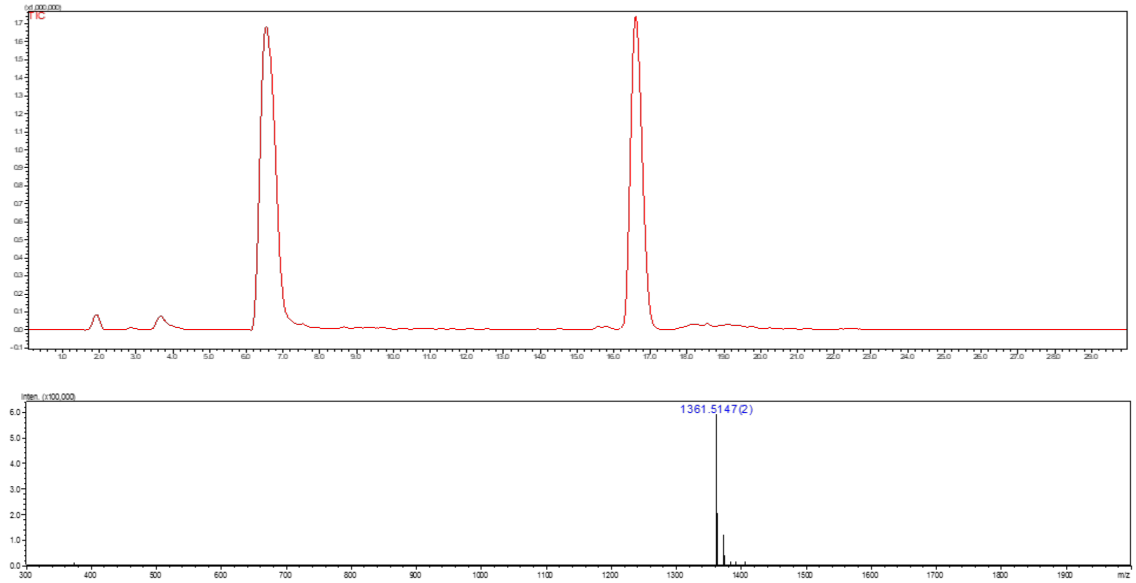

Figure S40. Liquid chromatography (LC) trace and mass (ESI-MS) spectrum (peak at retention time = 16.6 min) of **5**.

Compound L

The amine of *N*-glycan compound **5** was subjected for the hydrogenation using a general protocol **3k** to obtain **L** as a white solid.

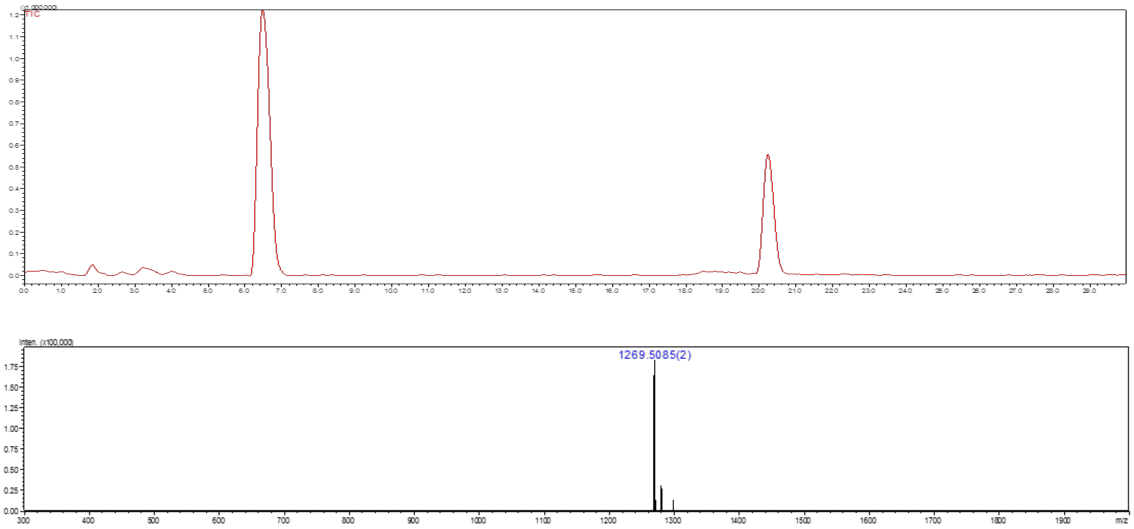

Figure S41. Liquid chromatography (LC) trace and mass (ESI-MS) spectrum (peak at retention time = 20.3 min) of **L**.

Compound 38

N-Glycan **22** (17 mg, 7.99 µmol) was dissolved in deionized water with the final reaction concentration of 5 mM. The pH of the reaction solution was adjusted to 10 using NaOH (1 M), and the mixture solution was incubated for 3-5 h at 37 °C with shaking. After complete conversion of starting material to amine intermediate **8** [confirmed by LC-ESI-MS]. Then, reaction was neutralized by aqueous acetic acid (1 M). The product was purified by C18 biogel [Eluent - water and water:acetonitrile (8:2)]. Next, compound **8** was subjected for the installation of a β1,4 Gal using the general procedure **3f** to obtained **37** as a white solid which was subjected for the acetylation using a general protocol **3j** to obtain **38** as a white solid (9.5 mg, 49% over 3 steps).

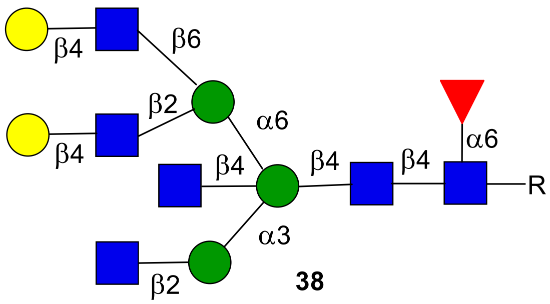

38

^{1}H NMR (900 MHz, D_2O): δ (ppm)

	H1	H2	H3	H4	H5	H6	NHAc
GlcNAc**1**	4.86	3.69	3.45	3.35	N/R[a]	N/R	2.02-1.79 (18H)
GlcNAc**2**	4.53	3.71	3.43	N/R	N/R	N/R	2.02-1.79 (18H)
GlcNAc**3**	4.54	3.64	3.56	3.44	N/R	N/R	2.02-1.79 (18H)
GlcNAc**4**	4.42	3.62	3.30	3.20	3.53	3.40	2.02-1.79 (18H)
GlcNAc**5**	4.51	3.64	3.54	3.42	N/R	N/R	2.02-1.79 (18H)
GlcNAc**6**	4.46	3.71	3.65	3.54	N/R	N/R	2.02-1.79 (18H)
Man**1**	4.62	4.09	3.80	3.43	N/R	N/R	-
Man**2**	4.99	4.16	3.82	3.40	3.52	N/R	-
Man**3**	4.83	4.04	3.72	3.34	3.61	N/R	-
Fuc**1**	4.74	3.65	3.71	3.60	3.93	1.05	-
Gal**1**	4.40	3.47	3.60	3.85	N/R	N/R	-
Gal**2**	4.41	3.48	3.61	3.85	N/R	N/R	-

Signal	Proton	Carbon
**Aromatic**	7.92-7.84 (m, 4H)<br>7.52-7.47 (m, 3H)	128.4-125.7
**CH$_2$**Ph	5.32 (d, *J* = 12.8 Hz)<br>5.14 (d, *J* = 12.7 Hz)	66.7
NH-**CH**-COOH	4.29 (dd, *J* = 9.5, 3.6 Hz)	53.0
C(O)-**CH$_2$**-CH	2.74 (dd, *J* = 14.8, 2.9 Hz)<br>2.51 (dd, *J* = 15.0, 9.9 Hz)	38.6

^{13}C NMR (225 MHz, D_2O): δ (ppm)

	**C1**
GlcNAc**1**	78.1
GlcNAc**2**	101.0
GlcNAc**3**	99.6
GlcNAc**4**	99.8
GlcNAc**5**	99.9
GlcNAc**6**	101.6
Man**1**	99.8
Man**2**	99.9
Man**3**	97.8
Fuc**1**	99.2
Gal**1**	103.0
Gal**2**	103.0

[a] Not reported

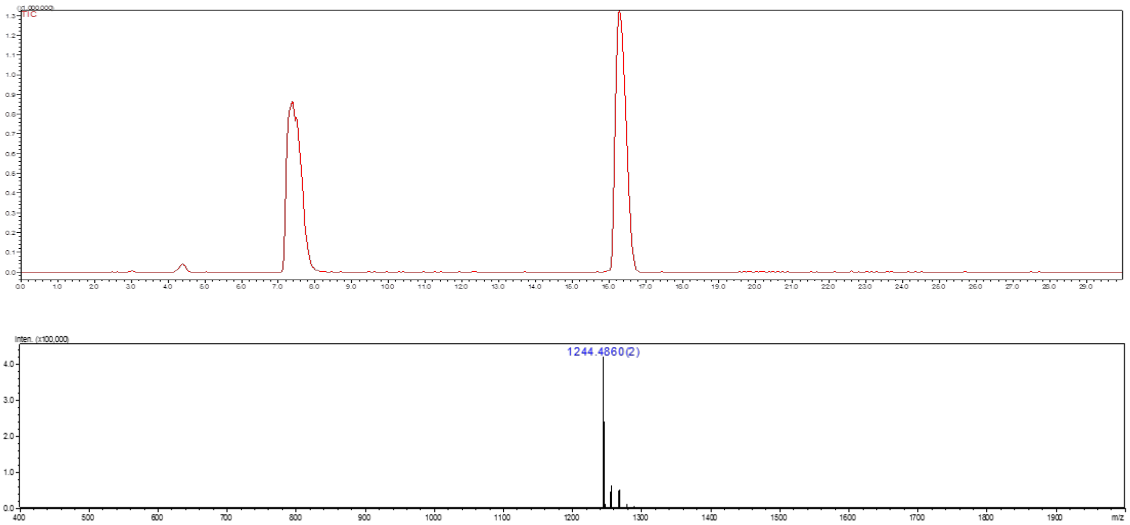

Figure S42. Liquid chromatography (LC) trace and mass (ESI-MS) spectrum (peak at retention time = 16.4 min) of **38**.

Compound M

The amine of *N*-glycan compound **38** was subjected for the hydrogenation using a general protocol **3k** to obtain **M** as a white solid.

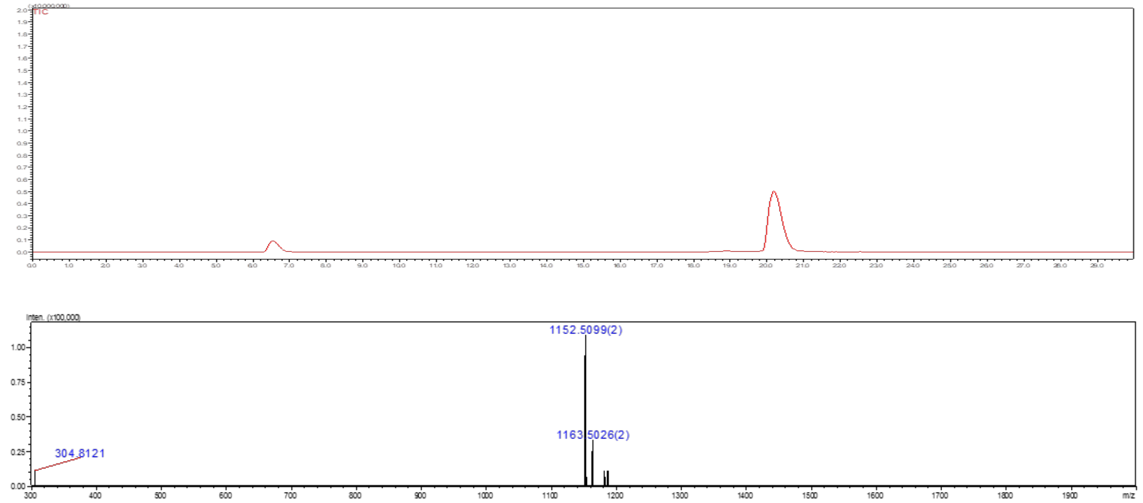

Figure S43. Liquid chromatography (LC) trace and mass (ESI-MS) spectrum (peak at retention time = 20.6 min) of **M**.

Compound 39

39 was synthesized from **38** (6.8 mg, 2.74 µmol) following the general protocol **3g** for the installation of Neu5Ac moiety using ST3Gal4. The product **39** was obtained as a white fluffy solid (7.7 mg, 92%).

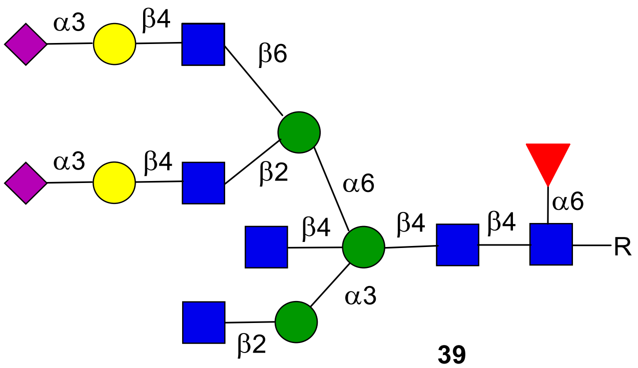

39

^{1}H NMR (600 MHz, D_2O): δ (ppm)

	H1	H2	H3	H4	H5	H6	NHAc
GlcNAc**1**	4.87	3.71	3.48	3.41	N/R[a]	N/R	2.02-1.79 (18H)
GlcNAc**2**	4.52	3.73	3.49	N/R	N/R	N/R	2.02-1.79 (18H)
GlcNAc**3**	4.52	3.66	3.55	3.43	N/R	N/R	2.02-1.79 (18H)
GlcNAc**4**	4.42	3.63	3.30	3.20	3.54	N/R	2.02-1.79 (18H)
GlcNAc**5**	4.52	3.66	3.55	3.43	N/R	N/R	2.02-1.79 (18H)
GlcNAc**6**	4.46	3.73	3.66	3.54	N/R	N/R	2.02-1.79 (18H)
Man**1**	4.63	4.10	3.79	3.43	N/R	N/R	-
Man**2**	4.99	4.17	3.83	3.39	3.62	N/R	-
Man**3**	4.83	4.05	3.72	3.34	N/R	N/R	-
Fuc**1**	4.75	3.67	3.71	3.60	3.94	1.06	-
Gal**1**	4.47	3.50	4.05	3.89	N/R	N/R	-
Gal**2**	4.48	3.51	4.05	3.89	N/R	N/R	-

	H1	H2	H3	H4	H5	H6	H7	H8	H9
Neu5Ac9-**1**	-	-	2.68<br>1.71	3.61	3.77	N/R	N/R	N/R	N/R
Neu5Ac9-**2**	-	-	2.68<br>1.71	3.61	3.77	N/R	N/R	N/R	N/R

Signal	Proton	Carbon
**Aromatic**	7.92-7.85 (m, 4H)<br>7.53-7.48 (m, 3H)	128.4-125.7
**CH_2**Ph	5.33 (d, *J* = 12.8 Hz)<br>5.13 (d, *J* = 12.7 Hz)	66.9
NH-**CH**-COOH	4.29 (dd, *J* = 9.5, 3.6 Hz)	52.4
C(O)-**CH_2**-CH	2.75 (dd, *J* = 14.8, 2.9 Hz)<br>2.51 (dd, *J* = 15.0, 9.9 Hz)	38.4

^{13}C NMR (150 MHz, D_2O): δ (ppm)

	**C1**
GlcNAc**1**	78.2
GlcNAc**2**	101.1
GlcNAc**3**	99.8
GlcNAc**4**	99.8
GlcNAc**5**	99.9
GlcNAc**6**	101.6
Man**1**	99.7
Man**2**	99.9
Man**3**	98.0
Fuc**1**	99.2
Gal**1**	102.6
Gal**2**	102.6

[a] Not reported

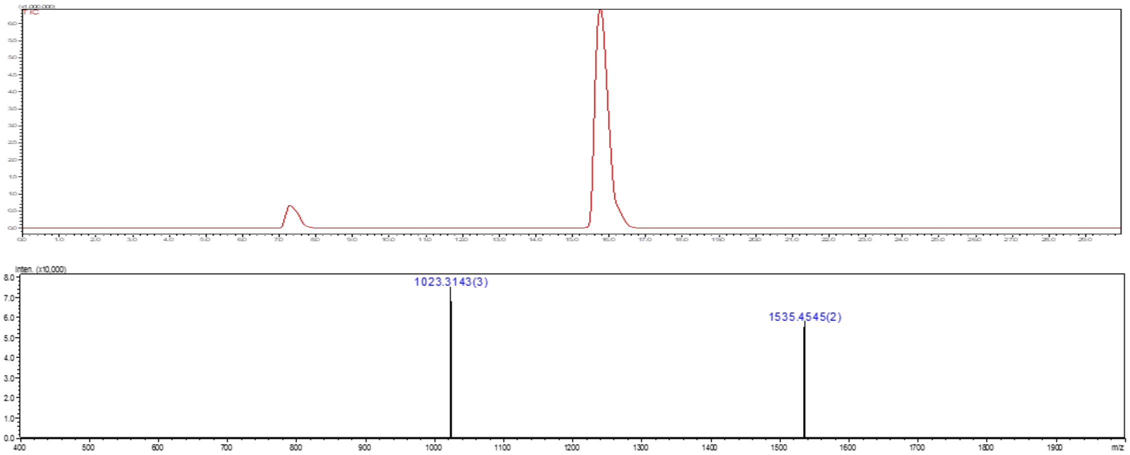

Figure S44. Liquid chromatography (LC) trace and mass (ESI-MS) spectrum (peak at retention time = 15.9 min) of **39**.

Compound N

The amine of *N*-glycan compound **39** was subjected for the hydrogenation using a general protocol **3k** to obtain **N** as a white solid.

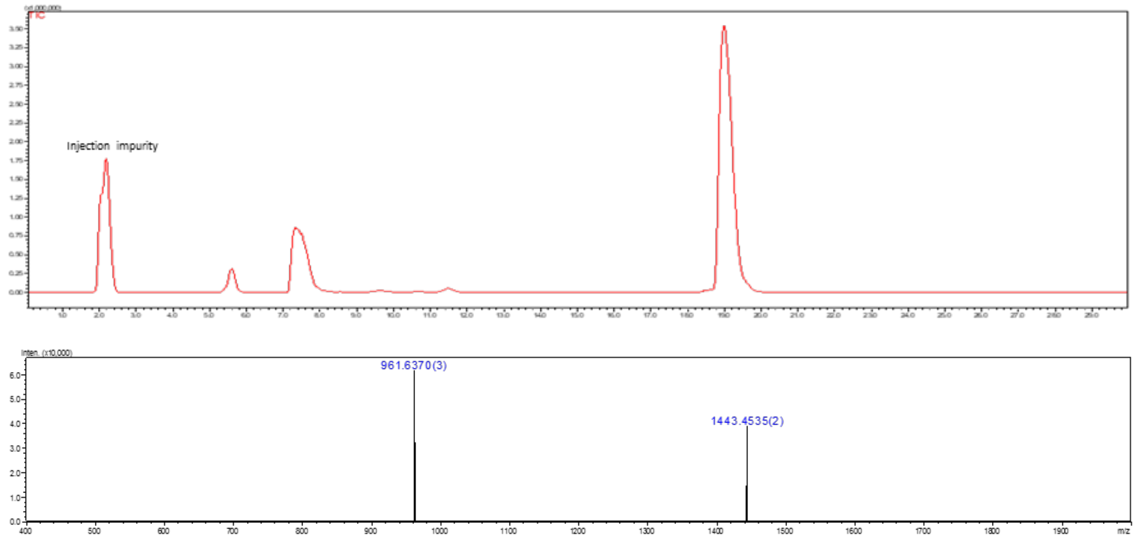

Figure S45. Liquid chromatography (LC) trace and mass (ESI-MS) spectrum (peak at retention time = 19.2 min) of **N**.

Compound 40

40 was synthesized from **39** (5.8 mg, 1.88 μmol) following the general protocol **3h** for the installation of Fuc moiety using FUT6. The product **40** was obtained as a white fluffy solid (5.4 mg, 86%).

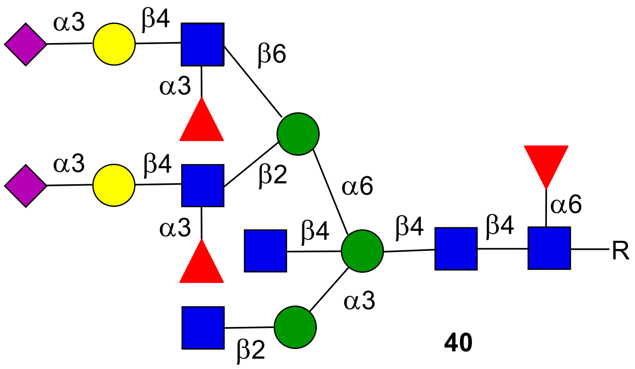

40

^{1}H NMR (900 MHz, D_2O): δ (ppm)

	**H1**	**H2**	**H3**	**H4**	**H5**	**H6**	**NHAc**
GlcNAc**1**	4.86	3.70	3.45	3.40	N/R[a]	N/R	2.03-1.79 (18H)
GlcNAc**2**	4.53	3.72	3.49	N/R	N/R	N/R	2.03-1.79 (18H)
GlcNAc**3**	4.53	3.64	3.54	3.44	N/R	N/R	2.03-1.79 (18H)
GlcNAc**4**	4.46	3.63	3.34	3.22	3.51	N/R	2.03-1.79 (18H)
GlcNAc**5**	4.53	3.64	3.54	3.41	N/R	N/R	2.03-1.79 (18H)
GlcNAc**6**	4.46	3.85	3.63	N/R	N/R	N/R	2.03-1.79 (18H)
Man**1**	4.62	4.08	3.77	3.52	N/R	N/R	-
Man**2**	4.96	4.15	3.84	3.35	3.60	N/R	-
Man**3**	4.83	4.06	3.74	3.33	N/R	N/R	-
Fuc**1**	4.73	3.66	3.71	3.61	3.94	1.06	-
Gal**1**	4.45	3.45	4.01	3.85	N/R	N/R	-
Gal**2**	4.45	3.45	4.01	3.85	N/R	N/R	-
Fuc**2**	5.04	3.62	3.71	3.61	4.76	1.11	-
Fuc**3**	5.08	3.62	3.71	3.61	4.76	1.11	-

	**H1**	**H2**	**H3**	**H4**	**H5**	**H6**	**H7**	**H8**	**H9**
Neu5Ac9-**1**	-	-	2.68<br>1.72	3.60	1.73	N/R	N/R	N/R	N/R
Neu5Ac9-**2**	-	-	2.68<br>1.72	3.60	1.73	N/R	N/R	N/R	N/R

**Signal**	**Proton**	**Carbon**
**Aromatic**	7.91-7.85 (m, 4H)<br>7.53-7.48 (m, 3H)	128.3-125.7
**CH_2**Ph	5.33 (d, *J* = 12.8 Hz)<br>5.14 (d, *J* = 12.7 Hz)	66.8
NH-**CH**-COOH	4.29 (dd, *J* = 9.5, 3.6 Hz)	53.0
C(O)-**CH_2**-CH	2.76 (dd, *J* = 14.8, 2.9 Hz)<br>2.51 (dd, *J* = 15.0, 9.9 Hz)	38.7

^{13}C NMR (225 MHz, D_2O): δ (ppm)

	**C1**
GlcNAc**1**	78.1
GlcNAc**2**	101.0
GlcNAc**3**	99.8
GlcNAc**4**	99.2
GlcNAc**5**	99.9
GlcNAc**6**	101.0
Man**1**	100.1
Man**2**	99.9
Man**3**	97.7
Fuc**1**	99.2
Gal**1**	101.5
Gal**2**	101.5

[a] Not reported

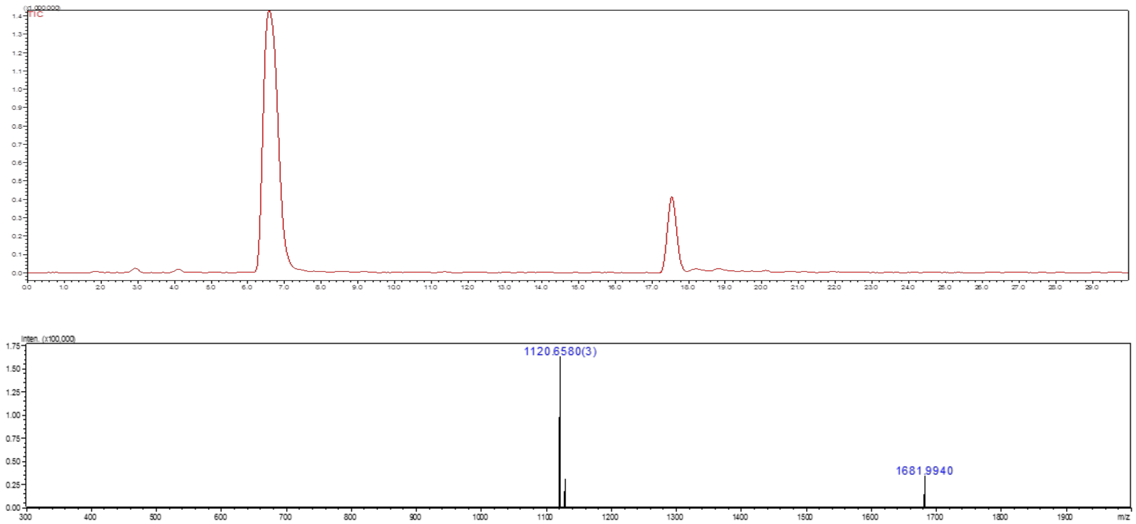

Figure S46. Liquid chromatography (LC) trace and mass (ESI-MS) spectrum (peak at retention time = 17.6 min) of **40**.

Compound P

The amine of *N*-glycan compound **40** was subjected for the hydrogenation using a general protocol **3k** to obtain **P** as a white solid.

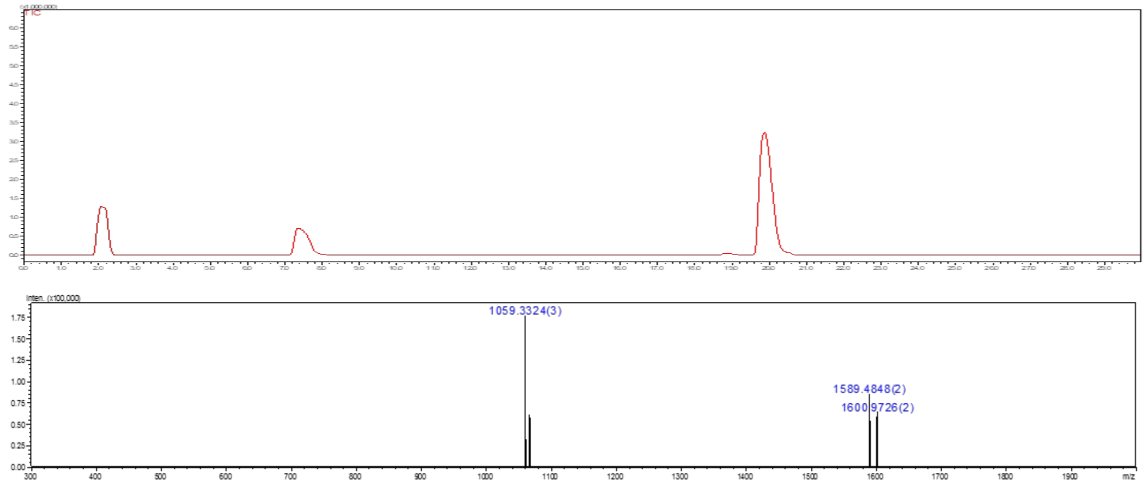

Figure S47. Liquid chromatography (LC) trace and mass (ESI-MS) spectrum (peak at retention time = 20.0 min) of **P**.

Compound 41

41 was synthesized from **39** (2.5 mg, 0.81 μmol) following the general protocol **3f** for the installation of Gal moiety using B4GalT1. The product **41** was obtained as a white fluffy solid (2.14 mg, 82%).

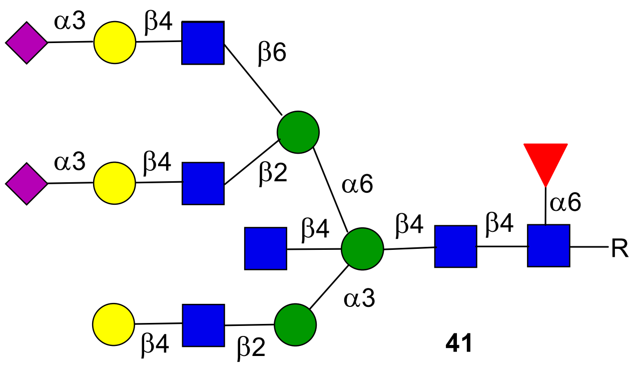

41

^{1}H NMR (900 MHz, D_2O): δ (ppm)

	H1	H2	H3	H4	H5	H6	NHAc
GlcNAc**1**	4.85	3.70	3.46	3.39	N/R[a]	N/R	2.03-1.79 (18H)
GlcNAc**2**	4.55	3.69	3.58	N/R	N/R	N/R	2.03-1.79 (18H)
GlcNAc**3**	4.52	3.71	3.54	3.43	N/R	N/R	2.03-1.79 (18H)
GlcNAc**4**	4.42	3.62	3.29	3.20	3.54	N/R	2.03-1.79 (18H)
GlcNAc**5**	4.52	3.61	3.55	3.48	N/R	N/R	2.03-1.79 (18H)
GlcNAc**6**	4.46	3.72	3.66	3.53	N/R	N/R	2.03-1.79 (18H)
Man**1**	4.61	4.09	3.78	3.44	N/R	N/R	-
Man**2**	4.97	4.17	3.83	3.39	3.54	N/R	-
Man**3**	4.82	4.03	3.71	3.34	N/R	N/R	-
Fuc**1**	4.74	3.66	3.71	3.61	3.93	1.06	-
Gal**1**	4.38	3.46	3.58	3.84	N/R	N/R	-
Gal**2**	4.47	3.50	4.05	3.88	N/R	N/R	-
Gal**3**	4.48	3.51	4.05	3.88	N/R	N/R	-

	H1	H2	H3	H4	H5	H6	H7	H8	H9
Neu5Ac9-**1**	-	-	2.68<br>1.72	3.61	3.77	N/R	N/R	N/R	N/R
Neu5Ac9-**2**	-	-	2.68<br>1.72	3.61	3.77	N/R	N/R	N/R	N/R

Signal	Proton	Carbon
**Aromatic**	7.91-7.84 (m, 4H)<br>7.52-7.47 (m, 3H)	128.3-125.6
**CH_2**Ph	5.33 (d, *J* = 12.8 Hz)<br>5.14 (d, *J* = 12.7 Hz)	66.8
NH-**CH**-COOH	4.32 (dd, *J* = 9.5, 3.6 Hz)	52.8
C(O)-**CH_2**-CH	2.76 (dd, *J* = 14.8, 2.9 Hz)<br>2.53 (dd, *J* = 15.0, 9.9 Hz)	38.7

^{13}C NMR (225 MHz, D_2O): δ (ppm)

	**C1**
GlcNAc**1**	78.2
GlcNAc**2**	101.1
GlcNAc**3**	99.6
GlcNAc**4**	99.7
GlcNAc**5**	99.6
GlcNAc**6**	101.7
Man**1**	99.8
Man**2**	99.8
Man**3**	97.8
Fuc**1**	99.2
Gal**1**	102.8
Gal**2**	102.6
Gal**3**	102.6

[a] Not reported

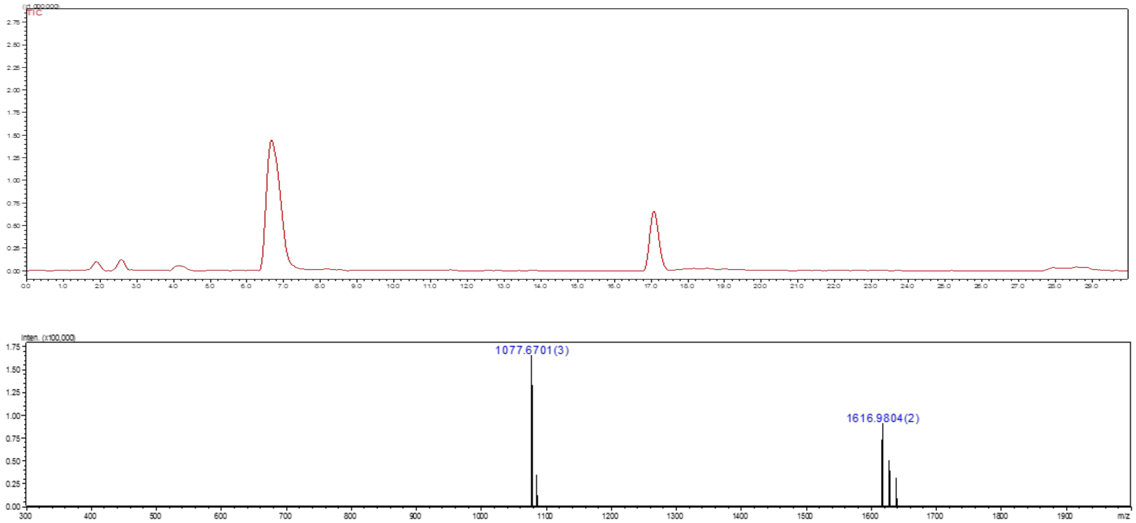

Figure S48. Liquid chromatography (LC) trace and mass (ESI-MS) spectrum (peak at retention time = 17.1 min) of **41**.

Compound O

The amine of *N*-glycan compound **41** was subjected for the hydrogenation using a general protocol **3k** to obtain **O** as a white solid.

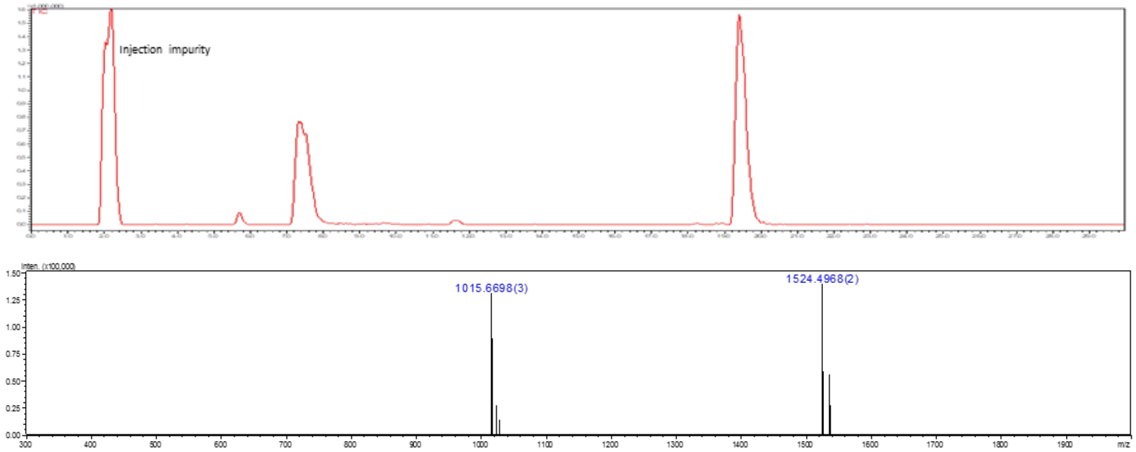

Figure S49. Liquid chromatography (LC) trace and mass (ESI-MS) spectrum (peak at retention time = 19.5 min) of **O**.

Compound 9

9 was synthesized from **40** (5 mg, 1.48 µmol) following the general protocol **3f** for the installation of Gal moiety using B4GalT1. The product **9** was obtained as a white fluffy solid (3.2 mg, 61%).

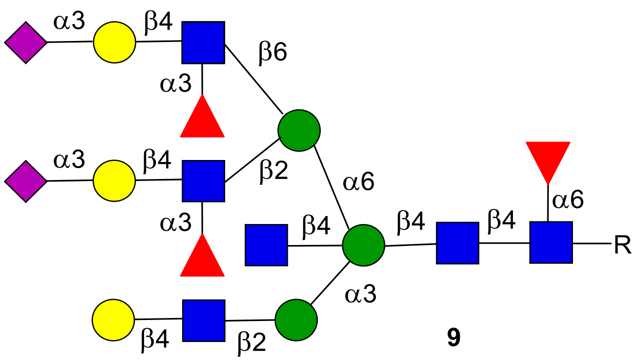

9

^{1}H NMR (600 MHz, D_2O) δ (ppm)

	H1	H2	H3	H4	H5	H6	NHAc
GlcNAc**1**	4.86	3.69	3.46	3.38	N/R[a]	N/R	2.03-1.95 (18H)
GlcNAc**2**	4.53	3.72	3.48	N/R	N/R	N/R	2.03-1.95 (18H)
GlcNAc**3**	4.57	3.64	3.53	N/R	N/R	N/R	2.03-1.95 (18H)
GlcNAc**4**	4.45	3.63	3.35	3.21	3.58	N/R	2.03-1.95 (18H)
GlcNAc**5**	4.49	3.64	3.53	N/R	N/R	N/R	2.03-1.95 (18H)
GlcNAc**6**	4.46	3.85	3.63	N/R	N/R	N/R	2.03-1.95 (18H)
Man**1**	4.62	4.09	3.74	N/R	N/R	N/R	-
Man**2**	4.96	4.16	3.85	3.35	N/R	N/R	-
Man**3**	4.83	4.06	3.74	N/R	N/R	N/R	-
Fuc**1**	4.72	3.67	3.70	3.62	3.94	1.08	-
Fuc**2**	5.04	3.63	3.72	3.61	4.75	1.10	-
Fuc**3**	5.09	3.63	3.72	3.61	4.75	1.10	-
Gal**1**	4.38	3.47	N/R	N/R	N/R	N/R	-
Gal**2**	4.45	3.44	4.01	3.86	N/R	N/R	-
Gal**3**	4.45	3.44	4.01	3.86	N/R	N/R	-

	H1	H2	H3	H4	H5	H6	H7	H8	H9
Neu5Ac9-**1**	-	-	2.68<br>1.72	3.60	3.72	-	-	-	-
Neu5Ac9-**2**	-	-	2.68<br>1.72	3.60	3.72	-	-	-	-

Signal	Proton	Carbon
**Aromatic**	7.92-7.86 (m, 4H)<br>7.53-7.48 (m, 3H)	128.4-125.8
**CH_2**Ph	5.33 (d, J = 12.8 Hz, 1H)<br>5.14 (d, J = 12.8 Hz, 1H)	66.8
NH-**CH**-COOH	4.29 (dd, J = 9.5, 3.6 Hz 1H)	52.8
C(O)-**CH**-COOH	2.75 (dd, J = 14.8, 2.9, 1H)<br>2.51 (dd, J = 15.0, 9.9, 1H)	38.7

[a] Not reported

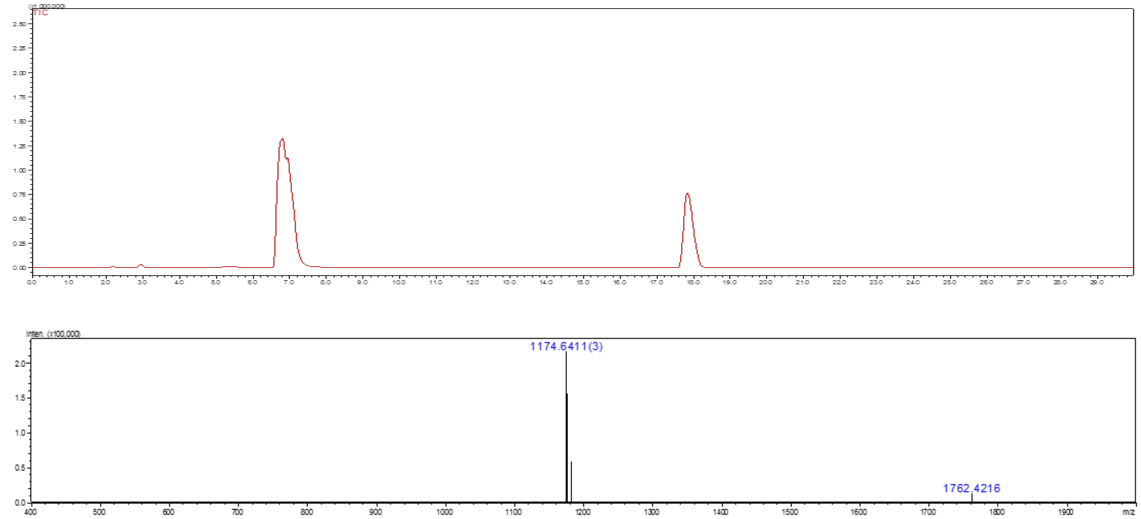

Figure S50. Liquid chromatography (LC) trace and mass (ESI-MS) spectrum (peak at retention time = 17.9 min) of **9**.

Compound Q

The amine of *N*-glycan compound **9** was subjected for the hydrogenation using a general protocol **3k** to obtain **Q** as a white solid.

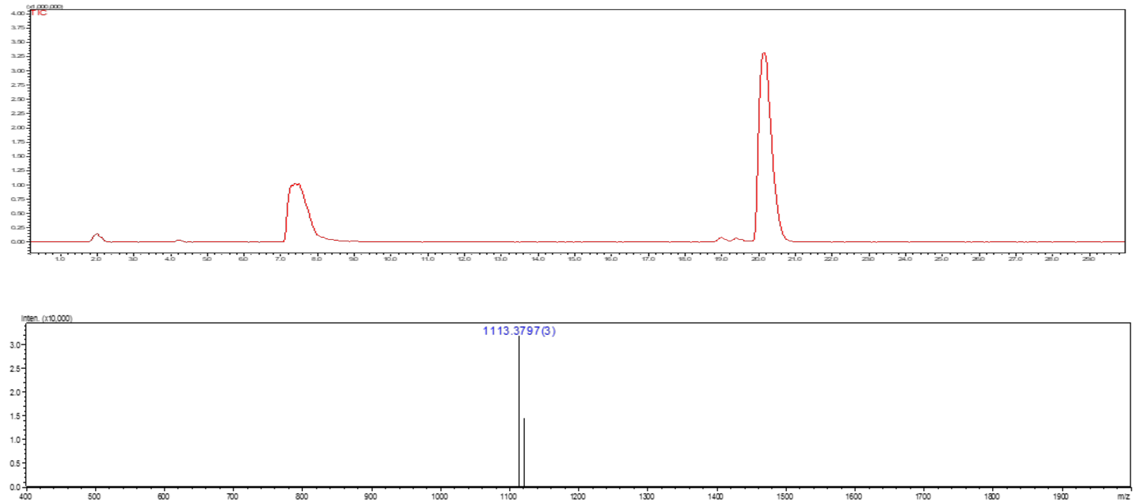

Figure S51. Liquid chromatography (LC) trace and mass (ESI-MS) spectrum (peak at retention time = 20.3 min) of **Q**.

Compound S11

N-Glycan **3** (10 mg, 6.41 µmol) was dissolved in deionized water with the final reaction concentration of 5 mM. The pH of the reaction solution was adjusted to 10 using NaOH (1 M), and the mixture solution was incubated for 3-5 h at 37 °C with shaking. After complete conversion of starting material to amine intermediate reaction was neutralized by aqueous acetic acid (1 M). The product was purified by C18 biogel [Eluent - water and water:acetonitrile (8:2)]. Next, resulting compound was subjected for the installation of a β1,4 Gal using the general procedure **3f** followed by acetylation using a general protocol **3j** to obtain **S9** as a white solid (8.8 mg, 78%). **S9** (6.1 mg, 3.4 µmol) was subjected to installation of 2, 3-Sialic acid following the general protocol **3g** for the installation of Neu5Ac moiety using ST3Gal4 to get product **S10** as a white fluffy solid. **S11** was synthesized from **S10** following the general protocol **3h** for the installation of Fuc moiety using FUT6. The product **S11** was obtained as a white fluffy solid (5.9 mg, 72%).

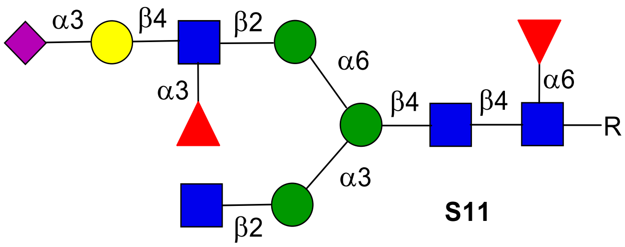

S11

^{1}**H NMR (600 MHz, D$_2$O) δ (ppm)**

	**H1**	**H2**	**H3**	**H4**	**H5**	**H6**	**NHAc**
GlcNAc**1**	4.86	3.69	3.44	3.37	N/R[a]	N/R	1.98-1.79 (12H)
GlcNAc**2**	4.54	3.70	3.51	3.65	N/R	N/R	1.98-1.79 (12H)
GlcNAc**3**	4.50	3.84	N/R	N/R	N/R	N/R	1.98-1.79 (12H)
GlcNAc**4**	4.47	3.63	3.47	3.37	N/R	N/R	1.98-1.79 (12H)
Man**1**	4.70	3.79	N/R	N/R	N/R	N/R	-
Man**2**	5.03	4.11	3.82	3.42	N/R	N/R	-
Man**3**	4.83	4.02	3.86	3.41	N/R	N/R	-
Fuc**1**	4.72	3.68	3.70	3.60	3.94	1.05	-
Gal**1**	4.44	3.45	4.01	3.85	N/R	N/R	-
Fuc**2**	5.05	3.60	3.83	3.71	3.74	1.10	-

	**H1**	**H2**	**H3**	**H4**	**H5**	**H6**	**H7**	**H8**	**H9**
Neu5Ac9	-	-	2.68<br>1.72	3.60	3.72	-	-	-	-

**Signal**	**Proton**	**Carbon**
**Aromatic**	7.91-7.84 (m, 4H)<br>7.52-7.47 (m, 3H)	128.3-125.6
**CH$_2$**Ph	5.33 (d, J = 12.8 Hz)<br>5.14 (d, J = 12.7 Hz)	66.8
NH-**CH**-COOH	4.32 (dd, J = 9.5, 3.6 Hz)	52.8

C(O)-**CH**-COOH	2.76 (dd, J = 14.8, 2.9 Hz)<br>2.53 (dd, J = 15.0, 9.9 Hz)	38.7

	**C1**
GlcNAc**1**	78.3
GlcNAc**2**	100.8
GlcNAc**3**	99.2
GlcNAc**4**	99.6
Man**1**	100.5
Man**2**	99.5
Man**3**	96.9
Fuc**1**	99.2
Gal**1**	101.4
Fuc**2**	98.4

[a] Not reported

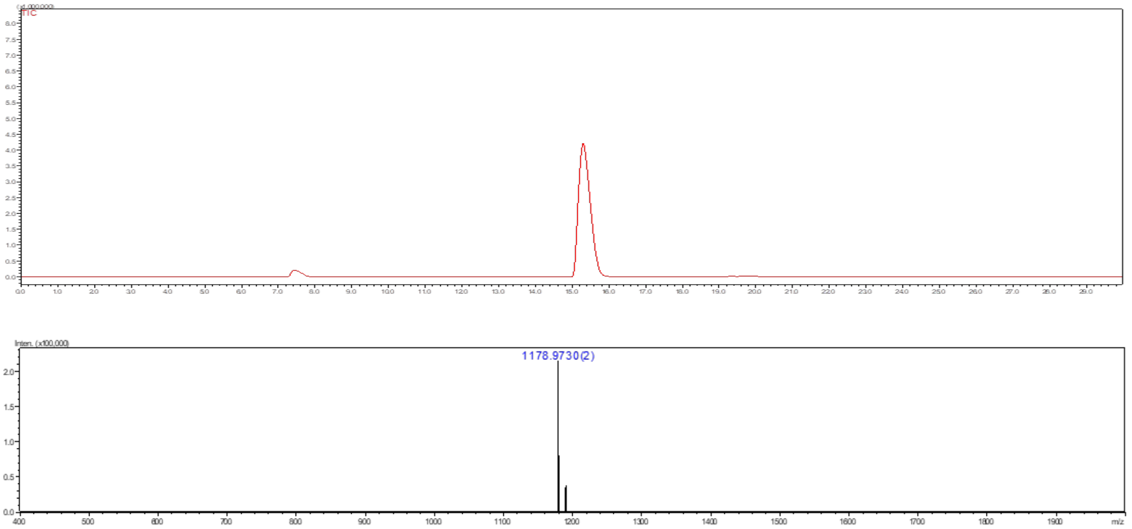

Figure S52. Liquid chromatography (LC) trace and mass (ESI-MS) spectrum (peak at retention time = 15.4 min) of **S11**.

Compound E′

The amine of *N*-glycan compound **S11** was subjected for the hydrogenation using a general protocol **3k** to obtain **E′** as a white solid.

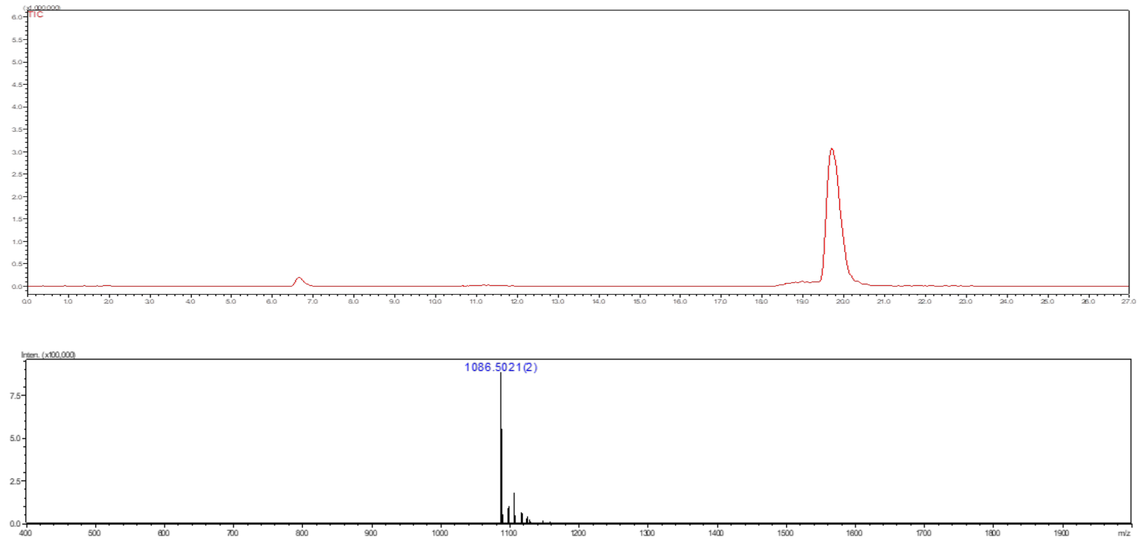

Figure S53. Liquid chromatography (LC) trace and mass (ESI-MS) spectrum (peak at retention time = 19.8 min) of **E′**.

Compound S12

S12 was synthesized from **S11** (3 mg, 1.27 μmol) following the general protocol **3f** for the installation of Gal moiety using B4GalT1. The product **S12** was obtained as a white fluffy solid (2.8 mg, 87%).

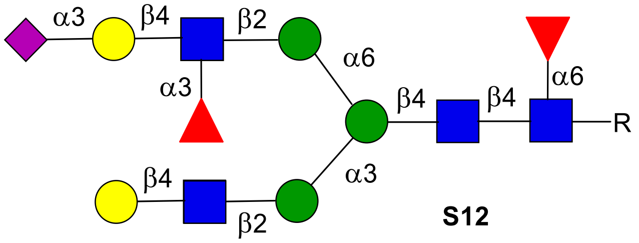

S12

^{1}H NMR (600 MHz, D_2O) δ (ppm)

	**H1**	**H2**	**H3**	**H4**	**H5**	**H6**	**NHAc**
GlcNAc**1**	4.85	3.70	3.61	N/R[a]	N/R	N/R	1.98-1.79 (12H)
GlcNAc**2**	4.54	3.70	3.65	N/R	N/R	N/R	1.98-1.79 (12H)
GlcNAc**3**	4.50	3.66	3.67	N/R	N/R	N/R	1.98-1.79 (12H)
GlcNAc**4**	4.50	3.66	3.67	N/R	N/R	N/R	1.98-1.79 (12H)
Man**1**	4.69	4.17	3.68	3.85	N/R	N/R	-
Man**2**	5.04	4.11	3.82	3.70	N/R	N/R	-
Man**3**	4.83	4.02	3.81	N/R	N/R	N/R	-
Fuc**1**	4.72	3.67	3.71	3.60	3.93	1.05	-
Fuc**2**	5.05	3.60	3.70	3.62	4.75	1.09	-
Gal**1**	4.39	3.46	3.86	N/R	N/R	N/R	-
Gal**2**	4.44	3.45	4.01	3.86	N/R	N/R	-

	**H1**	**H2**	**H3**	**H4**	**H5**	**H6**	**H7**	**H8**	**H9**
Neu5Ac9	**-**	**-**	2.69<br>1.72	3.60	3.77	**-**	**-**	**-**	**-**

**Signal**	**Proton**	
**Aromatic**	7.92-7.85 (m, 4H)<br>7.53-7.48 (m, 3H)	128.4-125.8
**CH_2**Ph	5.33 (d, *J* = 12.8 Hz, 1H)<br>5.14 (d, *J* = 12.8 Hz, 1H)	66.8
NH-**CH**-COOH	4.29 (dd, *J* = 9.5, 3.6 Hz 1H)	52.8
C(O)-**CH**-COOH	2.75 (dd, *J* = 14.8, 2.9, 1H)<br>2.51 (dd, *J* = 15.0, 9.9, 1H)	39.8

	**C1**
GlcNAc**1**	78.2
GlcNAc**2**	100.9
GlcNAc**3**	99.4
GlcNAc**4**	99.4
Man**1**	100.5
Man**2**	99.6
Man**3**	96.8
Fuc**1**	99.3
Fuc**2**	98.5
Gal**1**	103.0
Gal2	101.7

[a] Not reported

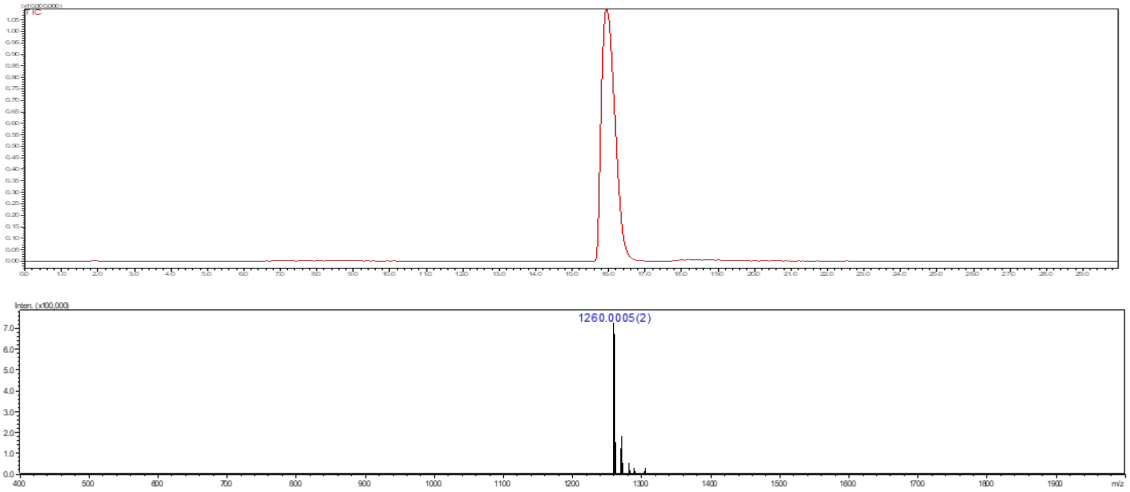

Figure S54. Liquid chromatography (LC) trace and mass (ESI-MS) spectrum (peak at retention time = 16.1 min) of **S12**.

Compound F′

The amine of *N*-glycan compound **S12** was subjected for the hydrogenation using a general protocol **3k** to obtain **F′** as a white solid.

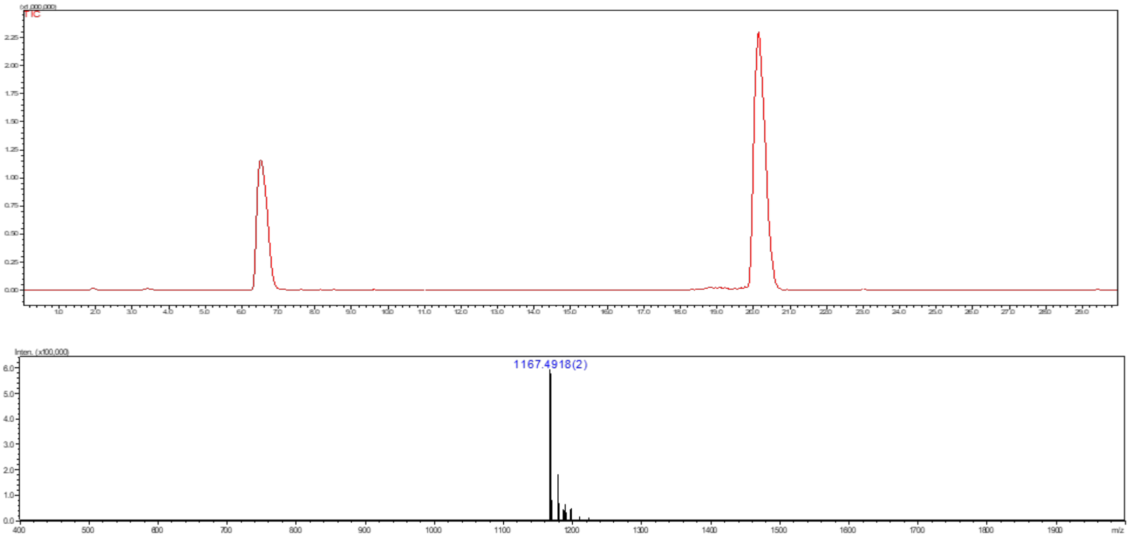

Figure S55. Liquid chromatography (LC) trace and mass (ESI-MS) spectrum (peak at retention time = 20.2 min) of **F′**.

Compound S15

N-Glycan **2** (12 mg, 10.90 μmol) was dissolved in deionized water with the final reaction concentration of 5 mM. The pH of the reaction solution was adjusted to 10 using NaOH (1 M), and the mixture solution was incubated for 3-5 h at 37 °C with shaking. The complete conversion of starting material to amine intermediate was confirmed by LC-MS. Then, reaction was neutralized by aqueous acetic acid (1 M). The product was purified by C18 biogel [Eluent - water and water:acetonitrile (8:2)]. Next, resulting compound was subjected for the installation of a β1,4 Gal using the general procedure **3f** to obtained followed by acetylation using a general protocol **3j** to obtain **S13** as a white solid (10.5 mg, 77%).

S13 (6 mg, 3.2 μmol) was subjected to installation of 2, 3-Sialic acid following the general protocol **3g** for the installation of Neu5Ac moiety using ST3Gal4 to get product **S14** as a white fluffy solid.

S15 was synthesized from **S14** following the general protocol **3h** for the installation of Fuc moiety using FUT6. The product **S15** was obtained as a white fluffy solid (6.2 mg, 78%).

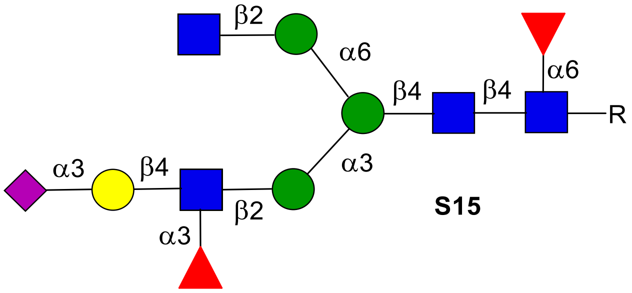

S15

^{1}H NMR (600 MHz, D_2O) δ (ppm)

	**H1**	**H2**	**H3**	**H4**	**H5**	**H6**	**NHAc**
GlcNAc**1**	4.86	3.69	3.60	3.45	N/R[a]	N/R	1.97-1.79 (12H)
GlcNAc**2**	4.54	3.70	3.64	N/R	N/R	N/R	1.97-1.79 (12H)
GlcNAc**3**	4.50	3.63	3.48	3.37	N/R	N/R	1.97-1.79 (12H)
GlcNAc**4**	4.48	3.63	3.48	3.37	N/R	N/R	1.97-1.79 (12H)
Man**1**	4.69	4.18	3.70	N/R	N/R	N/R	-
Man**2**	5.03	4.12	3.82	3.67	3.40	N/R	-
Man**3**	4.84	4.04	3.81	3.55	N/R	N/R	-
Fuc**1**	4.72	3.66	3.72	3.60	3.95	1.06	-
Gal**1**	4.43	3.44	4.01	3.86	N/R	N/R	-
Fuc**2**	5.04	3.61	3.83	3.70	4.74	1.09	-

	**H1**	**H2**	**H3**	**H4**	**H5**	**H6**	**H7**	**H8**	**H9**
Neu5Ac9	-	-	2.69<br>1.72	3.60	3.77	-	-	-	-

**Signal**	**Proton**	
**Aromatic**	7.92-7.85 (m, 4H)<br>7.53-7.48 (m, 3H)	128.4-125.8
**CH_2Ph**	5.33 (d, J = 12.8 Hz, 1H)	66.8

	5.14 (d, J = 12.8 Hz, 1H)	
NH-**CH**-COOH	4.29 (dd, J = 9.5, 3.6 Hz 1H)	52.8
C(O)-**CH**-COOH	2.75 (dd, J = 14.8, 2.9, 1H)<br>2.51 (dd, J = 15.0, 9.9, 1H)	39.8

	**C1**
GlcNAc**1**	78.2
GlcNAc**2**	100.7
GlcNAc**3**	99.2
GlcNAc**4**	99.5
Man**1**	100.4
Man**2**	99.5
Man**3**	97.0
Fuc**1**	99.1
Gal**1**	101.6
Fuc**2**	98.5

[a] Not reported

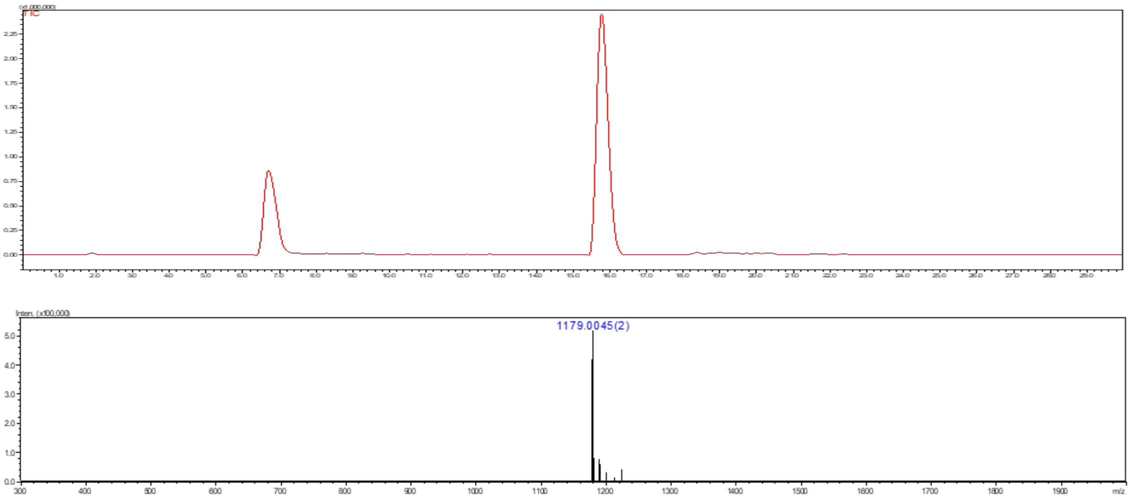

Figure S56. Liquid chromatography (LC) trace and mass (ESI-MS) spectrum (peak at retention time = 15.9 min) of **S15**.

Compound K′

The amine of *N*-glycan compound **S15** was subjected for the hydrogenation using a general protocol **3k** to obtain **K′** as a white solid.

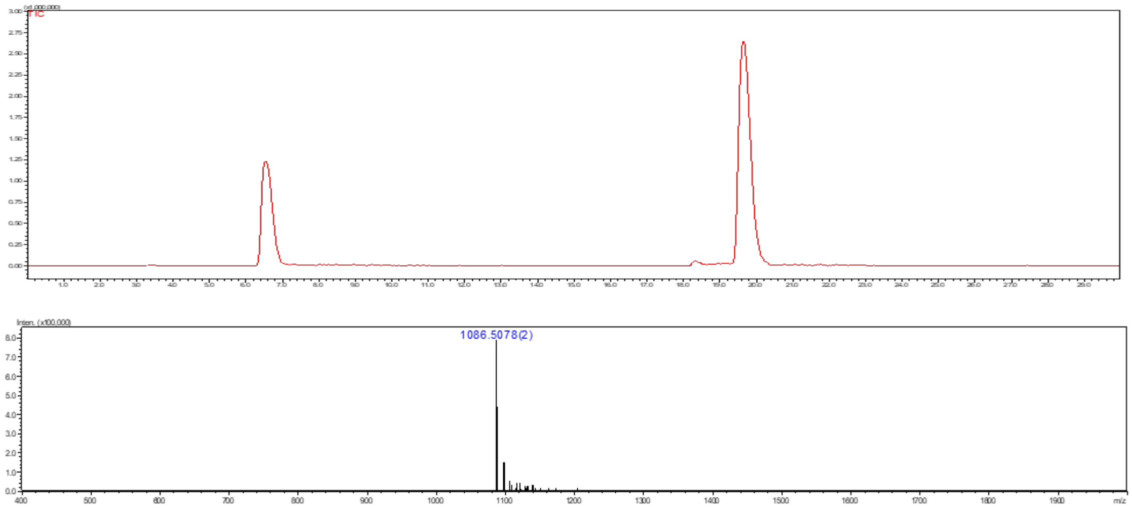

Figure S57. Liquid chromatography (LC) trace and mass (ESI-MS) spectrum (peak at retention time = 19.9 min) of **K′**.

Compound S16

S16 was synthesized from **S15** (2.5 mg, 0.81 μmol) following the general protocol **3f** for the installation of Gal moiety using B4GalT1. The product **S16** was obtained as a white fluffy solid (2.14 mg, 80%).

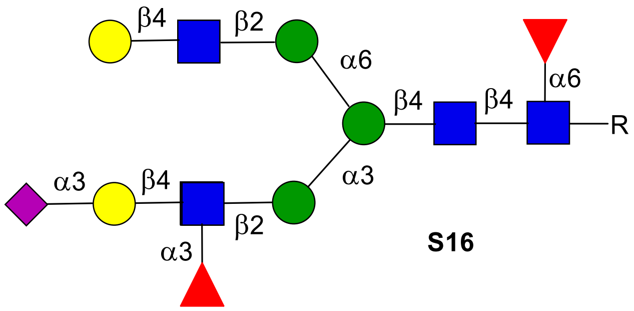

S16

^{1}H NMR (600 MHz, D_2O) δ (ppm)

	H1	H2	H3	H4	H5	H6	NHAc
GlcNAc**1**	4.86	3.70	3.60	N/R[a]	N/R	N/R	1.98-3.80 (12H)
GlcNAc**2**	4.55	3.71	3.65	3.65	3.49	N/R	1.98-3.80 (12H)
GlcNAc**3**	4.50	3.84	N/R	N/R	N/R	N/R	1.98-3.80 (12H)
GlcNAc**4**	4.50	3.68	3.60	3.49	N/R	N/R	1.98-3.80 (12H)
Man**1**	4.69	4.17	3.70	3.50	N/R	N/R	-
Man**2**	5.03	4.11	3.82	3.66	N/R	N/R	-
Man**3**	4.85	4.02	3.83	3.54	N/R	N/R	-
Fuc**1**	4.72	3.67	3.72	3.60	3.94	1.07	-
Fuc**2**	5.04	3.60	3.83	3.67	4.75	1.10	-
Gal**1**	4.43	3.45	4.00	3.85	N/R	N/R	-
Gal**2**	4.39	3.46	N/R	3.85	N/R	N/R	-

	H1	H2	H3	H4	H5	H6	H7	H8	H9
Neu5Ac9	-	-	2.69<br>1.72	3.60	3.77	-	-	-	-

Signal	Proton	Carbon
**Aromatic**	7.92-7.85 (m, 4H)<br>7.53-7.48 (m, 3H)	128.4-<br>125.8
**CH_2**Ph	5.33 (d, *J* = 12.8 Hz, 1H)<br>5.14 (d, *J* = 12.8 Hz, 1H)	66.8
NH-**CH**-COOH	4.29 (dd, *J* = 9.5, 3.6 Hz 1H)	52.8
C(O)-**CH**-COOH	2.75 (dd, *J* = 14.8, 2.9, 1H)<br>2.51 (dd, *J* = 15.0, 9.9, 1H)	39.8

	**13C**
GlcNAc**1**	78.0
GlcNAc**2**	100.8
GlcNAc**3**	99.1
GlcNAc**4**	99.1
Man**1**	100.3
Man**2**	99.5
Man**3**	96.9
Fuc**1**	99.4
Fuc**2**	98.5
Gal**1**	101.7
Gal**2**	103.1

[a] Not reported

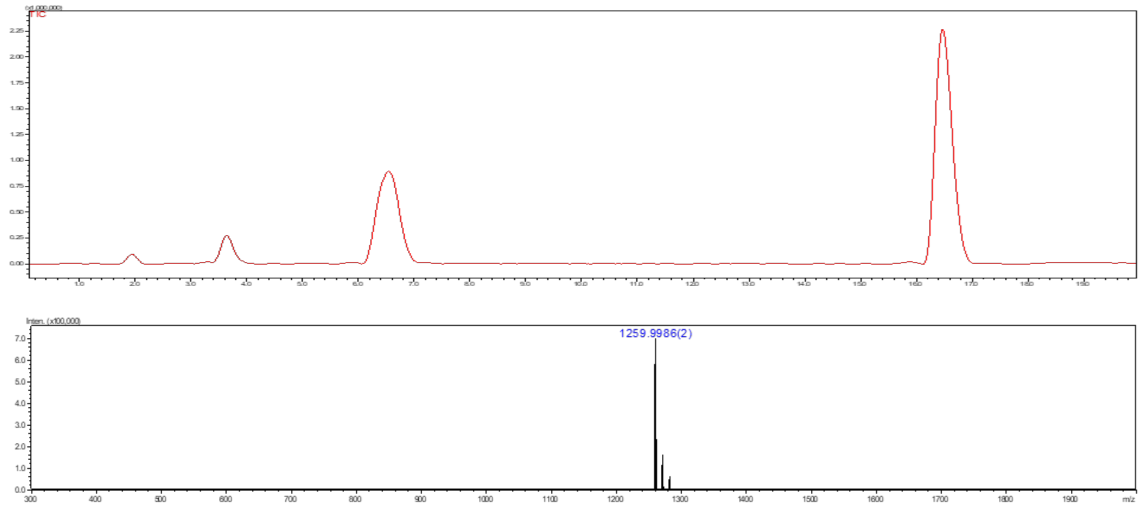

Figure S58. Liquid chromatography (LC) trace and mass (ESI-MS) spectrum (peak at retention time = 16.6 min) of **S16**.

Compound L′

The amine of *N*-glycan compound **47** was subjected for the hydrogenation using a general protocol **3k** to obtain **L′** as a white solid.

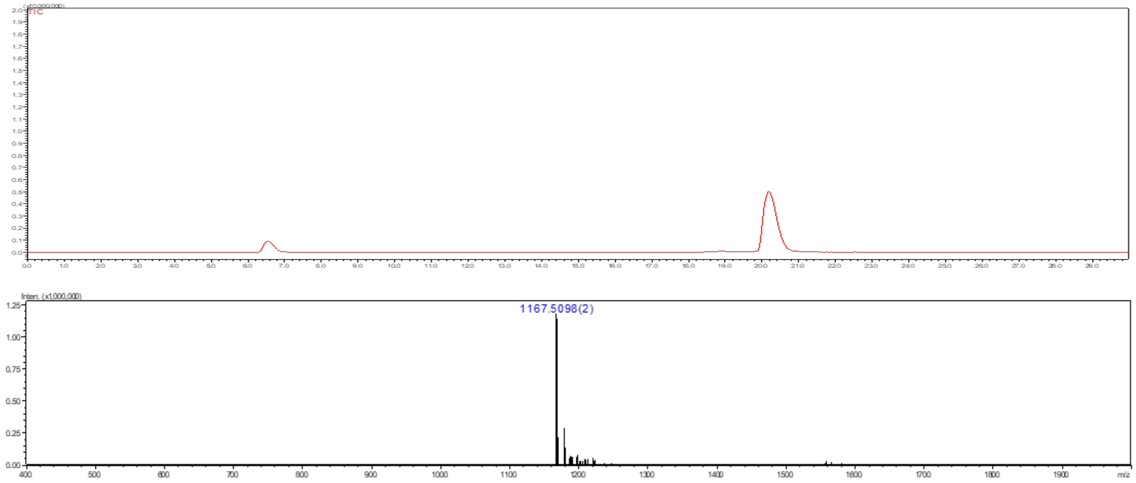

Figure S59. Liquid chromatography (LC) trace and mass (ESI-MS) spectrum (peak at retention time = 20.4 min) of **L′**.

Compound S19

S19 was synthesized from **S3** (5 mg, 0.81 μmol) following the general protocol **3c** for the installation of GlcNAc moiety using UDP-GlcNAc. The product **S19** was obtained as a white fluffy solid (4.7 mg, 84%).

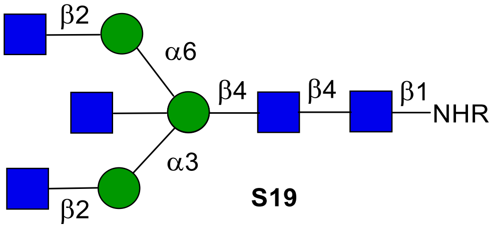

S19

^{1}H NMR (600 MHz, D_2O): δ (ppm)

	H1	H2	H3	H4	H5	H6	NHAc
GlcNAc**1**	4.83	3.68	3.47	3.44	N/R[a]	N/R	2.00-1.79 (15H)
GlcNAc**2**	4.47	3.72	3.40	3.65	N/R	N/R	2.00-1.79 (15H)
GlcNAc**3**	4.47	3.63	3.40	N/R	N/R	N/R	2.00-1.79 (15H)
GlcNAc**4**	4.39	3.62	3.48	3.20	3.33	N/R	2.00-1.79 (15H)
GlcNAc**5**	4.47	3.63	3.40	N/R	N/R	N/R	2.00-1.79 (15H)
Man**1**	4.61	4.09	3.78	4.00	3.46	N/R	**-**
Man**2**	4.98	4.17	3.82	3.47	N/R	N/R	**-**
Man**3**	4.92	4.06	3.75	3.40	N/R	N/R	**-**

[a] Not reported

Signal	Proton	Carbon
**Aromatic**	7.90-7.85 (m, 4H)<br>7.52-7.47 (m, 3H)	128.3-<br>125.8
**CH**$_2$Ph	5.30 (d, *J* = 12.8 Hz, 1H)<br>5.14 (d, *J* = 12.8 Hz, 1H)	66.9
NH-**CH**-COOH	4.32 (dd, *J* = 9.5, 3.6 Hz 1H)	52.9
C(O)-**CH**$_2$-CH	2.76 (dd, *J* = 14.8, 2.9, 1H)<br>2.50 (dd, *J* = 15.0, 9.9, 1H)	38.7

	13C
GlcNAc**1**	78.0
GlcNAc**2**	101.2
GlcNAc**3**	99.8
GlcNAc**4**	100.6
GlcNAc**5**	99.8
Man**1**	100.1
Man**2**	99.9
Man**3**	97.7

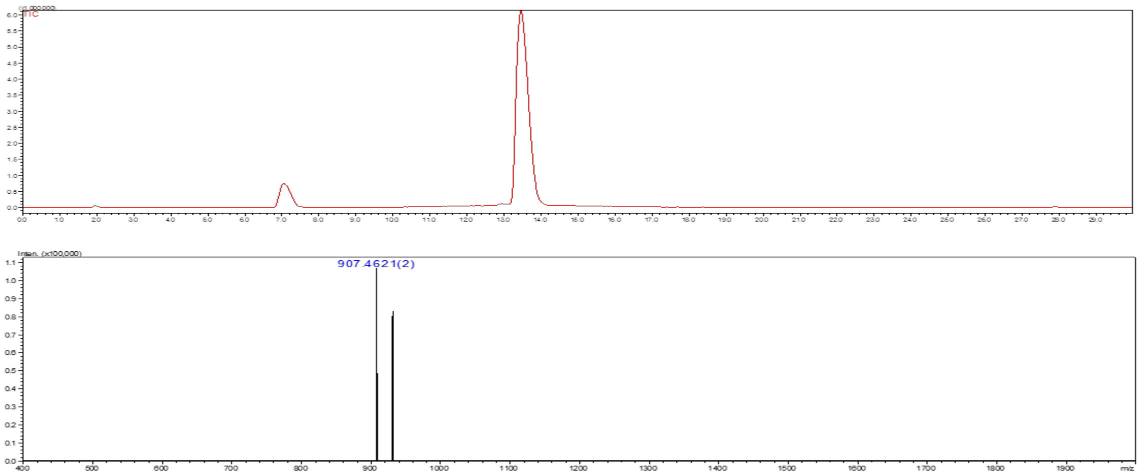

Figure S60. Liquid chromatography (LC) trace and mass (ESI-MS) spectrum (peak at retention time = 13.6 min) of **S19**.

Compound S20

S20 was synthesized from **3** (2 mg, 1.13 μmol) following the general protocol **3d** for the installation of GlcNAc moiety using UDP-GlcNAc. The product **S20** was obtained as a white fluffy solid (1.61 mg, 72%).

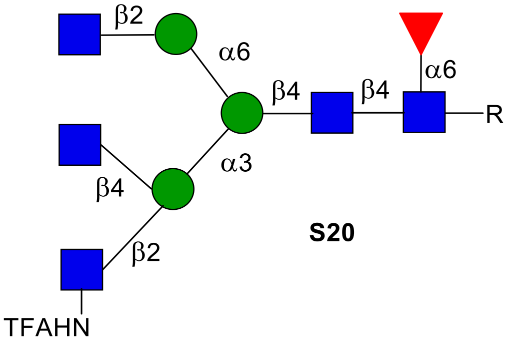

S20

^{1}H NMR (600 MHz, D_2O): δ (ppm)

	H1	H2	H3	H4	H5	H6	NHAc
GlcNAc**1**	4.84	3.69	3.45	3.37	N/R[a]	N/R	1.99-1.79 (12H)
GlcNAc**2**	4.53	3.69	3.50	3.63	N/R	N/R	1.99-1.79 (12H)
GlcNTFA	4.58	3.75	3.59	3.39	N/R	N/R	-
GlcNAc**3**	4.43	3.65	3.47	3.42	N/R	N/R	1.99-1.79 (12H)

GlcNAc**4**	4.46	3.64	3.46	3.40	N/R	N/R	1.99-1.79 (12H)
Man**1**	4.66	4.14	3.68	3.54	N/R	N/R	-
Man**2**	5.00	4.17	3.97	3.45	N/R	N/R	-
Man**3**	4.82	4.04	3.83	3.54	3.42	N/R	-
Fuc**1**	4.70	3.66	3.71	3.61	3.92	1.05	-

Signal	Proton	Carbon
**Aromatic**	7.91-7.84 (m, 4H)<br>7.52-7.47 (m, 3H)	128.3-<br>125.7
**CH$_2$**Ph	5.32 (d, *J* = 12.8 Hz, 1H)<br>5.13 (d, *J* = 12.8 Hz, 1H)	66.8
NH-**CH**-COOH	4.28 (dd, *J* = 9.5, 3.6 Hz 1H)	53.0
C(O)-**CH$_2$**-CH	2.75 (dd, *J* = 14.8, 2.9, 1H)<br>2.51 (dd, *J* = 15.0, 9.9, 1H)	38.5

[a] Not reported

	**13C**
GlcNAc**1**	77.8
GlcNAc**2**	100.9
GlcNTFA	98.73
GlcNAc**3**	101.67
GlcNAc**4**	99.53
Man**1**	100.38
Man**2**	98.85
Man**3**	96.95
Fuc**1**	99.28

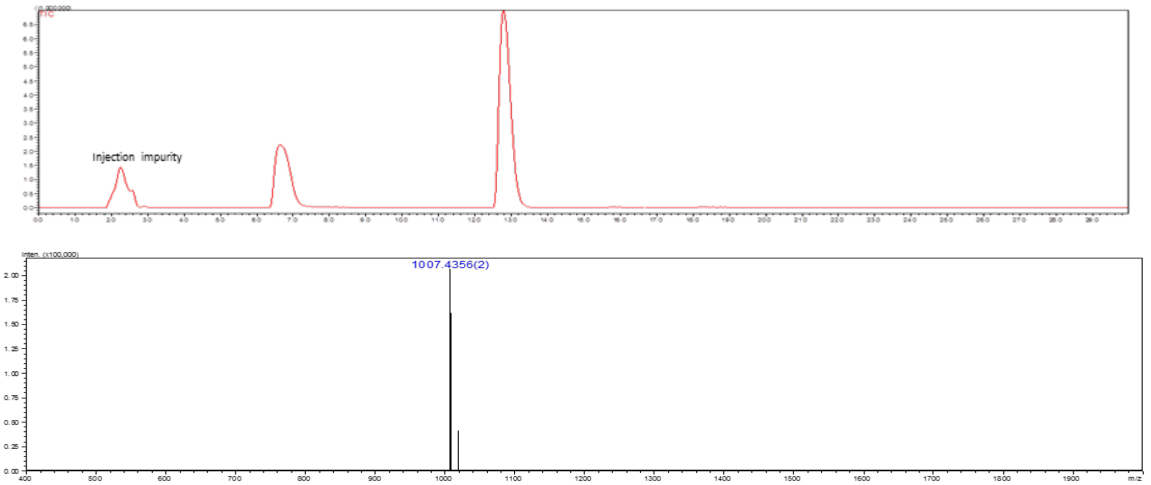

Figure S61. Liquid chromatography (LC) trace and mass (ESI-MS) spectrum (peak at retention time = 13.0 min) of **S20**.

Compound S21

S21 was synthesized from **2** (1.5 mg, 5.67 μmol) following the general protocol **3e** for the installation of GlcNAc moiety using UDP-GlcNAc. The product S**21** was obtained as a white fluffy solid (1.16 mg, 70%).

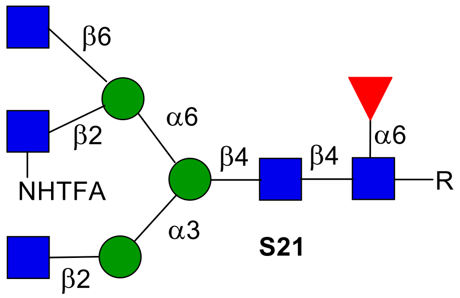

S21

^{1}H NMR (600 MHz, D_2O): δ (ppm)

	H1	H2	H3	H4	H5	H6	NHAc
GlcNAc**1**	4.86	3.70	3.40	3.44	N/R[a]	N/R	2.02-1.79 (12H)
GlcNAc**2**	4.55	3.71	3.51	3.65	N/R	N/R	2.02-1.79 (12H)
GlcNAc**3**	4.49	3.63	3.49	3.38	N/R	N/R	2.02-1.79 (12H)
GlcNTFA	4.60	3.72	3.60	3.41	3.39	N/R	-
GlcNAc**4**	4.40	3.65	3.49	3.38	N/R	N/R	2.02-1.79 (12H)

Man**1**	4.69	4.17	3.69	3.54	N/R	N/R	**-**
Man**2**	5.05	4.13	3.83	3.66	N/R	N/R	**-**
Man**3**	4.76	4.04	3.81	3.63	N/R	N/R	**-**
Fuc**1**	4.73	3.66	3.72	3.60	3.94	1.07	**-**

**Signal**	**Proton**	**Carbon**
**Aromatic**	7.91-7.85 (m, 4H)<br>7.53-7.48 (m, 3H)	128.7-125.7
**CH_2**Ph	5.33 (d, *J* = 12.8 Hz, 1H)<br>5.15 (d, *J* = 12.8 Hz, 1H)	66.9
NH-**CH**-COOH	4.29 (dd, *J* = 9.5, 3.6 Hz 1H)	53.1
C(O)-**CH_2**-CH	2.75 (dd, *J* = 14.8, 2.9, 1H)<br>2.52 (dd, *J* = 15.0, 9.9, 1H)	38.7

[a] Not reported

	**13C**
GlcNAc**1**	78.2
GlcNAc**2**	100.9
GlcNAc**3**	99.6
GlcNTFA	99.2
GlcNAc**4**	101.7
Man**1**	100.6
Man**2**	99.6
Man**3**	97.2
Fuc**1**	99.4

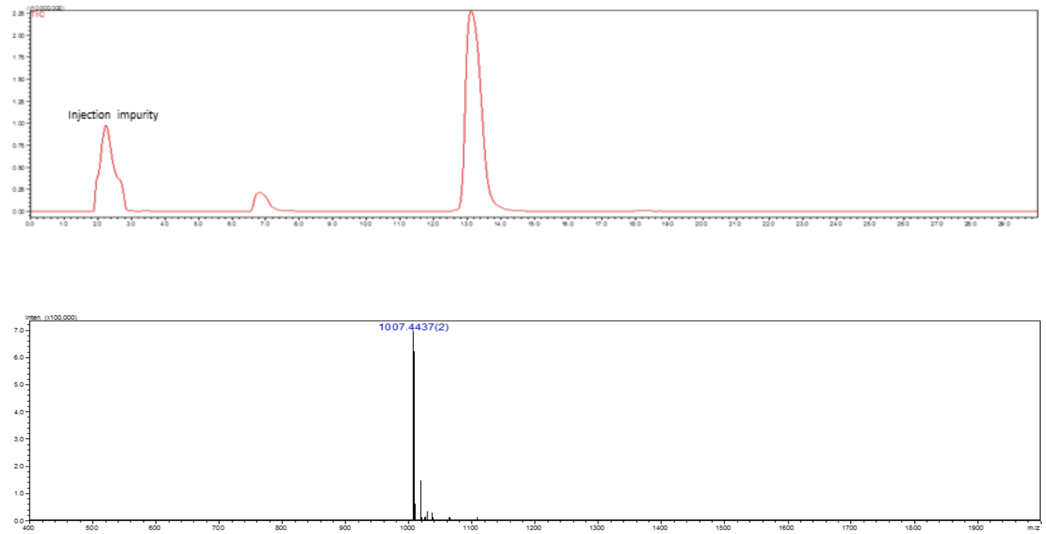

Figure S62. Liquid chromatography (LC) trace and mass (ESI-MS) spectrum (peak at retention time = 13.3 min) of **S21**.

9. Kinetic Parameters of GlcNAc Transfer GnT-III, GnT-IV and GnT-V for Multi-antennary Glycans

Following established protocol,[6] determination of GnT-III, GnT-IVB, and GnT-V steady state kinetic parameters for sugar donor and acceptor substrates was achieved in 5 µL reactions with 16 ng GnT-III, 50 ng GnT-IVB, and 35 ng GnT-V in Corning® Low Volume 384-well White Flat Bottom Polystyrene NBS Microplates (Catalog #3824). GnT-III and GnT-V were performed using a 50 mM MES, pH 6.5 buffer supplemented with 5 mM $MnCl_2$. GnT-IVB was performed using a 50 mM Tris-HCl, pH 7.5 supplemented with 5 mM $MnCl_2$. For substrate kinetics, acceptors were serially diluted from 2 to 0.03 mM or 10 to 0.16 mM for GnT-III/GnT-V and GnT-IVB respectively, with a constant donor concentration of 1, 7, and 10 mM for GnT-III, GnT-IVB, and GnT-V respectively. For GnT-III donor kinetics, UDP-GlcNAc was serially diluted from 6 mM to 0.09 mM with a constant acceptor concentration of 0.4 mM. Reactions were incubated for 1 h at 37 °C. UDP formation was detected using a UDP-Glo assay following the manufacturer's procedure (Catalog #V6971). Luminescence values were associated with a UDP standard curve for quantification of UDP generated and Michaelis-Menten kinetic K_{cat}, K_m, and V_{max} were calculated through nonlinear curve fitting in GraphPad Prism 6.

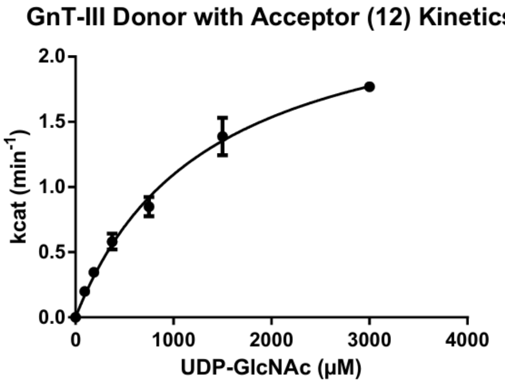

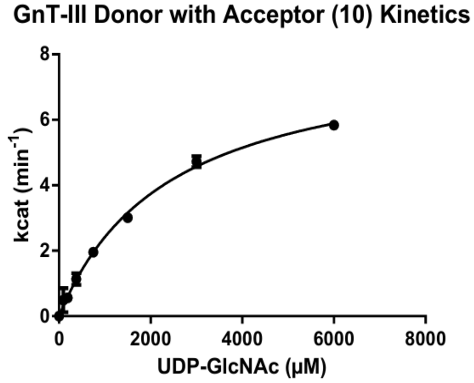

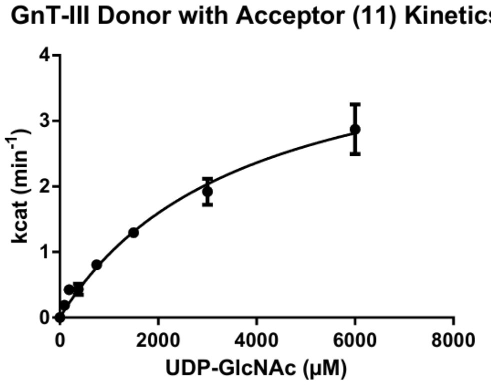

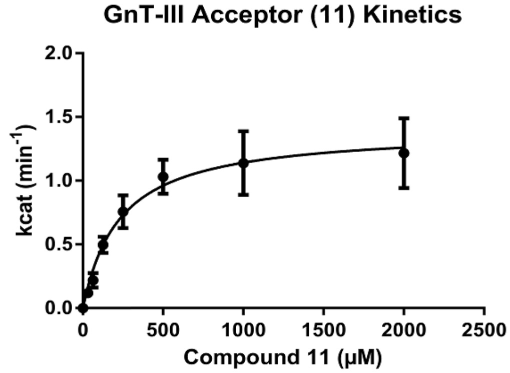

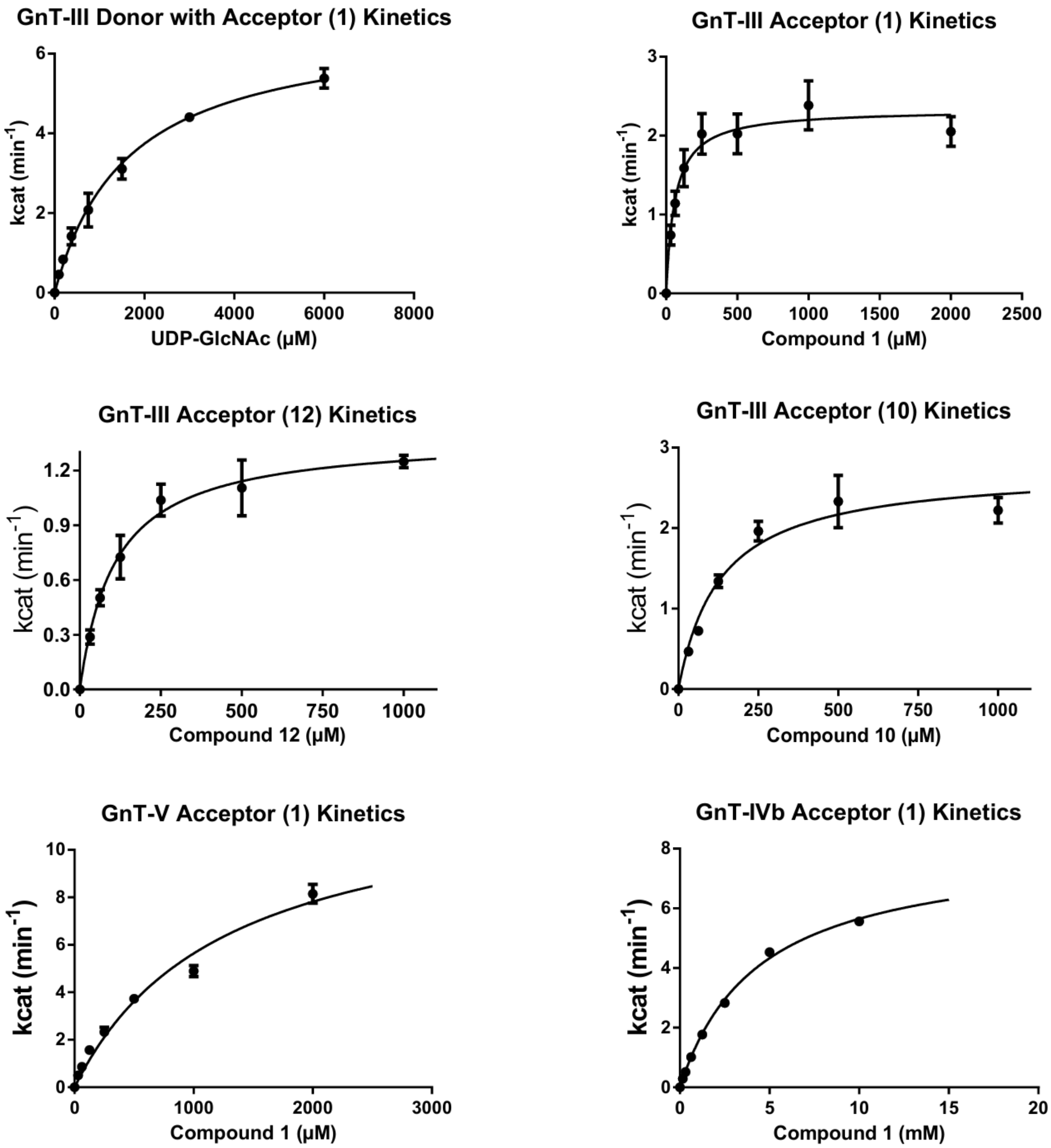

Figure S63. Steady state kinetics of GnT-III, GnT-IVB, and GnT-V as determined by UDP-Glo assay. Acceptors: **1**, **10**, **11**, **12**, **S17**, and **S18**. Donor: UDP-GlcNAc. All reactions performed in triplicate with kinetic parameters determined via nonlinear curve fitting in GraphPad Prism 6.

10. Glycan Microarray Development

The *N*-glycans containing free α-amine of Asn moiety were printed on NHS-ester activated glass slides (NEXTERION® Slide H, Schott Inc.) using a Scienion sciFLEXARRAYER S3 non-contact microarray equipped with a Scienion PDC80 nozzle (Scienion Inc.). All samples at a concentration of 100 μM in sodium phosphate buffer (0.225 M, pH 8.5) were printed in replicates of 6 with spot volume ~ 400 pL, at 20 °C and 50% relative humidity (each slide has 24 subarrays in a 3x8 layout). After printing, slides were incubated overnight in a saturated NaCl chamber (affording a 75% relative humidity environment), the remaining activated esters were then quenched with 5 mM ethanolamine in a Tris buffer (pH 9.0, 50 mM) at 50 °C for 1 h. Blocked slides were rinsed with DI water, spun dry, and kept in a desiccator at room temperature for future use. Printed glass slide was incubated with protein of interest. After 1 h, the slide was sequentially washed by dipping in TSM wash buffer (2 min, containing 0.05 % Tween 20), TSM buffer (2 min) and, water (2 x 2 min), spun dry. The slide was incubated with fluorescent detection antibody (for details see Tables S1 and S2, in case of anti-CD15s premixed primary antibody and secondary fluorescent antibody were employed) for 1 h and same washing sequence was repeated. The slides were scanned using a GenePix 4000B microarray scanner (Molecular Devices) at the appropriate excitation wavelength with a resolution of 5 μm. Various gains and PMT values were employed in the scanning to ensure that all the signals were within the linear range of the scanner's detector and there was no saturation of signals. The images were analyzed using GenePix Pro 7 software (Version 7.2.29.2, Molecular Devices). The data was analyzed with our home written Excel macro to provide the results. The highest and the lowest value of the total fluorescence intensity of the six replicates spots were removed, and the four values in the middle were used to provide the mean value and standard deviation. The data were fitted using Prism software 8 (GraphPad Software, Inc.), bar represents the mean ± SD for each compound. All experiments were performed three times at the minimum.

Table S1. List of protein, protein source (type of fusion tag, if present), primary antibodies and secondary antibodies (type of fluorophore, if present) screened against the *N*-glycans-microarray.

Sr. #	Protein	Source	Tag	Detection	Source
1	*Phaseolus vulgaris* E lectin (PHA-E)	Vector Labs Cat # B-1125-2	Biotin	Streptavidin-Alexa Fluor® 647	Thermo, Cat # S32364
2	*Concanavalin* A (ConA)	Vector Labs Cat # B-1005-5	Biotin	Streptavidin-Alexa Fluor® 647	Thermo, Cat # S32364
3	*Aleuria aurantia* lectin (AAL)	Vector Labs Cat # B-1395-1	Biotin	Streptavidin-Alexa Fluor® 647	Thermo, Cat # S32364
4	*Sambucus nigra* agglutinin (SNA)	Vector Labs Cat # B-1305-2	Biotin	Streptavidin-Alexa Fluor® 647	Thermo, Cat # S32364
5	*Maackia amurensis* lectin I (MAL-I)	Vector Labs Cat # B-1315-2	Biotin	Streptavidin-Alexa Fluor® 647	Thermo, Cat # S32364
6	*Wheat Germ* Agglutinin (WGA)	Vector Labs Cat # B-1025-5	Biotin	Streptavidin-Alexa Fluor® 647	Thermo, Cat # S32364
7	*Datura stramonium* lectin (DSL)	Vector Labs Cat # B-1185-2	Biotin	Streptavidin-Alexa Fluor® 647	Thermo, Cat # S32364
8	*Griffonia simplicifolia* lectin II (GSL-II)	Vector Labs Cat # B-1215-2	Biotin	Streptavidin-Alexa Fluor® 647	Thermo, Cat # S32364
9	*Wisteria floribunda* lectin (WFL)	Vector Labs	Biotin	Streptavidin-Alexa Fluor® 647	Thermo, Cat # S32364

		Cat # B-1355-2			
10	*Erythrina cristagalli* lectin (ECL)	Vector Labs Cat # B-1145-5	Biotin	Streptavidin-Alexa Fluor® 647	Thermo, Cat # S32364
11	Anti-CD15s antibody	BD Pharmigen Cat # 551344	untagged	Alexa Fluor® 647 anti-mouse IgG antibody	Jackson Immuno Res

Table S2. List of viruses, haemagglutinin anti-stem antibody, detecting antibody, and source screened against *N*-glycans-microarray.

Sr. #	Virus	Haemagglutinin anti-stem antibody	Detecting antibody	Source
A	A/California/07/2009 (pdmH1N1)	1A06[7]	Alexa Fluor® 647 anti-human IgG antibody	Jackson Immuno Res
B	A/Perth/16/2009 (H3N2)	CR8020	Alexa Fluor® 647 anti-human IgG antibody	Jackson Immuno Res
C	A/Vietnam/1203/2004 (H5N1)	1A06[7]	Alexa Fluor® 647 anti-human IgG antibody	Jackson Immuno Res
D	A/bovine/Ohio/B24OSU-432/2024 (H5N1)	1A06[7]	Alexa Fluor® 647 anti-human IgG antibody	Jackson Immuno Res

11. References

(1) Meng, L.; Forouhar, F.; Thieker, D.; Gao, Z.; Ramiah, A.; Moniz, H.; Xiang, Y.; Seetharaman, J.; Milaninia, S.; Su, M.; Bridger, R.; Veillon, L.; Azadi, P.; Kornhaber, G.; Wells, L.; Montelione, G. T.; Woods, R. J.; Tong, L.; Moremen, K. W. Enzymatic basis for *N*-glycan sialylation: structure of rat alpha2,6-sialyltransferase (ST6GAL1) reveals conserved and unique features for glycan sialylation. *J. Biol. Chem.* **2013**, *288*, 34680-34698.

(2) Kadirvelraj, R.; Yang, J. Y.; Sanders, J. H.; Liu, L.; Ramiah, A.; Prabhakar, P. K.; Boons, G. J.; Wood, Z. A.; Moremen, K. W. Human *N*-acetylglucosaminyltransferase II substrate recognition uses a modular architecture that includes a convergent exosite. *Proc. Natl. Acad. Sci. U. S. A.* **2018**, *115*, 4637-4642.

(3) Prudden, A. R.; Liu, L.; Capicciotti, C. J.; Wolfert, M. A.; Wang, S.; Gao, Z.; Meng, L.; Moremen, K. W.; Boons, G. J. Synthesis of asymmetrical multiantennary human milk oligosaccharides. *Proc. Natl. Acad. Sci. U. S. A.* **2017**, *114*, 6954-6959.

(4) Liu, L.; Prudden, A. R.; Capicciotti, C. J.; Bosman, G. P.; Yang, J. Y.; Chapla, D. G.; Moremen, K. W.; Boons, G. J. Streamlining the chemoenzymatic synthesis of complex *N*-glycans by a stop and go strategy. *Nat. Chem.* **2019**, *11*, 161-169.

(5) Liu, L.; Prudden, A. R.; Bosman, G. P.; Boons, G. J. Improved isolation and characterization procedure of sialylglycopeptide from egg yolk powder. *Carbohydr. Res*. **2017**, *452*, 122-128.

(6) Engel, L.; Alves, J.; Hennek, J.; Goueli, S.A.; Zegzouti, H. Utility of Bioluminescent Homogeneous Nucleotide Detection Assays in Measuring Activities of Nucleotide-Sugar Dependent Glycosyltransferases and Studying Their Inhibitors. *Molecules* **2021**, *26*, 6230.

(7) Ellebedy, A. H.; Nachbagauer, R.; Jackson, K. J. L.; Dai, Y.; Han, J.; Alsoussi, W. B.; Davis, C. W.; Stadlbauer, D.; Rouphael, N.; Chromikova, V.; McCausland, M.; Chang, C. Y.; Cortese, M.; Bower, M.; Chennareddy, C.; Schmitz, A. J.; Zarnitsyna, V. I.; Lai, L.; Rajabhathor, A.; Kazemian, C.; Antia R.; Mulligan M. J.; Ward, A. B.; Fremont, D. H.; Boyd, S. D.; Pulendran, B.; Krammer, F.; Ahmed, R. Adjuvanted H5N1 influenza vaccine enhances both cross-reactive memory B cell and strain-specific naive B cell responses in humans. *Proc. Natl. Acad. Sci. U. S. A.* **2020**, *117*, 17957-17964.

12. NMR Spectra

skb-251_A-Nap_P2.1.fid
skb-251_A-Nap_P2

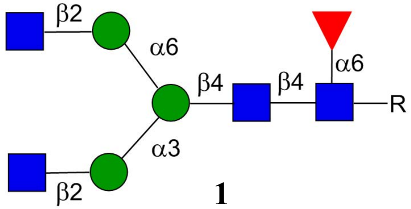

1

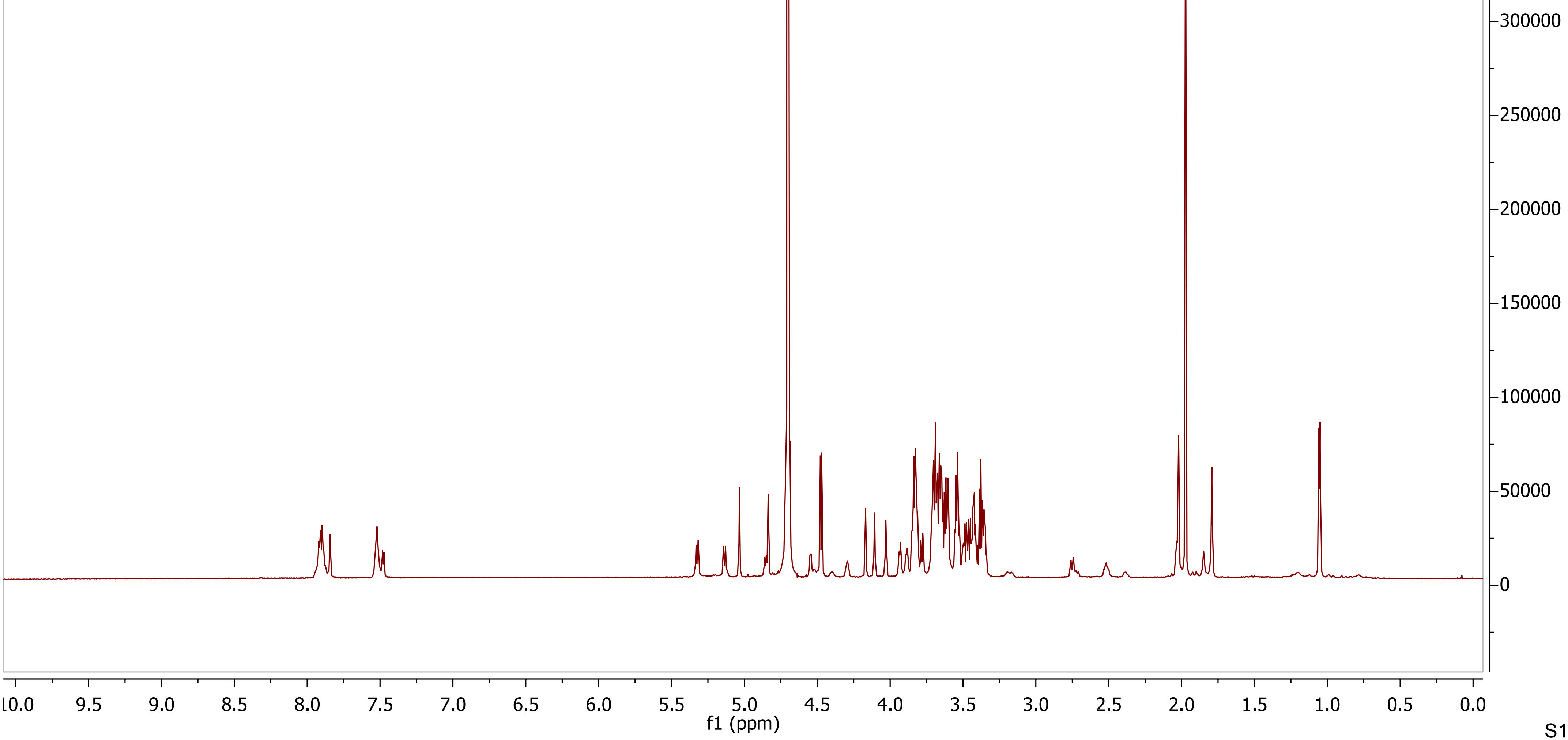

skb-251_A-Nap_P2.2.fid
skb-251_A-Nap_P2

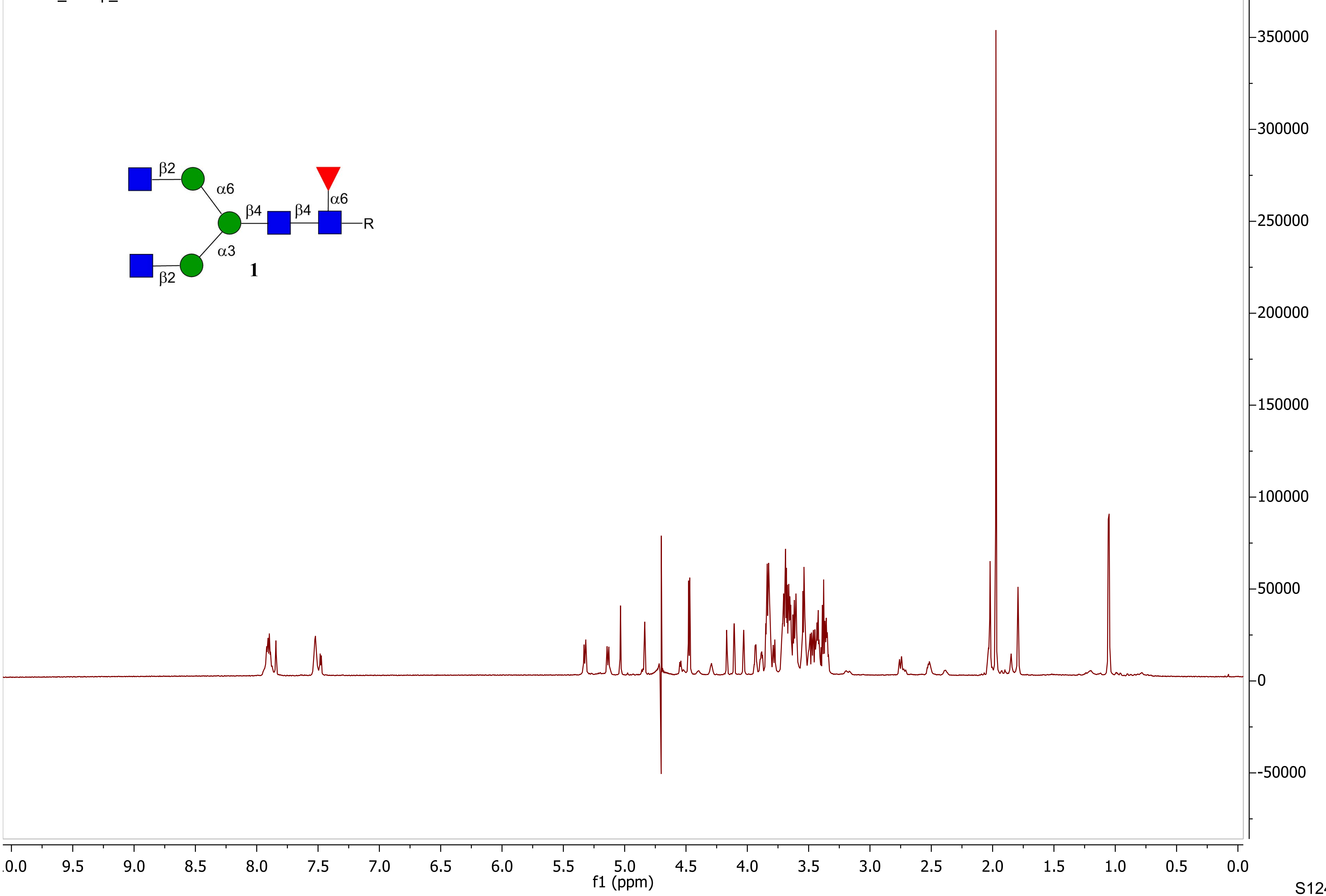

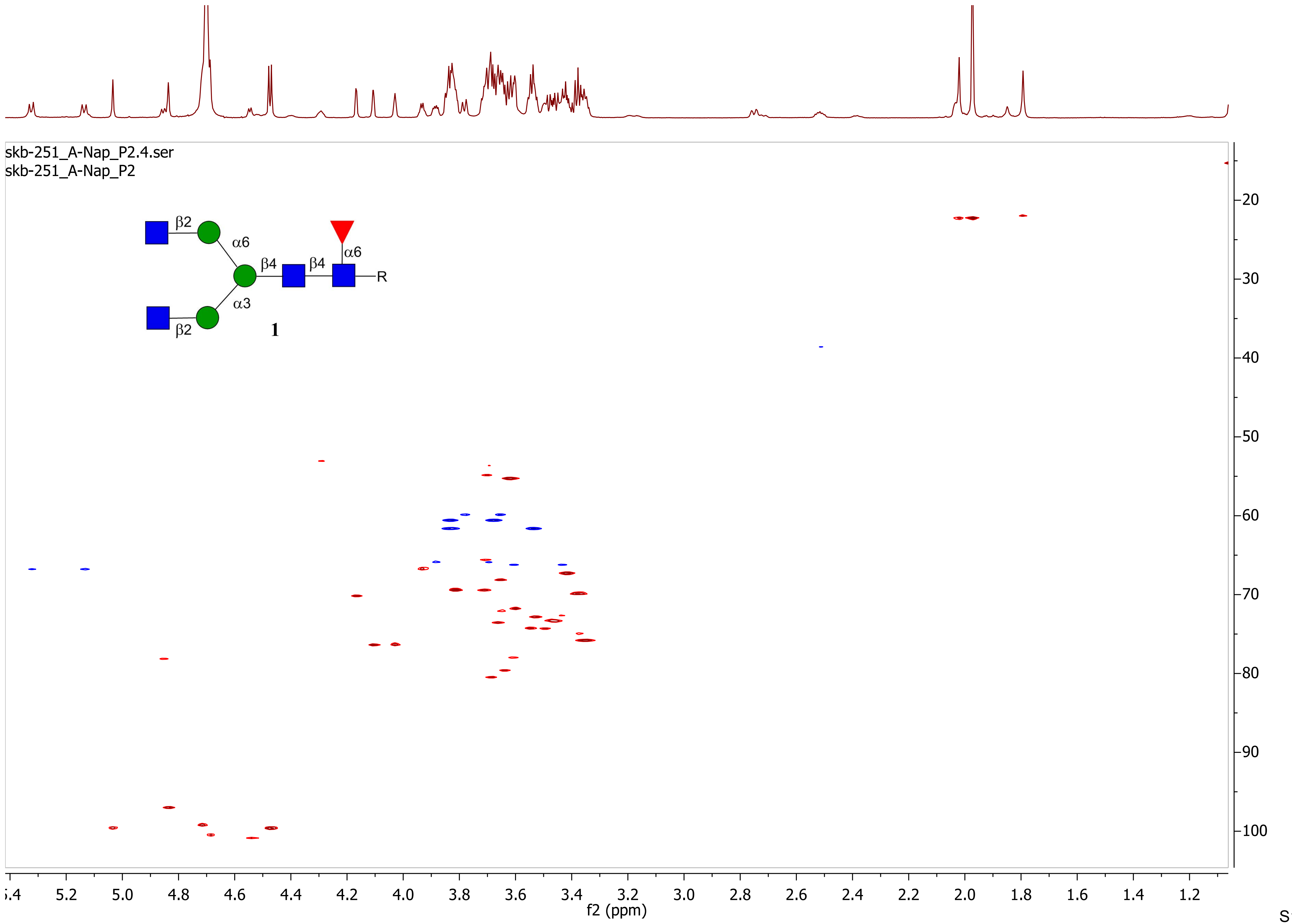

skb-251_A-Nap_P2.4.ser
skb-251_A-Nap_P2
β2
α6
β4
β4
α6
R
α3
β2
1
f2 (ppm)
f1 (ppm)

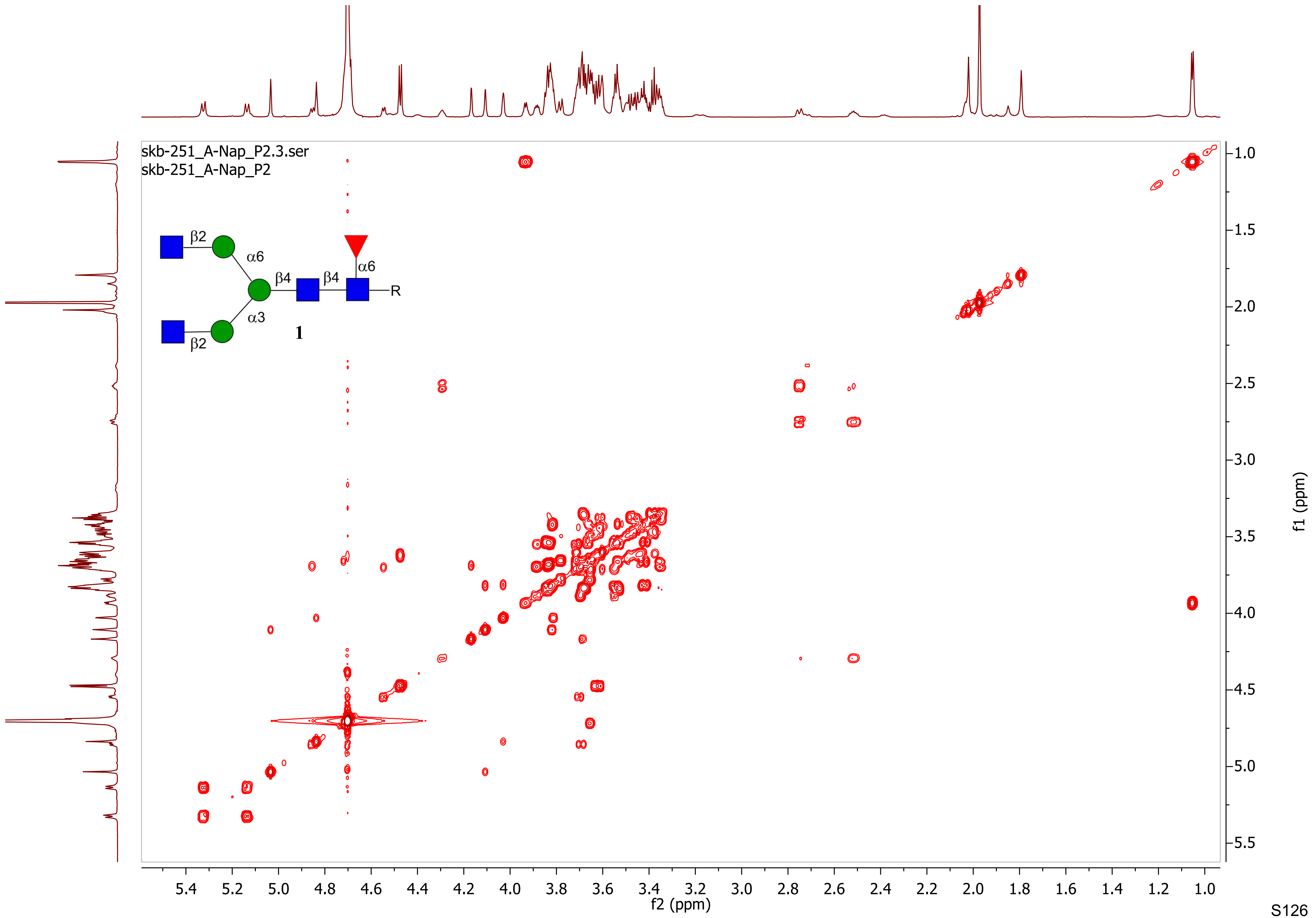
skb-251_A-Nap_P2.3.ser
skb-251_A-Nap_P2
β2
α6
β4
β4
α6
R
α3
β2
1
β2
5.4
5.2
5.0
4.8
4.6
4.4
4.2
4.0
3.8
3.6
3.4
3.2
3.0
2.8
2.6
2.4
2.2
2.0
1.8
1.6
1.4
1.2
1.0
f2 (ppm)
1.0
1.5
2.0
2.5
3.0
3.5
4.0
4.5
5.0
5.5
f1 (ppm)

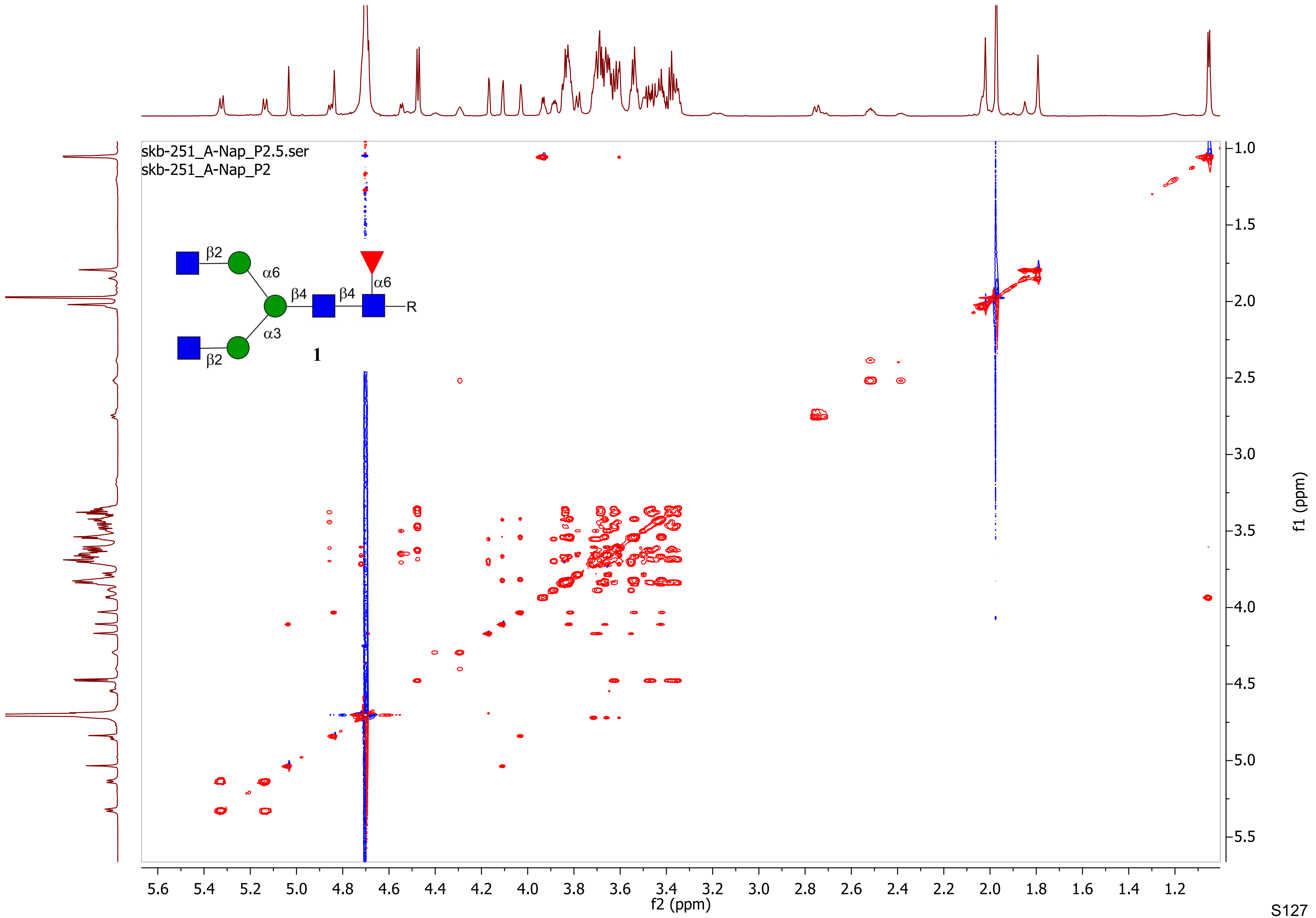

skb-251_A-Nap_P2.5.ser
skb-251_A-Nap_P2
β2
α6
β4
β4
α6
R
α3
β2
1
f2 (ppm)
f1 (ppm)

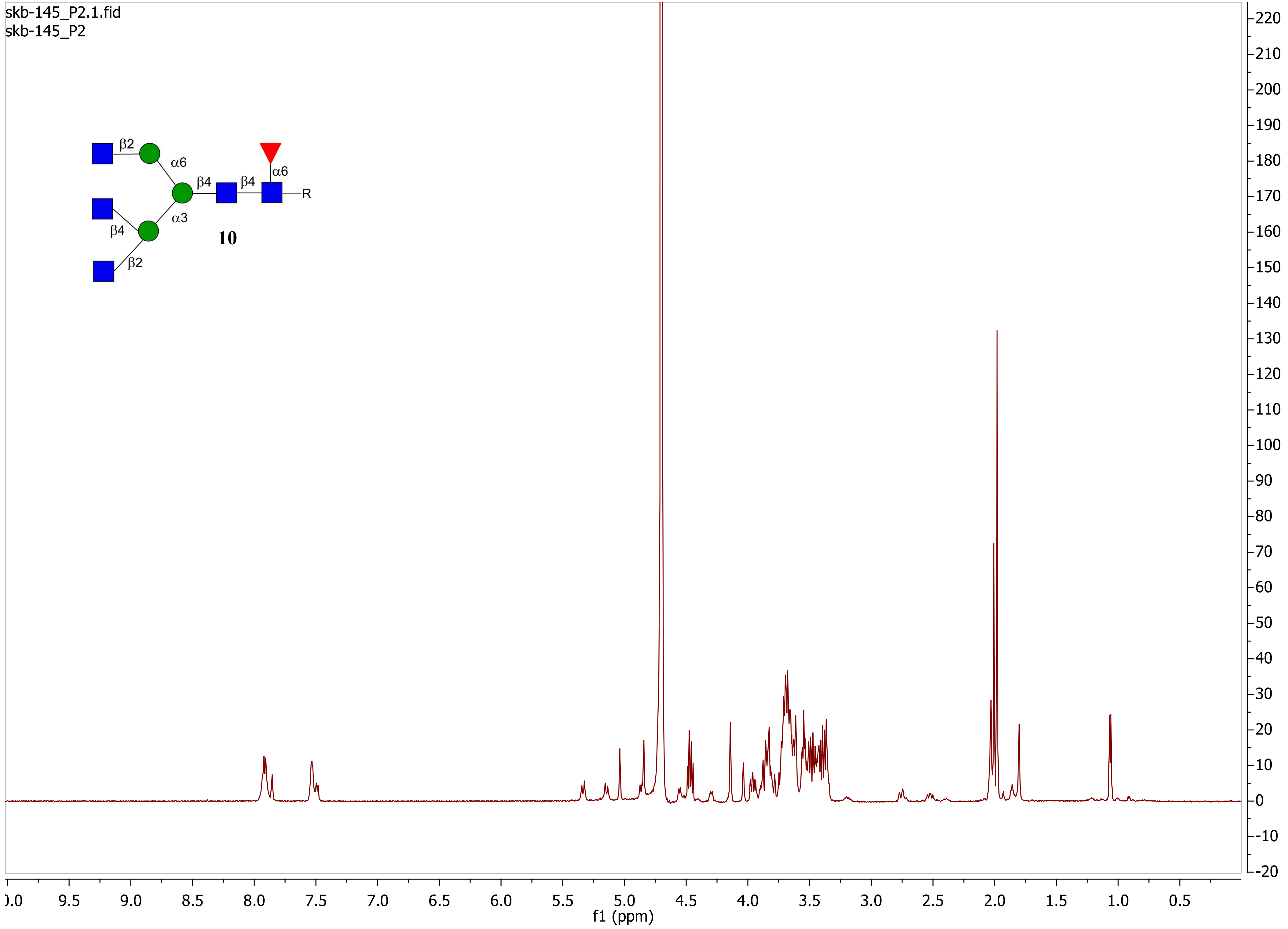

skb-145_P2.1.fid
skb-145_P2
β2
α6
β4
β4
α6
R
β4
α3
10
β2
f1 (ppm)

skb-145_P2.2.fid
skb-145_P2

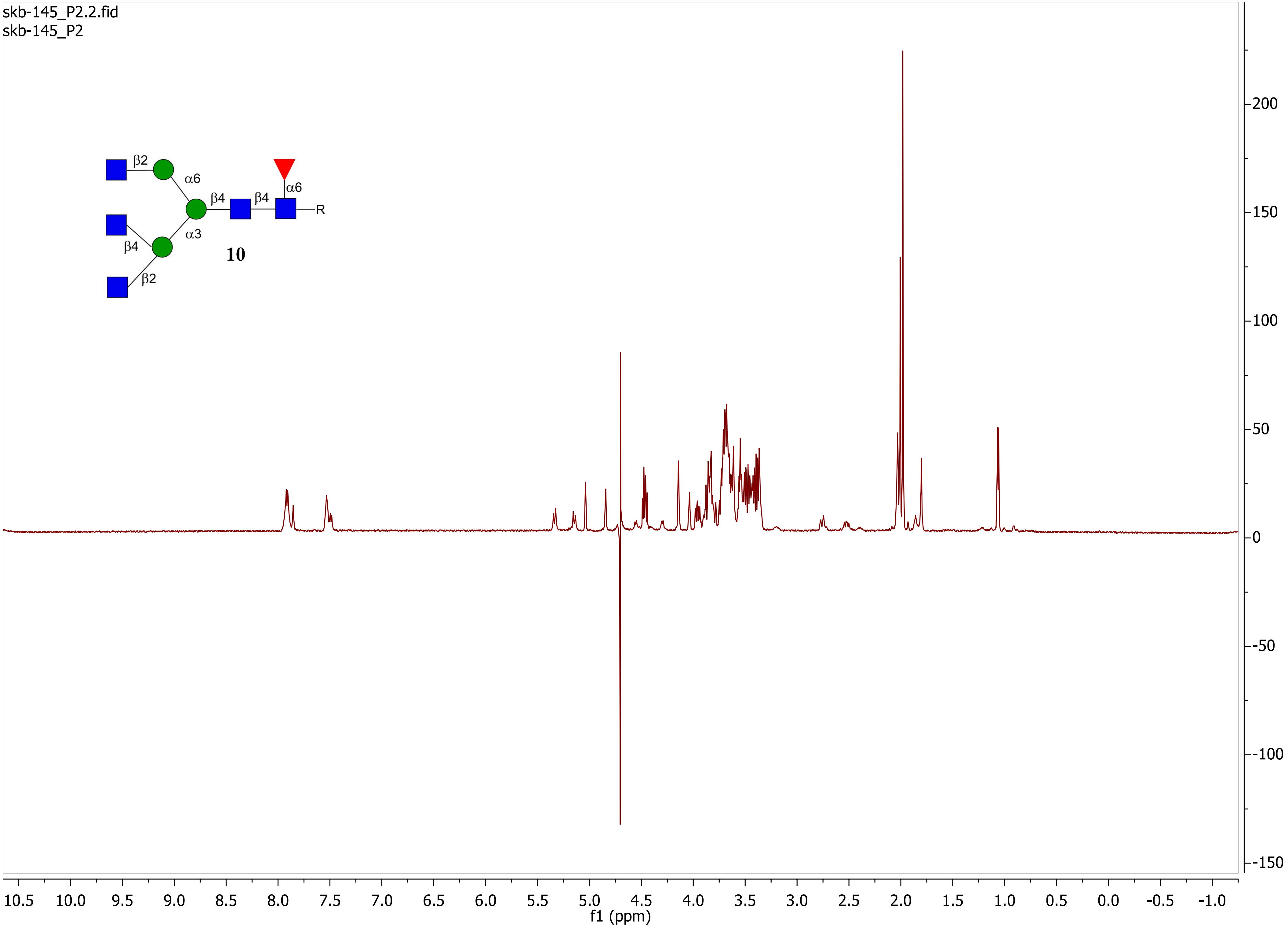

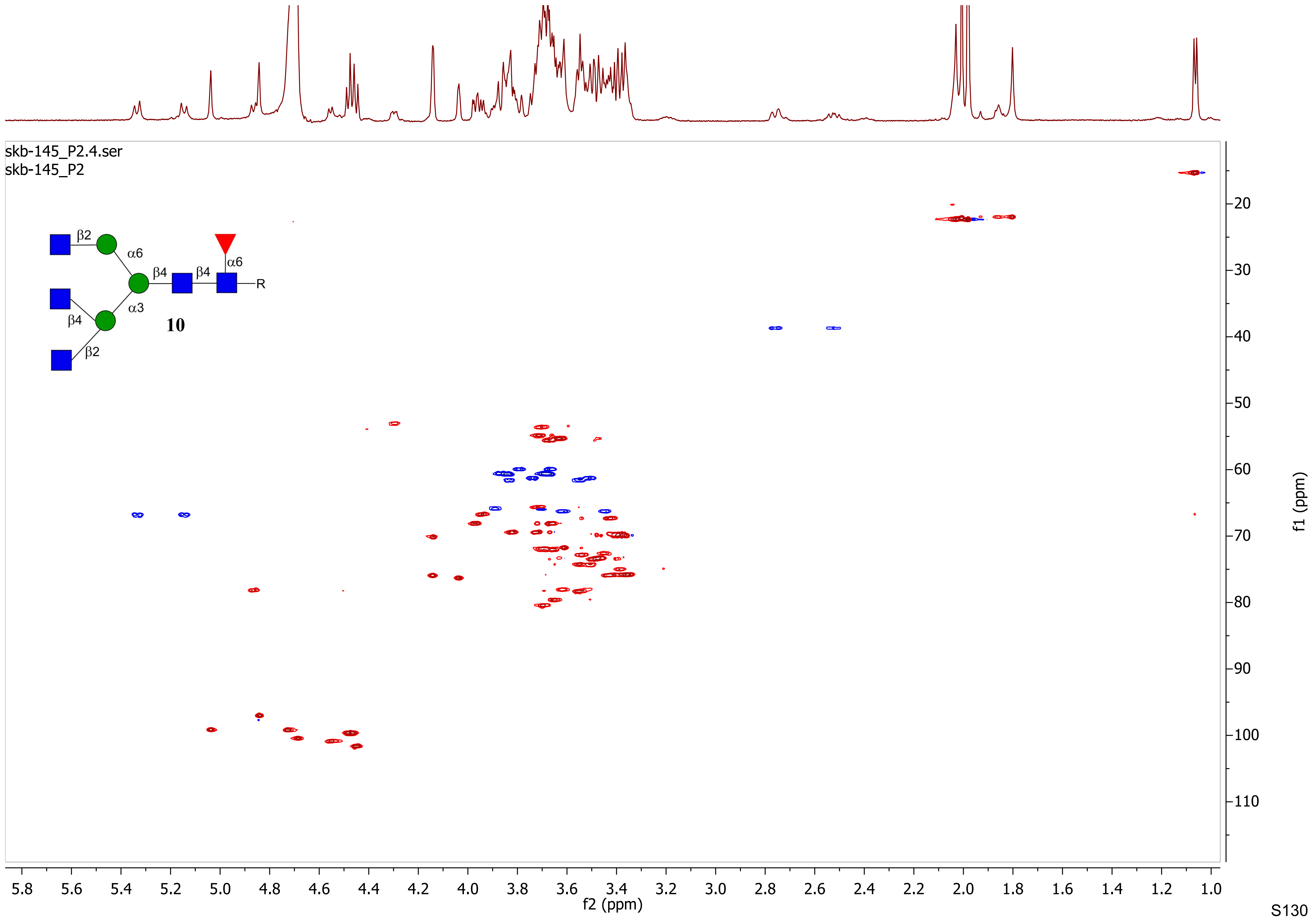
skb-145_P2.4.ser
skb-145_P2
β2
α6
β4
β4
α6
R
α3
β4
β2
10
f2 (ppm)
f1 (ppm)

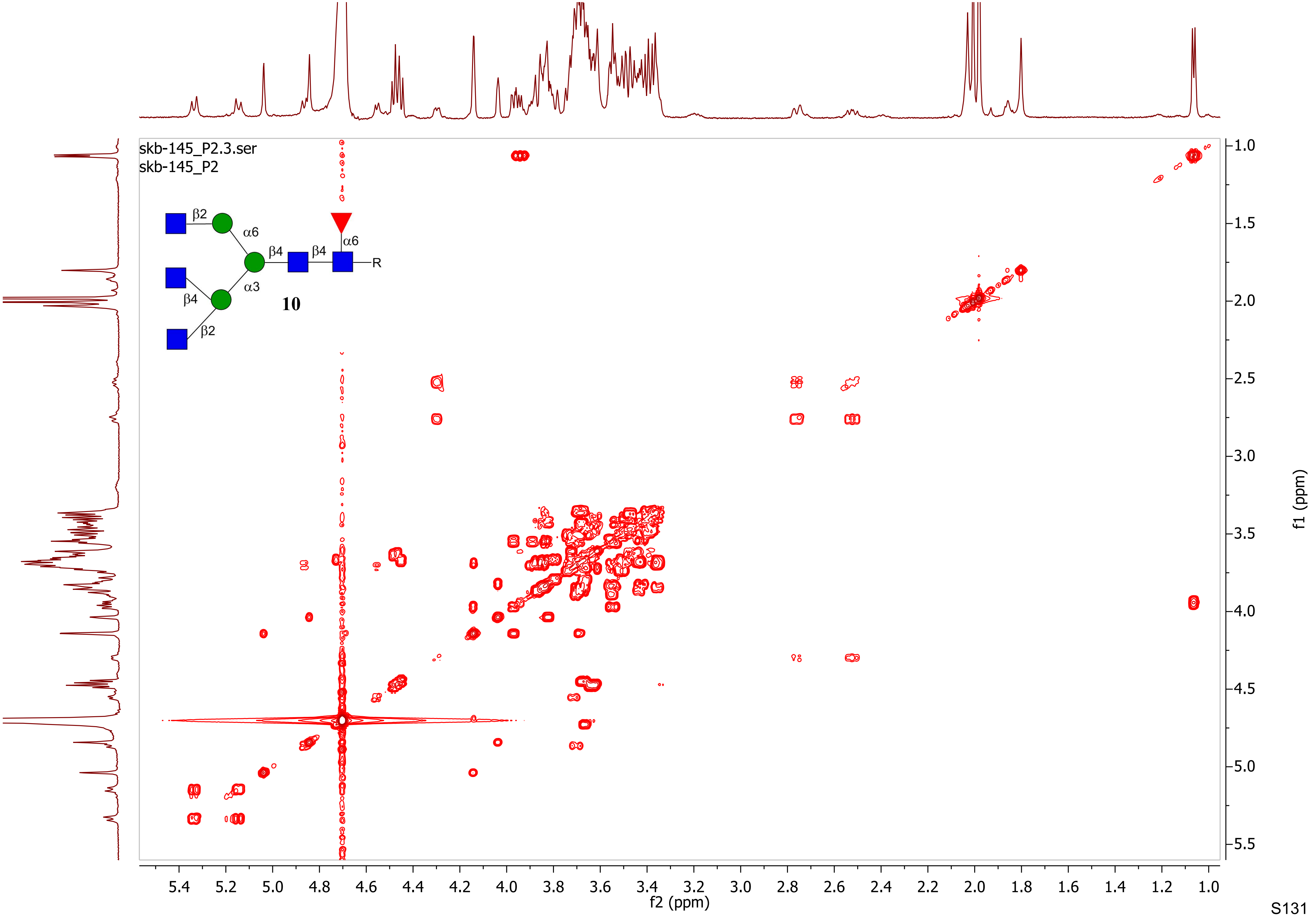
skb-145_P2.3.ser
skb-145_P2
β2
α6
β4
β4
α6
R
α3
β4
10
β2
f2 (ppm)
f1 (ppm)

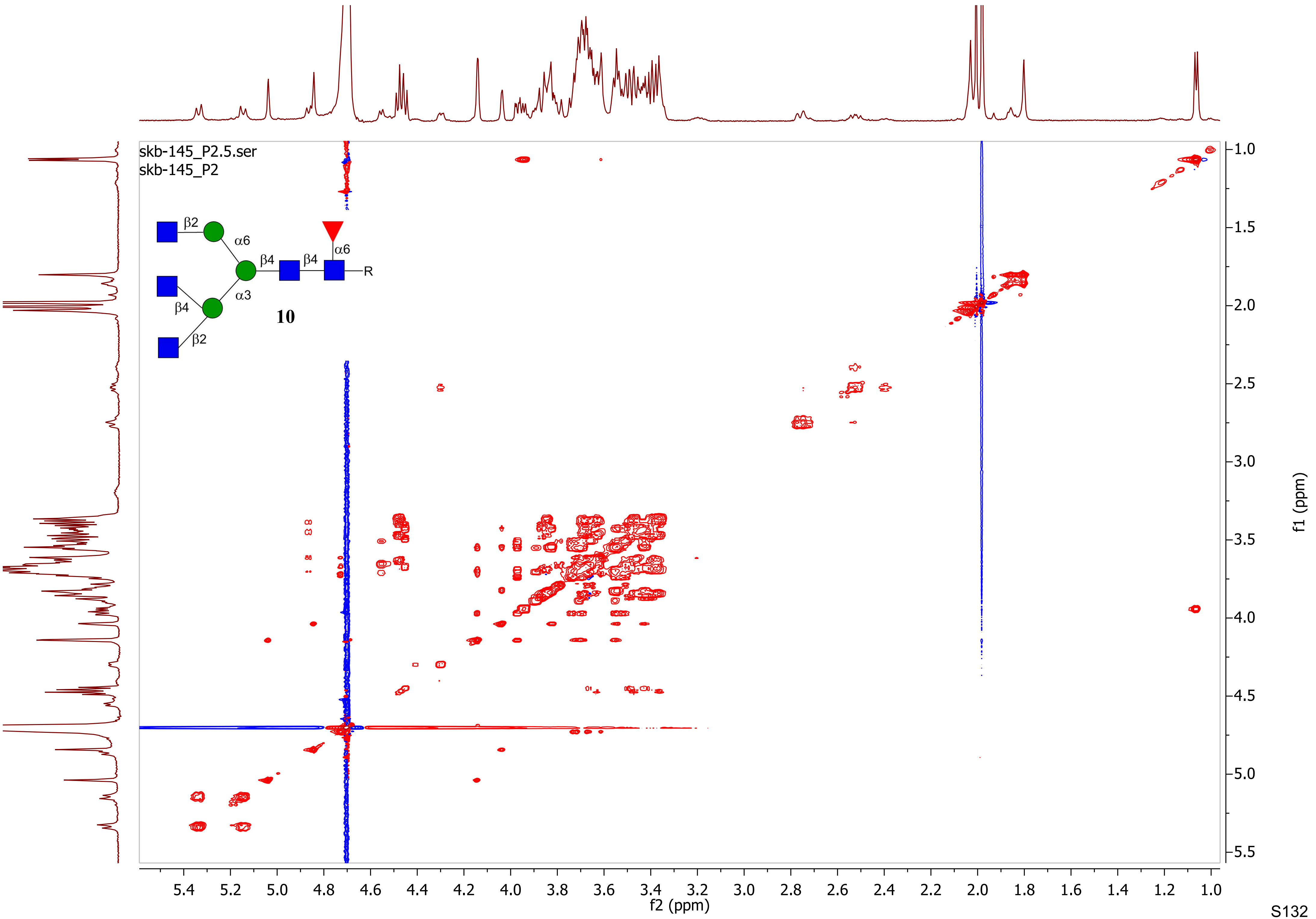
skb-145_P2.5.ser
skb-145_P2
β2
α6
β4
β4
α6
R
β4
α3
10
β2
f2 (ppm)
f1 (ppm)

skb-151_P4.1.fid
skb-151_P4

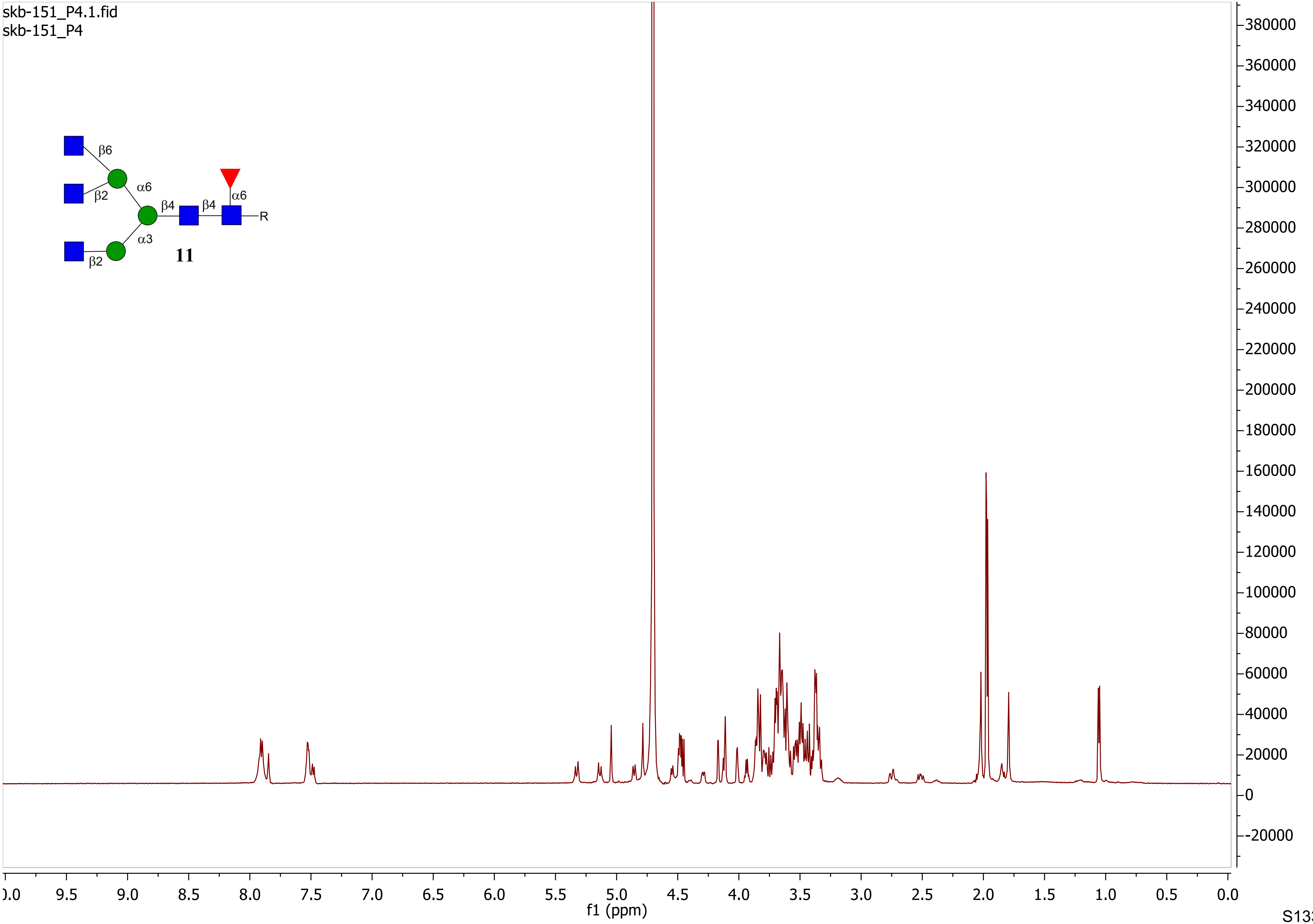

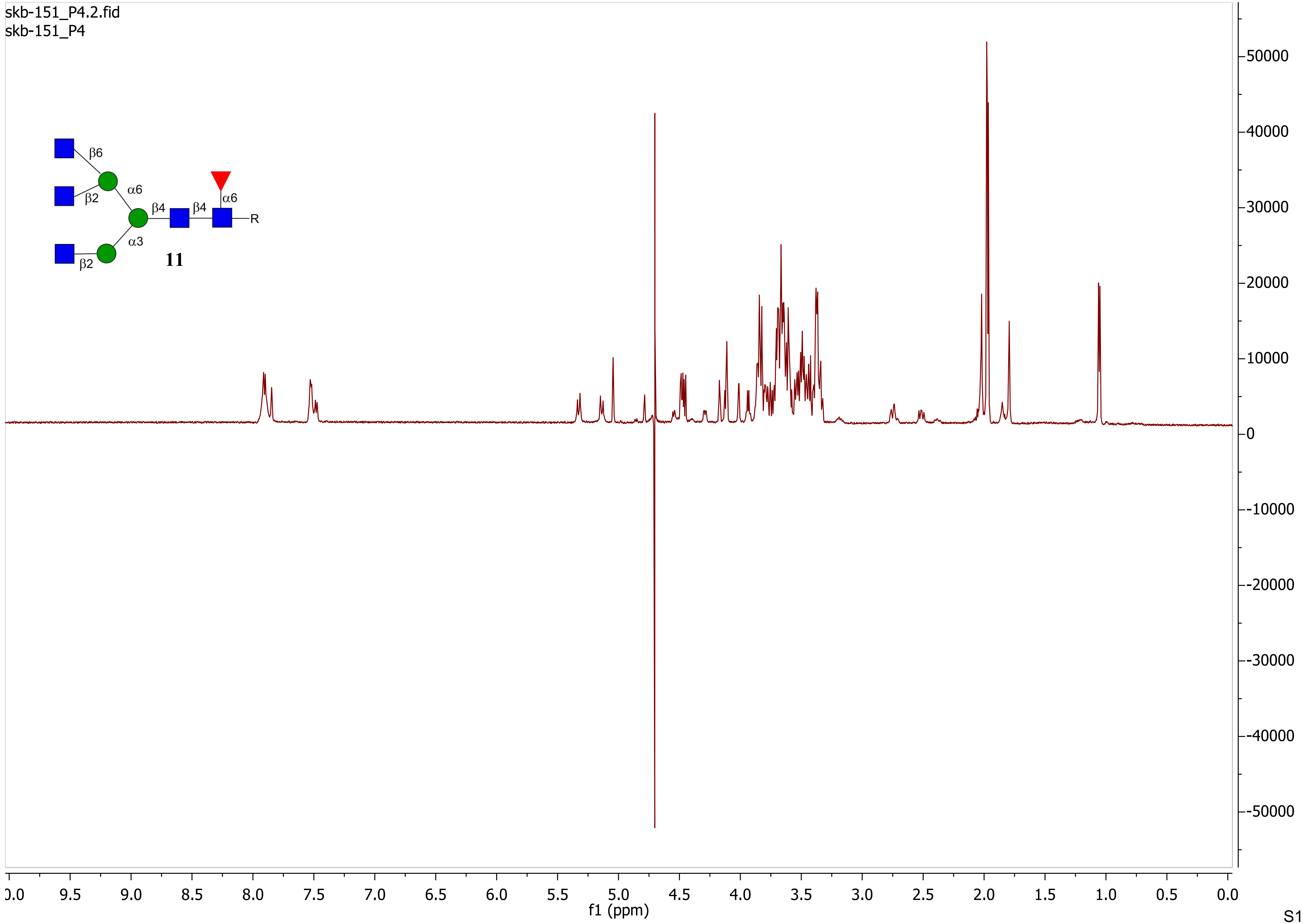
skb-151_P4.2.fid
skb-151_P4
β6
α6
β2
β4
β4
α6
R
α3
β2
11
f1 (ppm)

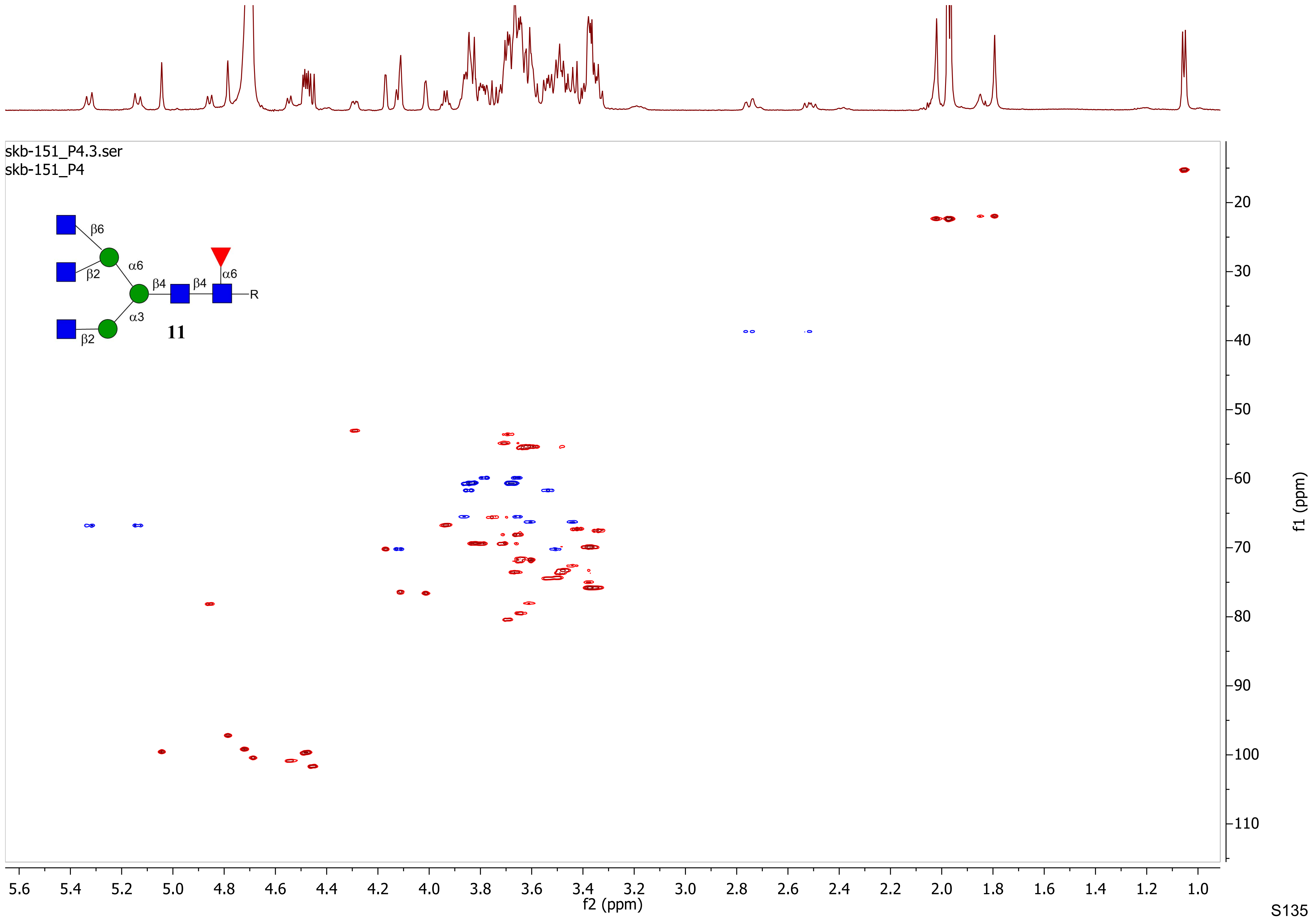

skb-151_P4.3.ser
skb-151_P4
β6
β2
α6
β4
β4
α6
R
α3
β2
11
20
30
40
50
60
70
80
90
100
110
f1 (ppm)
5.6
5.4
5.2
5.0
4.8
4.6
4.4
4.2
4.0
3.8
3.6
3.4
3.2
3.0
2.8
2.6
2.4
2.2
2.0
1.8
1.6
1.4
1.2
1.0
f2 (ppm)

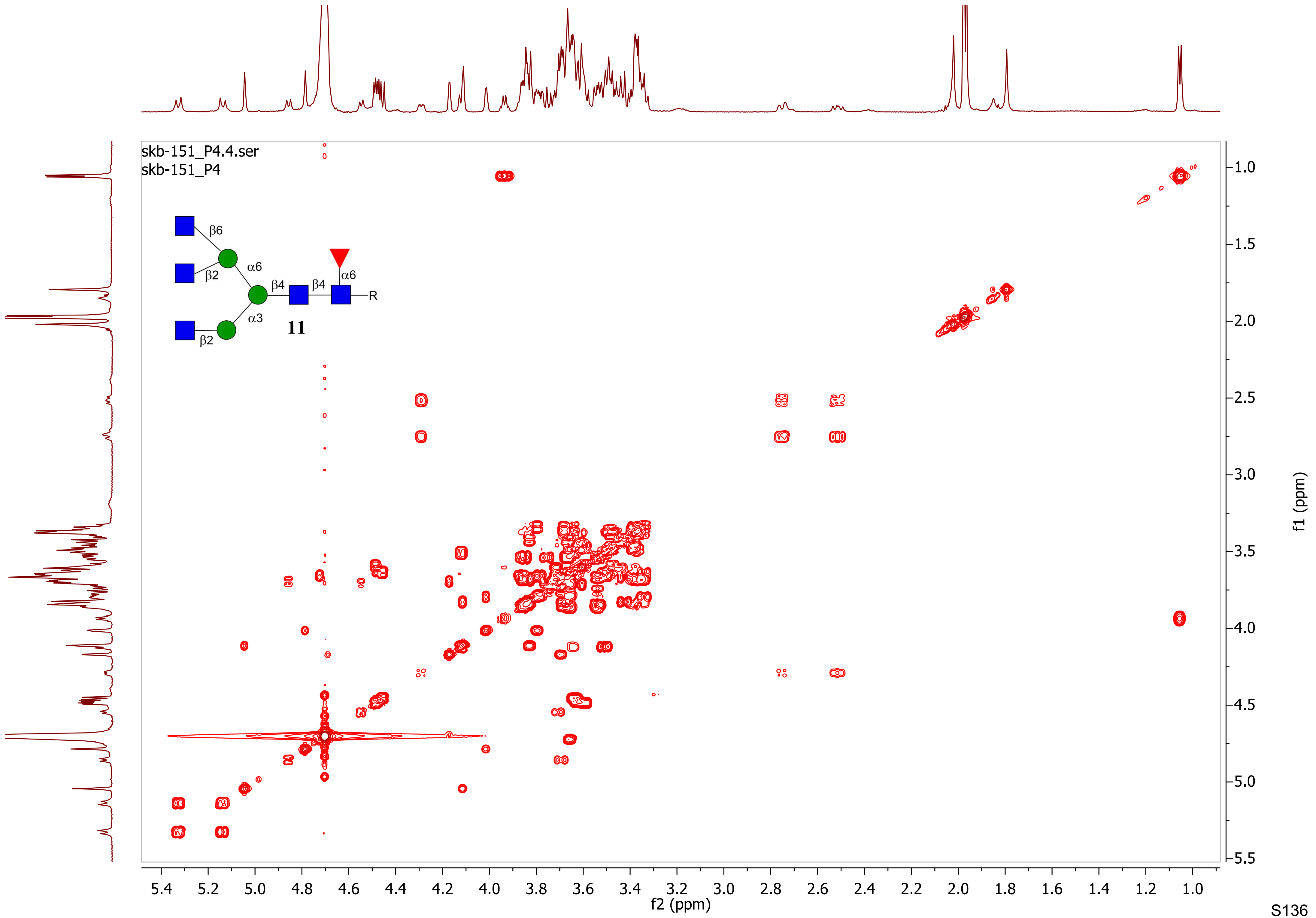

skb-151_P4.4.ser
skb-151_P4
β6
β2
α6
β4
β4
α6
R
α3
β2
11
f2 (ppm)
f1 (ppm)

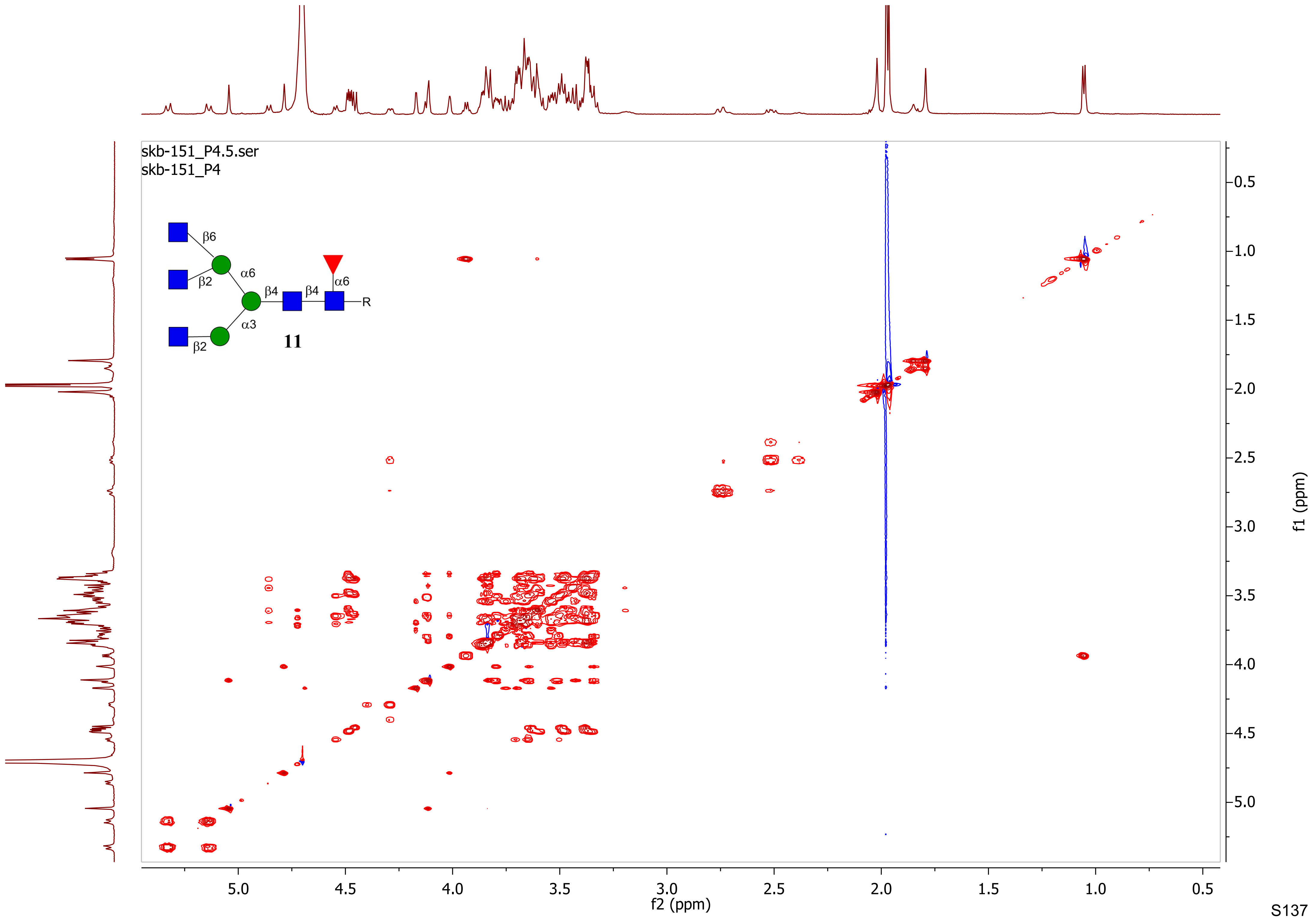

skb-151_P4.5.ser
skb-151_P4
β6
α6
β2
β4
β4
α6
R
α3
β2
11
f2 (ppm)
f1 (ppm)

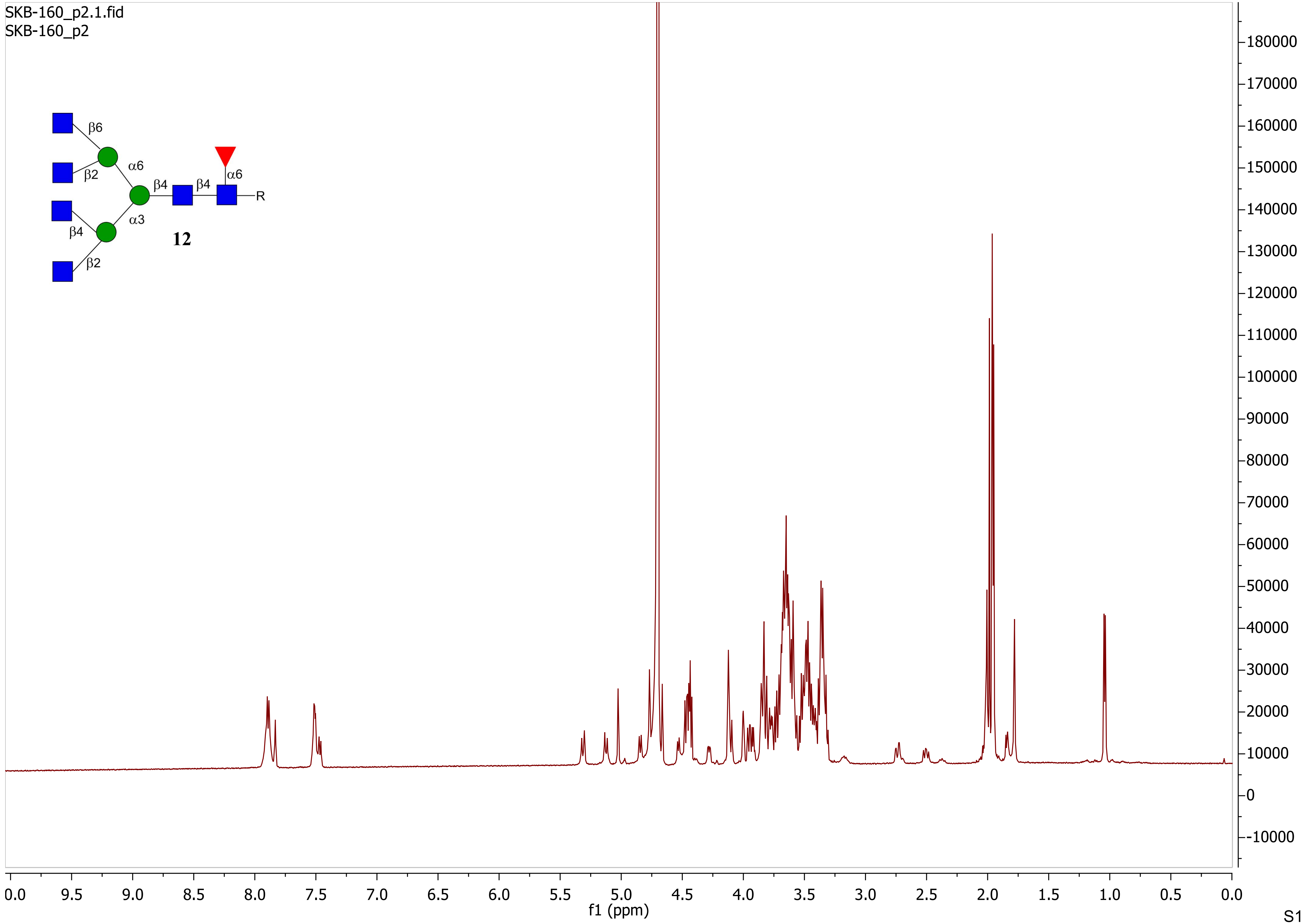
SKB-160_p2.1.fid
SKB-160_p2
β6
α6
β2
β4
β4
α6
R
α3
β4
β2
12
f1 (ppm)

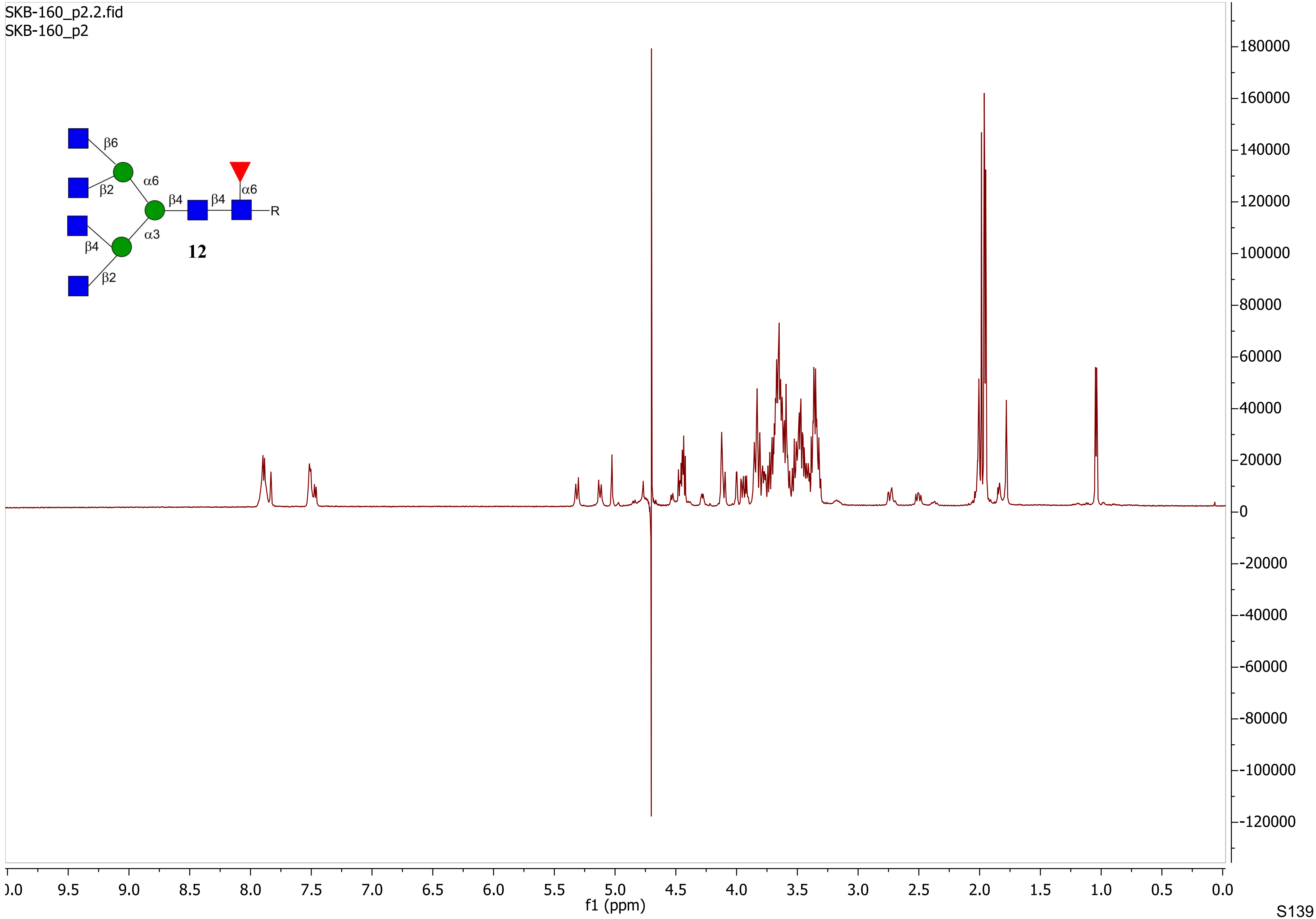
SKB-160_p2.2.fid
SKB-160_p2
β6
α6
β2
β4
β4
α6
R
β4
α3
β2
12
180000
160000
140000
120000
100000
80000
60000
40000
20000
0
-20000
-40000
-60000
-80000
-100000
-120000
9.5
9.0
8.5
8.0
7.5
7.0
6.5
6.0
5.5
5.0
4.5
4.0
3.5
3.0
2.5
2.0
1.5
1.0
0.5
0.0
f1 (ppm)

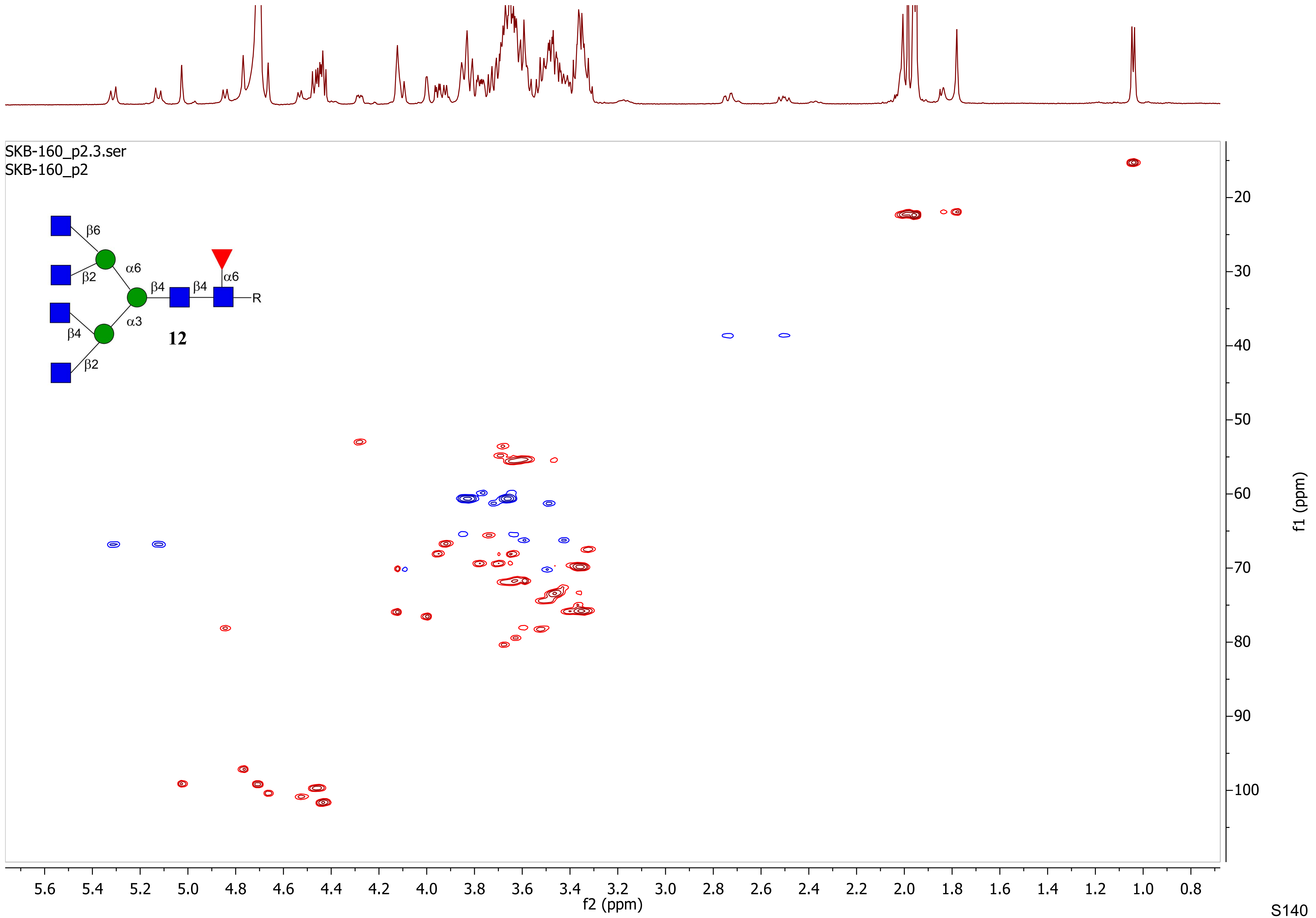

SKB-160_p2.3.ser
SKB-160_p2
β6
β2
α6
β4
β4
α6
R
β4
α3
β2
12
f2 (ppm)
f1 (ppm)

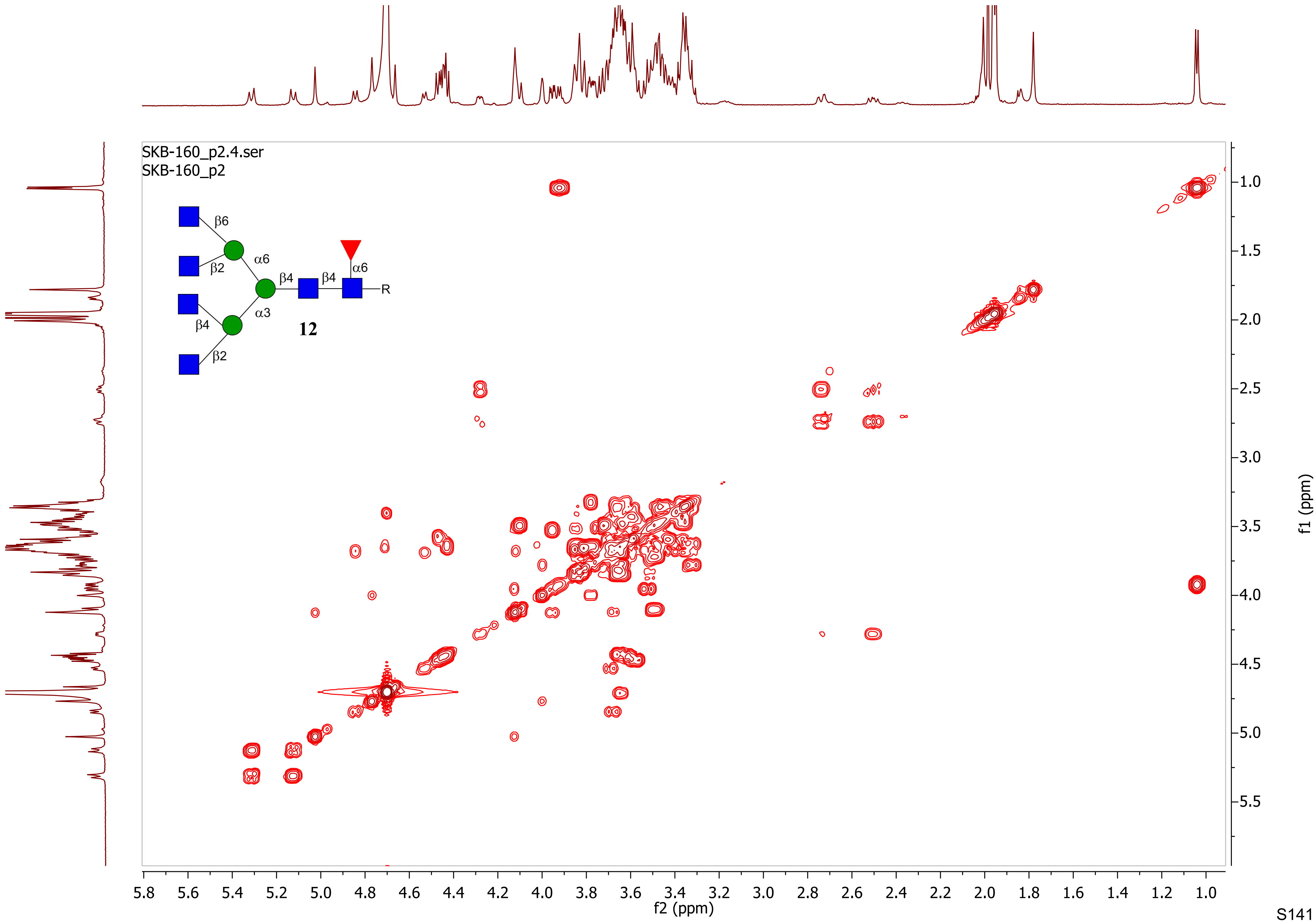
SKB-160_p2.4.ser
SKB-160_p2
β6
β2
α6
β4
β4
α6
R
β4
α3
β2
12
f2 (ppm)
f1 (ppm)

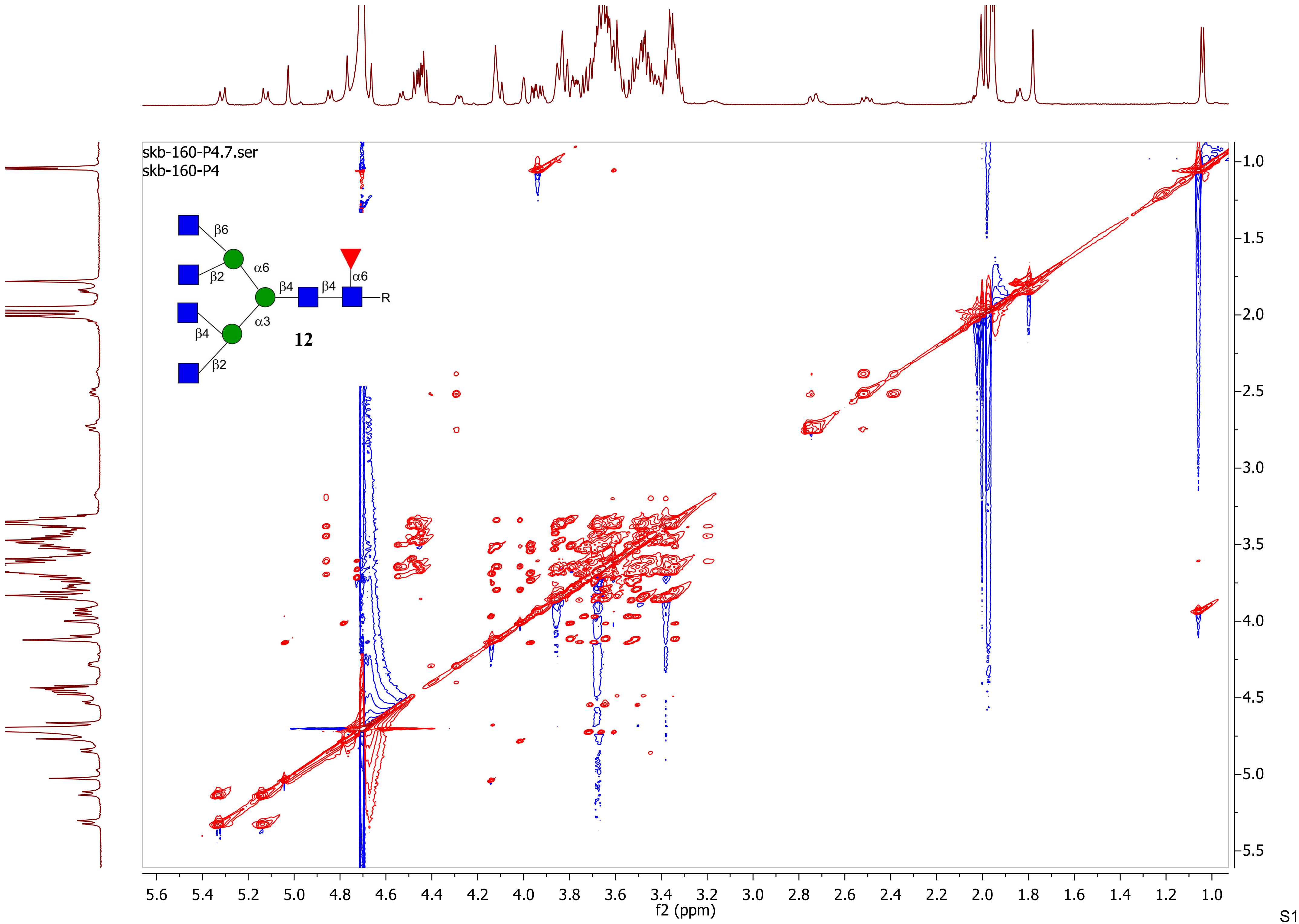
skb-160-P4.7.ser
skb-160-P4
β6
α6
β2
β4
β4
α6
R
α3
β4
β2
12
f2 (ppm)
f1 (ppm)

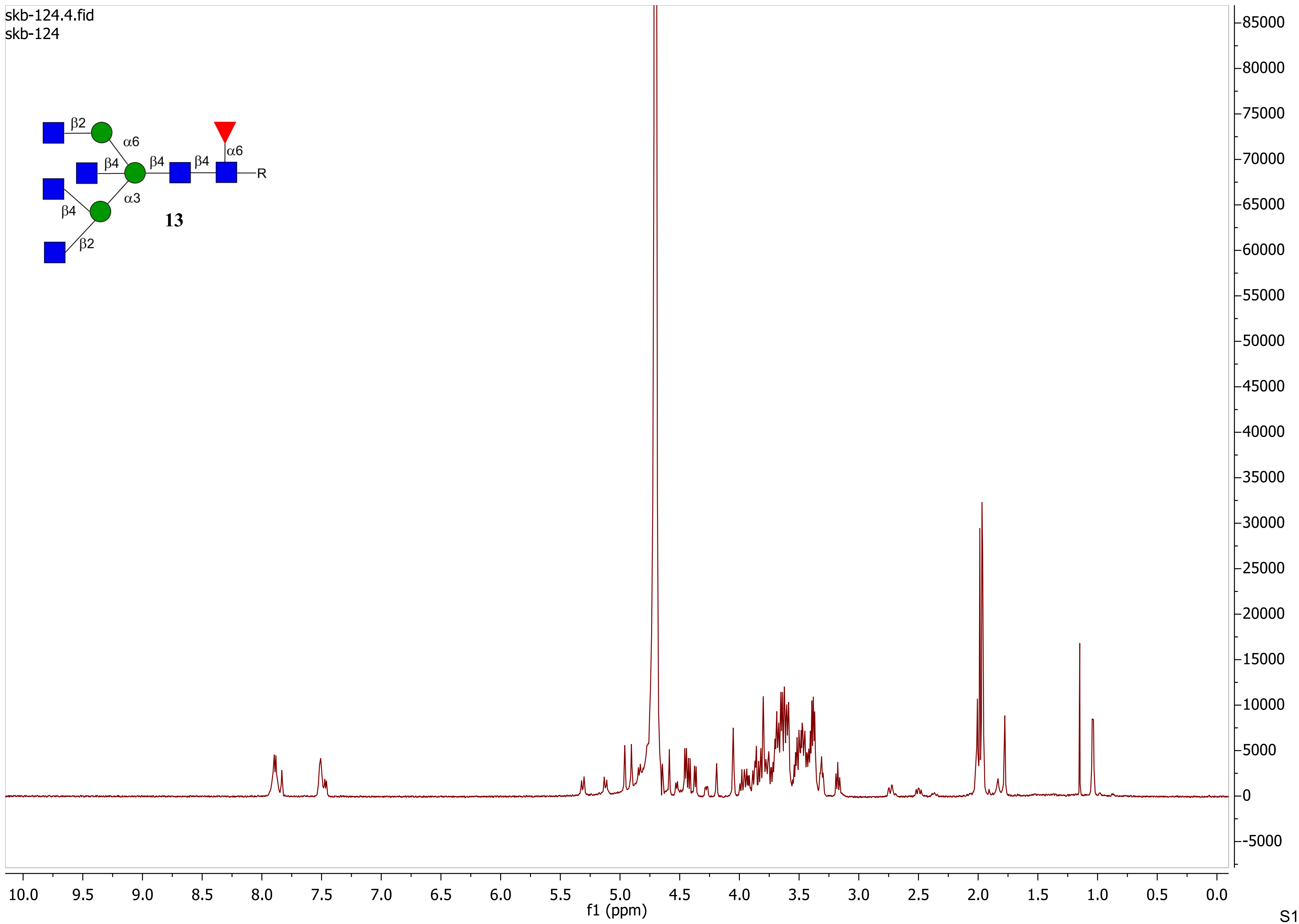
skb-124.4.fid
skb-124
β2
α6
α6
β4
β4
β4
R
α3
β4
13
β2
f1 (ppm)

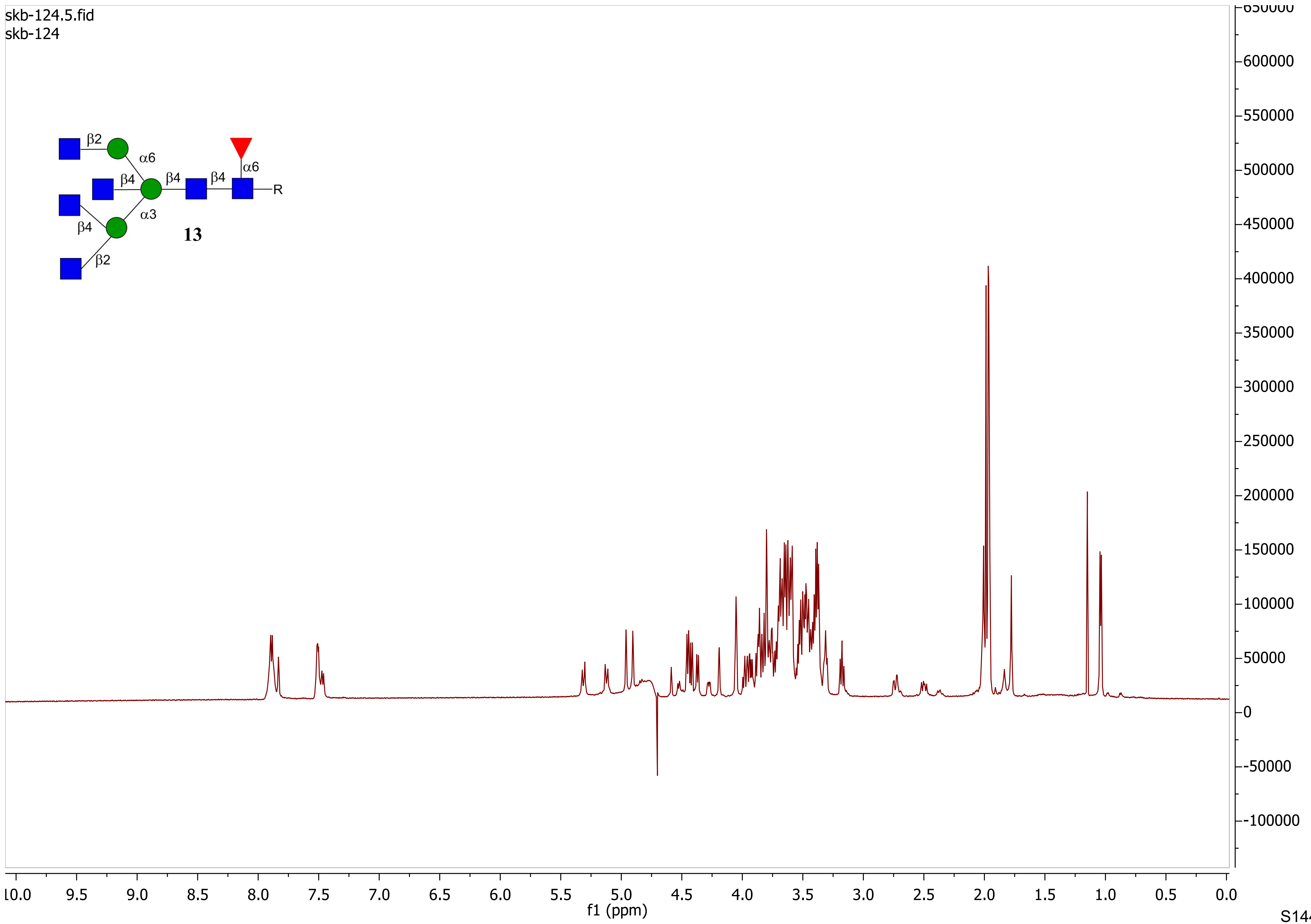

skb-124.5.fid
skb-124
β2
α6
β4
β4
β4
α6
R
β4
α3
13
β2
f1 (ppm)

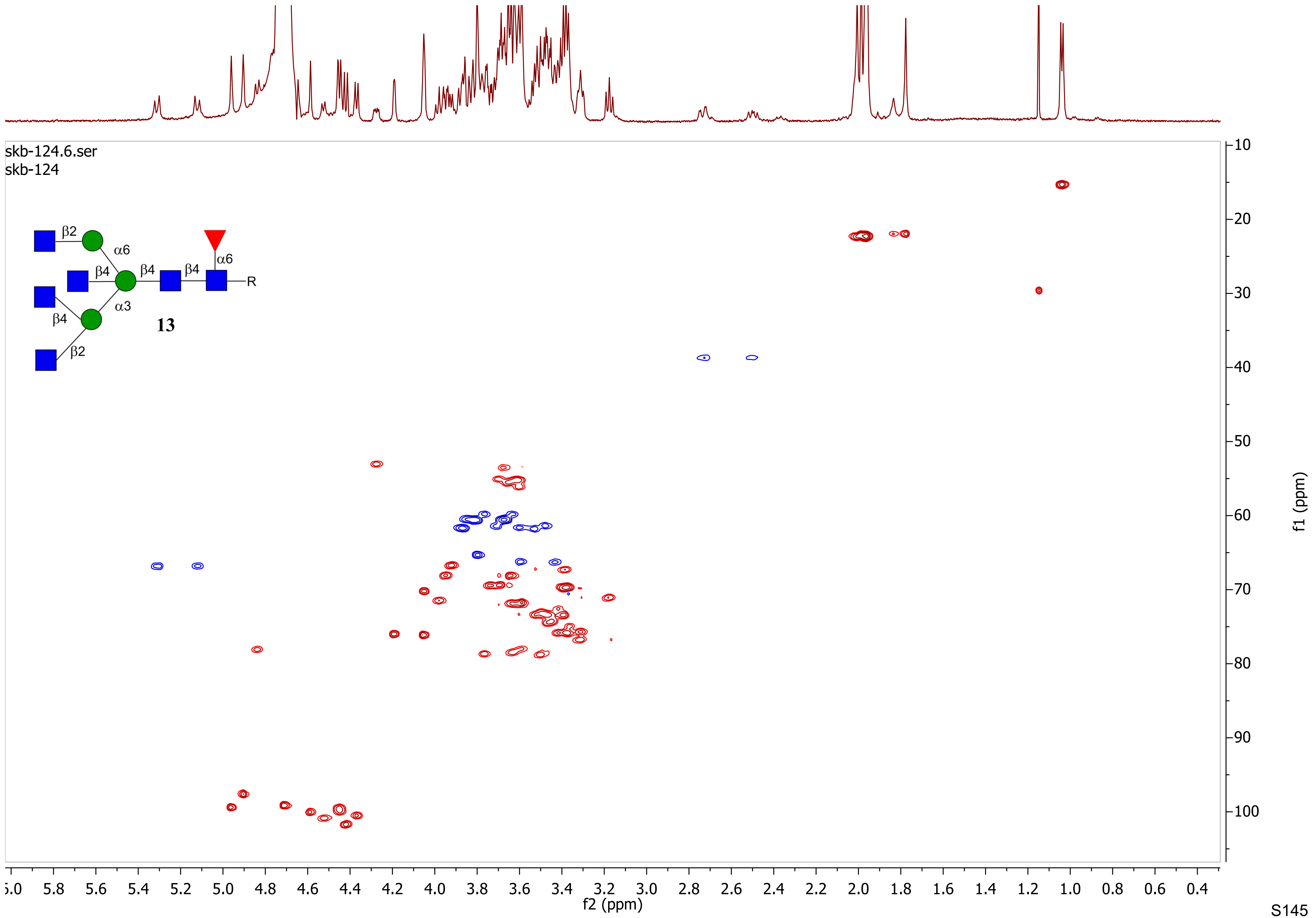

skb-124.6.ser
skb-124
β2
α6
β4
β4
β4
α6
R
α3
β4
β2
13
f2 (ppm)
f1 (ppm)

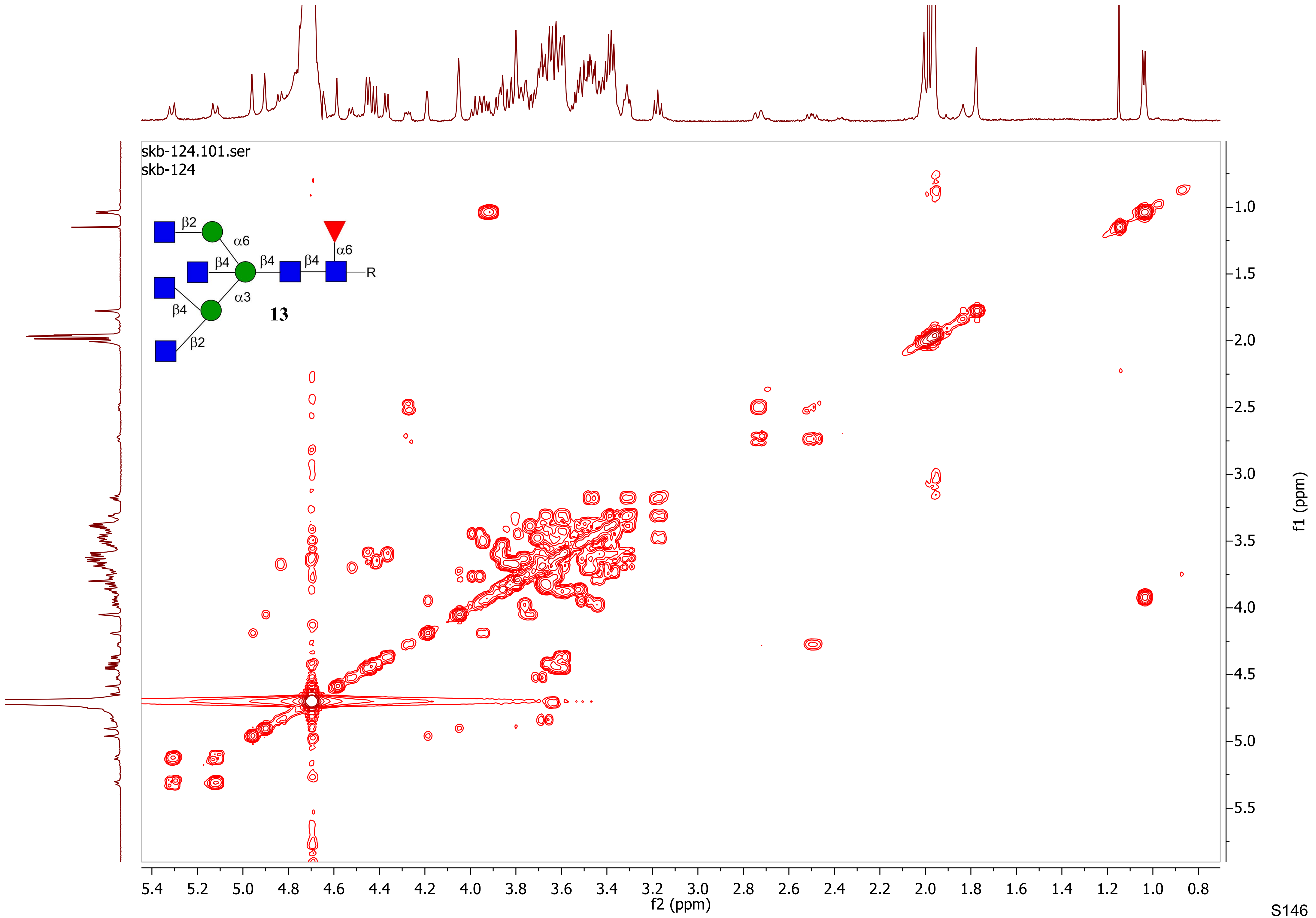
skb-124.101.ser
skb-124
β2
α6
β4
β4
β4
α6
R
α3
β4
β2
13
5.4
5.2
5.0
4.8
4.6
4.4
4.2
4.0
3.8
3.6
3.4
3.2
3.0
2.8
2.6
2.4
2.2
2.0
1.8
1.6
1.4
1.2
1.0
0.8
f2 (ppm)
1.0
1.5
2.0
2.5
3.0
3.5
4.0
4.5
5.0
5.5
f1 (ppm)

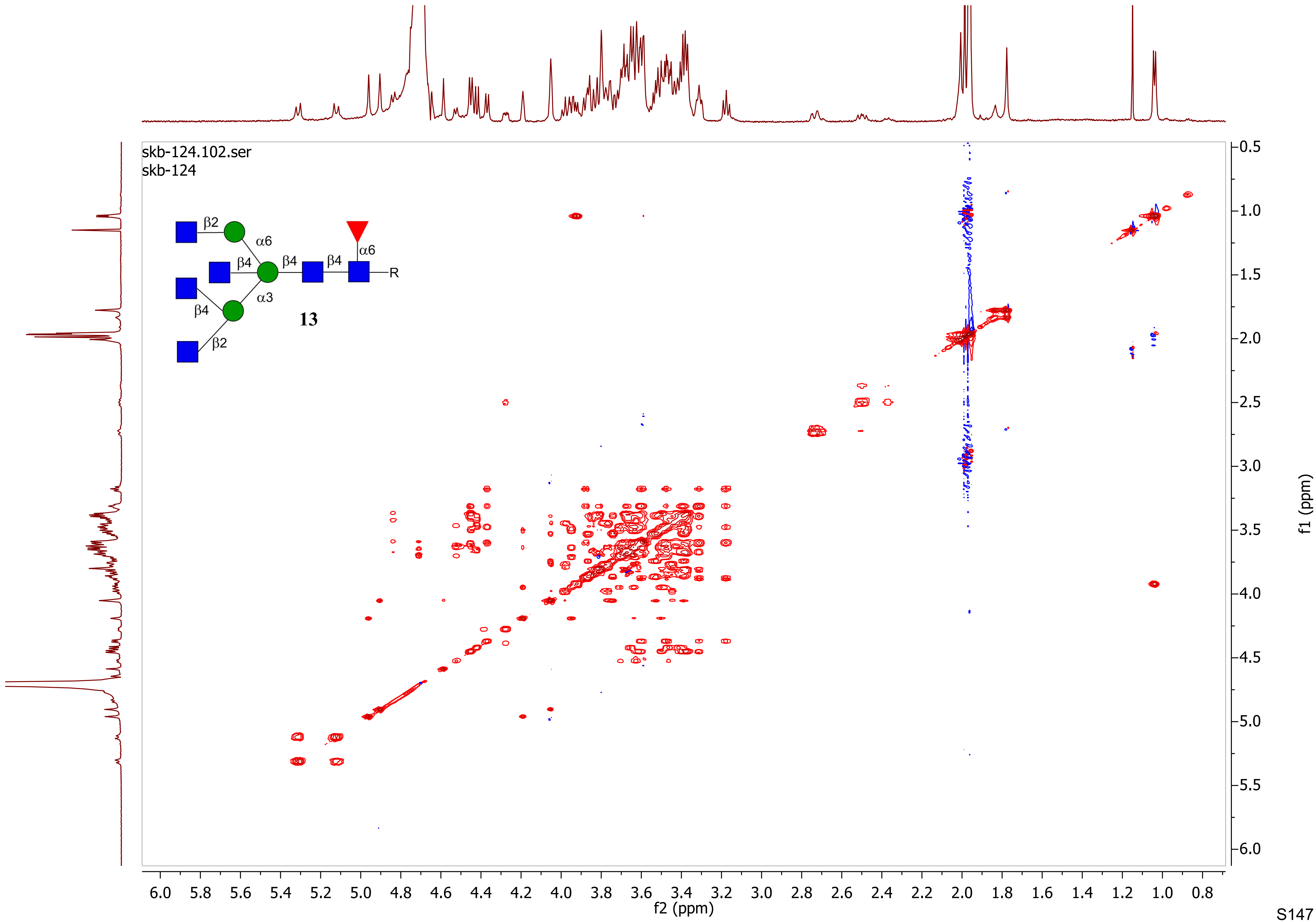
skb-124.102.ser
skb-124
β2
α6
β4
β4
β4
α6
R
β4
α3
β2
13
f1 (ppm)
f2 (ppm)
0.5
1.0
1.5
2.0
2.5
3.0
3.5
4.0
4.5
5.0
5.5
6.0
6.0
5.8
5.6
5.4
5.2
5.0
4.8
4.6
4.4
4.2
4.0
3.8
3.6
3.4
3.2
3.0
2.8
2.6
2.4
2.2
2.0
1.8
1.6
1.4
1.2
1.0
0.8

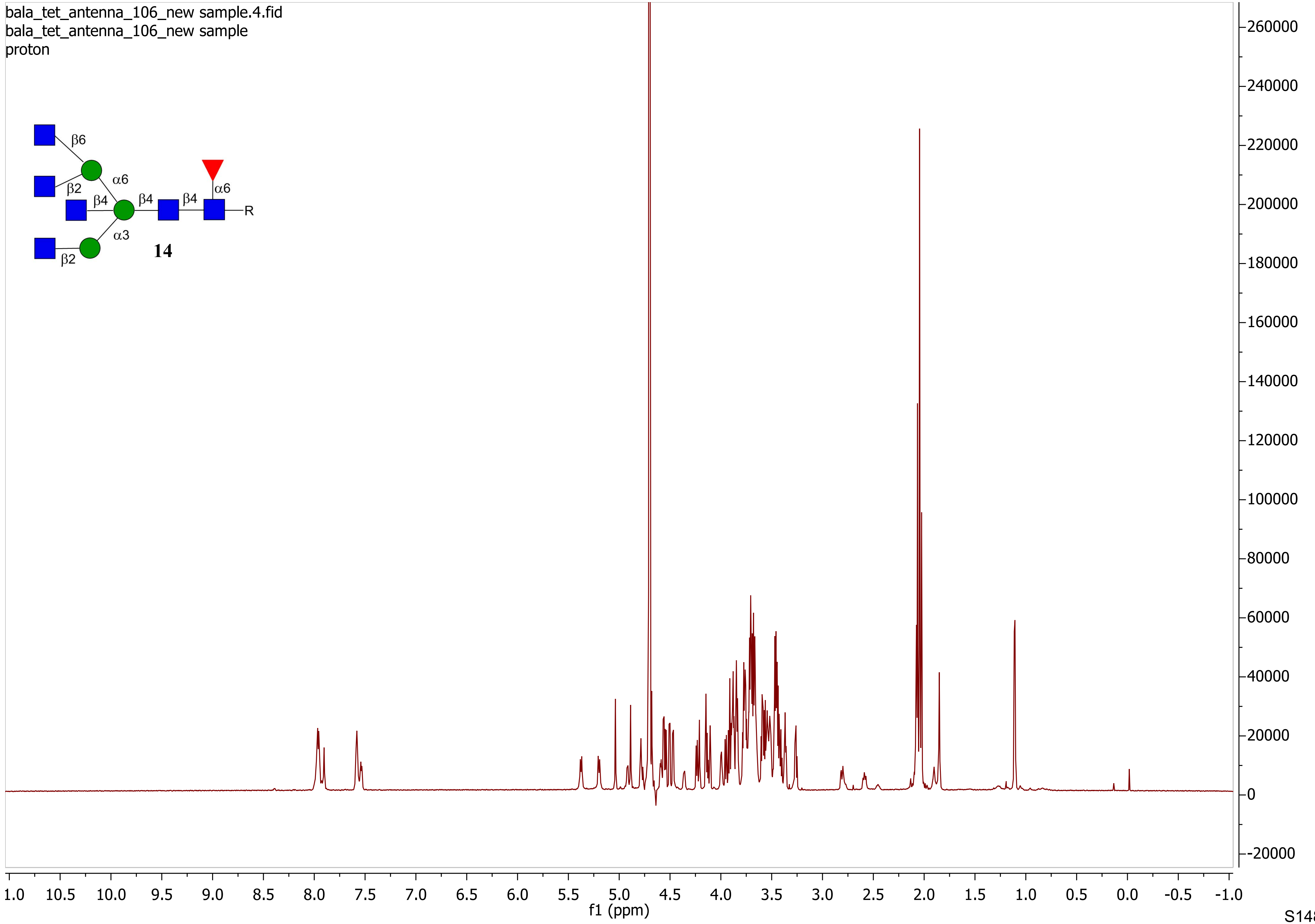
bala_tet_antenna_106_new sample.4.fid
bala_tet_antenna_106_new sample
proton
β6
β2
α6
β4
β4
β4
α6
R
α3
β2
14
f1 (ppm)

bala_tet_antenna_106_new sample.3.fid
bala_tet_antenna_106_new sample
noesy 30 C

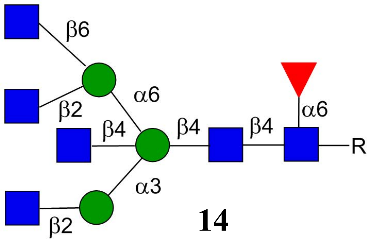

14

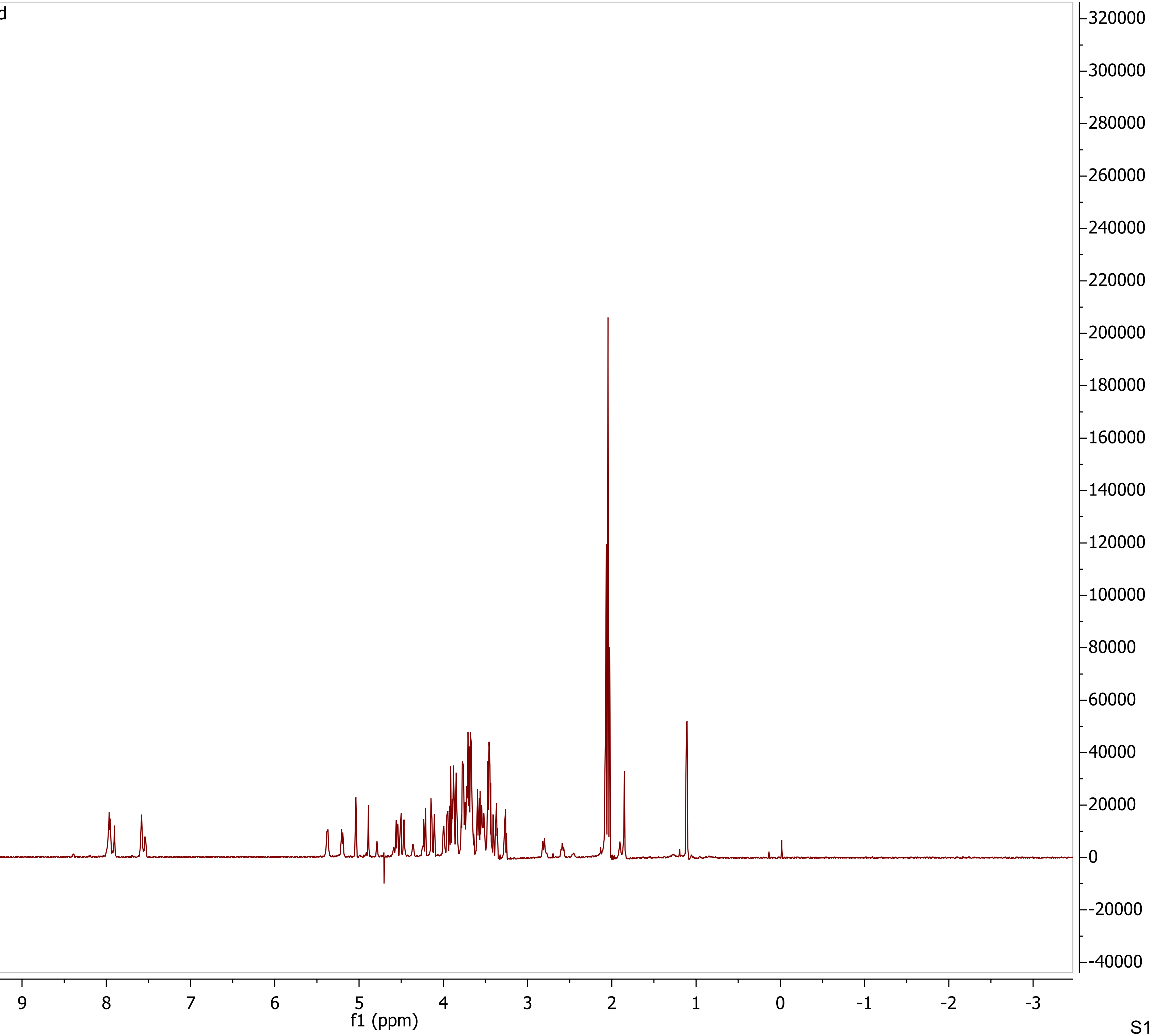

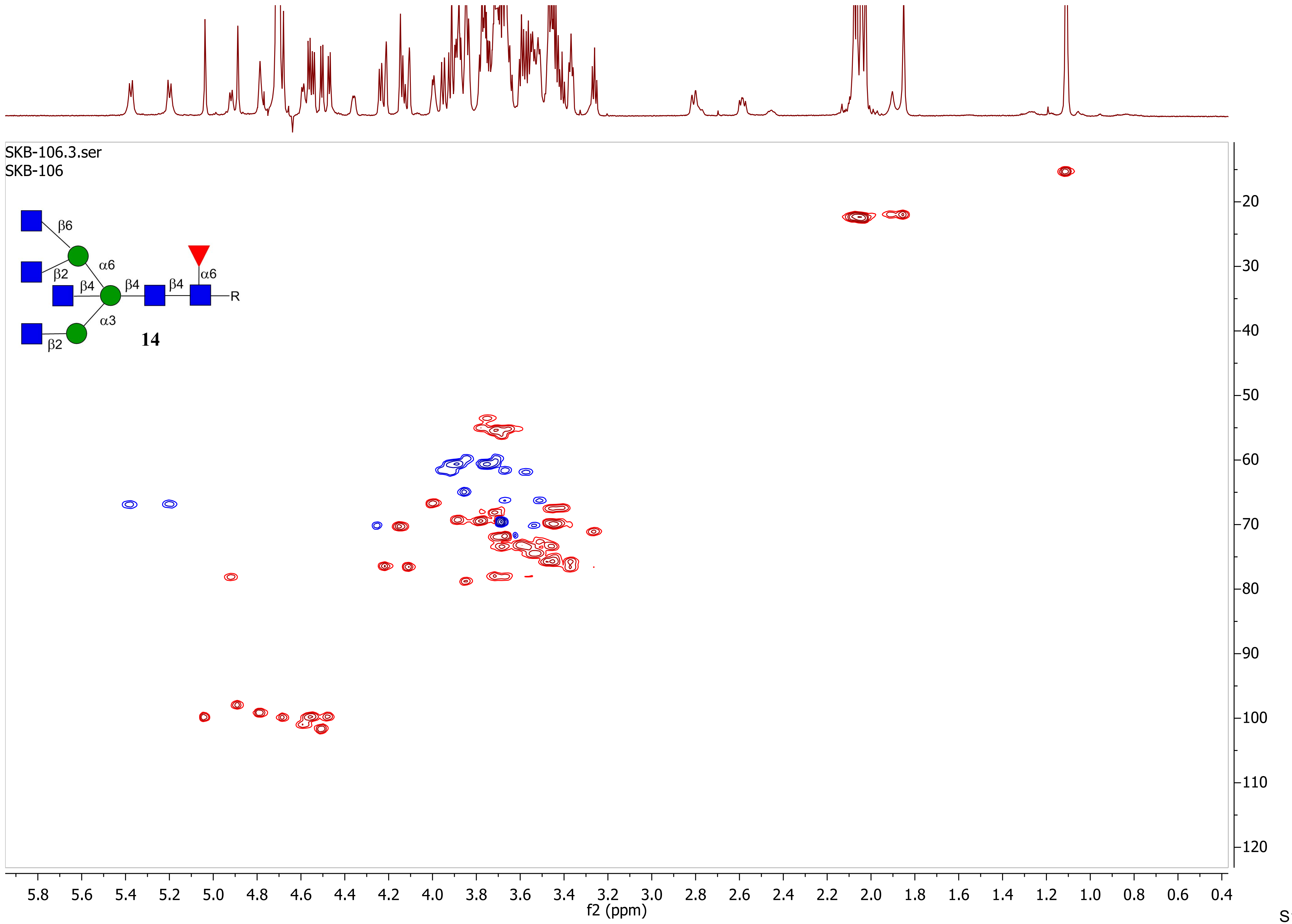
SKB-106.3.ser
SKB-106
β6
α6
β2
β4
β4
β4
α6
R
α3
β2
14
f2 (ppm)
f1 (ppm)

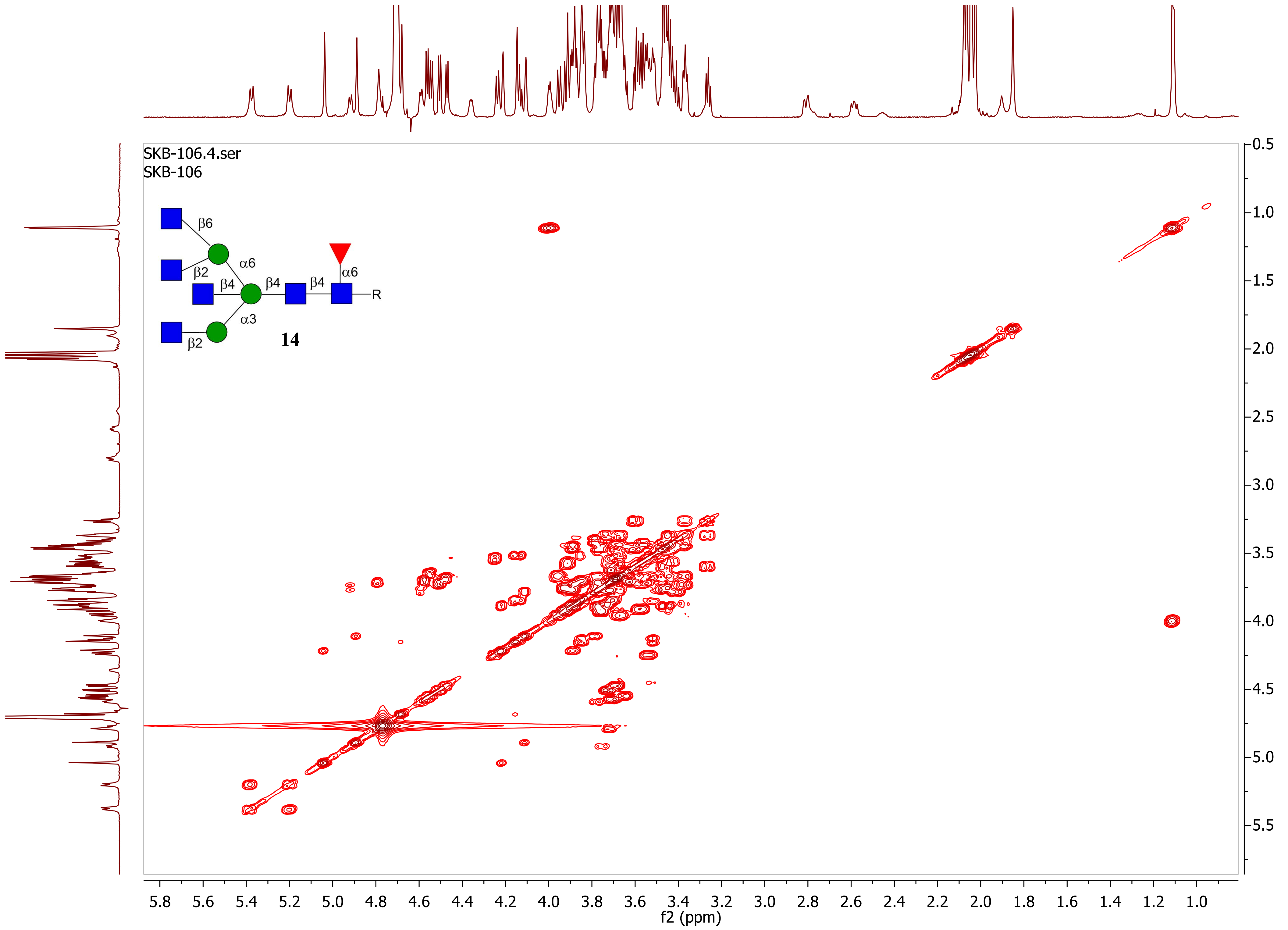

SKB-106.4.ser
SKB-106
β6
α6
β2
β4
β4
β4
α6
R
α3
β2
14
f2 (ppm)
f1 (ppm)

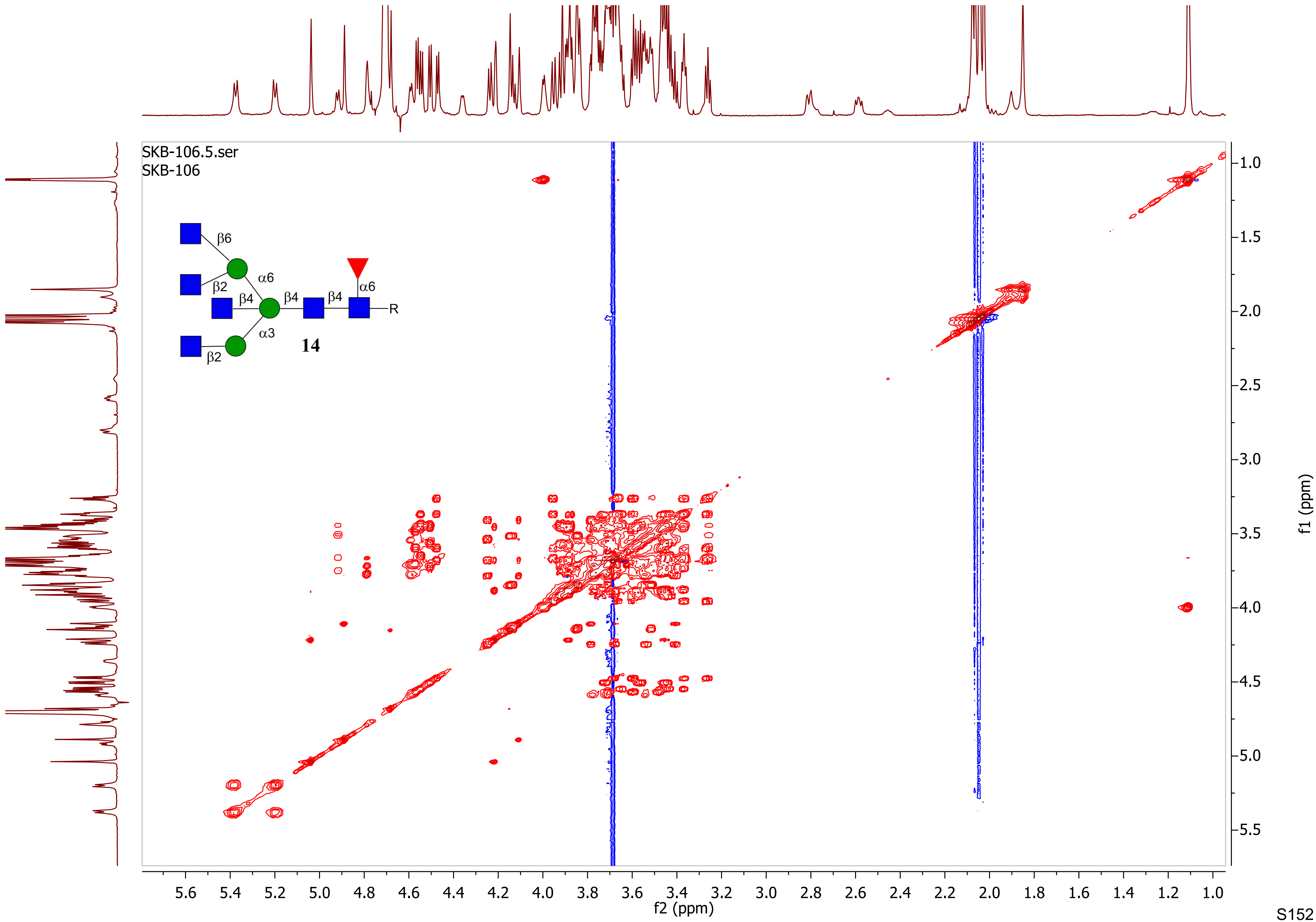
SKB-106.5.ser
SKB-106
β6
β2
α6
β4
β4
β4
α6
R
α3
β2
14
f1 (ppm)
f2 (ppm)

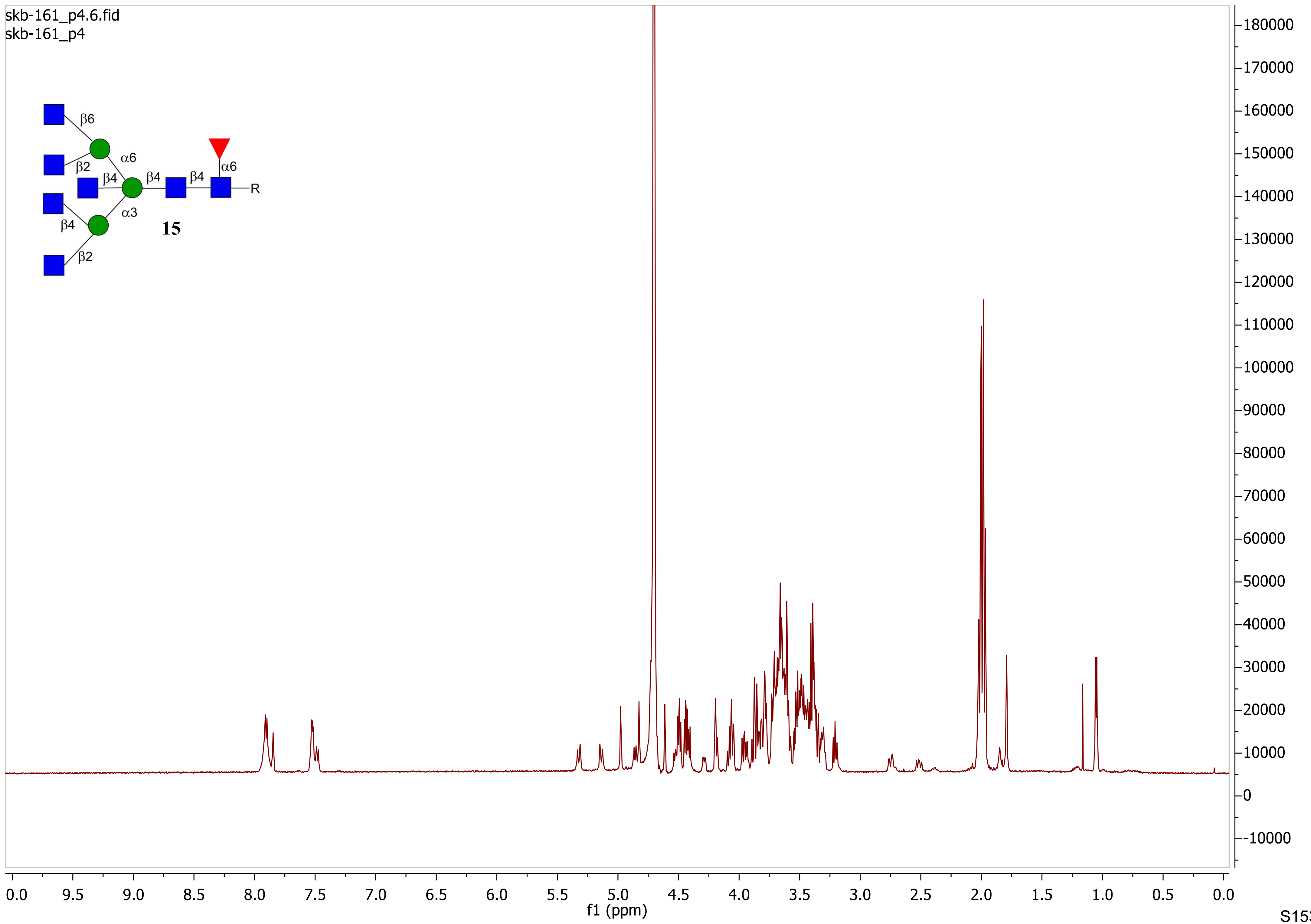
skb-161_p4.6.fid
skb-161_p4
β6
β2
α6
β4
β4
β4
α6
R
β4
α3
15
β2
f1 (ppm)
10.0 9.5 9.0 8.5 8.0 7.5 7.0 6.5 6.0 5.5 5.0 4.5 4.0 3.5 3.0 2.5 2.0 1.5 1.0 0.5 0.0
180000 170000 160000 150000 140000 130000 120000 110000 100000 90000 80000 70000 60000 50000 40000 30000 20000 10000 0 -10000

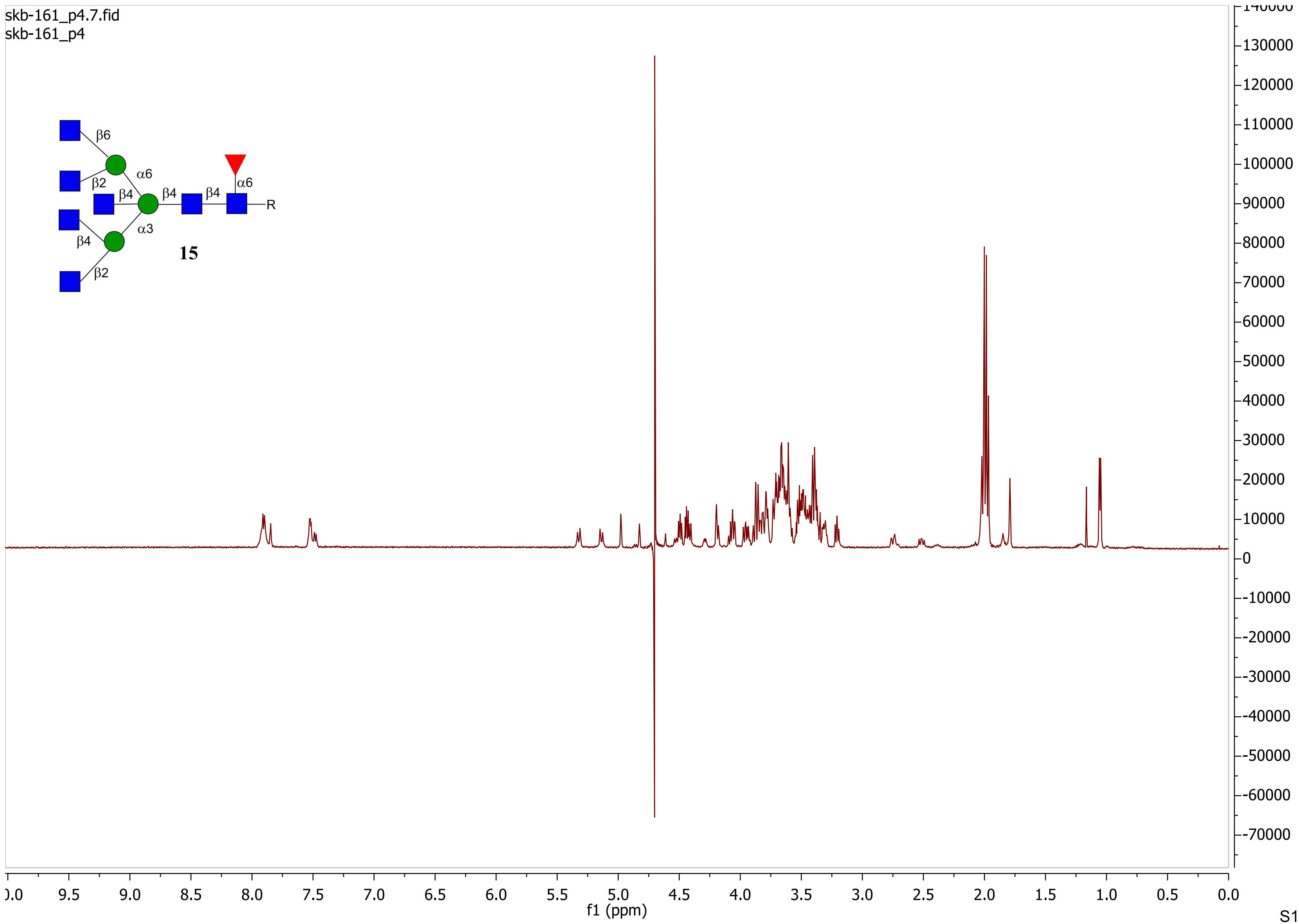
skb-161_p4.7.fid
skb-161_p4
β6
β2
α6
β4
β4
β4
α6
R
β4
α3
β2
15
140000
130000
120000
110000
100000
90000
80000
70000
60000
50000
40000
30000
20000
10000
0
-10000
-20000
-30000
-40000
-50000
-60000
-70000
10.0
9.5
9.0
8.5
8.0
7.5
7.0
6.5
6.0
5.5
5.0
4.5
4.0
3.5
3.0
2.5
2.0
1.5
1.0
0.5
0.0
f1 (ppm)

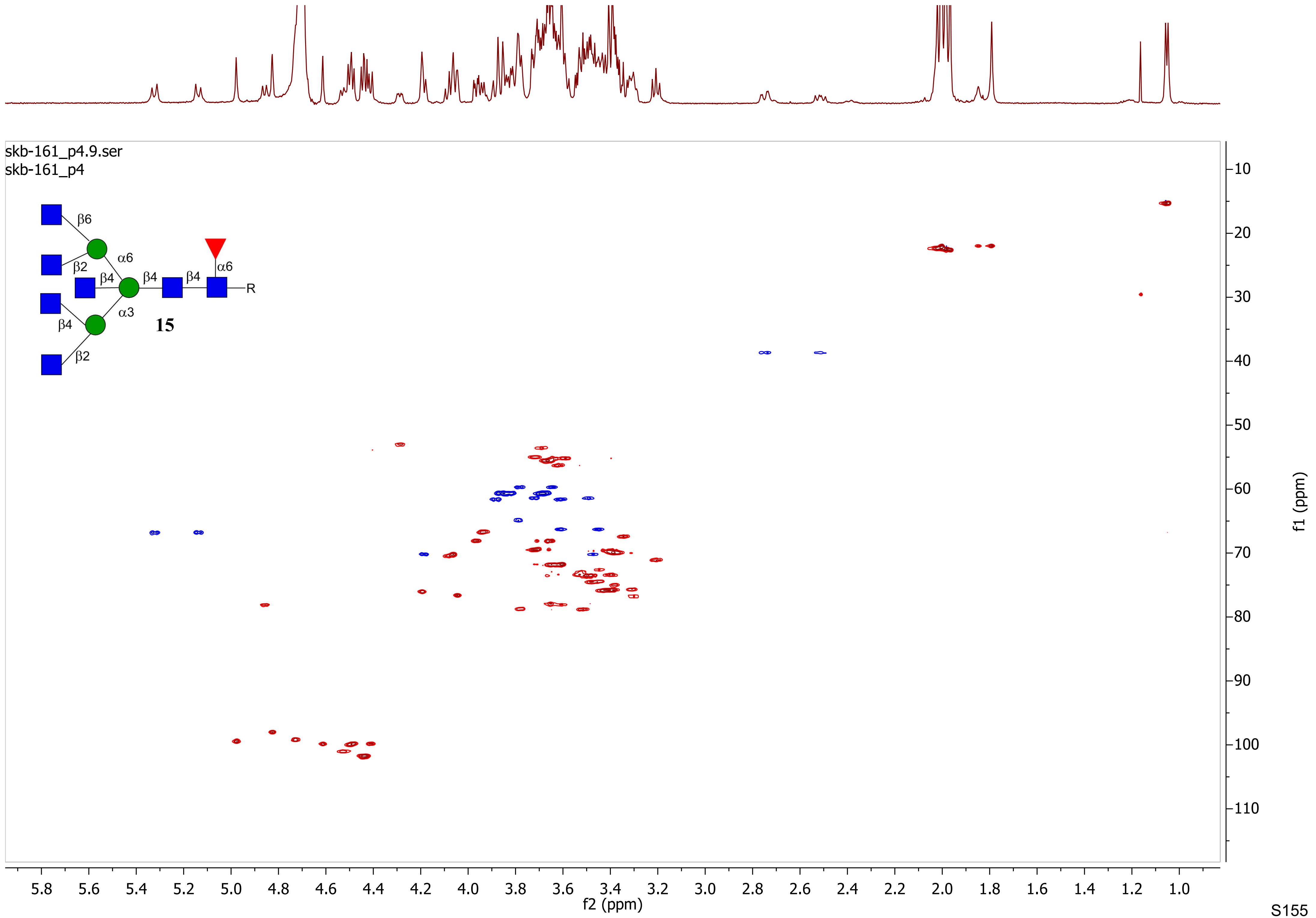
skb-161_p4.9.ser
skb-161_p4
β6
α6
β2
β4
β4
β4
α6
R
α3
β4
β2
15
f2 (ppm)
f1 (ppm)

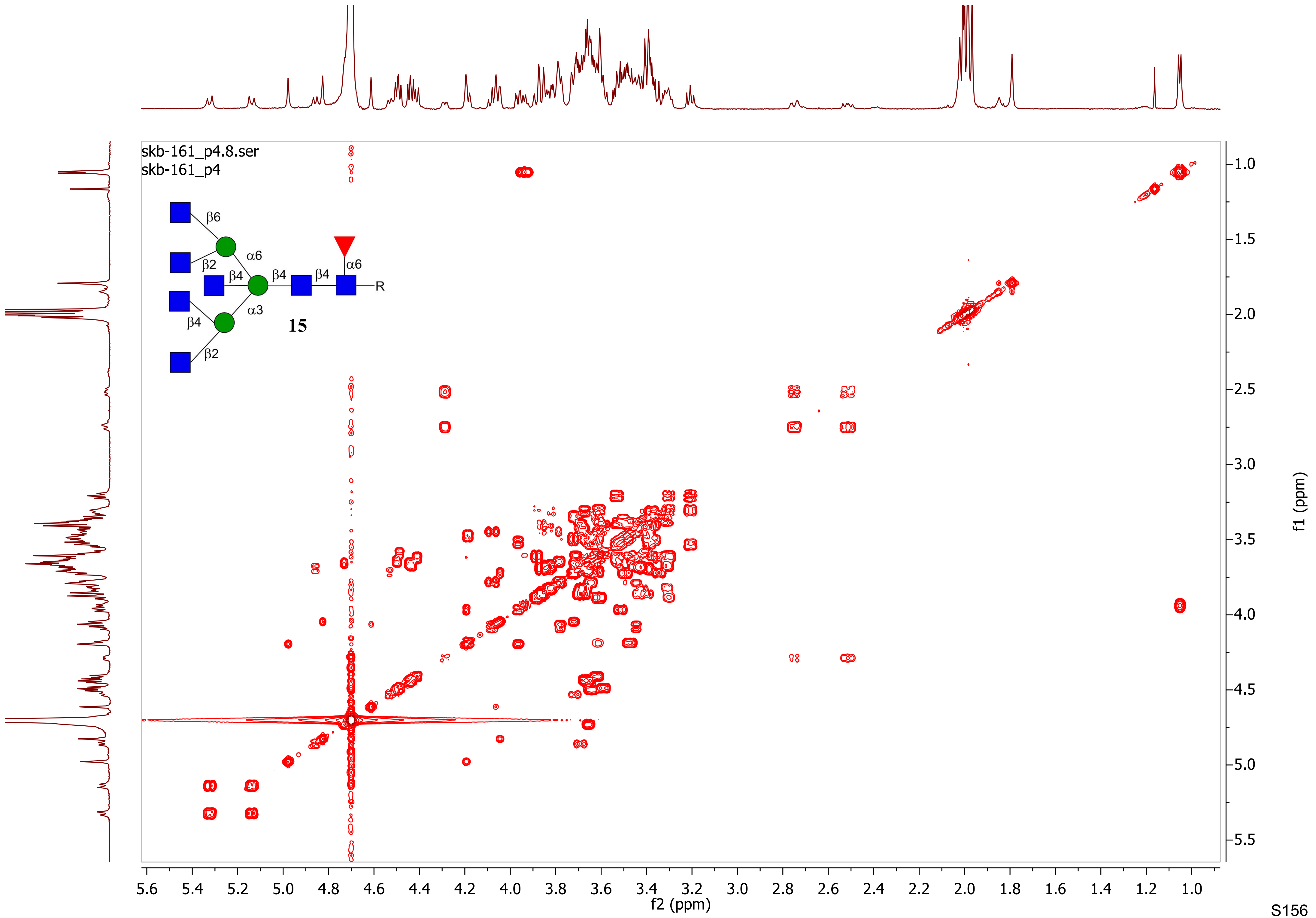

skb-161_p4.8.ser
skb-161_p4
β6
β2
α6
β4
β4
β4
α6
R
β4
α3
15
β2
f2 (ppm)
f1 (ppm)

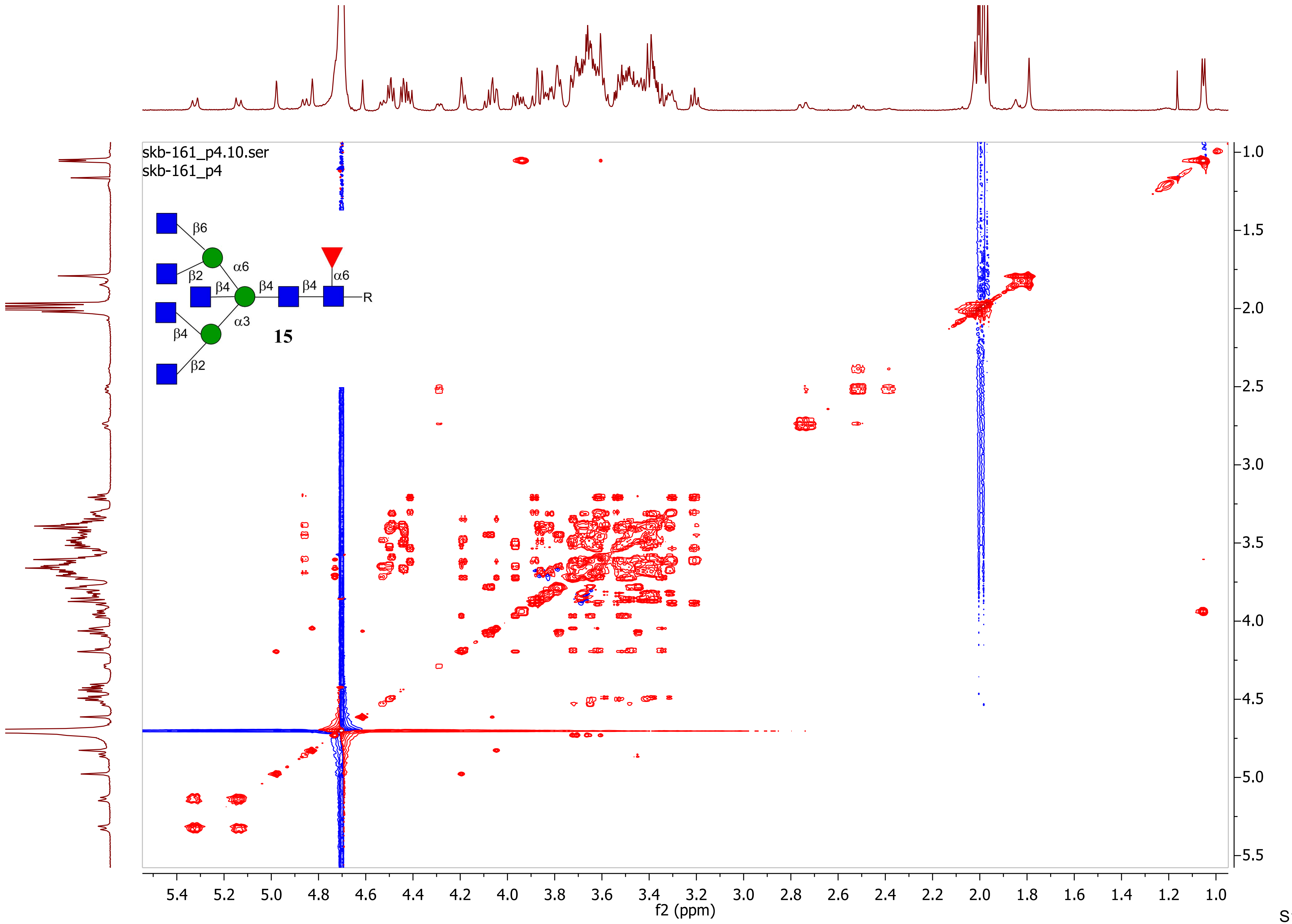

skb-161_p4.10.ser
skb-161_p4
β6
α6
β2
β4
β4
β4
α6
R
α3
15
β4
β2
f2 (ppm)
f1 (ppm)

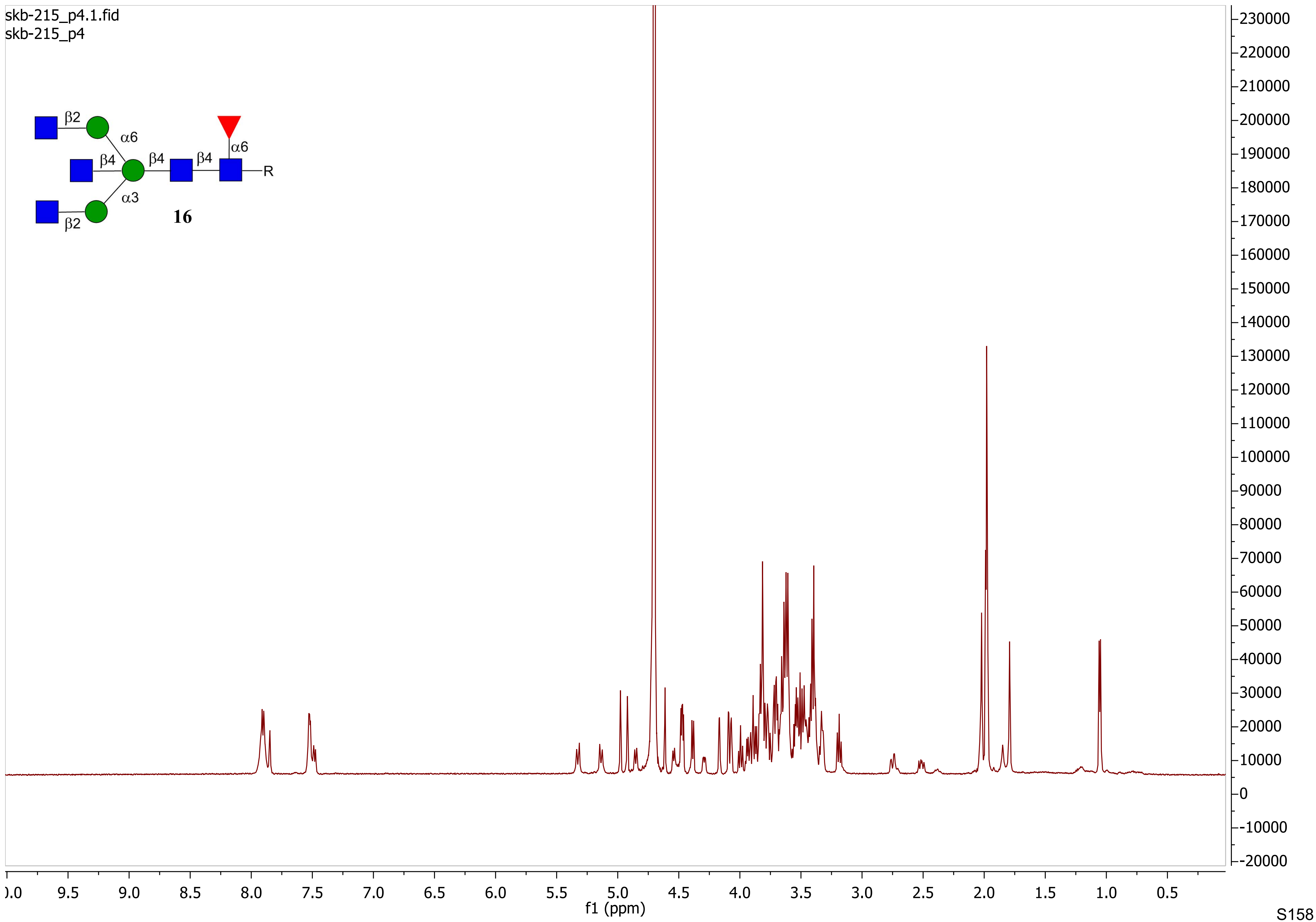
skb-215_p4.1.fid
skb-215_p4
β2
α6
β4
β4
β4
α6
R
α3
β2
16
f1 (ppm)

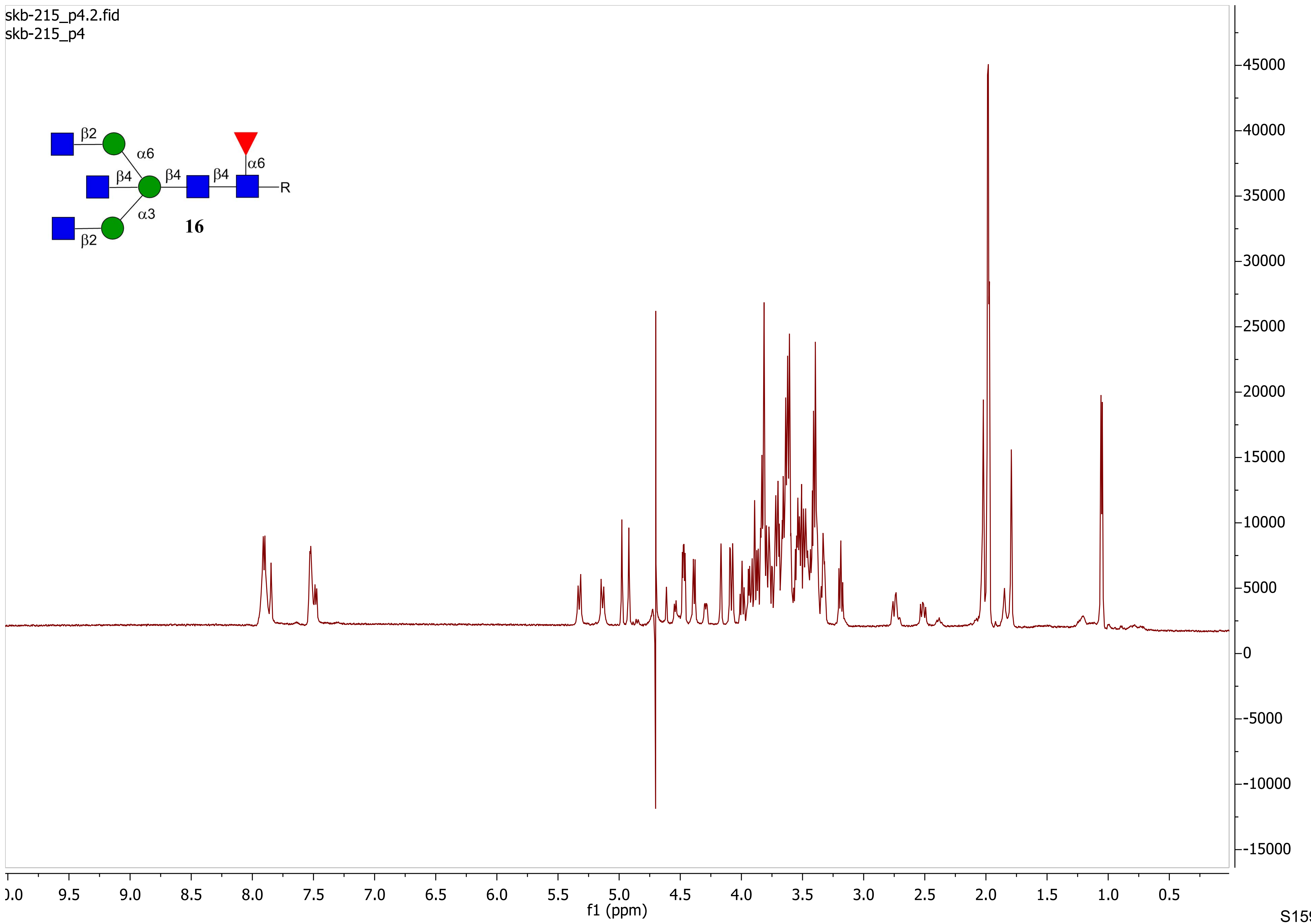
skb-215_p4.2.fid
skb-215_p4
β2
α6
β4
β4
β4
α6
R
α3
16
β2
45000
40000
35000
30000
25000
20000
15000
10000
5000
0
-5000
-10000
-15000
9.5
9.0
8.5
8.0
7.5
7.0
6.5
6.0
5.5
5.0
4.5
4.0
3.5
3.0
2.5
2.0
1.5
1.0
0.5
f1 (ppm)

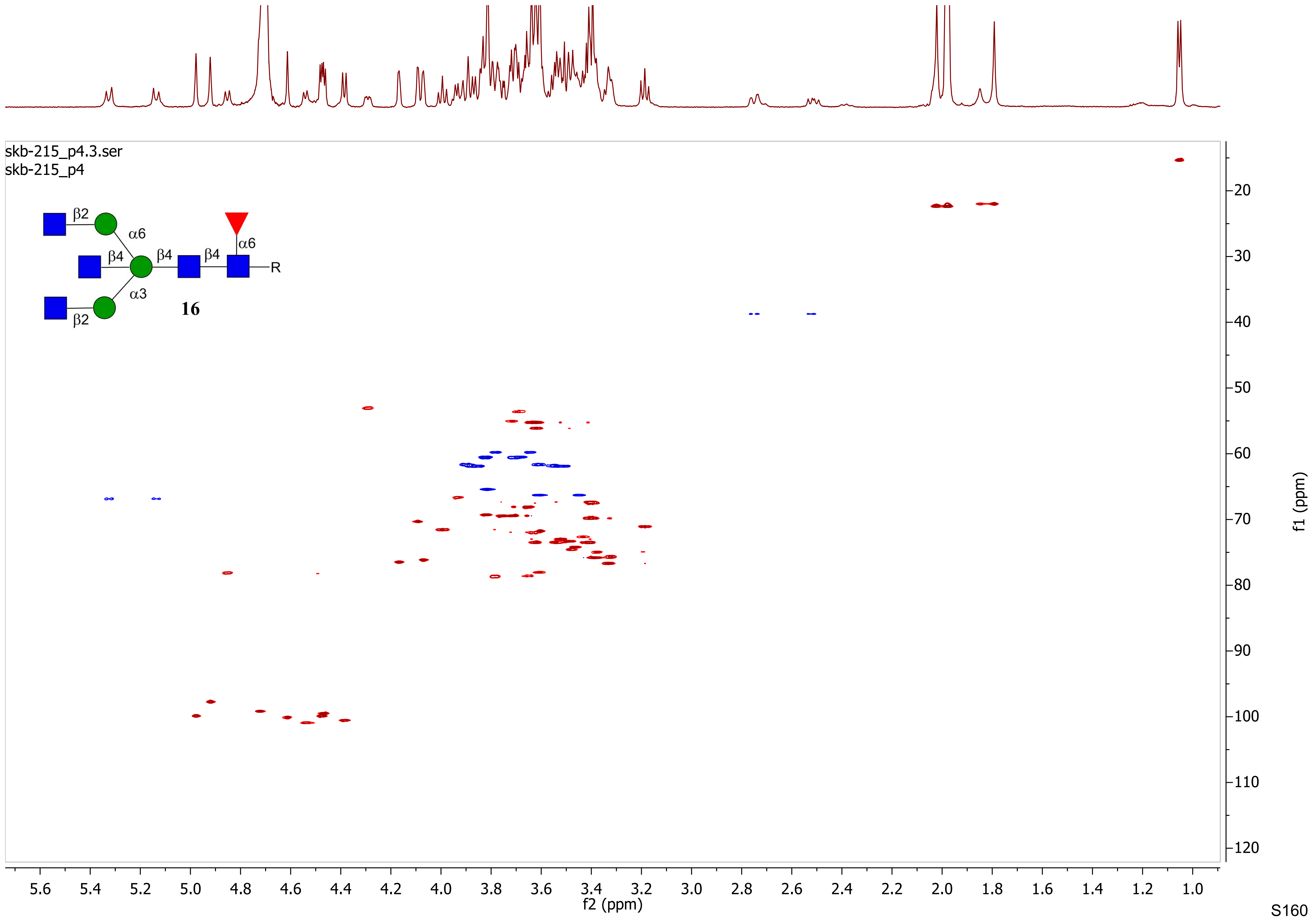
skb-215_p4.3.ser
skb-215_p4
β2
α6
β4
β4
β4
α6
R
α3
β2
16
f1 (ppm)
f2 (ppm)

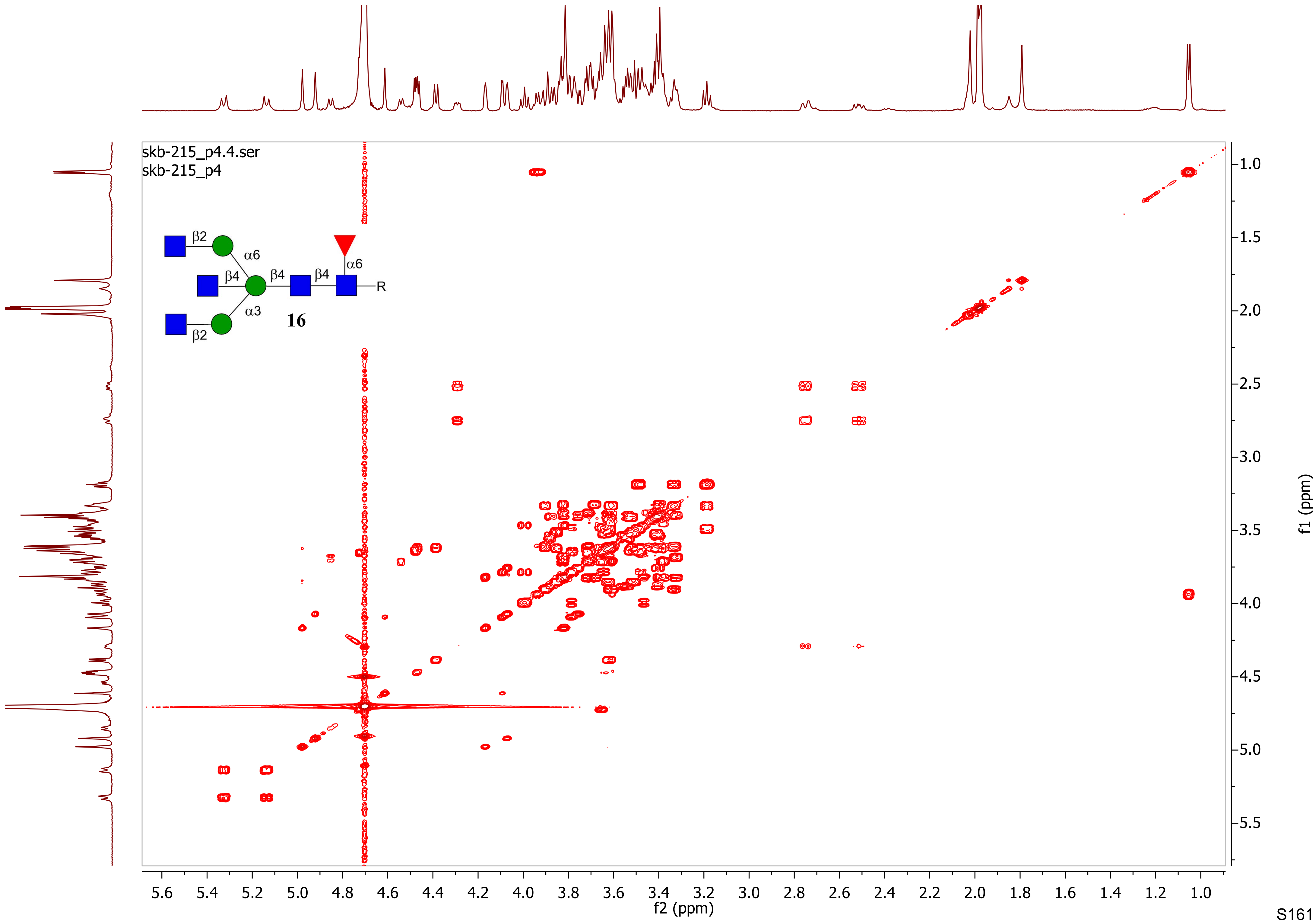
skb-215_p4.4.ser
skb-215_p4
β2
α6
β4
β4
β4
α6
R
α3
16
β2
f2 (ppm)
f1 (ppm)

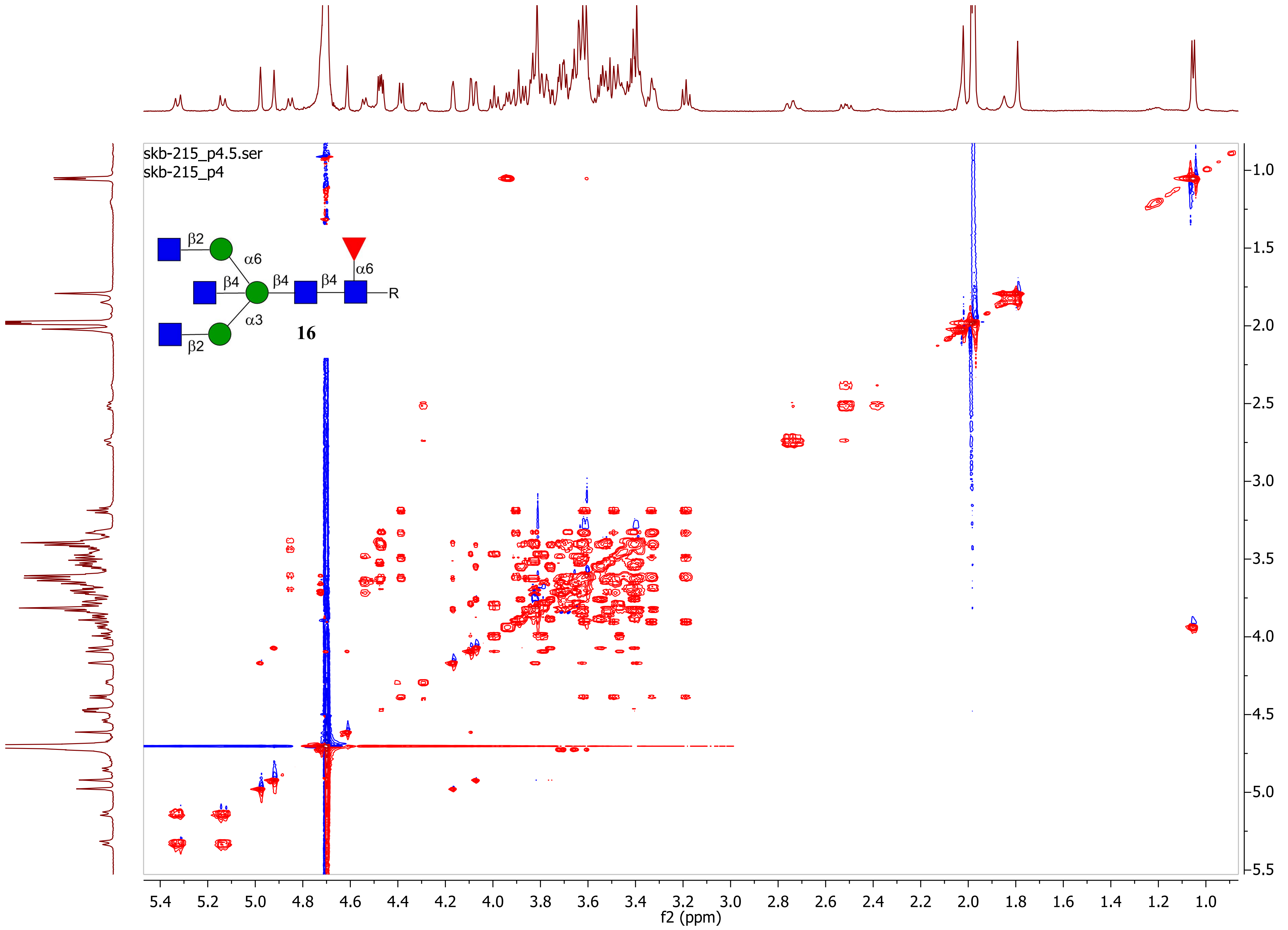

skb-215_p4.5.ser
skb-215_p4
β2
α6
β4
β4
β4
α6
R
α3
β2
16
f2 (ppm)
f1 (ppm)

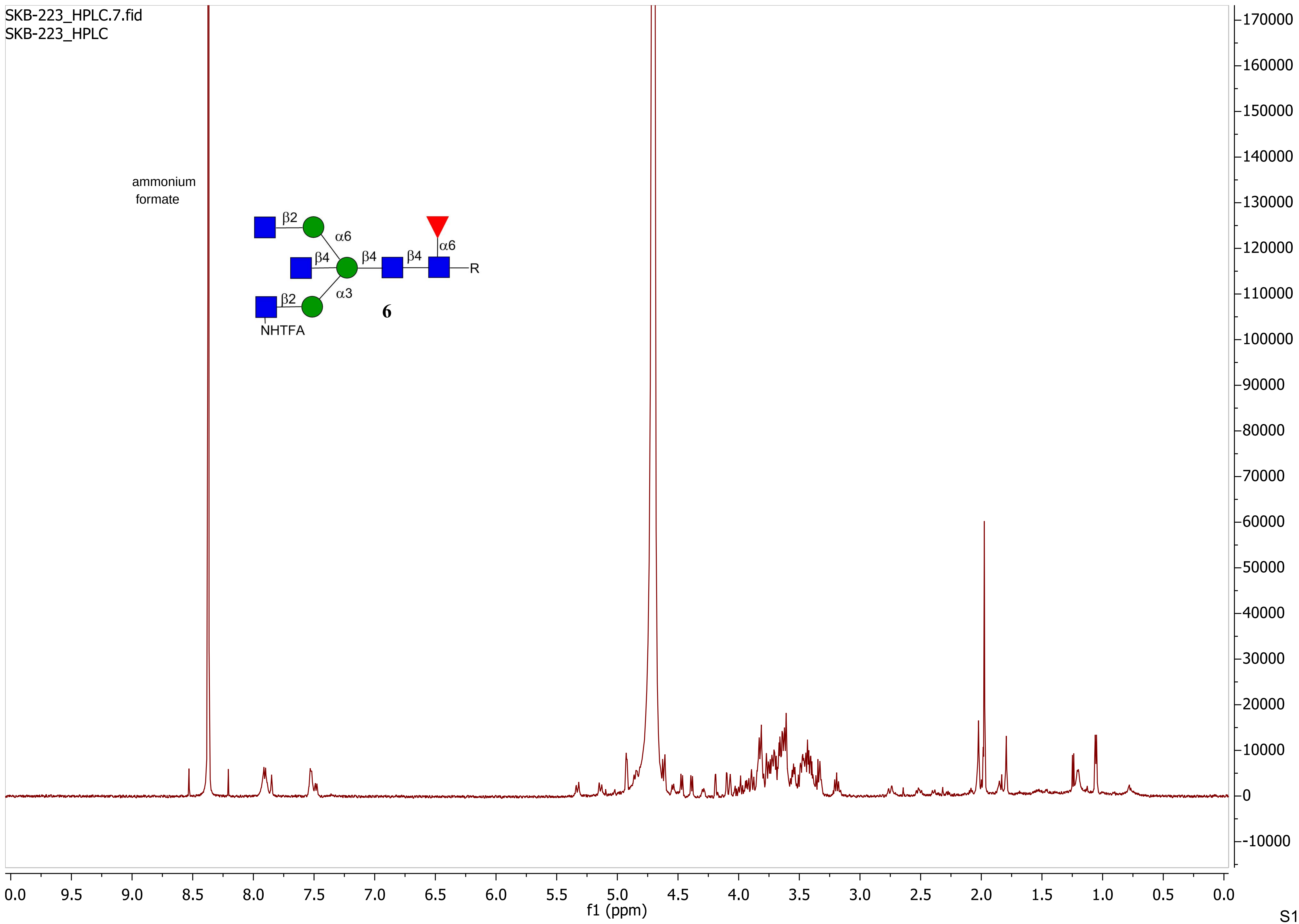
SKB-223_HPLC.7.fid
SKB-223_HPLC
ammonium formate
β2
α6
β4
β4
β4
α6
R
β2
α3
6
NHTFA
f1 (ppm)

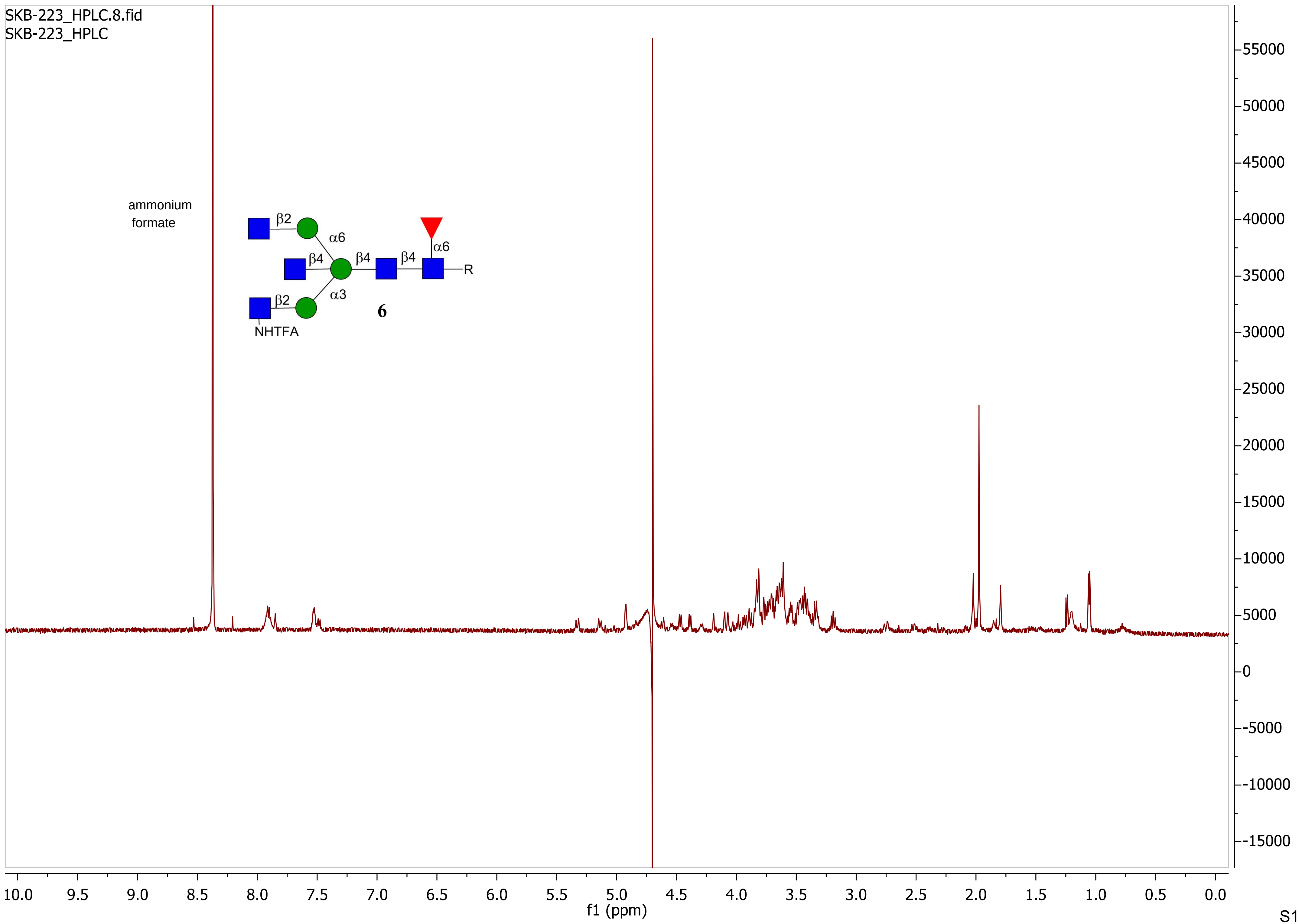

SKB-223_HPLC.8.fid
SKB-223_HPLC
ammonium formate
β2
α6
β4
β4
β4
α6
R
α3
β2
NHTFA
6
f1 (ppm)

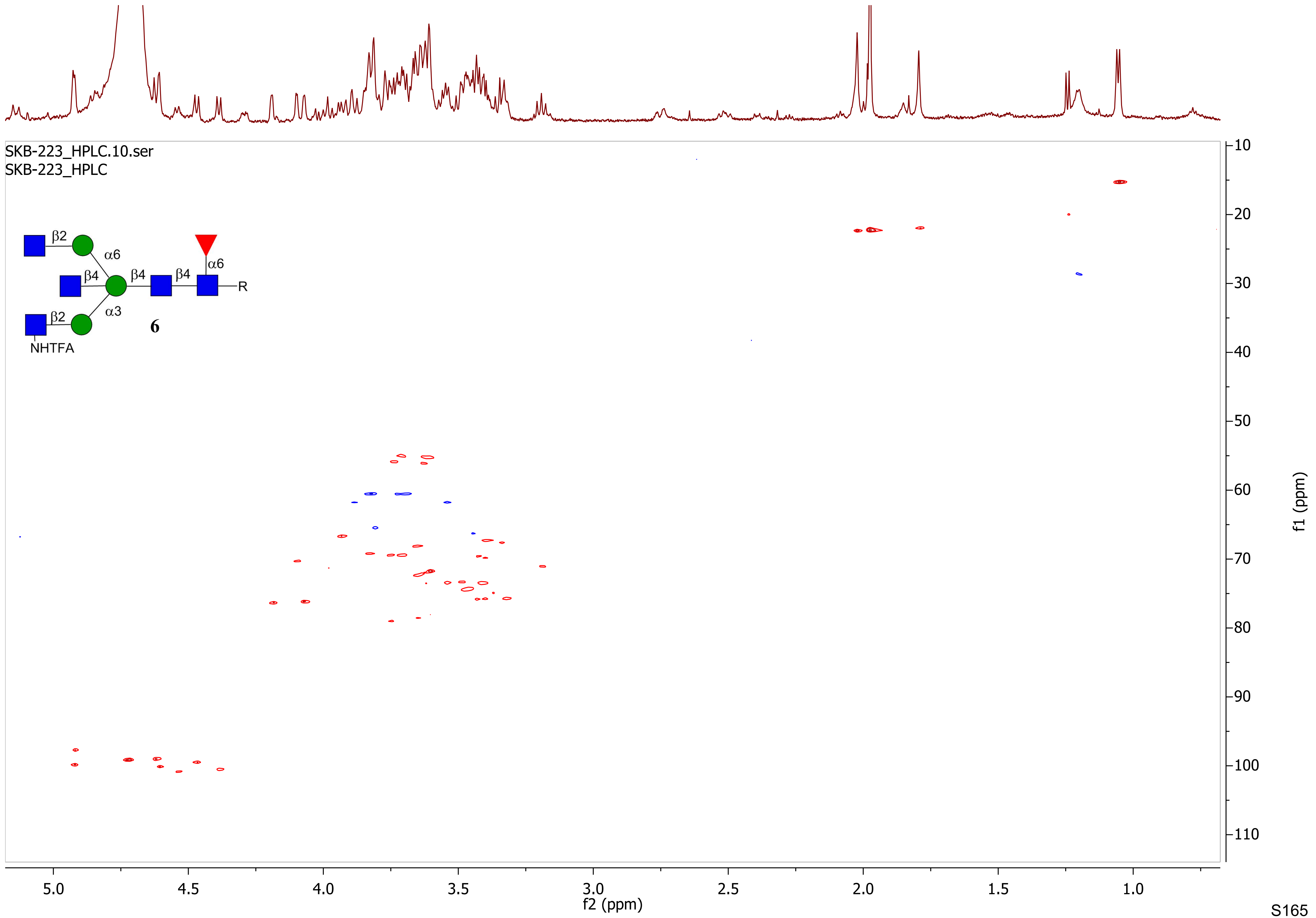
SKB-223_HPLC.10.ser
SKB-223_HPLC
β2
α6
β4
β4
β4
α6
R
α3
β2
6
NHTFA
f1 (ppm)
f2 (ppm)
5.0
4.5
4.0
3.5
3.0
2.5
2.0
1.5
1.0
10
20
30
40
50
60
70
80
90
100
110

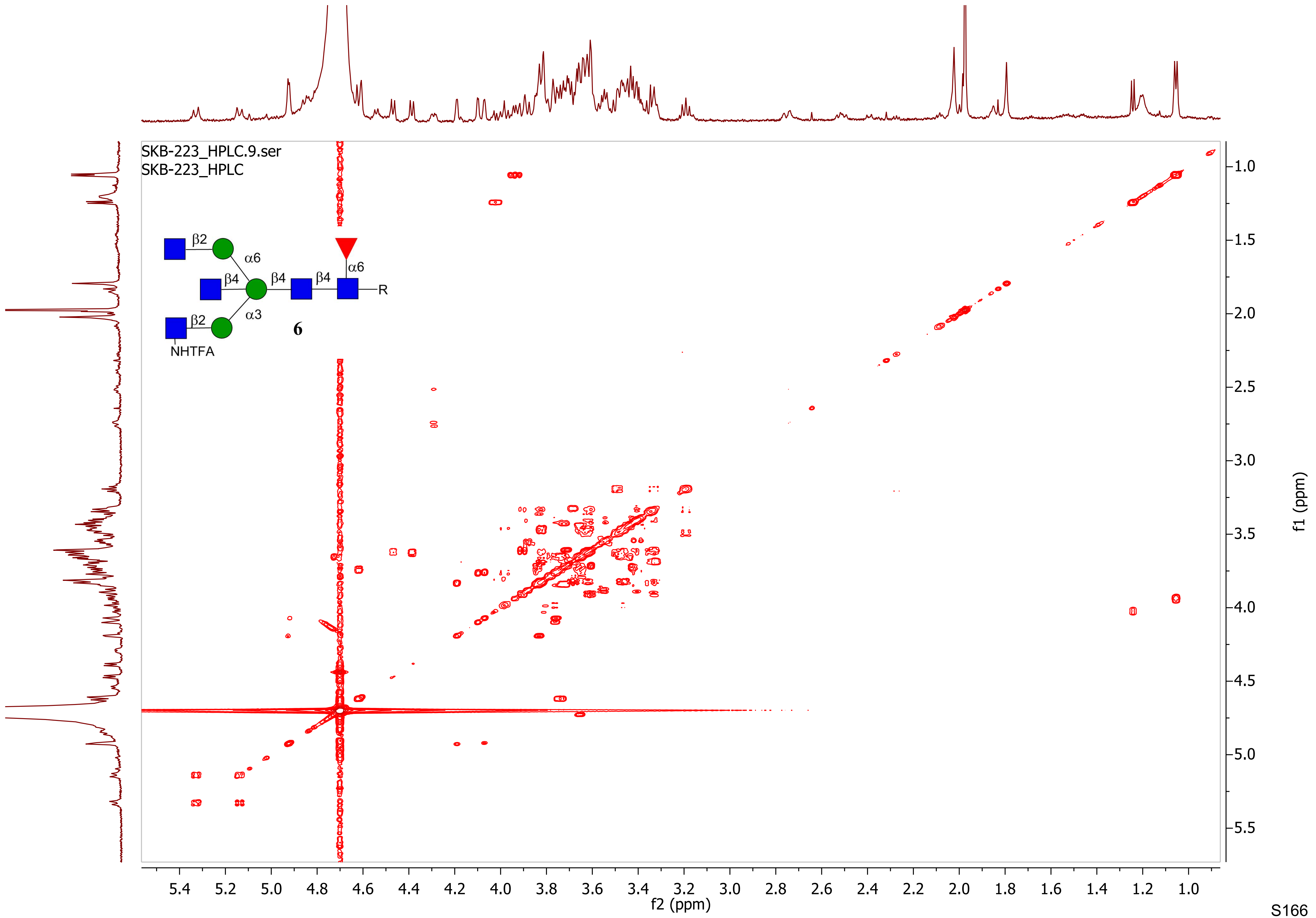
SKB-223_HPLC.9.ser
SKB-223_HPLC
β2
α6
β4
β4
β4
α6
R
β2
α3
NHTFA
6
f2 (ppm)
f1 (ppm)

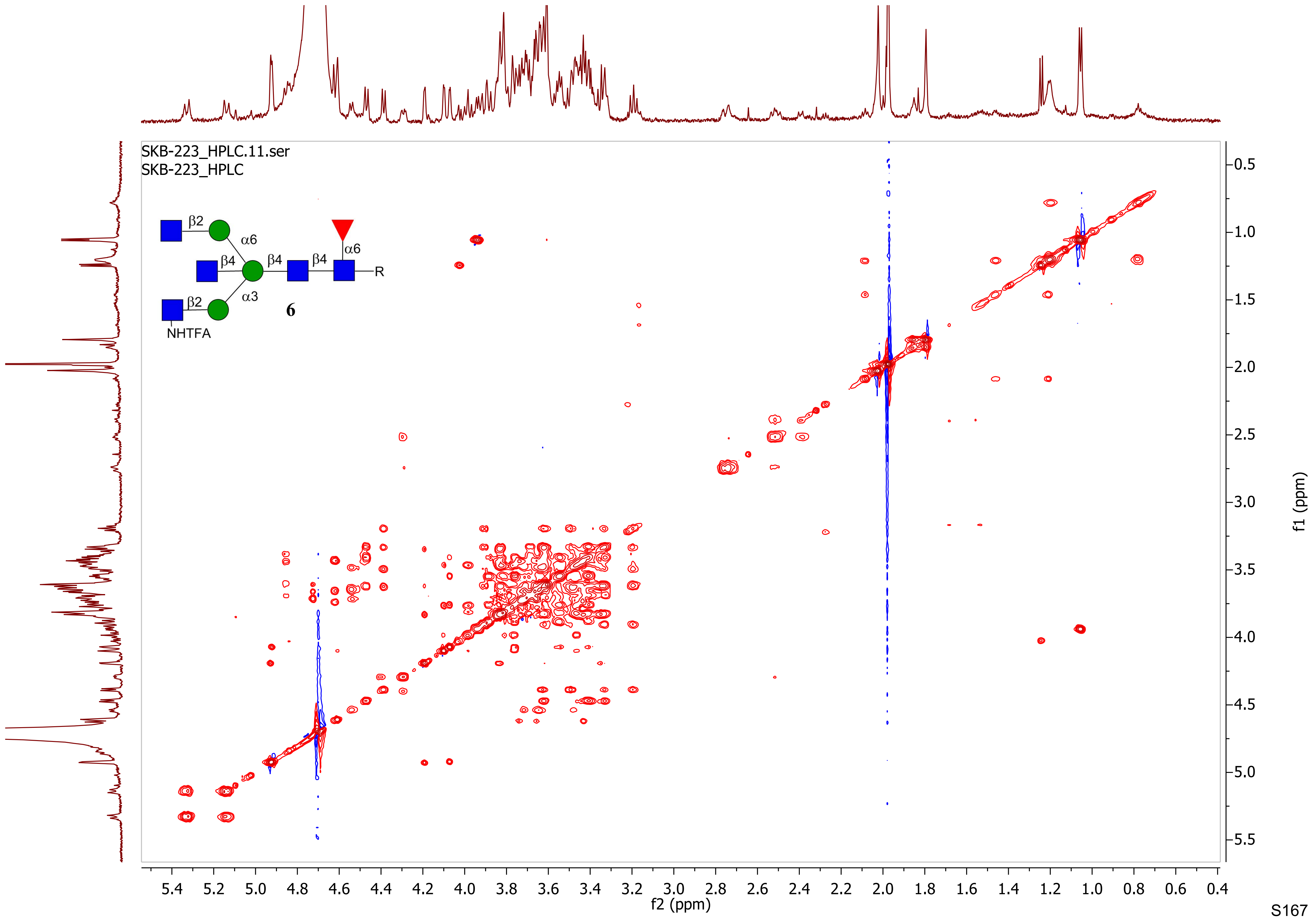
SKB-223_HPLC.11.ser
SKB-223_HPLC
β2
α6
β4
β4
β4
α6
R
α3
β2
6
NHTFA
f2 (ppm)
f1 (ppm)

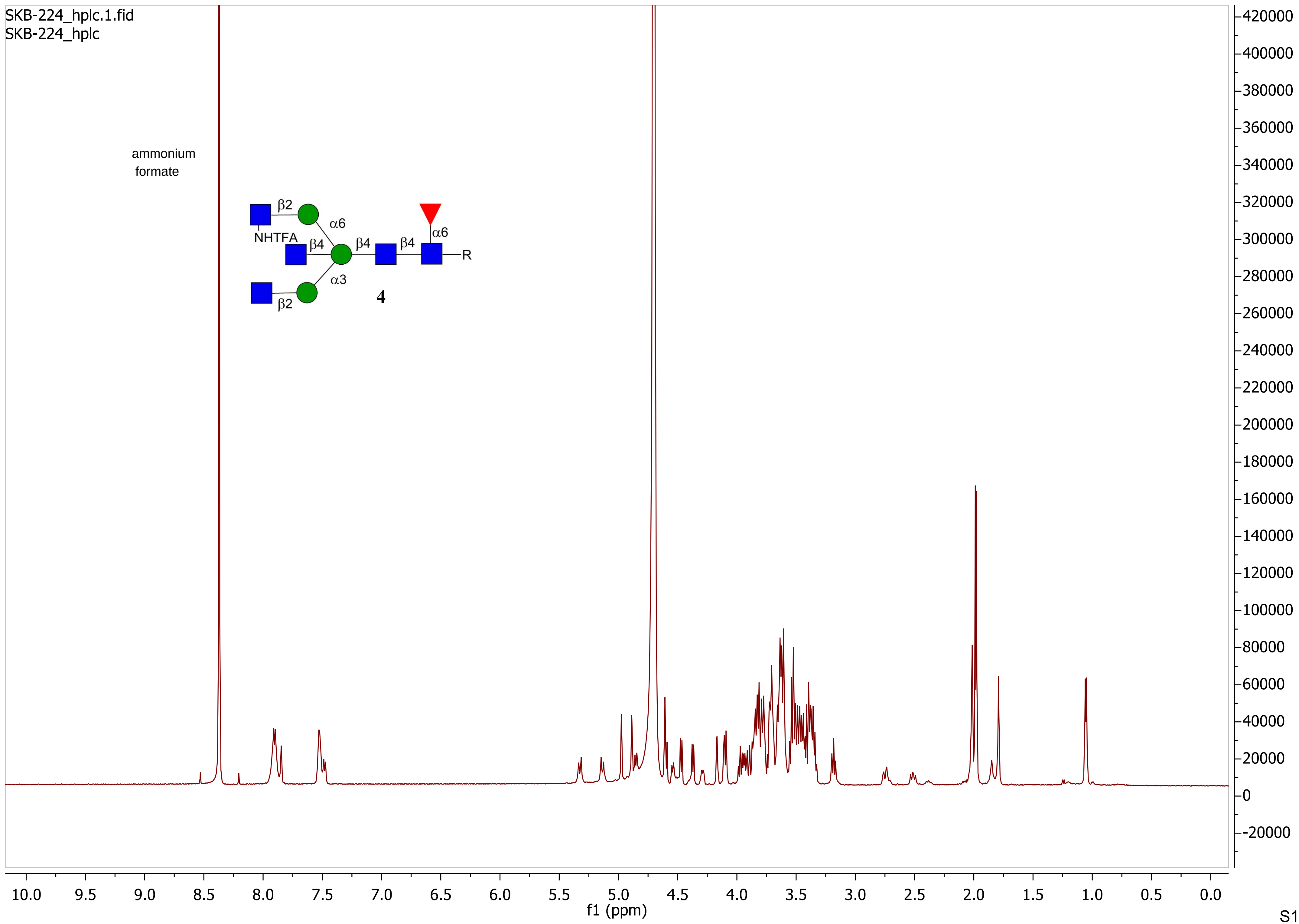

SKB-224_hplc.1.fid
SKB-224_hplc
ammonium
formate
β2
α6
NHTFA
β4
β4
β4
α6
R
α3
β2
4
420000
400000
380000
360000
340000
320000
300000
280000
260000
240000
220000
200000
180000
160000
140000
120000
100000
80000
60000
40000
20000
0
-20000
10.0 9.5 9.0 8.5 8.0 7.5 7.0 6.5 6.0 5.5 5.0 4.5 4.0 3.5 3.0 2.5 2.0 1.5 1.0 0.5 0.0
f1 (ppm)

SKB-224_hplc.2.fid
SKB-224_hplc

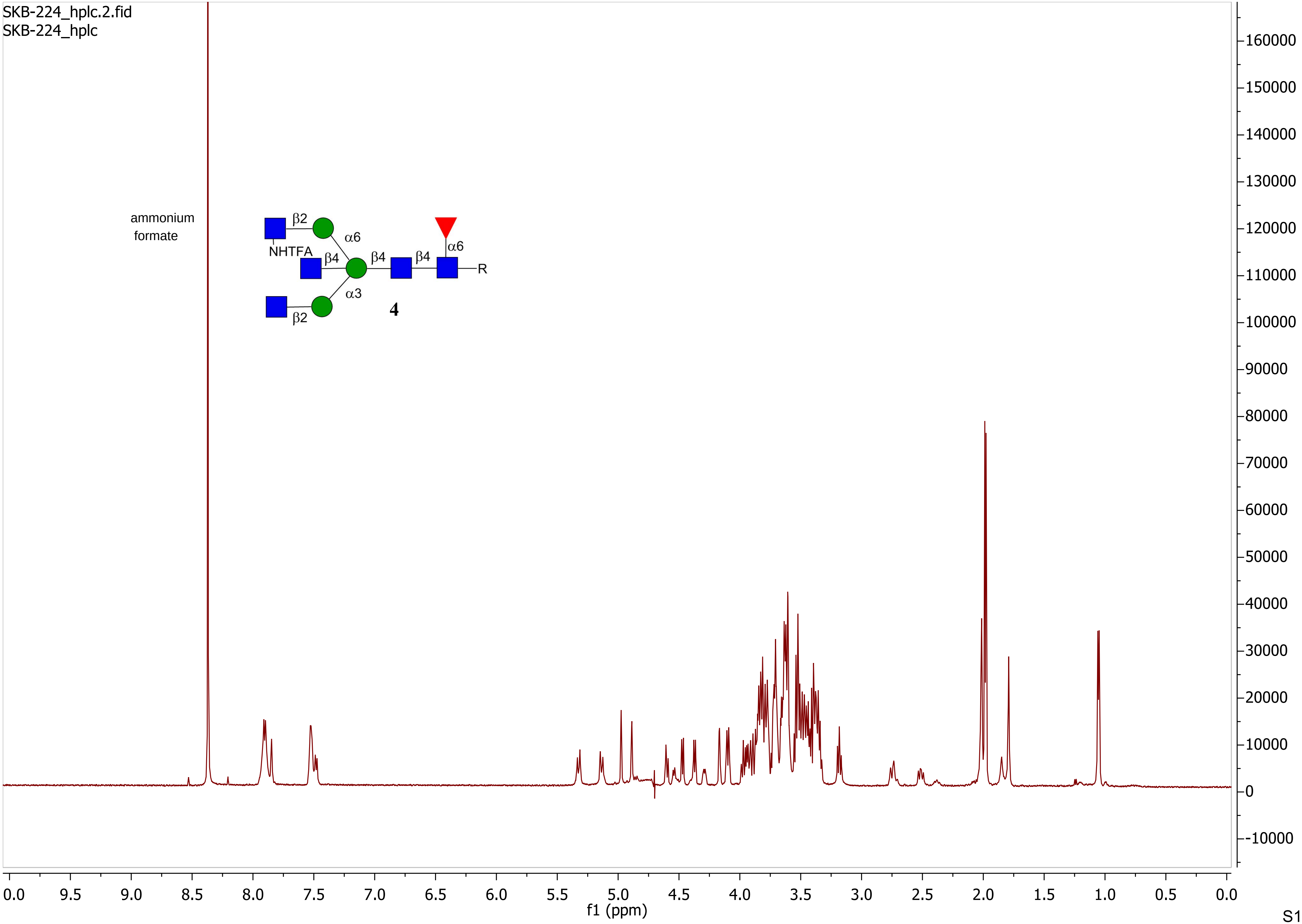

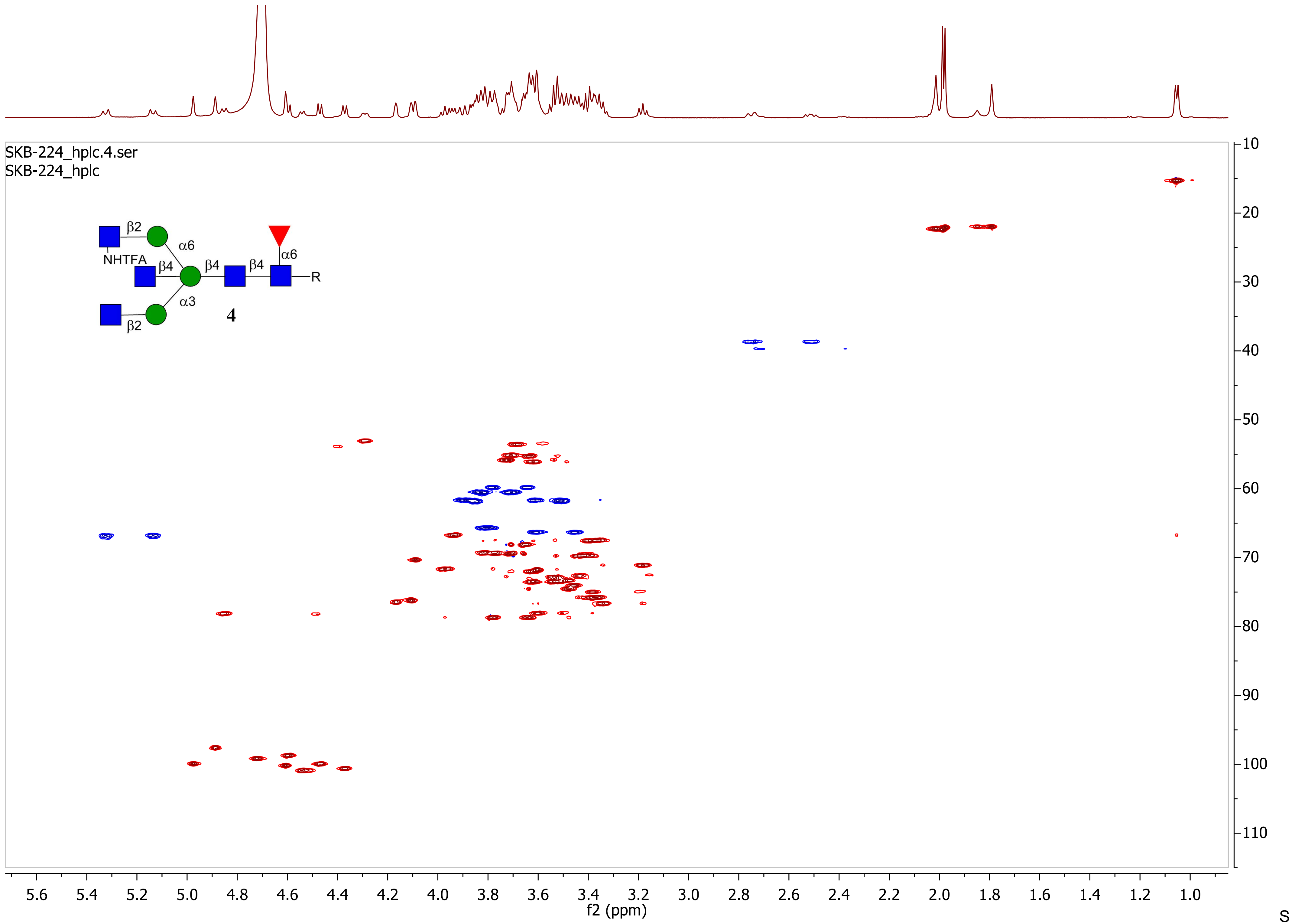
SKB-224_hplc.4.ser
SKB-224_hplc
β2
α6
NHTFA
β4
β4
β4
α6
R
α3
β2
4
f2 (ppm)
f1 (ppm)

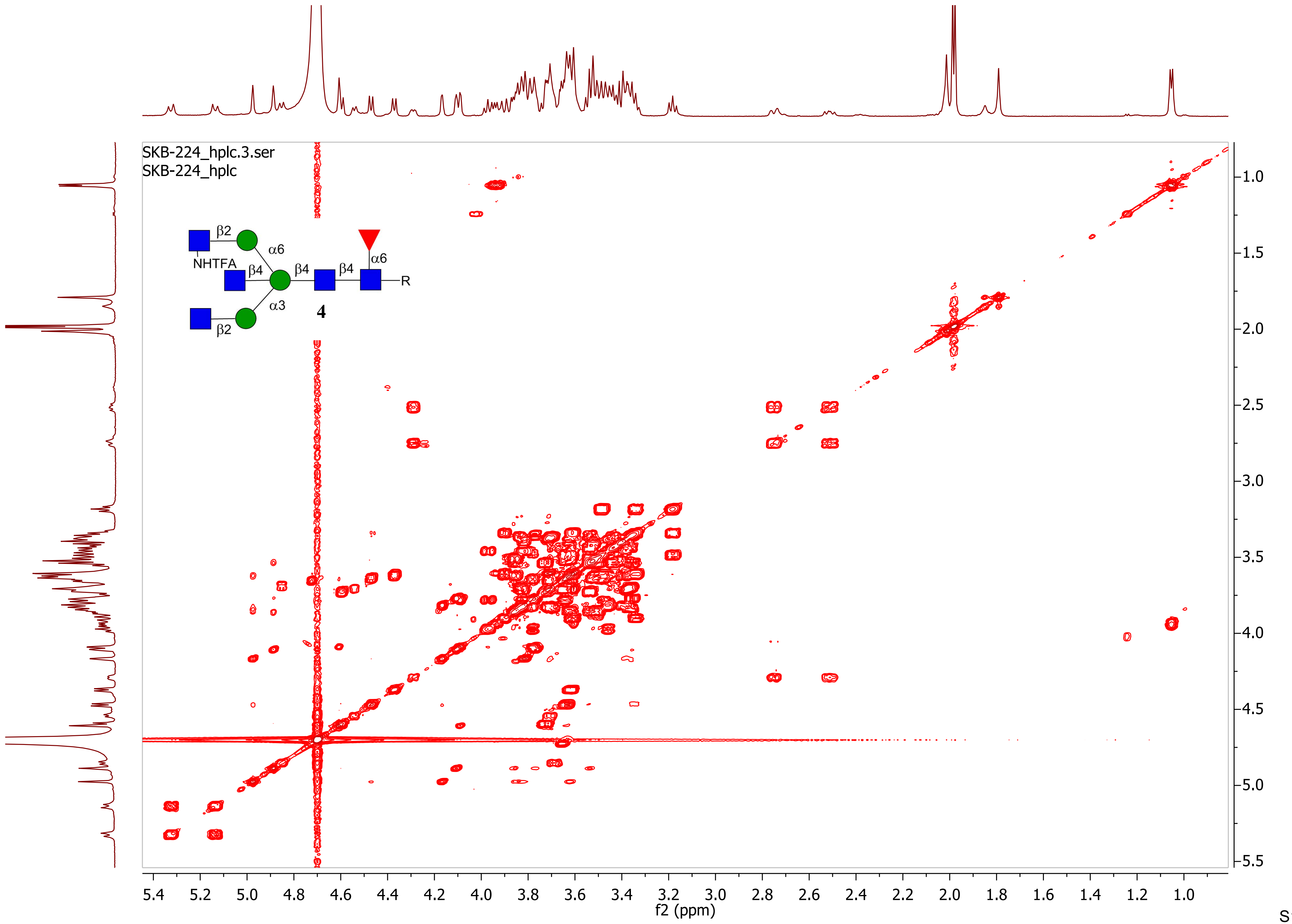

SKB-224_hplc.3.ser
SKB-224_hplc
β2
α6
NHTFA
β4
β4
β4
α6
R
α3
4
β2
f2 (ppm)
f1 (ppm)

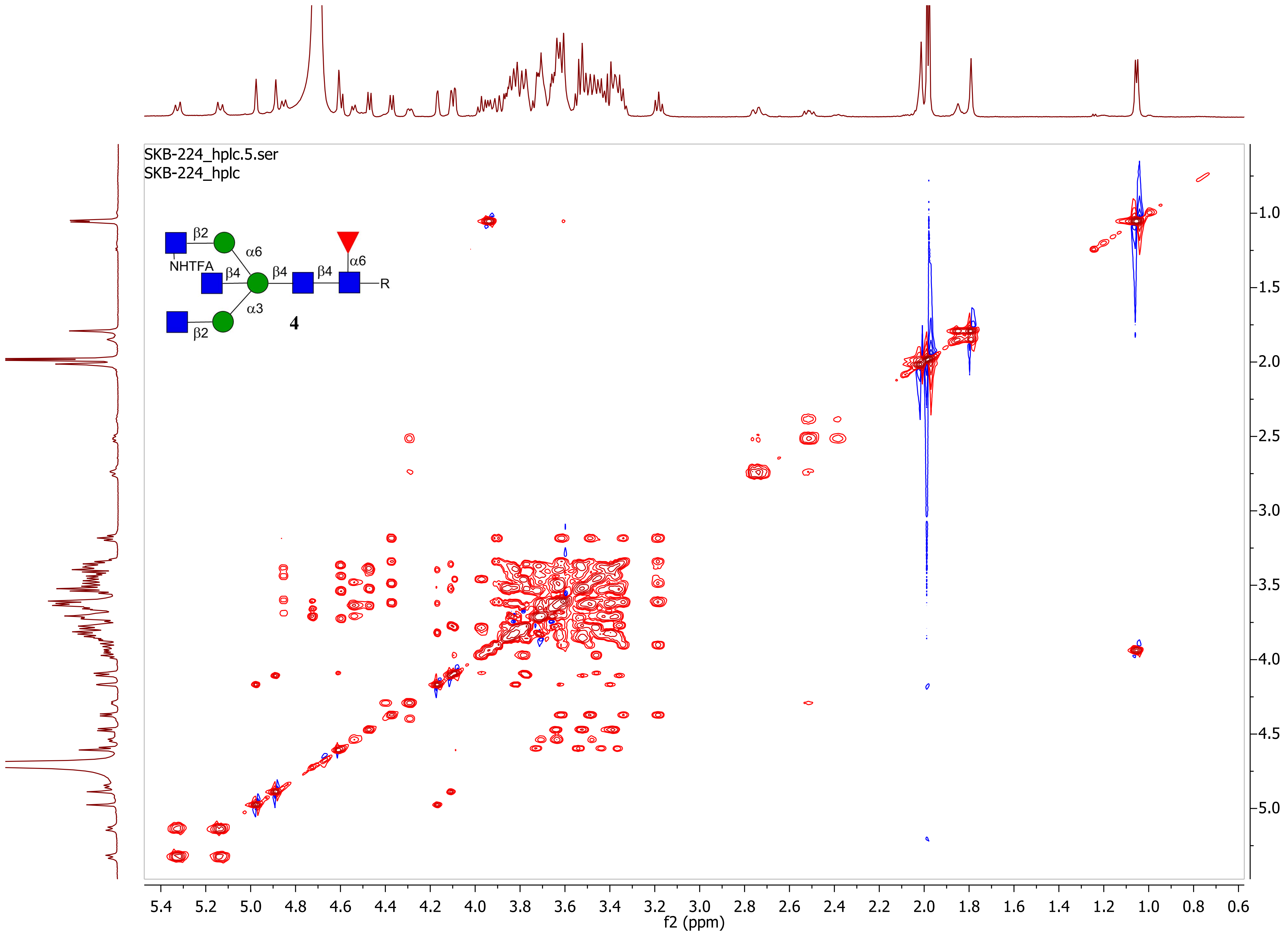
SKB-224_hplc.5.ser
SKB-224_hplc
β2
α6
NHTFA
β4
β4
β4
α6
R
α3
β2
4
f2 (ppm)
f1 (ppm)

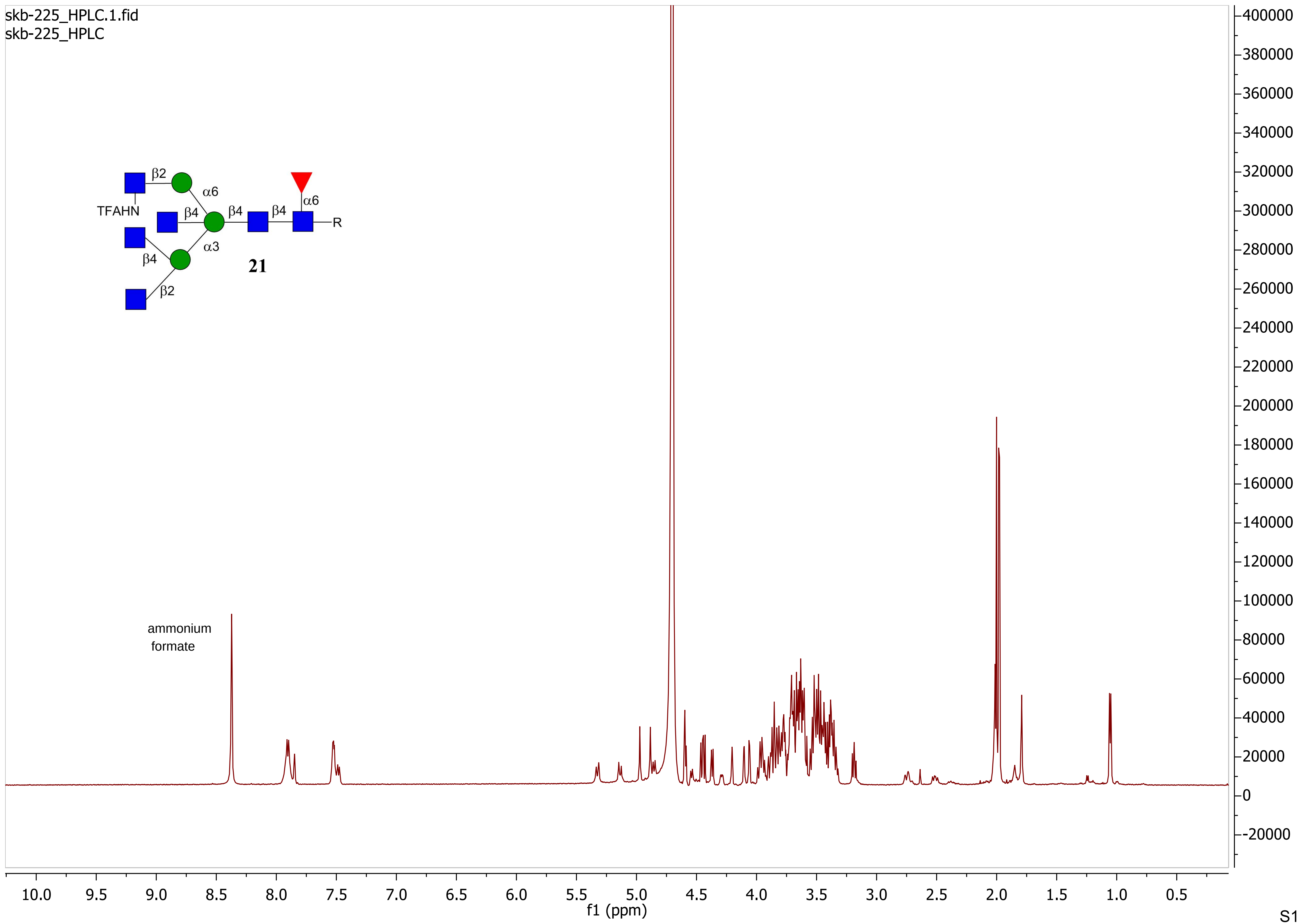

skb-225_HPLC.1.fid
skb-225_HPLC
TFAHN
β2
α6
β4
β4
β4
α6
R
α3
β4
β2
21
ammonium formate
f1 (ppm)

skb-225_HPLC.2.fid
skb-225_HPLC

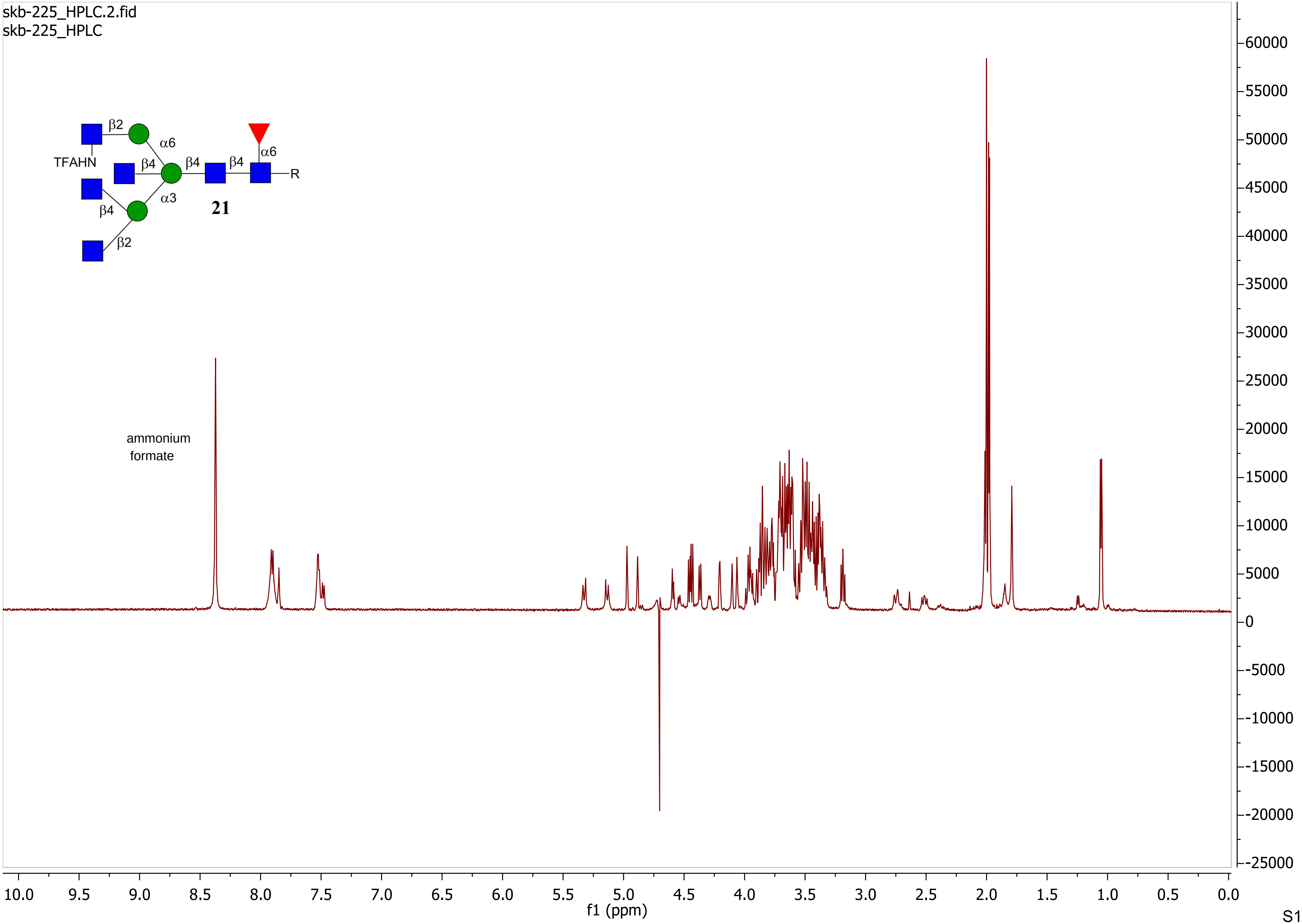

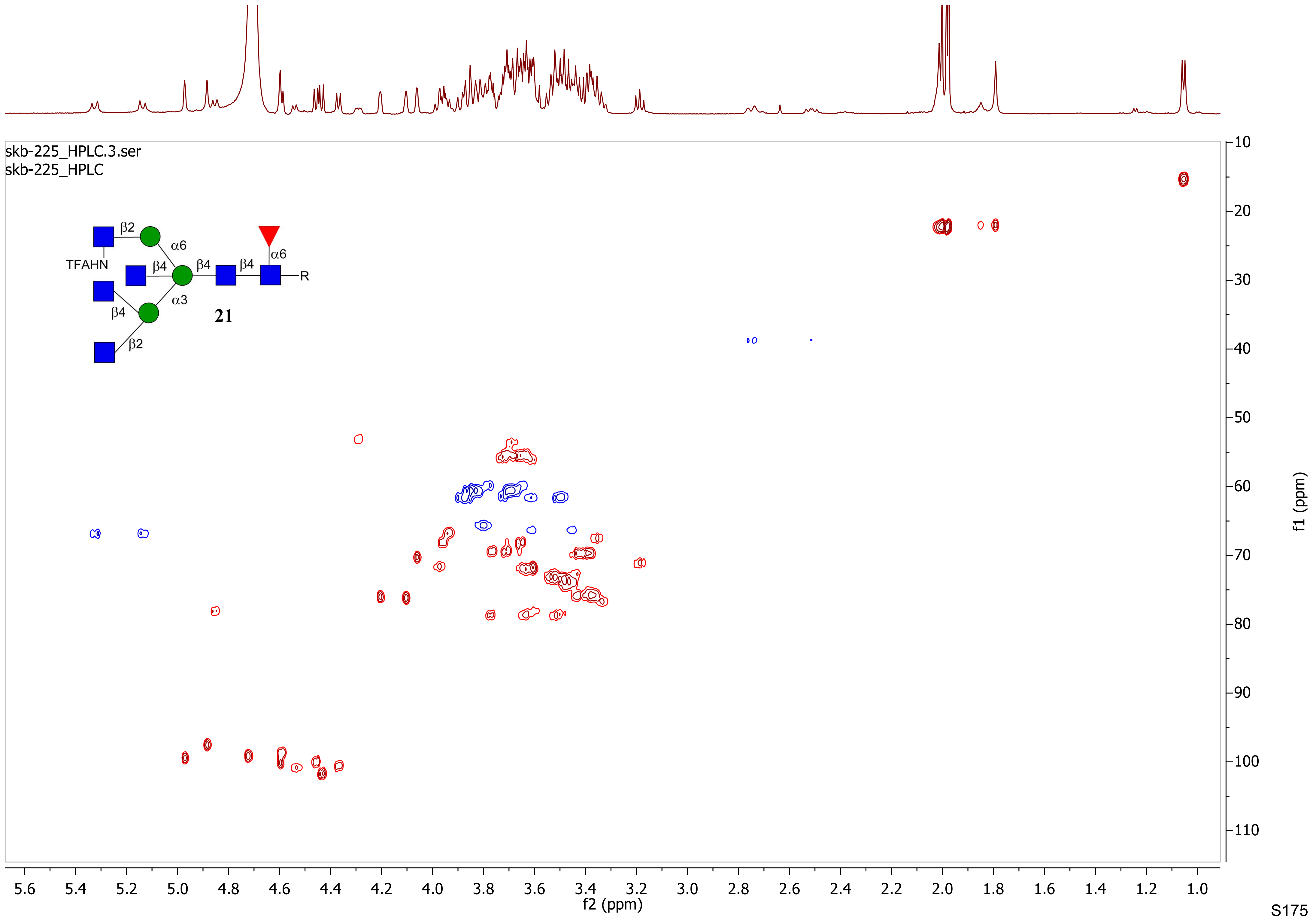

skb-225_HPLC.3.ser
skb-225_HPLC
β2
α6
TFAHN
β4
β4
β4
α6
R
α3
β4
21
β2
f1 (ppm)
f2 (ppm)

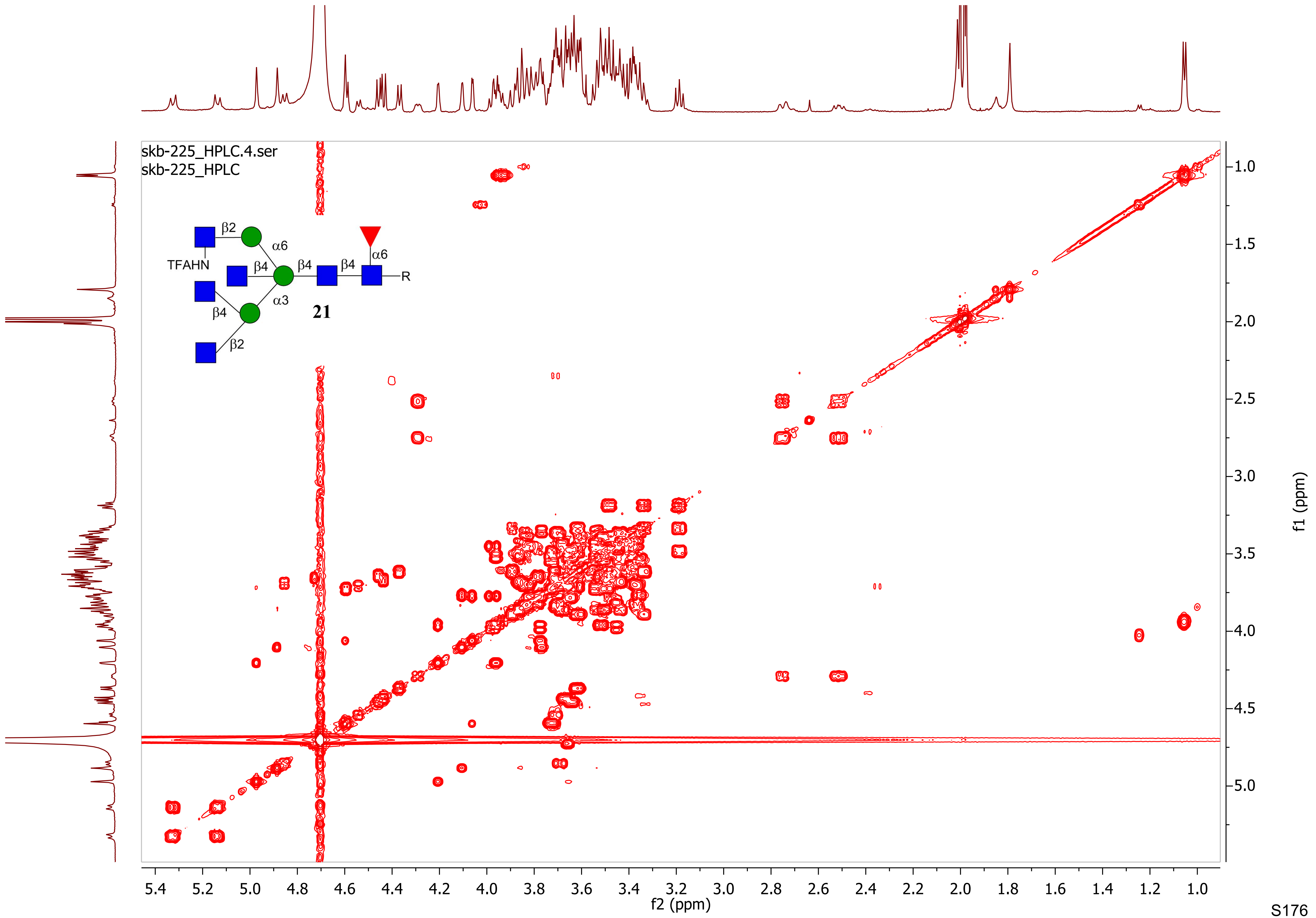
skb-225_HPLC.4.ser
skb-225_HPLC
β2
α6
TFAHN
β4
β4
β4
α6
R
α3
β4
β2
21
f2 (ppm)
f1 (ppm)

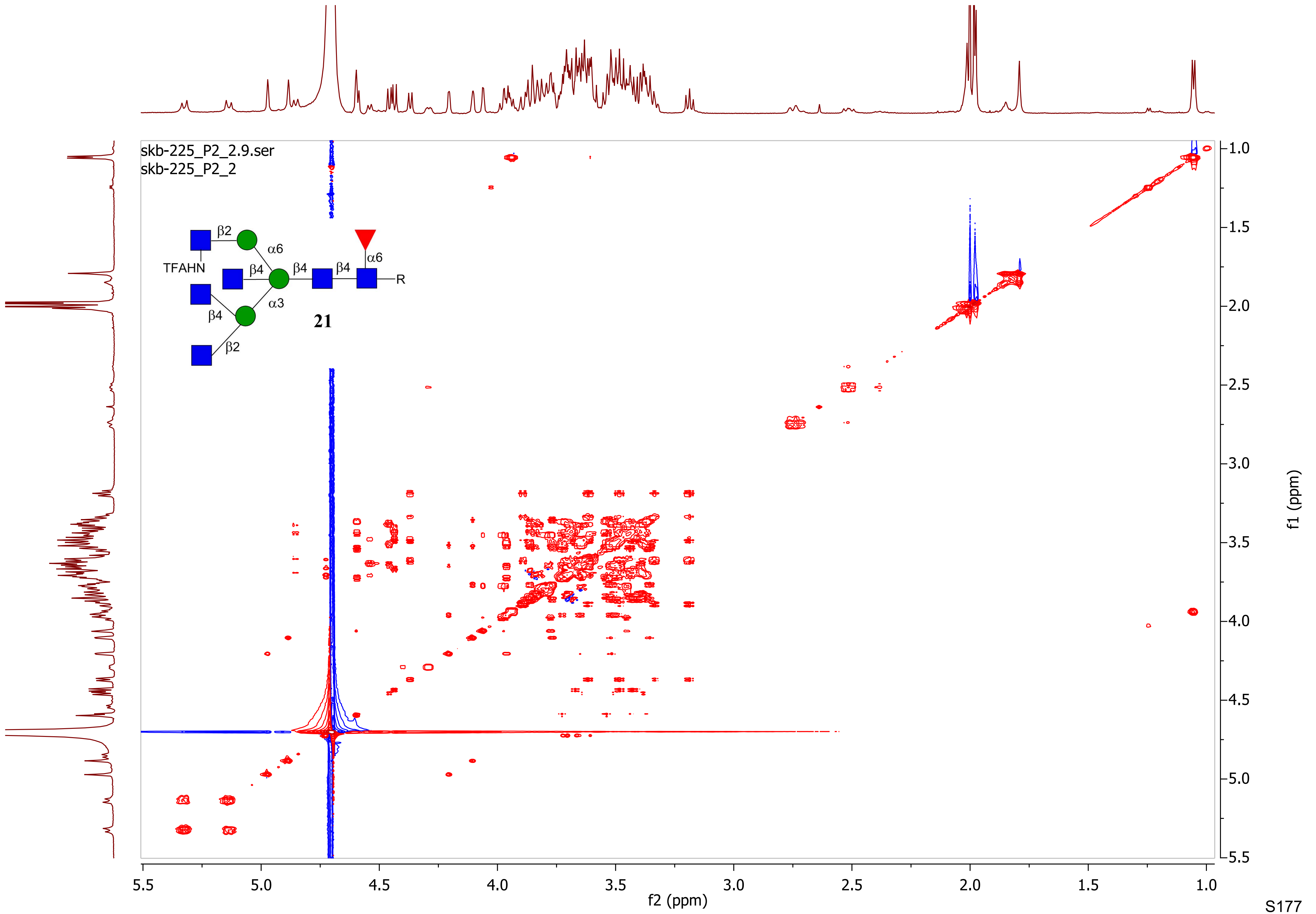
skb-225_P2_2.9.ser
skb-225_P2_2
β2
α6
TFAHN
β4
β4
β4
α6
R
α3
β4
β2
21
f2 (ppm)
f1 (ppm)

SKB-226_HPLC.1.fid
SKB-226_HPLC

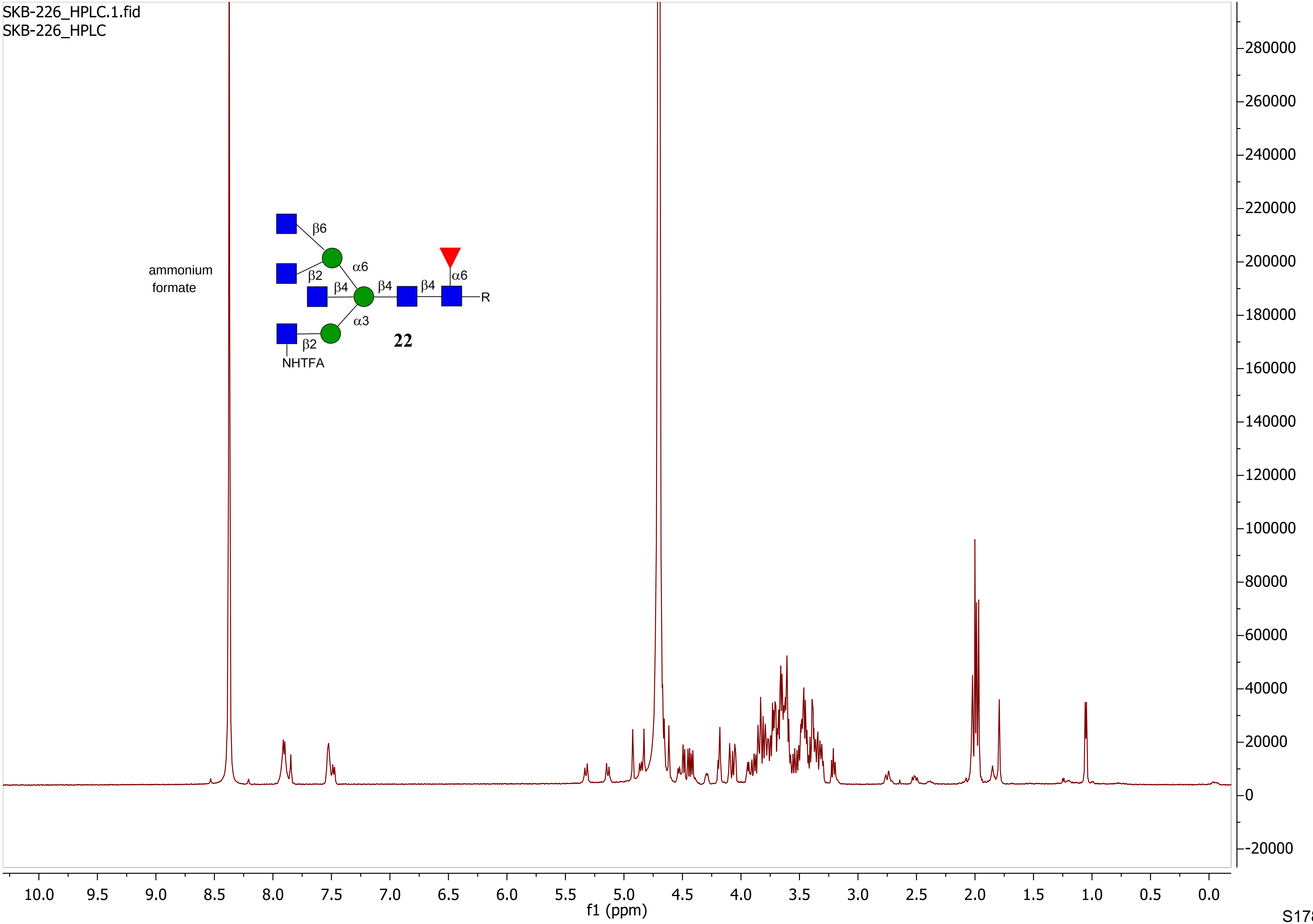

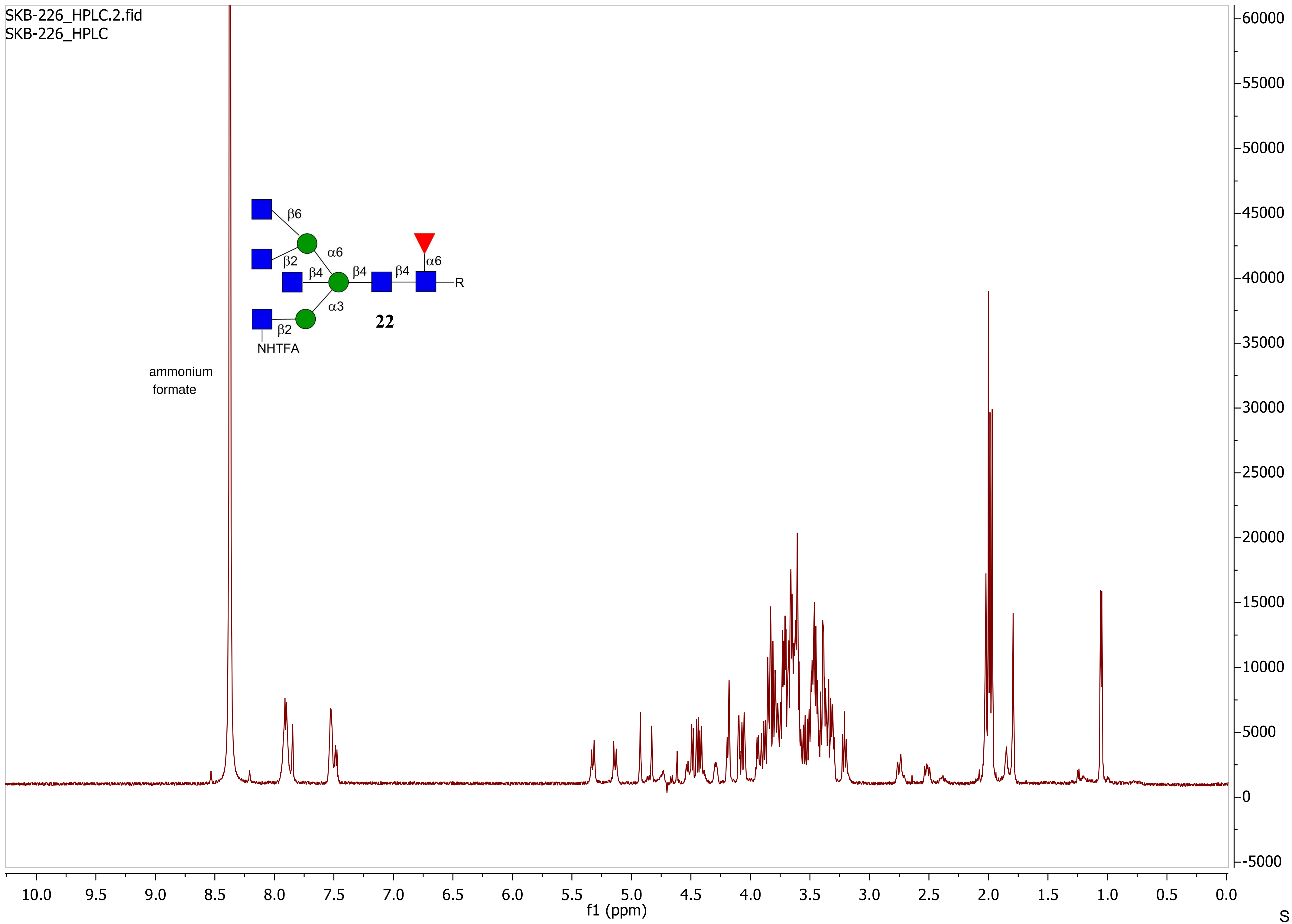

SKB-226_HPLC.2.fid
SKB-226_HPLC
β6
β2
α6
β4
β4
β4
α6
R
α3
β2
NHTFA
22
ammonium
formate
f1 (ppm)

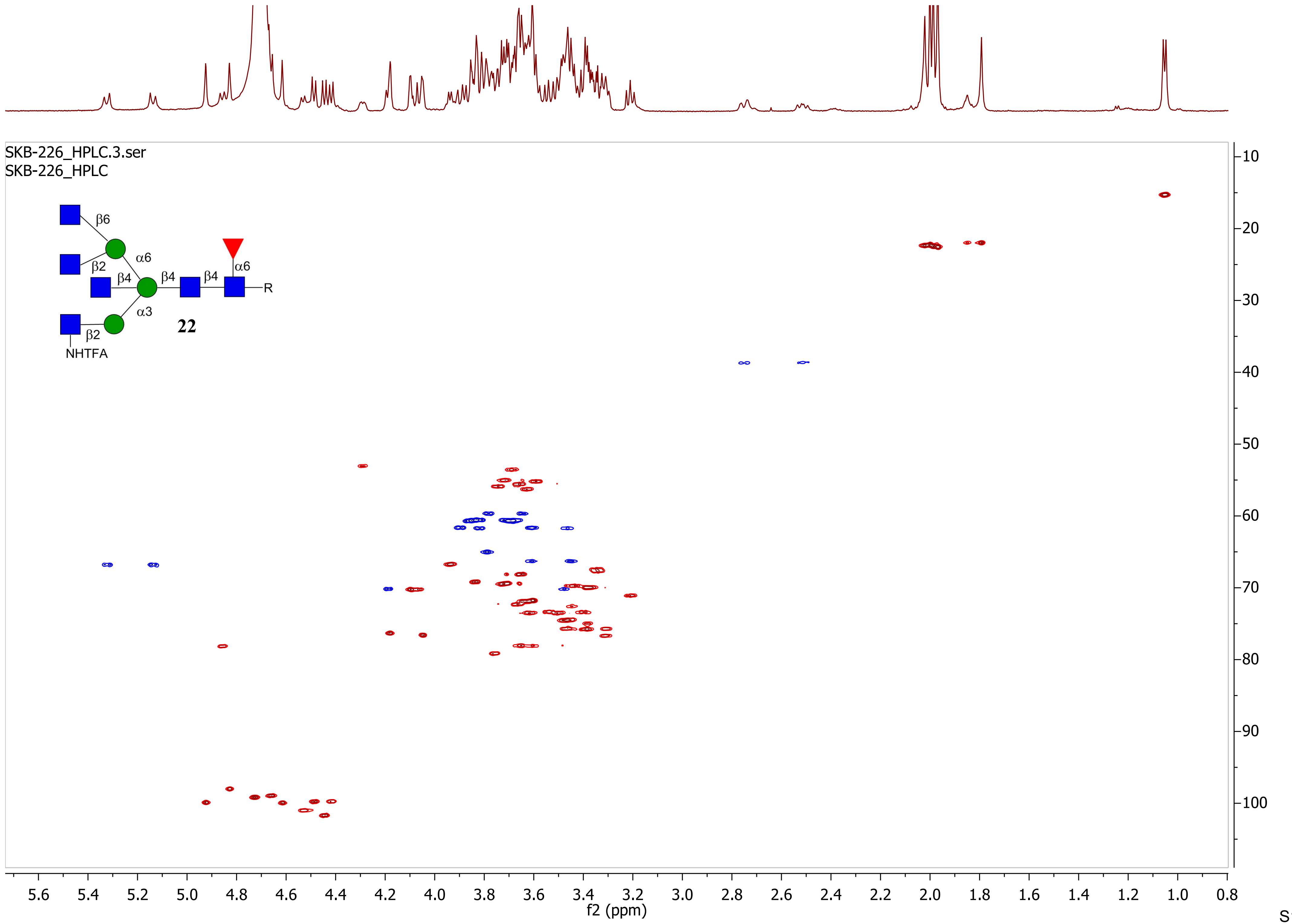
SKB-226_HPLC.3.ser
SKB-226_HPLC
β6
α6
β2
β4
β4
β4
α6
R
α3
22
β2
NHTFA
f2 (ppm)
f1 (ppm)

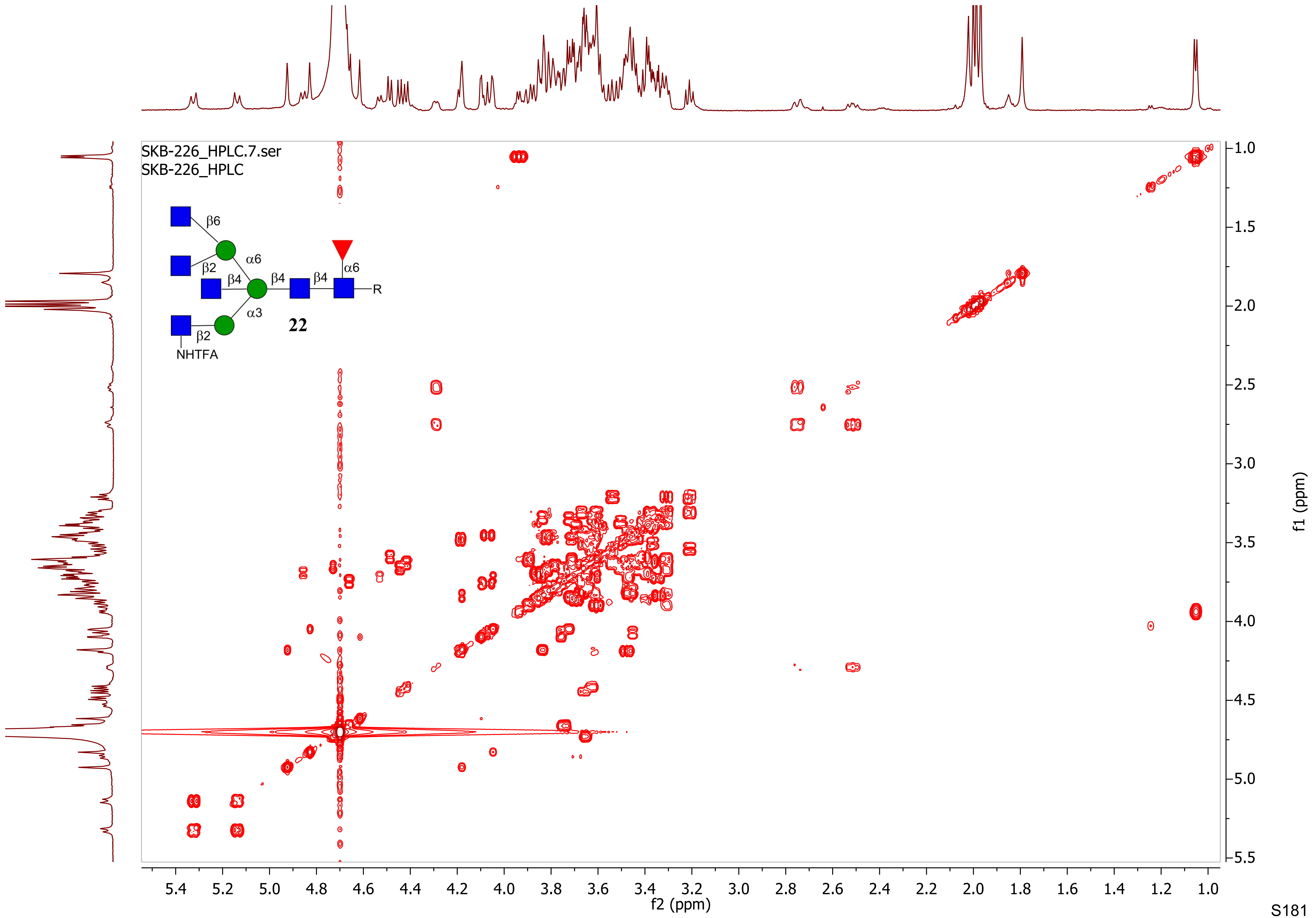
SKB-226_HPLC.7.ser
SKB-226_HPLC
β6
α6
β2
β4
β4
β4
α6
R
α3
22
β2
NHTFA
f2 (ppm)
f1 (ppm)

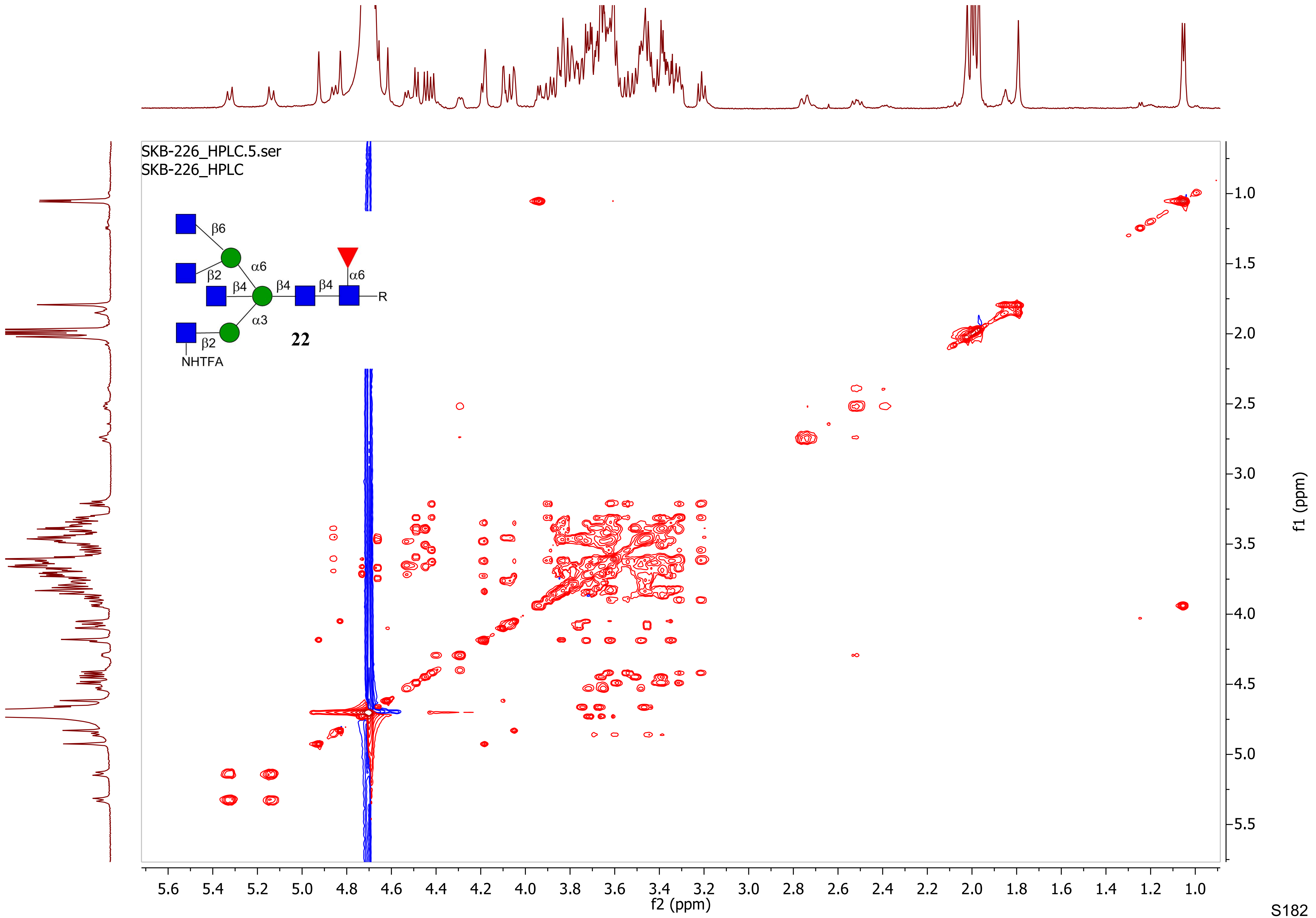
SKB-226_HPLC.5.ser
SKB-226_HPLC
β6
α6
β2
β4
β4
β4
α6
R
α3
22
β2
NHTFA
f1 (ppm)
f2 (ppm)

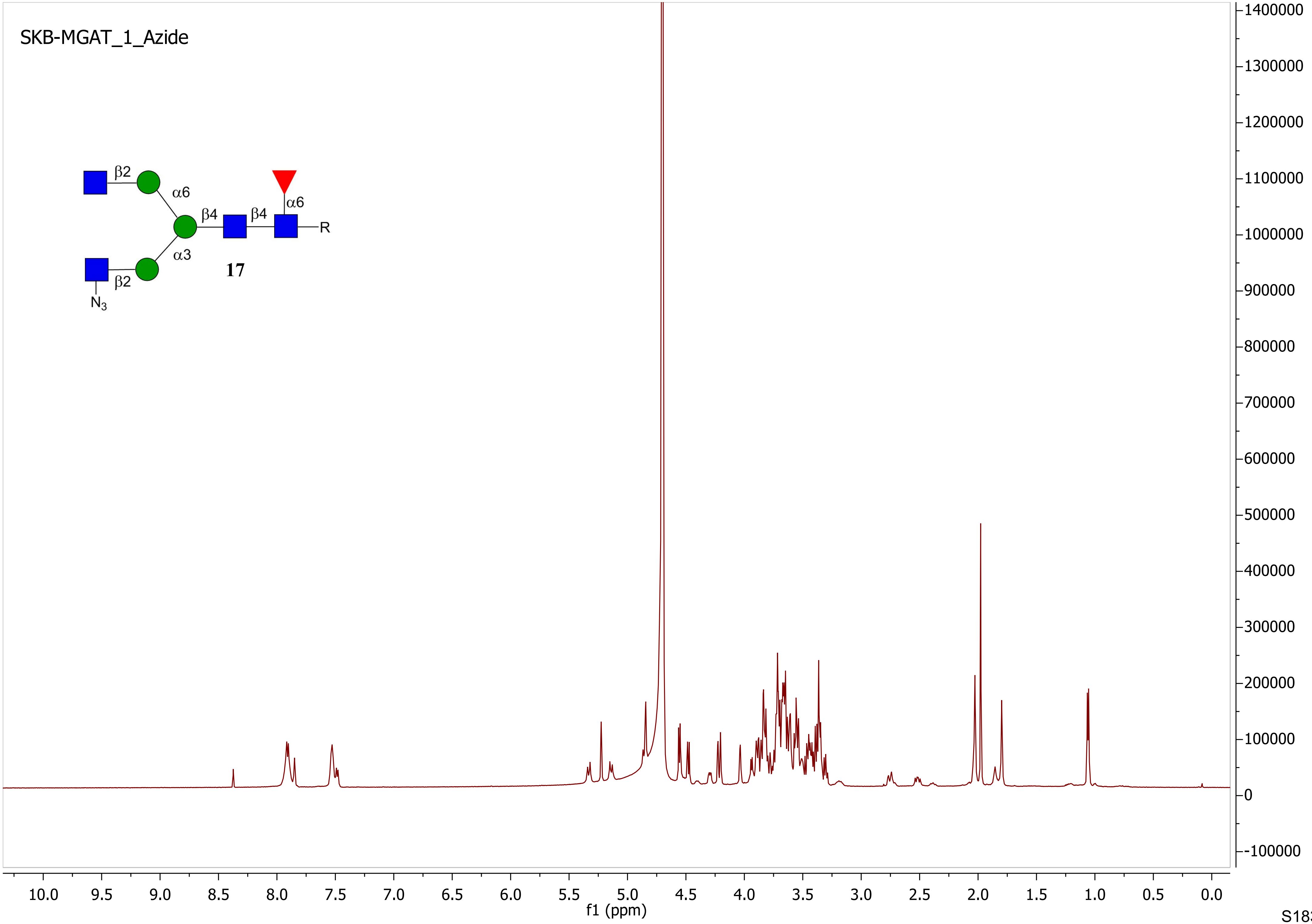
SKB-MGAT_1_Azide
β2
α6
β4
β4
α6
R
α3
β2
N_3
17
f1 (ppm)

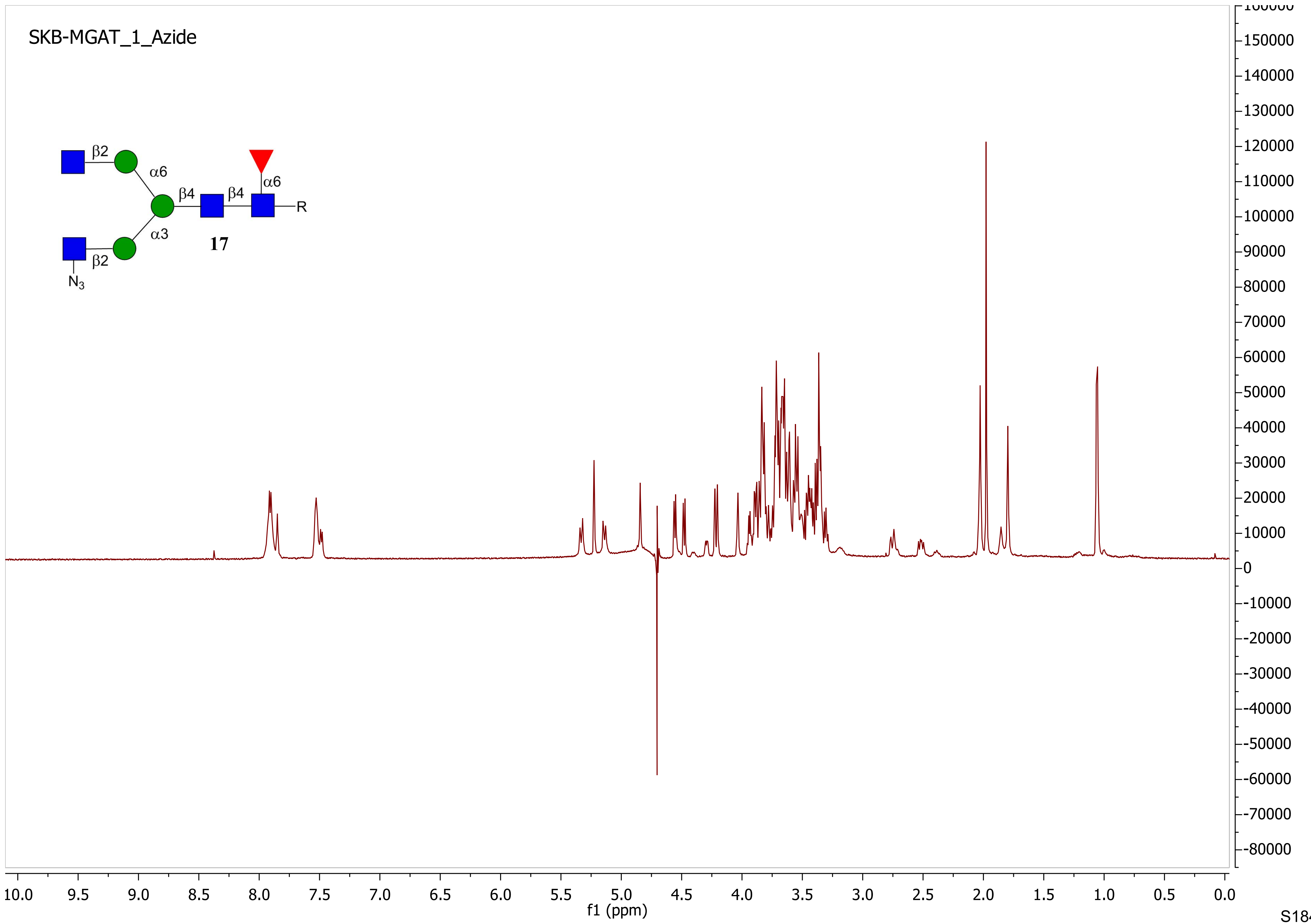
SKB-MGAT_1_Azide
β2
α6
β4
β4
α6
R
α3
17
β2
N3
f1 (ppm)

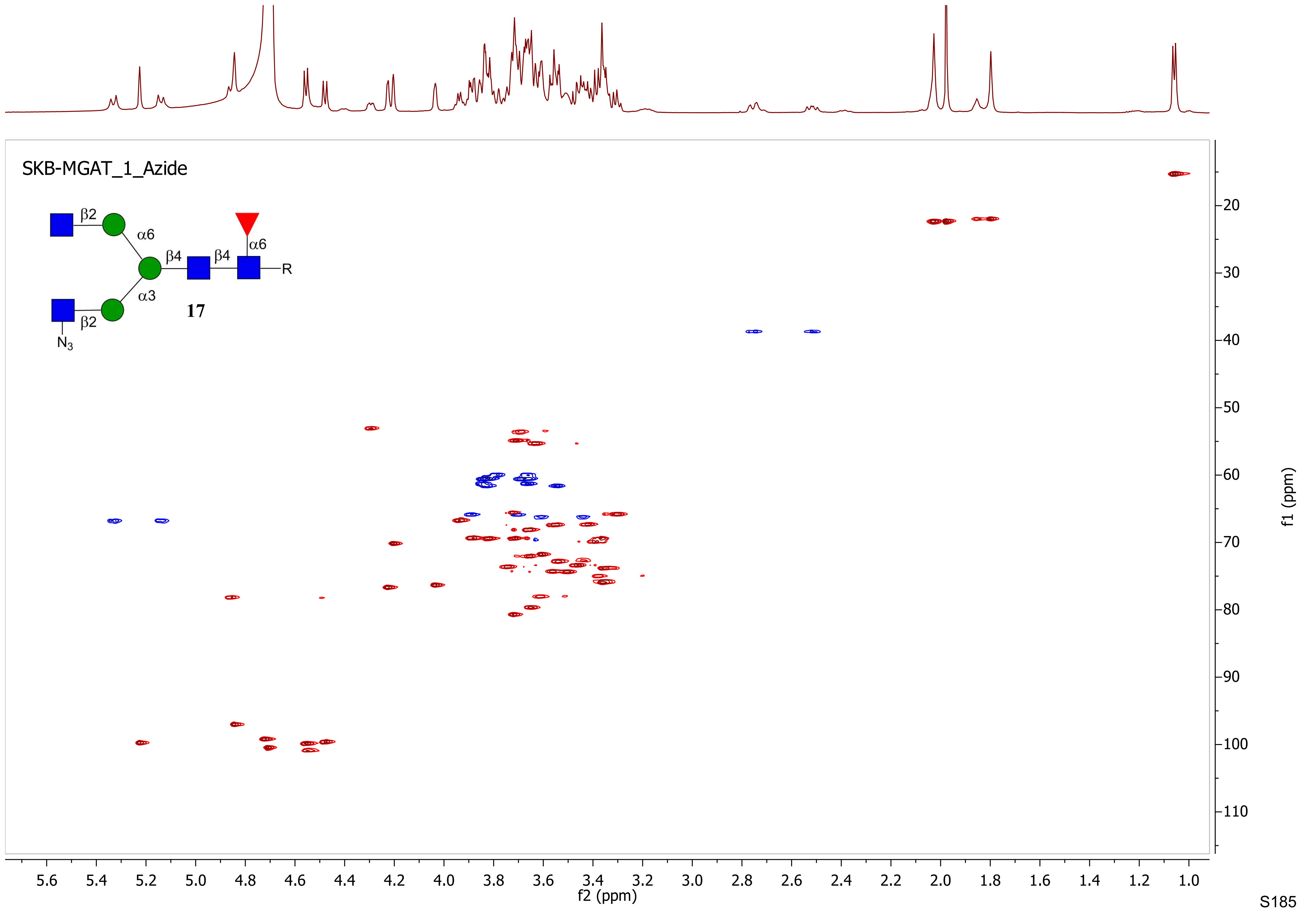
SKB-MGAT_1_Azide
β2
α6
β4
β4
α6
R
α3
17
β2
N3
f1 (ppm)
f2 (ppm)
5.6
5.4
5.2
5.0
4.8
4.6
4.4
4.2
4.0
3.8
3.6
3.4
3.2
3.0
2.8
2.6
2.4
2.2
2.0
1.8
1.6
1.4
1.2
1.0
20
30
40
50
60
70
80
90
100
110

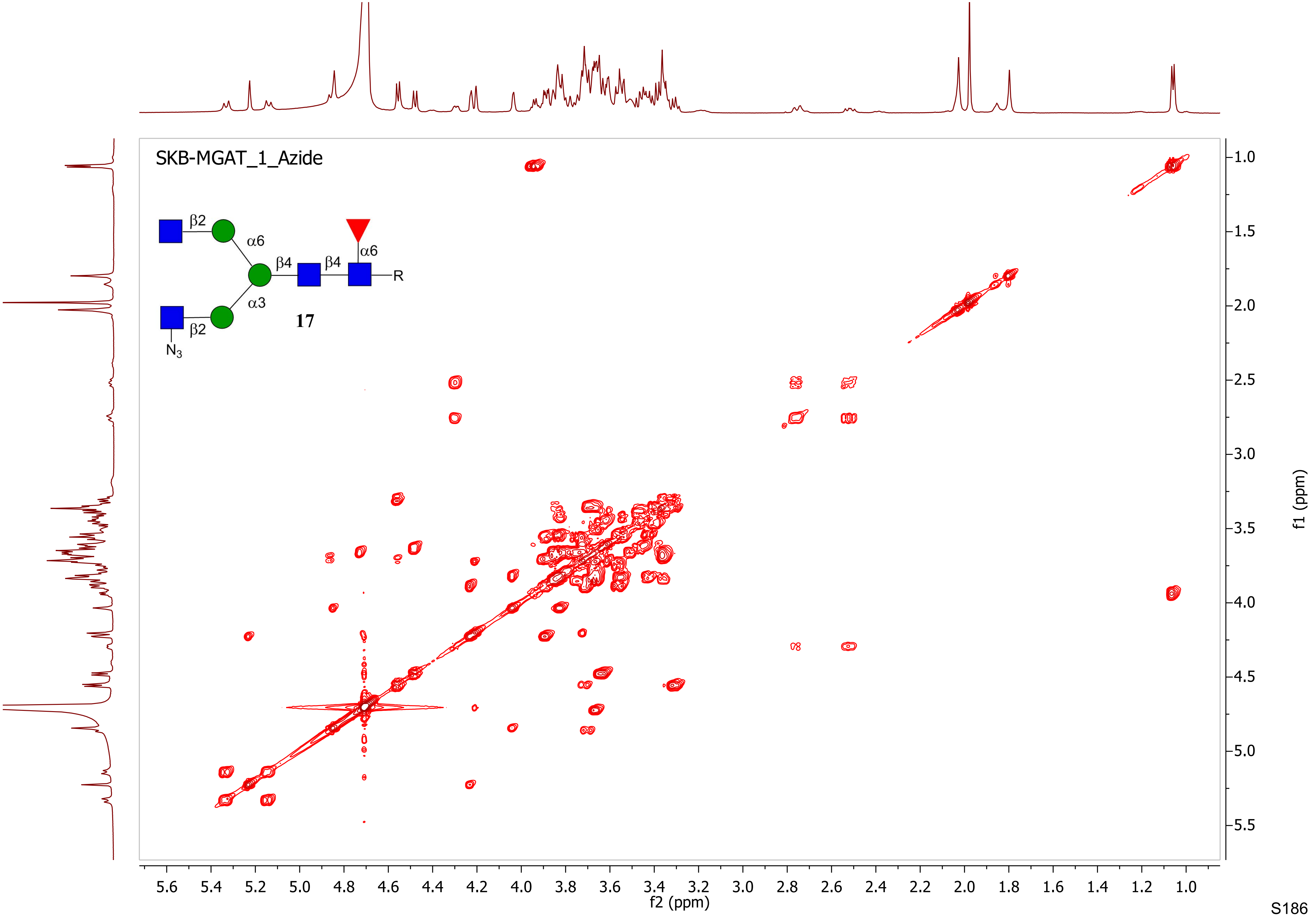
SKB-MGAT_1_Azide
β2
α6
β4
β4
α6
R
α3
β2
N3
17
f2 (ppm)
f1 (ppm)

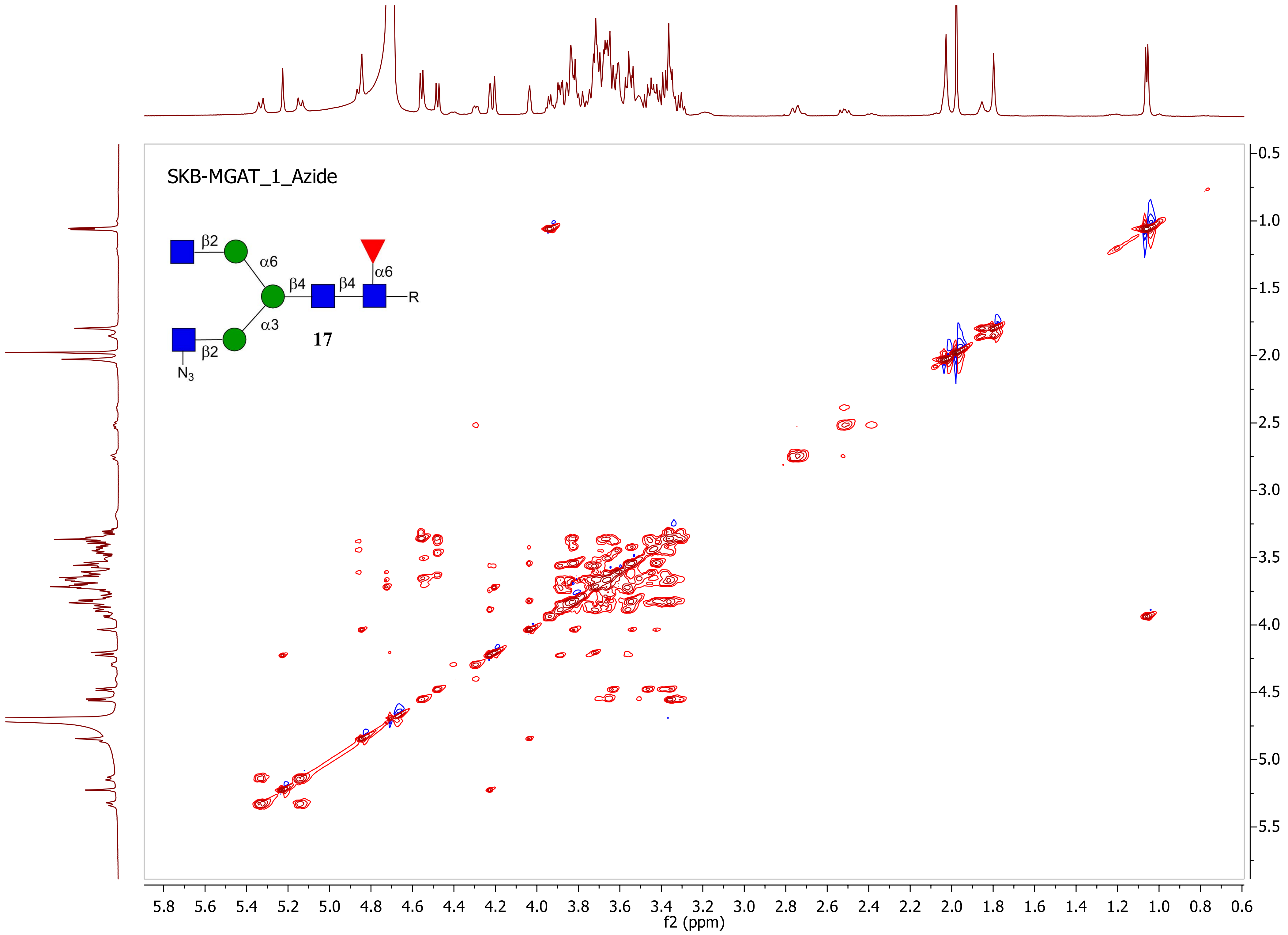
SKB-MGAT_1_Azide
β2
α6
β4
β4
α6
R
α3
β2
N3
17
f1 (ppm)
f2 (ppm)

SKB-116_Azide.12.fid
SKB-116_Azide

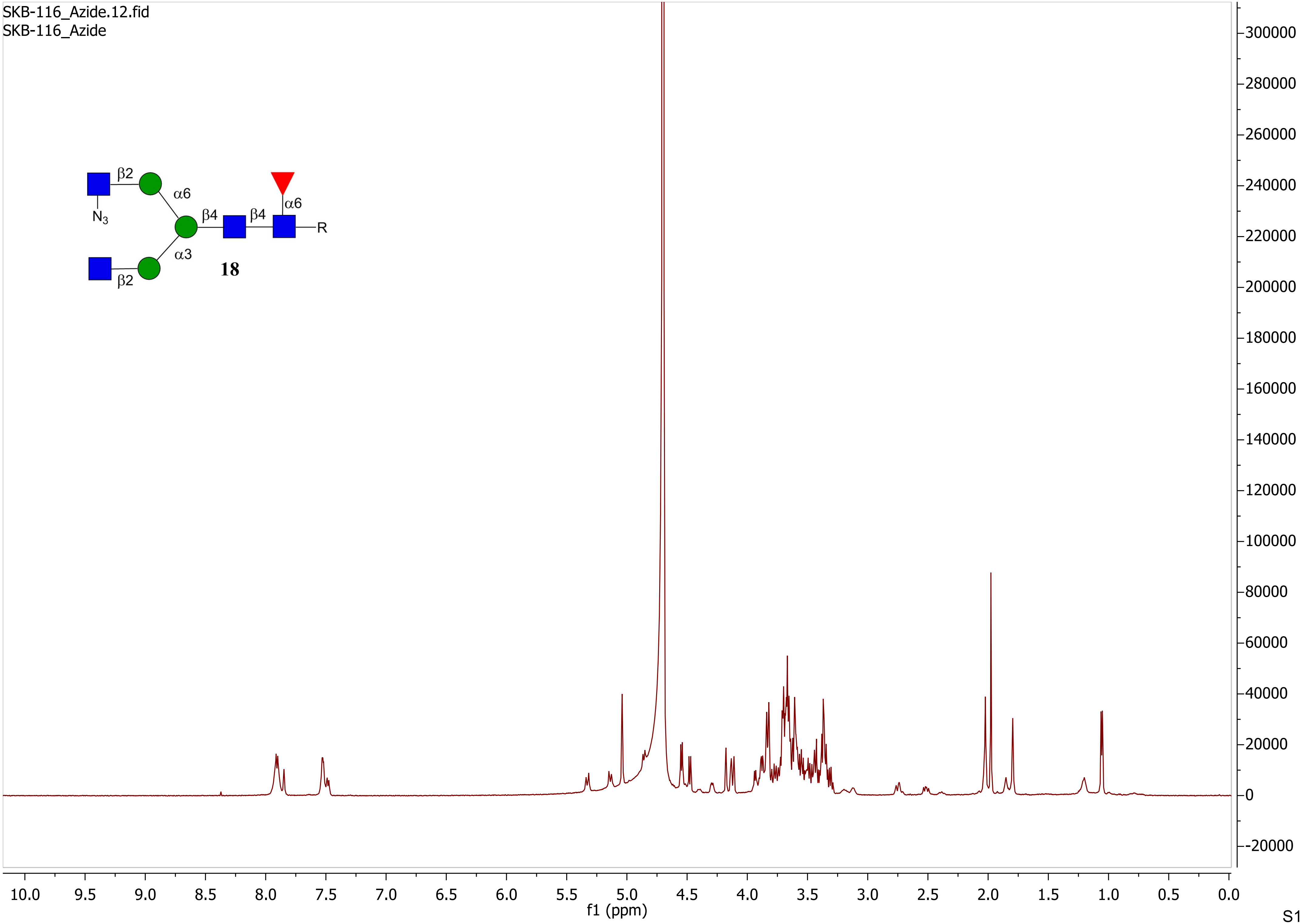

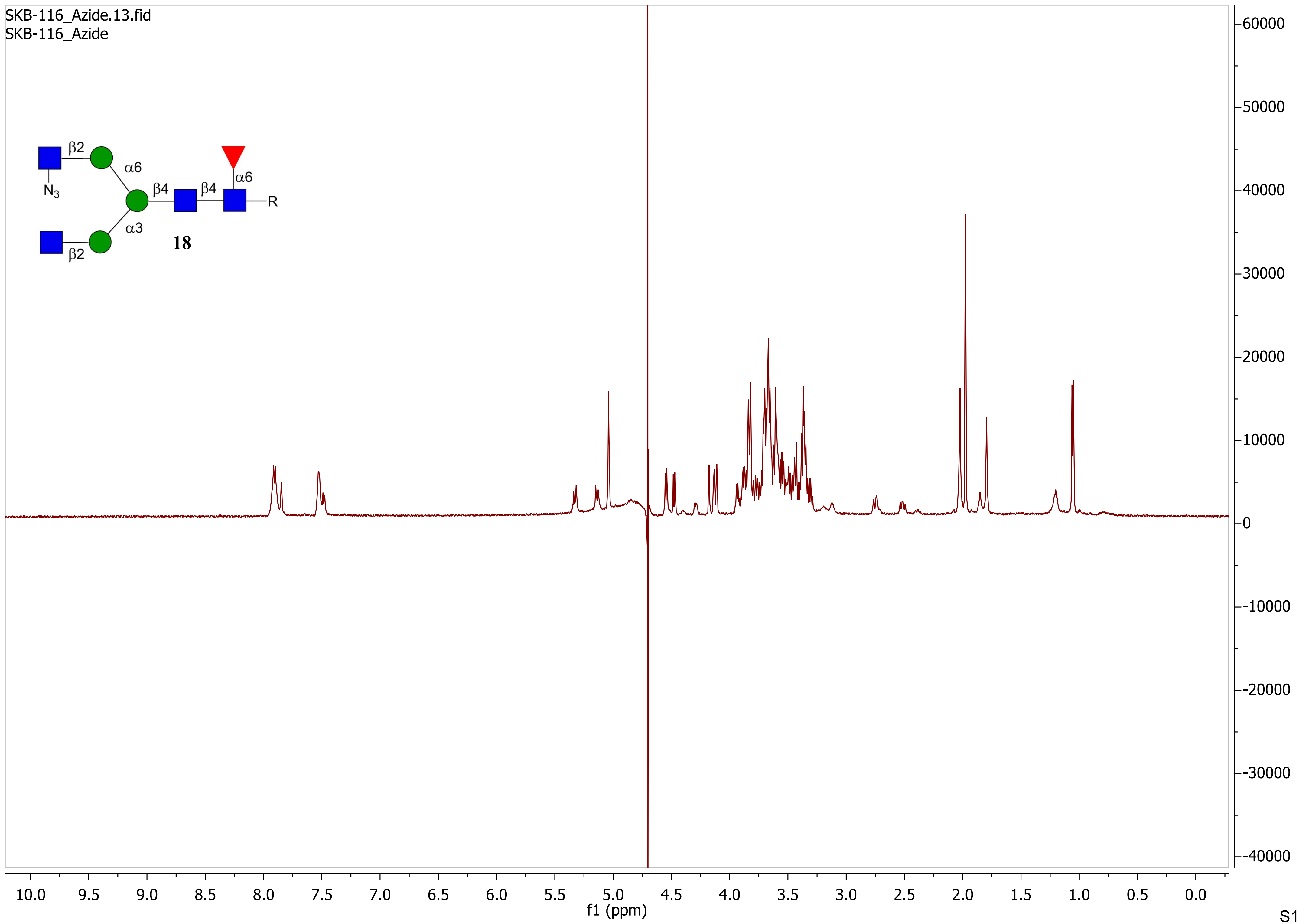
SKB-116_Azide.13.fid
SKB-116_Azide
β2
α6
N3
β4
β4
α6
R
α3
β2
18
f1 (ppm)
10.0
9.5
9.0
8.5
8.0
7.5
7.0
6.5
6.0
5.5
5.0
4.5
4.0
3.5
3.0
2.5
2.0
1.5
1.0
0.5
0.0
60000
50000
40000
30000
20000
10000
0
-10000
-20000
-30000
-40000

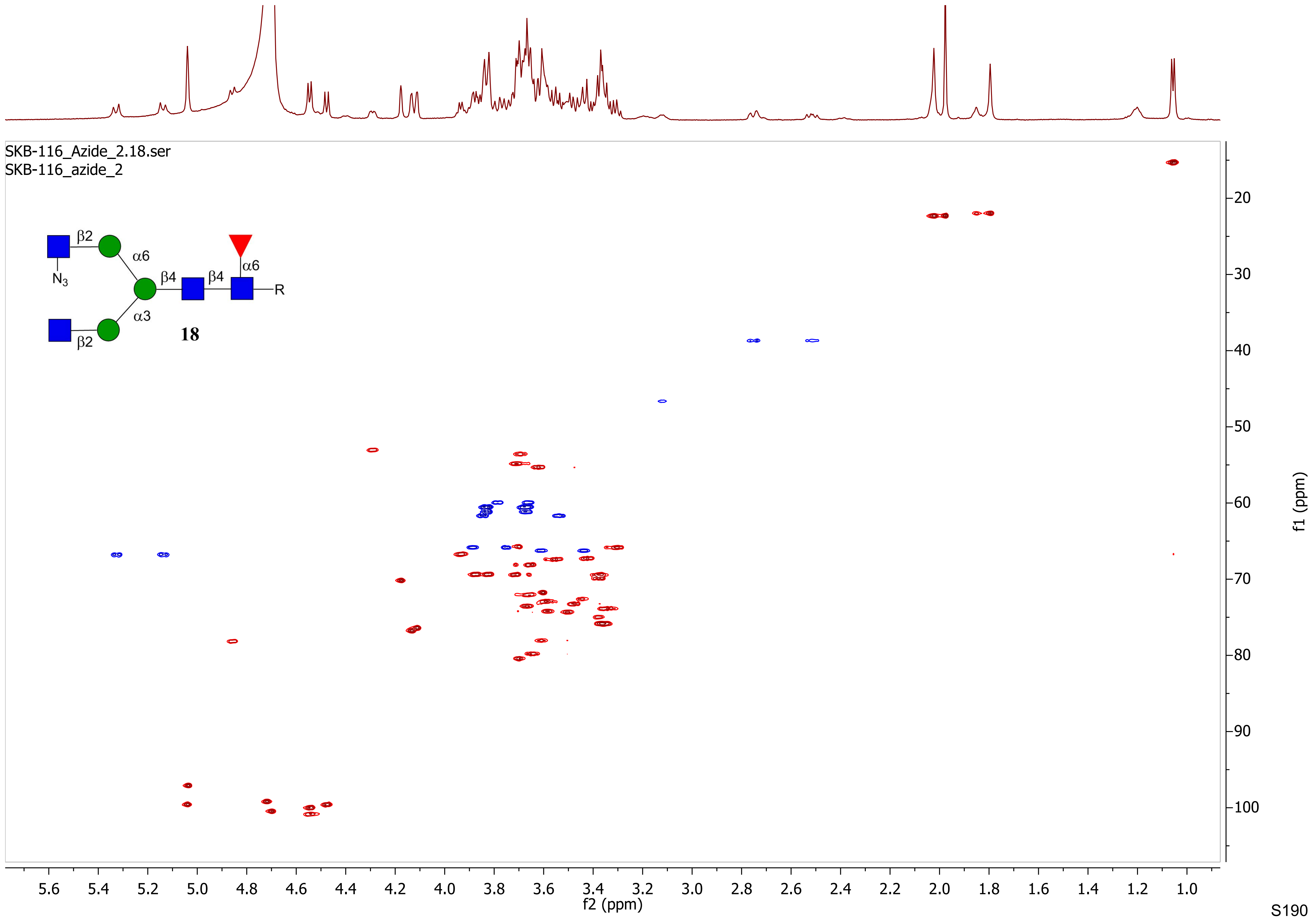
SKB-116_Azide_2.18.ser
SKB-116_azide_2
β2
α6
N3
β4
β4
α6
R
α3
β2
18
f2 (ppm)
f1 (ppm)

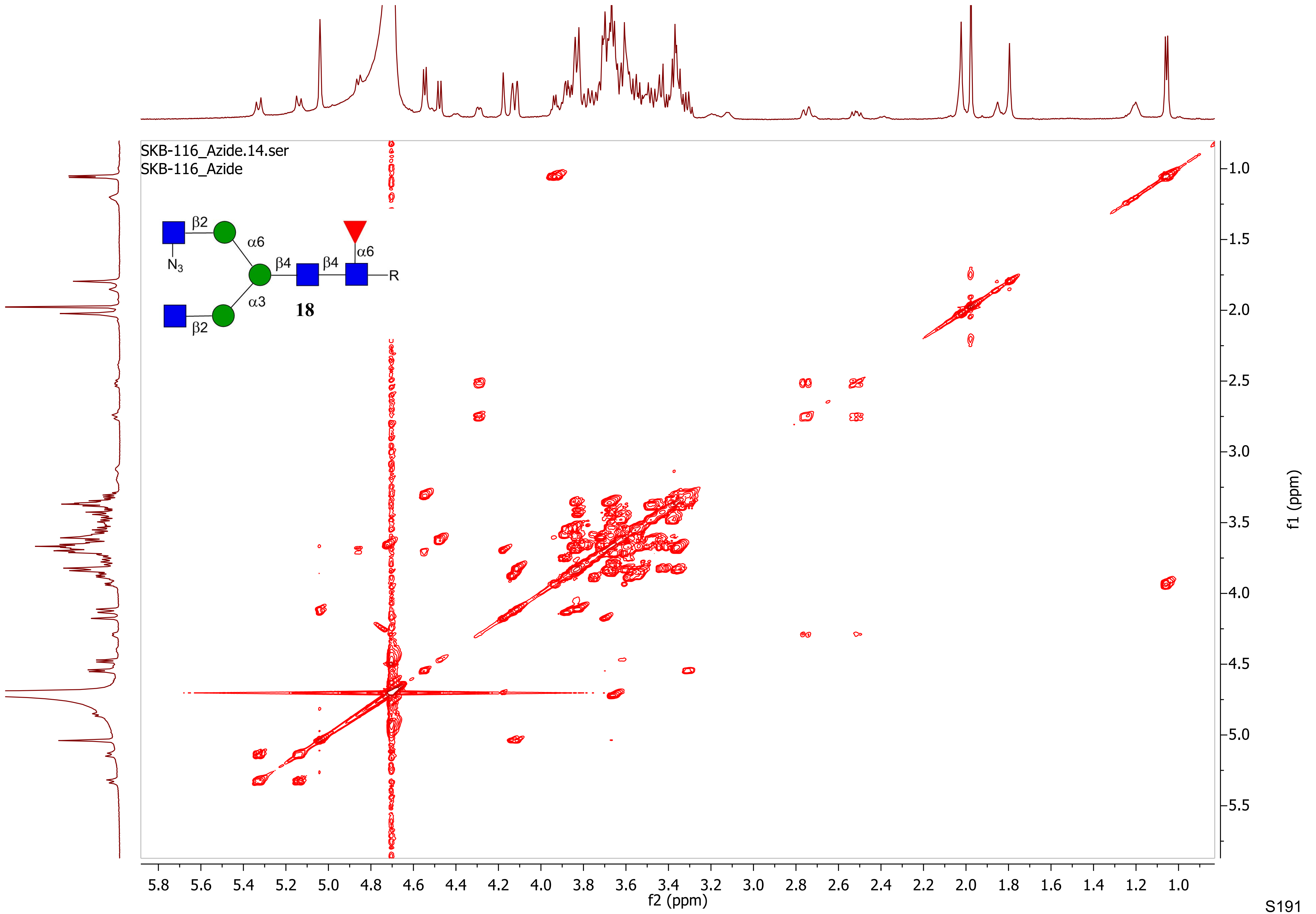
SKB-116_Azide.14.ser
SKB-116_Azide
β2
α6
N3
β4
β4
α6
R
α3
β2
18
f2 (ppm)
f1 (ppm)

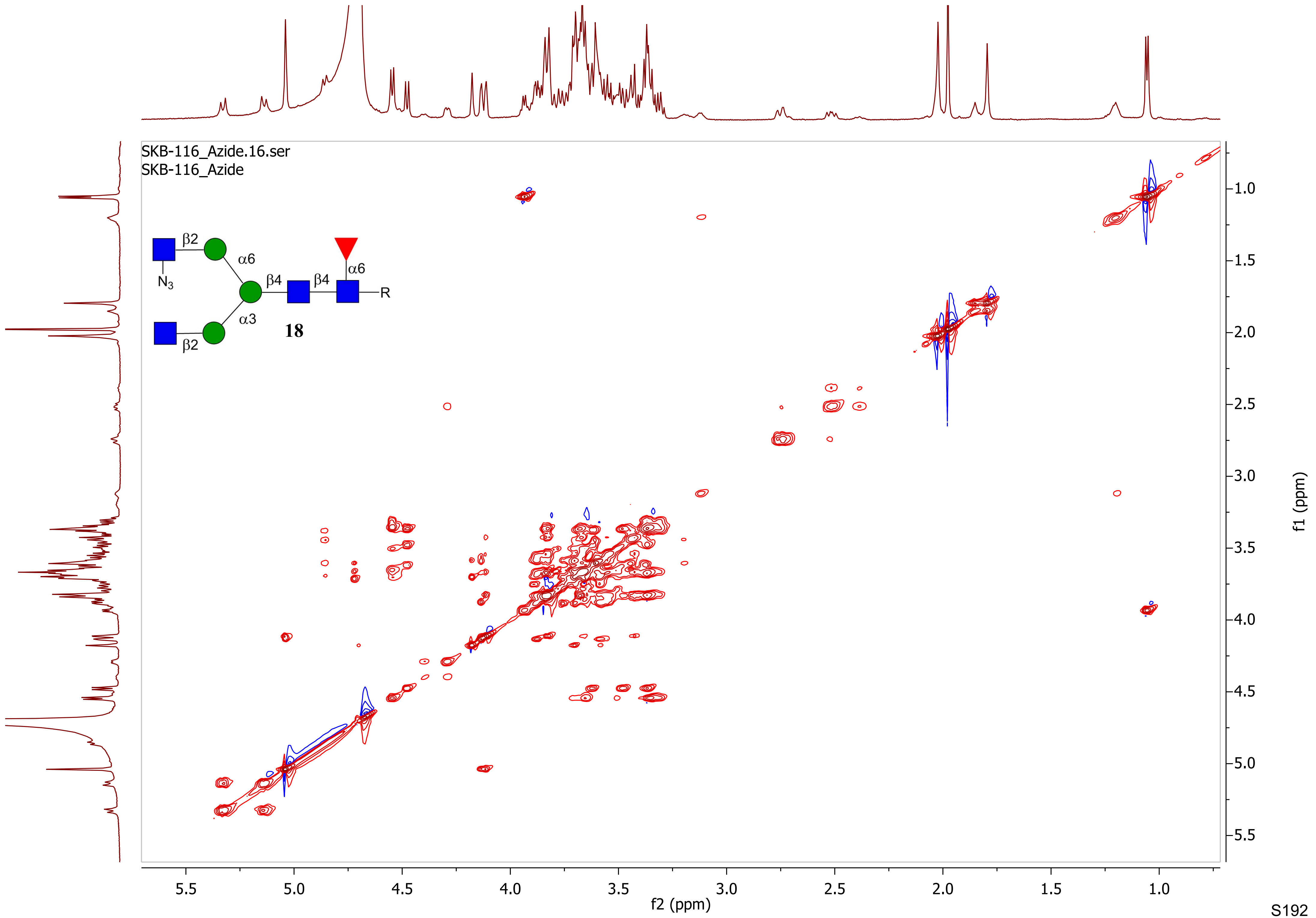
SKB-116_Azide.16.ser
SKB-116_Azide
β2
α6
N3
β4
β4
α6
R
α3
β2
18
f2 (ppm)
f1 (ppm)

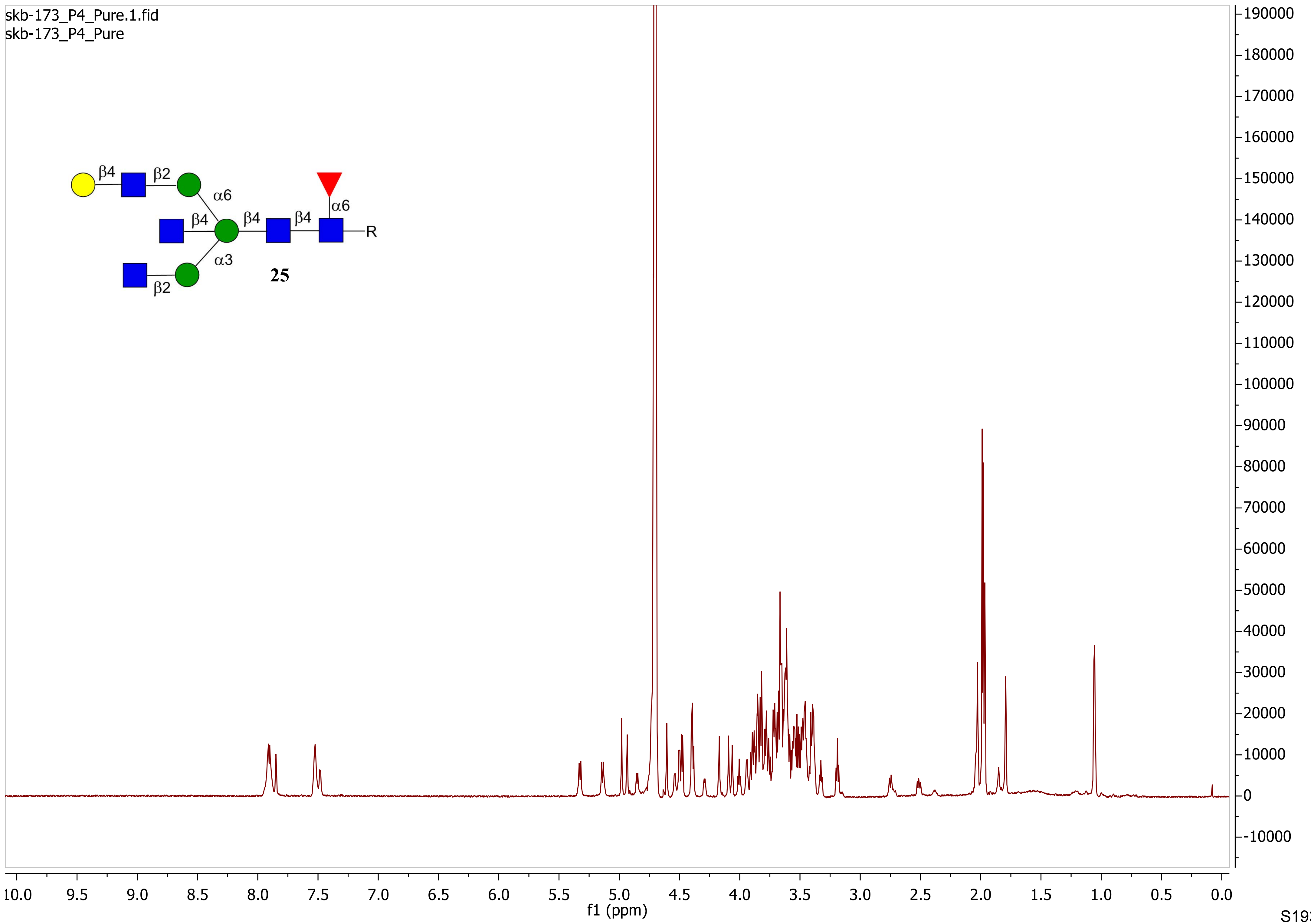
skb-173_P4_Pure.1.fid
skb-173_P4_Pure
β4
β2
α6
β4
β4
β4
α6
R
α3
β2
25
f1 (ppm)

skb-173_P4_Pure.2.fid
skb-173_P4_Pure

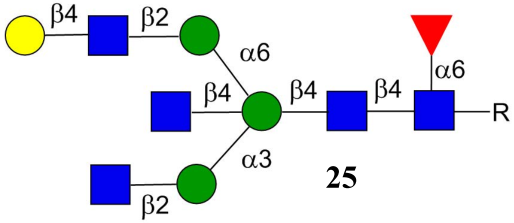

25

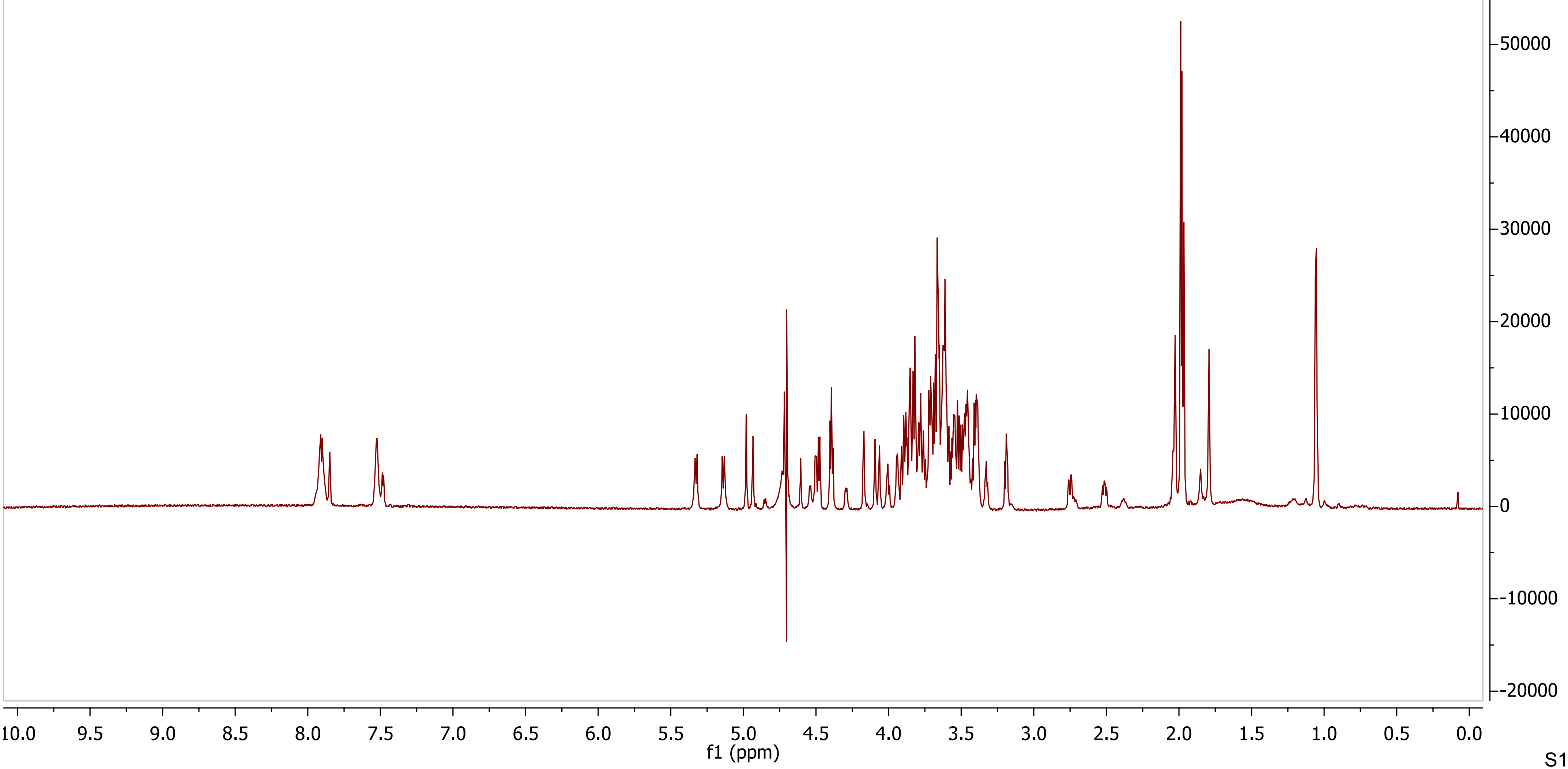

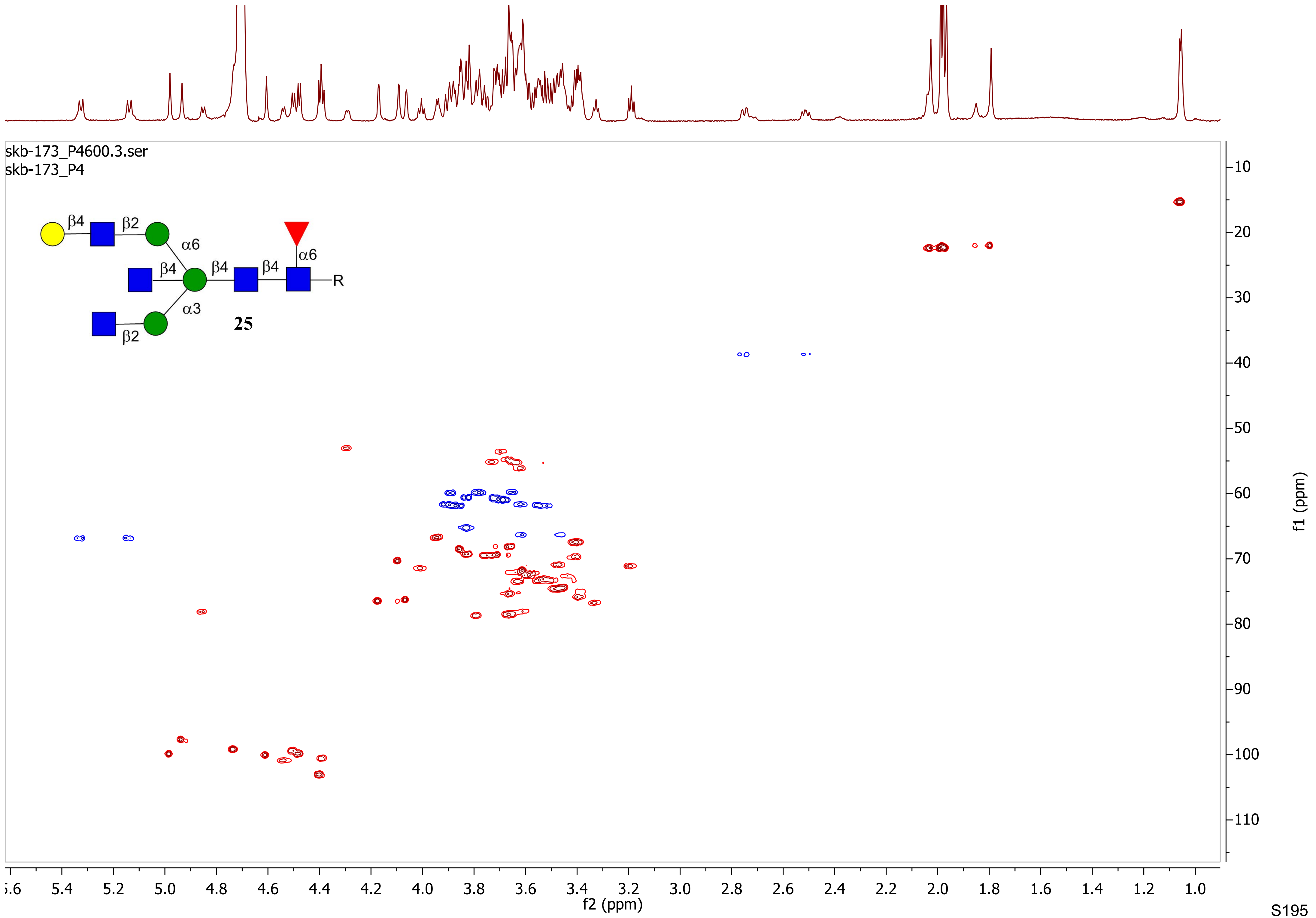
skb-173_P4600.3.ser
skb-173_P4
β4
β2
α6
β4
β4
β4
α6
R
α3
β2
25
f2 (ppm)
f1 (ppm)

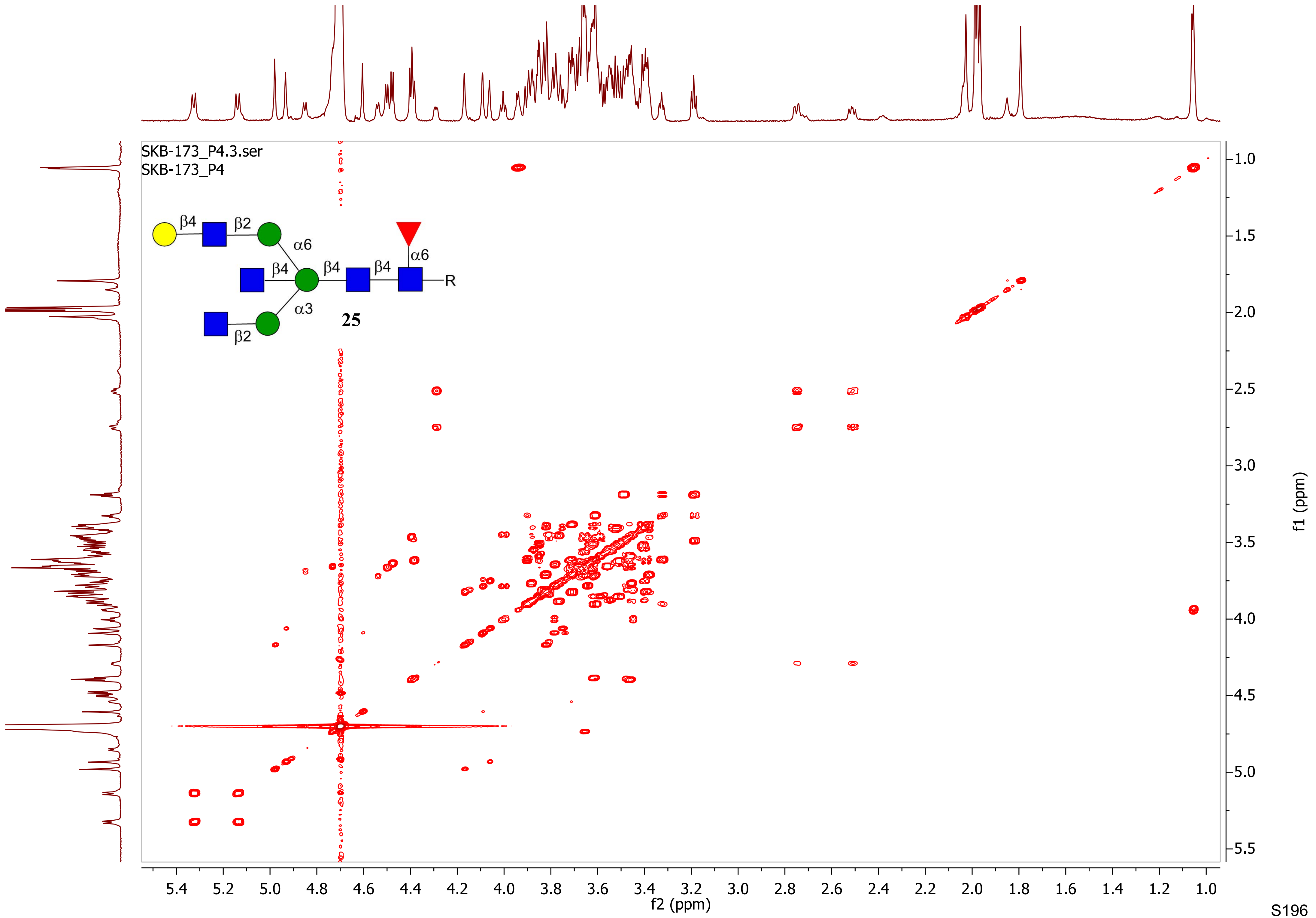
SKB-173_P4.3.ser
SKB-173_P4
β4
β2
α6
β4
β4
β4
α6
R
α3
β2
25
f2 (ppm)
f1 (ppm)

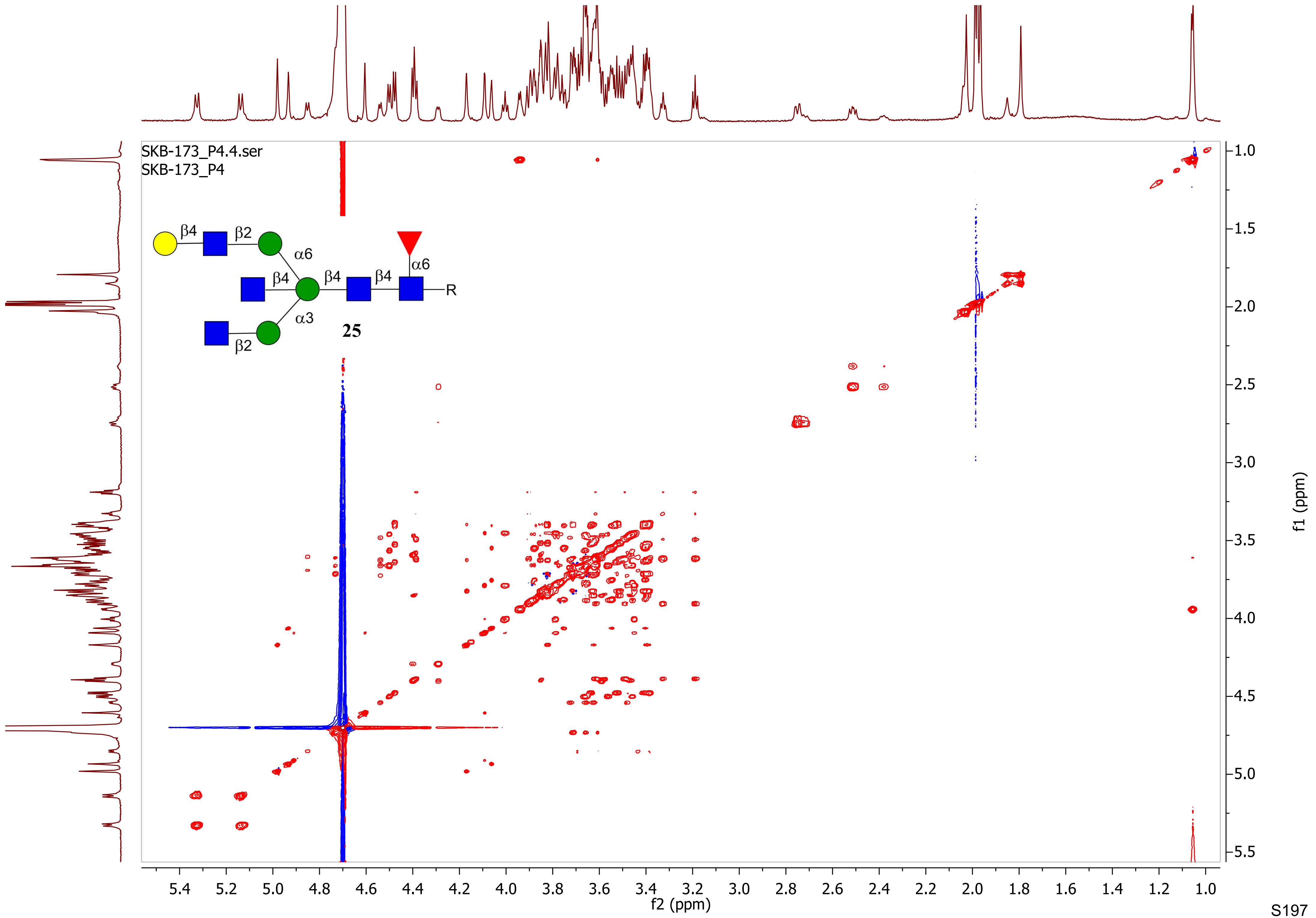

SKB-173_P4.4.ser
SKB-173_P4
β4
β2
α6
β4
β4
β4
α6
R
α3
β2
25
5.4
5.2
5.0
4.8
4.6
4.4
4.2
4.0
3.8
3.6
3.4
3.2
3.0
2.8
2.6
2.4
2.2
2.0
1.8
1.6
1.4
1.2
1.0
f2 (ppm)
1.0
1.5
2.0
2.5
3.0
3.5
4.0
4.5
5.0
5.5
f1 (ppm)

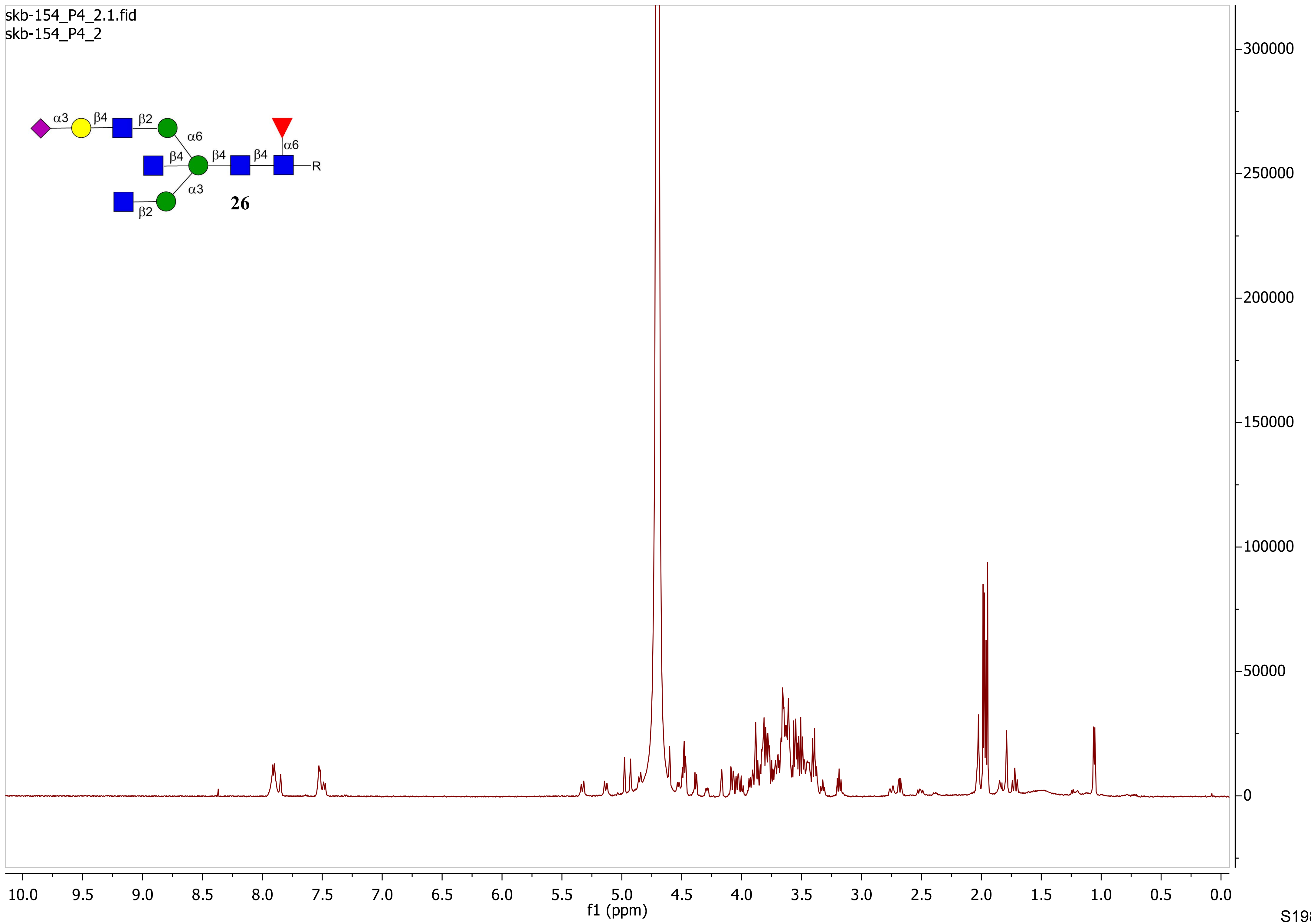
skb-154_P4_2.1.fid
skb-154_P4_2
α3
β4
β2
α6
β4
β4
β4
α6
R
α3
β2
26
f1 (ppm)

skb-154_P4_2.2.fid
skb-154_P4_2

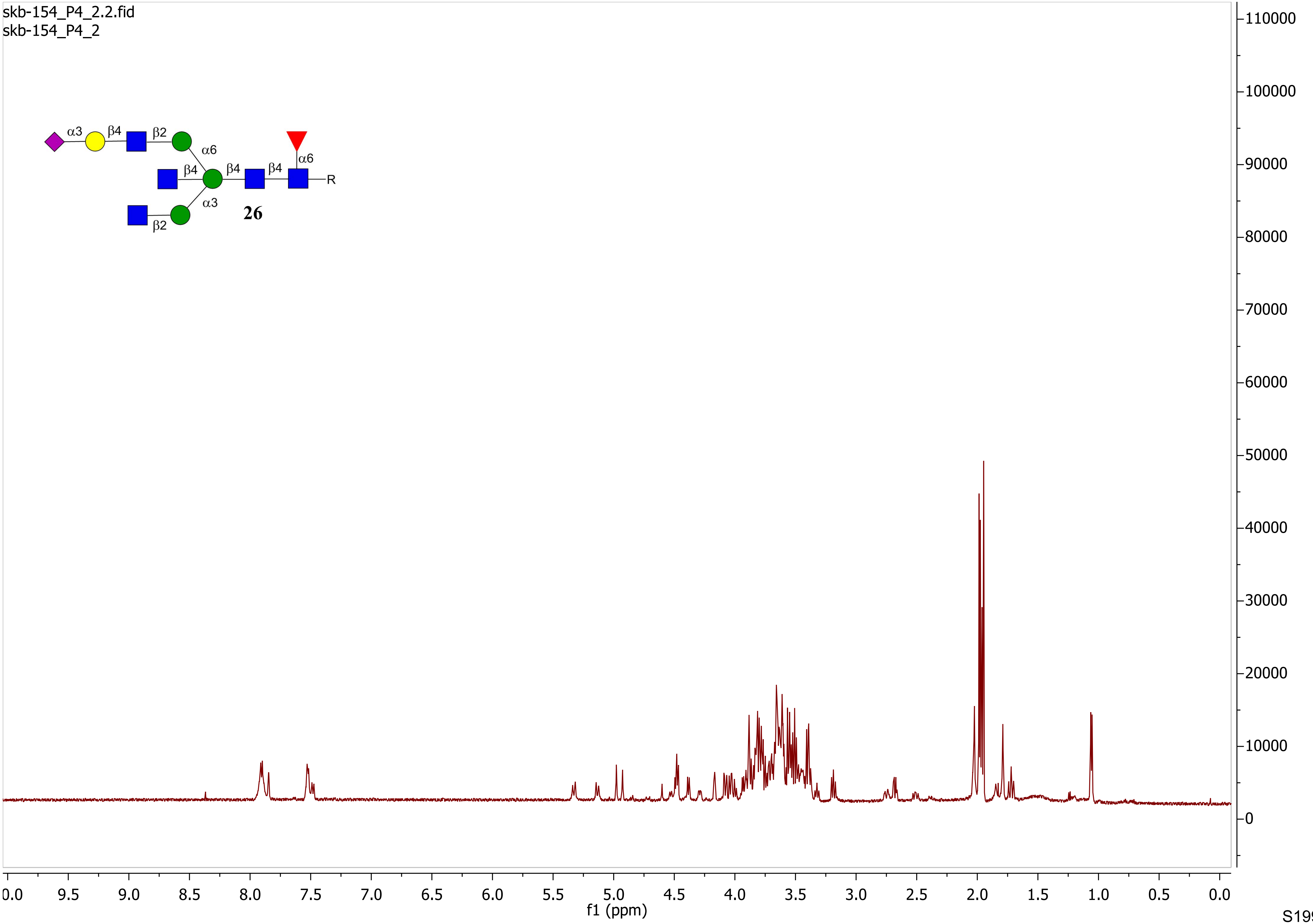

26

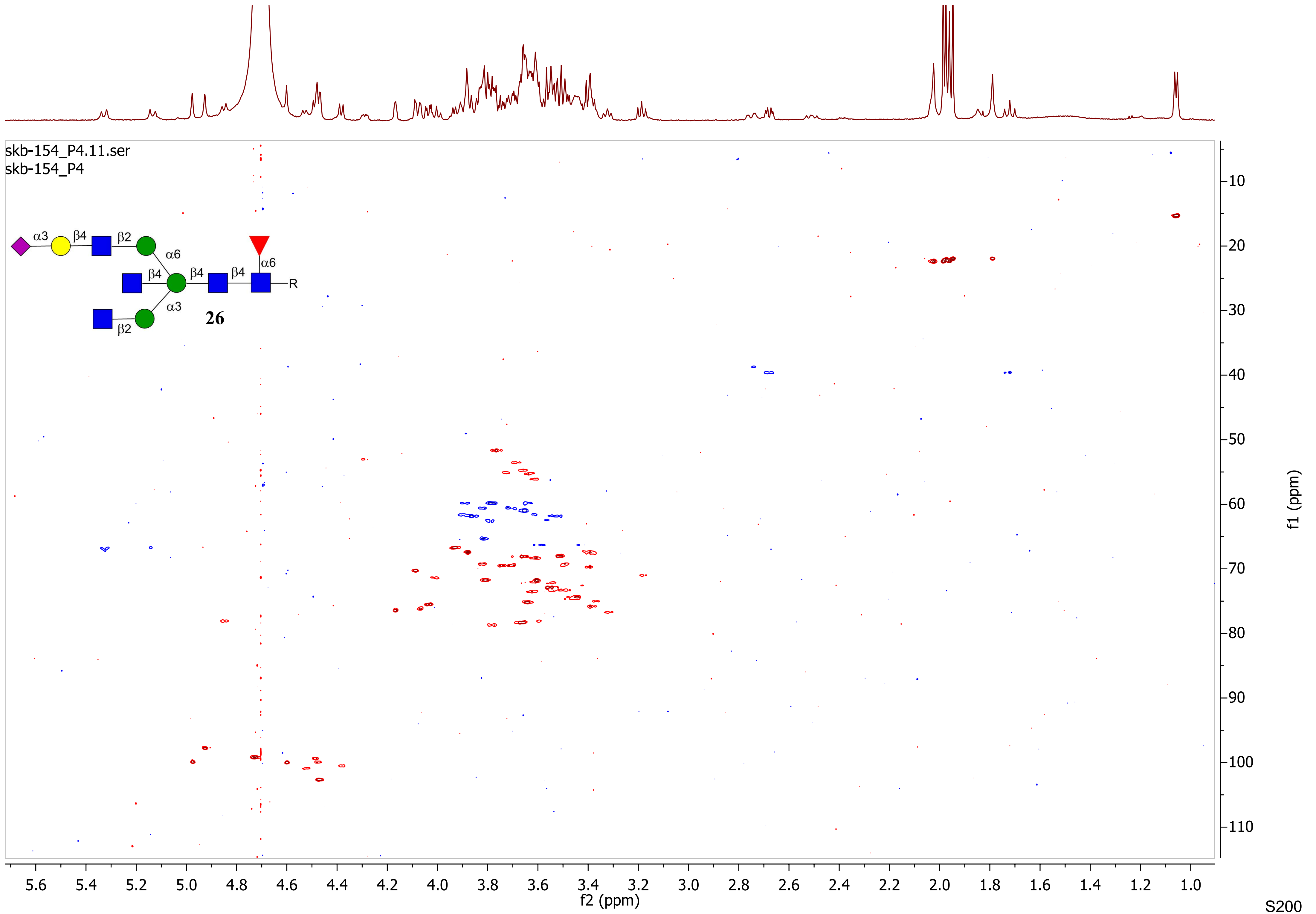
skb-154_P4.11.ser
skb-154_P4
α3
β4
β2
α6
β4
β4
β4
α6
R
α3
β2
26
f2 (ppm)
f1 (ppm)

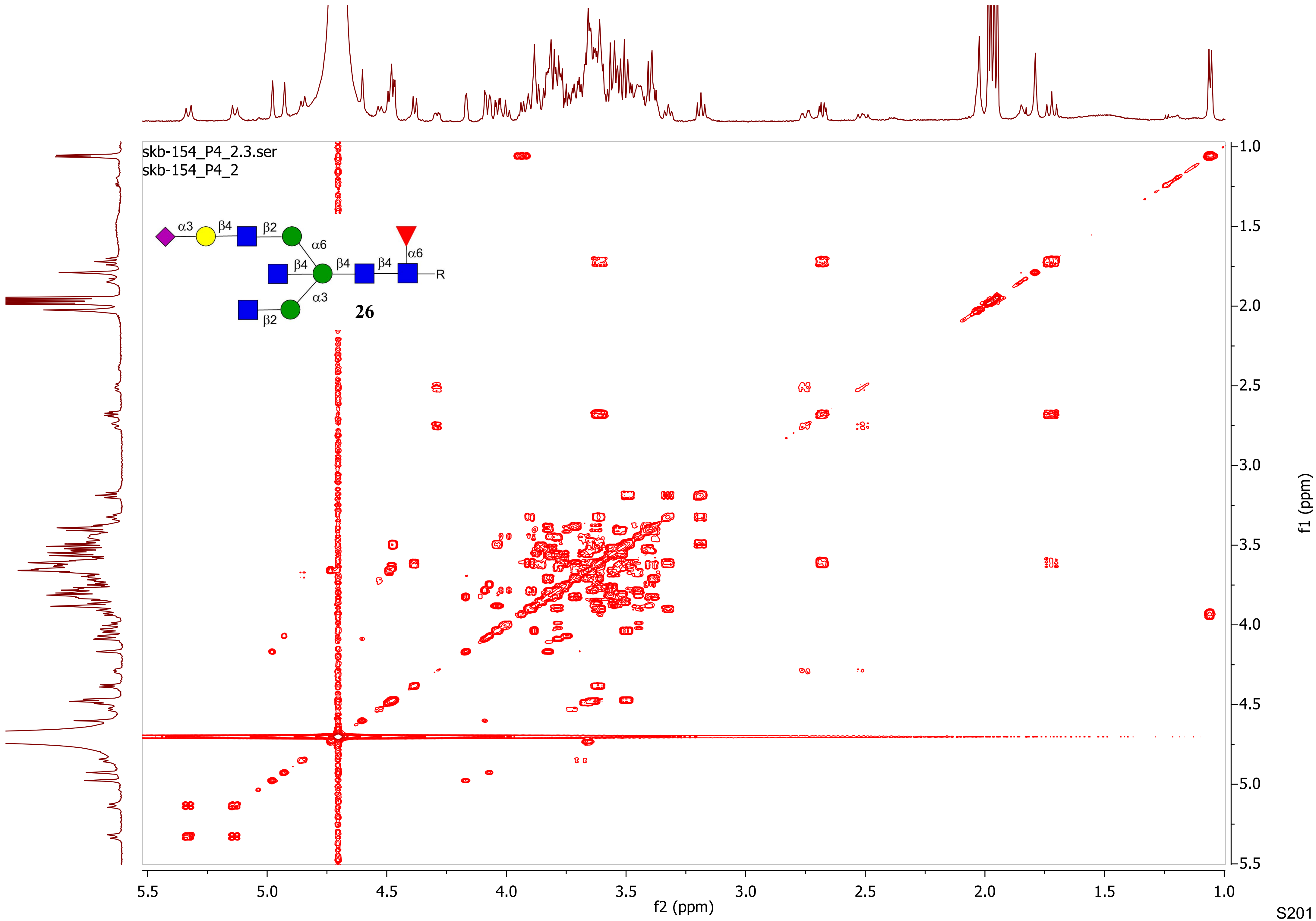

skb-154_P4_2.3.ser
skb-154_P4_2
α3
β4
β2
α6
β4
β4
β4
α6
R
α3
β2
26
f2 (ppm)
f1 (ppm)

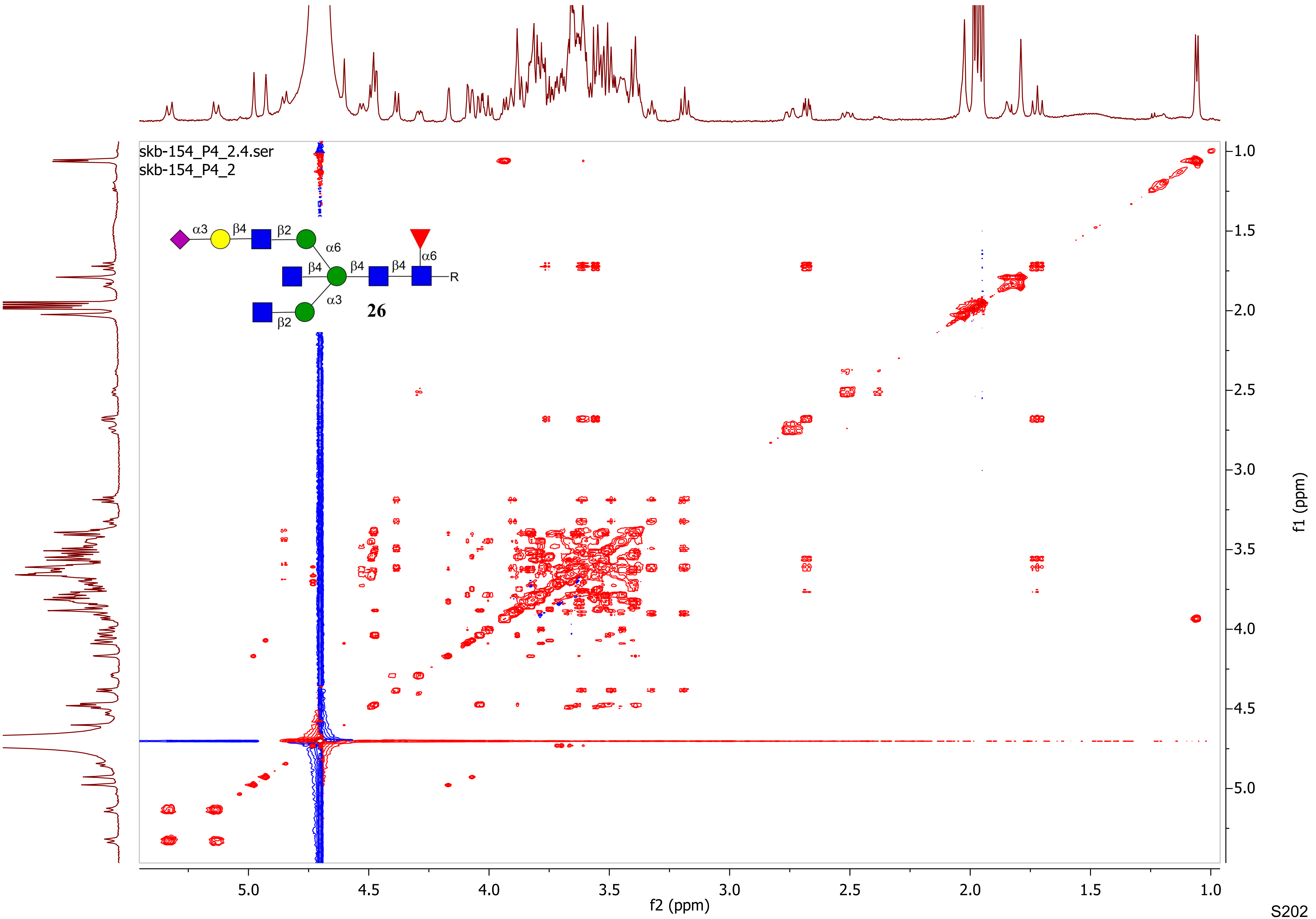
skb-154_P4_2.4.ser
skb-154_P4_2
α3
β4
β2
α6
β4
β4
β4
α6
R
α3
β2
26
f2 (ppm)
f1 (ppm)

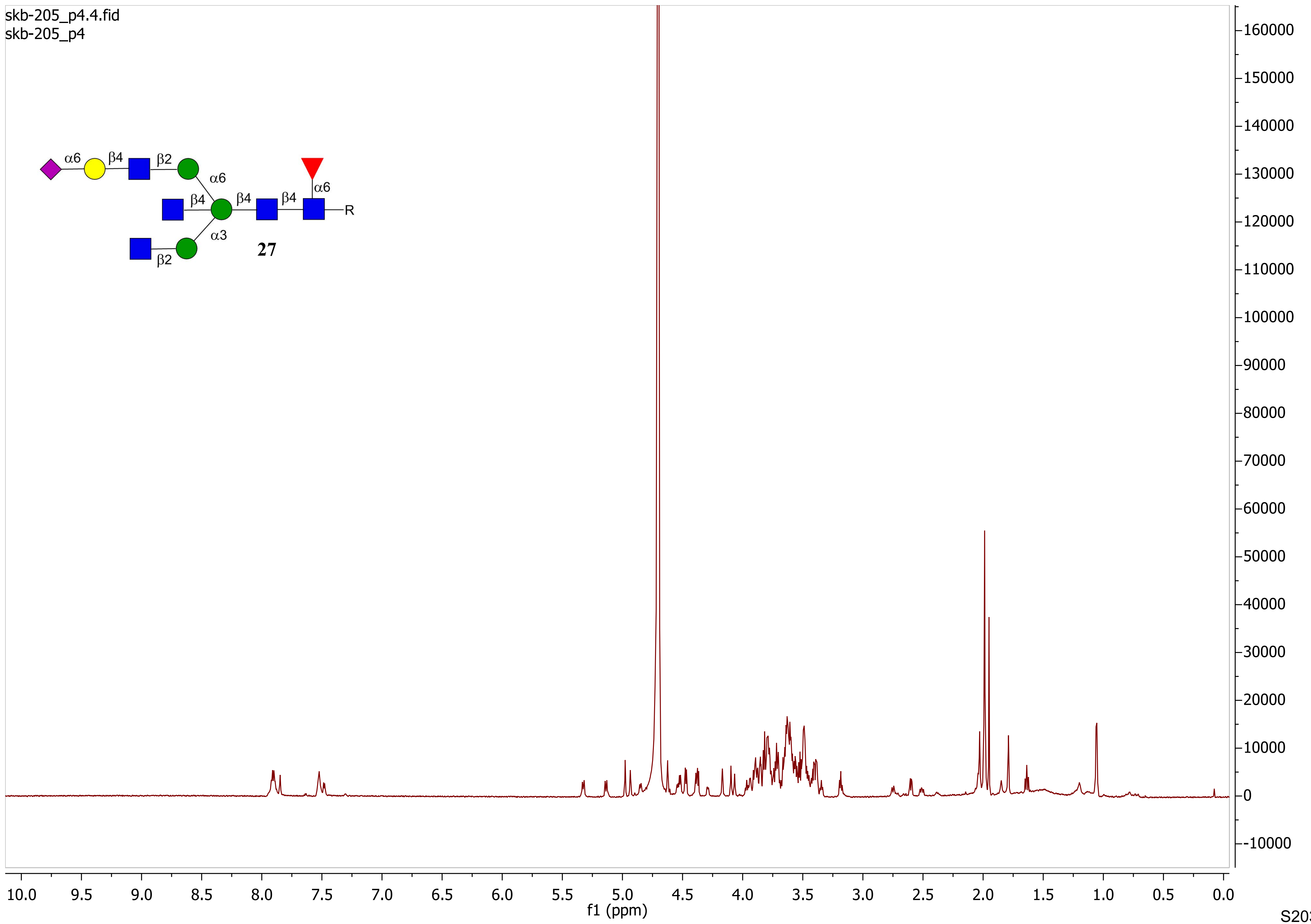
skb-205_p4.4.fid
skb-205_p4
α6
β4
β2
α6
β4
β4
β4
α6
R
α3
β2
27
f1 (ppm)

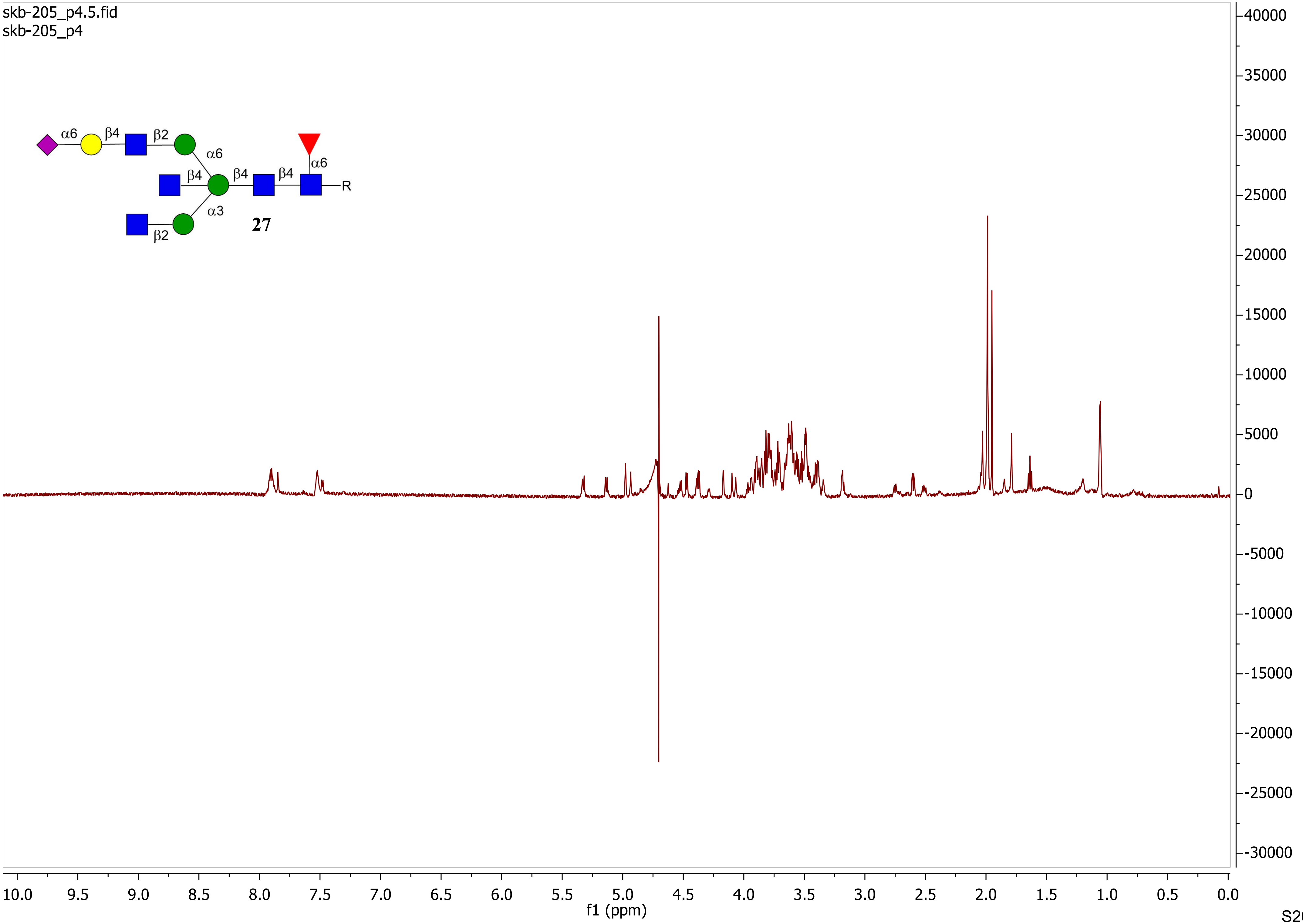
skb-205_p4.5.fid
skb-205_p4
α6
β4
β2
α6
β4
β4
β4
α6
R
α3
β2
27
40000
35000
30000
25000
20000
15000
10000
5000
0
-5000
-10000
-15000
-20000
-25000
-30000
10.0
9.5
9.0
8.5
8.0
7.5
7.0
6.5
6.0
5.5
5.0
4.5
4.0
3.5
3.0
2.5
2.0
1.5
1.0
0.5
0.0
f1 (ppm)

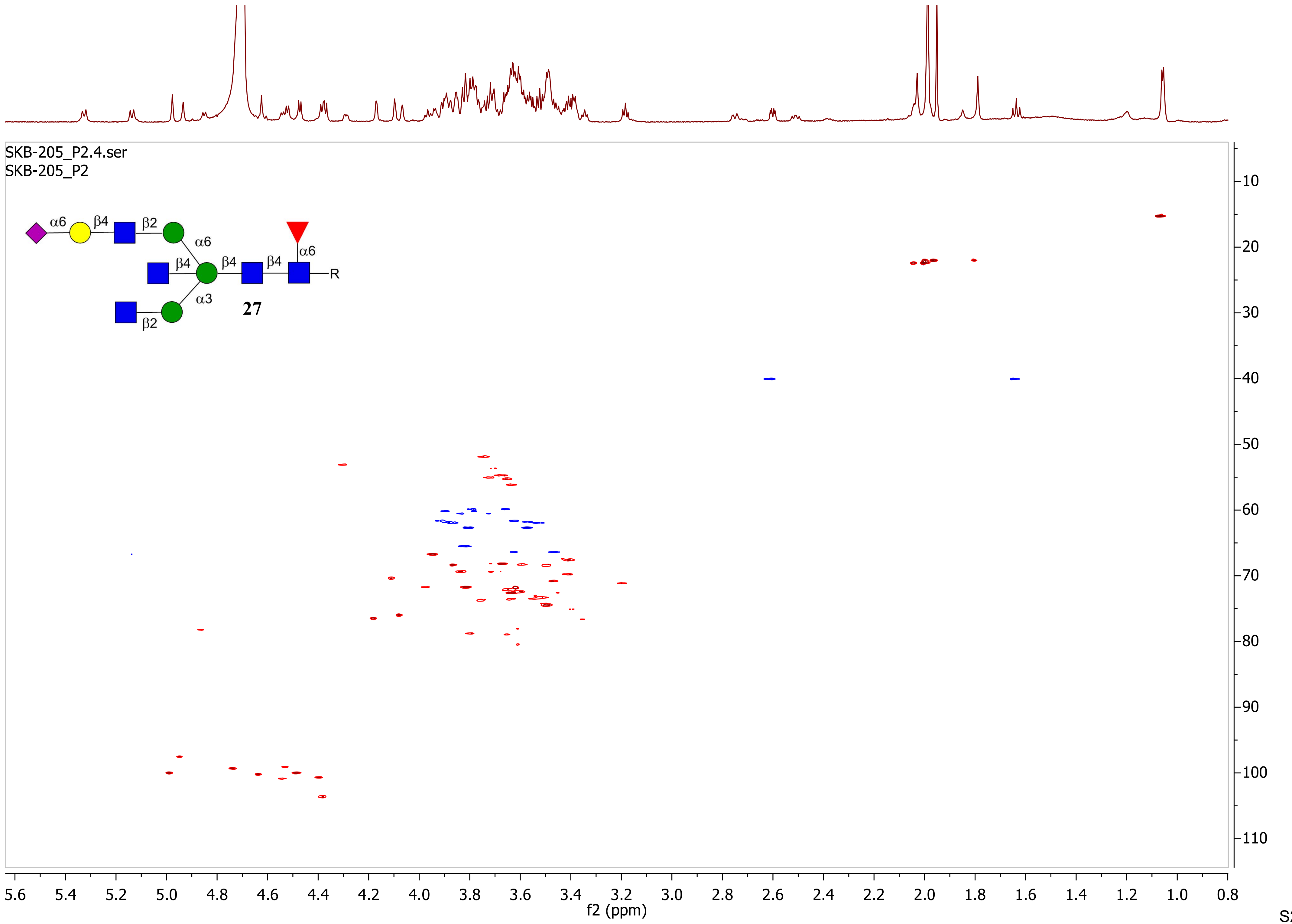
SKB-205_P2.4.ser
SKB-205_P2
α6
β4
β2
α6
β4
β4
β4
α6
R
α3
27
β2
10
20
30
40
50
60
70
80
90
100
110
f1 (ppm)
5.6
5.4
5.2
5.0
4.8
4.6
4.4
4.2
4.0
3.8
3.6
3.4
3.2
3.0
2.8
2.6
2.4
2.2
2.0
1.8
1.6
1.4
1.2
1.0
0.8
f2 (ppm)

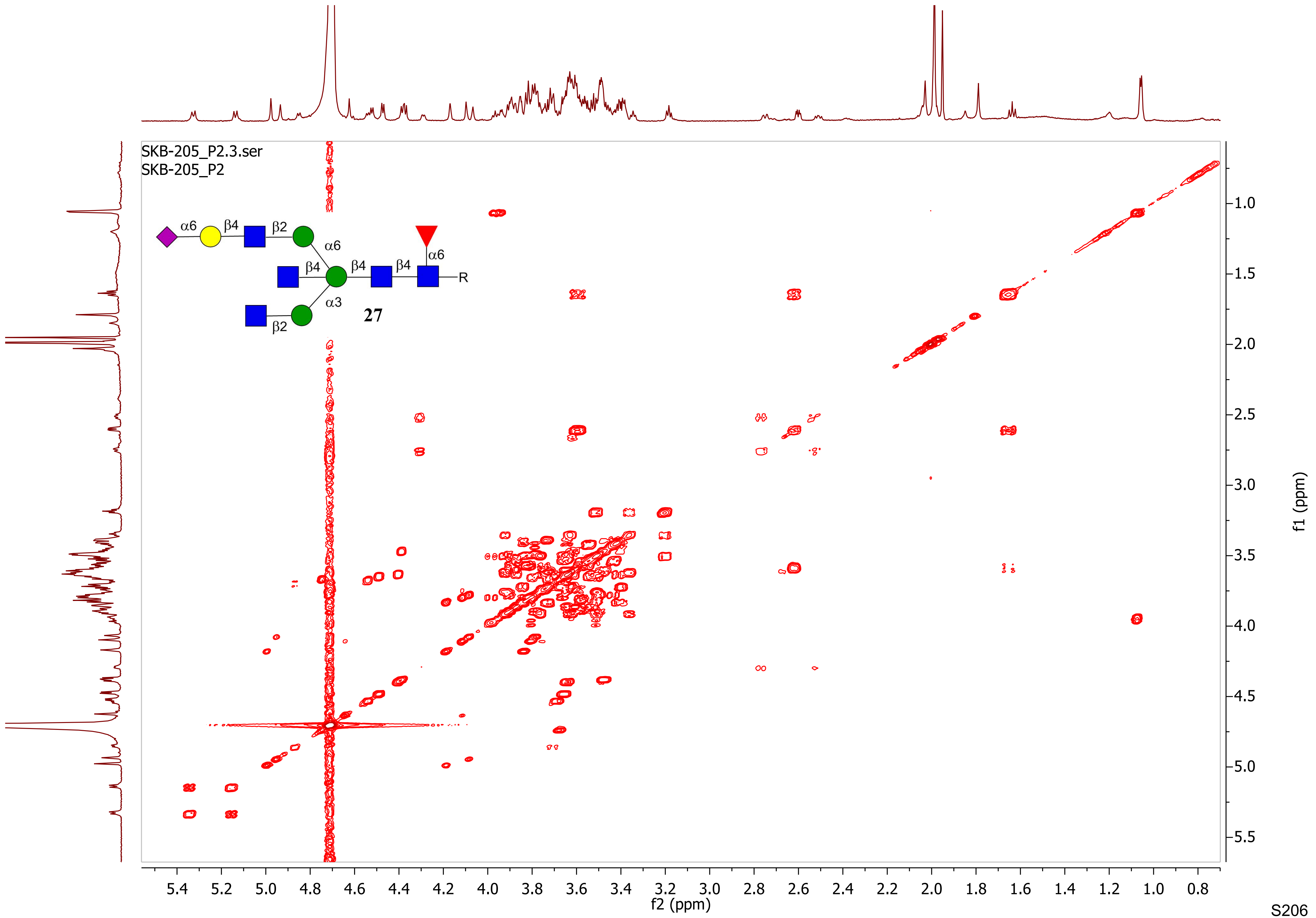

SKB-205_P2.3.ser
SKB-205_P2
α6
β4
β2
α6
β4
β4
β4
α6
R
α3
β2
27
f2 (ppm)
f1 (ppm)

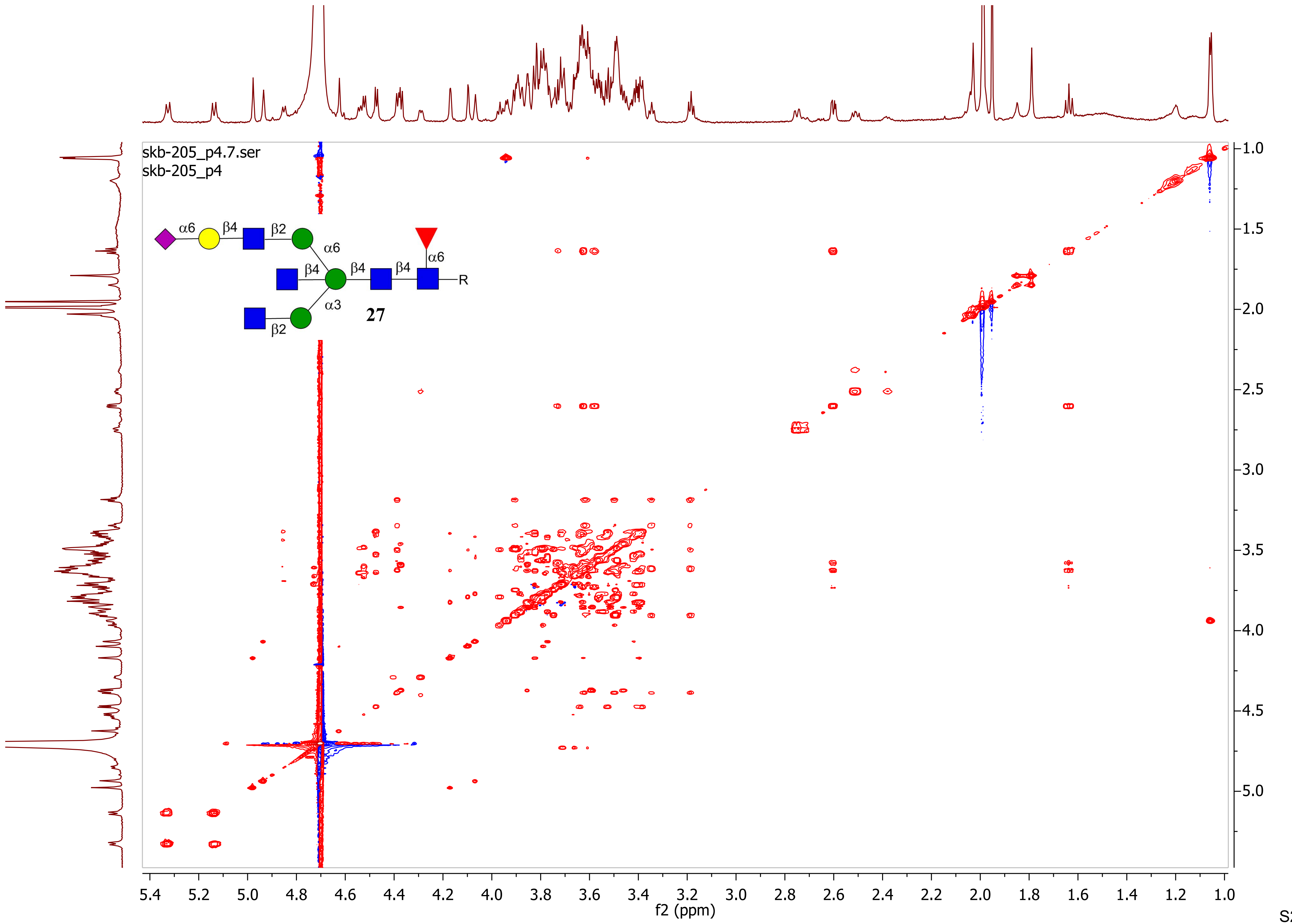
skb-205_p4.7.ser
skb-205_p4
α6
β4
β2
α6
β4
β4
β4
α6
R
α3
β2
27
f2 (ppm)
f1 (ppm)

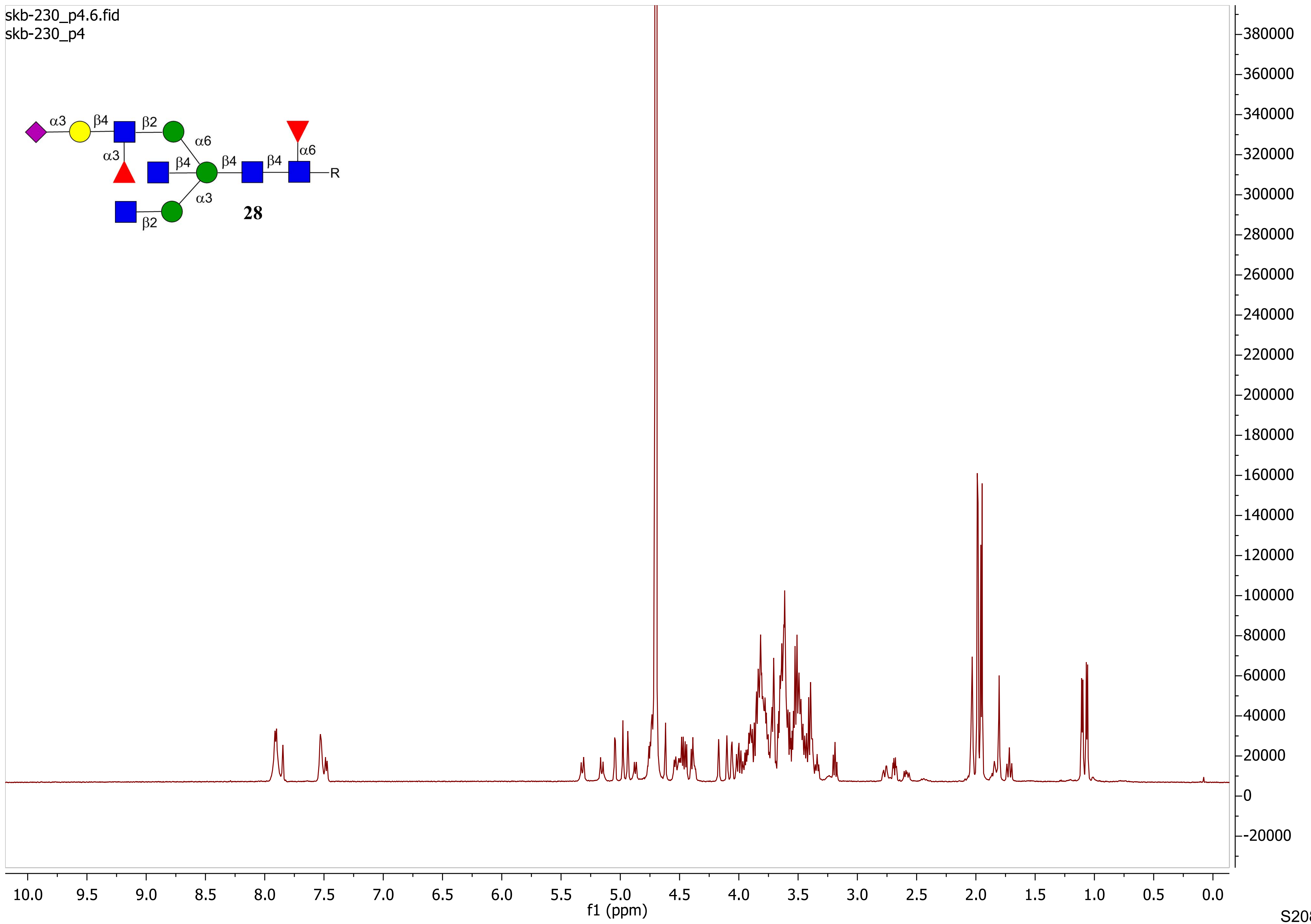

skb-230_p4.6.fid
skb-230_p4
α3
β4
β2
α6
α3
β4
β4
β4
α6
R
α3
β2
28
f1 (ppm)

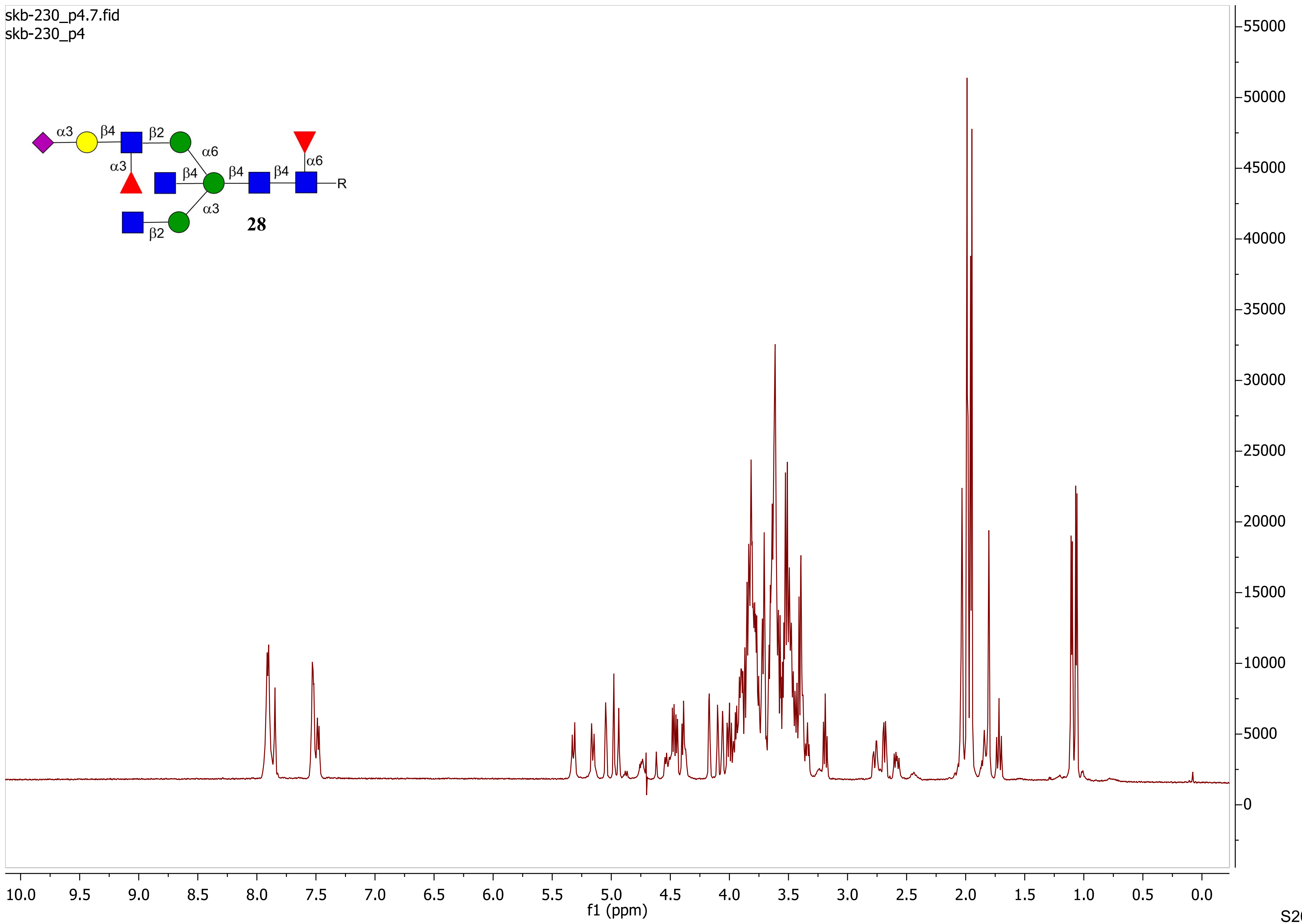

skb-230_p4.7.fid
skb-230_p4
α3
β4
β2
α6
α3
β4
β4
β4
α6
R
α3
β2
28
55000
50000
45000
40000
35000
30000
25000
20000
15000
10000
5000
0
10.0
9.5
9.0
8.5
8.0
7.5
7.0
6.5
6.0
5.5
5.0
4.5
4.0
3.5
3.0
2.5
2.0
1.5
1.0
0.5
0.0
f1 (ppm)

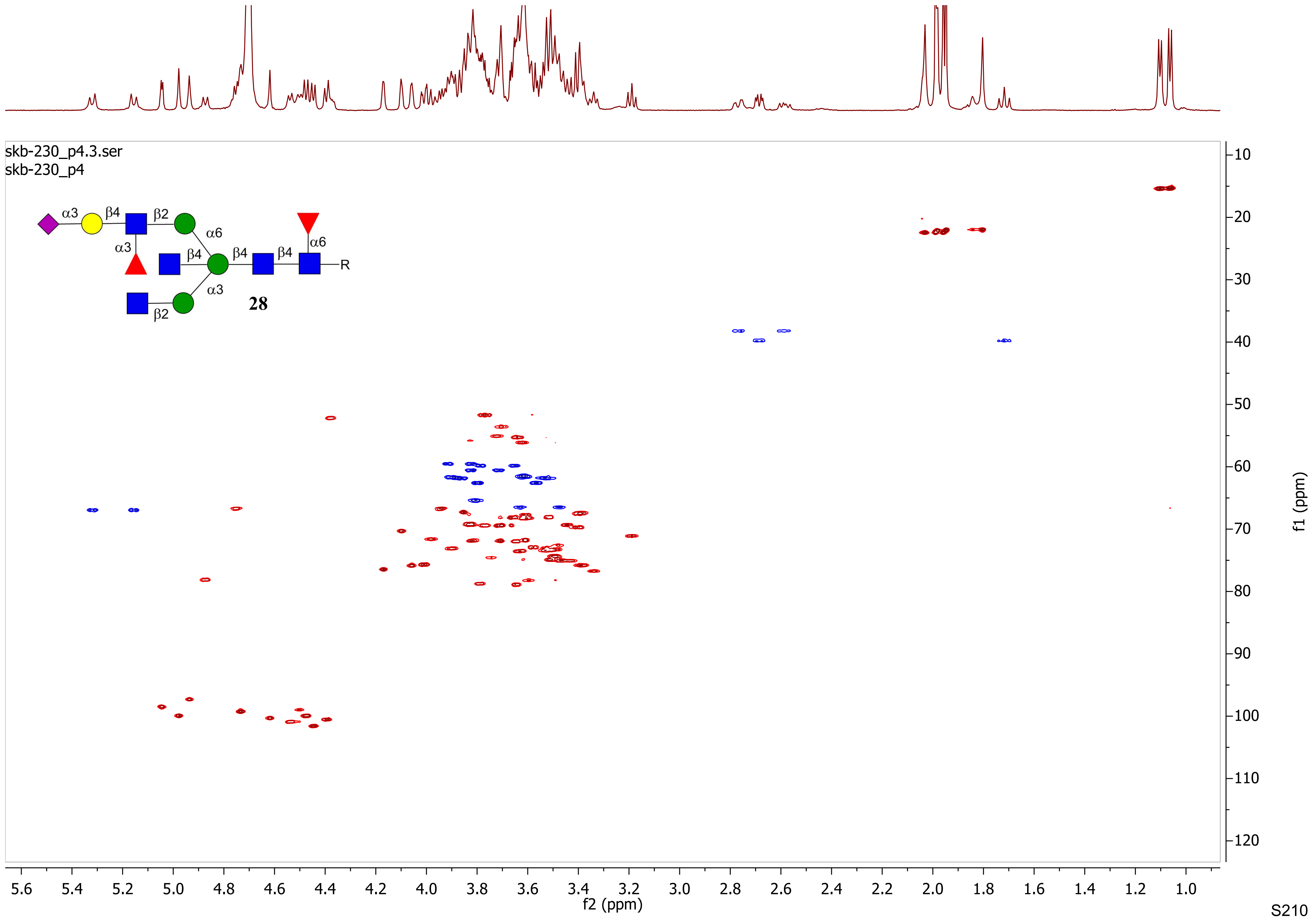
skb-230_p4.3.ser
skb-230_p4
α3
β4
β2
α6
α3
β4
β4
β4
α6
R
α3
β2
28
f1 (ppm)
f2 (ppm)

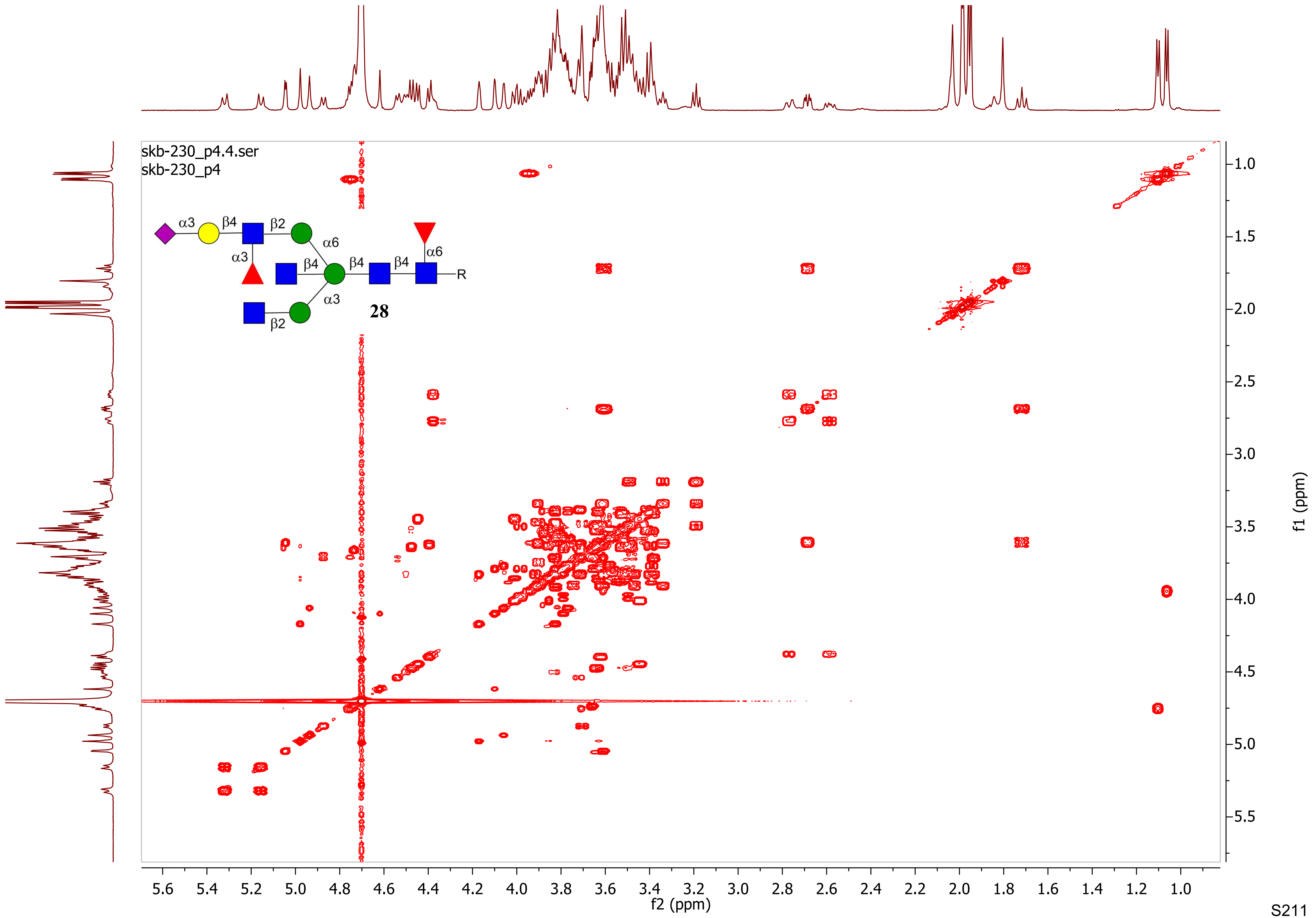

skb-230_p4.4.ser
skb-230_p4
α3
β4
β2
α6
α3
β4
β4
β4
α6
R
α3
β2
28
f2 (ppm)
f1 (ppm)

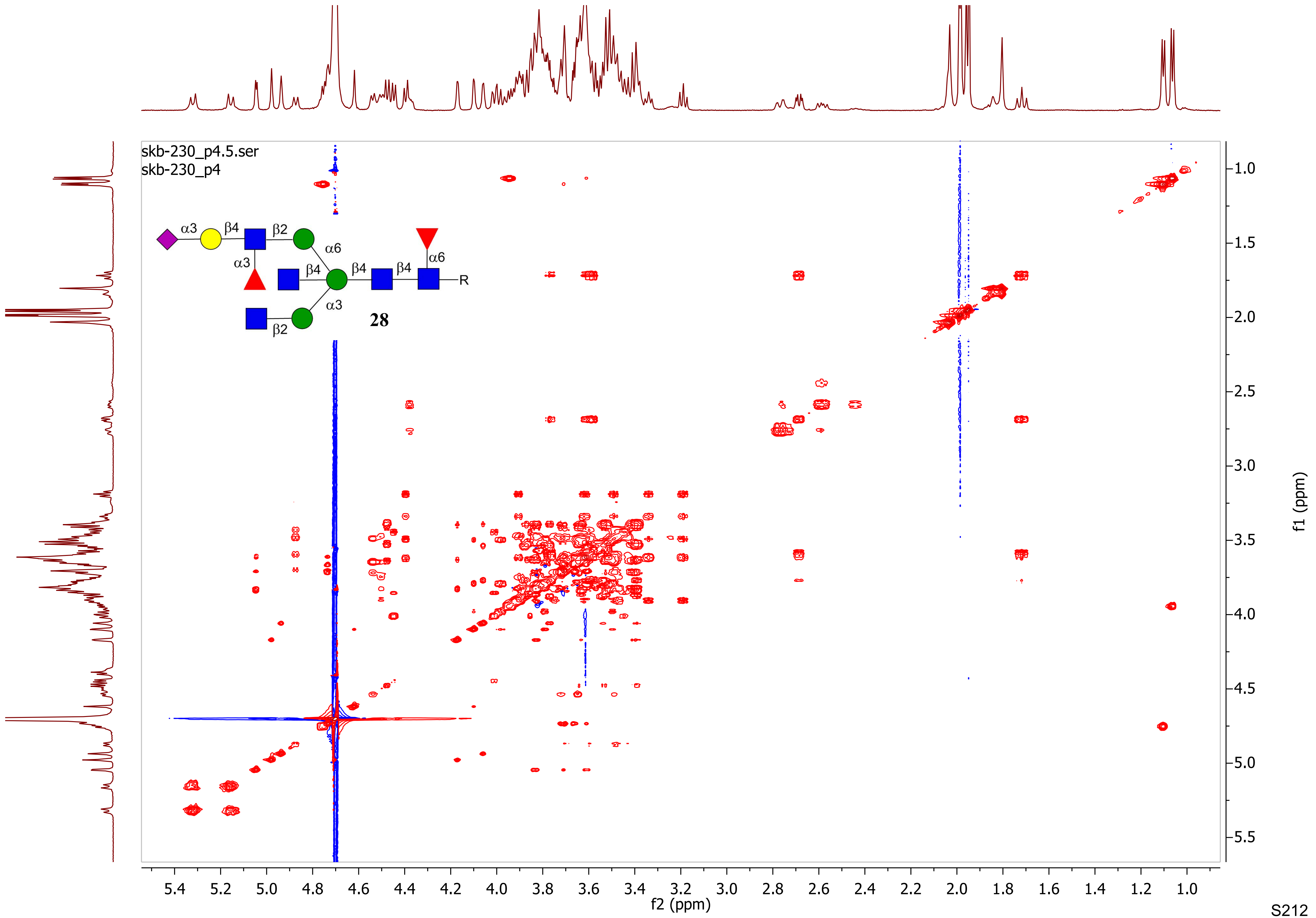
skb-230_p4.5.ser
skb-230_p4
α3
β4
β2
α6
α3
β4
β4
β4
α6
R
α3
β2
28
f2 (ppm)
f1 (ppm)

skb-229_P4_3.1.fid
skb-229_P4_3

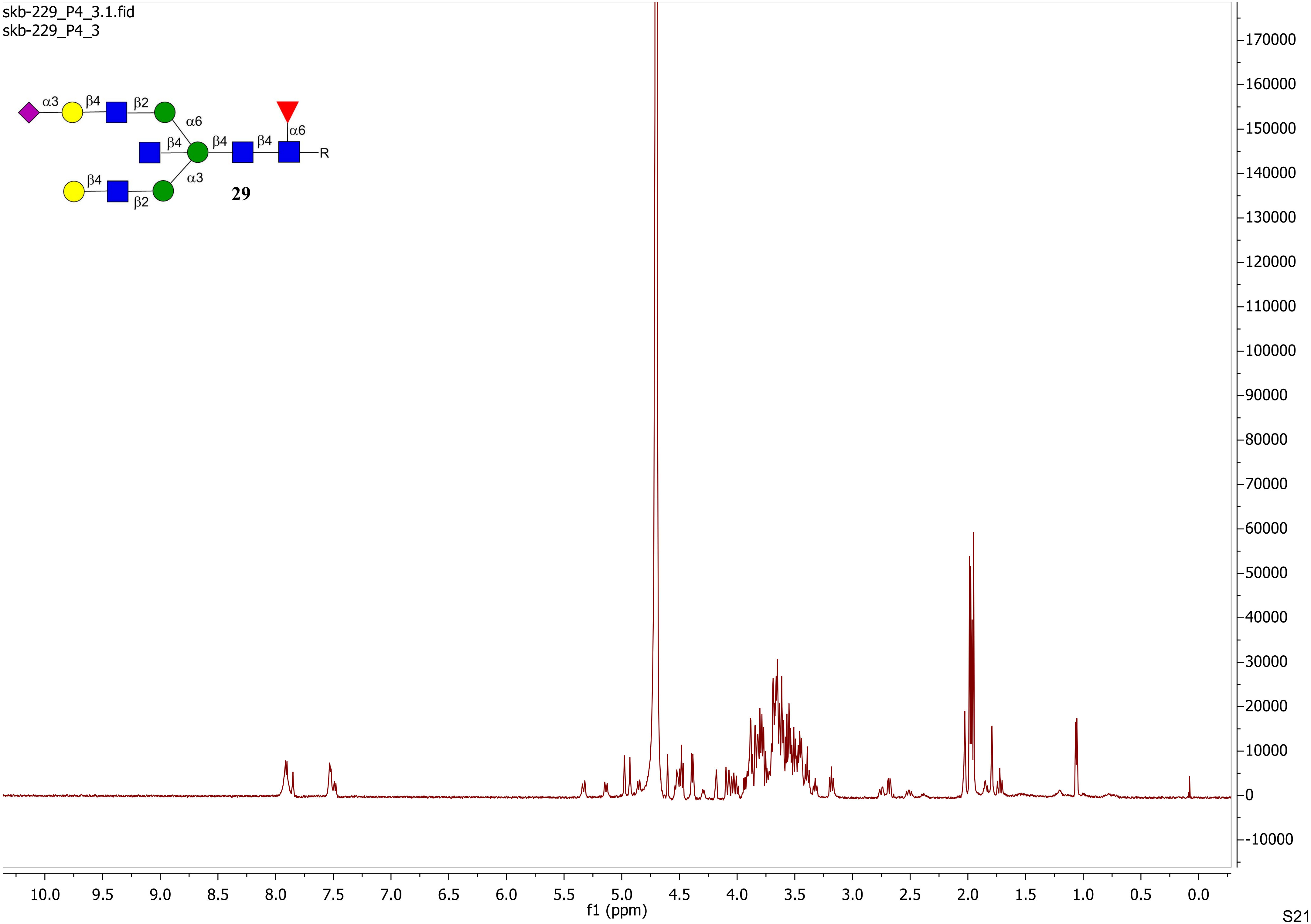

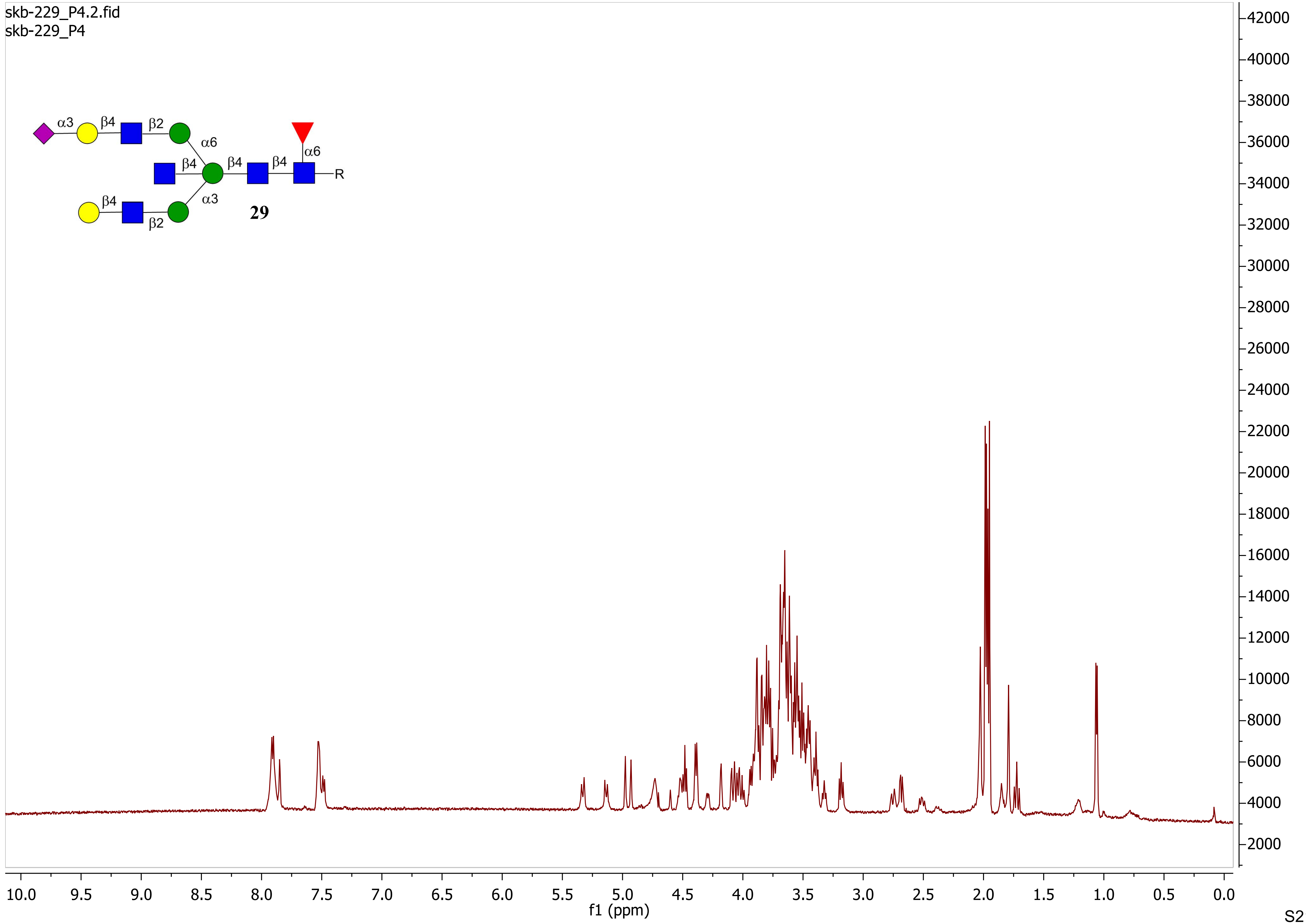

skb-229_P4.2.fid
skb-229_P4
α3
β4
β2
α6
β4
β4
β4
α6
R
β4
α3
β2
29
f1 (ppm)

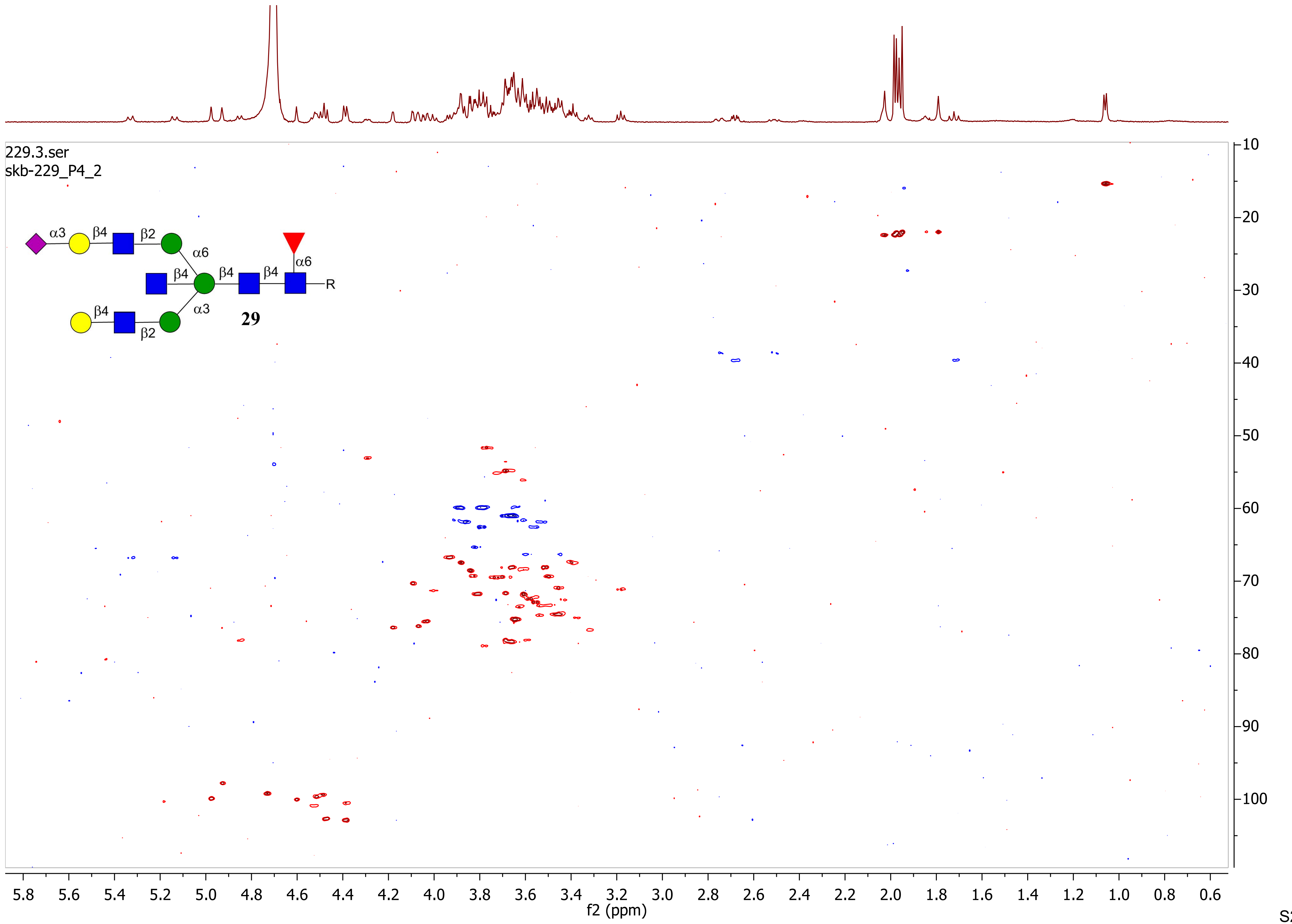

229.3.ser
skb-229_P4_2
α3
β4
β2
α6
β4
β4
β4
α6
R
α3
β4
β2
29
f2 (ppm)
f1 (ppm)

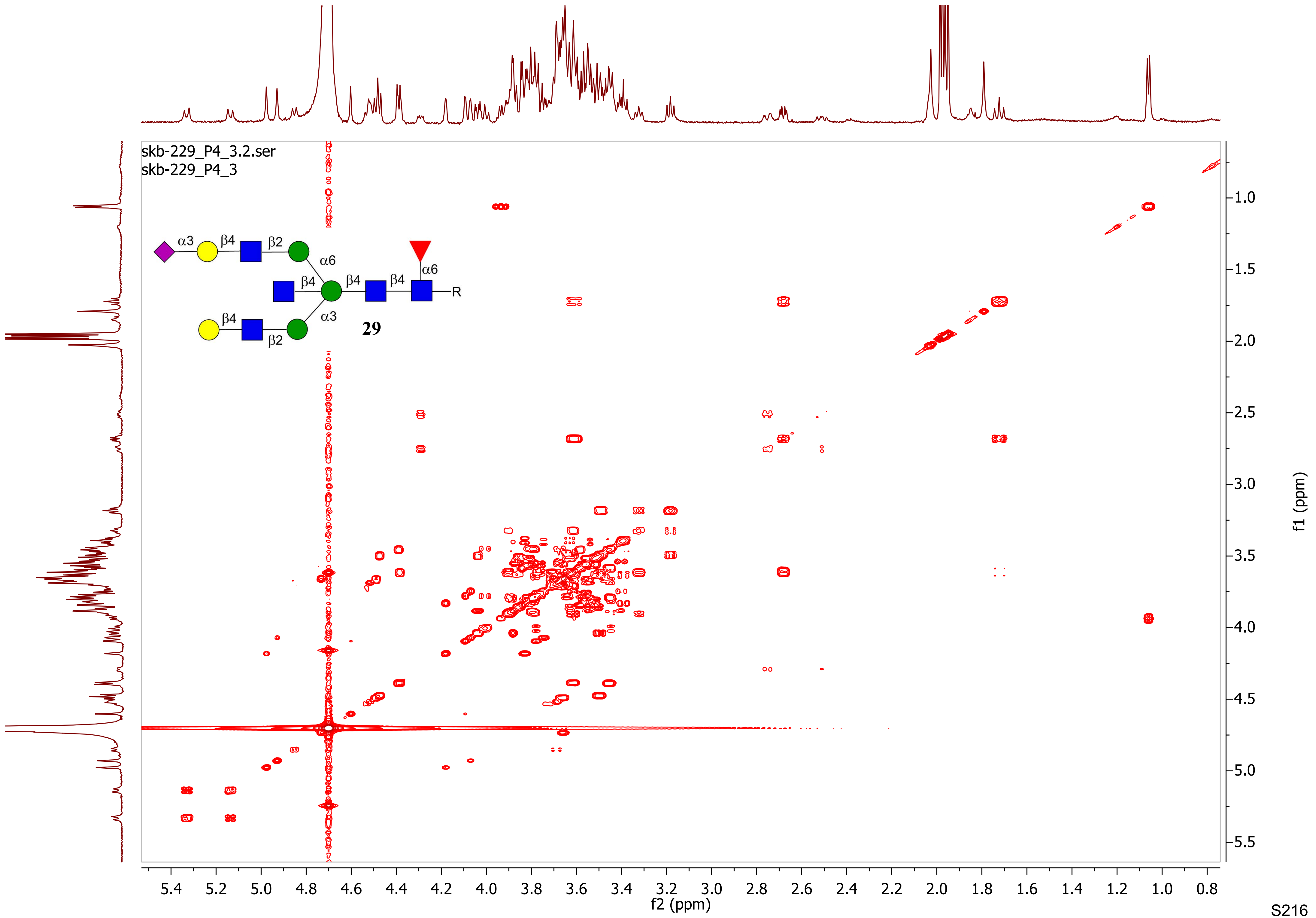
skb-229_P4_3.2.ser
skb-229_P4_3
α3
β4
β2
α6
β4
β4
β4
α6
R
β4
α3
β2
29
f2 (ppm)
f1 (ppm)
5.4
5.2
5.0
4.8
4.6
4.4
4.2
4.0
3.8
3.6
3.4
3.2
3.0
2.8
2.6
2.4
2.2
2.0
1.8
1.6
1.4
1.2
1.0
0.8
1.0
1.5
2.0
2.5
3.0
3.5
4.0
4.5
5.0
5.5

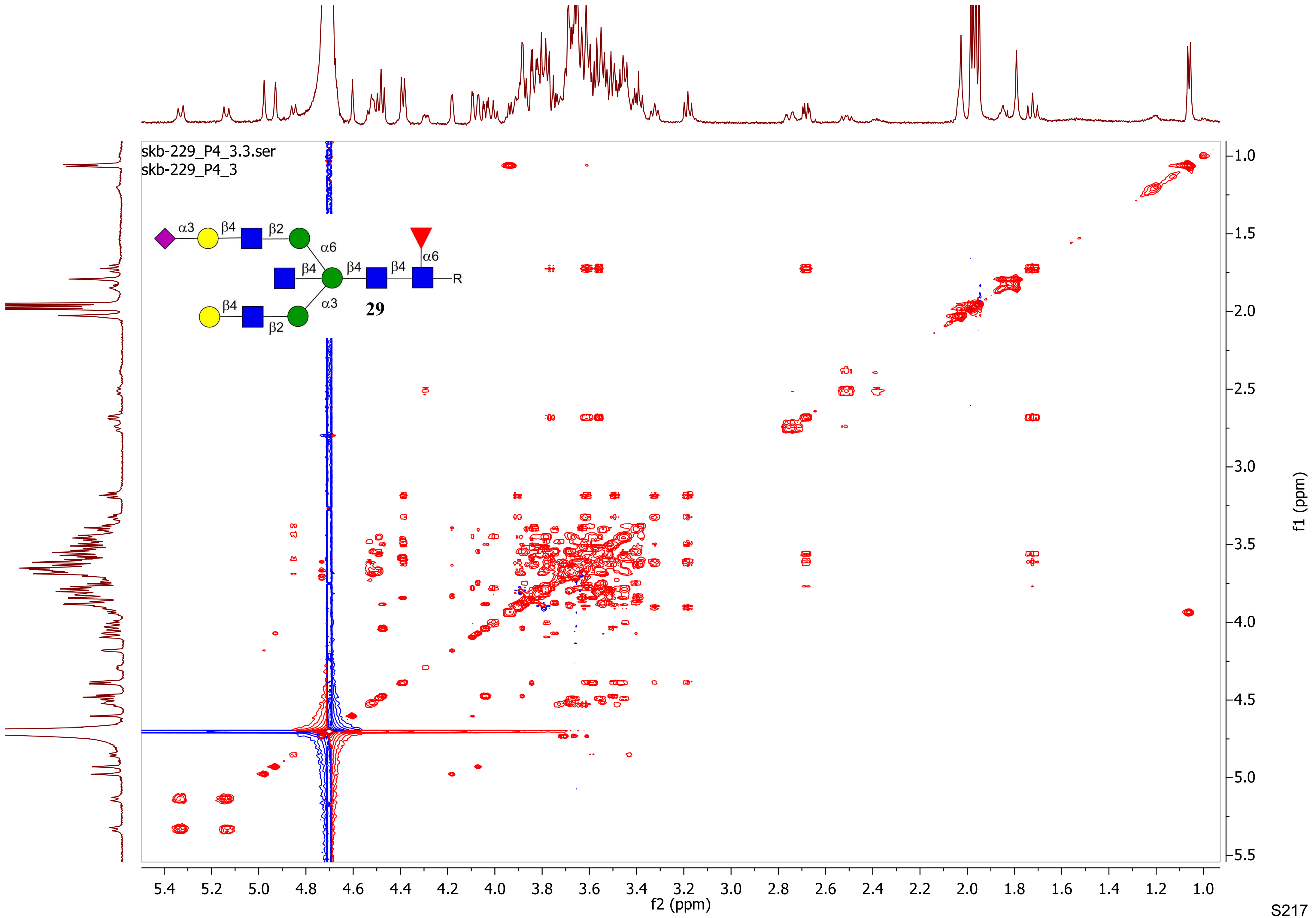

skb-229_P4_3.3.ser
skb-229_P4_3
α3
β4
β2
α6
β4
β4
β4
α6
R
α3
β4
β2
29
f2 (ppm)
f1 (ppm)

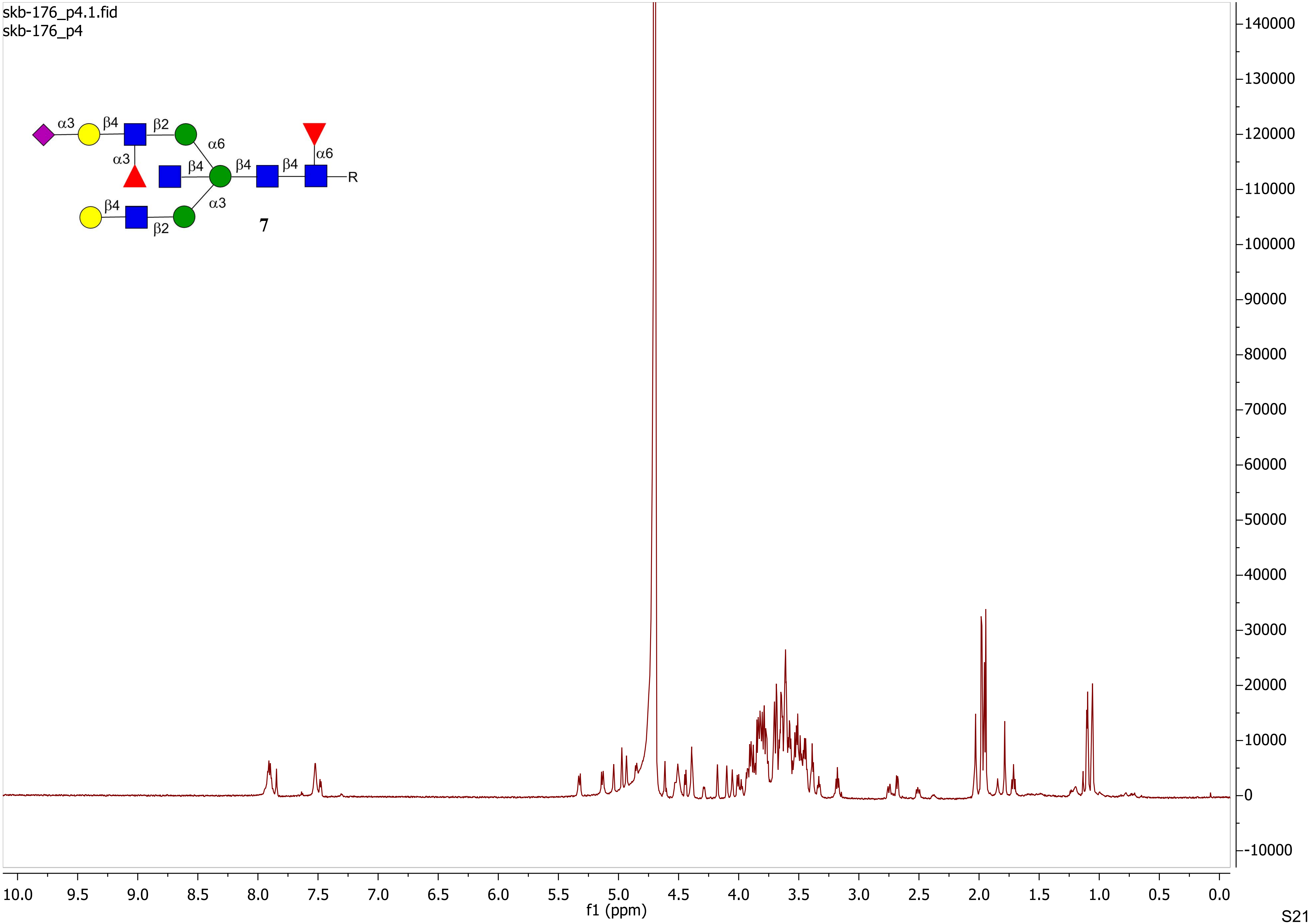
skb-176_p4.1.fid
skb-176_p4
α3
β4
β2
α6
α3
β4
β4
β4
α6
R
α3
β4
β2
7
f1 (ppm)

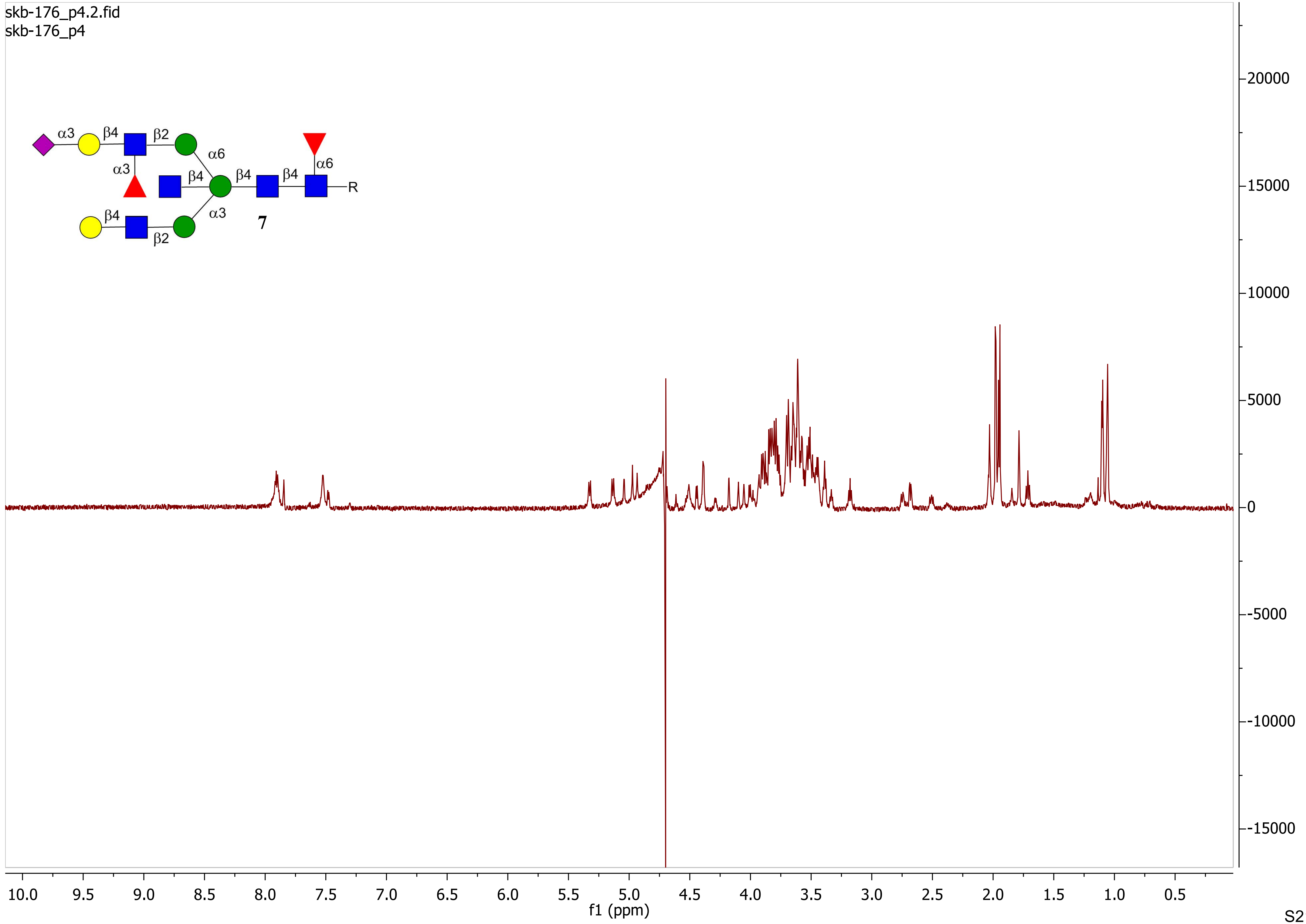

skb-176_p4.2.fid
skb-176_p4
α3
β4
β2
α6
α3
β4
β4
β4
α6
R
β4
β2
α3
7
f1 (ppm)

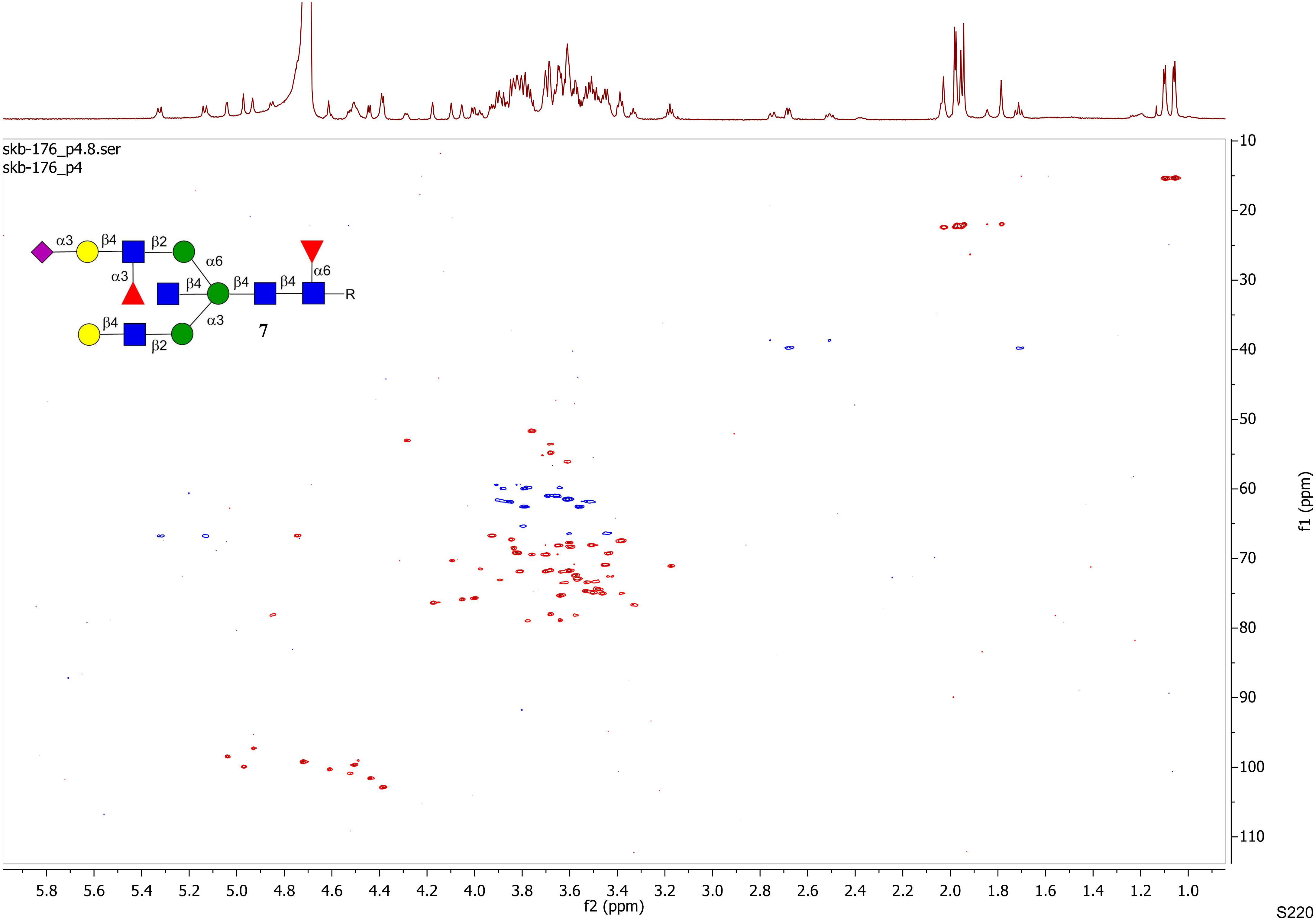
skb-176_p4.8.ser
skb-176_p4
α3
β4
β2
α6
α3
β4
β4
β4
α6
R
β4
α3
β2
7
f2 (ppm)
f1 (ppm)

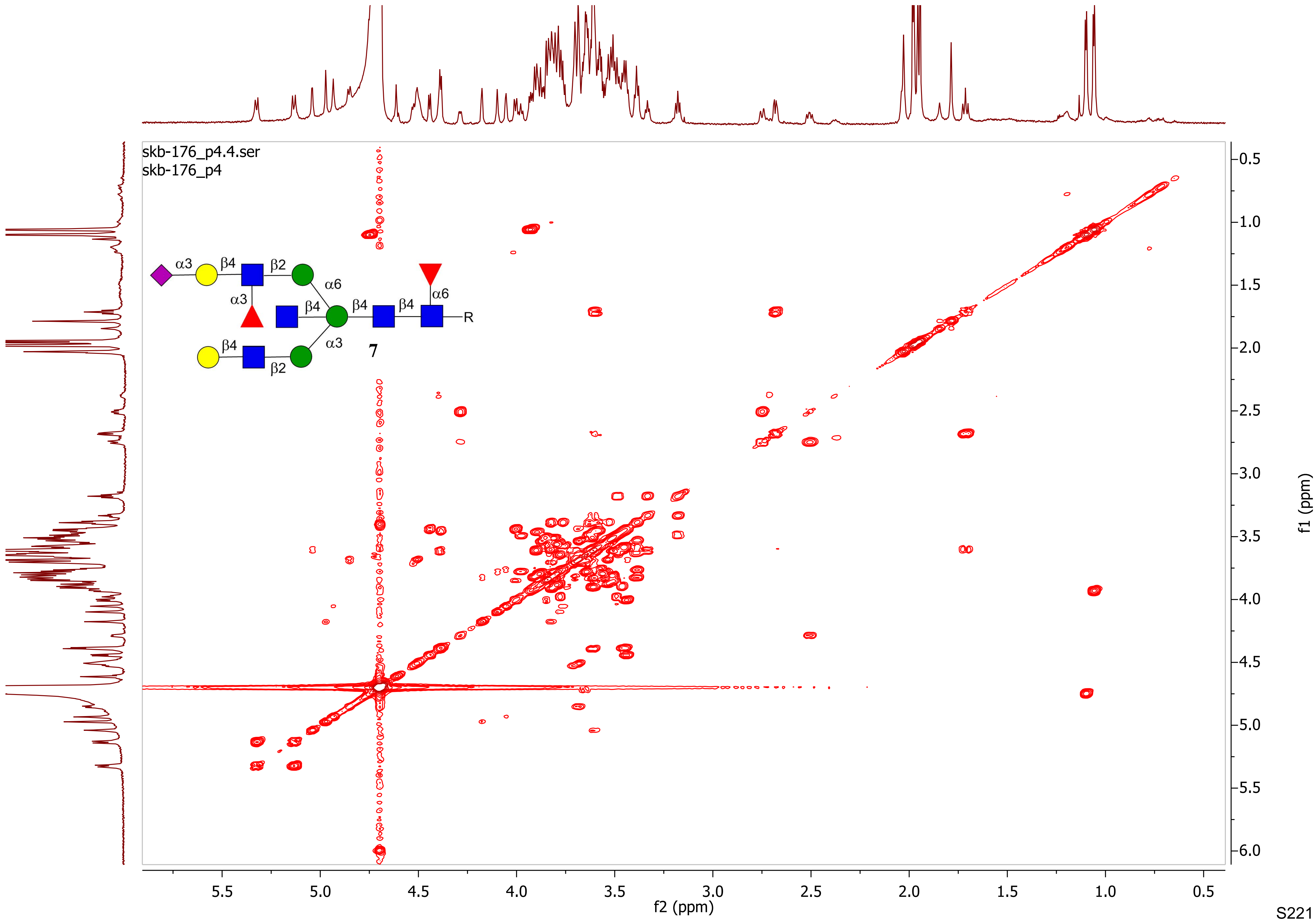

skb-176_p4.4.ser
skb-176_p4
α3
β4
β2
α6
α3
β4
β4
β4
α6
R
β4
α3
β2
7
f2 (ppm)
f1 (ppm)

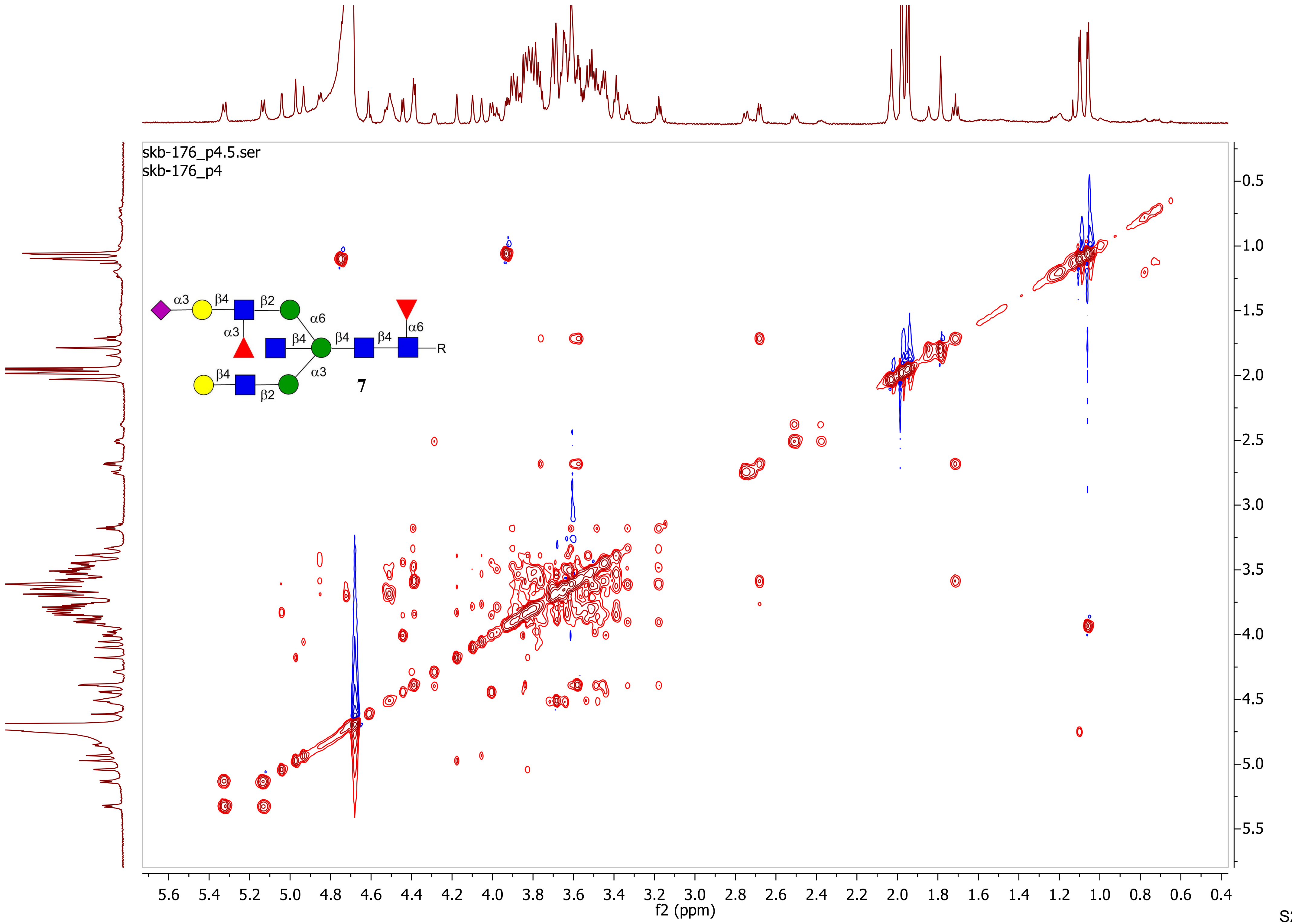
skb-176_p4.5.ser
skb-176_p4
α3
β4
β2
α6
α3
β4
β4
β4
α6
R
β4
β2
α3
7
f2 (ppm)
f1 (ppm)

skb-187_P4900.3.fid
skb-87_P4

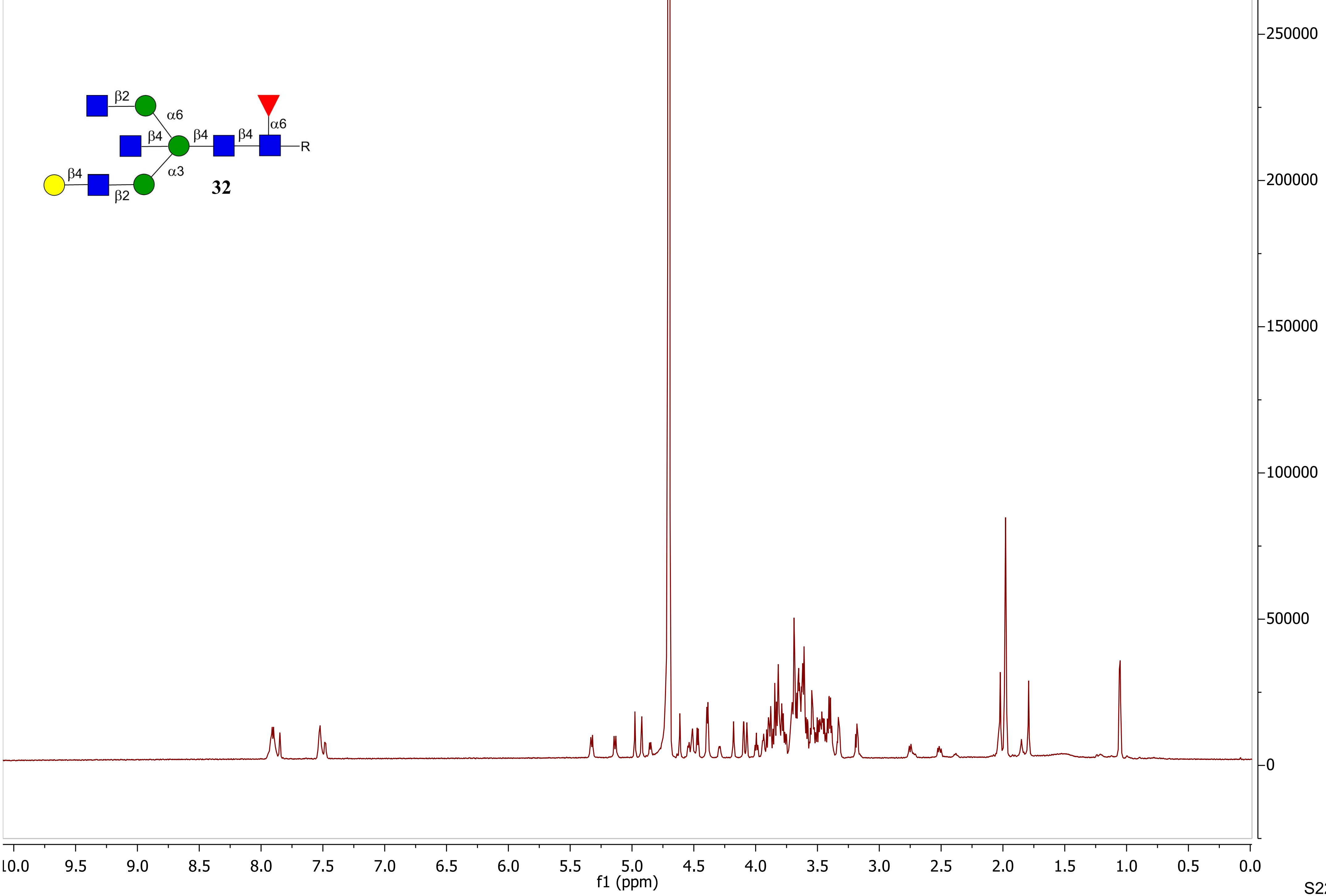

32

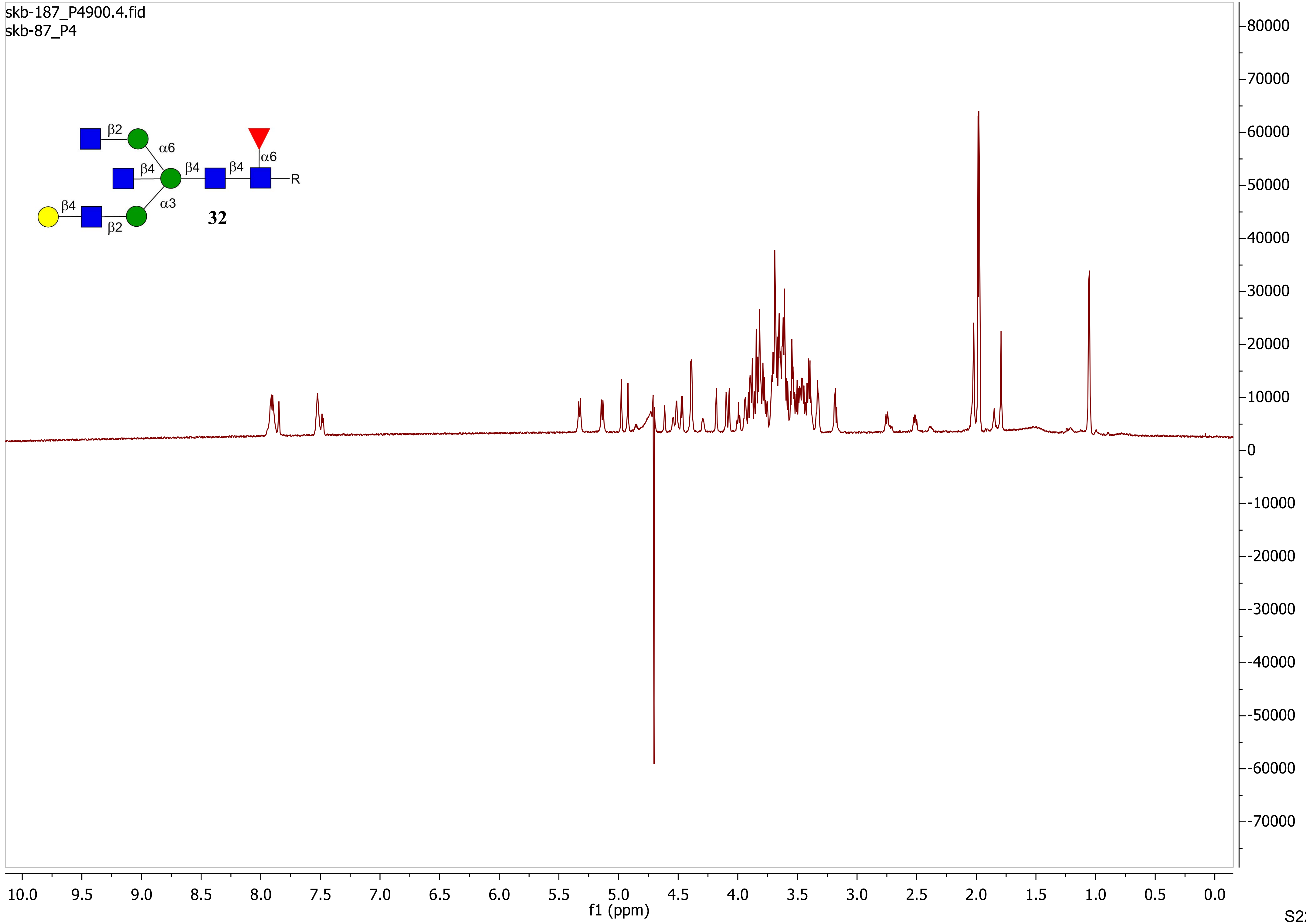
skb-187_P4900.4.fid
skb-87_P4
β2
α6
β4
β4
β4
α6
R
β4
α3
β2
32
80000
70000
60000
50000
40000
30000
20000
10000
0
-10000
-20000
-30000
-40000
-50000
-60000
-70000
10.0
9.5
9.0
8.5
8.0
7.5
7.0
6.5
6.0
5.5
5.0
4.5
4.0
3.5
3.0
2.5
2.0
1.5
1.0
0.5
0.0
f1 (ppm)

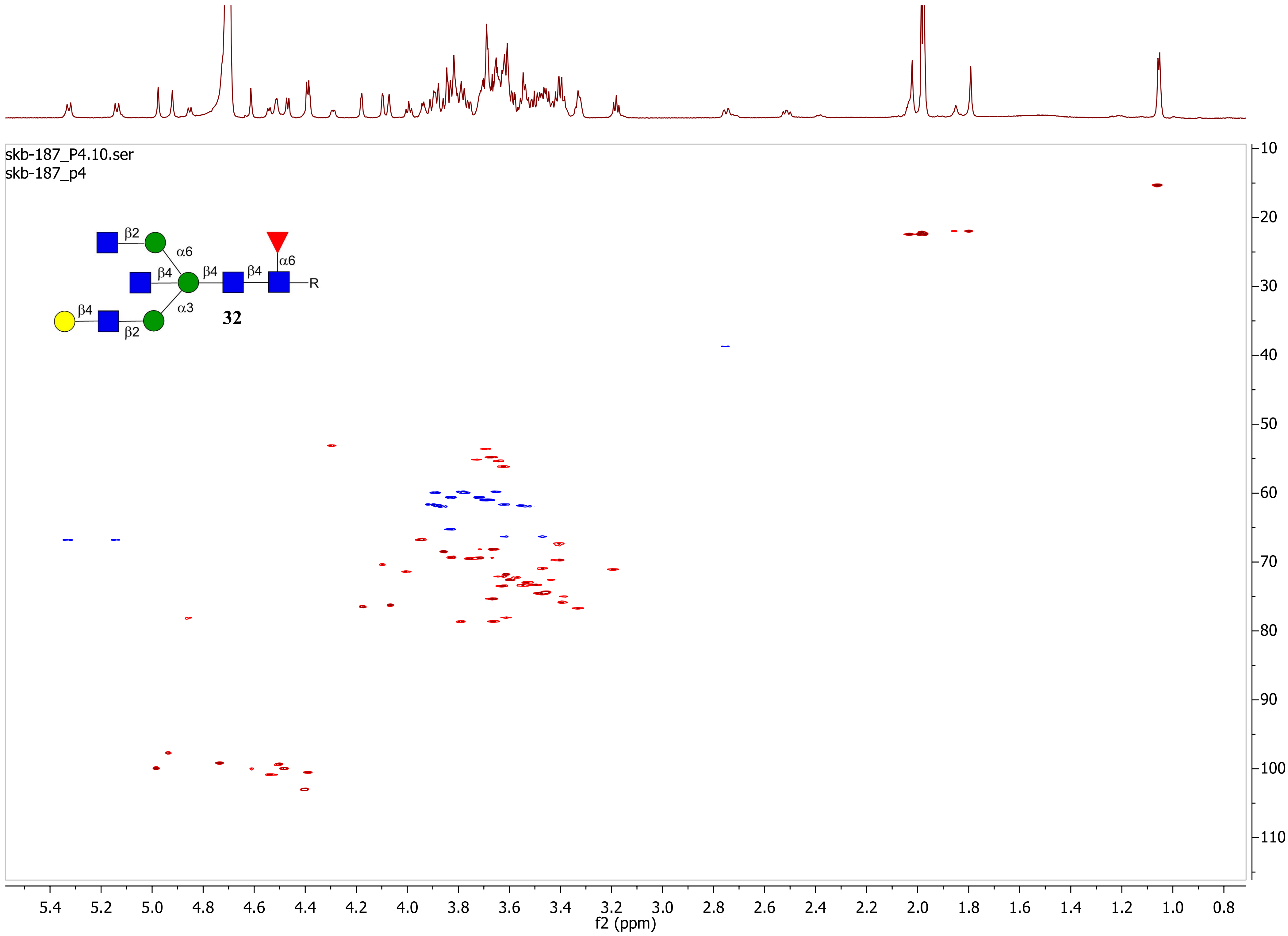

skb-187_P4.10.ser
skb-187_p4
β2
α6
β4
β4
β4
α6
R
α3
β4
β2
32
f1 (ppm)
f2 (ppm)

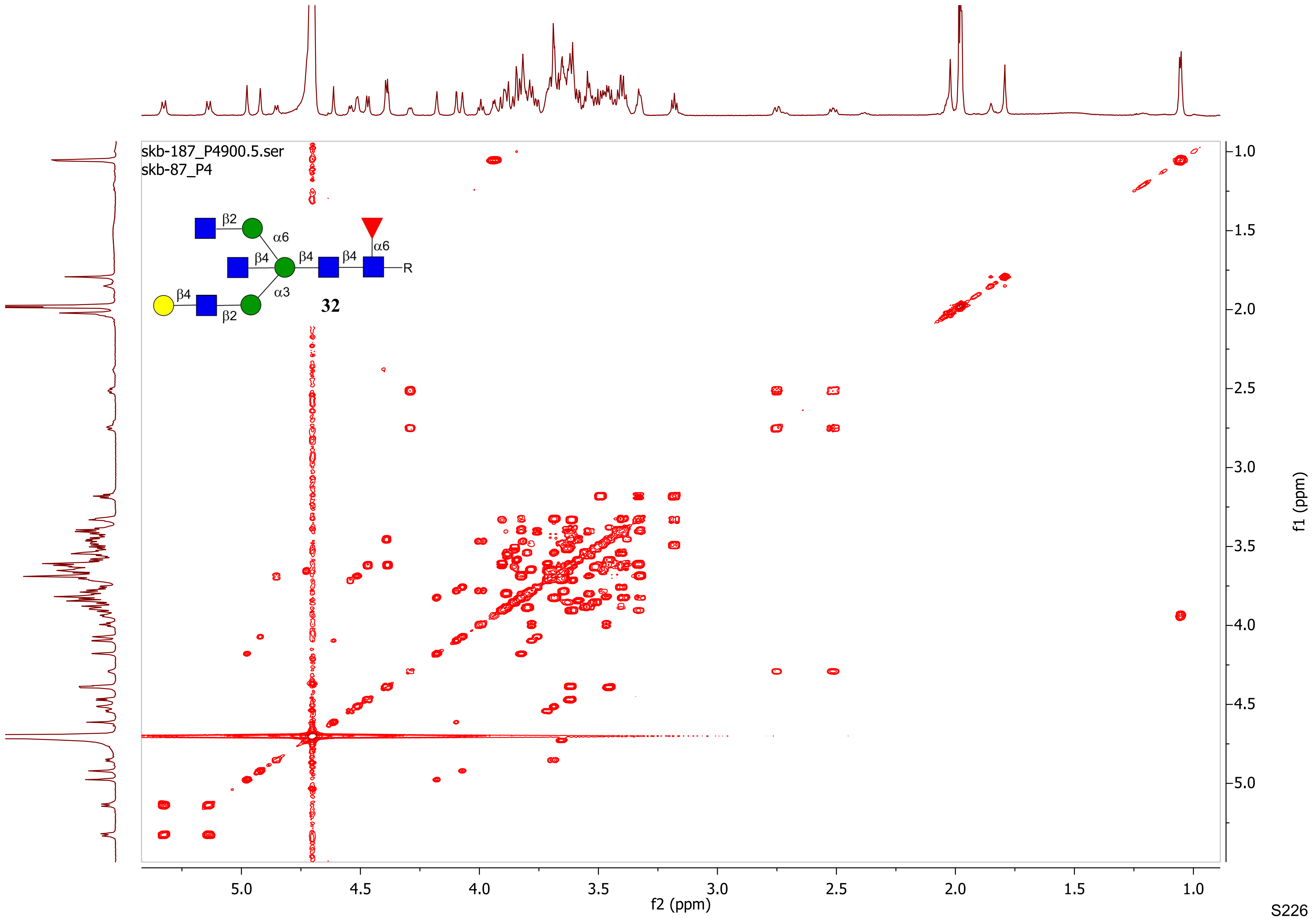
skb-187_P4900.5.ser
skb-87_P4
β2
α6
β4
β4
β4
α6
R
β4
α3
β2
32
f2 (ppm)
f1 (ppm)

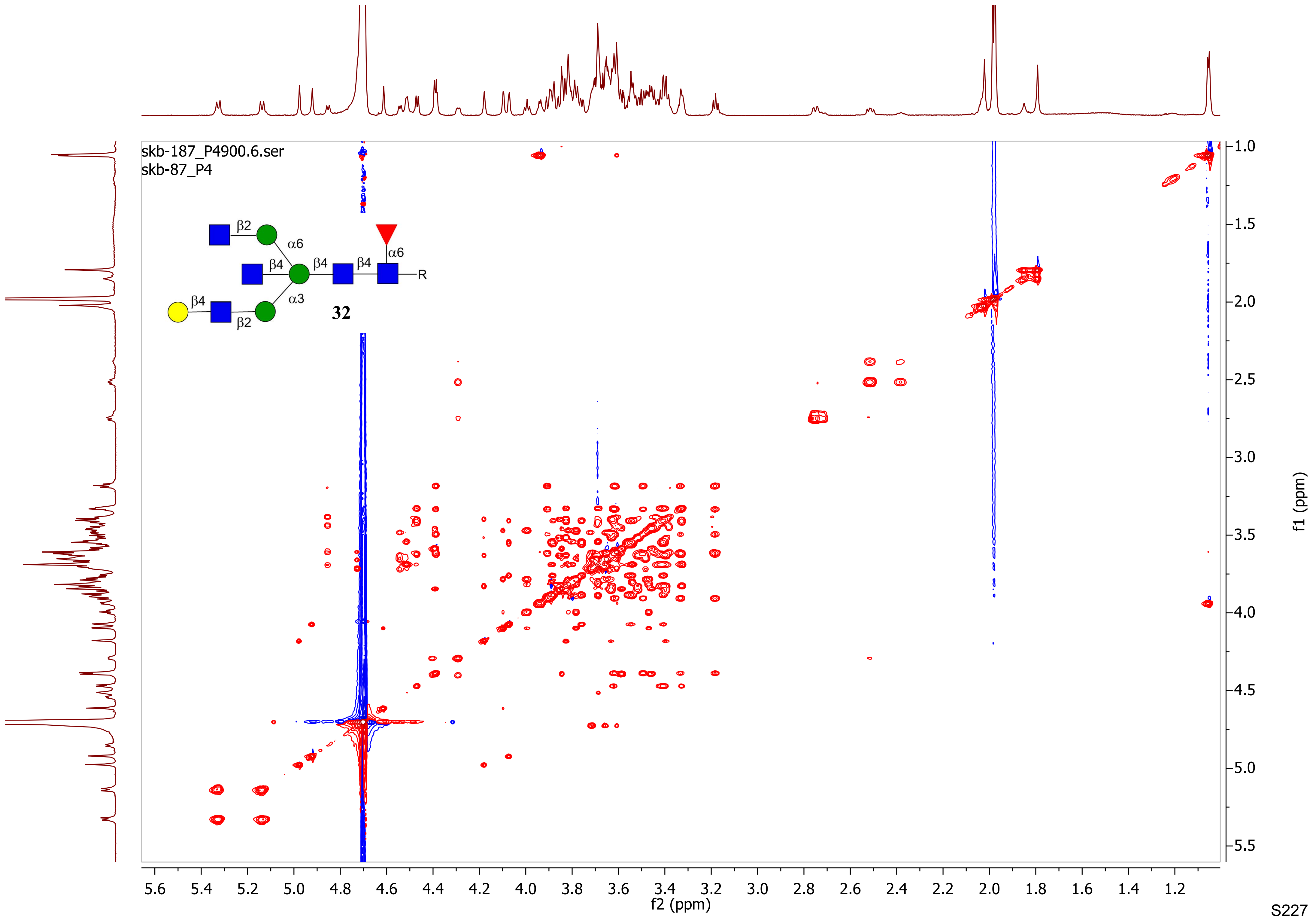
skb-187_P4900.6.ser
skb-87_P4
β2
α6
β4
β4
β4
α6
R
β4
α3
β2
32
f2 (ppm)
f1 (ppm)

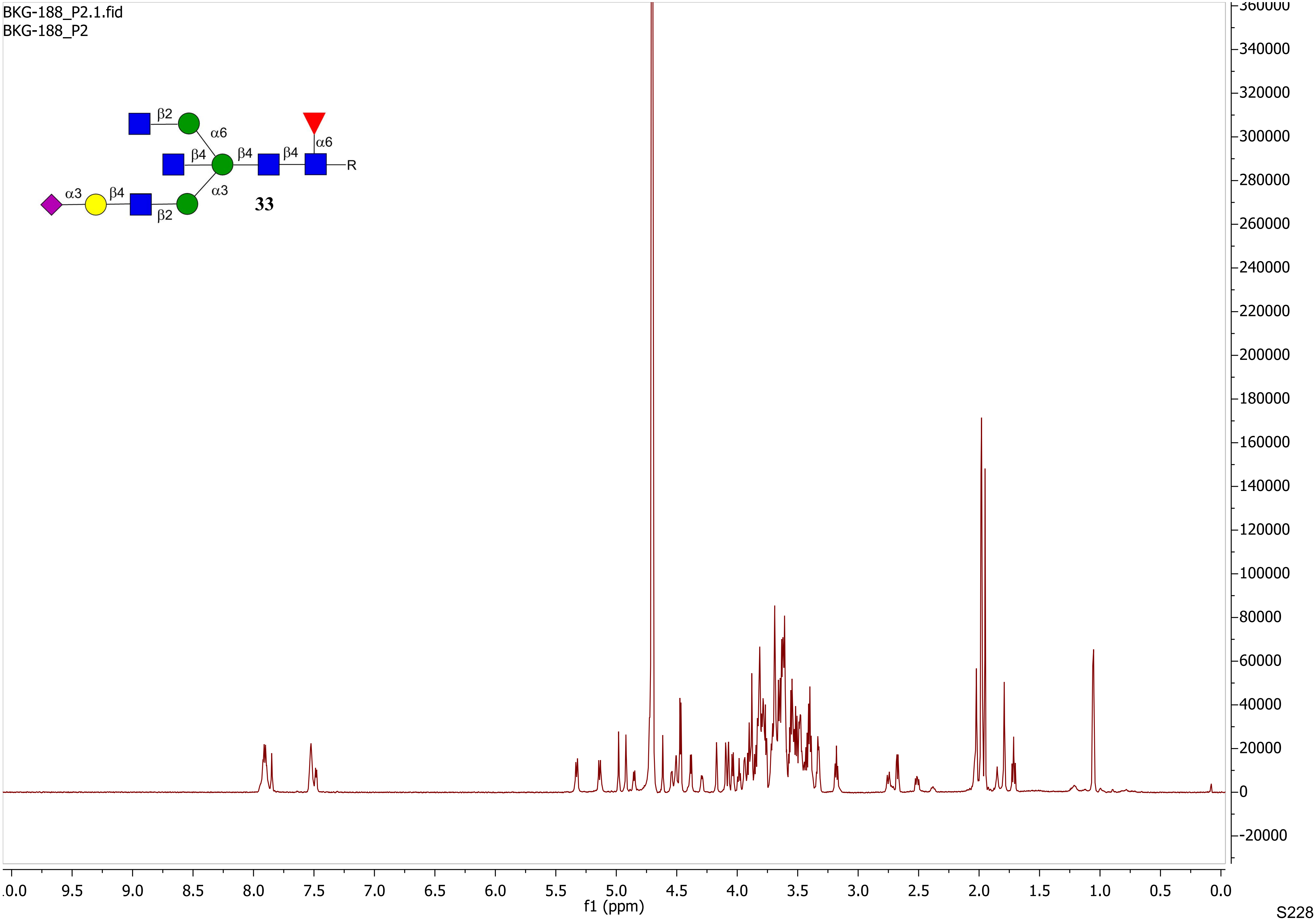
BKG-188_P2.1.fid
BKG-188_P2
β2
α6
β4
β4
β4
α6
R
α3
β4
α3
β2
33
f1 (ppm)
10.0 9.5 9.0 8.5 8.0 7.5 7.0 6.5 6.0 5.5 5.0 4.5 4.0 3.5 3.0 2.5 2.0 1.5 1.0 0.5 0.0
360000 340000 320000 300000 280000 260000 240000 220000 200000 180000 160000 140000 120000 100000 80000 60000 40000 20000 0 -20000

BKG-188_P2.2.fid
BKG-188_P2

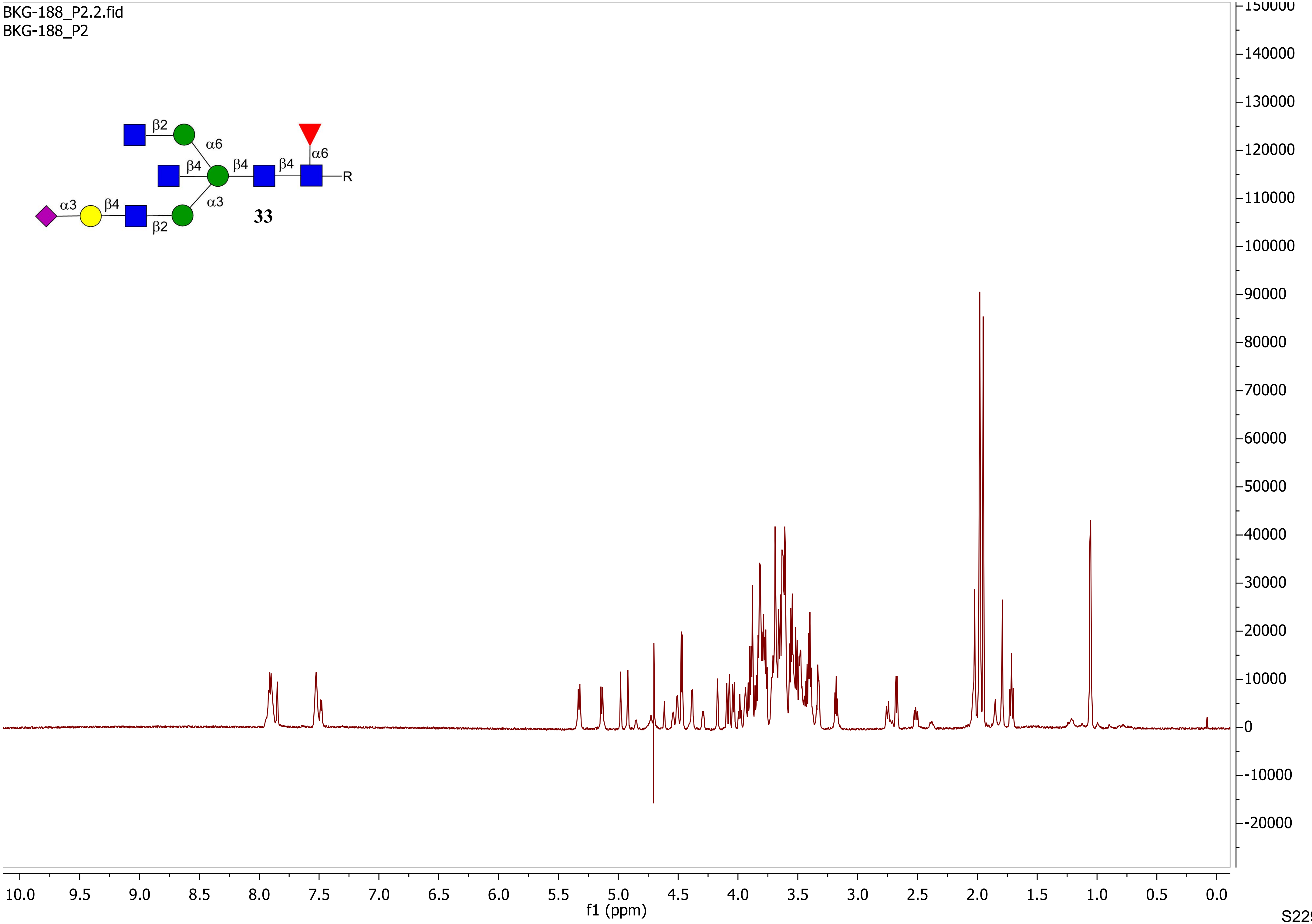

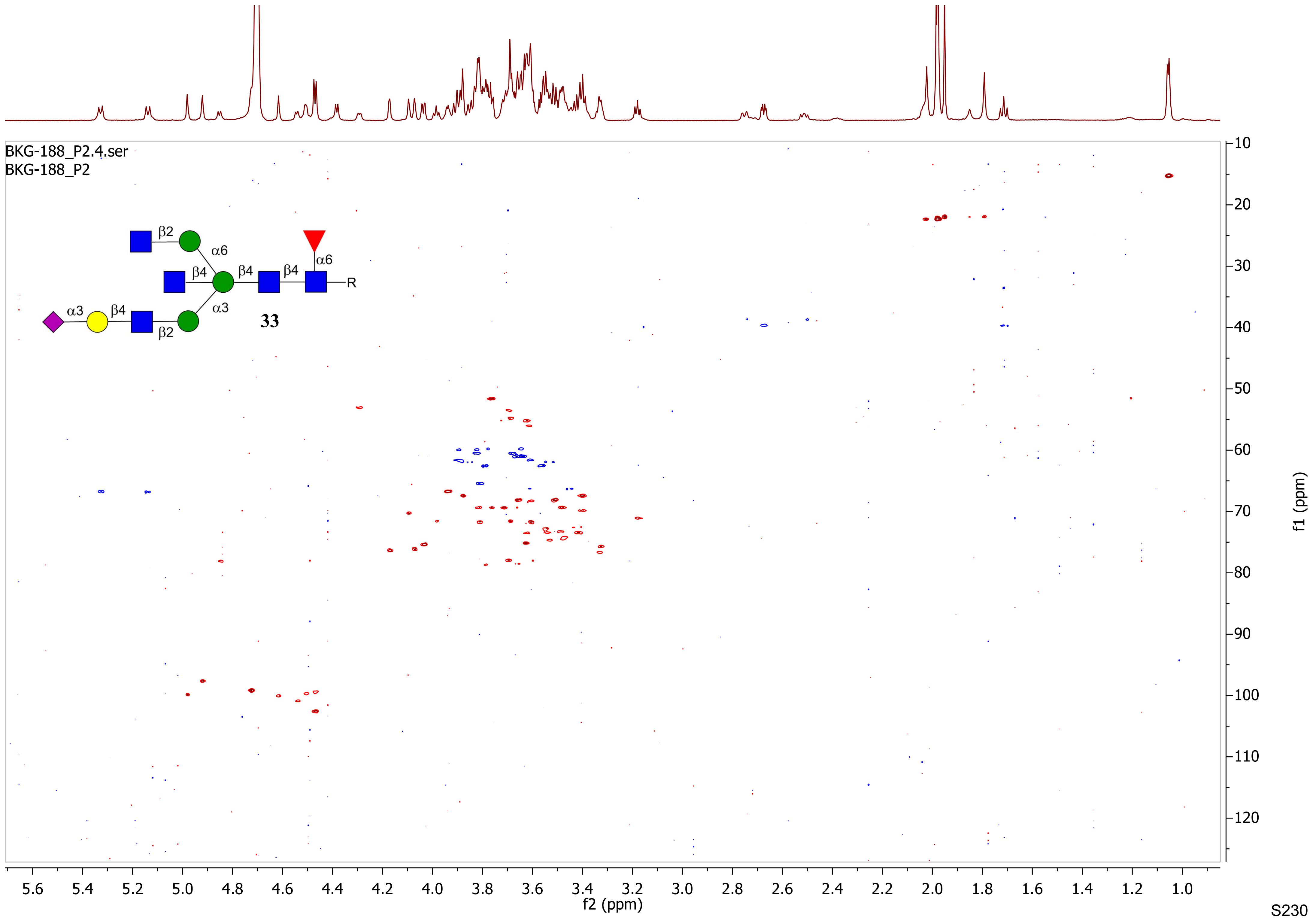
BKG-188_P2.4.ser
BKG-188_P2
β2
α6
β4
β4
β4
α6
R
α3
β4
α3
β2
33
f1 (ppm)
f2 (ppm)

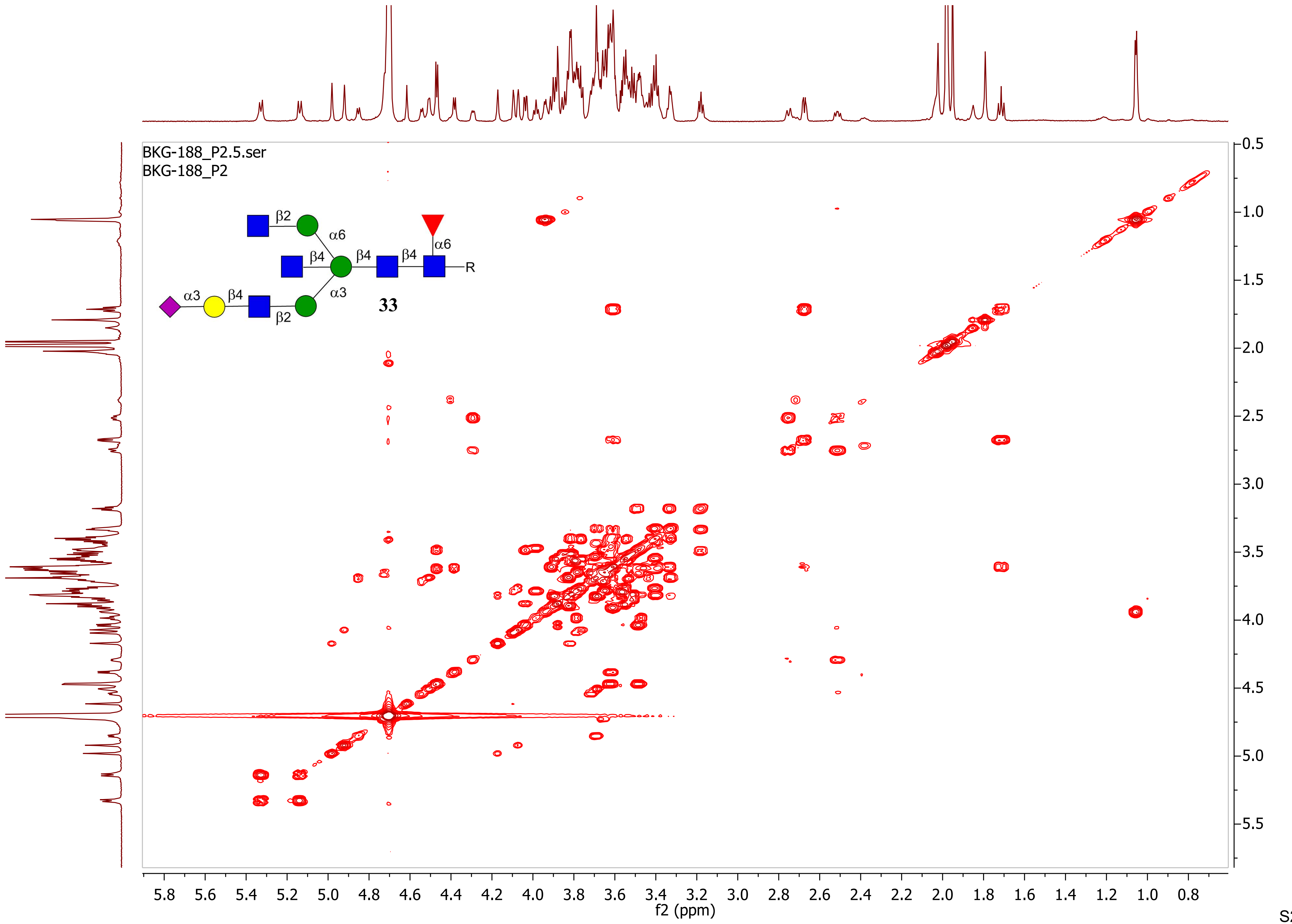
BKG-188_P2.5.ser
BKG-188_P2
β2
α6
β4
β4
β4
α6
R
α3
β4
α3
β2
33
f2 (ppm)
f1 (ppm)

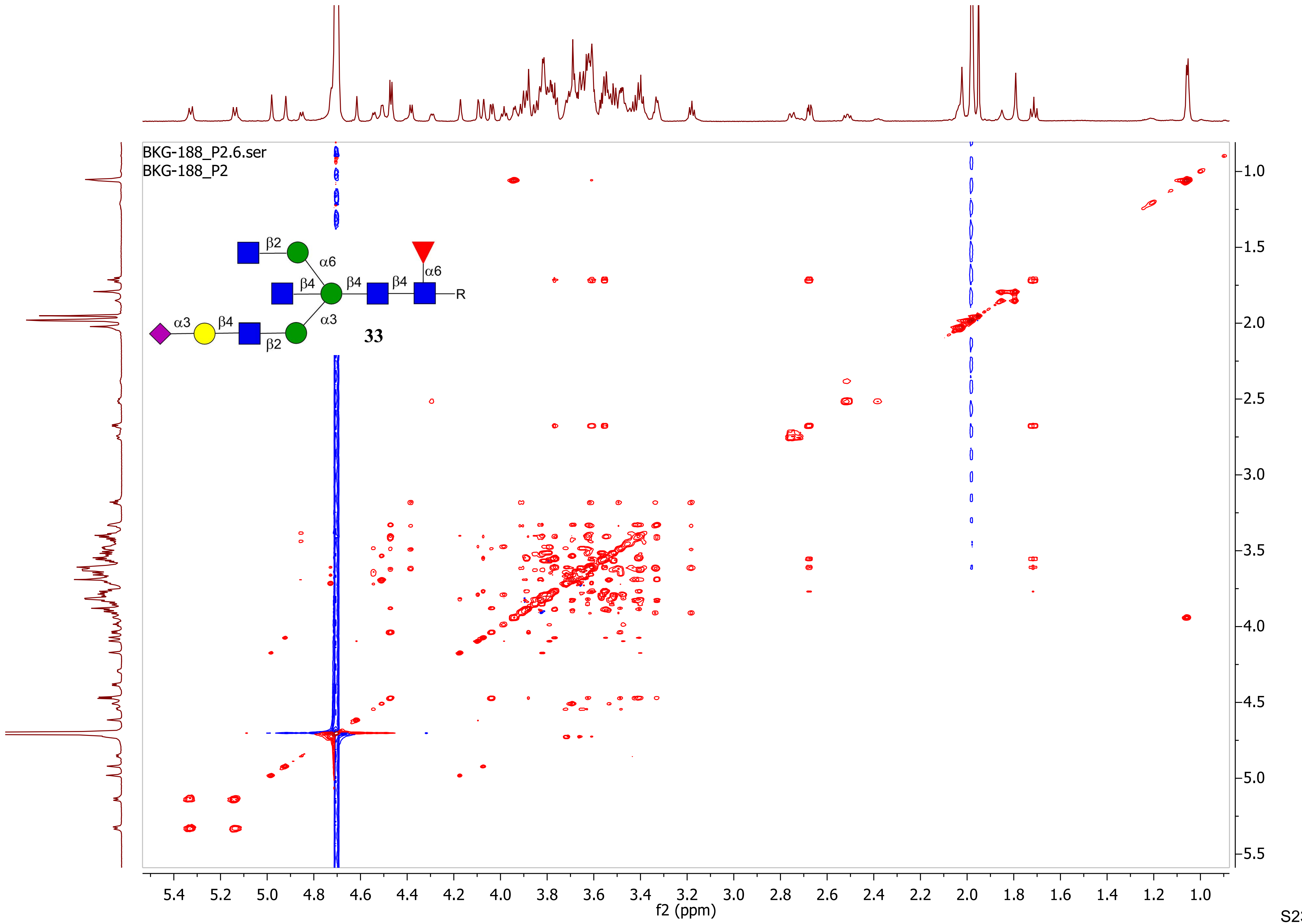

BKG-188_P2.6.ser
BKG-188_P2
β2
α6
β4
β4
β4
α6
R
α3
β4
α3
β2
33
f2 (ppm)
f1 (ppm)

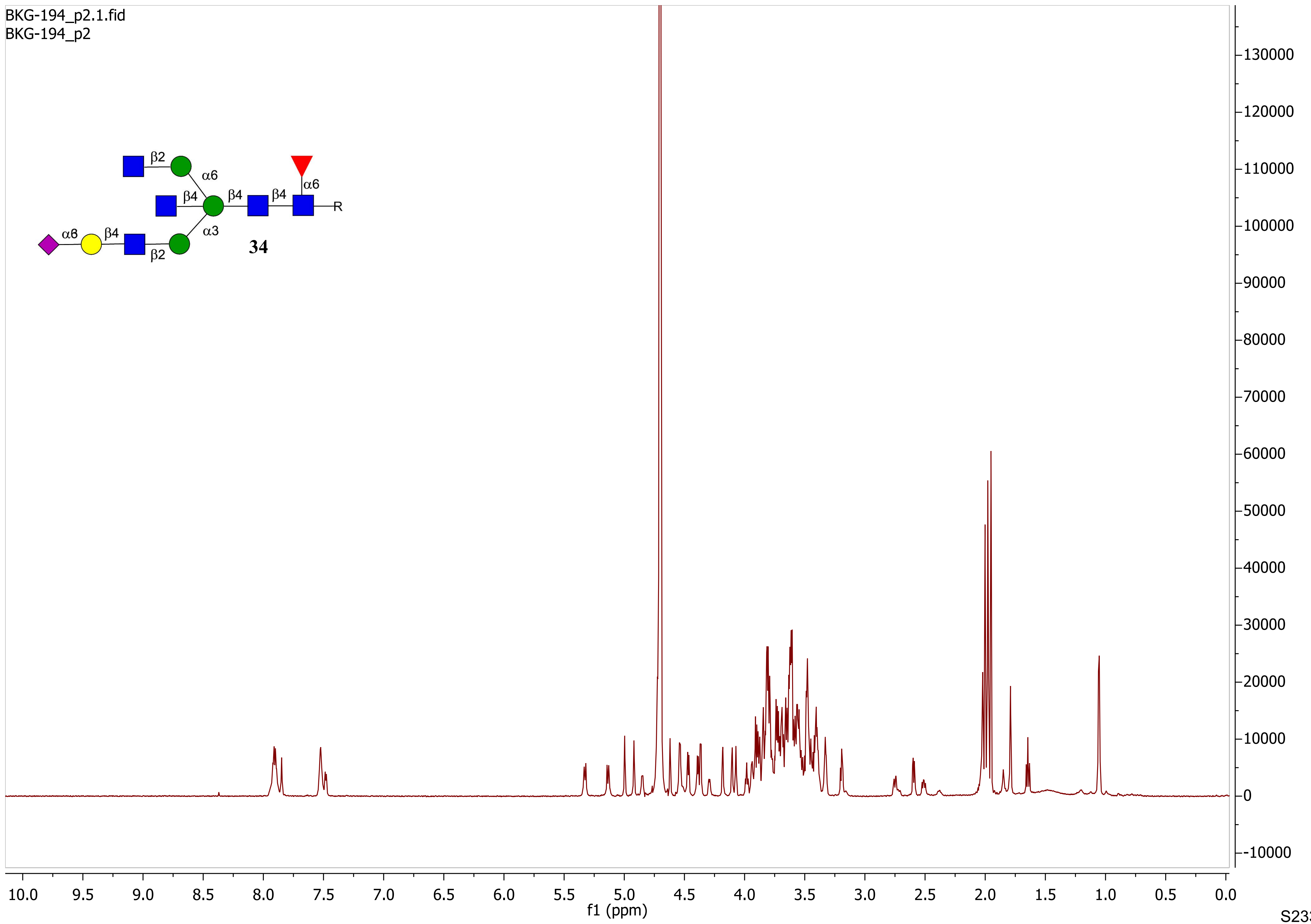

BKG-194_p2.1.fid
BKG-194_p2
β2
α6
β4
β4
β4
α6
R
α8
β4
α3
β2
34
130000
120000
110000
100000
90000
80000
70000
60000
50000
40000
30000
20000
10000
0
-10000
10.0
9.5
9.0
8.5
8.0
7.5
7.0
6.5
6.0
5.5
5.0
4.5
4.0
3.5
3.0
2.5
2.0
1.5
1.0
0.5
0.0
f1 (ppm)

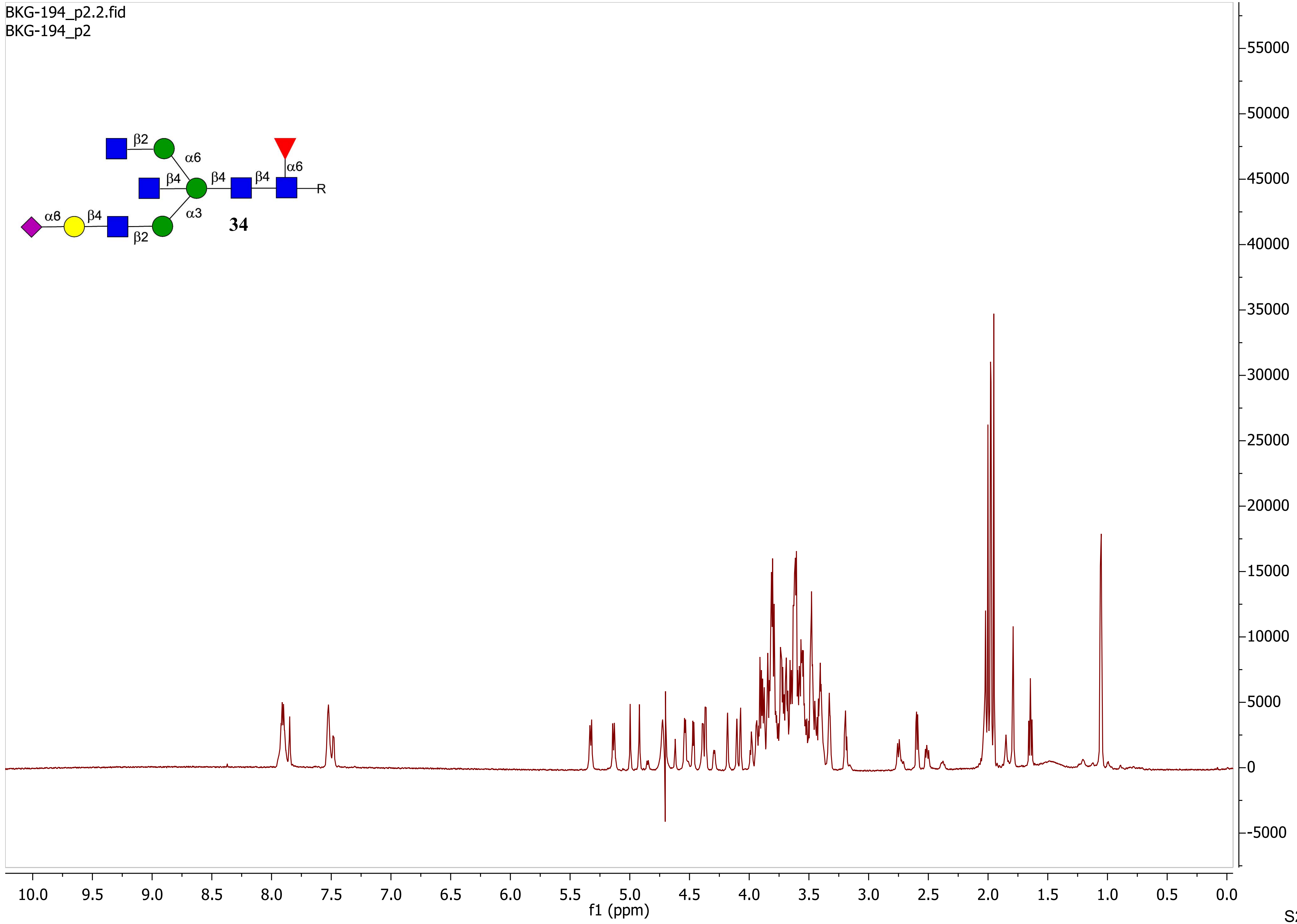
BKG-194_p2.2.fid
BKG-194_p2
β2
α6
β4
β4
β4
α6
R
α3
α6
β4
β2
34
f1 (ppm)

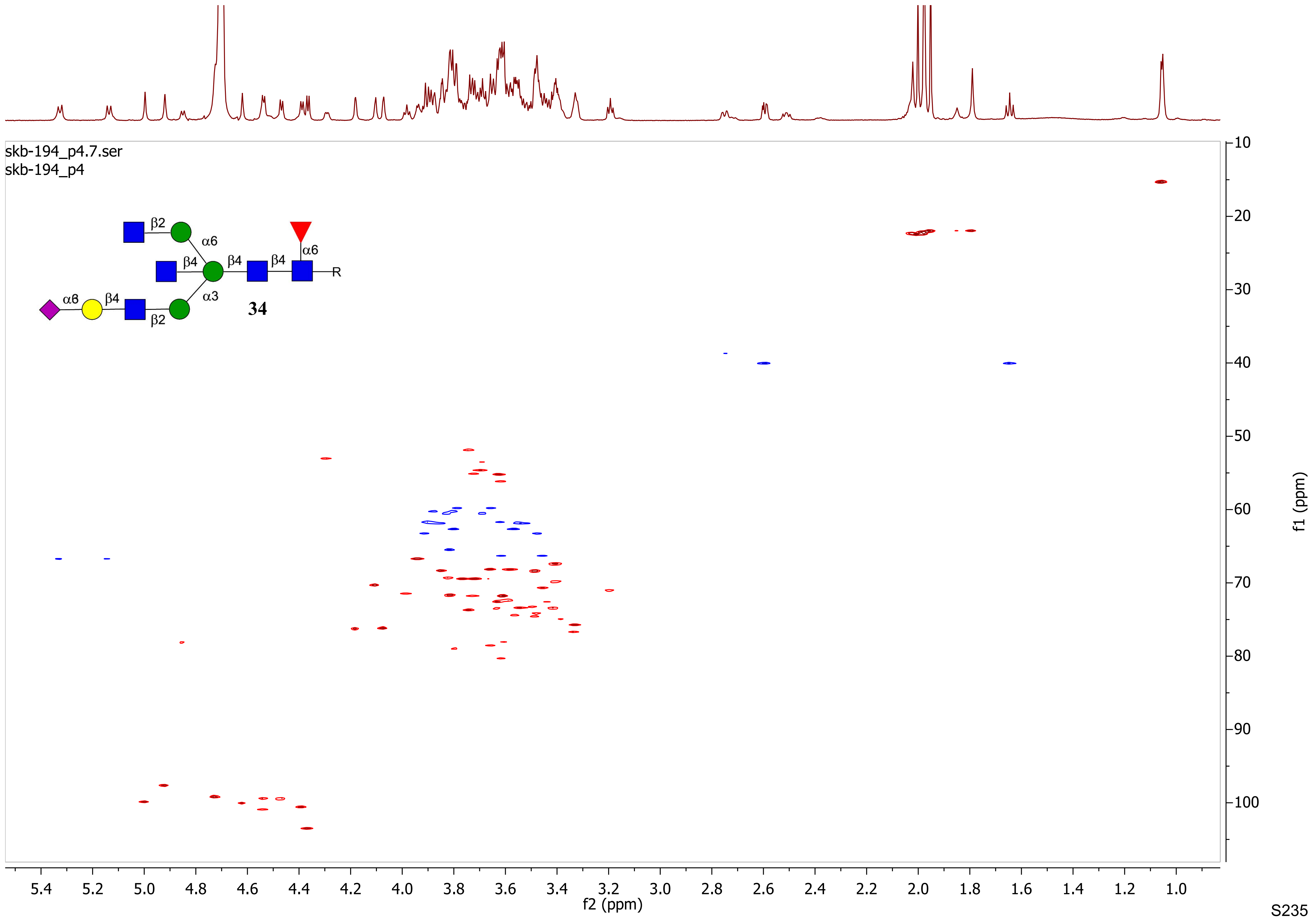
skb-194_p4.7.ser
skb-194_p4
β2
α6
β4
β4
β4
α6
R
α8
β4
α3
β2
34
f2 (ppm)
f1 (ppm)

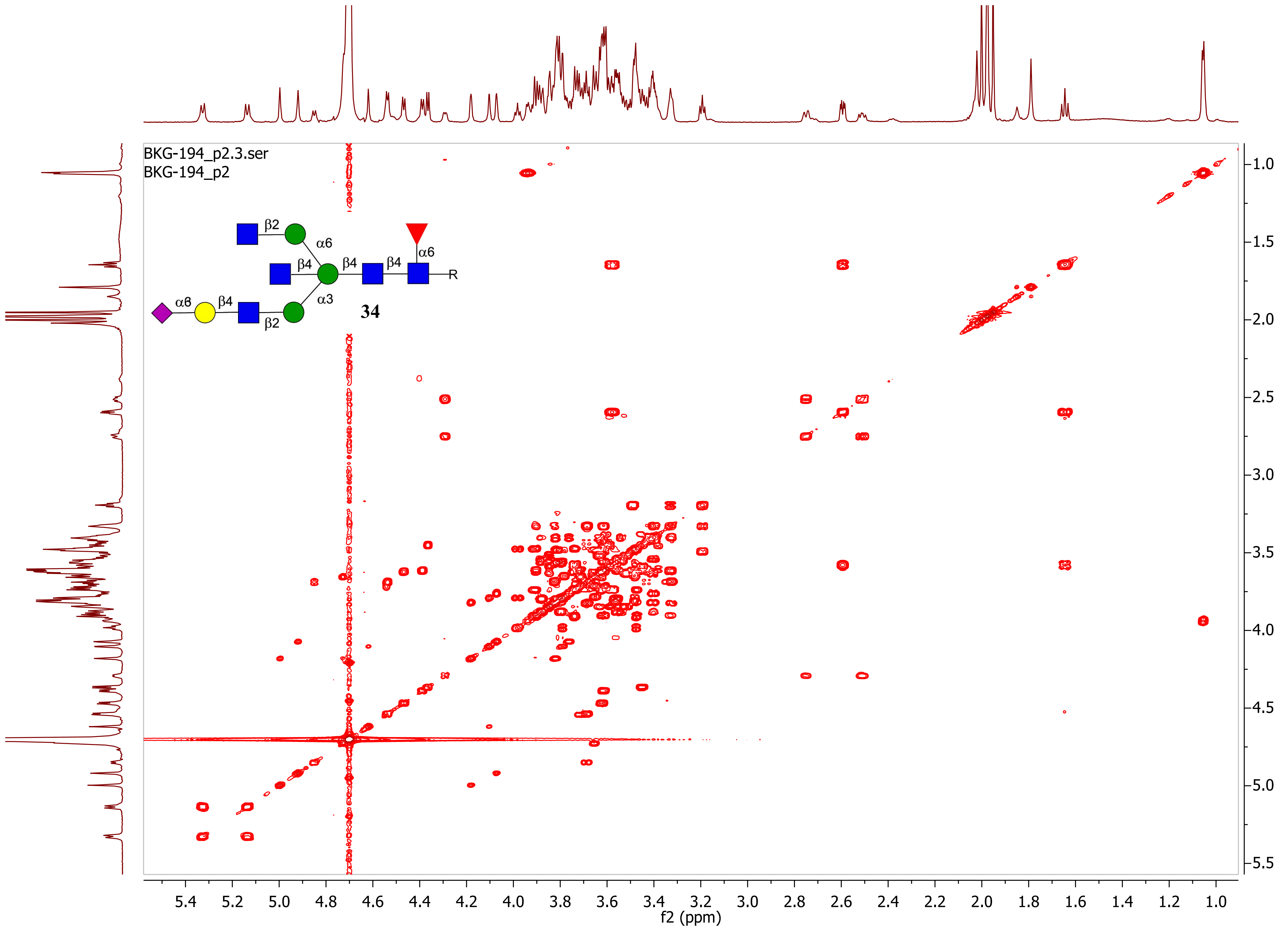

BKG-194_p2.3.ser
BKG-194_p2
β2
α6
β4
β4
β4
α6
R
α6
β4
α3
β2
34
f2 (ppm)
f1 (ppm)

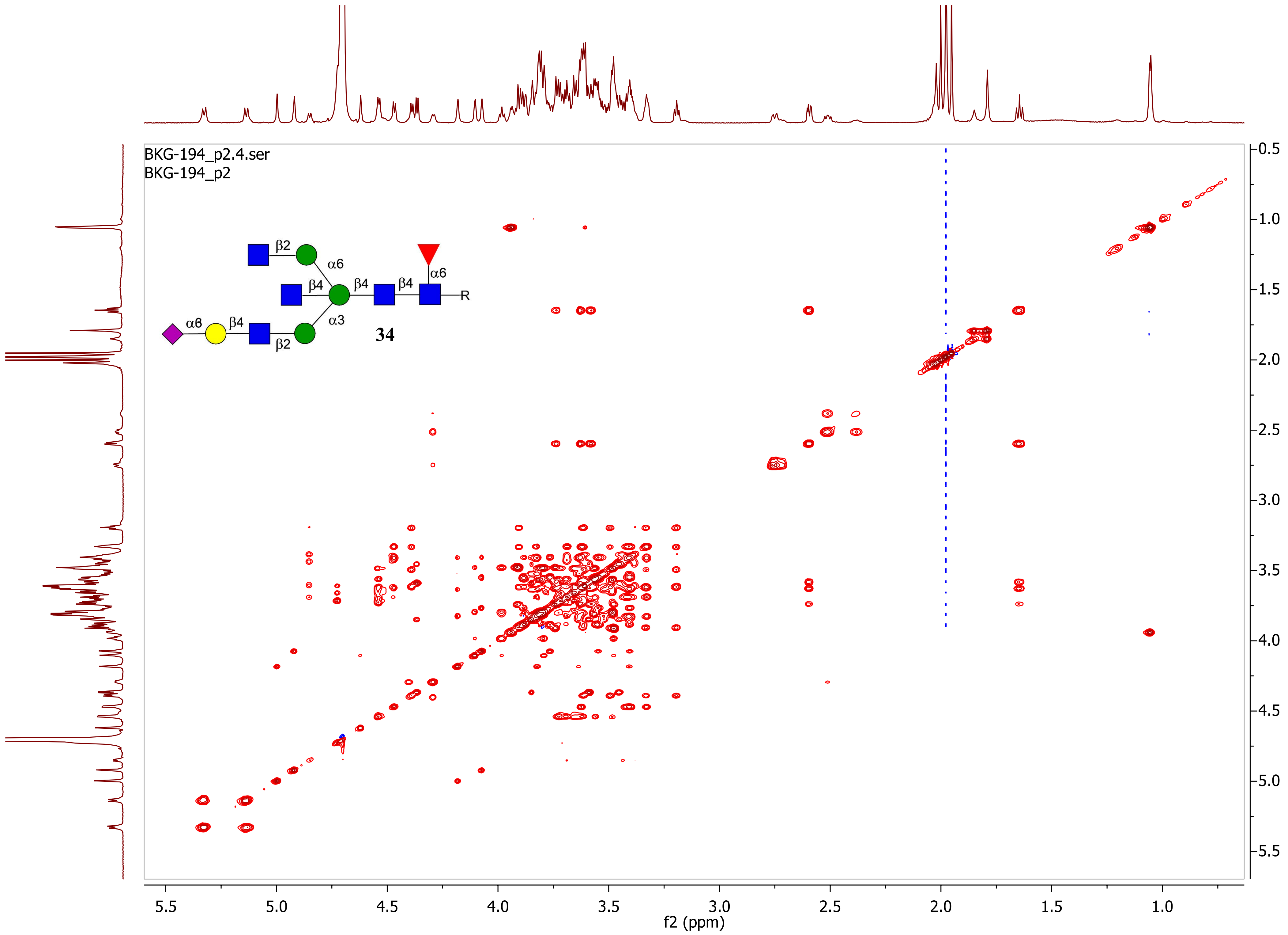
BKG-194_p2.4.ser
BKG-194_p2
β2
α6
β4
β4
β4
α6
R
α6
β4
β2
α3
34
f2 (ppm)
f1 (ppm)

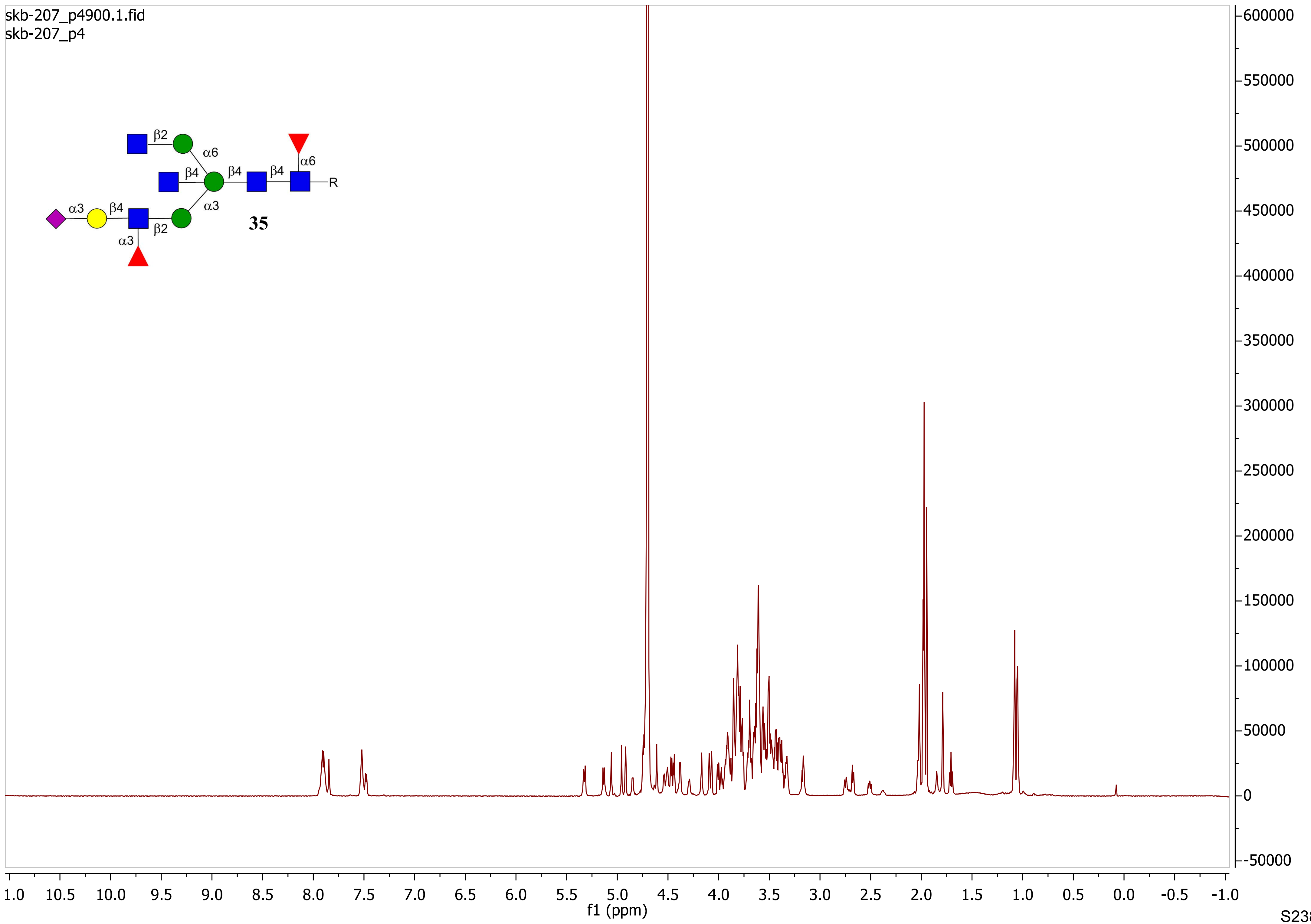

skb-207_p4900.1.fid
skb-207_p4
β2
α6
β4
β4
β4
α6
R
α3
β4
α3
β2
α3
35
f1 (ppm)

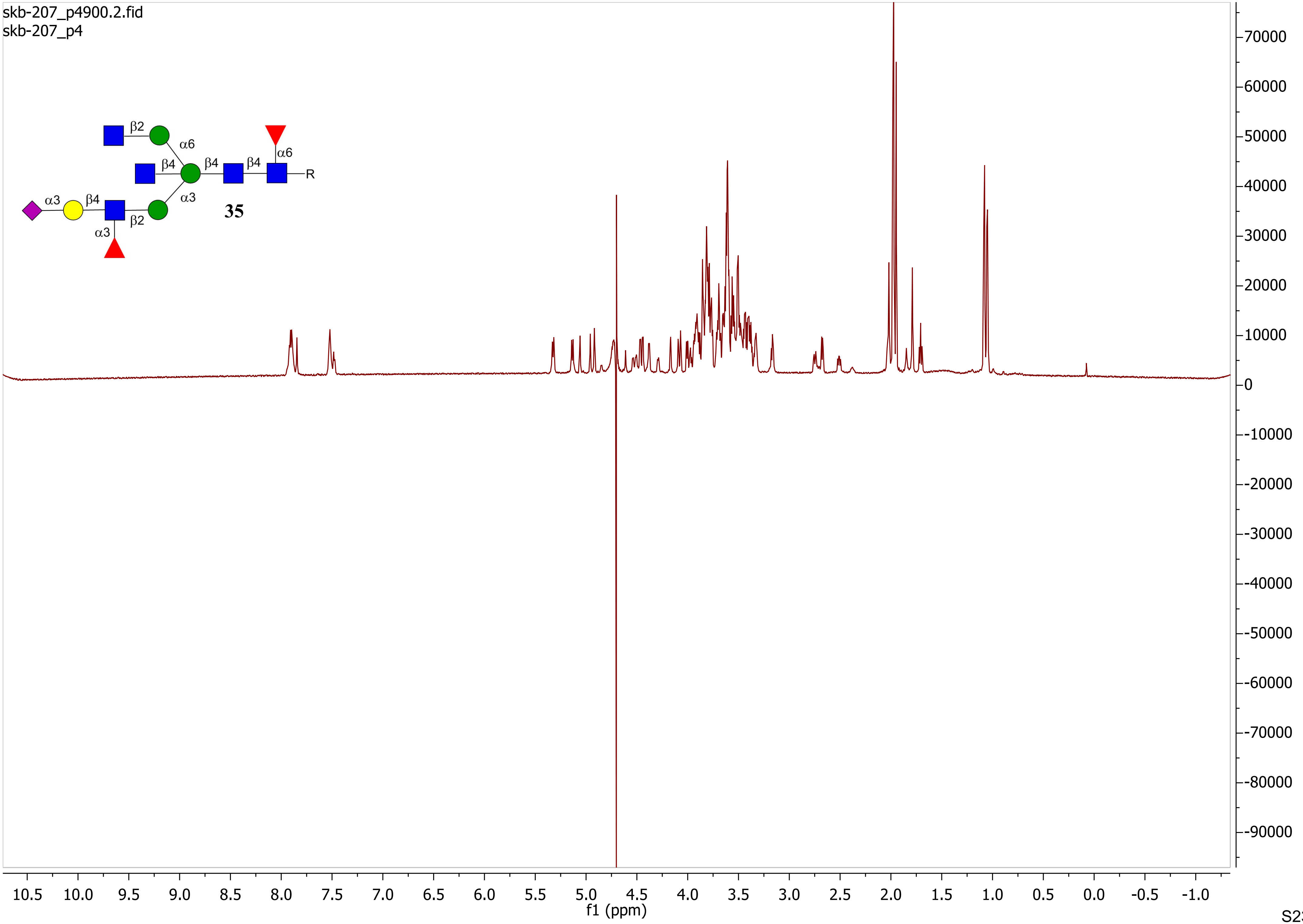
skb-207_p4900.2.fid
skb-207_p4
β2
α6
β4
β4
β4
α6
R
α3
β4
α3
β2
α3
35
70000
60000
50000
40000
30000
20000
10000
0
-10000
-20000
-30000
-40000
-50000
-60000
-70000
-80000
-90000
10.5 10.0 9.5 9.0 8.5 8.0 7.5 7.0 6.5 6.0 5.5 5.0 4.5 4.0 3.5 3.0 2.5 2.0 1.5 1.0 0.5 0.0 -0.5 -1.0
f1 (ppm)

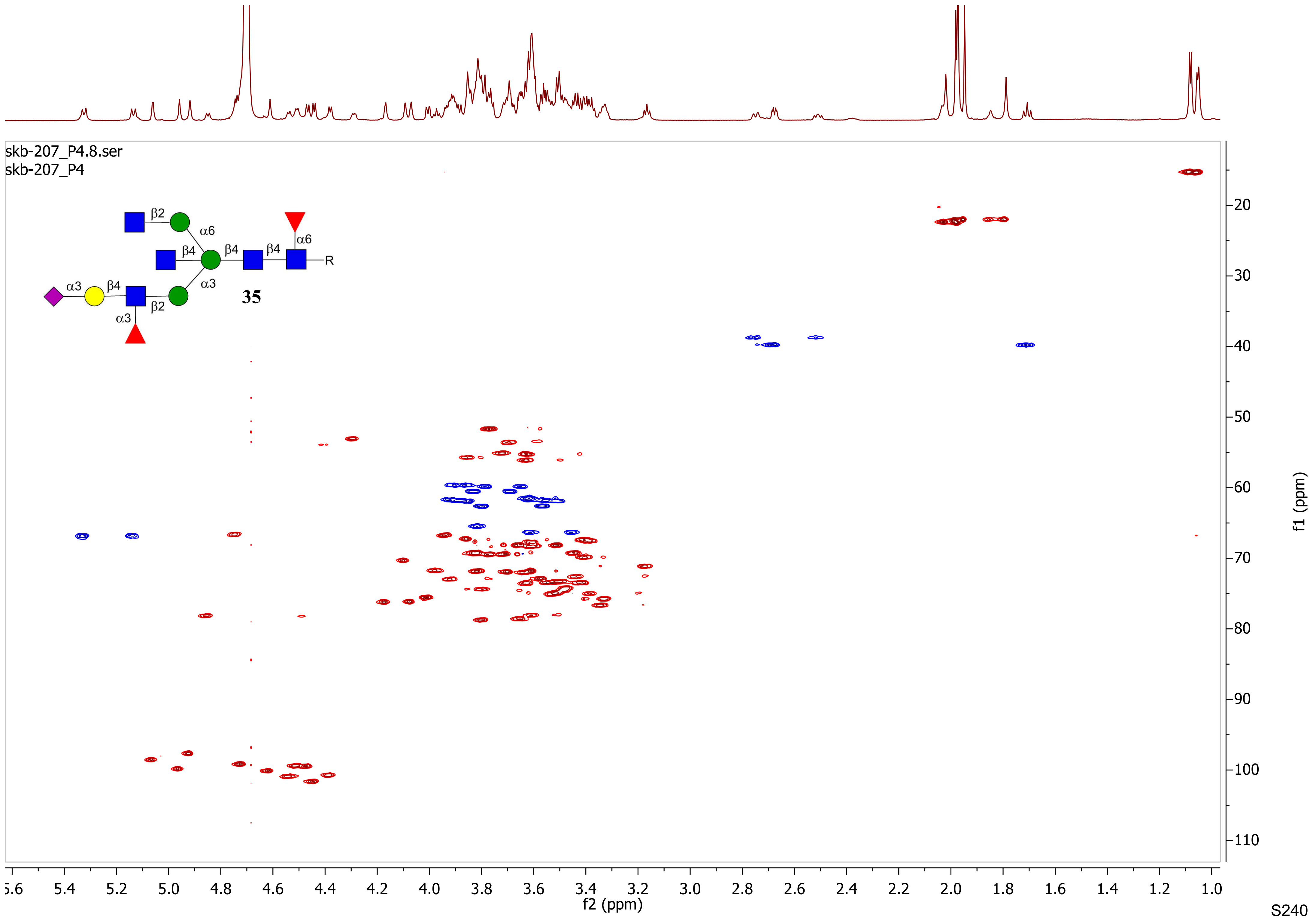
skb-207_P4.8.ser
skb-207_P4
β2
α6
β4
β4
β4
α6
R
α3
β4
α3
β2
α3
35
f2 (ppm)
f1 (ppm)

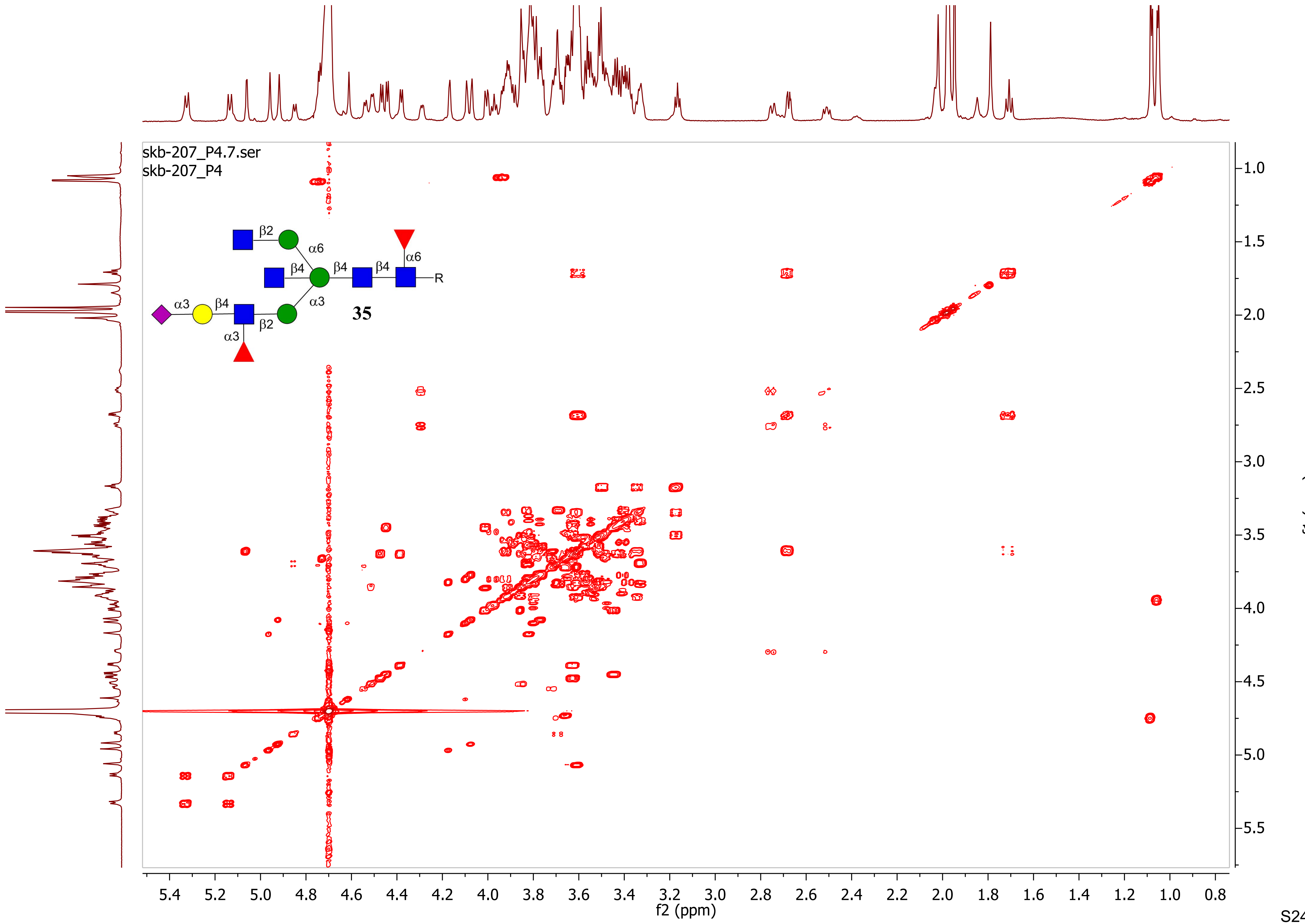
skb-207_P4.7.ser
skb-207_P4
β2
α6
β4
β4
β4
α6
R
α3
β4
α3
β2
α3
35
f2 (ppm)
f1 (ppm)

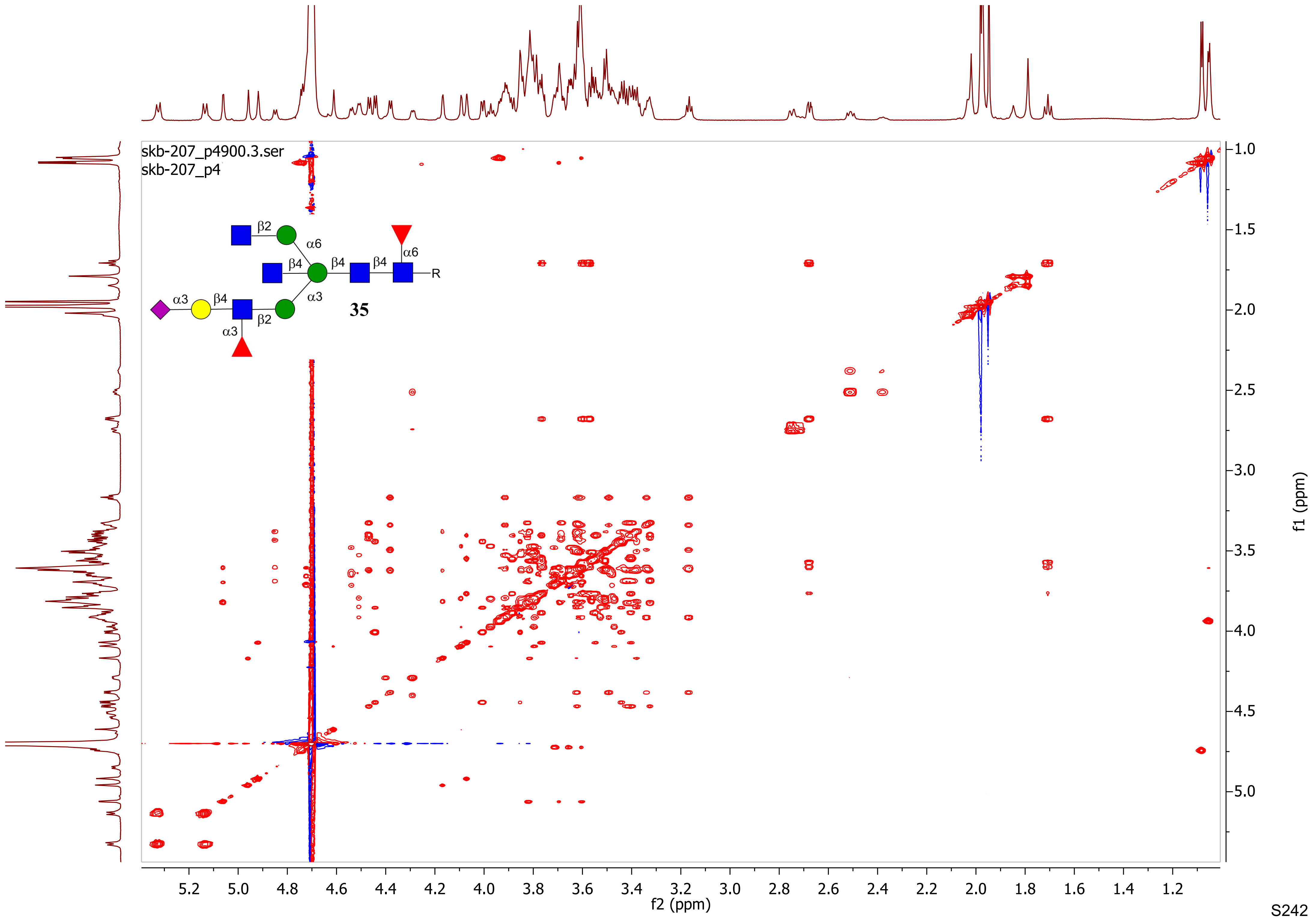

skb-207_p4900.3.ser
skb-207_p4
β2
α6
β4
β4
β4
α6
R
α3
β4
α3
β2
α3
35
f2 (ppm)
f1 (ppm)

skb-213_P4900.1.fid
skb-213_P4

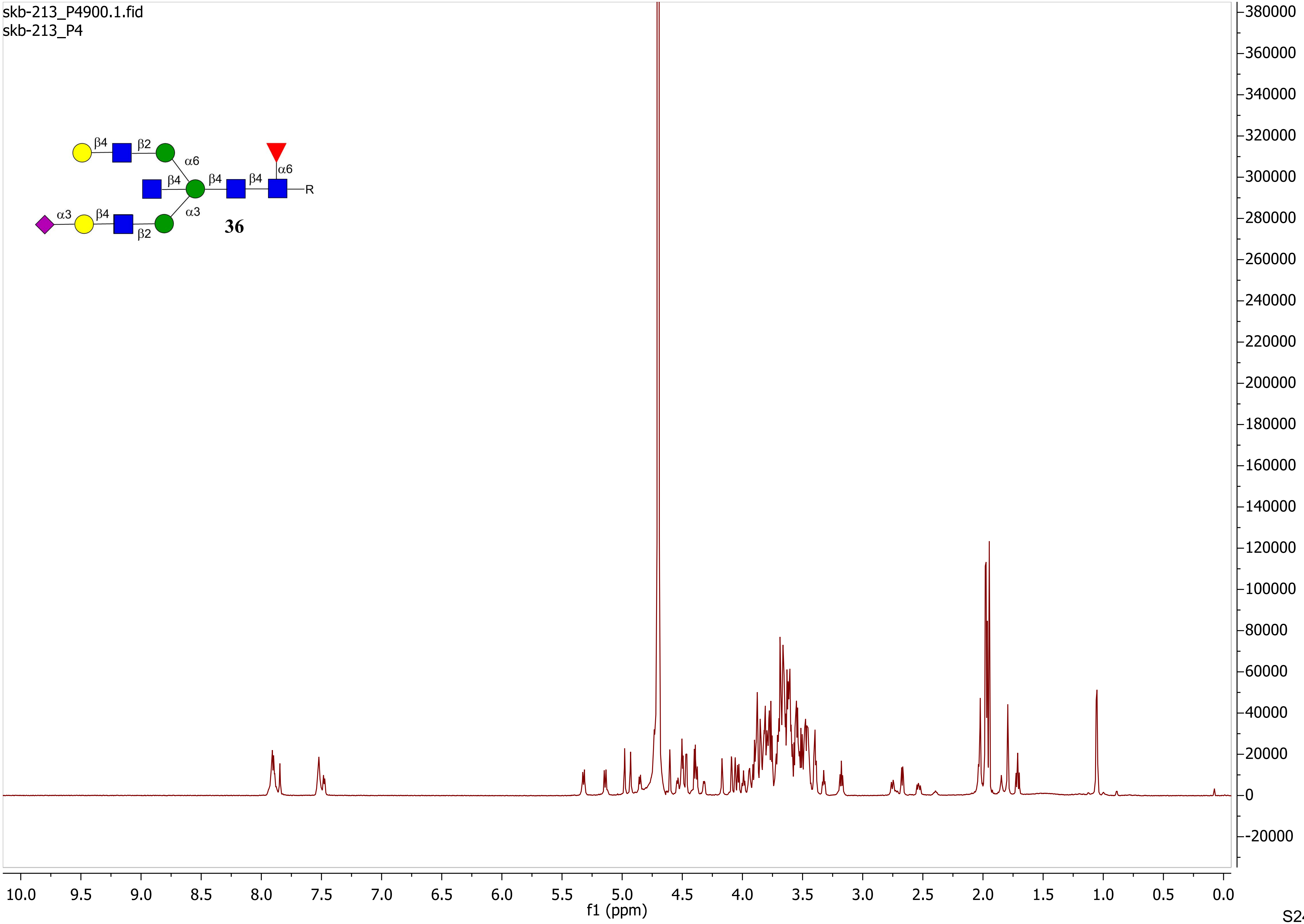

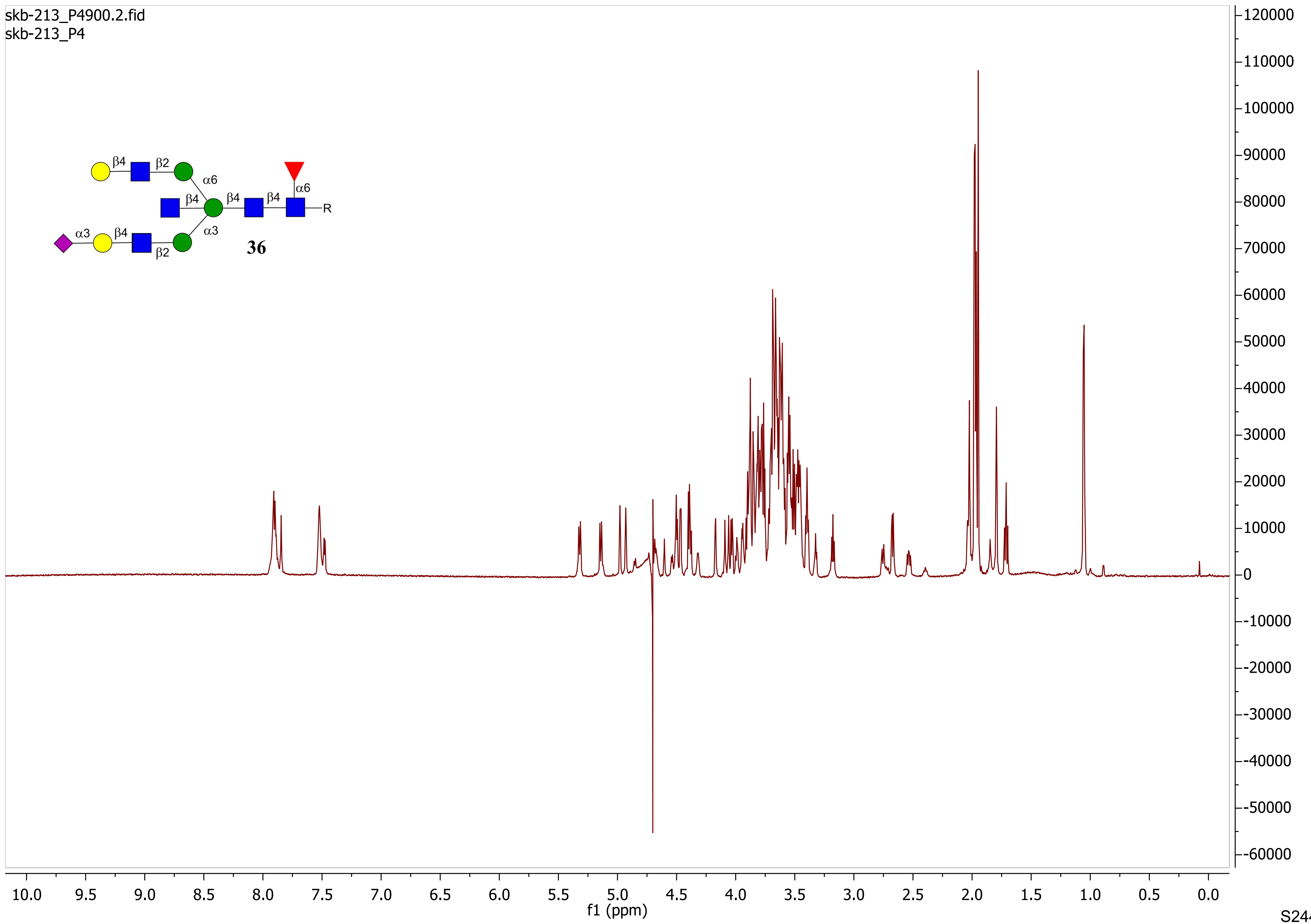
skb-213_P4900.2.fid
skb-213_P4
β4
β2
α6
β4
β4
β4
α6
R
α3
β4
α3
β2
36
f1 (ppm)

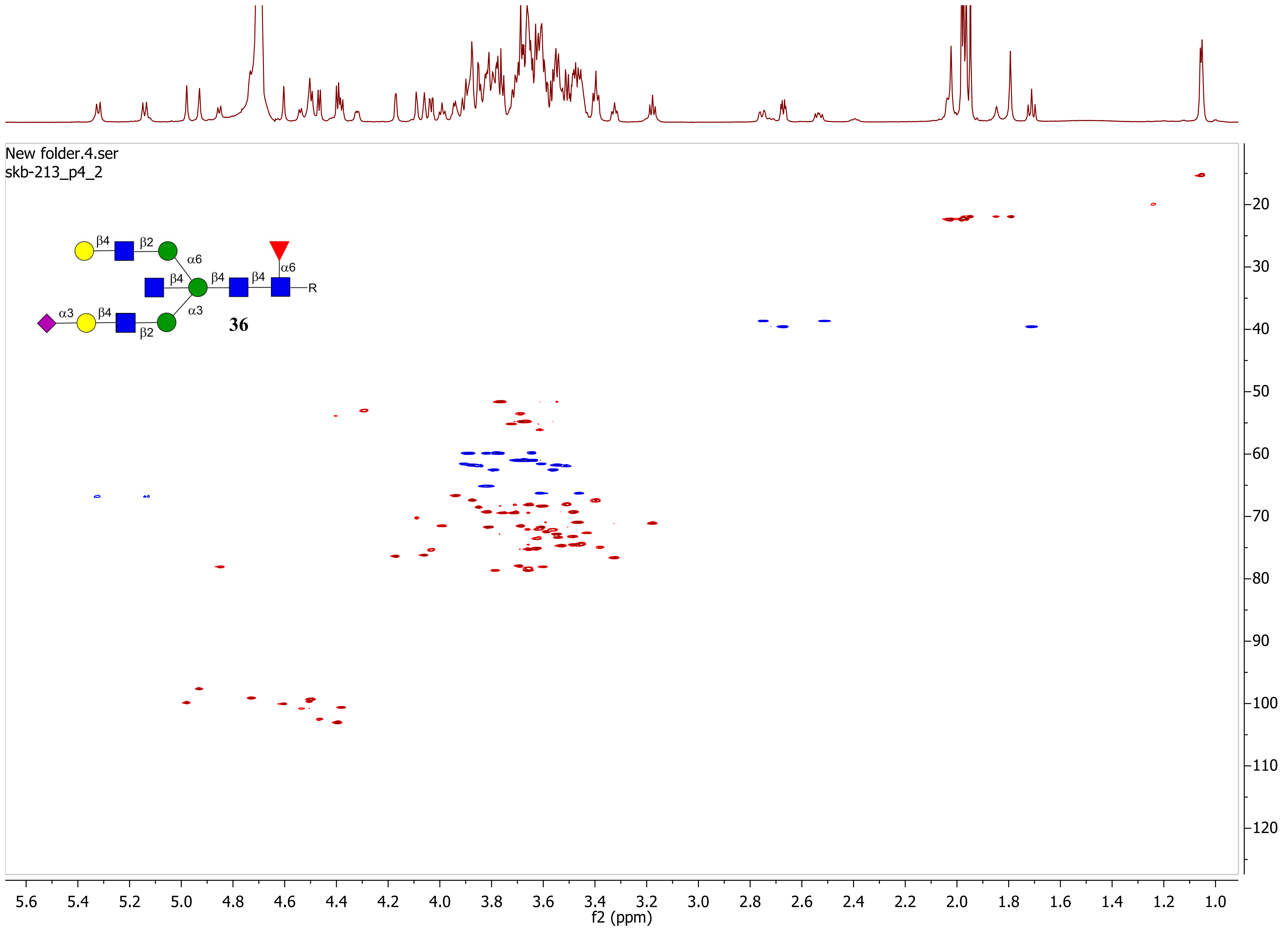
New folder.4.ser
skb-213_p4_2
β4
β2
α6
α6
β4
β4
β4
R
α3
β4
α3
β2
36
f2 (ppm)
f1 (ppm)

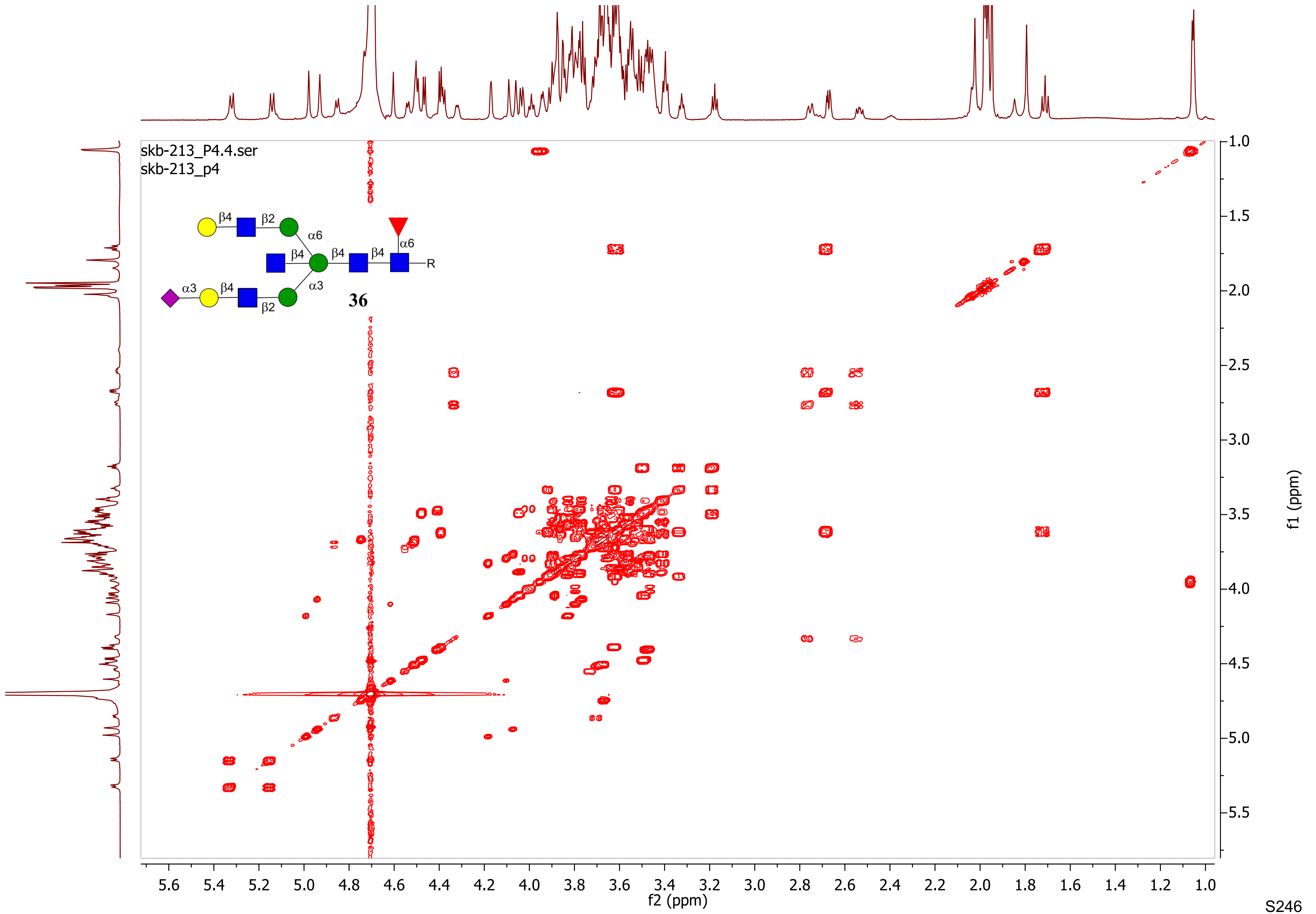
skb-213_P4.4.ser
skb-213_p4
β4
β2
α6
β4
β4
β4
α6
R
α3
β4
α3
β2
36
f1 (ppm)
f2 (ppm)

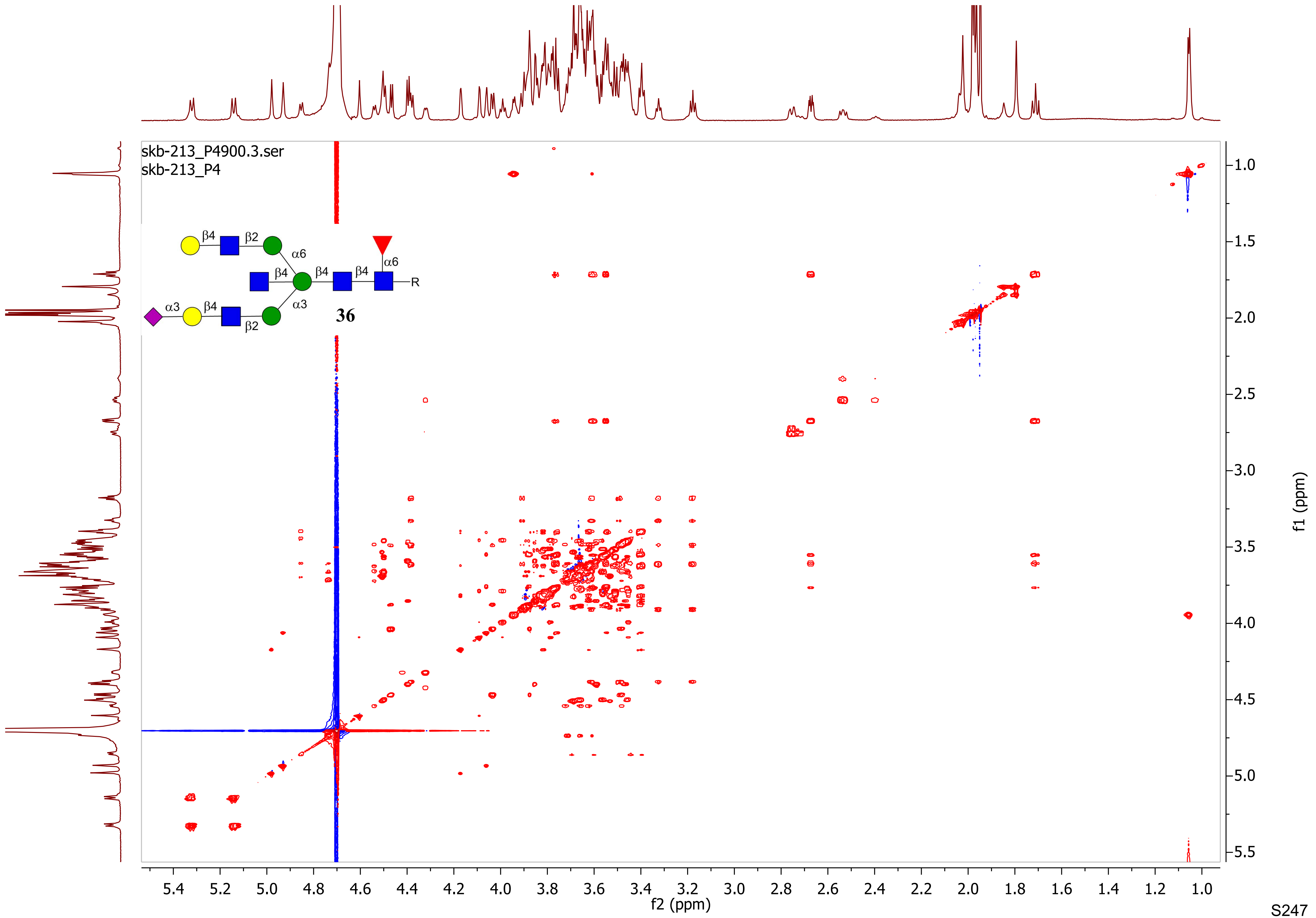
skb-213_P4900.3.ser
skb-213_P4
36
f2 (ppm)
f1 (ppm)

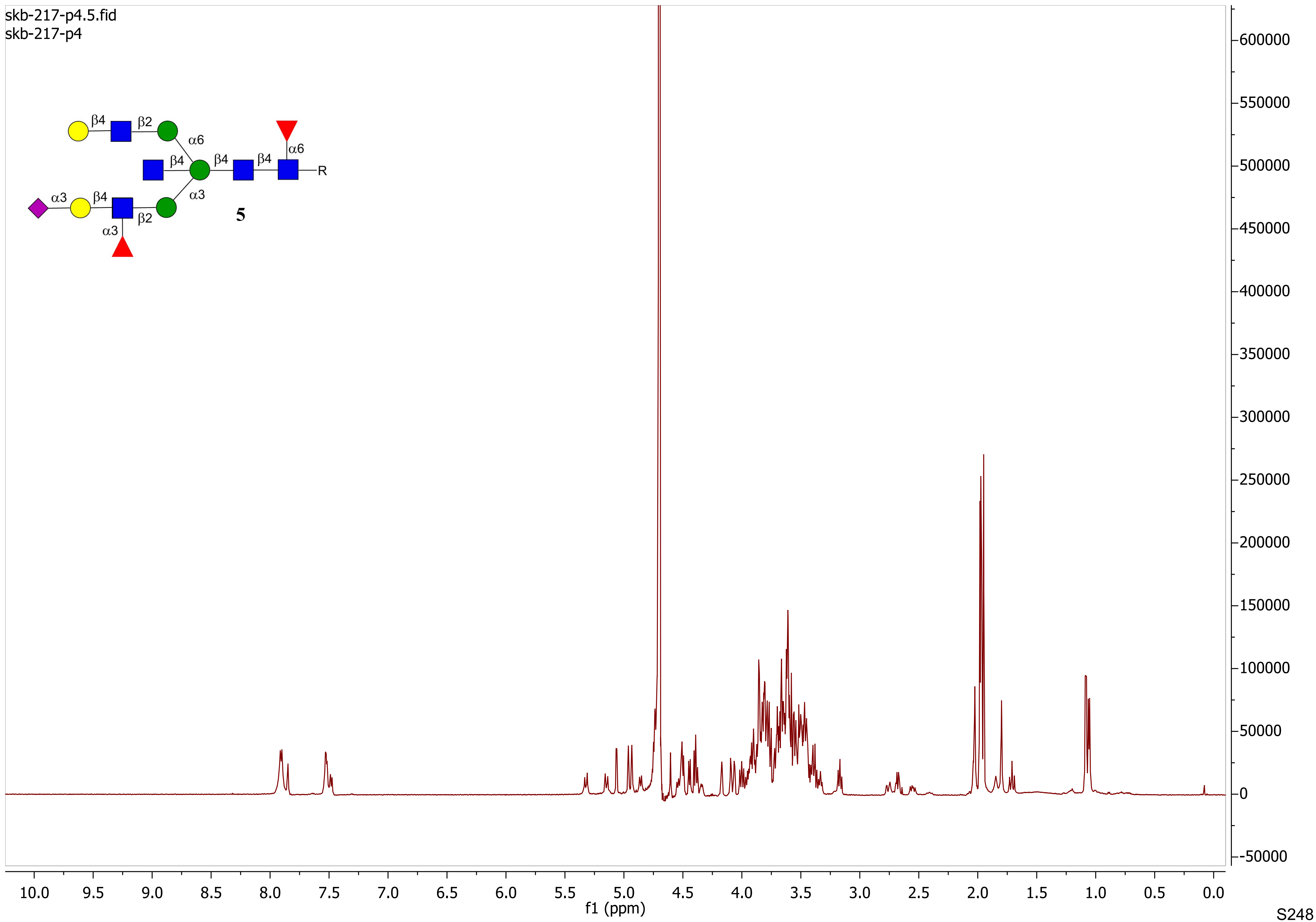

skb-217-p4.5.fid
skb-217-p4
β4
β2
α6
α6
β4
β4
β4
R
α3
β4
α3
β2
α3
5
f1 (ppm)

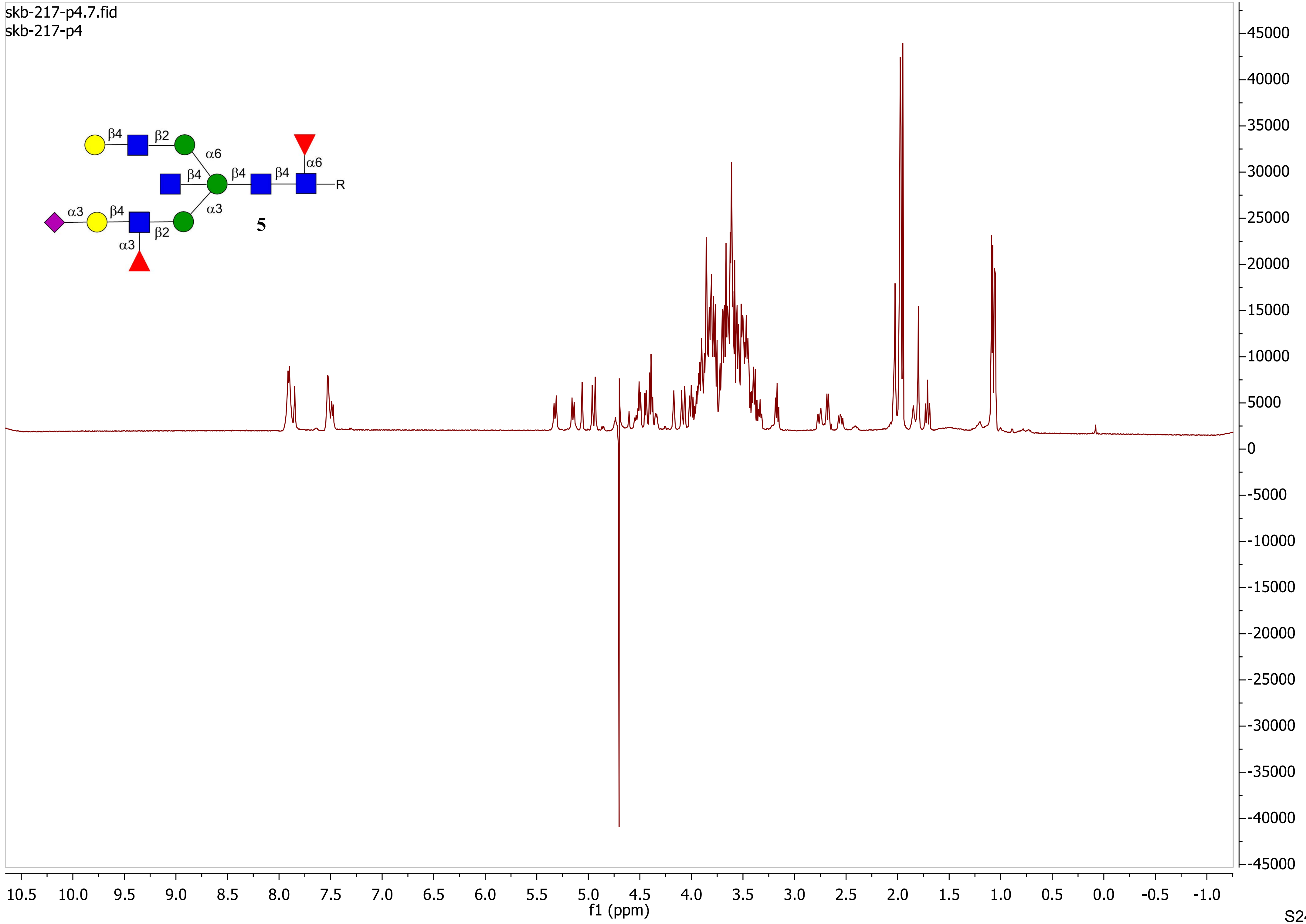
skb-217-p4.7.fid
skb-217-p4
β4
β2
α6
α6
β4
β4
β4
R
α3
β4
α3
β2
α3
5
f1 (ppm)

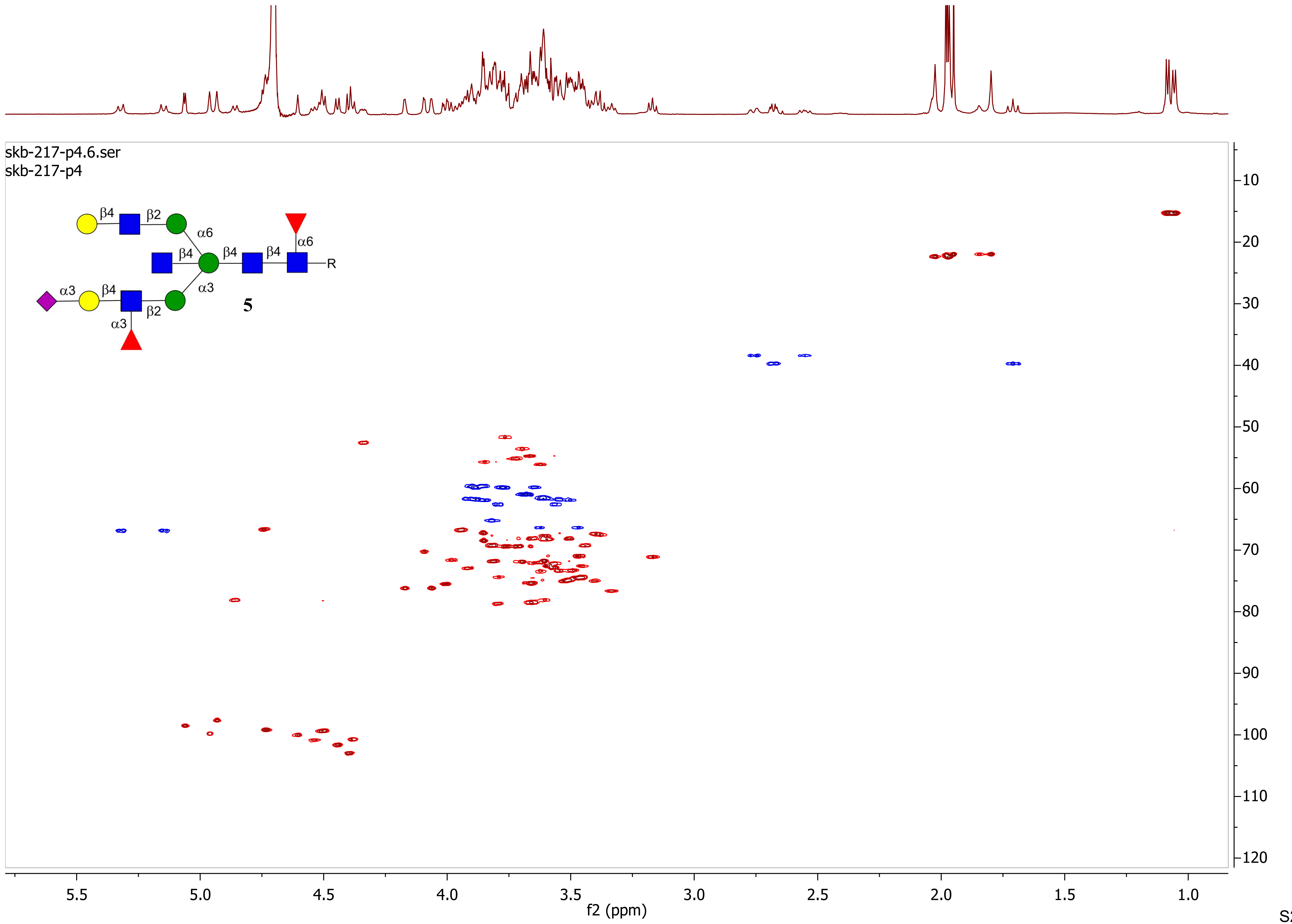

skb-217-p4.6.ser
skb-217-p4
β4
β2
α6
β4
β4
β4
α6
R
α3
β4
α3
β2
α3
5
f2 (ppm)
f1 (ppm)

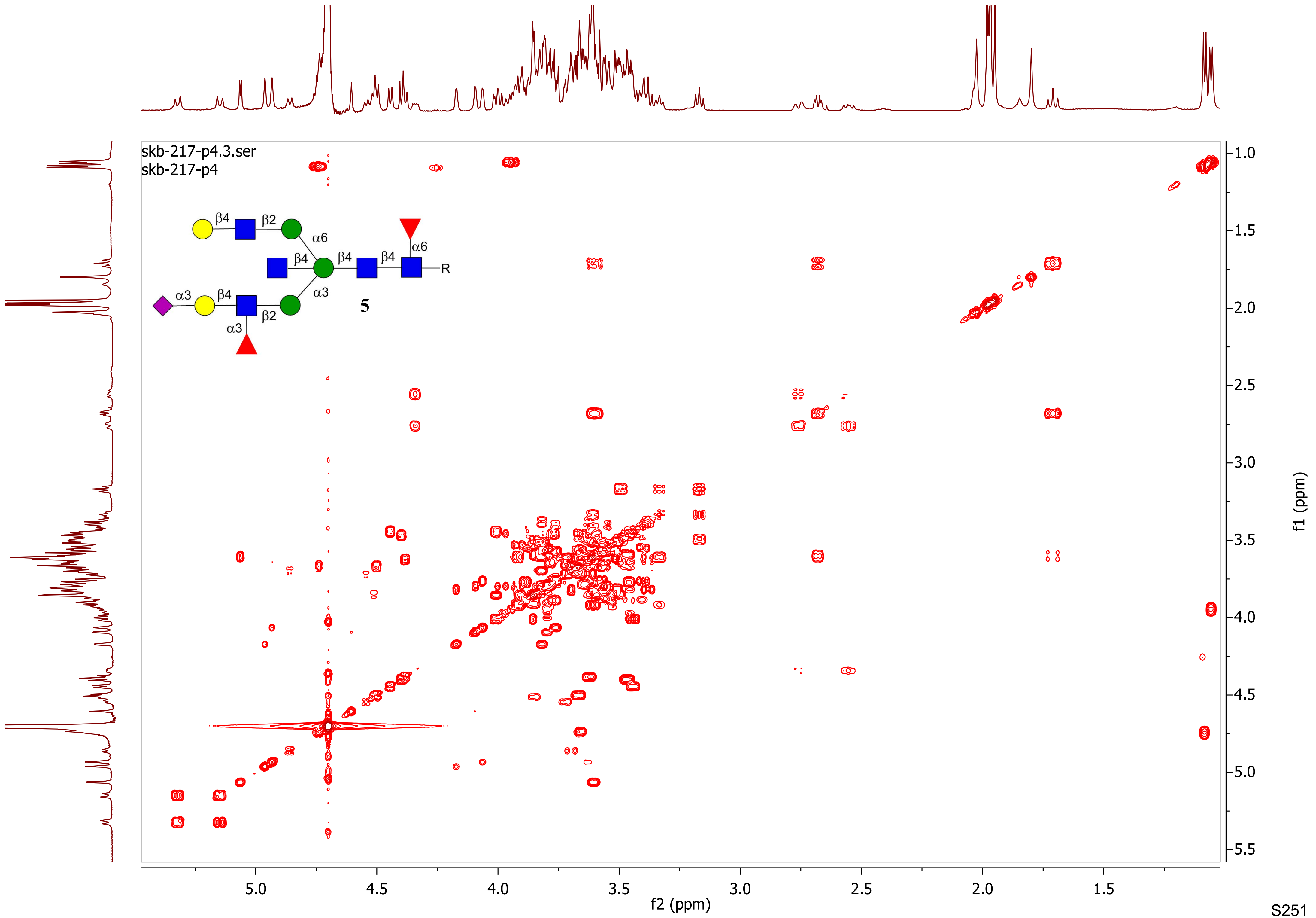

skb-217-p4.3.ser
skb-217-p4
β4
β2
α6
β4
β4
β4
α6
R
α3
β4
α3
β2
α3
5
f2 (ppm)
f1 (ppm)

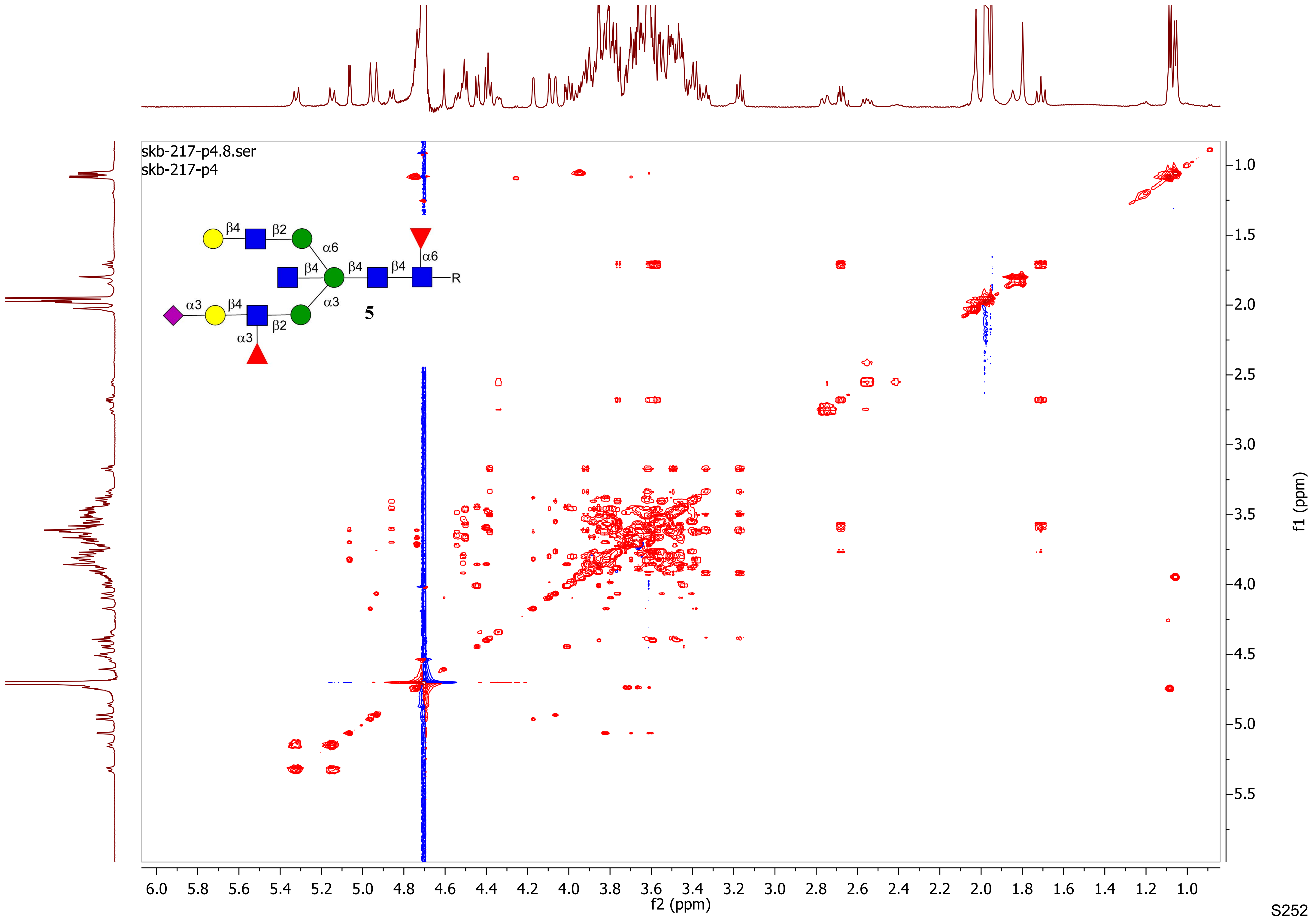
skb-217-p4.8.ser
skb-217-p4
β4
β2
α6
β4
β4
β4
α6
R
α3
β4
α3
β2
α3
5
f1 (ppm)
f2 (ppm)

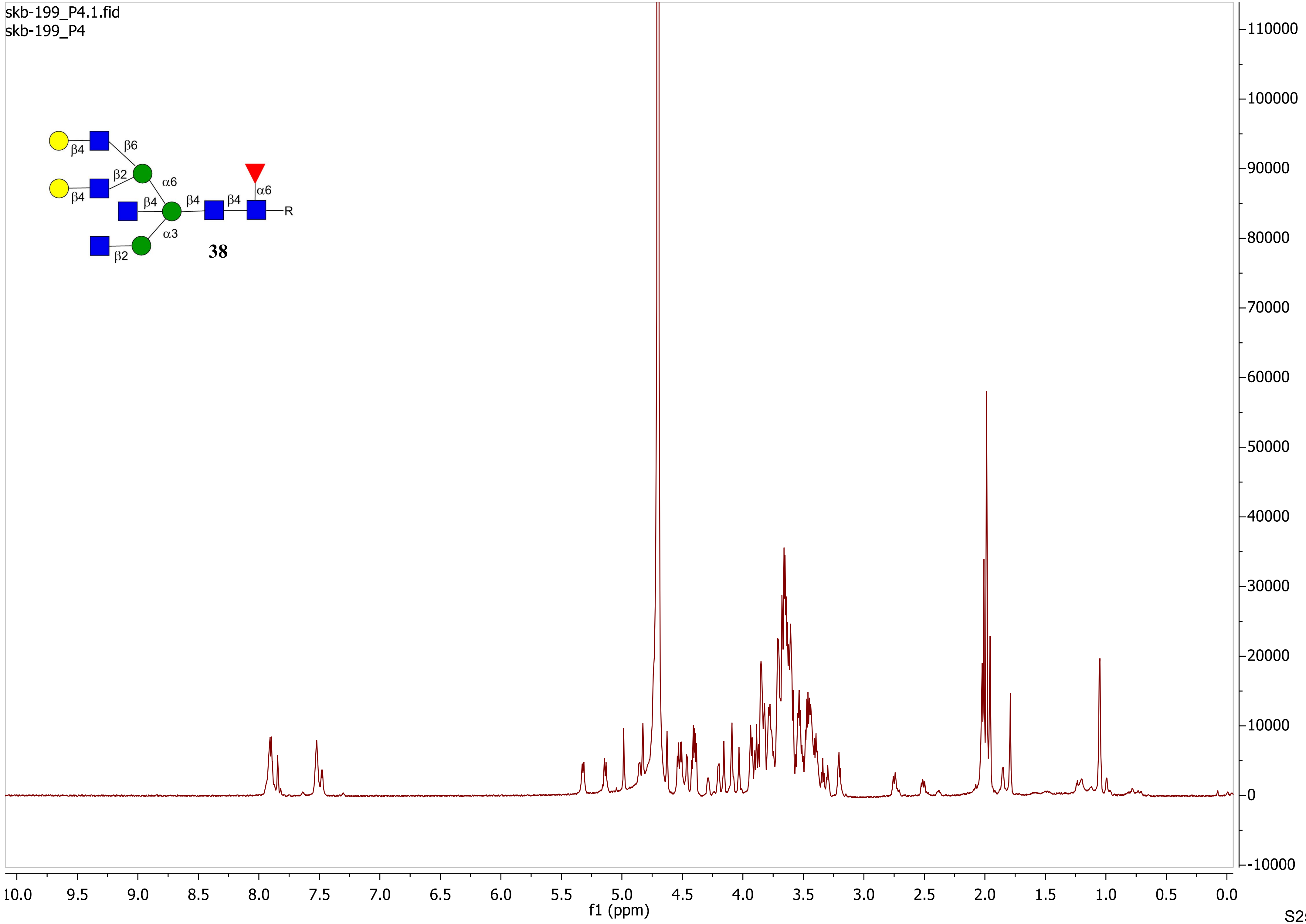
skb-199_P4.1.fid
skb-199_P4
β4
β6
β2
α6
β4
β4
β4
β4
α6
R
α3
β2
38
f1 (ppm)
10.0
9.5
9.0
8.5
8.0
7.5
7.0
6.5
6.0
5.5
5.0
4.5
4.0
3.5
3.0
2.5
2.0
1.5
1.0
0.5
0.0
110000
100000
90000
80000
70000
60000
50000
40000
30000
20000
10000
0
-10000

skb-199_P4.2.fid
skb-199_P4

38

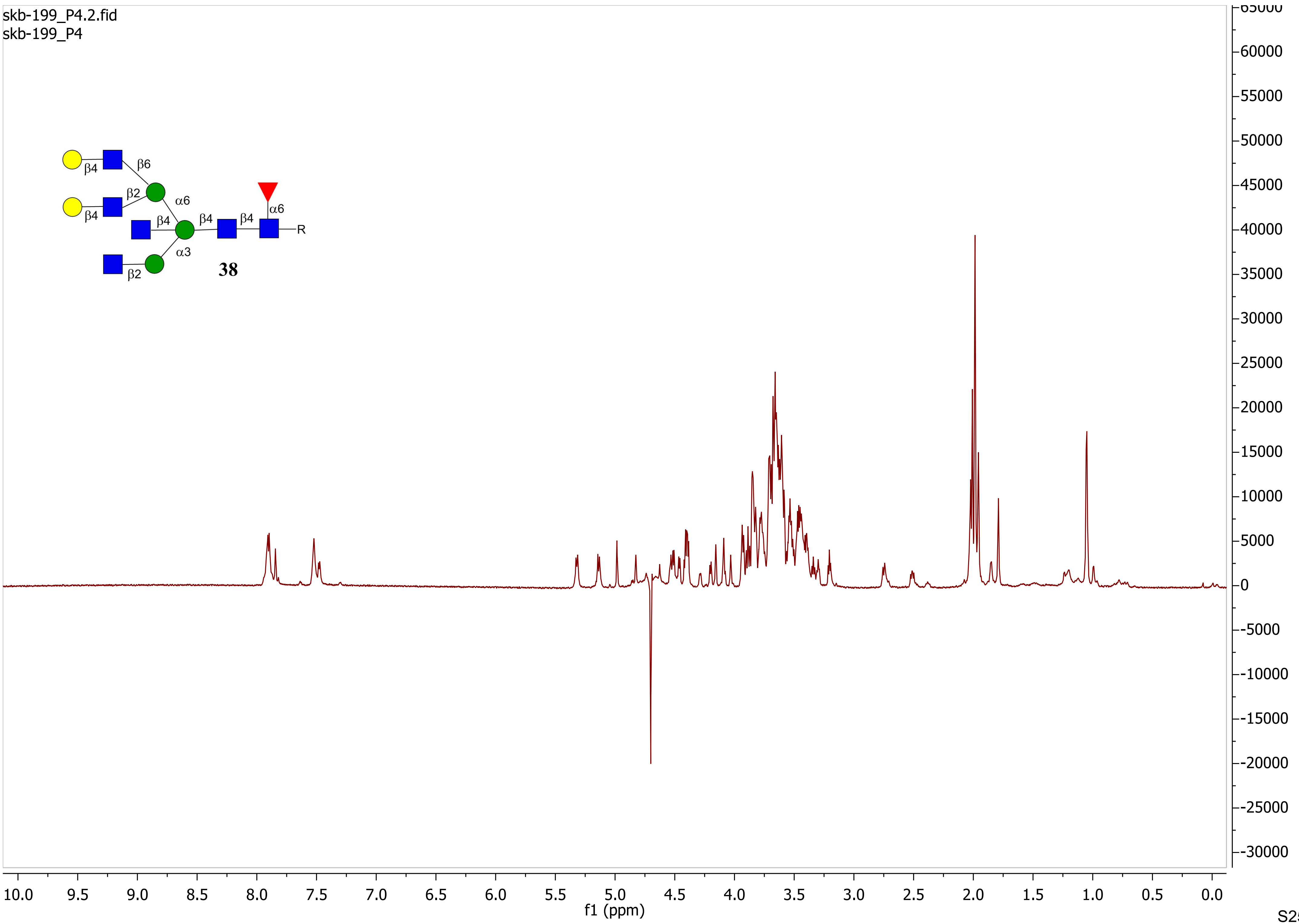

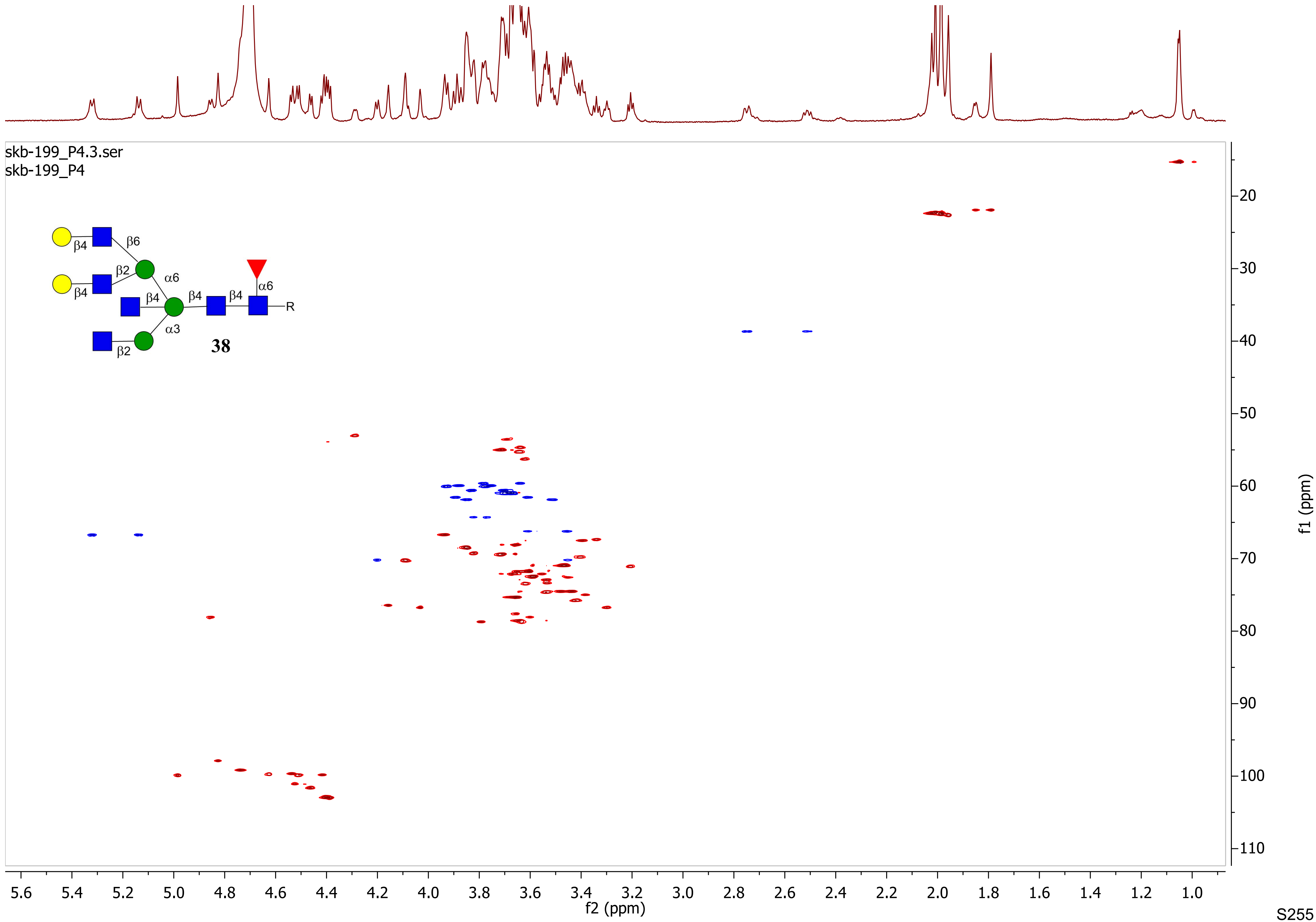
skb-199_P4.3.ser
skb-199_P4
β4
β6
β2
α6
β4
β4
β4
β4
α6
R
α3
β2
38
f2 (ppm)
f1 (ppm)

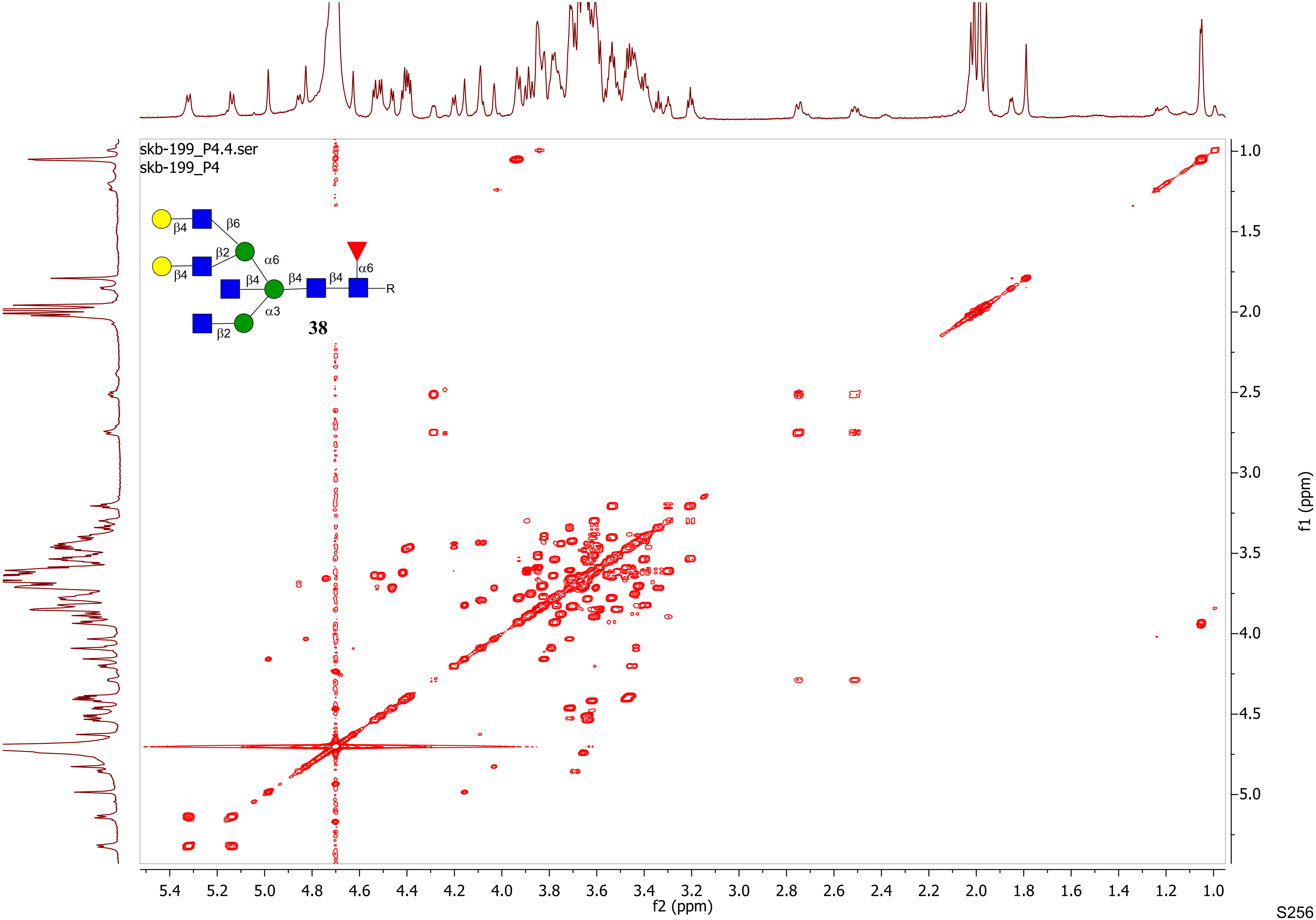
skb-199_P4.4.ser
skb-199_P4
β4
β6
β2
α6
β4
β4
β4
β4
α6
R
α3
β2
38
f2 (ppm)
f1 (ppm)

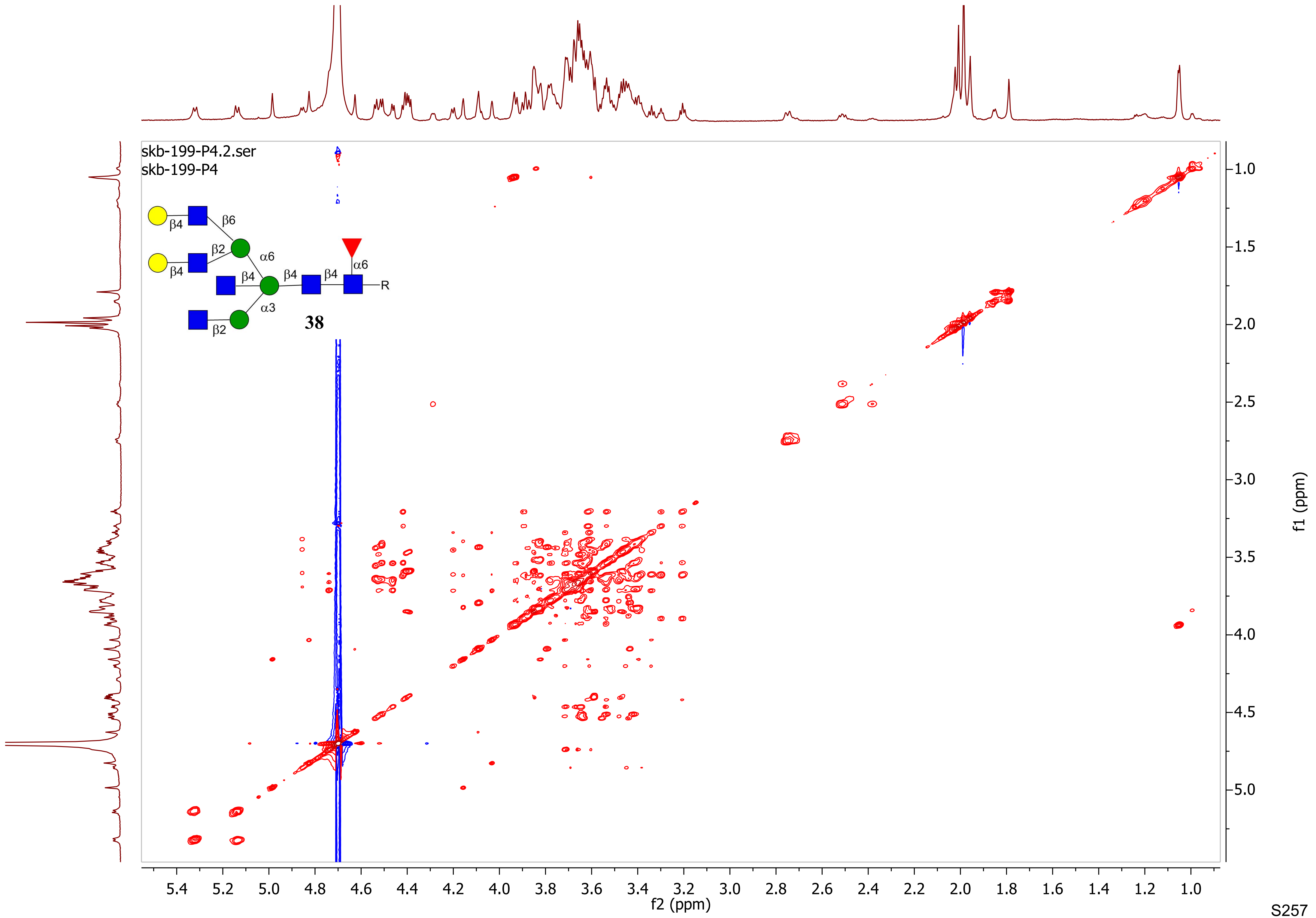

skb-199-P4.2.ser
skb-199-P4
β4
β6
β2
α6
β4
β4
β4
α6
R
α3
β2
38
f2 (ppm)
f1 (ppm)

skb-202_P4.1.fid
skb-202_P4

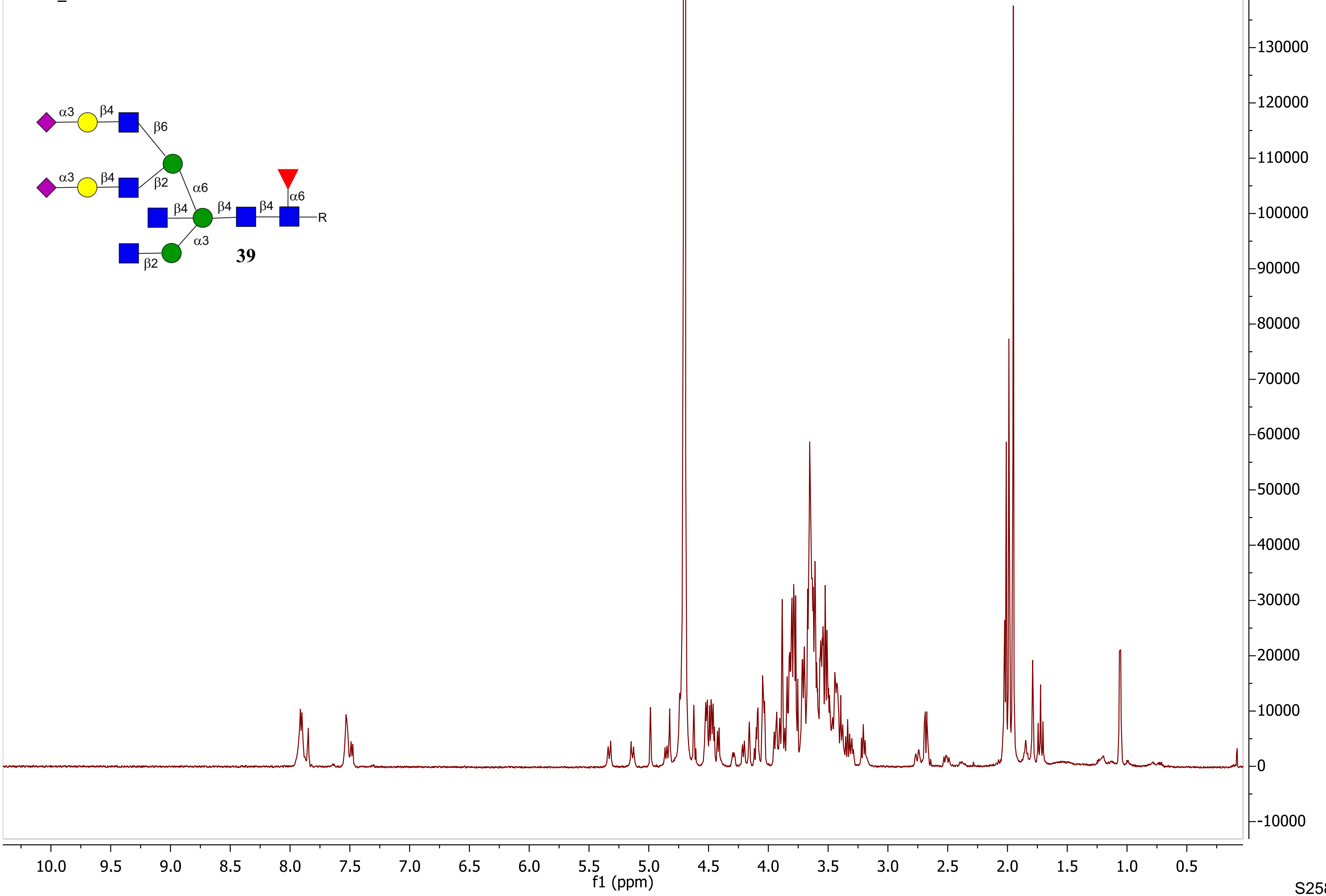

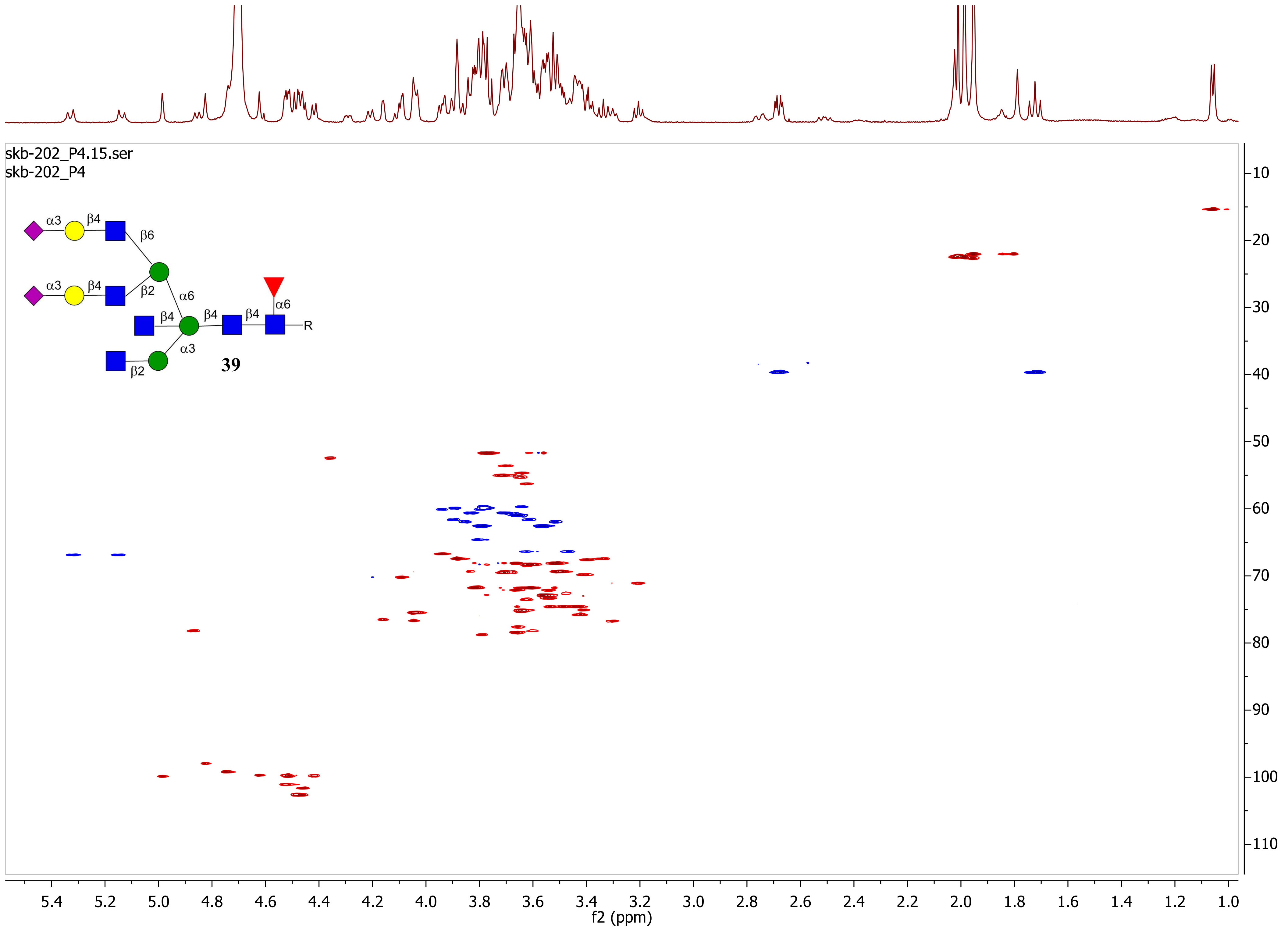
skb-202_P4.15.ser
skb-202_P4
α3
β4
β6
β2
α6
β4
β4
β4
α6
R
α3
β2
39
f2 (ppm)
f1 (ppm)

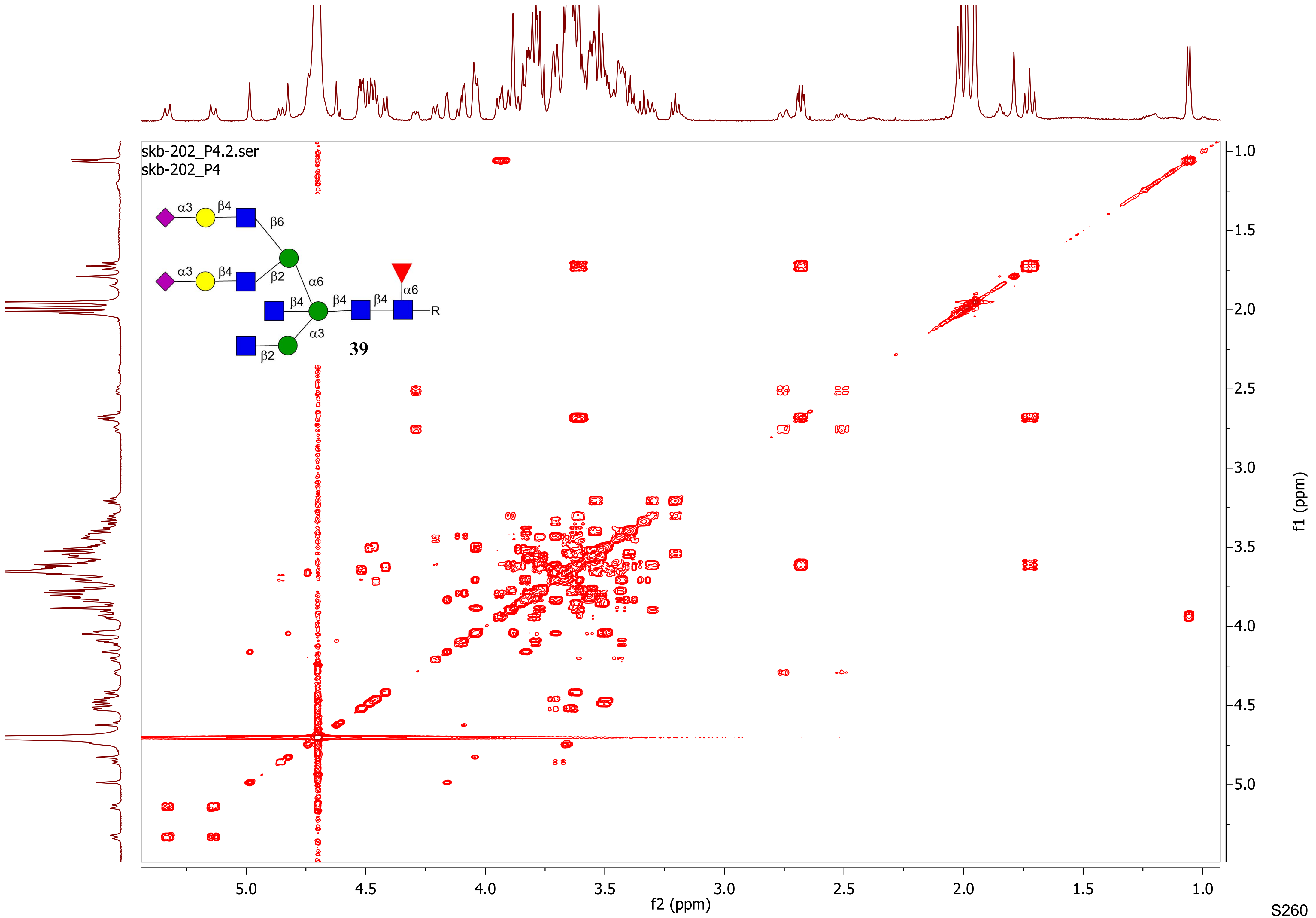

skb-202_P4.2.ser
skb-202_P4
α3
β4
β6
α3
β4
β2
α6
β4
β4
β4
α6
R
α3
β2
39
f2 (ppm)
f1 (ppm)

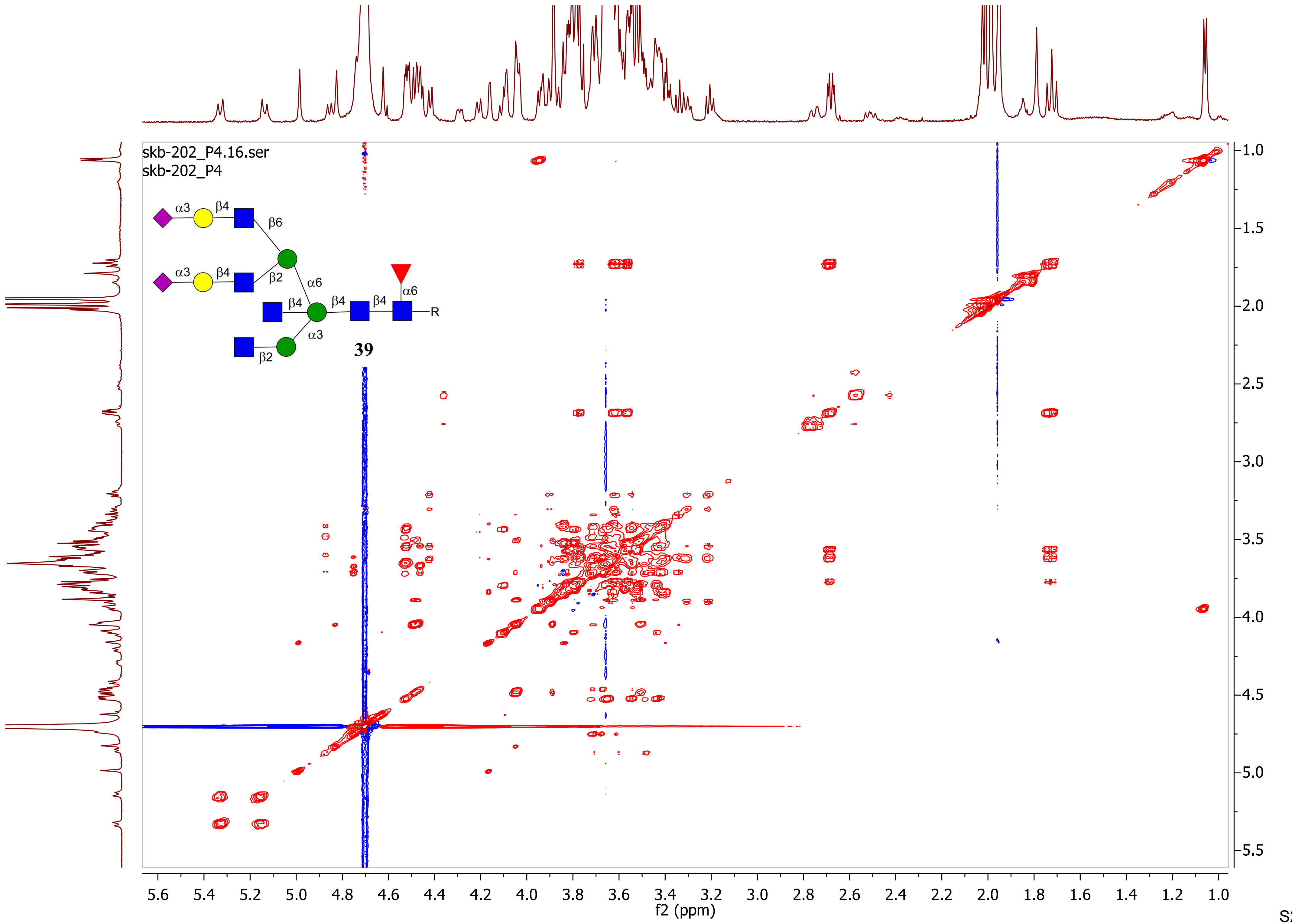
skb-202_P4.16.ser
skb-202_P4
α3
β4
β6
β2
α6
β4
β4
β4
α6
R
α3
β2
39
f2 (ppm)
f1 (ppm)

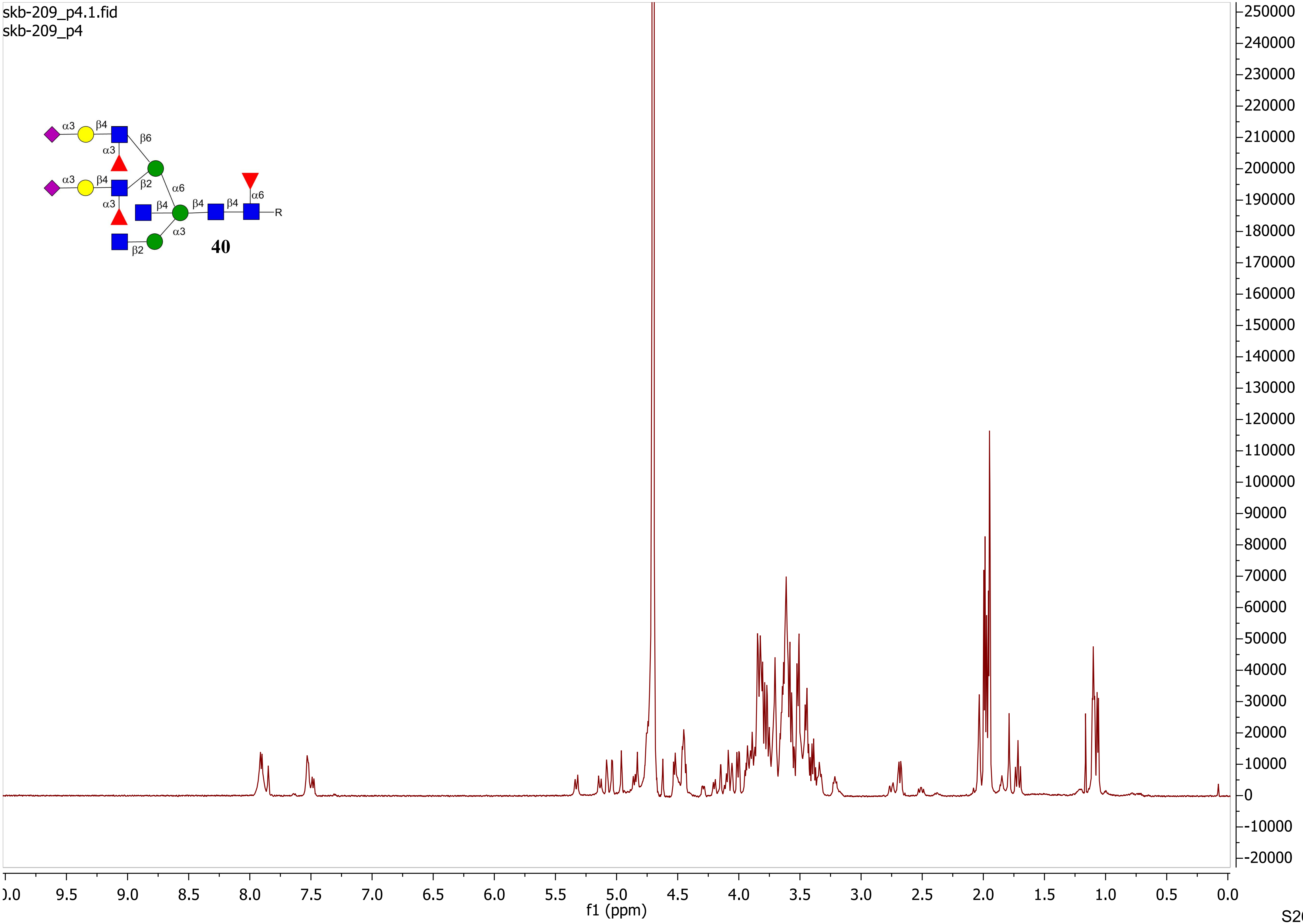
skb-209_p4.1.fid
skb-209_p4
α3
β4
β6
α3
α3
β4
β2
α6
α3
β4
β4
β4
α6
R
α3
β2
40
9.5
9.0
8.5
8.0
7.5
7.0
6.5
6.0
5.5
5.0
4.5
4.0
3.5
3.0
2.5
2.0
1.5
1.0
0.5
0.0
f1 (ppm)
250000
240000
230000
220000
210000
200000
190000
180000
170000
160000
150000
140000
130000
120000
110000
100000
90000
80000
70000
60000
50000
40000
30000
20000
10000
0
-10000
-20000

skb-209_P4_2.5.fid
skb-209_P4

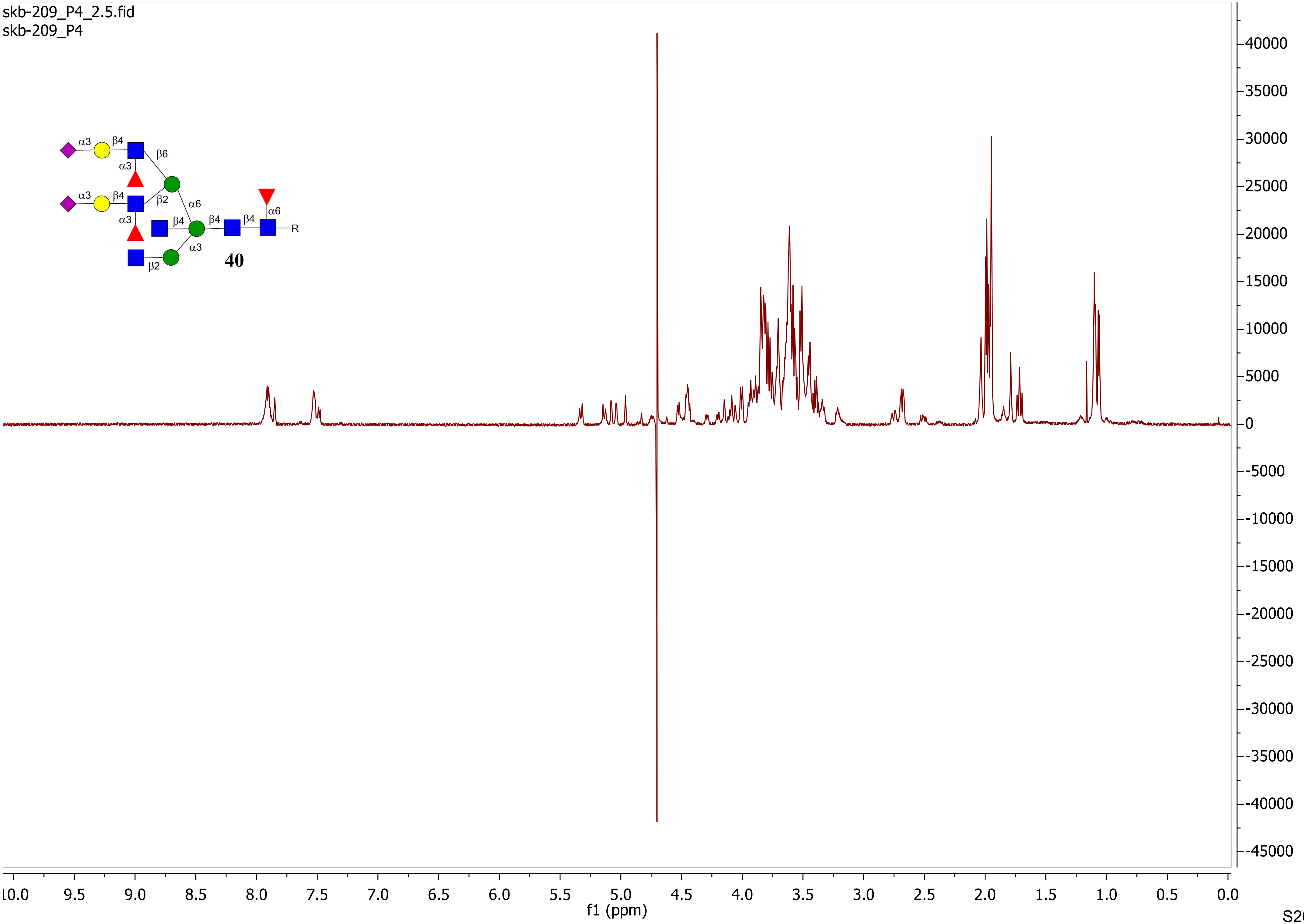

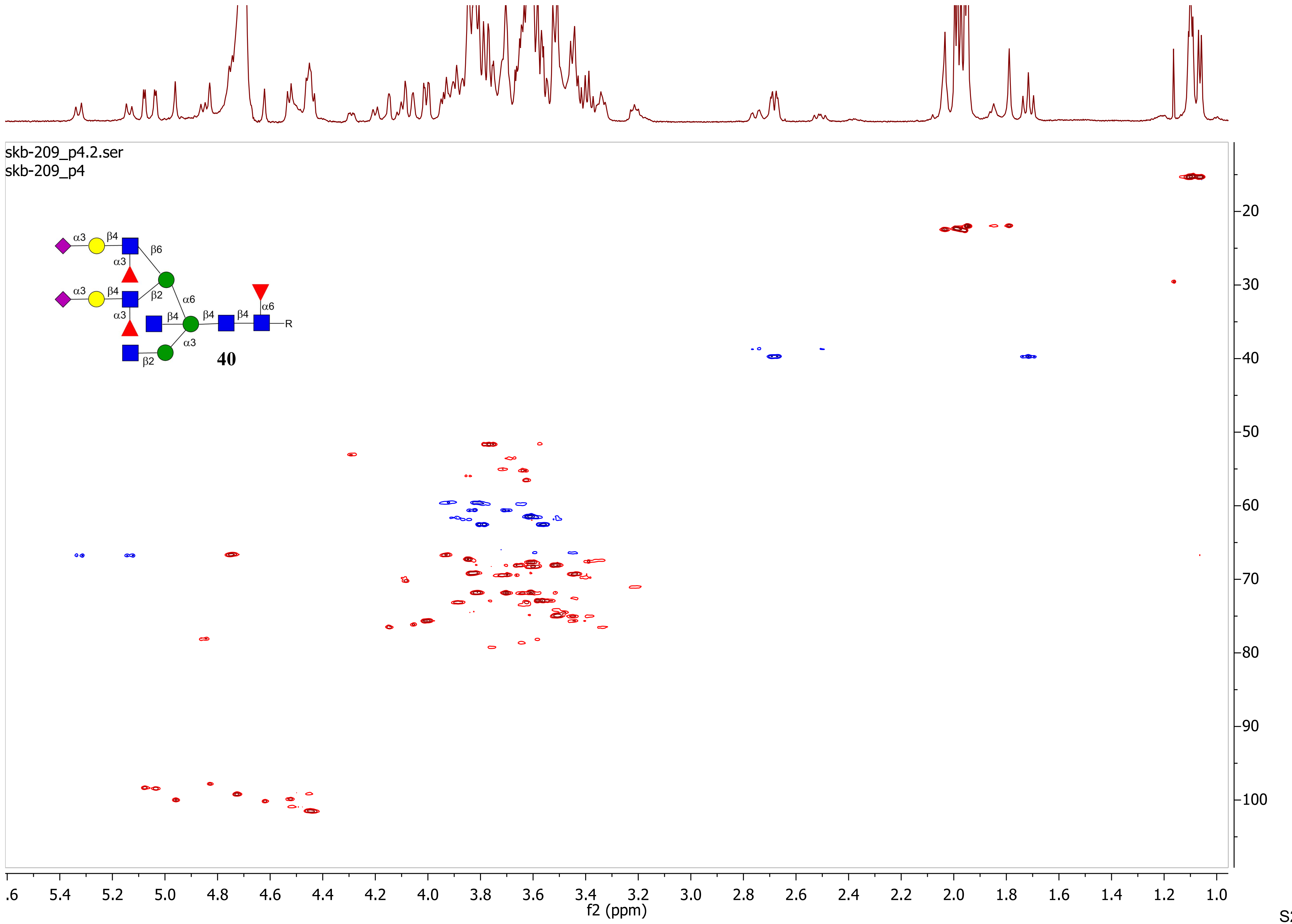
skb-209_p4.2.ser
skb-209_p4
α3
β4
β6
α3
α3
β4
β2
α6
α6
β4
β4
β4
R
α3
α3
β2
40
f2 (ppm)
f1 (ppm)

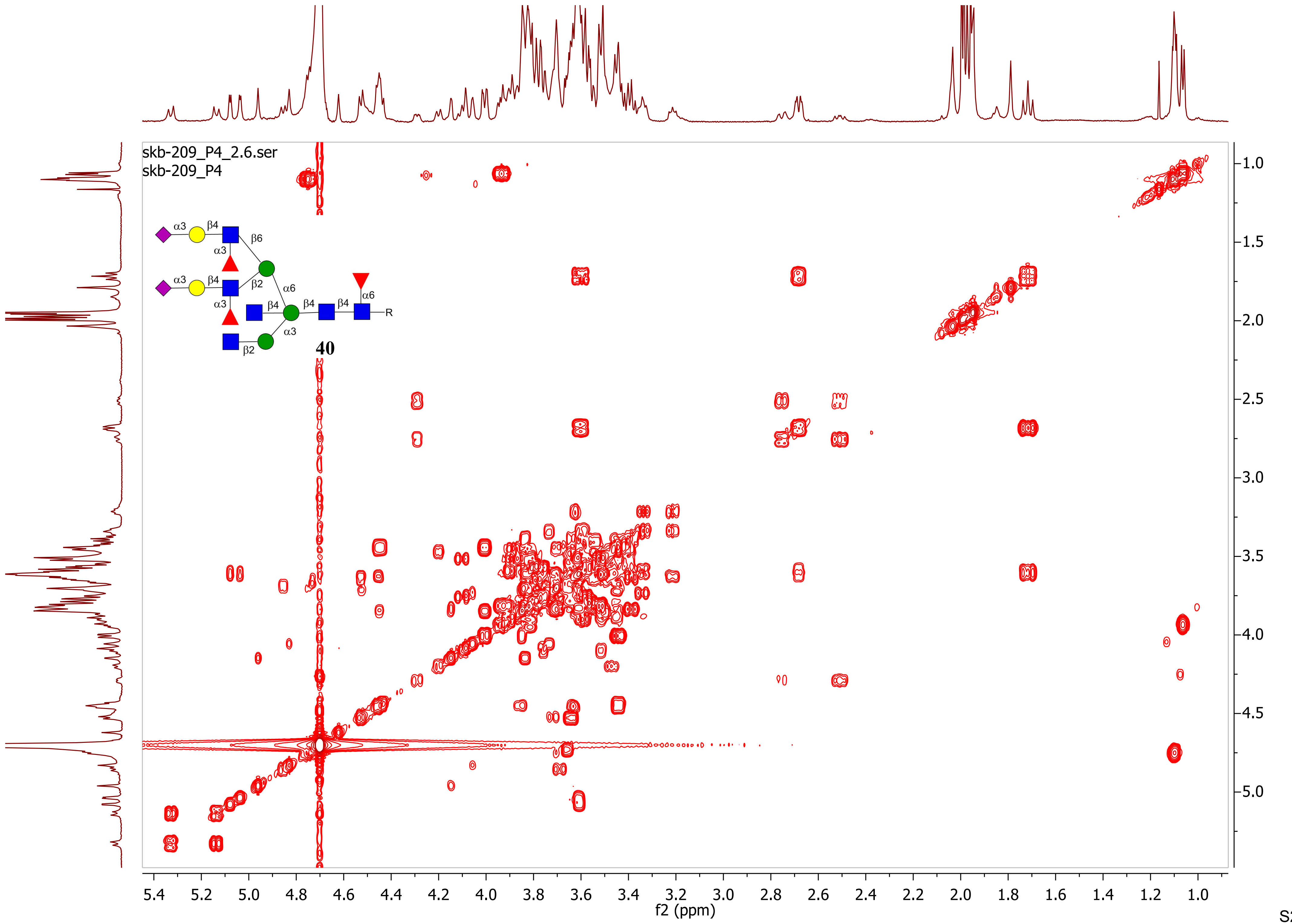

skb-209_P4_2.6.ser
skb-209_P4
α3
β4
β6
α3
α3
β4
β2
α6
α3
β4
β4
β4
α6
R
α3
β2
40
f2 (ppm)
f1 (ppm)

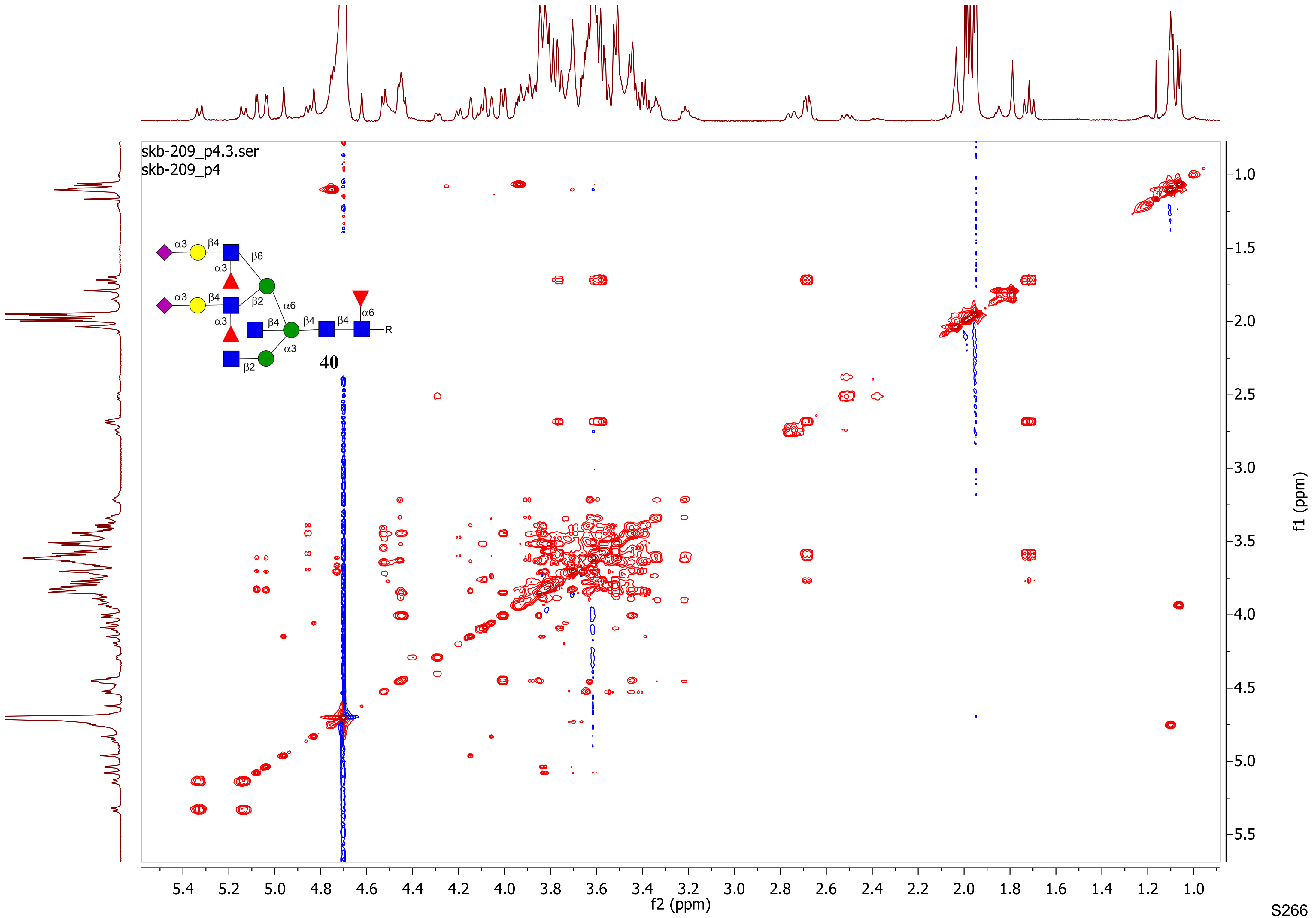
skb-209_p4.3.ser
skb-209_p4
α3
β4
β6
α3
α3
β4
β2
α6
α3
β4
β4
β4
α6
R
α3
β2
40
f2 (ppm)
f1 (ppm)

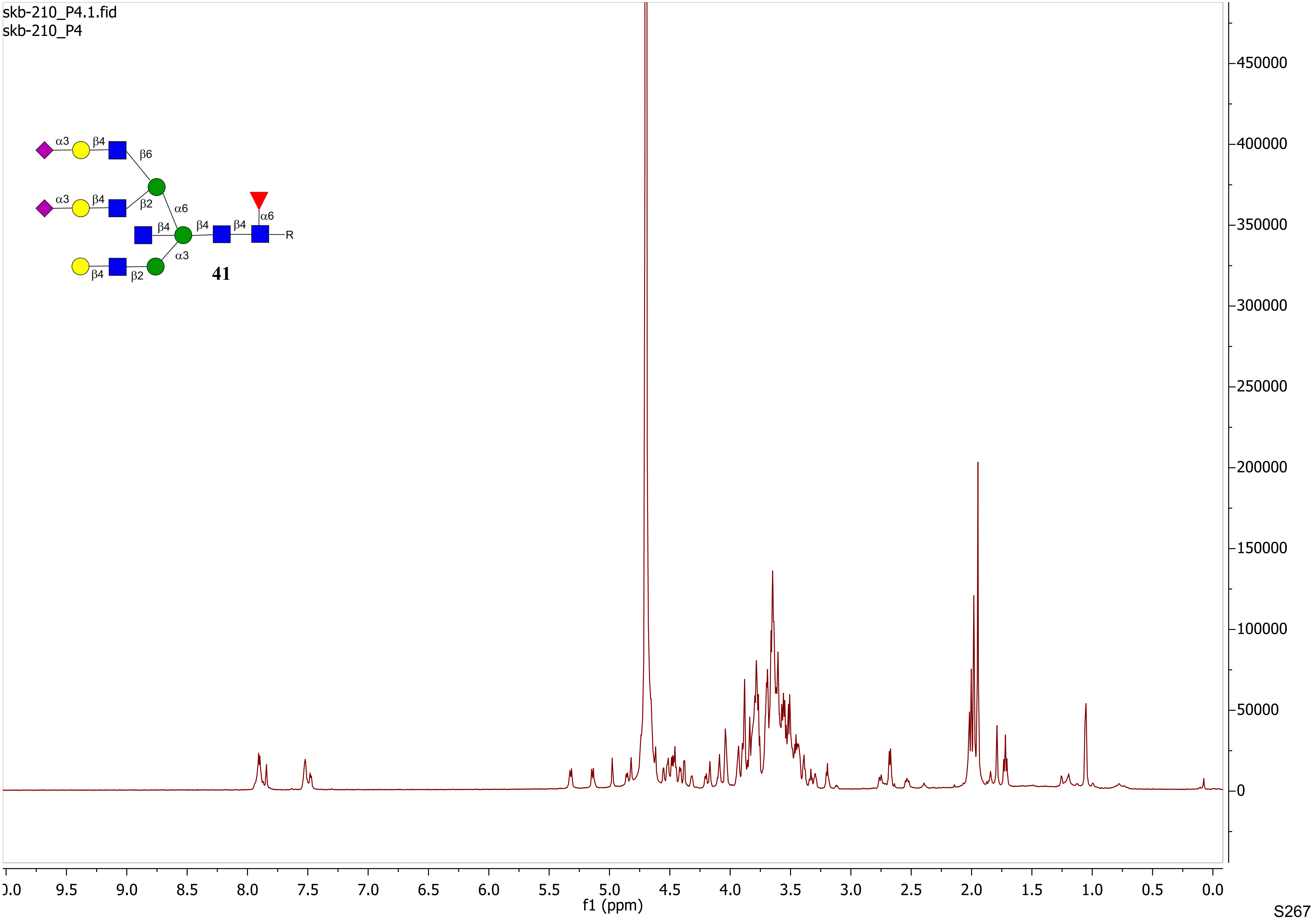
skb-210_P4.1.fid
skb-210_P4
α3
β4
β6
β2
α6
R
41
f1 (ppm)
450000
400000
350000
300000
250000
200000
150000
100000
50000
0
10.0
9.5
9.0
8.5
8.0
7.5
7.0
6.5
6.0
5.5
5.0
4.5
4.0
3.5
3.0
2.5
2.0
1.5
1.0
0.5
0.0

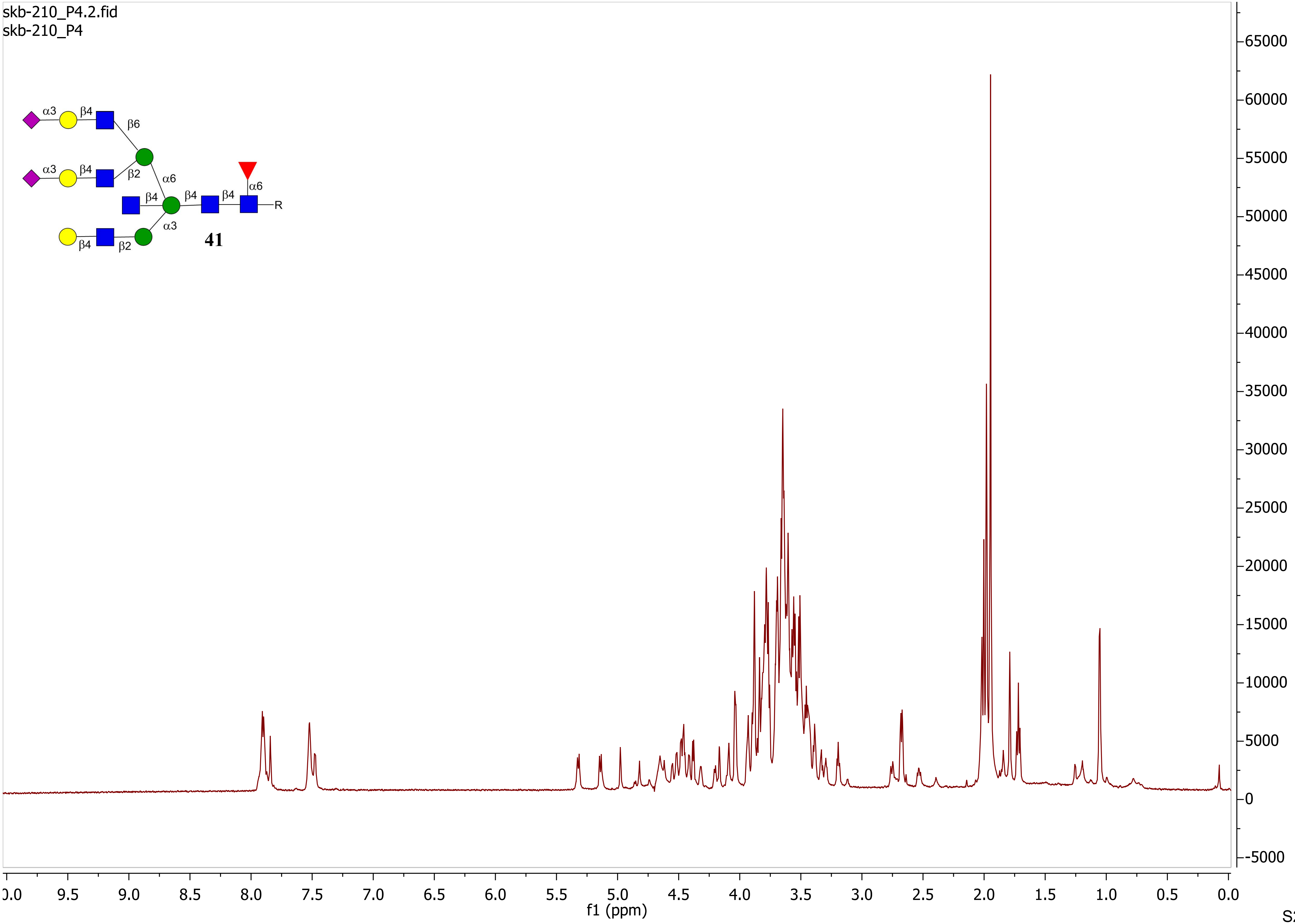
skb-210_P4.2.fid
skb-210_P4
α3
β4
β6
β2
α6
β4
β4
β4
α6
R
α3
β4
β2
41
f1 (ppm)

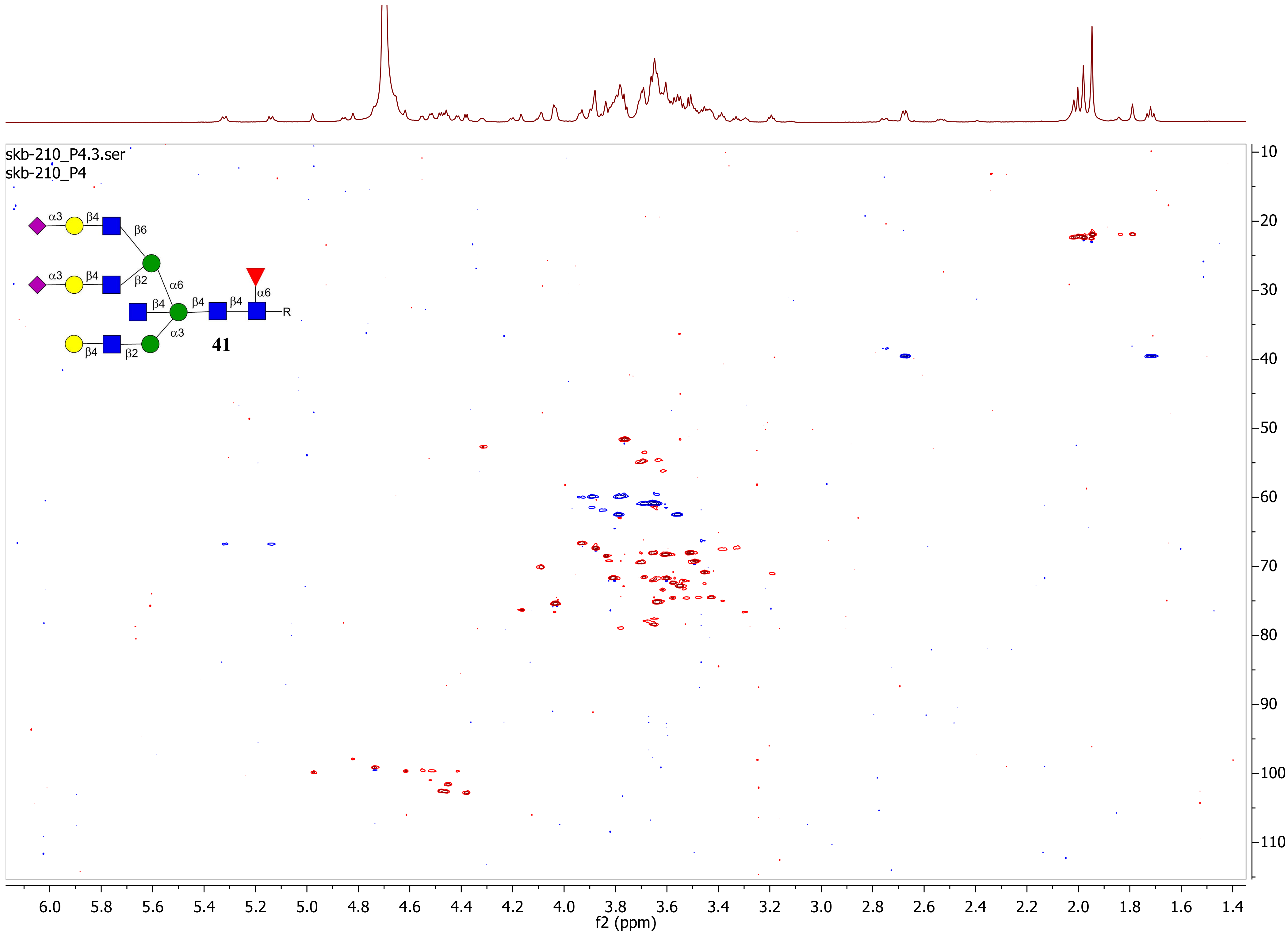
skb-210_P4.3.ser
skb-210_P4
α3
β4
β6
α3
β4
β2
α6
β4
β4
β4
α6
R
α3
β4
β2
41
f2 (ppm)
f1 (ppm)

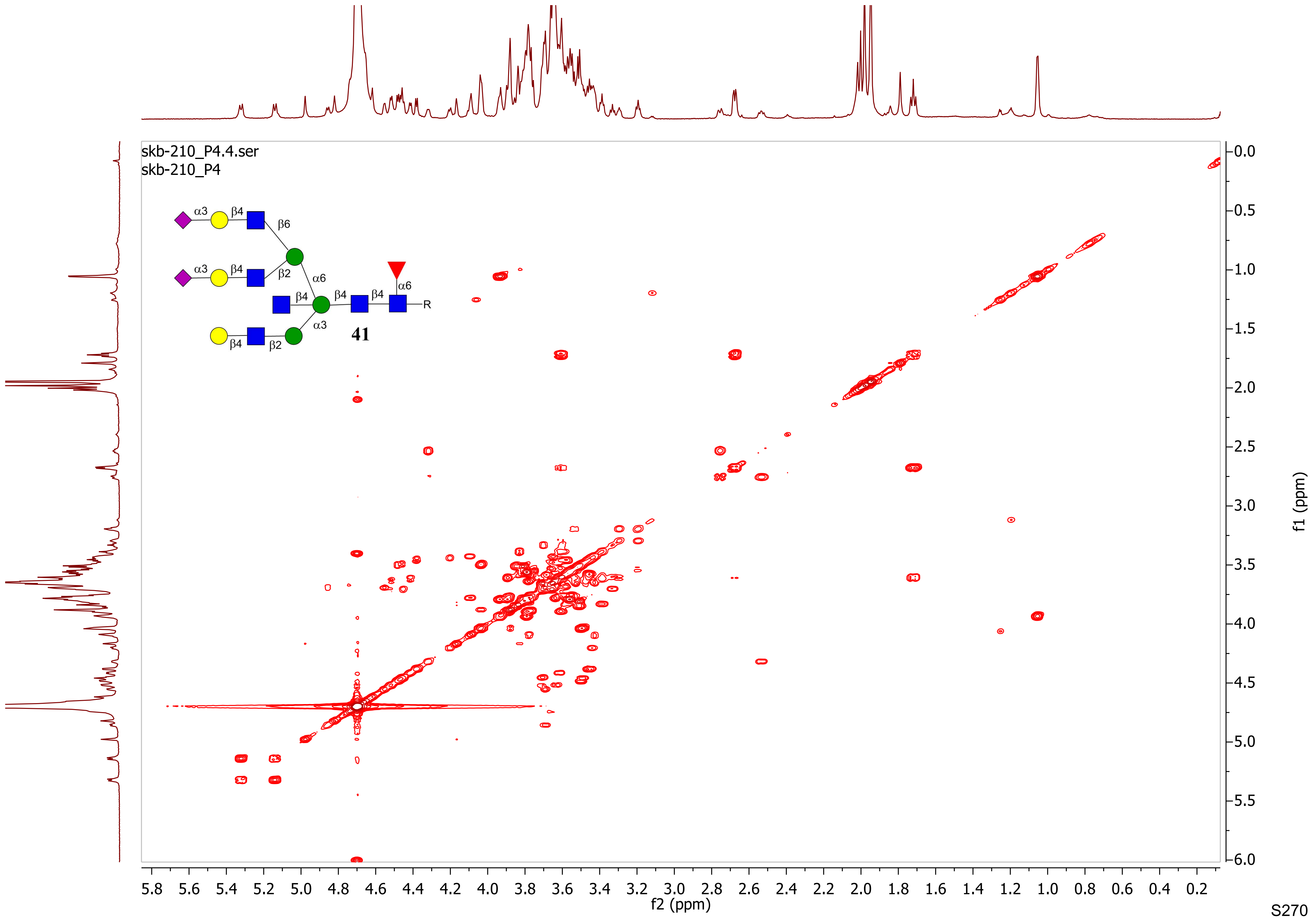
skb-210_P4.4.ser
skb-210_P4
α3
β4
β6
β2
α6
β4
β4
β4
α6
R
α3
β4
β2
41
f2 (ppm)
f1 (ppm)

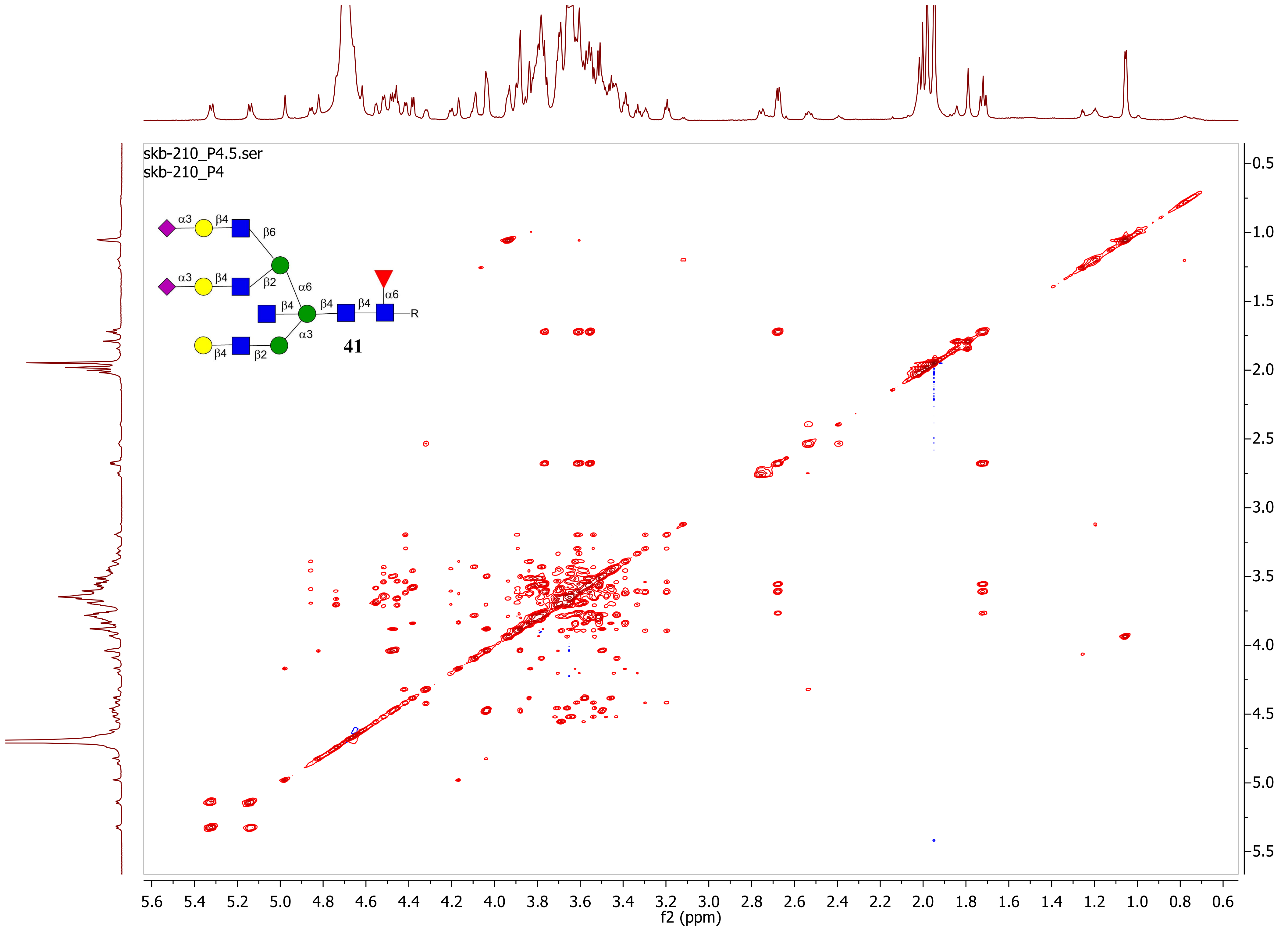
skb-210_P4.5.ser
skb-210_P4
α3
β4
β6
β2
α6
β4
β4
β4
α6
R
α3
β4
β2
41
f2 (ppm)
f1 (ppm)

SKB-235_P4.1.fid
SKB-235_P4

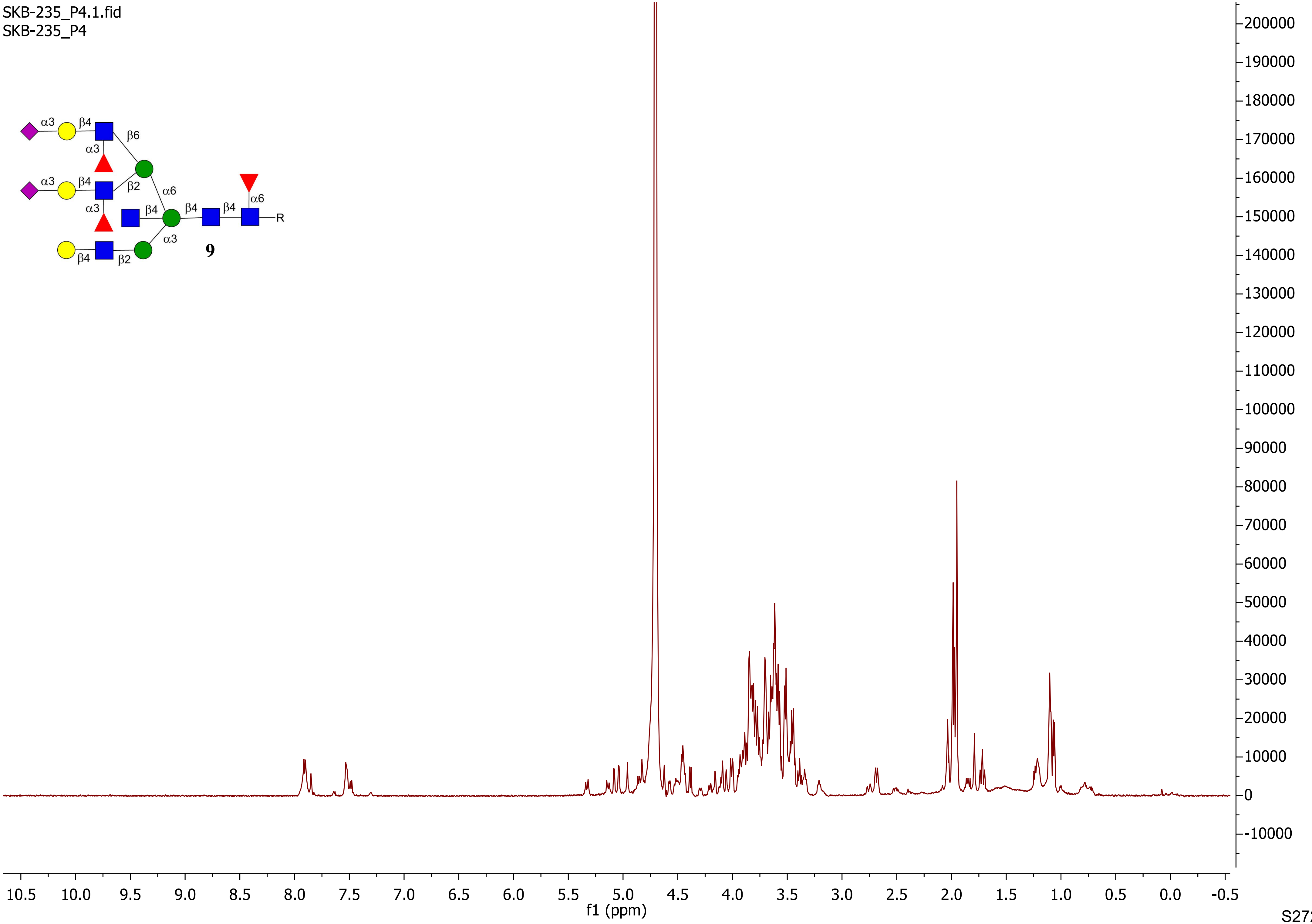

SKB-235_P4.2.fid
SKB-235_P4

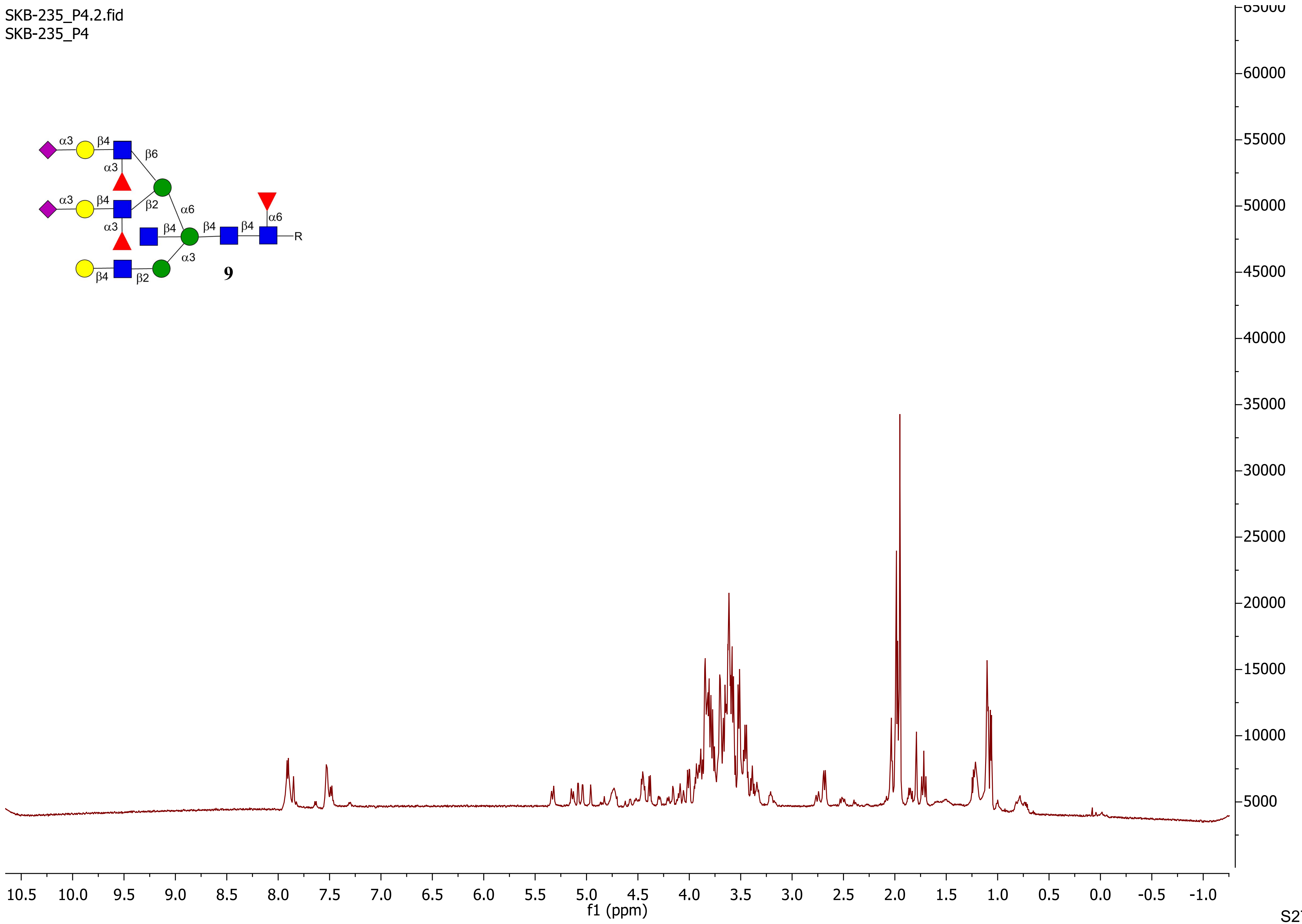

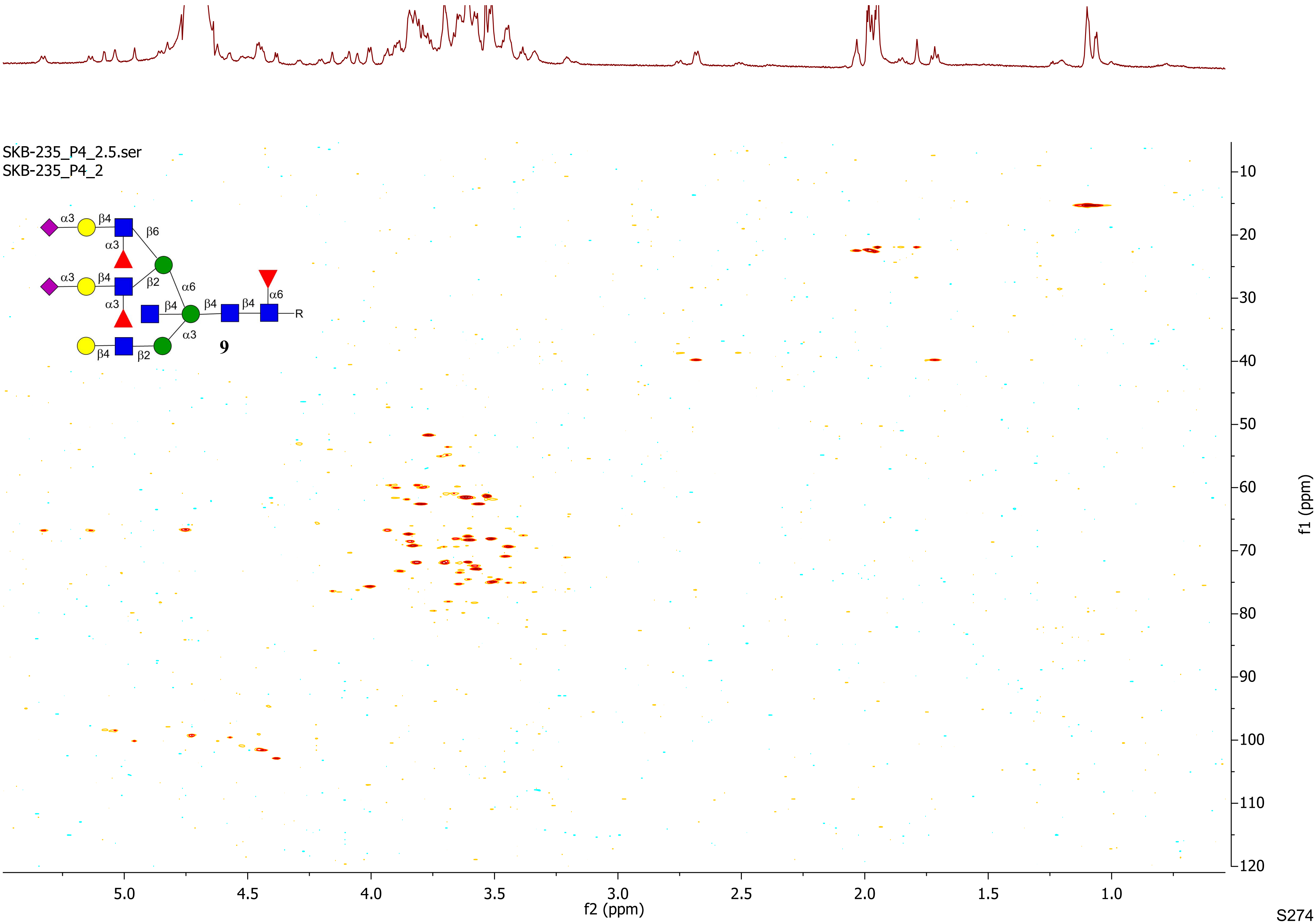

SKB-235_P4_2.5.ser
SKB-235_P4_2
α3
β4
β6
α3
α3
β4
β2
α6
α3
β4
β4
β4
α6
R
α3
β4
β2
9
f2 (ppm)
f1 (ppm)

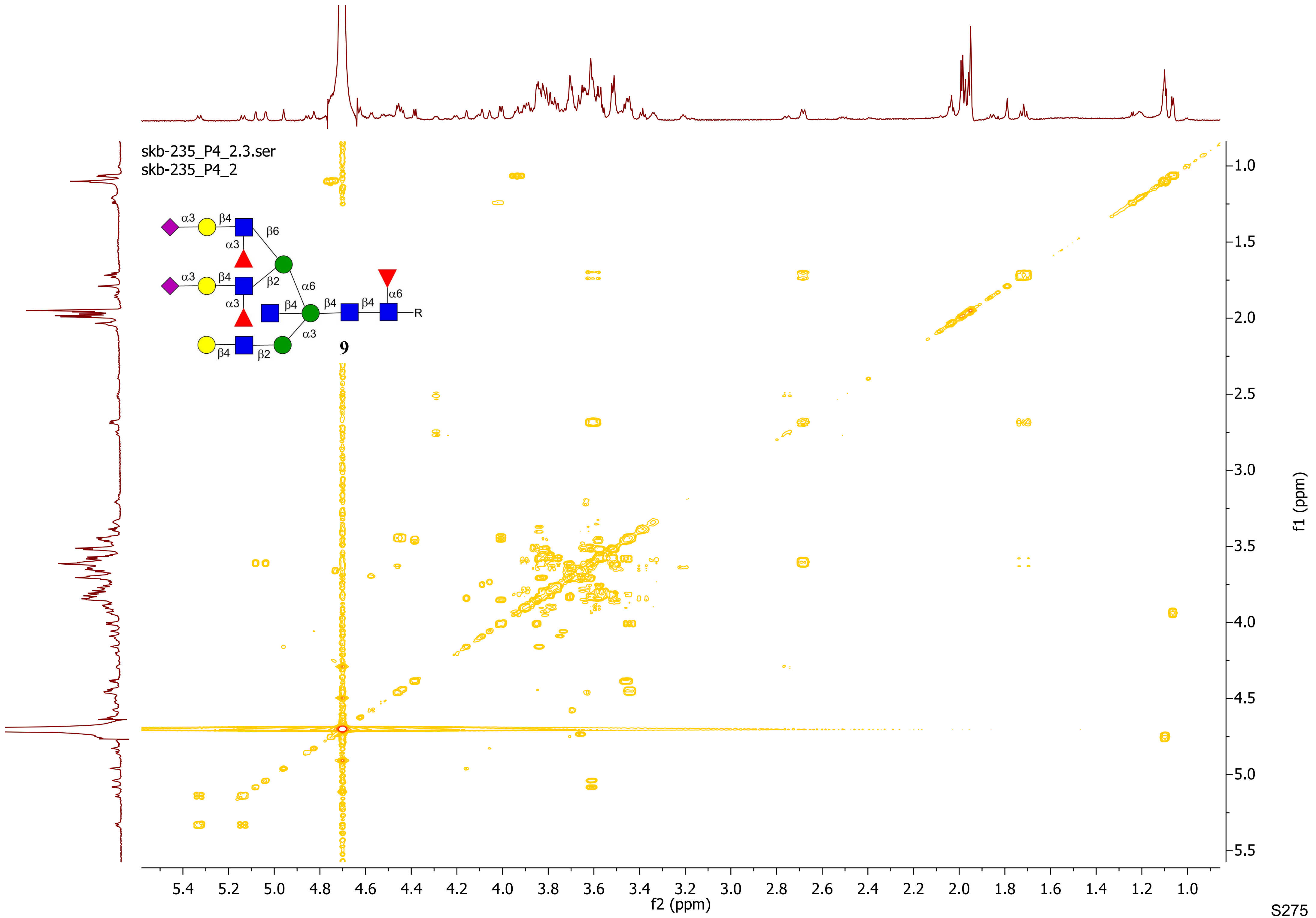

skb-235_P4_2.3.ser
skb-235_P4_2
α3
β4
β6
α3
α3
β4
β2
α6
α3
β4
β4
β4
α6
R
α3
β4
β2
9
f2 (ppm)
f1 (ppm)

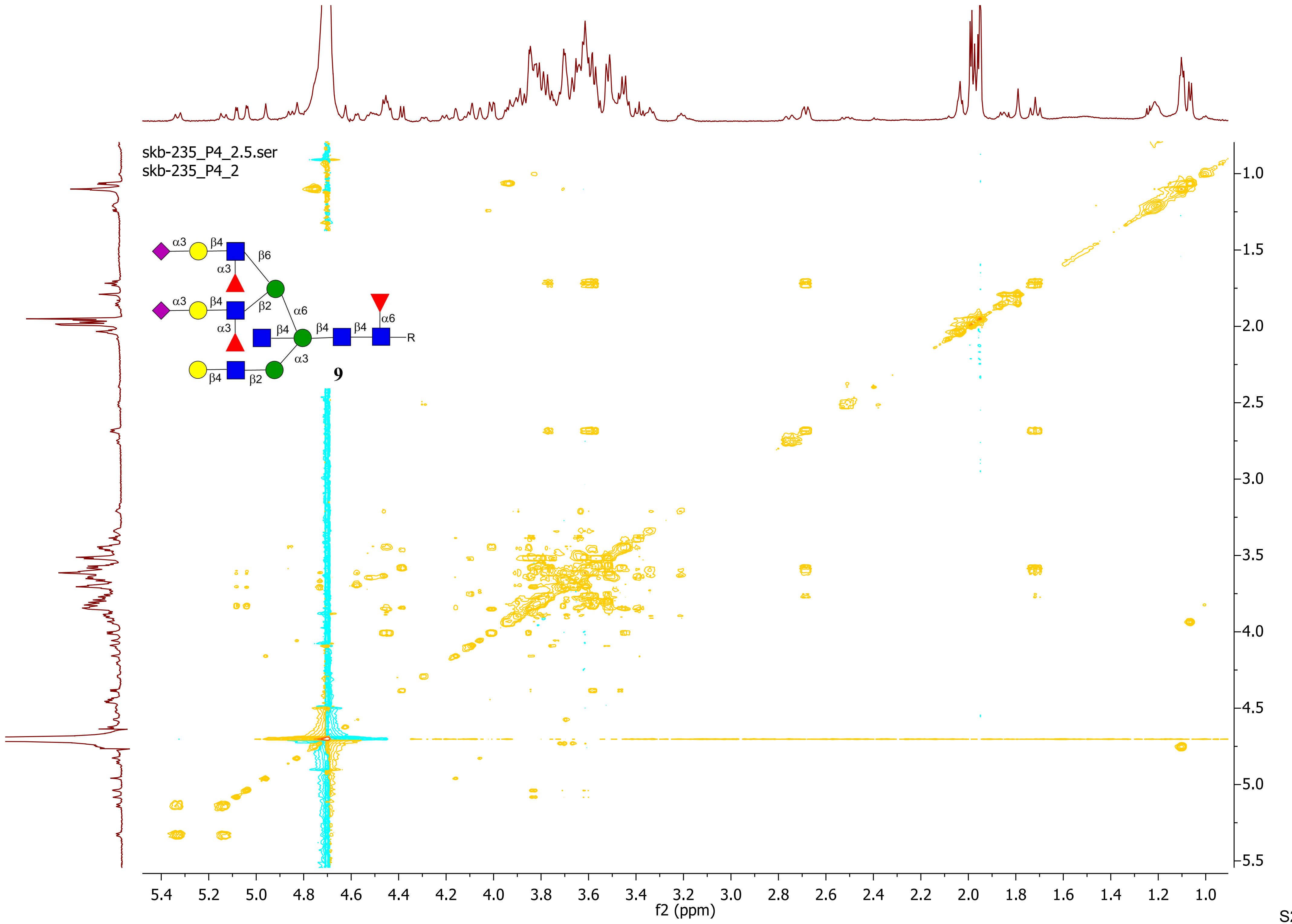
skb-235_P4_2.5.ser
skb-235_P4_2
9
α3
β4
β6
α3
β2
α6
β4
β4
β4
α6
R
α3
β2
f2 (ppm)
f1 (ppm)

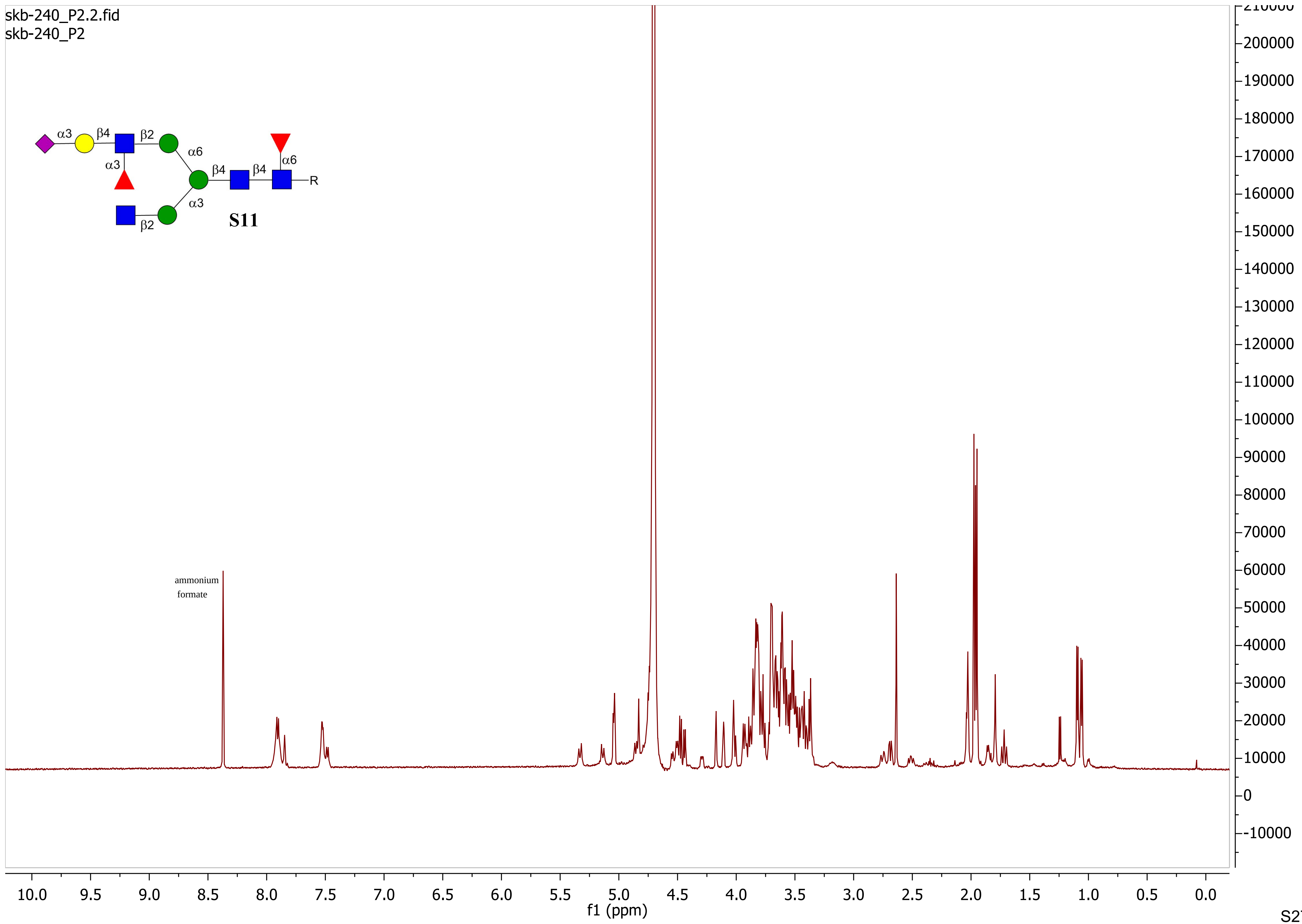

skb-240_P2.2.fid
skb-240_P2
α3
β4
β2
α6
α3
β4
β4
α6
R
α3
β2
S11
ammonium
formate
210000
200000
190000
180000
170000
160000
150000
140000
130000
120000
110000
100000
90000
80000
70000
60000
50000
40000
30000
20000
10000
0
-10000
10.0
9.5
9.0
8.5
8.0
7.5
7.0
6.5
6.0
5.5
5.0
4.5
4.0
3.5
3.0
2.5
2.0
1.5
1.0
0.5
0.0
f1 (ppm)

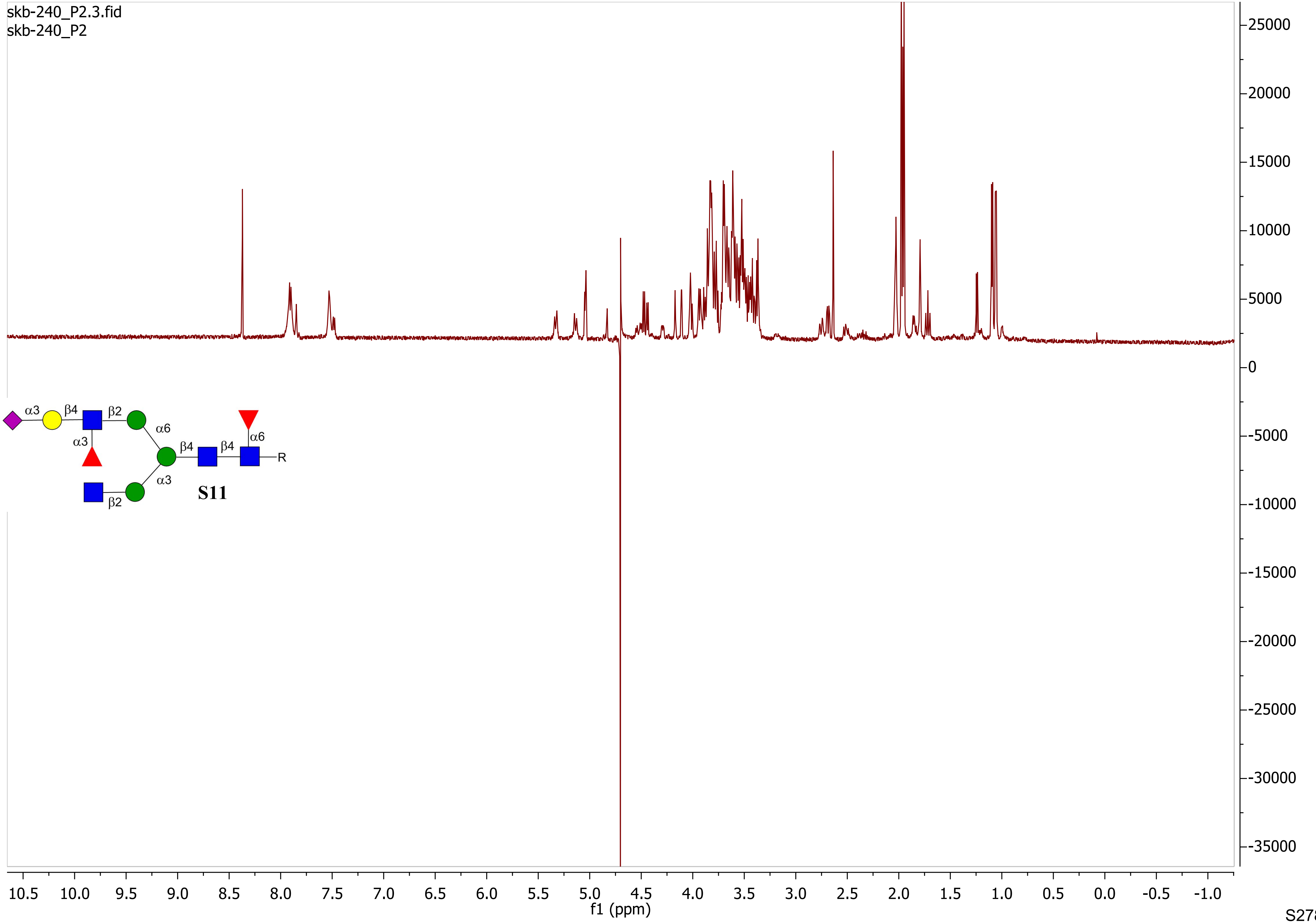

S11

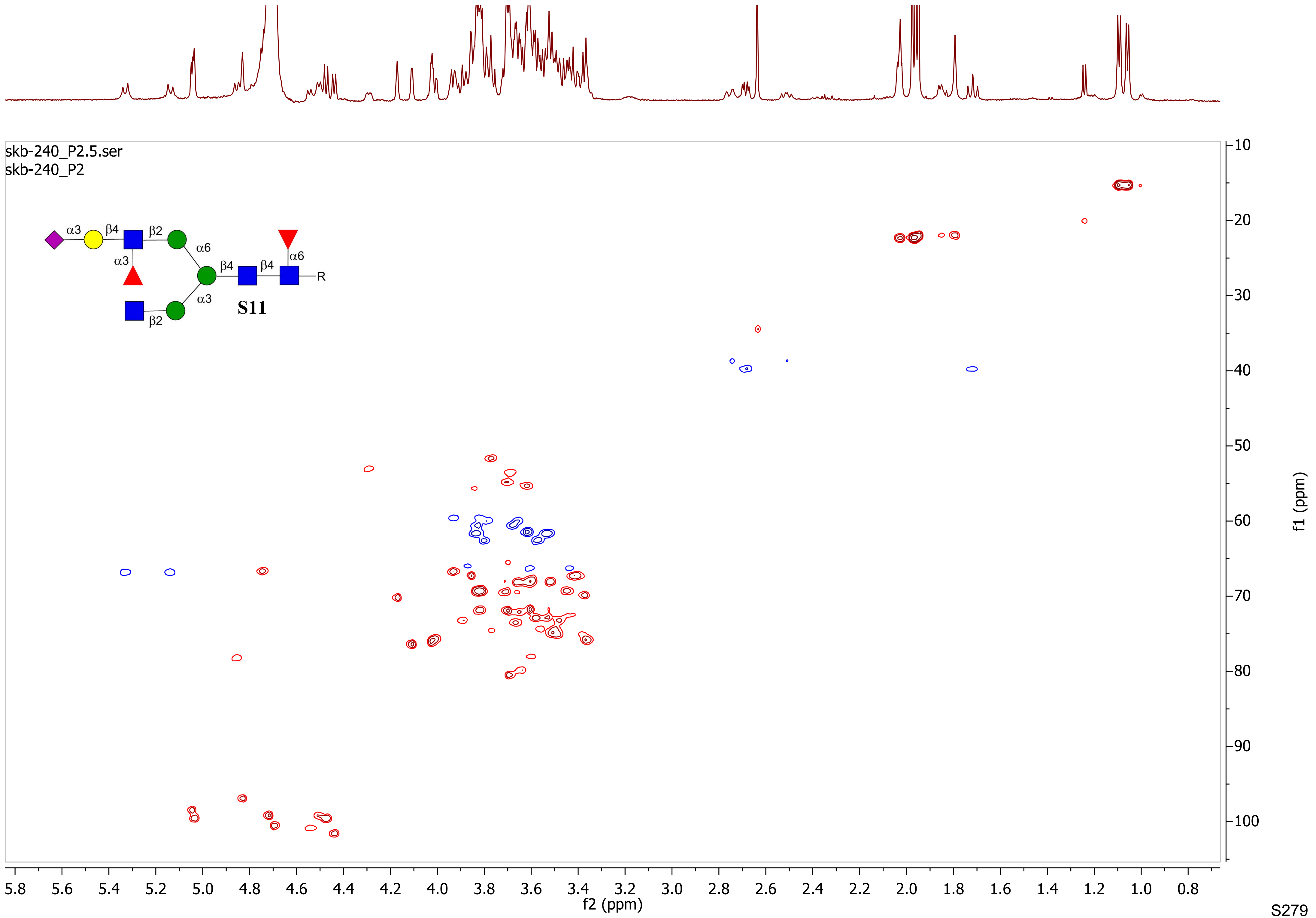

skb-240_P2.5.ser
skb-240_P2
α3
β4
β2
α6
α3
β4
β4
α6
R
α3
β2
S11
f1 (ppm)
f2 (ppm)

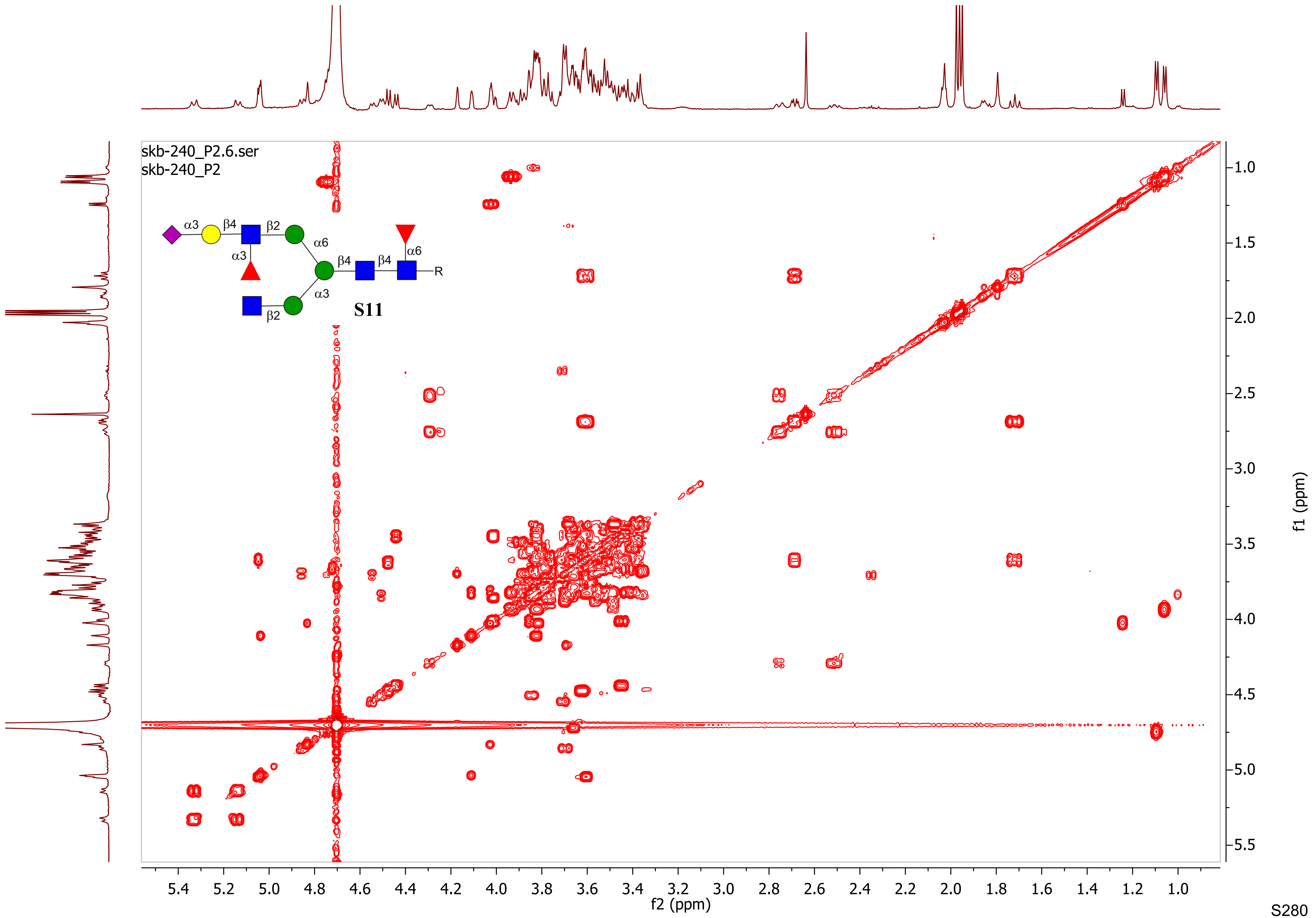
skb-240_P2.6.ser
skb-240_P2
α3
β4
β2
α6
α3
β4
β4
α6
R
α3
β2
S11
5.4
5.2
5.0
4.8
4.6
4.4
4.2
4.0
3.8
3.6
3.4
3.2
3.0
2.8
2.6
2.4
2.2
2.0
1.8
1.6
1.4
1.2
1.0
f2 (ppm)
1.0
1.5
2.0
2.5
3.0
3.5
4.0
4.5
5.0
5.5
f1 (ppm)

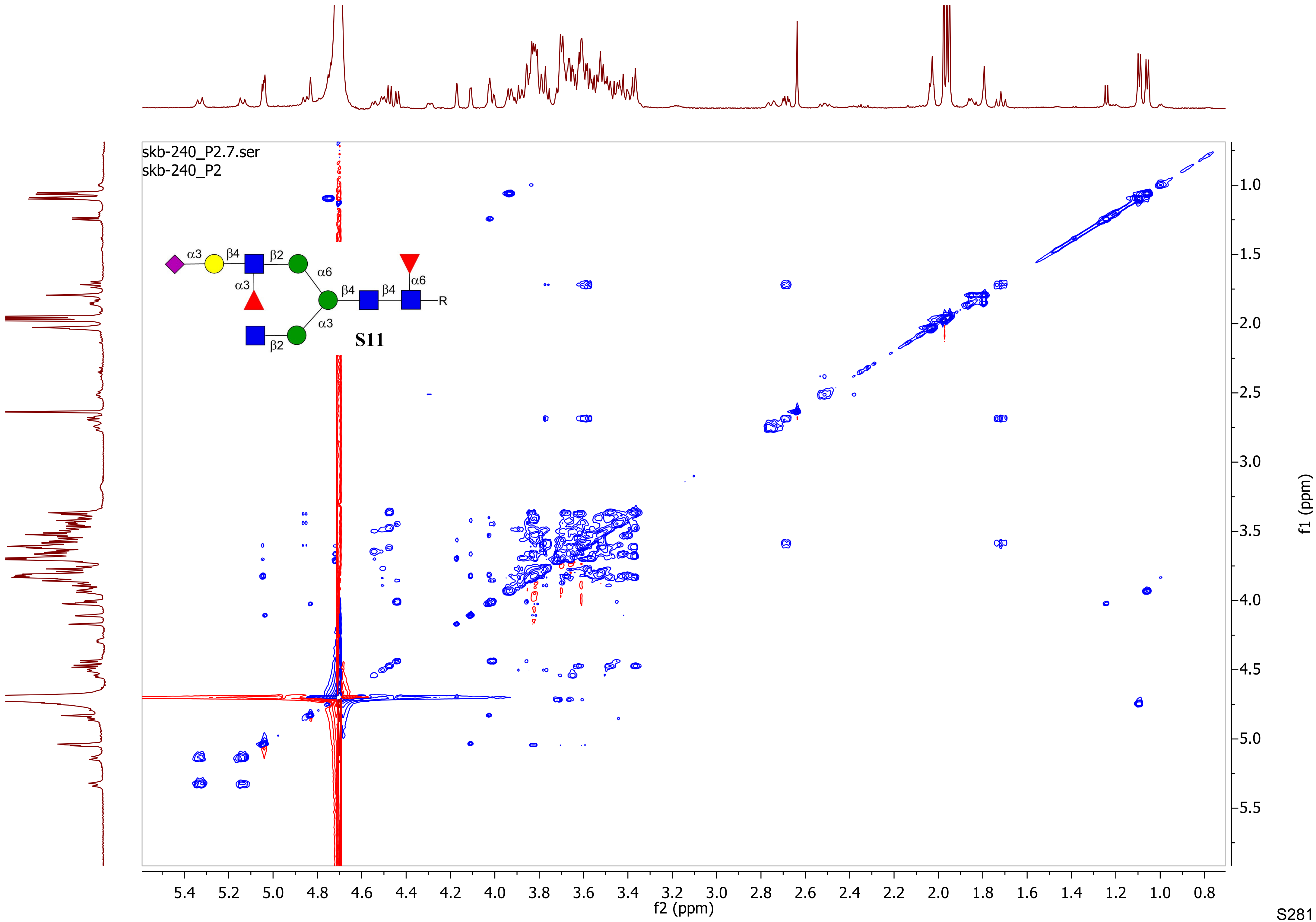

skb-240_P2.7.ser
skb-240_P2
α3
β4
β2
α6
α3
β4
β4
α6
R
α3
β2
S11
f2 (ppm)
f1 (ppm)

skb-241_P2.8.fid
skb-241_P2

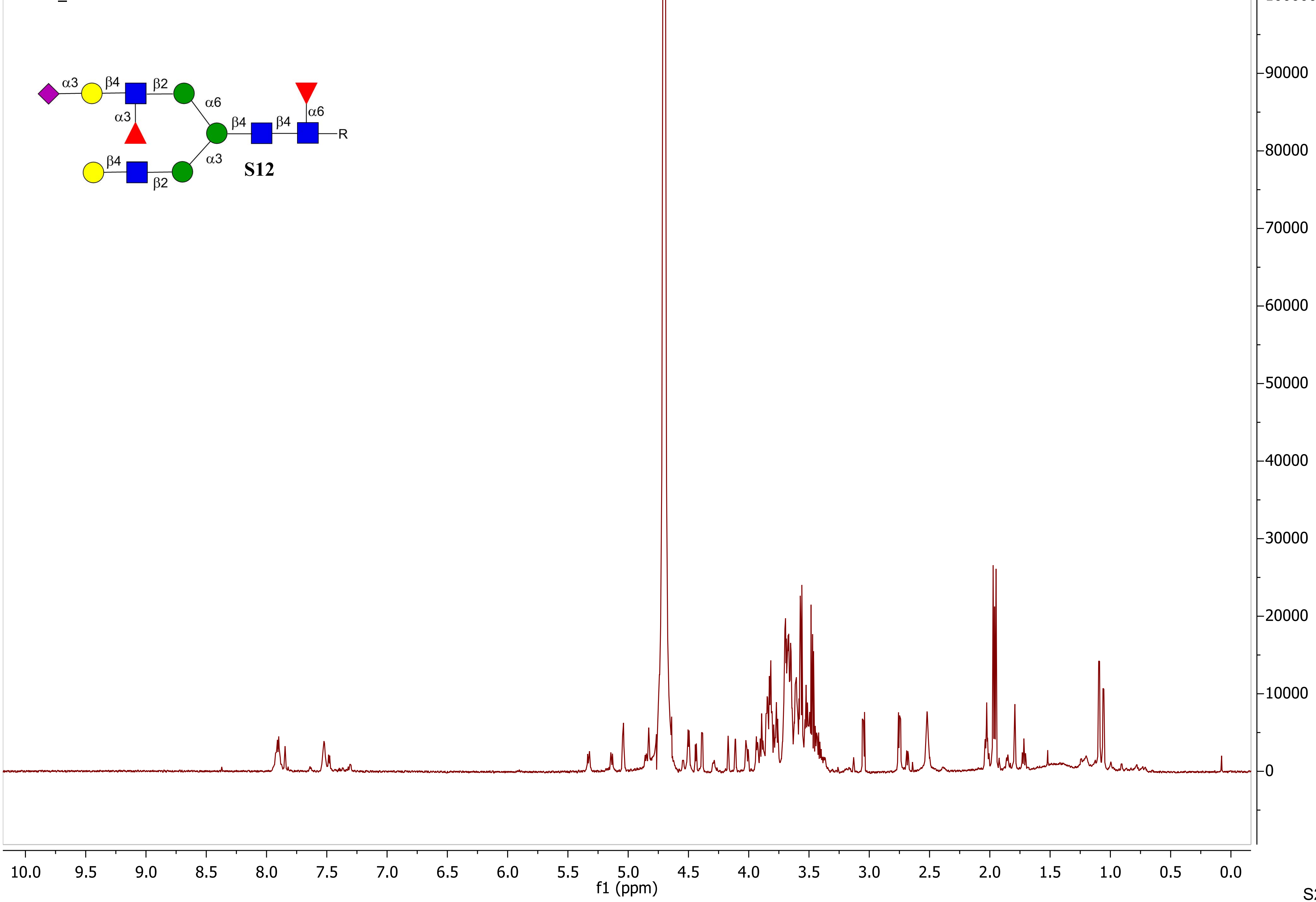

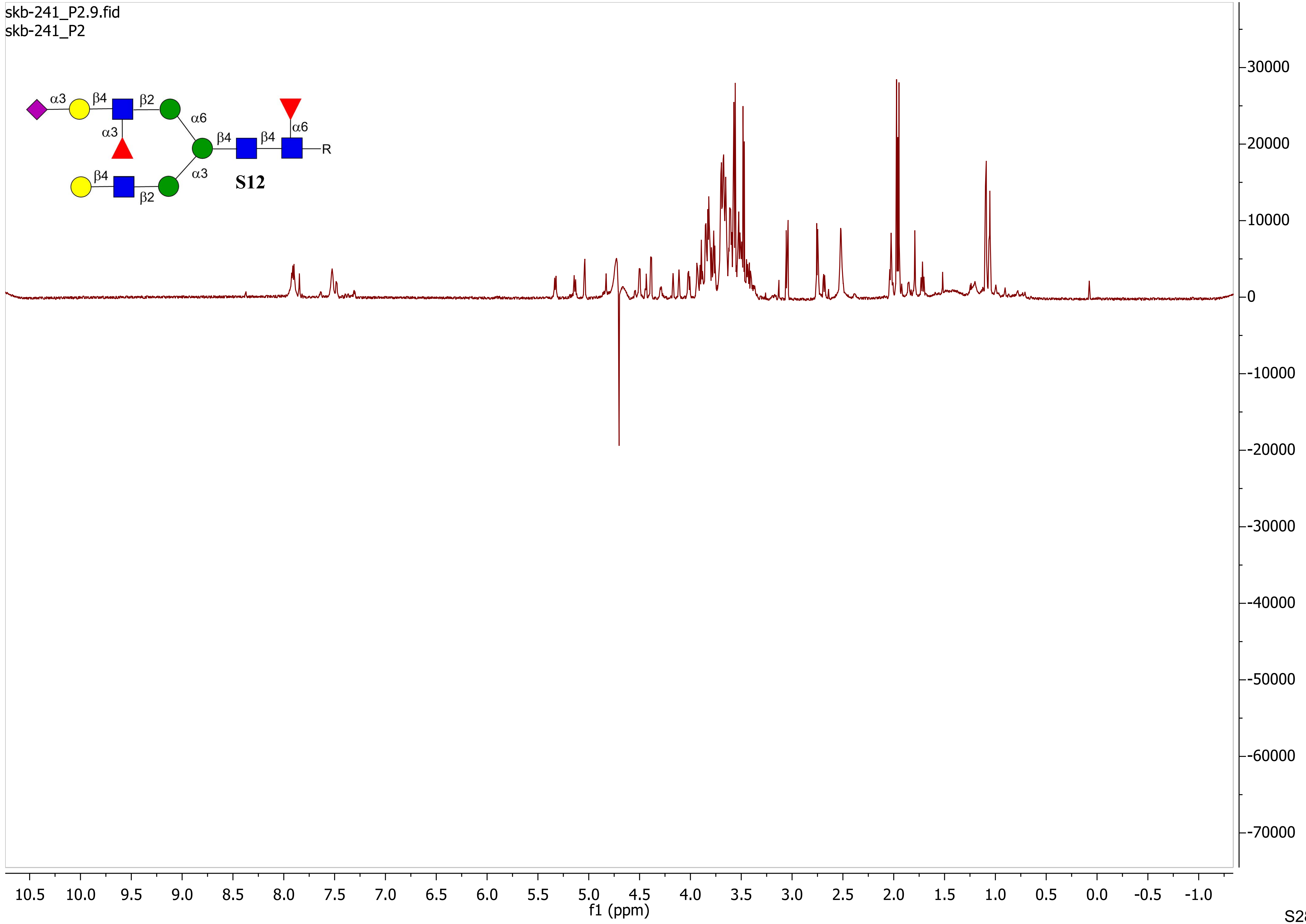
skb-241_P2.9.fid
skb-241_P2
α3
β4
β2
α6
α3
β4
β4
α6
R
α3
β4
β2
S12
f1 (ppm)

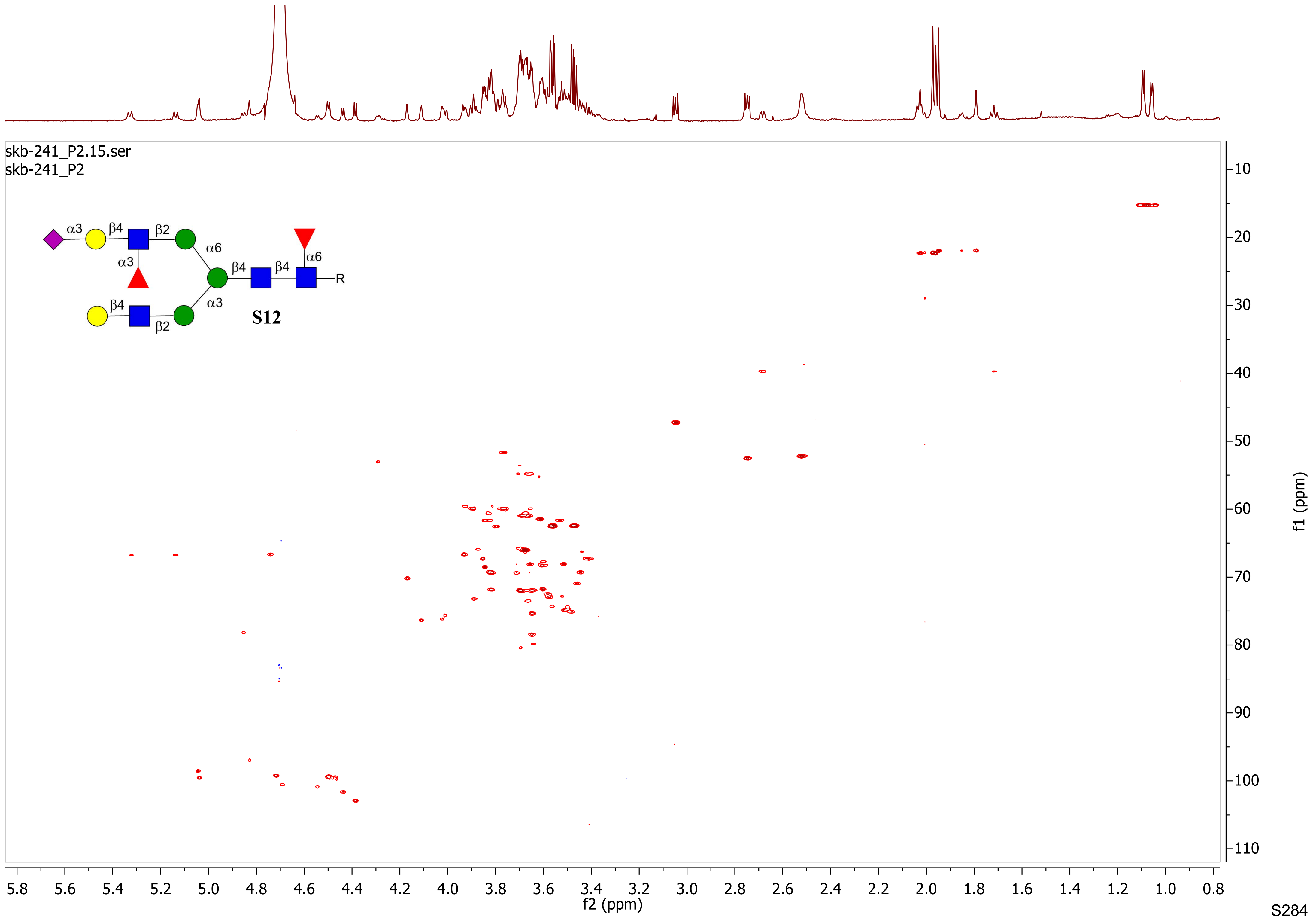

skb-241_P2.15.ser
skb-241_P2
α3
β4
β2
α6
α3
β4
β4
α6
R
β4
β2
α3
S12
f2 (ppm)
f1 (ppm)

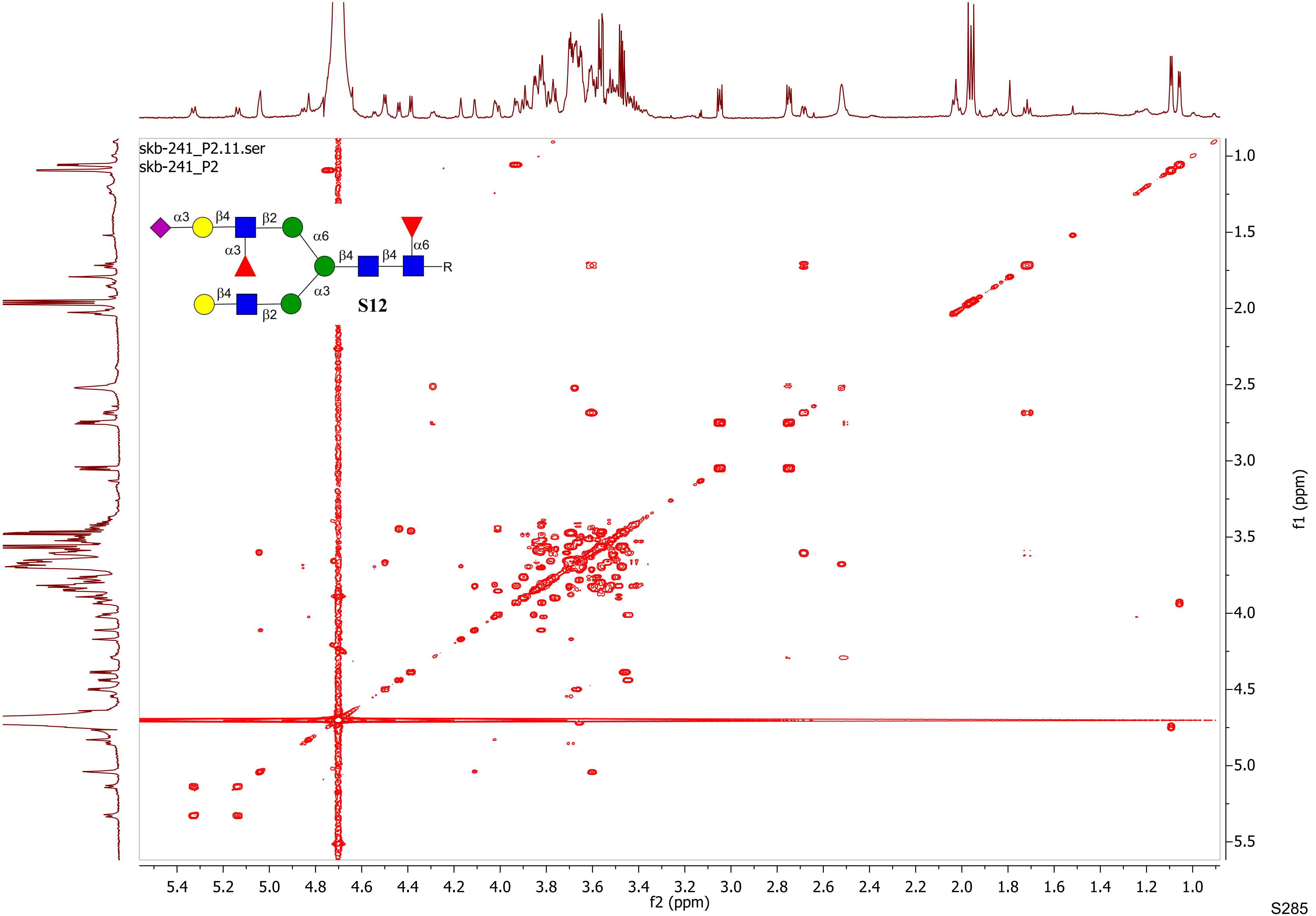
skb-241_P2.11.ser
skb-241_P2
α3
β4
β2
α6
α3
β4
β4
α6
R
α3
β4
β2
S12
f2 (ppm)
f1 (ppm)

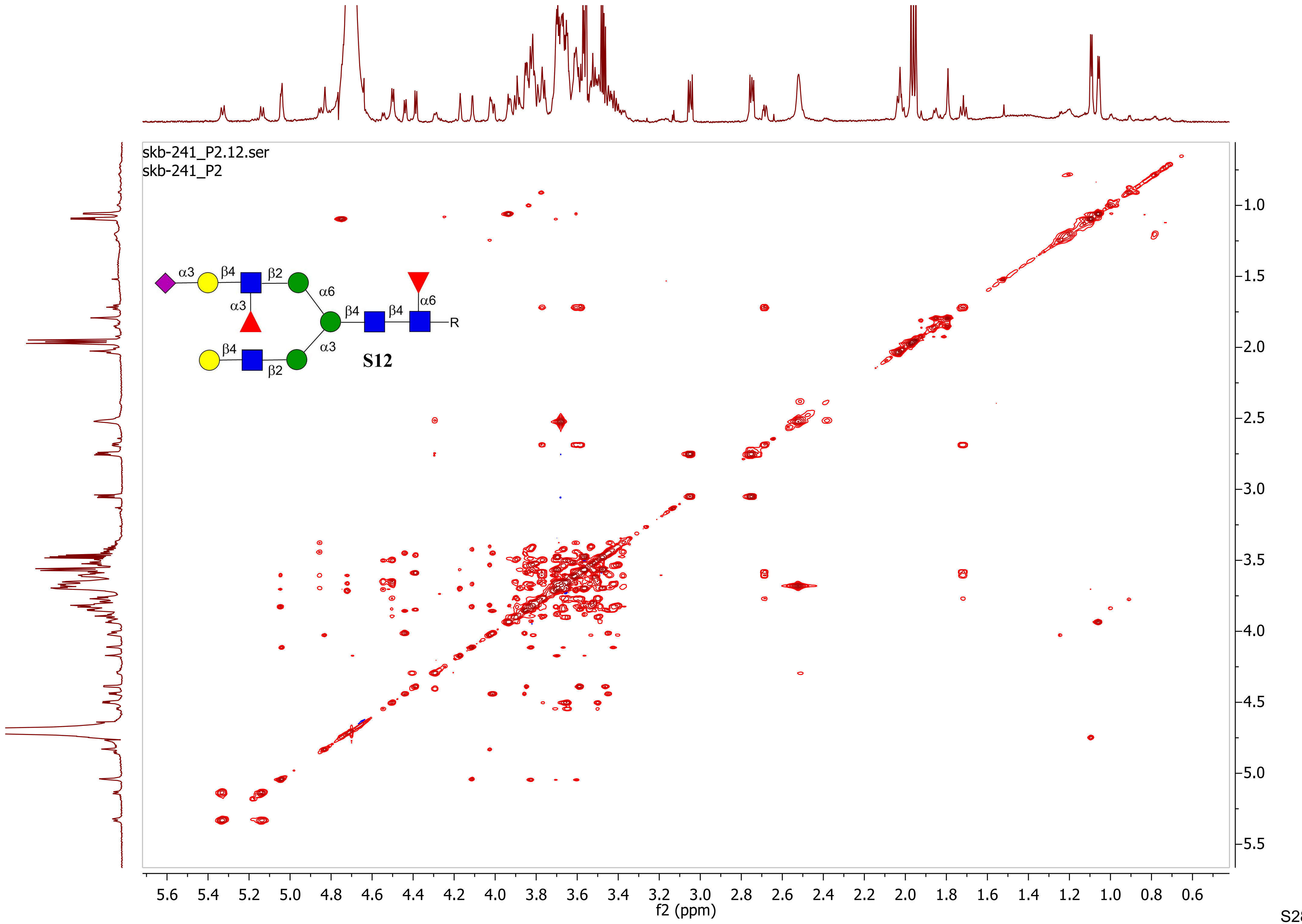
skb-241_P2.12.ser
skb-241_P2
α3
β4
β2
α6
α3
β4
β4
α6
R
α3
β4
β2
S12
f2 (ppm)
f1 (ppm)

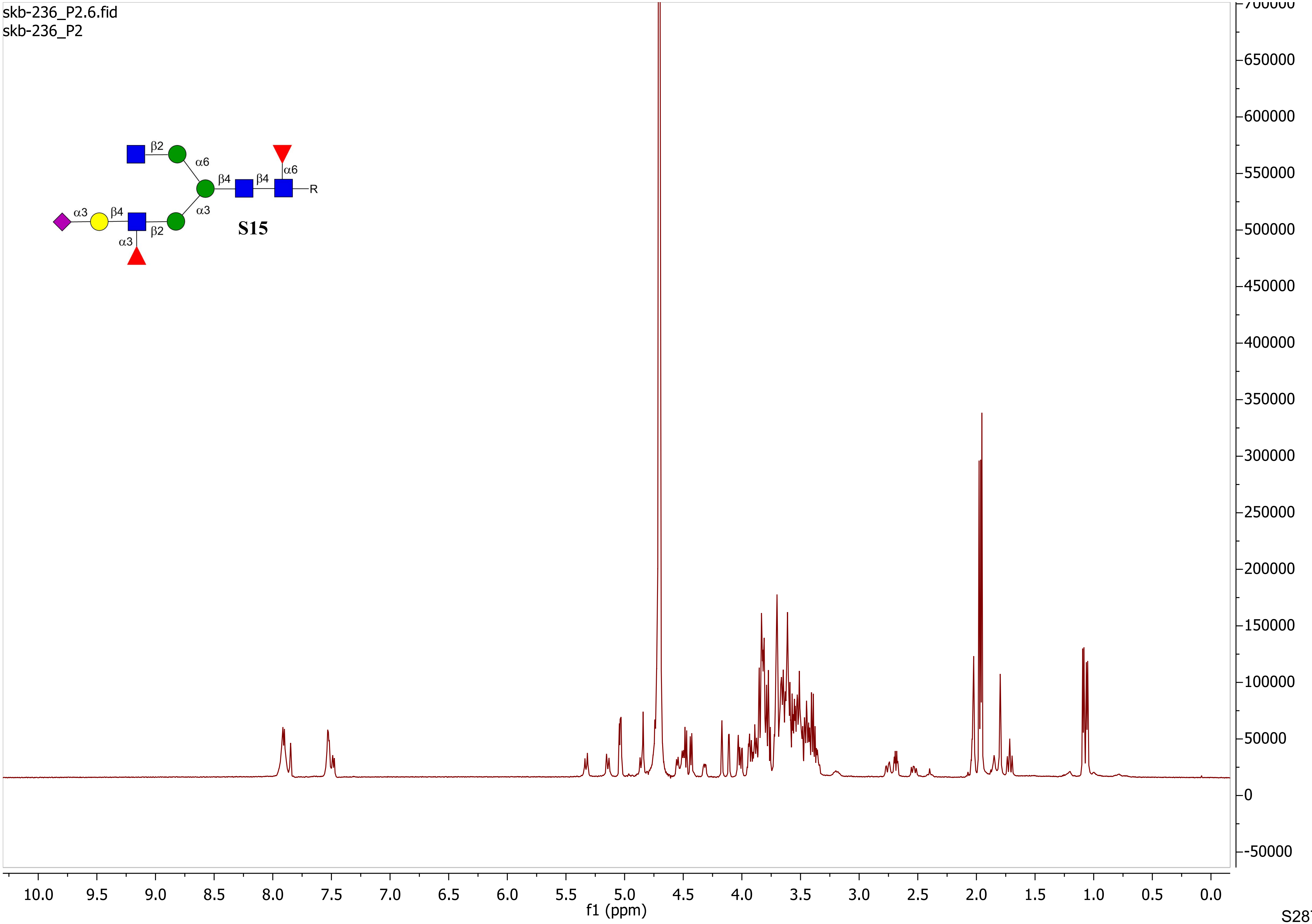
skb-236_P2.6.fid
skb-236_P2
β2
α6
β4
β4
α6
R
α3
β4
α3
β2
α3
S15
f1 (ppm)

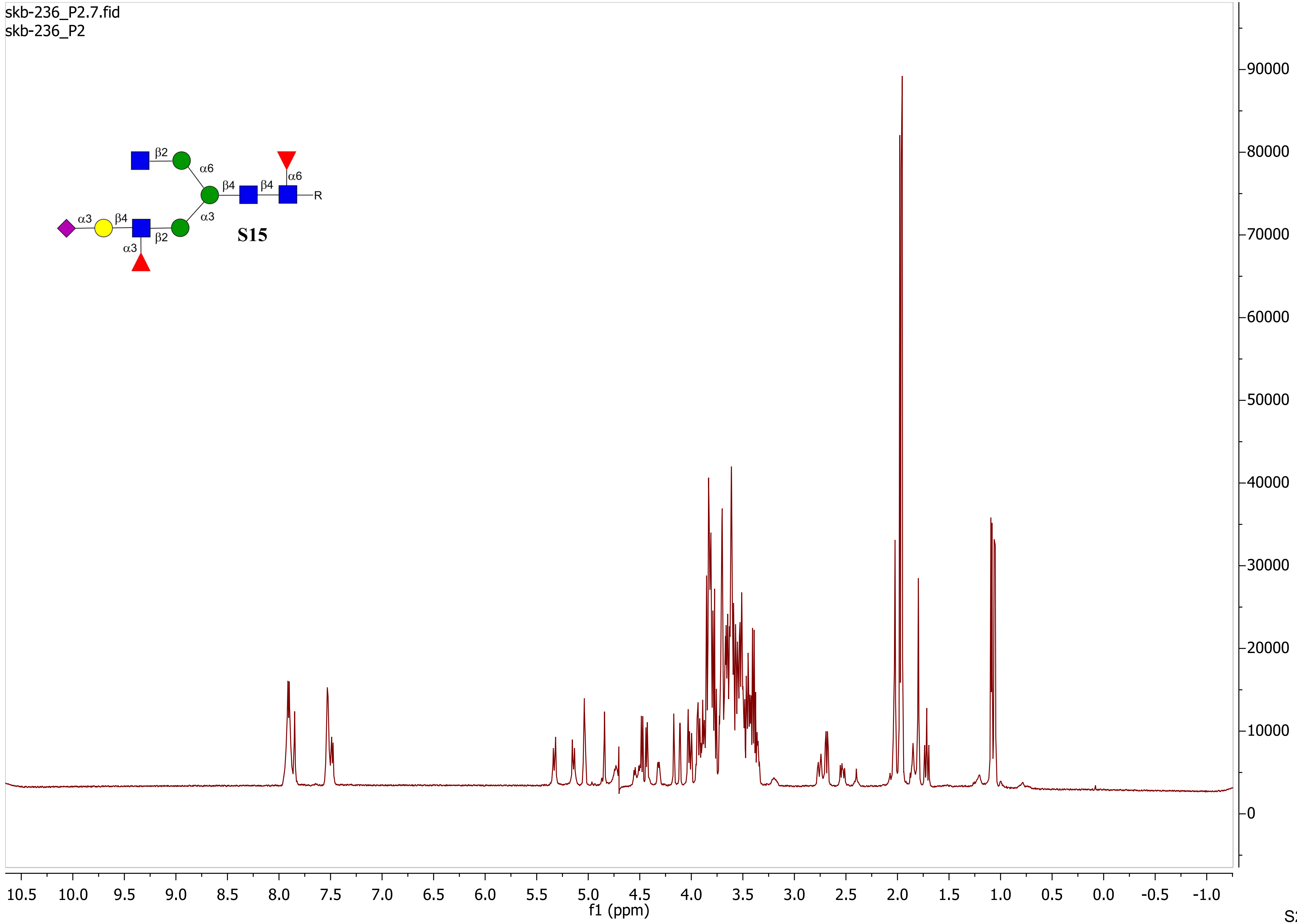
skb-236_P2.7.fid
skb-236_P2
β2
α6
β4
β4
α6
R
α3
β4
α3
β2
α3
S15
90000
80000
70000
60000
50000
40000
30000
20000
10000
0
10.5
10.0
9.5
9.0
8.5
8.0
7.5
7.0
6.5
6.0
5.5
5.0
4.5
4.0
3.5
3.0
2.5
2.0
1.5
1.0
0.5
0.0
-0.5
-1.0
f1 (ppm)

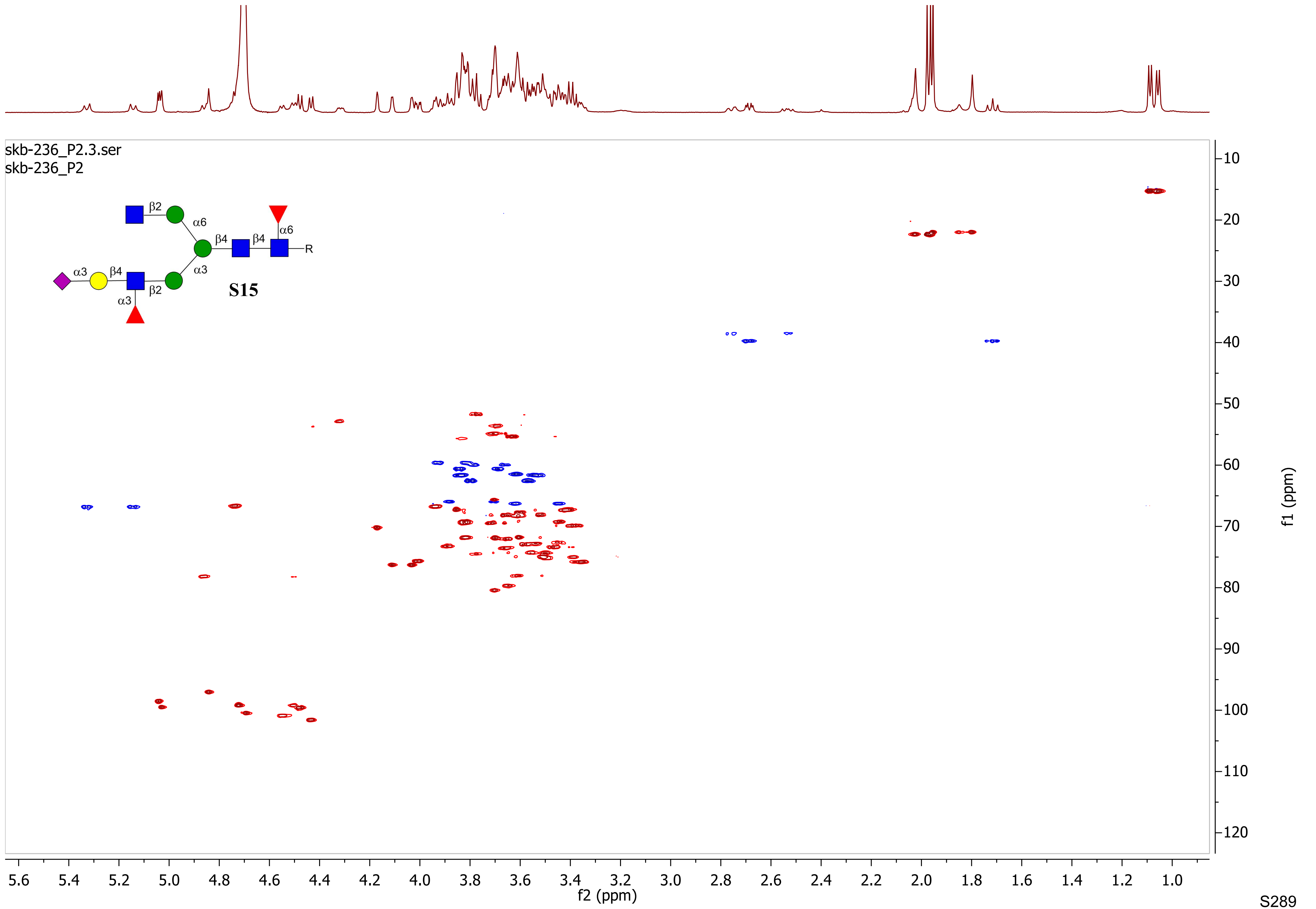

skb-236_P2.3.ser
skb-236_P2
β2
α6
β4
β4
α6
R
α3
β4
α3
β2
α3
S15
10
20
30
40
50
60
70
80
90
100
110
120
f1 (ppm)
5.6
5.4
5.2
5.0
4.8
4.6
4.4
4.2
4.0
3.8
3.6
3.4
3.2
3.0
2.8
2.6
2.4
2.2
2.0
1.8
1.6
1.4
1.2
1.0
f2 (ppm)

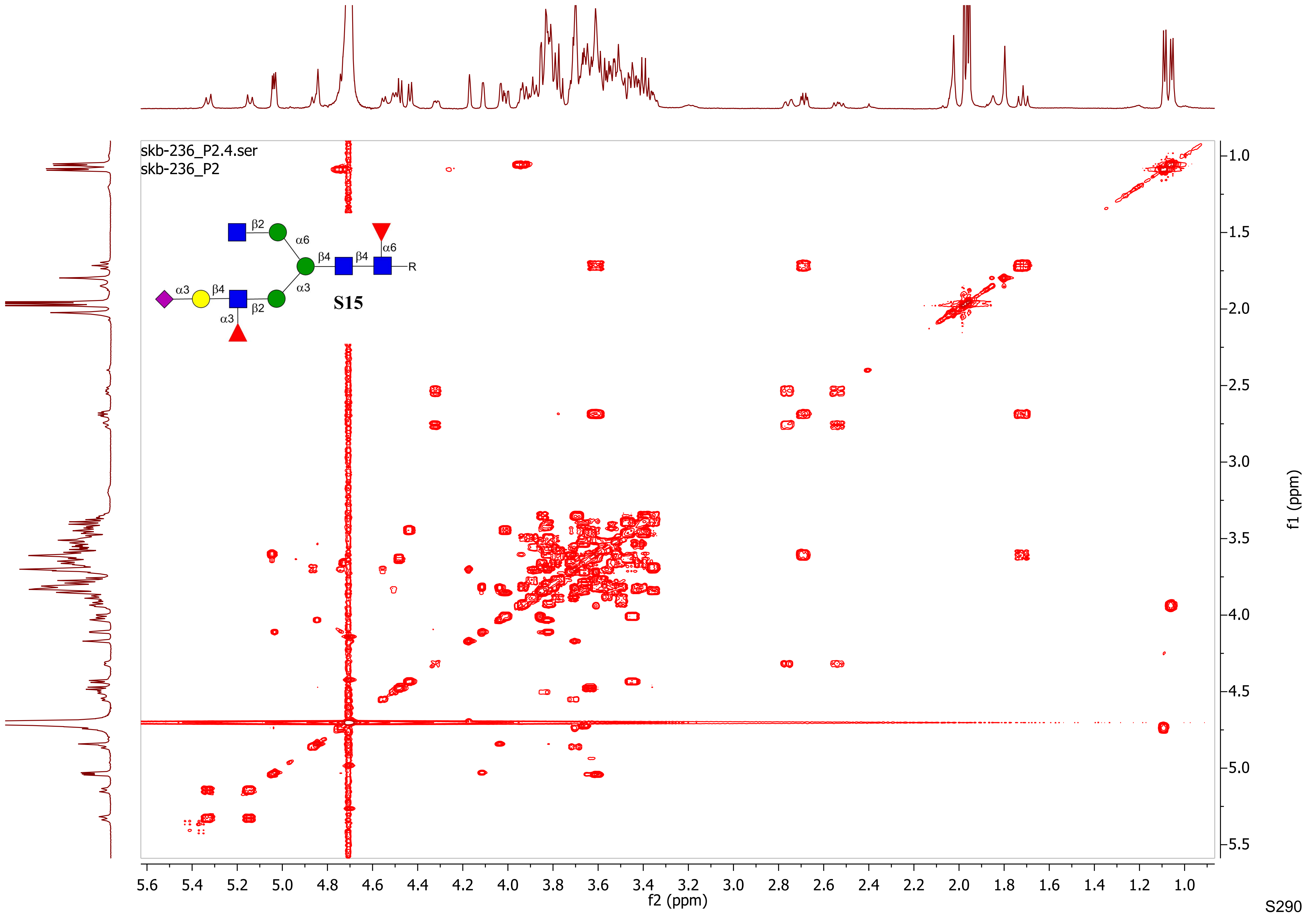
skb-236_P2.4.ser
skb-236_P2
S15
f2 (ppm)
f1 (ppm)

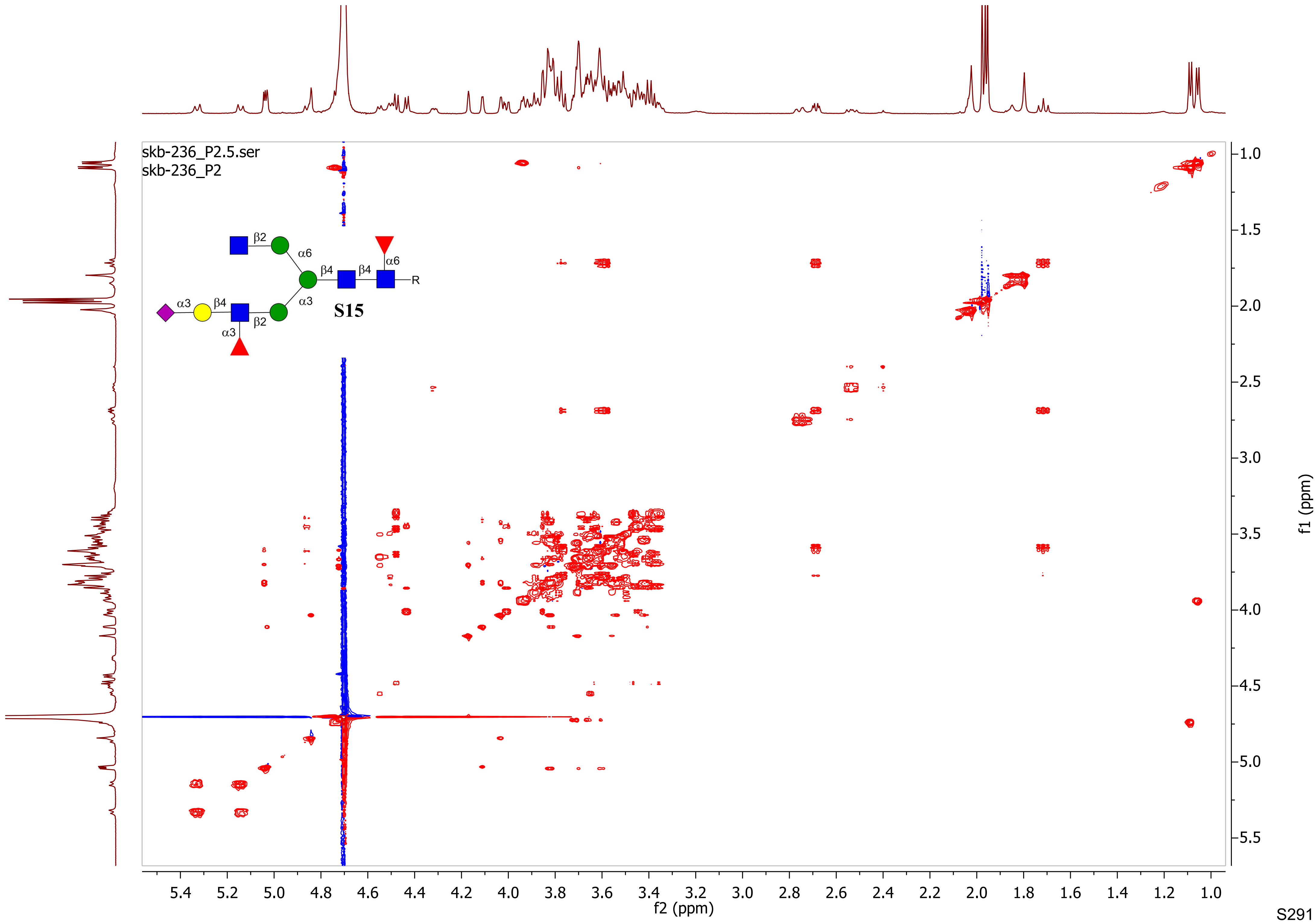
skb-236_P2.5.ser
skb-236_P2
β2
α6
β4
β4
α6
R
α3
β4
α3
β2
α3
S15
f2 (ppm)
f1 (ppm)
5.4
5.2
5.0
4.8
4.6
4.4
4.2
4.0
3.8
3.6
3.4
3.2
3.0
2.8
2.6
2.4
2.2
2.0
1.8
1.6
1.4
1.2
1.0
1.5
2.0
2.5
3.0
3.5
4.0
4.5
5.0
5.5

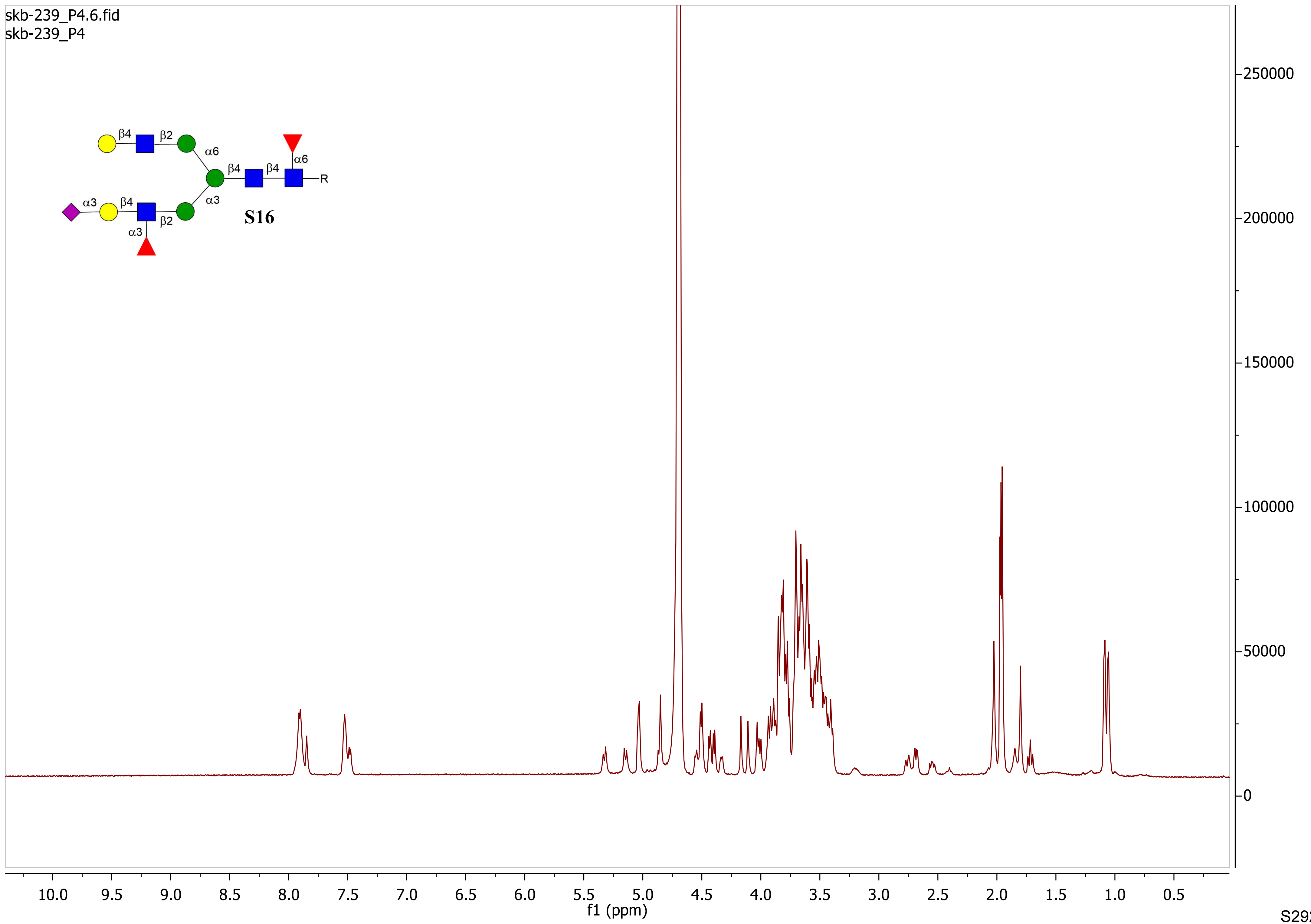
skb-239_P4.6.fid
skb-239_P4
β4
β2
α6
β4
β4
α6
R
α3
β4
α3
β2
α3
S16
250000
200000
150000
100000
50000
0
10.0
9.5
9.0
8.5
8.0
7.5
7.0
6.5
6.0
5.5
5.0
4.5
4.0
3.5
3.0
2.5
2.0
1.5
1.0
0.5
f1 (ppm)

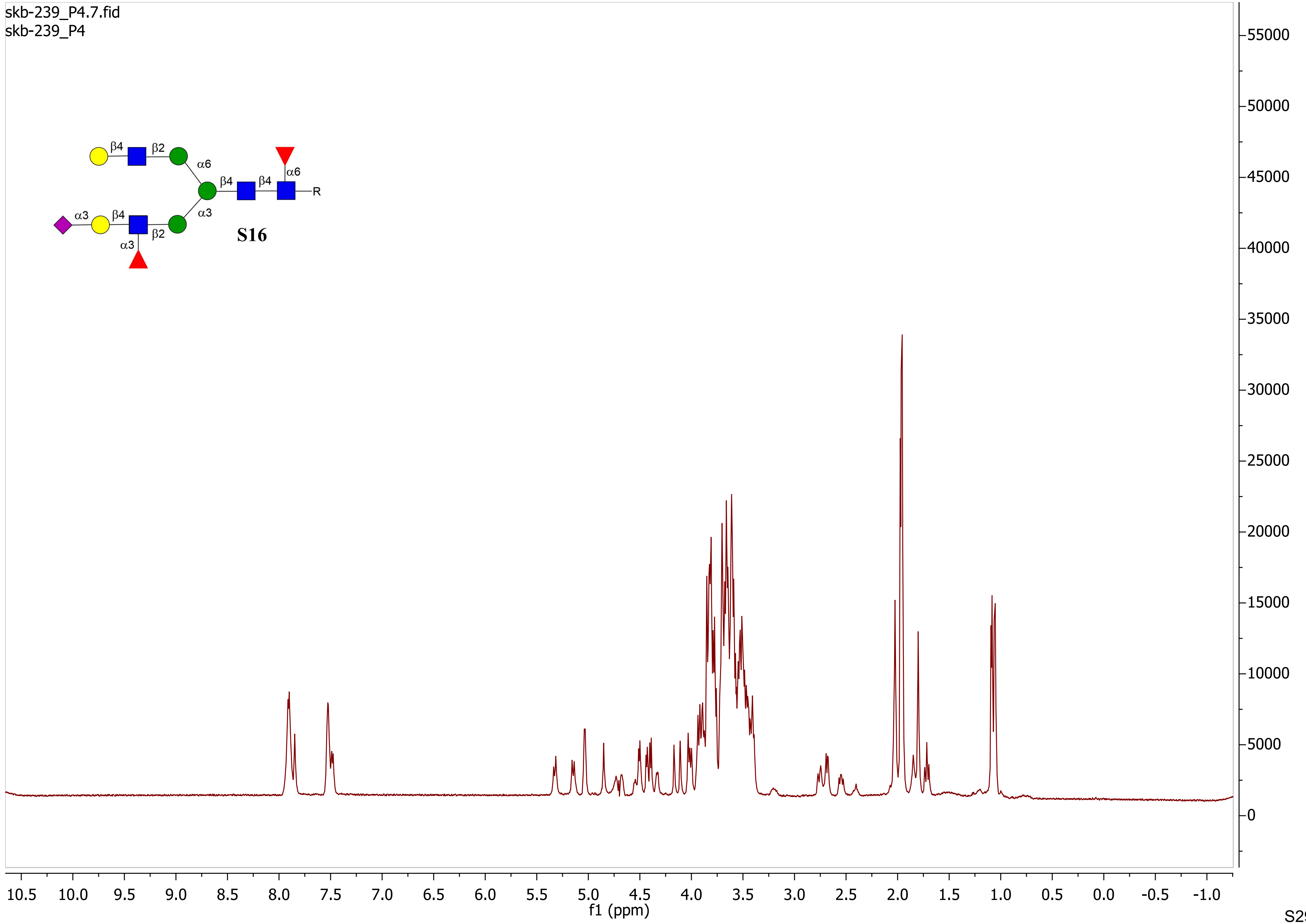

skb-239_P4.7.fid
skb-239_P4
β4
β2
α6
β4
β4
α6
R
α3
β4
α3
β2
α3
S16
f1 (ppm)

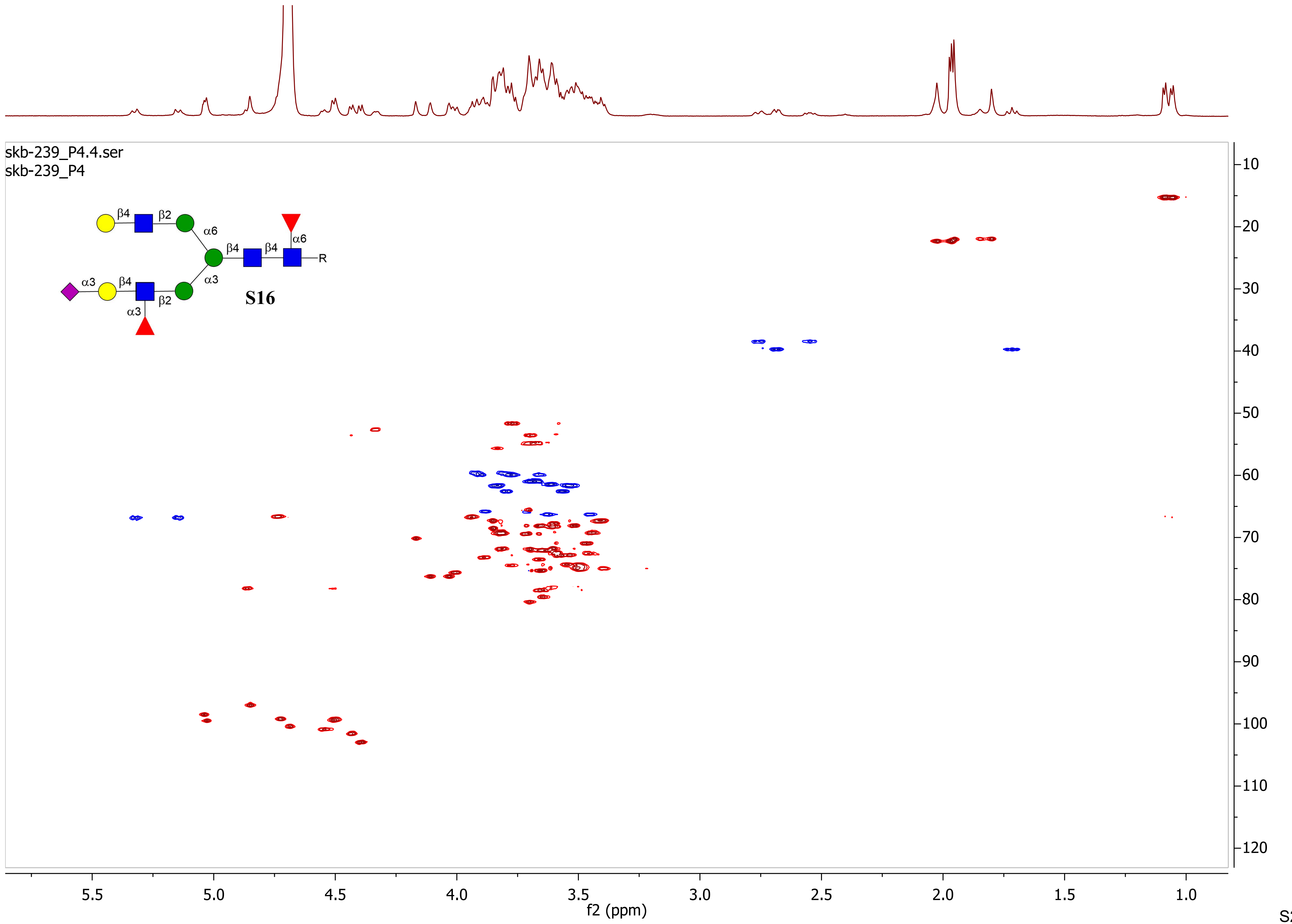

skb-239_P4.4.ser
skb-239_P4
β4
β2
α6
β4
β4
α6
R
α3
β4
α3
β2
α3
S16
f1 (ppm)
f2 (ppm)

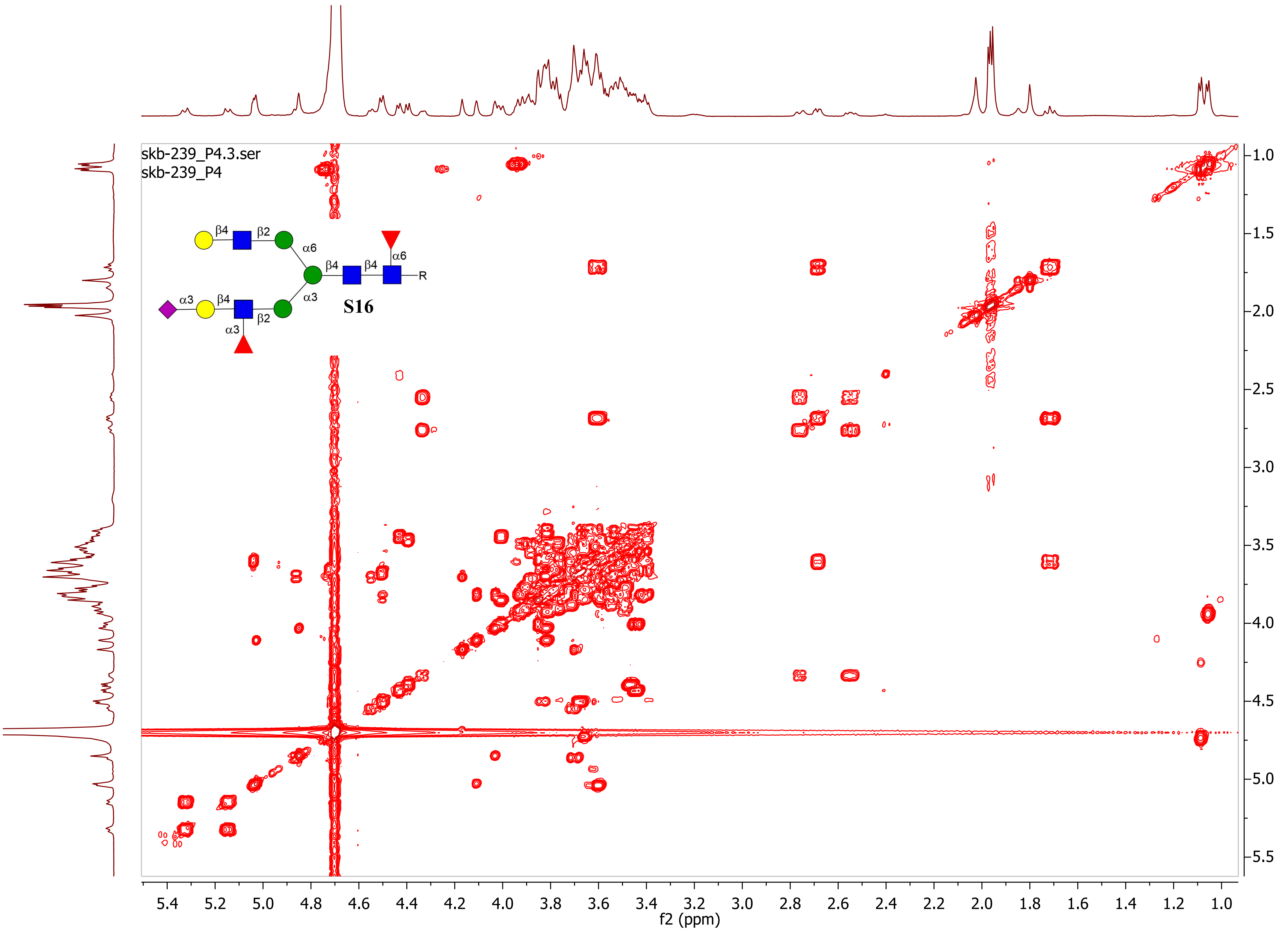
skb-239_P4.3.ser
skb-239_P4
β4
β2
α6
β4
β4
α6
R
α3
β4
α3
β2
α3
S16
f2 (ppm)
f1 (ppm)

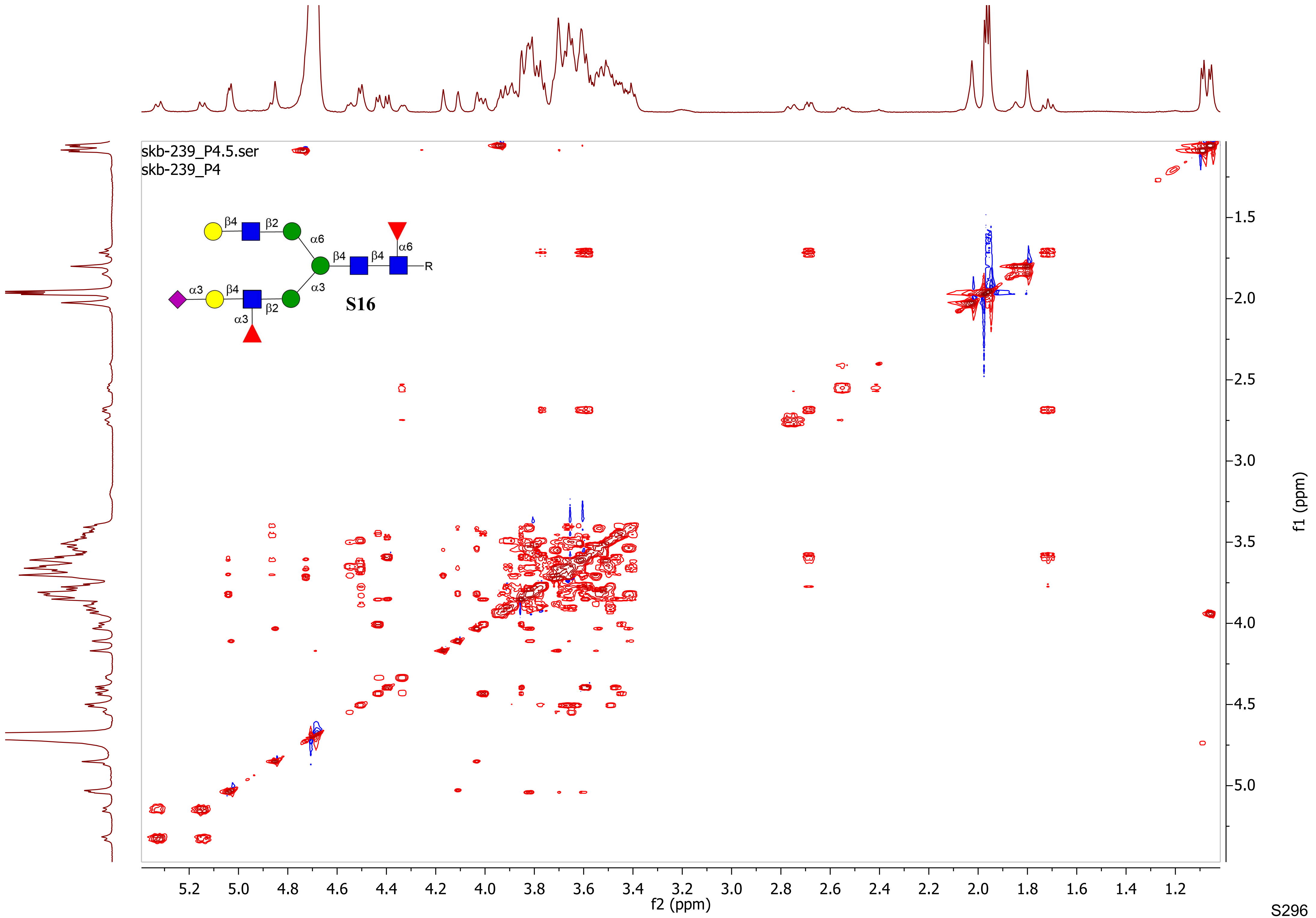

skb-239_P4.5.ser
skb-239_P4
β4
β2
α6
β4
β4
α6
R
α3
β4
α3
β2
α3
S16
f2 (ppm)
f1 (ppm)

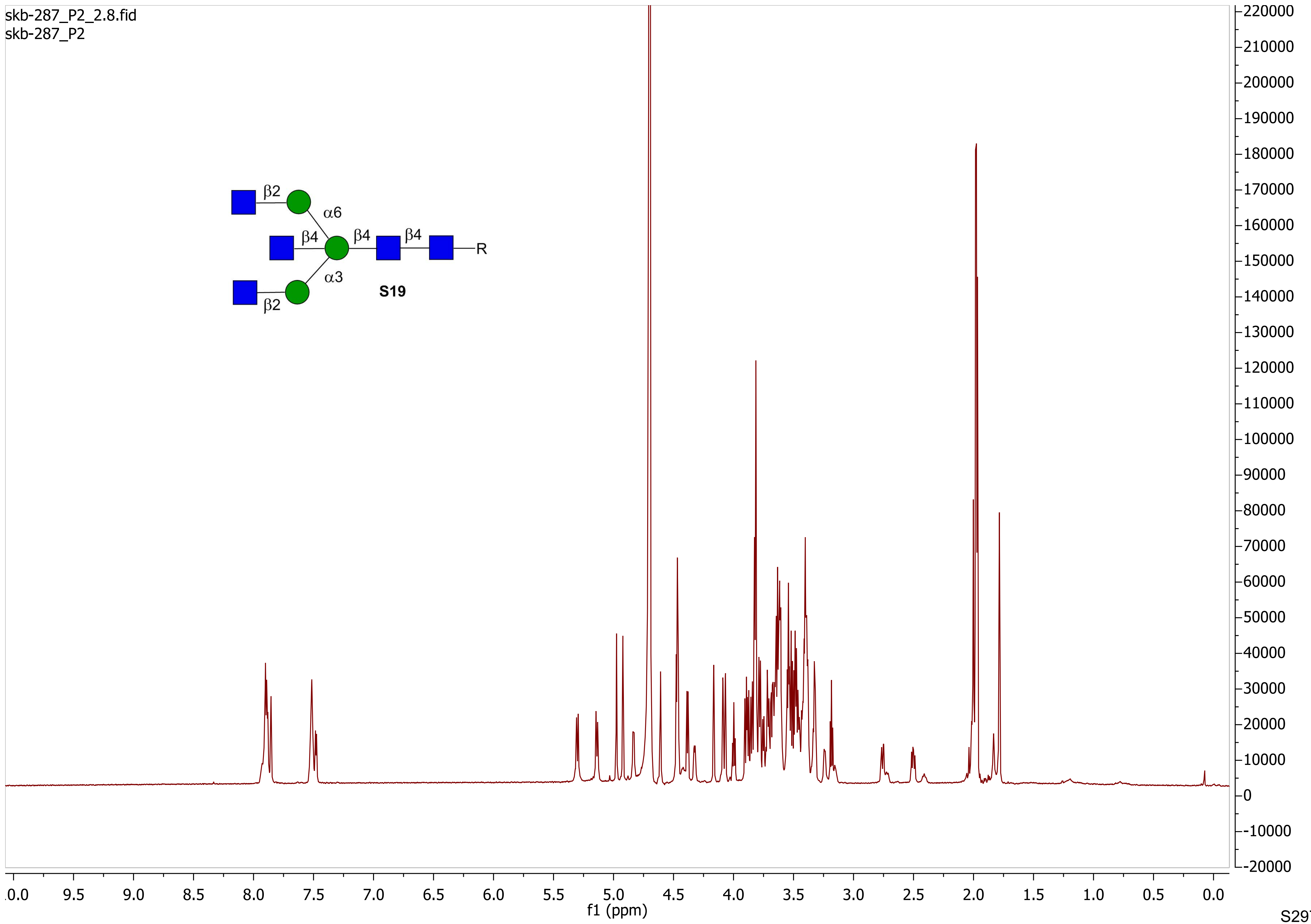
skb-287_P2_2.8.fid
skb-287_P2
β2
α6
β4
β4
β4
R
α3
β2
S19
f1 (ppm)

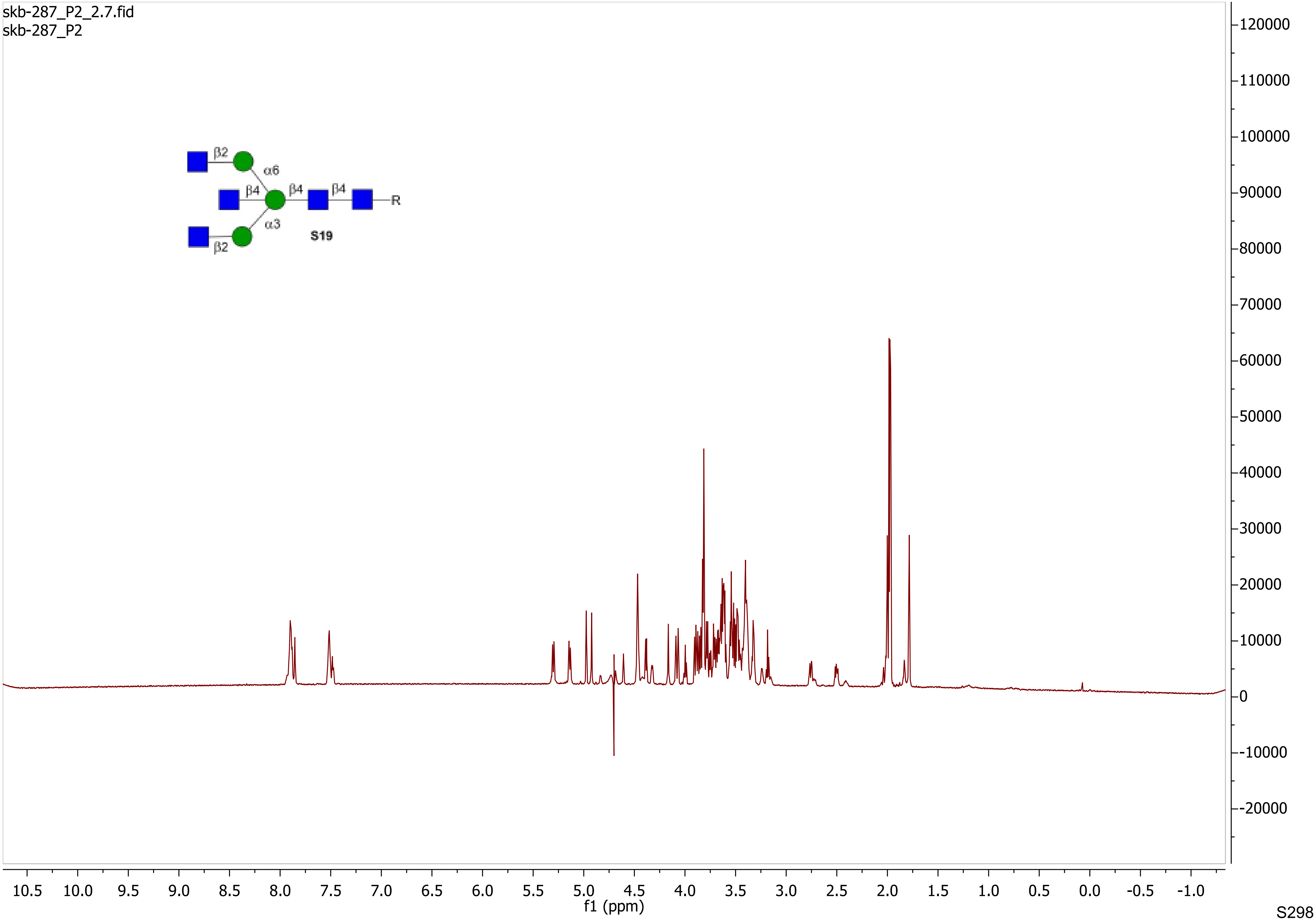
skb-287_P2_2.7.fid
skb-287_P2
β2
α6
β4
β4
β4
R
α3
β2
S19
f1 (ppm)

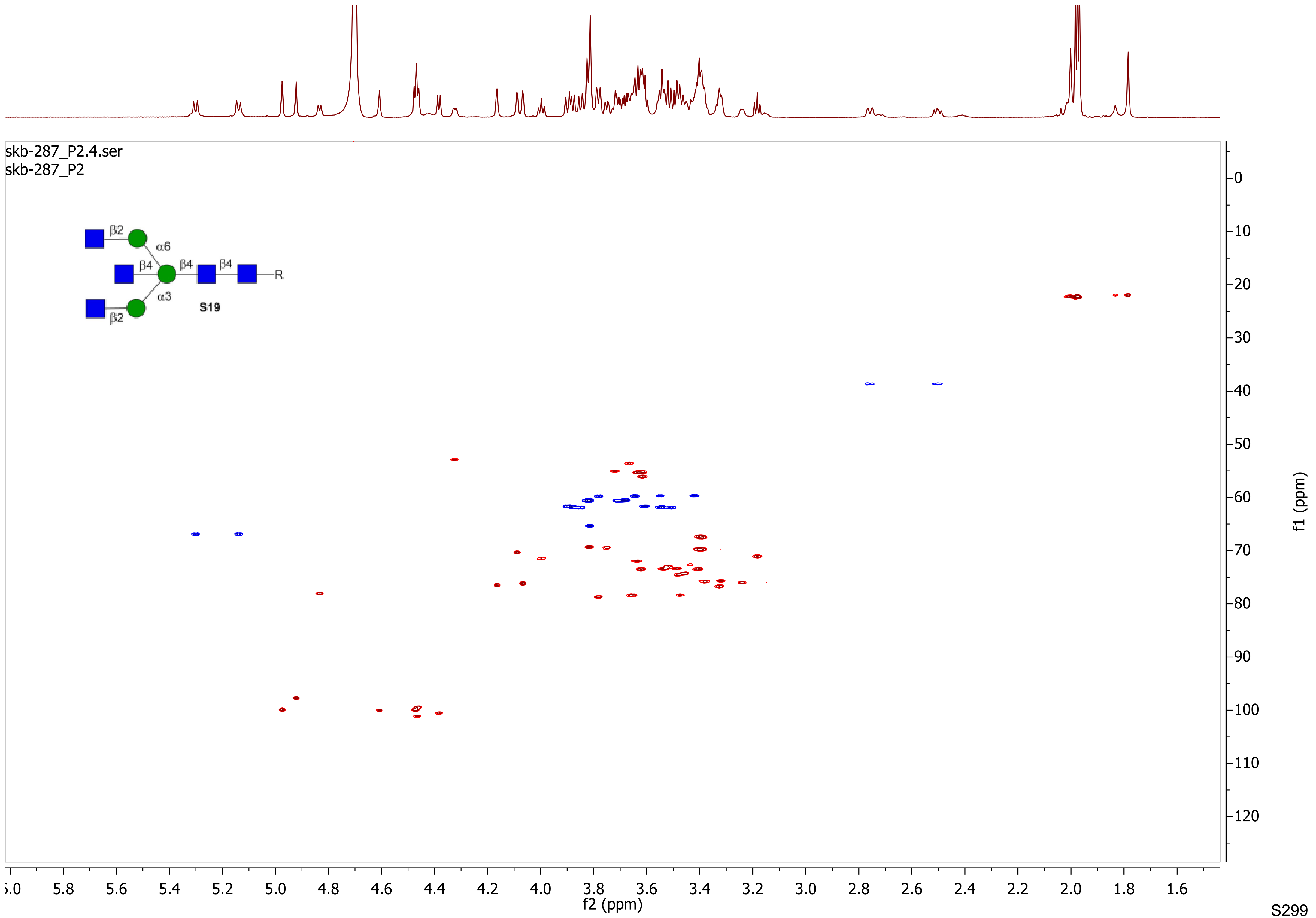
skb-287_P2.4.ser
skb-287_P2
β2
α6
β4
β4
β4
R
α3
S19
β2
f2 (ppm)
f1 (ppm)

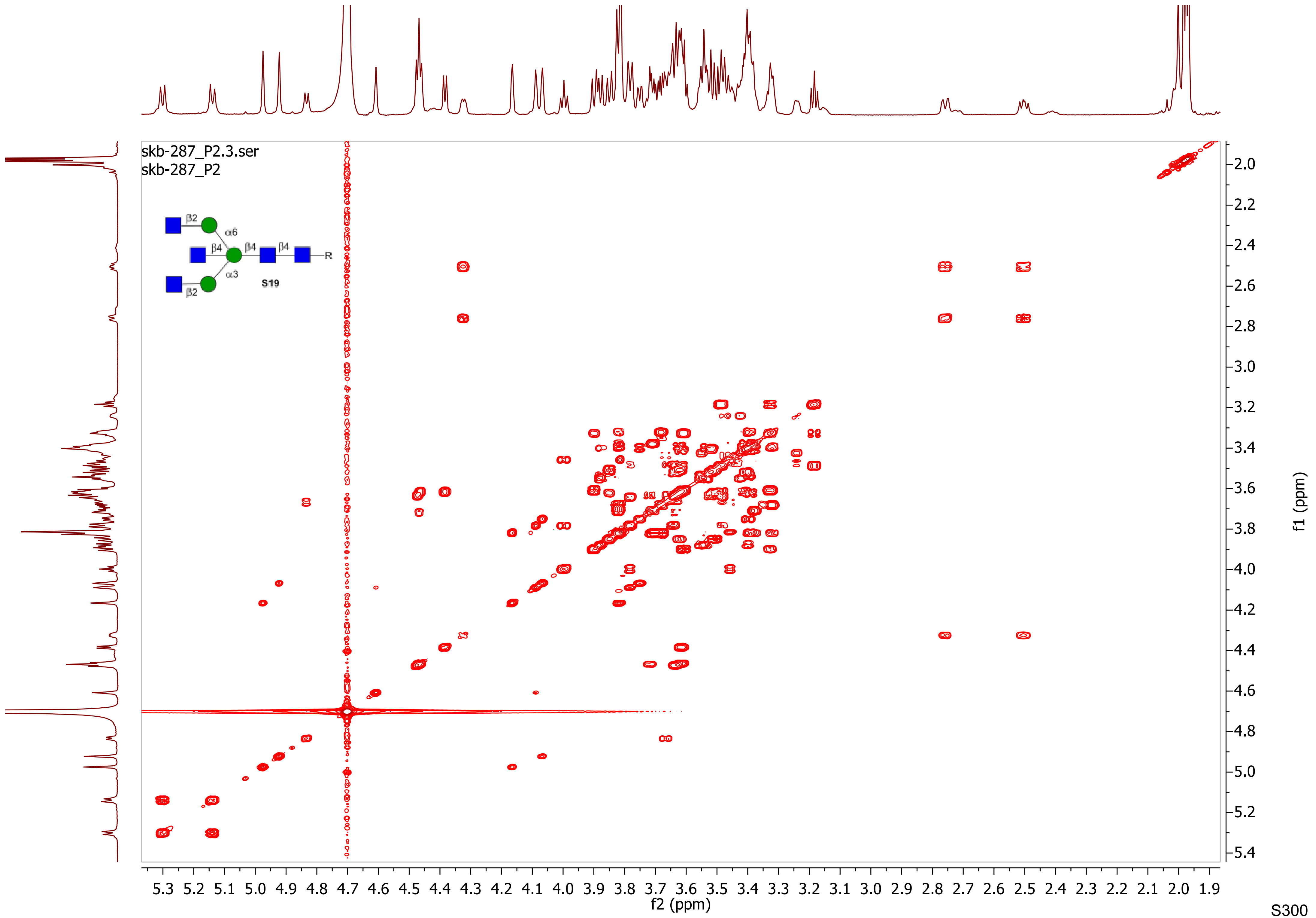
skb-287_P2.3.ser
skb-287_P2
β2
α6
β4
β4
β4
R
α3
β2
S19
f2 (ppm)
f1 (ppm)

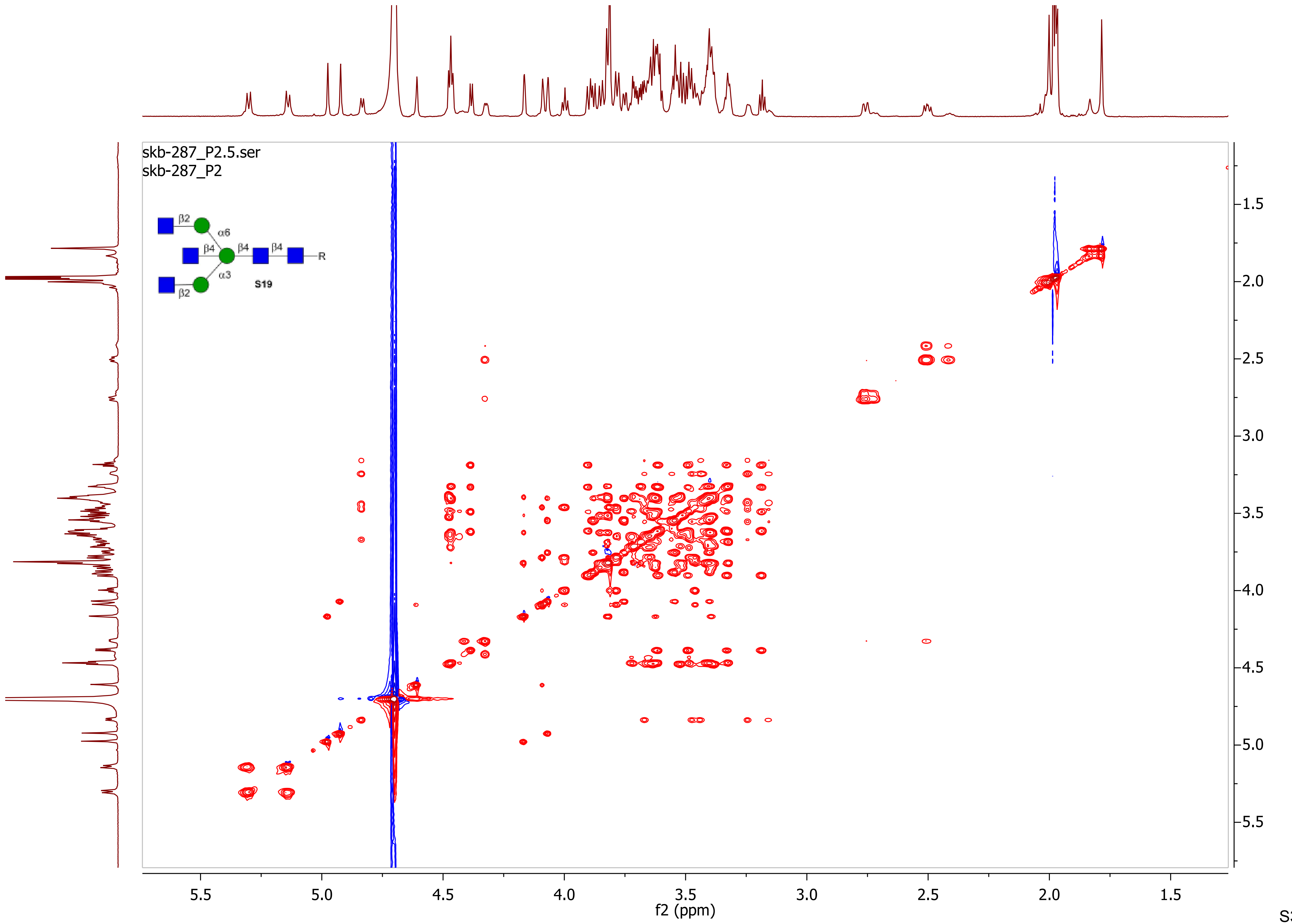
skb-287_P2.5.ser
skb-287_P2
β2
α6
β4
β4
β4
R
α3
β2
S19
f2 (ppm)
f1 (ppm)

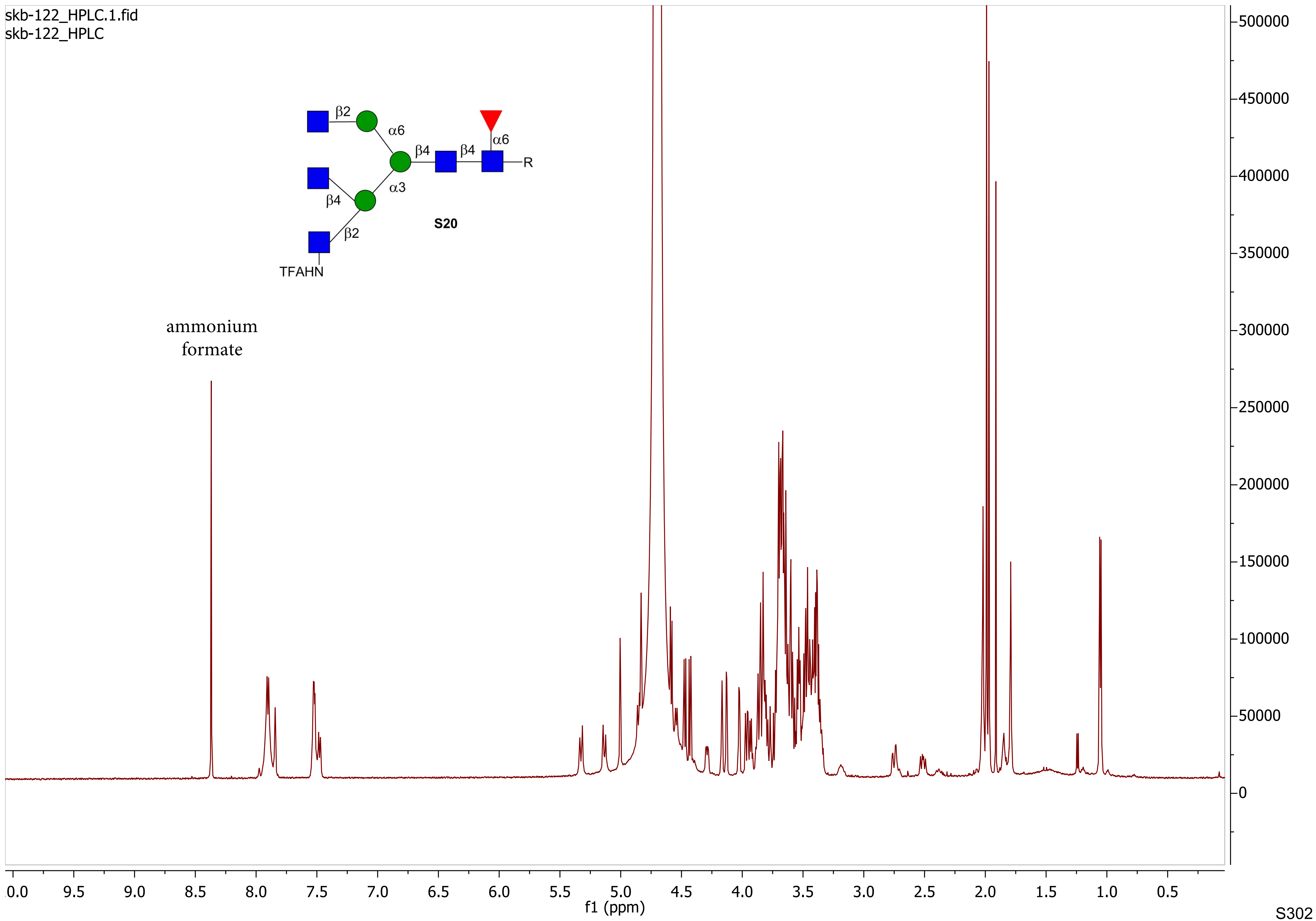
skb-122_HPLC.1.fid
skb-122_HPLC
β2
α6
β4
β4
α6
R
β4
α3
β2
TFAHN
S20
ammonium formate
f1 (ppm)

skb-122_HPLC.2.fid
skb-122_HPLC

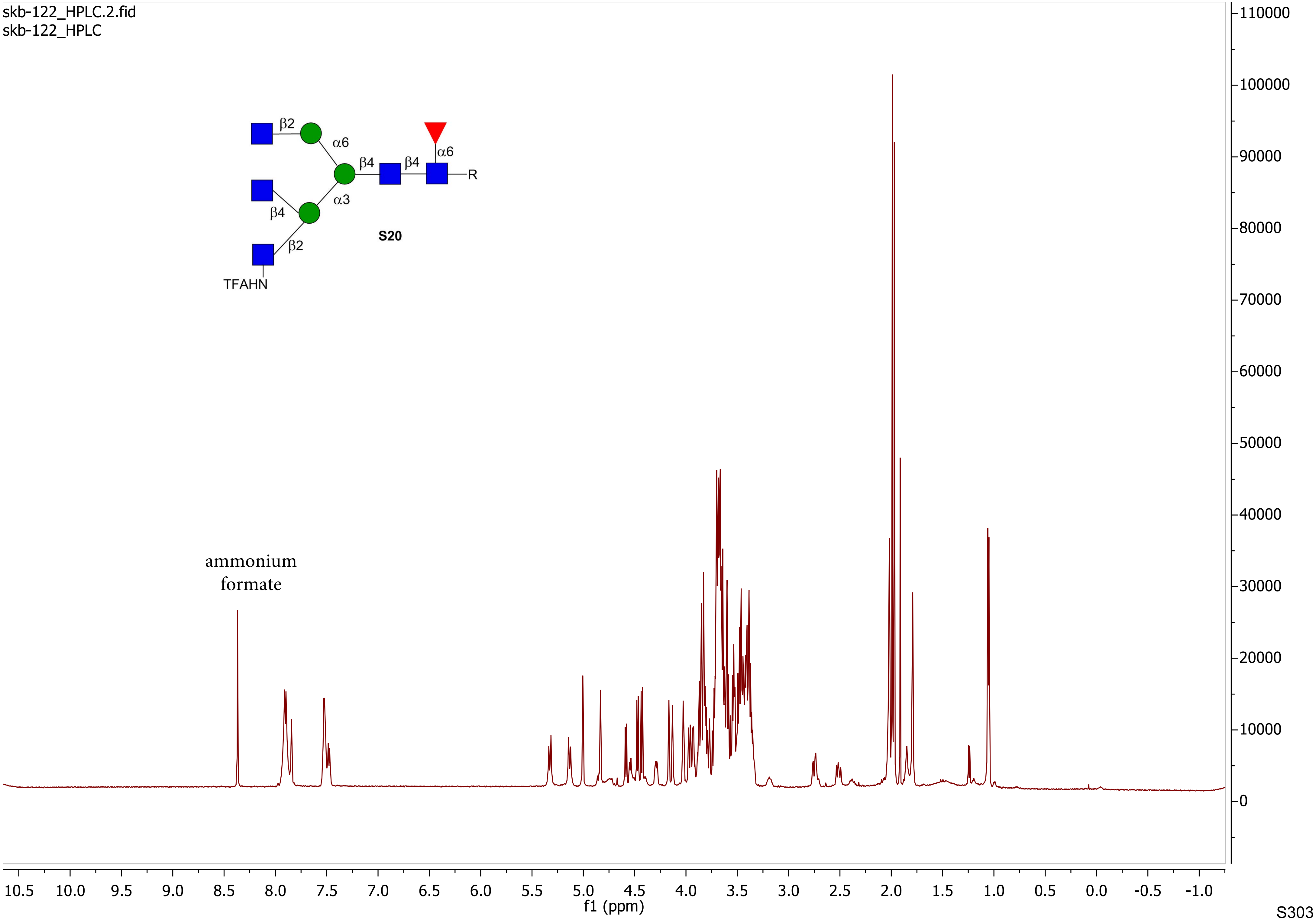

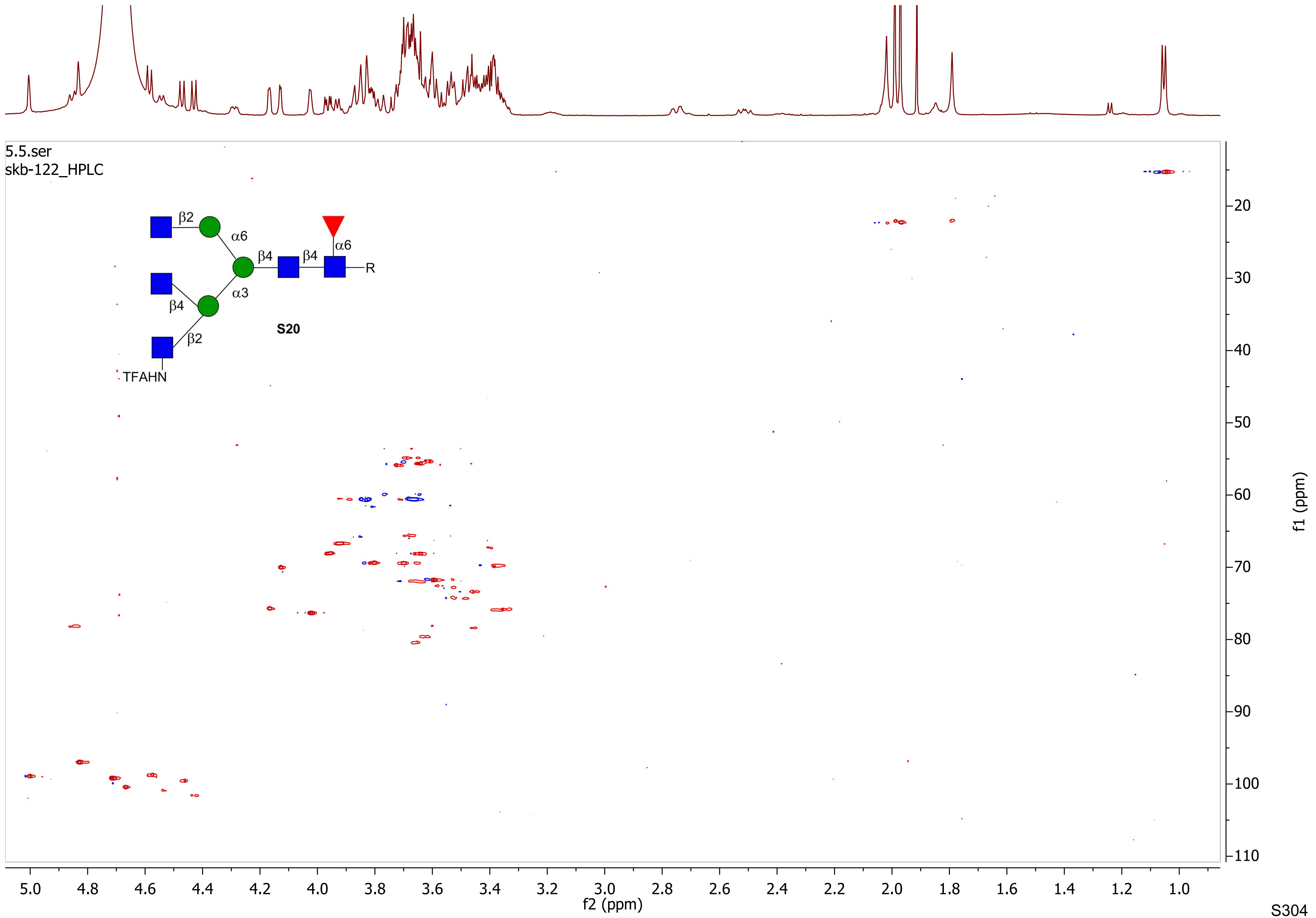

5.5.ser
skb-122_HPLC
β2
α6
β4
β4
α6
R
β4
α3
β2
TFAHN
S20
f2 (ppm)
f1 (ppm)

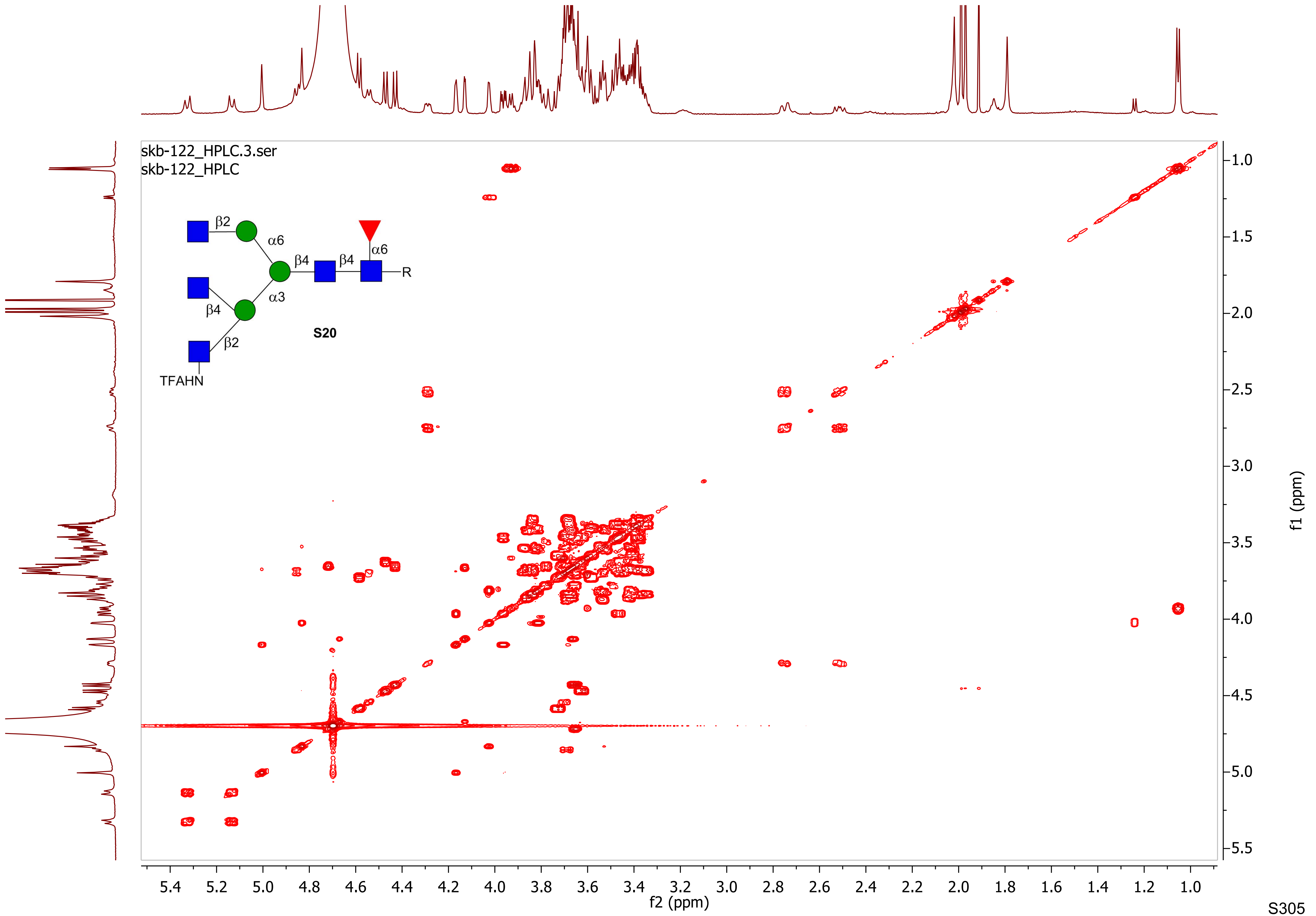

skb-122_HPLC.3.ser
skb-122_HPLC
β2
α6
β4
β4
α6
R
β4
α3
β2
TFAHN
S20
f2 (ppm)
f1 (ppm)

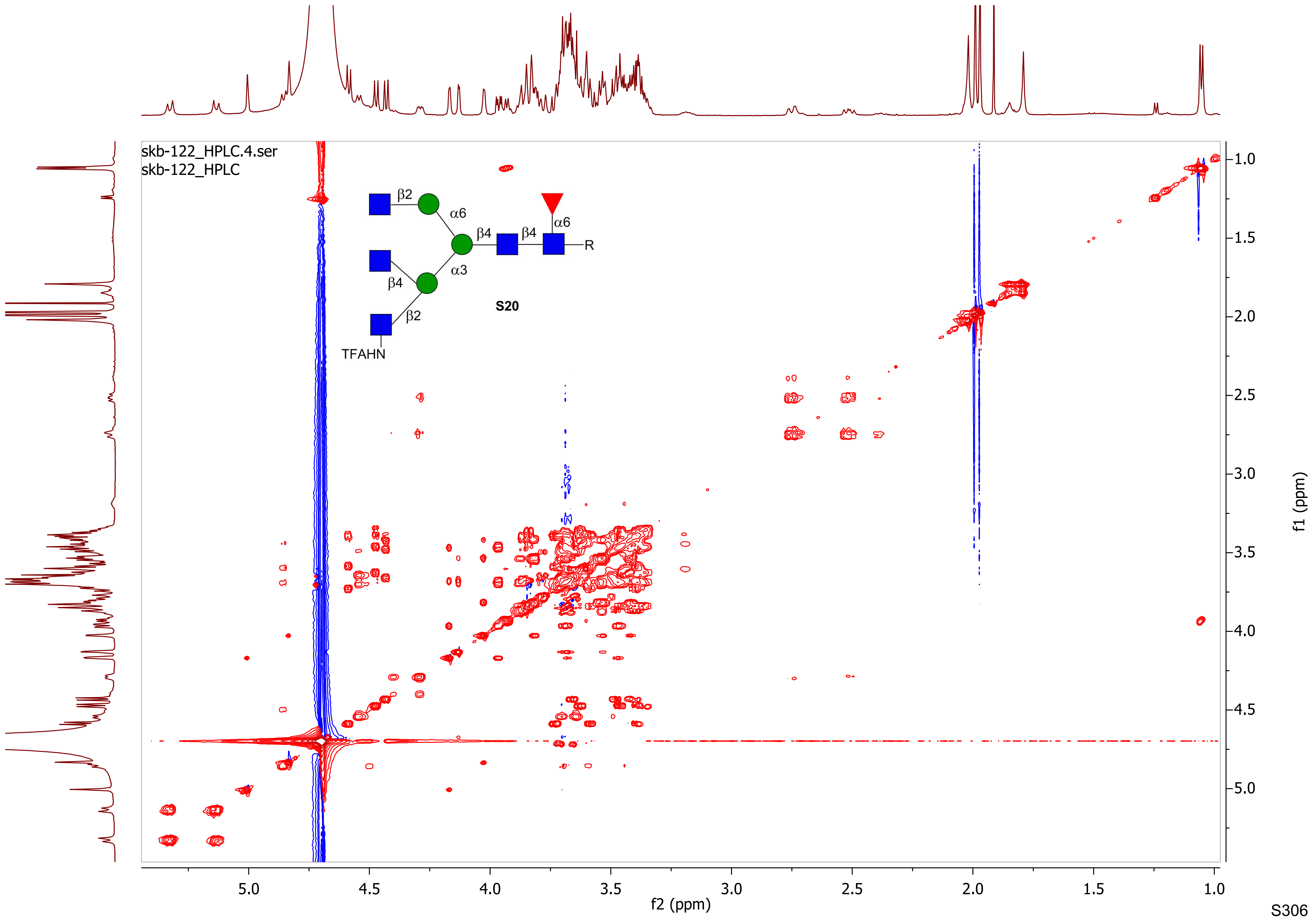

skb-122_HPLC.4.ser
skb-122_HPLC
β2
α6
β4
β4
α6
R
β4
α3
β2
TFAHN
S20
f2 (ppm)
f1 (ppm)

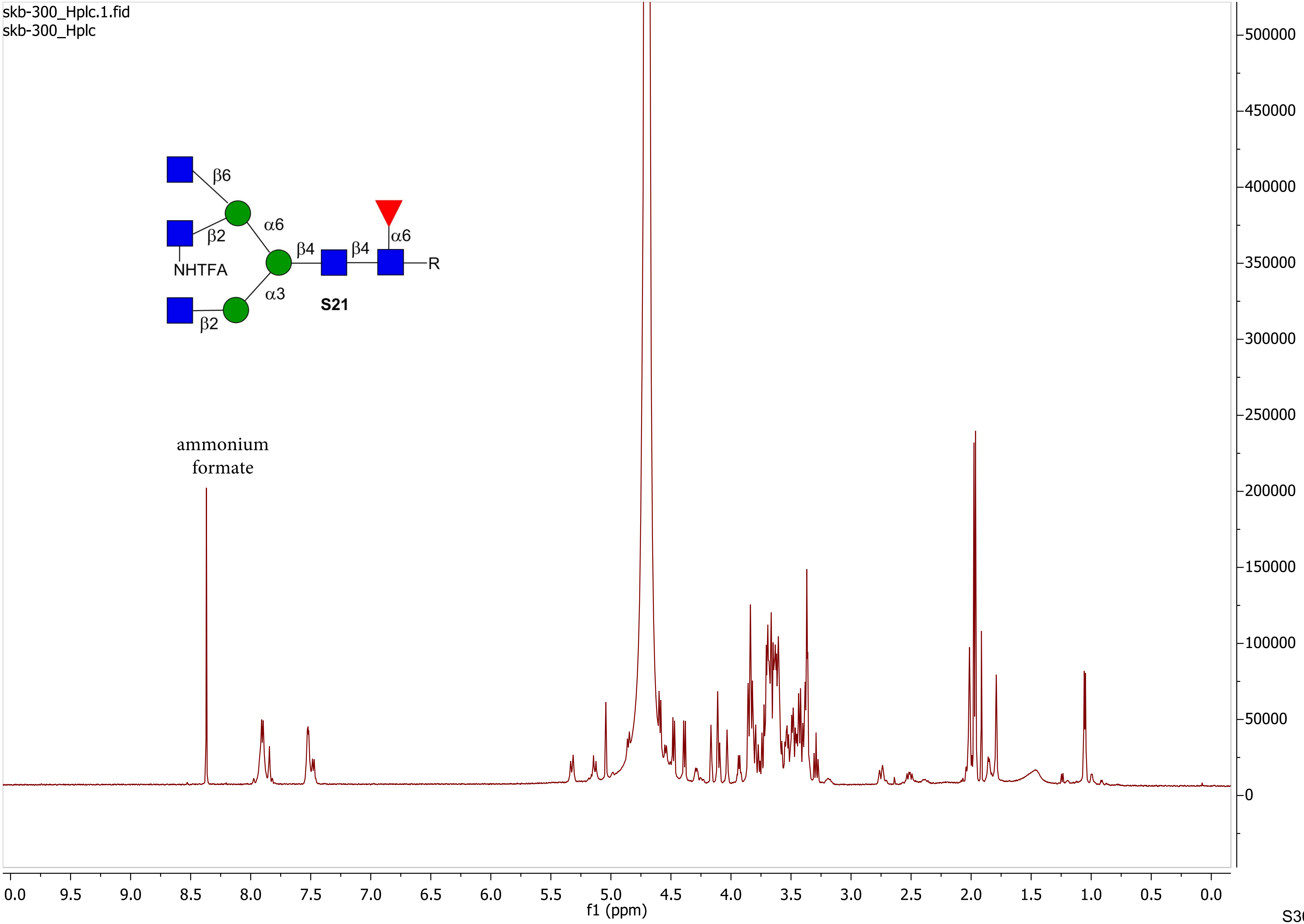
skb-300_Hplc.1.fid
skb-300_Hplc
β6
α6
β2
NHTFA
β4
β4
α6
R
α3
S21
β2
ammonium formate
f1 (ppm)

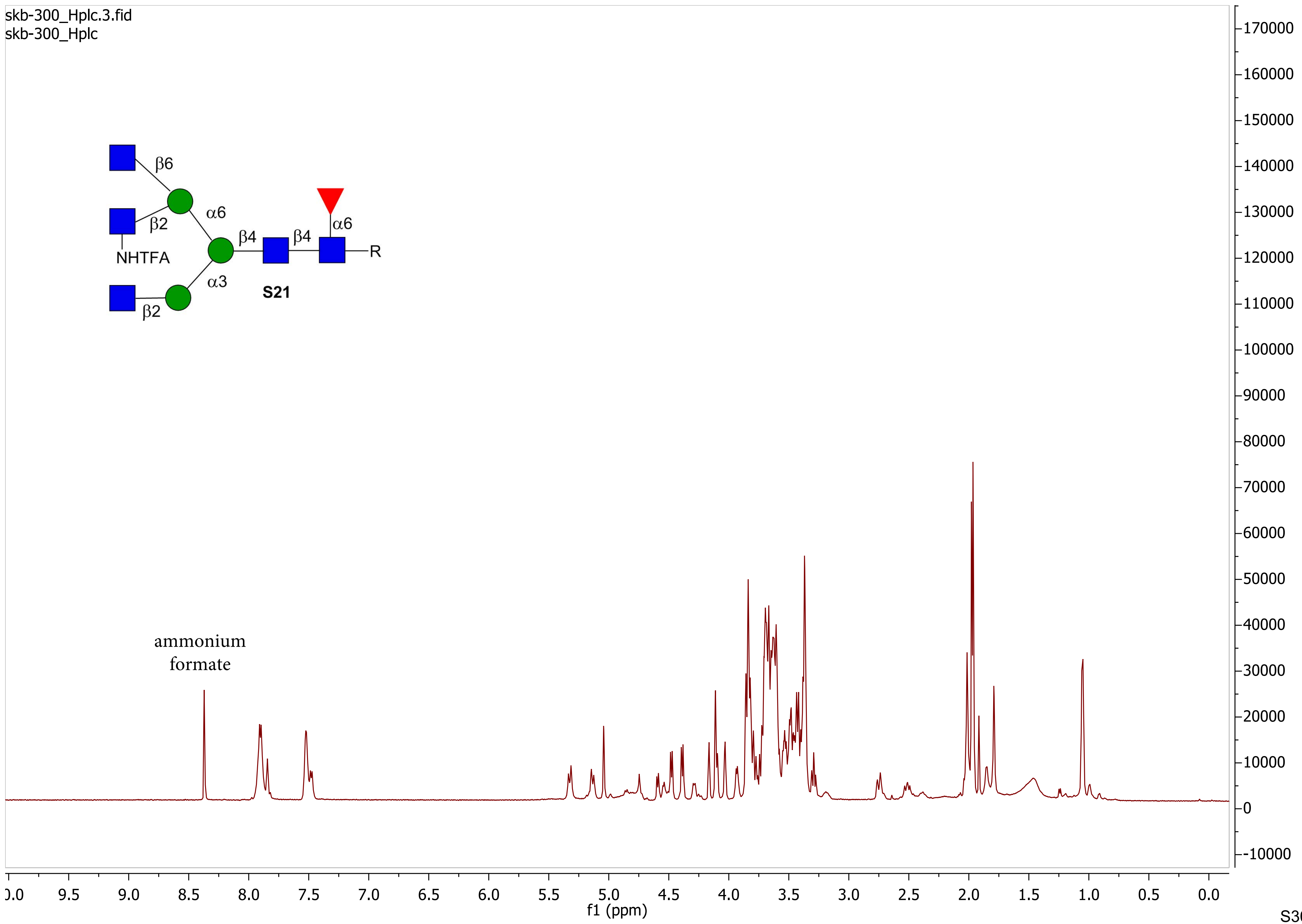
skb-300_Hplc.3.fid
skb-300_Hplc
β6
α6
β2
NHTFA
β4
β4
α6
R
α3
β2
S21
ammonium formate
f1 (ppm)

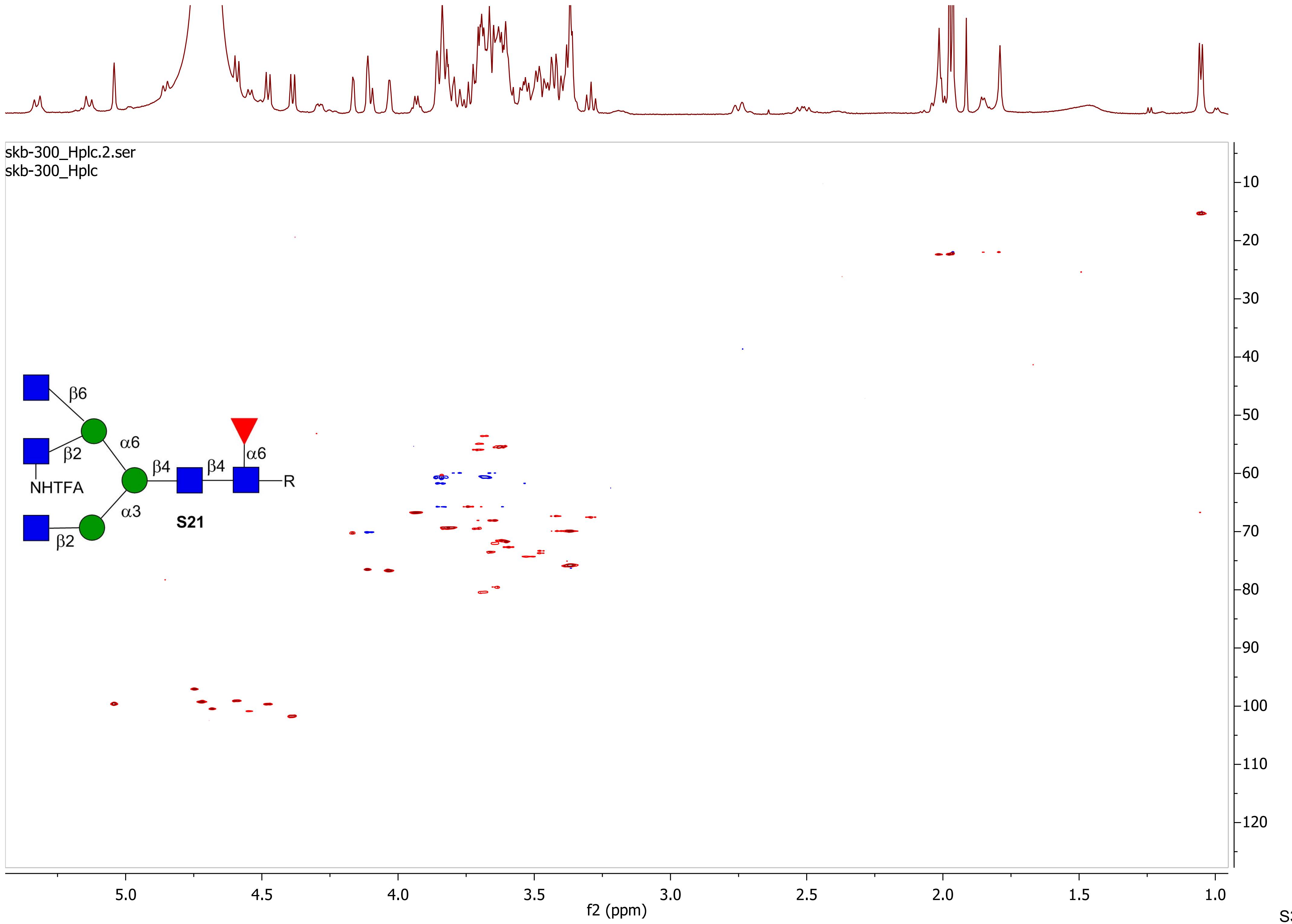
skb-300_Hplc.2.ser
skb-300_Hplc
β6
α6
β2
NHTFA
β4
β4
α6
R
α3
S21
β2
f2 (ppm)
f1 (ppm)

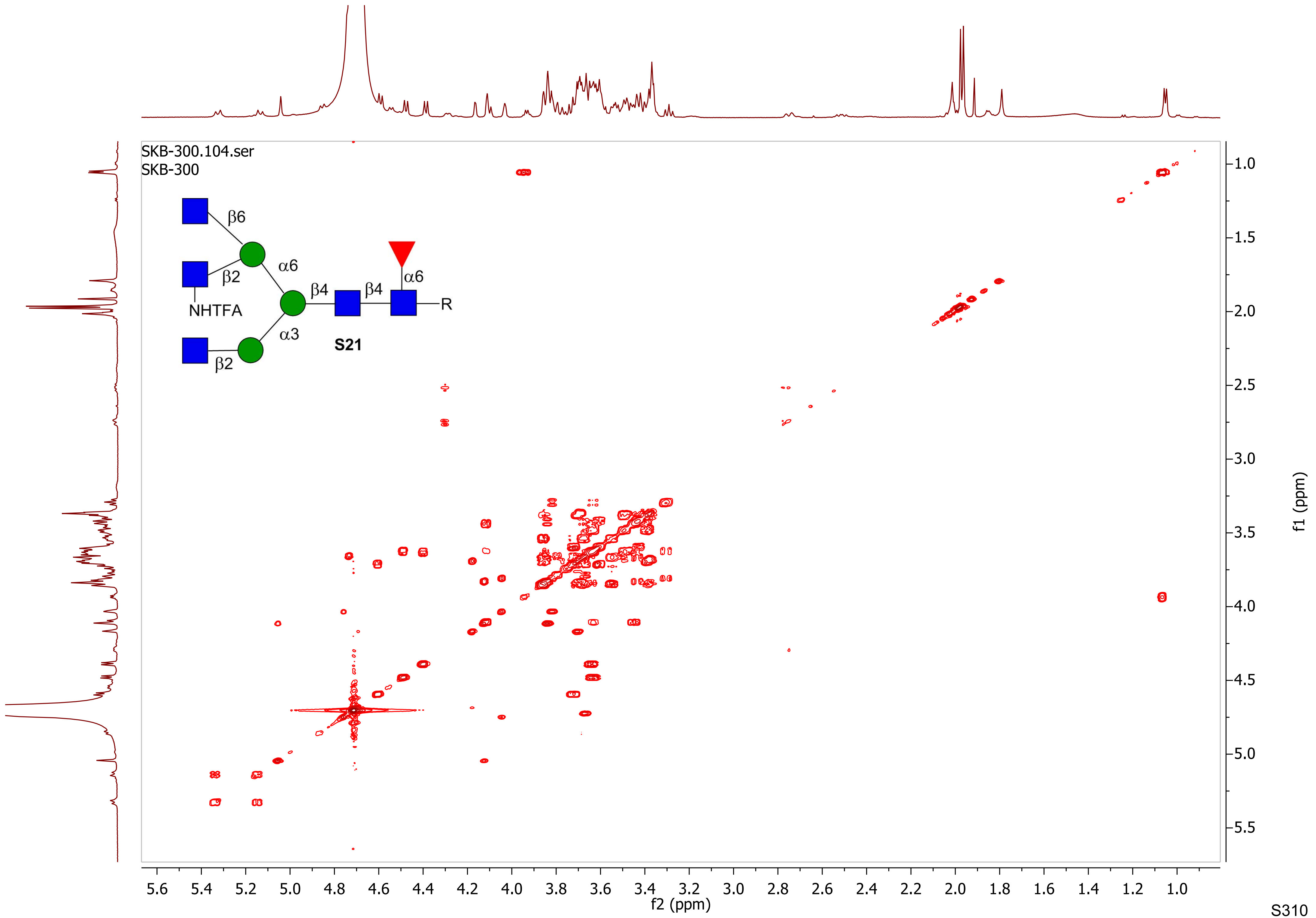
SKB-300.104.ser
SKB-300
β6
β2
NHTFA
α6
β4
β4
α6
R
α3
β2
S21
5.6
5.4
5.2
5.0
4.8
4.6
4.4
4.2
4.0
3.8
3.6
3.4
3.2
3.0
2.8
2.6
2.4
2.2
2.0
1.8
1.6
1.4
1.2
1.0
f2 (ppm)
1.0
1.5
2.0
2.5
3.0
3.5
4.0
4.5
5.0
5.5
f1 (ppm)

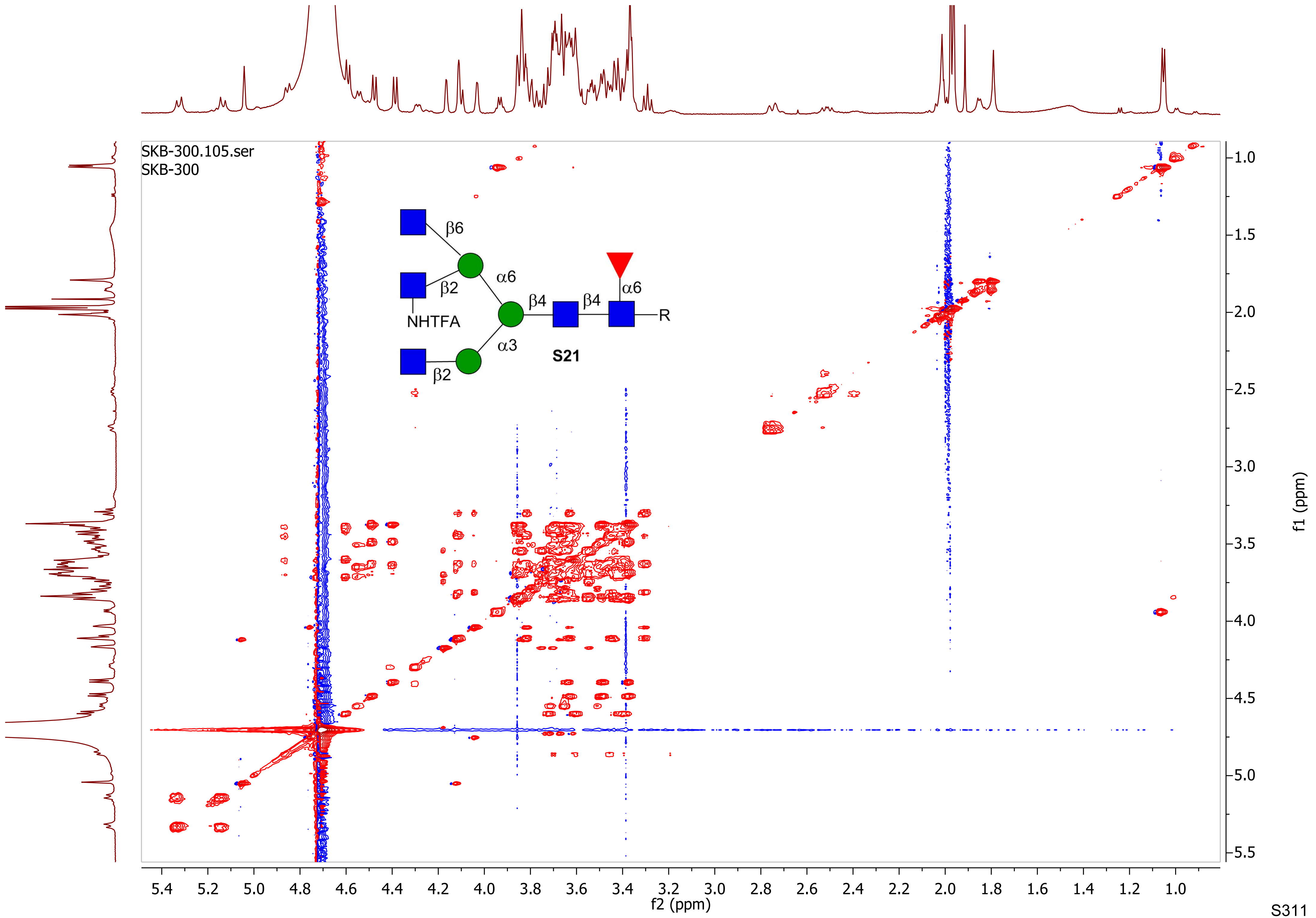

SKB-300.105.ser
SKB-300
β6
β2
α6
β4
β4
α6
NHTFA
R
α3
β2
S21
f2 (ppm)
f1 (ppm)
